# A purified diet affects intestinal epithelial proliferation and barrier functions through gut microbial alterations

**DOI:** 10.1093/intimm/dxae003

**Published:** 2024-01-23

**Authors:** Hiroaki Shiratori, Kisara M Hattori, Kazuaki Nakata, Takuma Okawa, Seiga Komiyama, Yusuke Kinashi, Yuma Kabumoto, Yuria Kaneko, Motoyoshi Nagai, Tomoko Shindo, Nobuko Moritoki, Yuki I Kawamura, Taeko Dohi, Daisuke Takahashi, Shunsuke Kimura, Koji Hase

**Affiliations:** Division of Biochemistry, Department of Pharmaceutical Sciences, Faculty of Pharmacy, and Graduate School of Pharmaceutical Sciences, Keio University, Minato-ku, Tokyo 105-8512, Japan; Clinical Research Advancement Section, Research Institute, National Center for Global Health and Medicine, Tokyo 162-8655, Japan; Division of Biochemistry, Department of Pharmaceutical Sciences, Faculty of Pharmacy, and Graduate School of Pharmaceutical Sciences, Keio University, Minato-ku, Tokyo 105-8512, Japan; Clinical Research Advancement Section, Research Institute, National Center for Global Health and Medicine, Tokyo 162-8655, Japan; Division of Biochemistry, Department of Pharmaceutical Sciences, Faculty of Pharmacy, and Graduate School of Pharmaceutical Sciences, Keio University, Minato-ku, Tokyo 105-8512, Japan; Clinical Research Advancement Section, Research Institute, National Center for Global Health and Medicine, Tokyo 162-8655, Japan; Division of Biochemistry, Department of Pharmaceutical Sciences, Faculty of Pharmacy, and Graduate School of Pharmaceutical Sciences, Keio University, Minato-ku, Tokyo 105-8512, Japan; Division of Biochemistry, Department of Pharmaceutical Sciences, Faculty of Pharmacy, and Graduate School of Pharmaceutical Sciences, Keio University, Minato-ku, Tokyo 105-8512, Japan; Division of Biochemistry, Department of Pharmaceutical Sciences, Faculty of Pharmacy, and Graduate School of Pharmaceutical Sciences, Keio University, Minato-ku, Tokyo 105-8512, Japan; Division of Biochemistry, Department of Pharmaceutical Sciences, Faculty of Pharmacy, and Graduate School of Pharmaceutical Sciences, Keio University, Minato-ku, Tokyo 105-8512, Japan; Clinical Research Advancement Section, Research Institute, National Center for Global Health and Medicine, Tokyo 162-8655, Japan; Division of Biochemistry, Department of Pharmaceutical Sciences, Faculty of Pharmacy, and Graduate School of Pharmaceutical Sciences, Keio University, Minato-ku, Tokyo 105-8512, Japan; Clinical Research Advancement Section, Research Institute, National Center for Global Health and Medicine, Tokyo 162-8655, Japan; Electron Microscope Laboratory, Keio University School of Medicine, Tokyo 160-8582, Japan; Electron Microscope Laboratory, Keio University School of Medicine, Tokyo 160-8582, Japan; Clinical Research Advancement Section, Research Institute, National Center for Global Health and Medicine, Tokyo 162-8655, Japan; Division of Biochemistry, Department of Pharmaceutical Sciences, Faculty of Pharmacy, and Graduate School of Pharmaceutical Sciences, Keio University, Minato-ku, Tokyo 105-8512, Japan; Division of Biochemistry, Department of Pharmaceutical Sciences, Faculty of Pharmacy, and Graduate School of Pharmaceutical Sciences, Keio University, Minato-ku, Tokyo 105-8512, Japan; Division of Biochemistry, Department of Pharmaceutical Sciences, Faculty of Pharmacy, and Graduate School of Pharmaceutical Sciences, Keio University, Minato-ku, Tokyo 105-8512, Japan; Division of Biochemistry, Department of Pharmaceutical Sciences, Faculty of Pharmacy, and Graduate School of Pharmaceutical Sciences, Keio University, Minato-ku, Tokyo 105-8512, Japan; The Institute of Fermentation Sciences (IFeS), Faculty of Food and Agricultural Sciences, Fukushima University, Kanayagawa, Fukushima 960-1296, Japan; International Research and Development Centre for Mucosal Vaccines, The Institute of Medical Science, The University of Tokyo (IMSUT), Tokyo 108-8639, Japan

**Keywords:** epithelial barrier function, epithelial metabolism, gut microbiota, segmented filamentous bacteria

## Abstract

The gut microbiota plays a crucial role in maintaining epithelial barrier function. Although multiple studies have demonstrated the significance of dietary factors on the gut microbiota and mucosal barrier function, the impact of a purified diet, which has long been used in various animal experiments, on intestinal homeostasis remains to be elucidated. Here, we compared the impact of two different types of diets, a crude diet and an AIN-93G-formula purified diet, on epithelial integrity and the gut microbiota. Purified diet-fed mice exhibited shorter villi and crypt lengths and slower epithelial turnover, particularly in the ileum. In addition, antimicrobial products, including REG3γ, were substantially decreased in purified diet-fed mice. Purified diet feeding also suppressed α1,2-fucosylation on the epithelial surface. Furthermore, the purified diet induced metabolic rewiring to fatty acid oxidation and ketogenesis. 16S ribosomal RNA gene sequencing of the ileal contents and mucus layer revealed distinct gut microbiota compositions between the purified and crude diet-fed mice. Purified diet feeding reduced the abundance of segmented filamentous bacteria (SFB), which potently upregulate REG3γ and fucosyltransferase 2 (Fut2) by stimulating group 3 innate lymphoid cells (ILC3s) to produce IL-22. These observations illustrate that the intake of a crude diet secures epithelial barrier function by facilitating SFB colonization, whereas a purified diet insufficiently establishes the epithelial barrier, at least partly owing to the loss of SFB. Our data suggest that the influence of purified diets on the epithelial barrier integrity should be considered in experiments using purified diets.

## Teaser

How a purified diet suppresses epithelial barrier functions

## Graphical Abstract

**Graphical Abstract:**
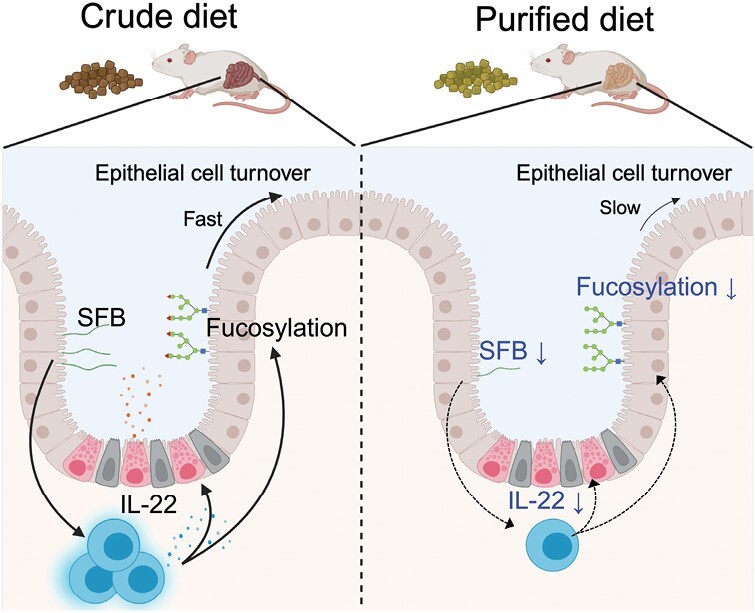

## Introduction

The intestine not only digests and absorbs nutrients but also establishes barrier machinery to prevent invasion by foreign substances. For example, epithelial cells serve as physical barriers by forming tight junctions (1). In addition, goblet cells produce mucins to prevent the adhesion of foreign substances to the epithelium, and various transmembrane glycans, termed the glycocalyx, are expressed on the apical surface of intestinal epithelial cells (2). Within crypts, Paneth cells secrete antimicrobial proteins such as cryptdins and lysozyme (3), which constitute a chemical barrier against harmful microorganisms. Moreover, vigorous epithelial cell turnover (approximately 3–5 days) (4) is a barrier that constantly replaces infected and physically injured cells (5, 6). Thus, the intestinal epithelium forms a complex and redundant barrier machinery to prevent the infiltration of food antigens, foodborne pathogens, and commensal microorganisms into the body. Increased intestinal permeability following epithelial barrier dysfunction often leads to leaky gut syndrome, which is implicated in various systemic disorders (7) such as autoimmune hepatitis (8), rheumatoid arthritis (9), and multiple sclerosis (10). These facts demonstrate that epithelial barrier function significantly contributes to the maintenance of biological homeostasis.

Increasing evidence has shown that the intestinal microbiota is crucial for regulating epithelial barrier function. For instance, the intestinal microbiota promotes α1,2-fucosylation of epithelial glycans, which prevents enteric infections, by inducing IL-22 production from group 3 innate lymphoid cells (ILC3s) in the ileum (11, 12). In addition, microbiota-associated molecular patterns, such as flagellin and lipopolysaccharide, facilitate the production and secretion of mucus (13) and antimicrobial products (such as REG3γ and lipocalin 2) (14, 15). Therefore, germ-free mice exhibit a thinner mucus layer (16) and downregulation of antimicrobial products (17). An imbalance in the gut microbiota (dysbiosis) also disrupts the epithelial barrier integrity by suppressing tight junction (7) and increasing mucus permeability (18). These observations highlight the importance of commensal bacteria in maintaining the host epithelial barrier functions (14).

The diet–microbiota axis is vital for the regulation of epithelial barrier function. Dietary fiber is fermented and metabolized by commensal bacteria into short-chain fatty acids (SCFAs) (such as acetate, propionate, and butyrate) and lactate (19, 20). Butyrate promotes MUC2 production in goblet cells (21) and acetate, propionate, and butyrate induce epithelial turnover (22). Furthermore, butyrate stabilizes tight junctions by inducing hypoxia-inducible factor 1α (HIF-1α) (23). *Lactobacillus*-derived lactate promotes epithelial cell hyper-proliferation during re-feeding after fasting (20). Lactate stimulates Gpr81 on Paneth and stromal cells to induce epithelial stem cell proliferation and differentiation in a Wnt3/β-catenin dependent manner (24). Feeding a low-fiber diet reduces mucus thickness, thereby increasing susceptibility to *Citrobacter rodentium* infection (25). In addition, a high-fat diet causes dysbiosis characterized by the underrepresentation of *Bifidobacterium*, *Lactobacillus*, *Clostridium*, and *Bacteroides* and the overrepresentation of *Oscillibacter* and *Desulfovibrio* (26). *Bifidobacterium* and *Lactobacillus* stabilize tight junctions (27, 28), whereas *Oscillibacter* and *Desulfovibrio* increase the gut permeability (29). Therefore, high-fat diets disrupt tight junctions (30), reduce mucus thickness (31), and enhance the translocation of bacterial endotoxins into the lamina propria (14, 26, 32). Similarly, a Western-style diet rich in fat (33), increases the risk of inflammatory bowel disease (34).

There are two main types of experimental diets for laboratory animals: crude diets (CDs) and purified diets (PDs), which are represented by the AIN-93G-formula diet (35). CDs are grain- or cereal-based diets composed of natural ingredients, such as soybean, corn, and fish meal. However, the exact composition varies among CDs and may differ between the batches of each CD because manufacturers alter the ingredients of the CD depending on the harvest supply (36). As the precise formula for a CD is not disclosed to the public, the addition or removal of specific nutrients is technically impossible. However, the formula for a PD is well-known; thus, PDs have been widely used in nutritional and medical studies (36). CDs usually contain abundant soluble and insoluble dietary fibers (approximately 20% of the total weight), whereas the total dietary fiber content of PDs is approximately 5%, most of which is insoluble fiber cellulose (36). Several studies have shown that feeding a PD alters the host metabolic and immune functions. For instance, a PD elevates plasma insulin (37) and transaminase levels and increases hepatic *de novo* lipogenesis (38). A PD also exacerbates dextran sulfate sodium-induced colitis in chronically retrained mouse models by impairing epithelial stem cell renewal (39). Meanwhile, a PD ameliorates *Clostridioides difficile* infection (40). These early studies illustrated the significant impact of dietary ingredients on host physiology. However, the influence of a PD on intestinal homeostasis, including the maintenance of mucosal barrier integrity mediated by the gut microbiota, remains to be elucidated.

This study demonstrates that epithelial turnover, fucosylation, and antimicrobial product generation are suppressed in PD-fed mice. Moreover, the PD affects the gut microbiota composition, characterized by a decrease in segmented filamentous bacteria (SFB), leading to attenuated epithelial barrier functions. Further, this study demonstrates that feeding a PD significantly affects epithelial turnover and barrier establishment by affecting intestinal microbiota.

## Materials

### Animals

Three-week-old BALB/cAJcl and BALB/cCrSlc mice were purchased from CLEA Japan (Tokyo, Japan) and Sankyo Labo Service Corporation (Tokyo, Japan), respectively. The Lgr5-EGFP-ires-creERT2 mice were purchased from Jackson Laboratory (Bar Harbor, ME, USA). The mice were fed a CD (CE-2) (CLEA, Tokyo, Japan) or a PD (AIN-93G) (Oriental Yeast, Tokyo, Japan) for 3 weeks. The nutritional formulas for each diet are shown in Supplementary Table S1. All animal experiments were performed according to the protocols approved by the Animal Studies Committee of Keio University and the National Center for Global Health and Medicine.

### Histology and immunofluorescence

We collected 10 cm each of the small-intestinal tissue from the pyloric region or ileocecal valve, representing the duodenum and ileum, respectively. Swiss roll-like sections of the duodenal and ileal tissue were subjected to histological examination and immunofluorescence. The intestinal tissue specimens were fixed in Mildform (Fujifilm Wako) overnight at 4°C, embedded in paraffin, and cut into 5 µm sections. For histological analysis, paraffin sections were deparaffinized in xylene, rehydrated in a series of ethanol concentrations (Nacalai Tesque), and stained with hematoxylin (Agilent Technologies, Inc., Santa Clara, CA, USA) and eosin (Wako Pure Chemical Industries, Osaka, Japan). The slides were mounted using Mount-Quick (DM-01; Cosmo Bio).

Deparaffinized sections were incubated in 10 mM sodium citrate buffer (pH 6.0) for antigen retrieval from the immunofluorescent paraffin sections. The sections were permeabilized with methanol (Nacalai Tesque), blocked with 3% BSA and an Avidin/Biotin Blocking kit (SP-2001, Vector), and incubated overnight with anti-Ki67 (GTX16667, GeneTex, 1:200), anti-Lysozyme C (sc-27958, SANTA CRUZ, 1:200), anti-TFF3 (RQ4090, NSJ Bioreagents, 1:200), anti-Muc2 (sc-15334, SANTA CRUZ, 1:200), or biotinylated UEA-1 (B-1065-2, Vector, 1:200). After washing with PBS, sections were incubated with Alexa Fluor 488-conjugated anti-rabbit IgG (A-11070, Invitrogen, 1:200), anti-goat IgG (A32814, Invitrogen, 1:200), Alexa Fluor 555-conjugated anti-rabbit IgG (A32794, Invitrogen, 1:200), or Alexa Fluor 647 streptavidin (405237, BioLegend, 1:200), and Hoechest33342 (H3570, Life Technologies, 1:500). The sections were subsequently mounted in ProLong™ Gold Antifade Mountant (P36930, Invitrogen). The slides were observed under a confocal laser microscope FV3000 (Olympus) and processed using Fiji (ImageJ).

EdU (5-ethynyl-2ʹ-deoxyuridine) labeling experiments were conducted as described previously (41, 42). Briefly, EdU (5 mg/kg) was administered intraperitoneally 24 h before euthanasia. EdU was detected using the Click-iT Plus EdU Cell Proliferation Kit for Imaging and Alexa Fluor 594 dye (Life Technologies), according to the manufacturer’s protocol.

### Intestinal permeability

A paracellular transport assay was performed as described previously with some modifications (43). Briefly, CD- or PD-fed 6-week-old mice were fasted for 4 h and subsequently received 60 mg/100 g body weight of 4 kDa FITC-dextran by intragastric administration. 45 min after intragastric administration, blood was collected by cardiac puncture and mixed with heparin (Mochida Pharmaceutical Co., Ltd.). Blood was centrifuged at 4°C, 1000 × *g* for 10 min to collect plasma. Plasma was diluted 1:5 in PBS, and the concentration of FITC-dextran was measured by using a fluorescence spectrophotometer (Infinite 2000; Tecan, Männedorf, Switzerland) at an excitation wavelength of 485 nm and an emission wavelength of 535 nm. A standard curve was prepared using values of 4-kDa FITC-dextran step-diluted in PBS.

### Flow cytometry of intestinal epithelial cells

Intestinal tissues were incubated for 30 min in Hanks' balanced salt solution (HBSS) containing 2% fetal bovine serum, 1 mM EDTA, and 1 mM dithiothreitol with gentle shaking. The epithelial cells were dissociated from the lamina propria by scratching, followed by incubation in collagenase RPMI1640 medium (Nacalai Tesque) solution containing 0.125 mg/mL collagenase (Wako Pure Chemical Industries), 0.5 mg/mL DNase I (Roche Diagnostics) at 37°C for 5 min. The isolated cells were incubated with anti-CD16/32 (FcγR) antibodies to block non-specific reactions and stained with specific antibodies. The cells were subsequently analyzed using a FACS LSRFortessa (BD Biosciences). The following antibodies, lectin, and streptavidin were used for flow cytometry: anti-CD16/32 (93), BV510 anti-mouse CD45 (30-F11), PE anti-CD24 (30-F1), FITC anti-mouse CD326 (EpCAM) (G8.8), APC anti-CD44 (IM7), and PE-Cy7 streptavidin were purchased from BioLegend; biotinylated-UEA1 was purchased from Vector Laboratories. Dead cells were detected using 7-AAD Viability Staining Solution (BioLegend).

### Bacterial DNA extraction and 16S rRNA gene sequencing

Bacterial genomic DNA was extracted using a QIAamp PowerFecal Pro DNA Kit (Qiagen) according to the manufacturer’s protocol. Before extracting genomic DNA, the ileal contents and tissues were homogenized for 10 min using a Shake Master Neo (Biomedical Sciences, Tokyo, Japan). A 16S ribosomal RNA (rRNA) genomic library was constructed following the protocol of the Illumina technical note with some modifications as described previously (44). Libraries were purified using AMPure XP beads (Beckman Coulter), diluted to 10 nM in Tris-HCl buffer, and pooled. The libraries were sequenced on a MiSeq (Illumina) with 300 bp paired-end reads.

### Microbiome analysis

Microbiome analysis was performed as described previously (44). Briefly, after removing host genomic DNA contaminants using Bowtie2, the FASTQ files were analyzed using QIIME2 (QIIME2 version 2020.8). The sequence data were demultiplexed and summarized using the QIIME2 paired-end demux. The sequences were subsequently trimmed and denoised using the dada2 plugin of QIIME2. Taxonomic assignment was performed with a naïve Bayes classifier trained on the SILVA_132 reference database (SSURef_NR99_132_SILVA) using the feature classifier plugin for QIIME2 (45–47). The phylogenetic tree for diversity analysis was reconstructed using the QIIME2 align-to-tree-mafft-fast tree. Diversity analysis was performed using QIIME2 core-metrics-phylogenetic analysis. The relative abundance of each taxon was calculated using the taxa collapse QIIME2 plugin.

### qPCR analysis to determine bacterial load

Real-time quantitative PCR (qPCR) was performed to estimate the bacterial load, following a previously published protocol (44). In brief, 2 µL of extracted DNA template, 10 µL of SsoAdvanced Universal SYBR Green Supermix (Bio-Rad Laboratories), 0.4 µL of forward primer and reverse primer for the 16S rRNA V3–V4 gene region, and 7.2 µL of nuclease-free water were mixed. A calibration curve was prepared using a dilution series of *Escherichia coli* genomic DNA (10^1^–10^8^ CFU). qPCR was conducted using a CFX96 Real-Time System (Bio-Rad Laboratories). The sequences of the primer sets are available upon request.

### Scanning electron microscopy

For scanning electron microscopy observation, gut samples were primary fixed with 2.5% glutaraldehyde for 12–24 h at 4°C, washed in 0.1M PBS, and secondary fixed with 1.0% osmium tetroxide for 2 h at 4°C. Samples were dehydrated using a series of increasing ethanol concentrations. After drying with a critical point dryer (CPD300, Leica Biosystems), the samples were placed on an aluminum scanning stage and then coated twice with Pt-Pd using a conductive quick coater at a current of 5 mA for 60 s two times (sc-701, SANYU ELECTRON). The Pt-Pd coated samples were imaged using SEM (SU6600, Hitachi High Tech) at 5 kV.

### RNA extraction and RNA-sequencing

Epithelial monolayers were isolated as described previously (42). Briefly, intestinal tissues were soaked in ice-cold HBSS (Fujifilm Wako Pure Chemical Corporation) containing 30 mM EDTA. After incubation on ice for 10 min, the epithelial monolayers were carefully separated from the lamina propria by manipulation using a fine needle under stereomicroscopic monitoring. Total RNA from the isolated epithelium was extracted using the NucleoSpin RNA Plus kit (740984.250, MACHEREY-NAGEL) and quantified using the Qubit RNA BR Assay Kit (Q10210, Invitrogen). A cDNA library was synthesized using Collibri 3ʹ mRNA Library Prep Kit for Illumina Systems (A38110024, Invitrogen) per the manufacturer’s protocol. The libraries were purified and enriched using AMPure XP beads (Beckman Coulter) and sequenced on a NovaSeq 6000 (Illumina) with 300 bp paired-end reads.

FASTQ files were mapped with STAR (version 5.0.1) (48) to the mouse mm10 reference genome with default parameters. Differential gene expression analyses were performed in R using RStudio (RStudio Inc.). Raw nonnormalized counts were imported into R and subsequently analyzed using the DESeq2 package. Genes with a total of fewer than 10 counts across all samples were removed, and normalization was calculated with default parameters for estimating size factors and dispersions. Genes with a *q* value < 0.05 and Log_2_ Fold Change > 1 were defined as “differentially expressed genes” and taken forward for further analysis (49). Volcano plots were generated with the R command EnhancedVolcano (https://github.com/kevinblighe/EnhancedVolcano). Gene Ontology (GO) enrichment and Kyoto Encyclopedia of Genes and Genomes (KEGG) pathway analyses were performed using the Database for Annotation, Visualization, and Integrated Discovery (DAVID; https://david.ncifcrf.gov/) (50). GO categories in the biological process ontology and KEGG pathways were identified with the significance criterion being *q*-value < 0.05, and |Log_2_(Fold Change)| > 1.

### Gas chromatography–mass spectrometry

Organic acid concentrations in the small intestinal and cecal contents were analyzed using gas chromatography–mass spectrometer (GC–MS) (51, 52). Briefly, small-intestinal or cecal contents were homogenized using 3 mm zirconia/silica beads (Biomedical Science) in ultrapure water for 10 min with Shake Master Neo (Biomedical Sciences, Tokyo, Japan). The homogenates were centrifuged at 4°C and 10 000 × *g* for 5 min and then the supernatants were collected. Next, 20% 5-sulfosalicylic acid and 1 mM ethylbutyric acid were added to the supernatants. The samples were incubated at room temperature for 10 min and centrifuged at 20°C and 15 000 × *g* for 15 min. HCl (37%) was added to the samples, and then diethyl ether was added and vortexed at room temperature (approximately 25°C) for 15 min. After centrifuging at 20°C and 15 000 × *g* for 5 min, the supernatants were collected, and 1g/mL *N*-methyl-*N*-trifluoroacetamide was added. The samples were incubated at room temperature and analyzed using a GC–MS system (JMS-Q1500GC, Japan Electron Optics Laboratory).

### Flow cytometry of lymphocytes

Small-intestinal lamina propria lymphocytes were isolated as described previously (51, 53), with some modifications. Briefly, intestinal tissues were incubated in HBSS containing 1 mM dithiothreitol and 20 mM EDTA at 37°C for 30 min to remove epithelial cells. The tissues were minced and subsequently incubated in an enzyme solution containing 0.2 unit/mL Liberase (Roche), 0.5 mg/mL DNase I (Roche Diagnostics), 2% FBS, 100 U/mL penicillin, 100 μg/mL streptomycin, and 12.5 mM HEPES, in RPMI1640 medium (Nacalai Tesque) at 37°C for 30 min. Cell suspensions were washed with 2% FBS in PBS and subjected to Percoll (Cytiva) gradient separation.

Analysis of *ex vivo* cytokine production in ILC3s was performed as described previously (54), with some modifications. Isolated lamina propria lymphocytes were incubated for 1.5 h at 37°C in RPMI1640 containing 10% FBS, and subsequently for 1.5 h at 37°C in RPMI1640 containing 10% FBS and eBioscience Protein Transport Inhibitor Cocktail (Thermo Fisher Scientific).

The lymphocytes were incubated with anti-CD16/32 (FcγR) antibodies to block non-specific reactions and stained with specific antibodies. The cells were fixed for intracellular transcription factor staining using the eBioscience Foxp3/Transcription Factor Staining Buffer Set (Thermo Fisher Scientific) for 45 min. After washing, the transcription factors and IL-22 were stained with specific antibodies. The cells were subsequently analyzed using a FACS Celesta (BD Biosciences). The antibodies used for flow cytometry were as follows: anti-CD16/32 (93), BV510 anti-mouse CD45 (30-F11), BV421 anti-mouse CD3e (145-2C11), APC anti-F4/80 (BM8), and Alexa Fluor 647 anti-CD8a (53-6.7) (all from BioLegend); BV786 anti-CD4 (GK1.5) and PE-CF594 anti-RORγt (Q31-378) (both from BD Biosciences); and FITC anti-Foxp3 (FJK-16s), PE anti-IL-22 (1H8PWSR), APC anti-CD11c (N418), and APC anti-CD45R (B220) (RA3-6B2) (all from Thermo Fisher Scientific). Dead cells were stained using the Fixable Viability Stain 780 (BD Biosciences).

### Reverse transcription and qPCR analysis

Epithelial monolayers were isolated as described above and homogenized in 1 mL Sepasol (Nacalai Tesque). After incubating the samples at room temperature for 5 min, 0.2 mL of chloroform (Nacalai Tesque) was added and the mixture was vortexed for 15 s. The samples were incubated at room temperature for 3 min and then centrifuged at 4°C and 12 000 × *g* for 15 min. The aqueous layer was collected, and 500 µL of 2-propanol was added to the samples and shaken. The samples were incubated at room temperature for 10 min and centrifuged at 4°C and 12 000 × *g* for 10 min. The supernatants were removed and the pellets were dissolved in 75% ethanol. After centrifuging at 4°C and 7500 × *g* for 5 min, the supernatants were removed and air dried for 5 min. The samples were dissolved in nuclease-free water and incubated at 55°C for 10 min. The RNA concentration was measured using a NanoDrop 2000 spectrophotometer (Thermo Fisher Scientific). First-strand cDNA synthesis was performed using the ReverTra Ace qPCR RT Master Mix with gDNA Remover (TOYOBO) according to the manufacturer’s protocol. qPCR was conducted on a CFX96 Real-Time System (Bio-Rad Laboratories) using SsoAdvanced Universal SYBR Green Supermix (Bio-Rad Laboratories). The sequences of the primer sets are available upon request.

### qPCR analysis to determine bacterial load

Real-time qPCR was performed to estimate the bacterial load, following a previously published protocol (44). In brief, 2 µL of extracted DNA template, 10 µL of SsoAdvanced Universal SYBR Green Supermix (Bio-Rad Laboratories), 0.4 µL of forward primer and reverse primer for the 16S rRNA V3-V4 gene region, and 7.2 µL of nuclease-free water were mixed. A calibration curve was prepared using a dilution series of *E. coli* genomic DNA (10^1^–10^8^ CFU). qPCR was conducted using a CFX96 Real-Time System (Bio-Rad Laboratories). Primer sequences are available upon request.

### Immunoblotting

Epithelial monolayers were isolated as described above and homogenized in 1 mL RIPA buffer containing 0.1% SDS and 10 mM NaF. After incubating on ice for 30 min, the samples were centrifuged at 4°C and 10 000 × *g* for 10 min, and the supernatants were collected. The protein concentration was measured using the Pierce Rapid Gold BCA Protein Assay Kit (Thermo Fisher Scientific). Sample Buffer Solution containing 2-ME (Nacalai Tesque) was added to the supernatant and denatured at 95°C for 5 min. Samples were subsequently subjected to SDS-PAGE and transferred onto polyvinylidene fluoride membranes (Immobilon-P; Merck). Membranes were blocked with PVDF Blocking Reagent for Can Get Signal (TOYOBO) and incubated at room temperature for 1 h with the following primary antibodies: anti-REG3G (ab198216, Abcam, 1:2000), anti-HMGCS2 (AV41563, Sigma-Aldrich, 1:1000), anti-PDK4 (ab214938, Abcam, 1:1000), anti-PPARα (sc-398394, SANTA CRUZ, 1:1000), anti-FABP1 (13368, Cell Signaling, 1:1000), and anti-β-actin (010-27841, Fujifilm Wako Pure Chemical Corporation, 1:2000). After washing with TBS-T, membranes were incubated at room temperature for 1 h with the following HRP-conjugated secondary antibodies: anti-rabbit IgG, HRP-linked antibody (7074S, Cell Signaling, 1:2000) and anti-mouse IgG, HRP-linked antibody (7076S, Cell Signaling, 1:2000). Signals were detected using Chemi-Lumi One L (Nacalai Tesque) or Chemi-Lumi One Super (Nacalai Tesque) and visualized using ImageQuant LAS 4000 (GE Healthcare, Little Chalfont, UK). The membranes were visualized using an Amersham ImageQuant 800 (Cytiva, Tokyo, Japan), and band densities were quantified using Fiji (ImageJ). For the detection of STAT3 and pSTAT3, the membrane used in immunoblotting for HMGC2, PDK4, and PPARα was incubated with WB stripping solution (Nacalai Tesque) for 15 min to remove the bound antibodies, and was subjected to immunoblotting using anti-pSTAT3 (9145, Cell Signaling, 1:2000) and anti-STAT3 (9139, Cell Signaling, 1:2000) antibodies.

### Statistical analysis

GraphPad version 9 was used for statistical analyses, unless otherwise specified. Values are expressed as the mean ± SD. Differences between the mean values were analyzed using unpaired *t*-tests, or one-way ANOVA or two-way ANOVA followed by Tukey’s multiple comparison test or Šídák’s multiple comparison test. Differences were considered statistically significant when *P* values were less than 0.05. Statistical significance is shown as *P* < .05*, *P* < .01**, *P* < .001***, and *P* < .0001****. A comparison of the bacterial taxa at the genus level was performed using the LEfSe method (55) on the website (https://huttenhower.sph.harvard.edu/galaxy/root). Differences in β-diversities between the two groups were analyzed using PERMANOVA.

## Results

### The PD attenuates intestinal epithelial turnover

**Figure 1.**
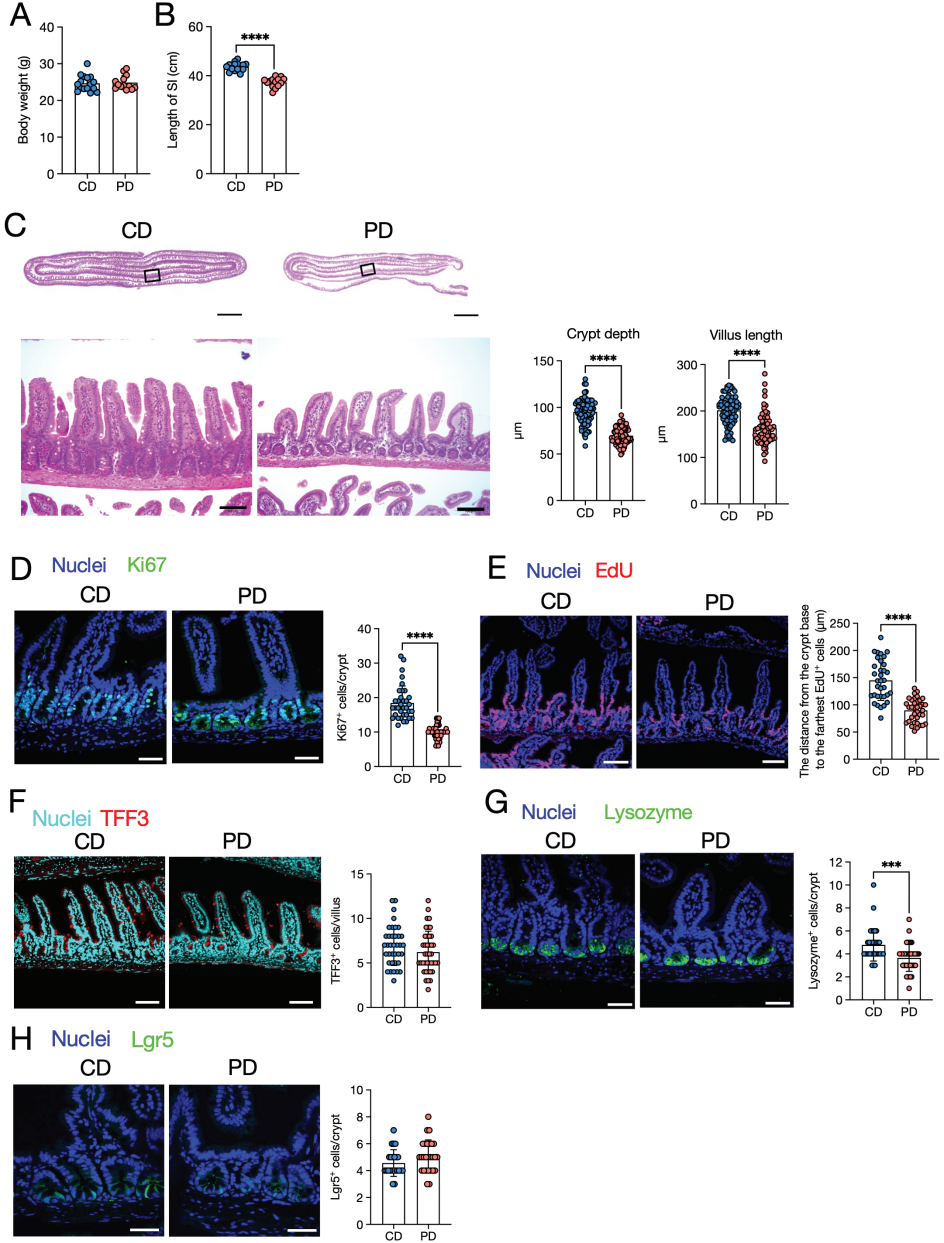
A purified diet (PD) attenuates epithelial proliferation and turnover. Three-week-old mice were fed a crude diet (CD) or a PD for 3 weeks, and the small intestine was analyzed. (A) Body weights. *n* ≥ 13 mice per group. (B) Length of the small intestine. *n* ≥ 14 mice per group. (C) Representative images of hematoxylin and eosin-staining of the ileum from PD- and CD-fed mice are shown. Images of the Swiss roll-like sections (top) and ileal epithelium (bottom). Scale bars: 2000 µm (top) or 100 µm (bottom). Scale bars: 100 µm. *n* ≥ 80 crypt regions from four individual mice per group were analyzed for crypt depth. *n* ≥ 60 villi regions from four individual mice per group for villus length. (D) Immunofluorescence images of Ki67 in the ileal crypts. Ki67^+^ cells only in the epithelial monolayer in the crypt region were counted. The green staining merged with nuclei was recognized as the specific signal. Scale bars: 50 µm. *n* ≥ 35 crypt regions from four individual mice per group were analyzed. (E) Representative images of EdU fluorescence and distance from the crypt base to the farthest EdU-labeled cells. Scale bars: 100 µm. *n* ≥ 30 crypt-villus regions from three individual mice per group were analyzed. (F) Representative immunofluorescence images of TFF3 and number of TFF3^+^ cells per ileal villus. Scale bars: 100 µm. *n* ≥ 35 villi regions from four individual mice per group were analyzed. (G) Immunofluorescence images of lysozyme and the number of lysozyme^+^ cells per crypt. Scale bars: 50 µm. *n* ≥ 30 crypt regions from four individual mice per group were analyzed. (H) Fluorescent images of Lgr5 and number of Lgr5^+^ cells per ileal crypt. Scale bars: 100 µm. *n* ≥ 35 crypt regions from four individual mice per group were analyzed. The data represent the mean ± SD. *P < .05, **P < .01, ***P < .001, ****P < .0001. *P* values were determined by unpaired *t*-test. CD, crude diet; PD, purified diet.

First, we examined the effects of PD feeding on intestinal morphology. Despite no significant changes in body weight, PD-fed mice exhibited a significant reduction in gut length compared to CD-fed mice (Fig. 1A and B). Additionally, we observed that the ileum of PD-fed mice displayed shorter crypt depth and villus length than those of CD-fed mice (Fig. 1C). Immunofluorescent staining for Ki67, a marker of cell proliferation, also showed that PD-fed mice had significantly fewer Ki67^+^ cells than CD-fed mice (Fig. 1D). We further performed a label tracing experiment using EdU. At 24 h after the EdU pulse, the distance from the crypt base to the farthest EdU-labeled cell was significantly shorter in the PD-fed mice than in the CD-fed mice (Fig. 1E). Notably, the crypt depth was also shortened in the duodenum; however, the villus length and number of Ki67^+^ cells were comparable between the two groups (Supplementary Fig. 1A and B). The distance from the crypt base to the farthest EdU-labeled cells considerably decreased in the duodenum of PD-fed mice; however, the difference was marginal compared to that in the ileum (Supplementary Fig. 1C). These results suggest that PD feeding leads to a relatively attenuated epithelial turnover, particularly in the ileum. On the contrary, the paracellular translocation assay exhibited no significant changes in the physical barrier function between the two groups (Supplementary Fig. 1D).

We also observed the influence of the PD on epithelial cell composition. The PD did not significantly alter the number of TFF3^+^ goblet cells (Fig. 1F). Meanwhile, the number of lysozyme^+^ Paneth cells was slightly, but significantly, less in PD-fed mice than in CD-fed mice (Fig. 1G). Furthermore, the number of Lgr5^+^ intestinal stem cells (ISCs) was comparable between the two groups (Fig. 1H). Thus, PD feeding has a minimal, if any, impact on epithelial cell composition.

### The PD suppresses antimicrobial product expression

**Figure 2.**
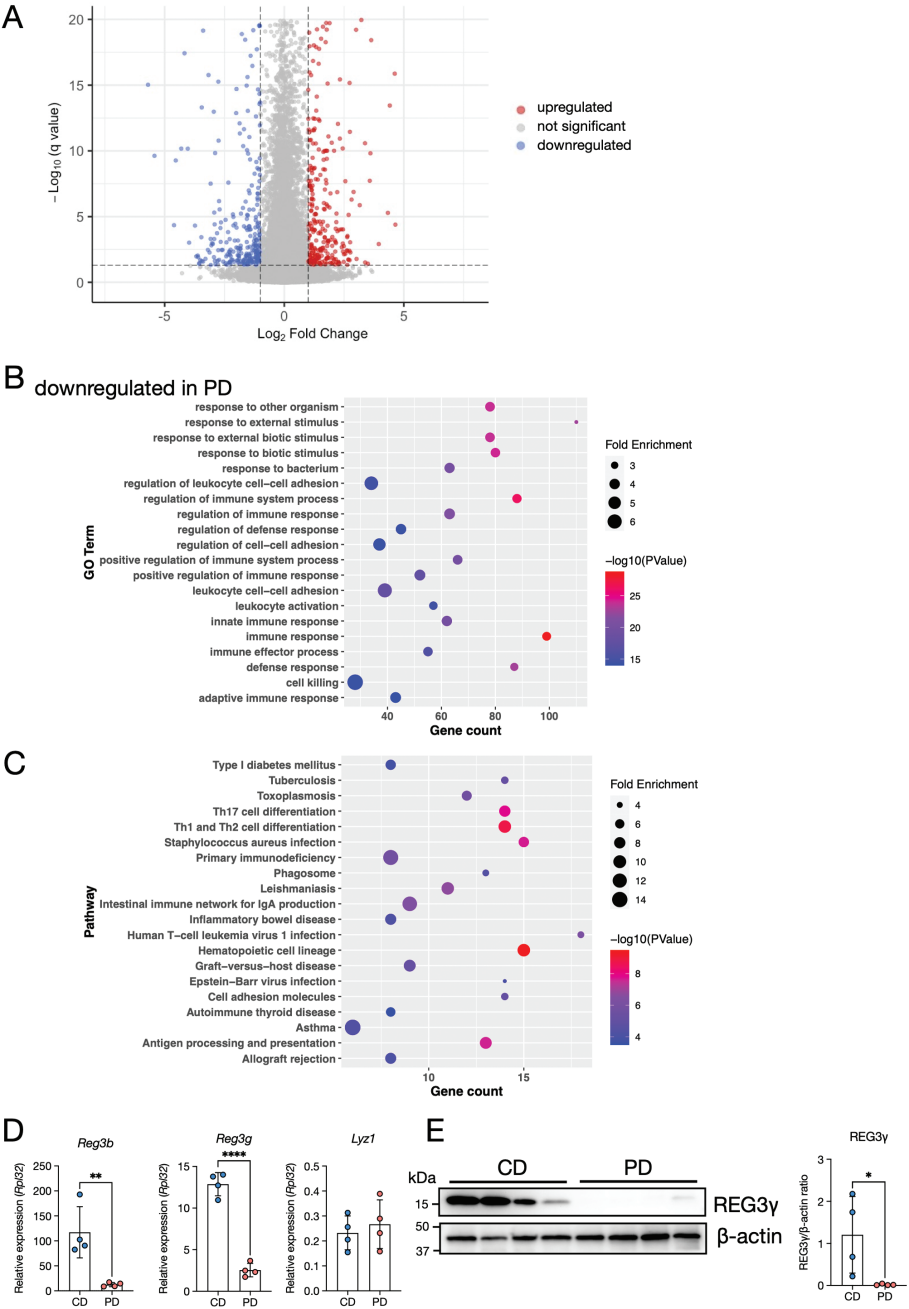
Impact of the PD on the gene expression pattern in the ileal epithelium. (A) Volcano plots comparing the ileal epithelium of CD- vs. PD-fed mice on the basis of RNA-sequencing data. *n* = 6. Genes up- or downregulated (Log_2_(fold change) > 1 or  *q* < 0.05) are highlighted. (B, C) Gene ontology (GO) enrichment (B) and Kyoto Encyclopedia of Genes and Genomes (KEGG) pathway analyses (C) of downregulated genes in PD-fed mice. The 20 most significant GO terms are represented in the accompanying bubble plot. Bubble colors represent −log_10_ (*P* values). Bubble sizes indicate fold enrichment. (D) Relative mRNA expression of *Reg3b*, *Reg3g*, *and Lyz1* in the ileal epithelium of CD- and PD-fed mice. *n* = 4 mice per group. (E) Representative immunoblot of the ileal epithelium from CD- and PD-fed mice for detecting REG3γ and β-actin (loading control). Band intensities were measured using densitometry. *n* = 4 mice per group. The data represent the mean ± SD. *P < .05, **P < .01, ***P < .001, ****P < .0001. *P* values were determined by unpaired *t*-test. CD, crude diet; PD, purified diet.

To better understand the effect of the PD on epithelial integrity, we comprehensively analyzed the gene expression profiles of the ileal and duodenal epithelia using RNA-sequencing. PD feeding significantly downregulated 2750 genes and upregulated 3013 genes compared with CD feeding (Fig. 2A, Supplementary Table S2A and B). GO enrichment analysis showed that gene clusters related to immune responses (such as response to other organism, response to bacterium, and defense response) were significantly decreased in PD (Fig. 2B, Supplementary Table S3A). KEGG pathway analysis also showed the downregulation of immune-related gene clusters (Fig. 2C, Supplementary Table S3B). These gene clusters included genes encoding antimicrobial products such as *Reg3b* and *Reg3g* (Supplementary Table S3A). qPCR analysis confirmed downregulation of the antimicrobial products *Reg3b* and *Reg3g* in the ileal epithelium of PD-fed mice (Fig. 2D). We also confirmed that the protein level of REG3γ was significantly lower in PD-fed mice (Fig. 2E). In contrast, *Lyz1* expression was comparable between the two groups, suggesting that PD feeding may attenuate the activation but not the differentiation of Paneth cells.

In the duodenum, PD feeding resulted in the downregulation of 2741 genes and the upregulation of 3017 genes (Supplementary Fig. 2A, Table S2C and D). Among the GO enrichments of the downregulated genes were the regulation of immune system, lipid metabolic, organic acid metabolic, and carboxylic acid metabolic processes (Supplementary Fig. 2B, Table S4A). KEGG pathway analysis revealed the downregulation of metabolic pathways, including those of the cytochrome P450 family (Supplementary Fig. 2C, Table S4B). Similar to that in the ileum, *Reg3b* and *Reg3g* decreased in the duodenum of PD-fed mice, although the expression levels of these genes were lower in the duodenum than in the ileum (Supplementary Fig. 2D and Fig. 2D). Thus, the PD attenuated the expression of antimicrobial products, especially in the ileum.

### The PD induces fatty acid oxidation and ketogenesis in the ileum

**Figure 3.**
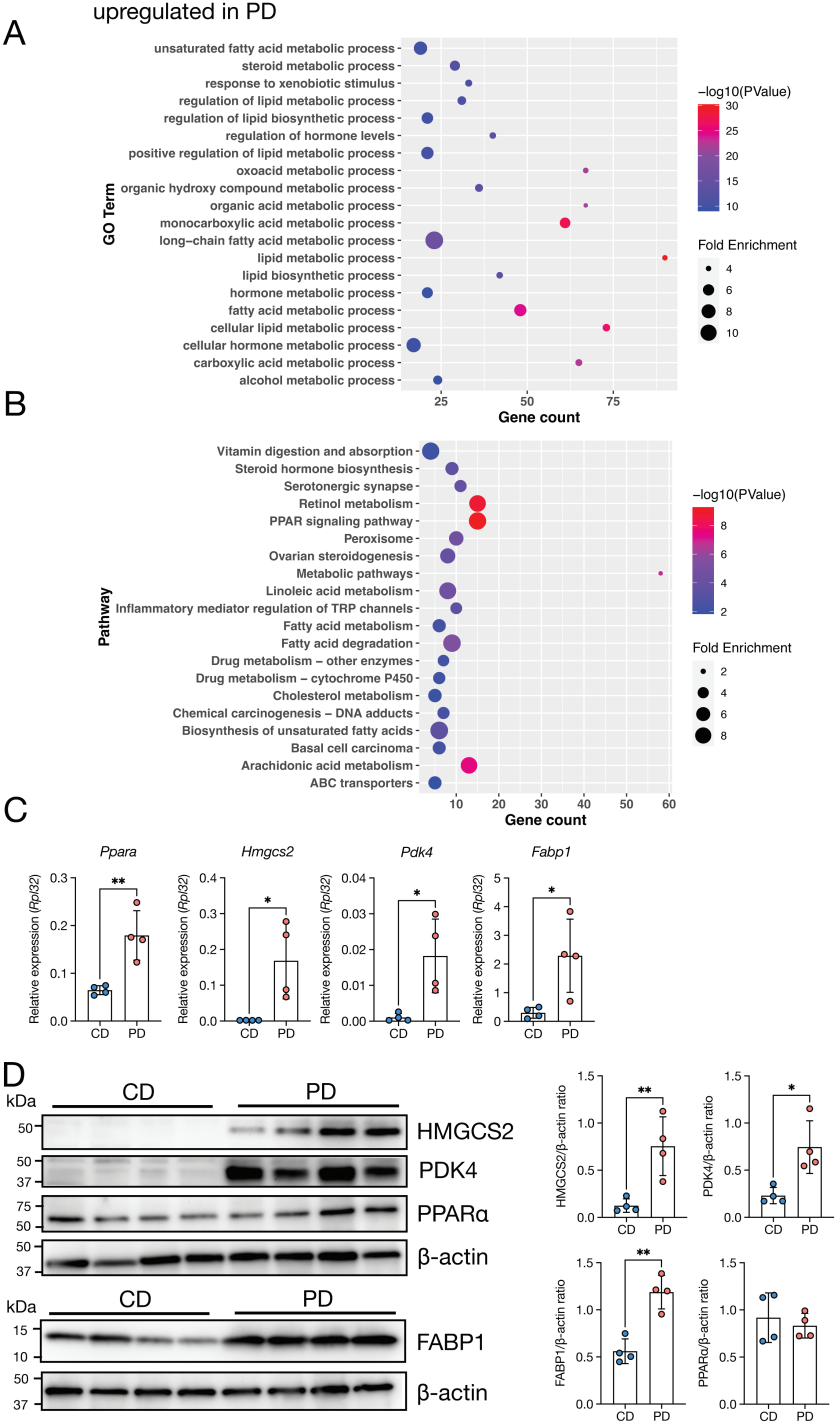
PD feeding induces metabolic rewiring to fatty acid oxidation and ketogenesis in the ileum. (A, B) GO enrichment (A) and KEGG pathway analyses (B) of upregulated genes in PD-fed mice. The 20 most significant GO terms are represented in the accompanying bubble plot. Bubble colors represent −log_10_ (*P* values). Bubble sizes indicate fold enrichment. (C) Relative mRNA expression of *Ppara*, *Hmgcs2*, *Pdk4*, and *Fabp1* in the ileal epithelium of CD- and PD-fed mice. *n* = 4 mice per group. (D) Representative immunoblots of the ileal epithelium of CD- and PD-fed mice, with detection of HMGCS2, PDK4, PPARα, FABP1, and β-actin (loading control). Band intensities were measured using densitometry. *n* = 4 mice per group. The data represent the mean ± SD. *P < .05, **P < .01. *P* values were determined by unpaired *t*-test. CD, crude diet; PD, purified diet.

RNA-sequencing data further showed that the PD profoundly increased the expression of genes involved in lipid and fatty acid metabolism in the ileum (Fig. 3A). KEGG pathway analysis revealed that gene clusters involved in metabolic pathways such as retinol metabolism, fatty acid degradation, and peroxisome proliferator-activated receptor (PPAR) signaling were upregulated by PD feeding (Fig. 3B). Notably, these gene clusters included *Ppara*, *Hmgcs2*, *Pdk4*, and *Fabp1*, encoding PPARα, 3-hydroxy-3-methylglutaryl-CoA synthase 2 (HMGCS2), pyruvate dehydrogenase kinase 4 (PDK4), and fatty acid binding protein 1 (FABP1), respectively (Supplementary Table S3C and D). qPCR analysis confirmed the upregulation of *Ppara*, *Hmgcs2*, *Pdk4*, and *Fabp1* in the PD-fed mice (Fig. 3C). Likewise, PD feeding increased protein expression of HMGCS2, PDK4, and FABP1, although PPARα levels were not significantly altered (Fig. 3D). Given that these PPARα target molecules promote ketogenesis and fatty acid oxidation under insufficient nutritional conditions (56, 57), the PD may cause metabolic rewiring to fatty acid oxidation and ketogenesis in the ileal epithelium.

In the duodenum of PD-fed mice, the upregulated gene clusters included regulation of signaling, regulation of molecular function, and positive regulation of responses to stimuli (Supplementary Fig. 3A, Table S4C). Meanwhile, carbohydrate metabolic pathways, including the carbohydrate digestion and absorption, fructose and mannose metabolism, and glycolysis/gluconeogenesis, were increased in the duodenum of PD-fed mice (Supplementary Fig. 3B, Table S4D). GO terms and pathways included *Slc2a5*, *Aldoart1*, *Gp6c*, *Aldob*, and *Fbp1*, which encode glucose transporter type 5 (GLUT5), aldolase 1 A, retrogene 1 (Aldoart1), glucose-6-phosphatase catalytic subunit 1 (GP6C), aldolase, fructose-bisphosphate B (Aldob), and fructose 1,6-bisphosphate (FBP), respectively (Supplementary Table S4C and D). These molecules are responsible for fructose transport from the intestinal lumen to the epithelium and its subsequent metabolism in the small intestine (41). These results imply that the PD may facilitate carbohydrate metabolism in the duodenum. Thus, the PD may induce different metabolic statuses in the duodenum and the ileum.

### The PD suppresses epithelial fucosylation in the ileum

**Figure 4.**
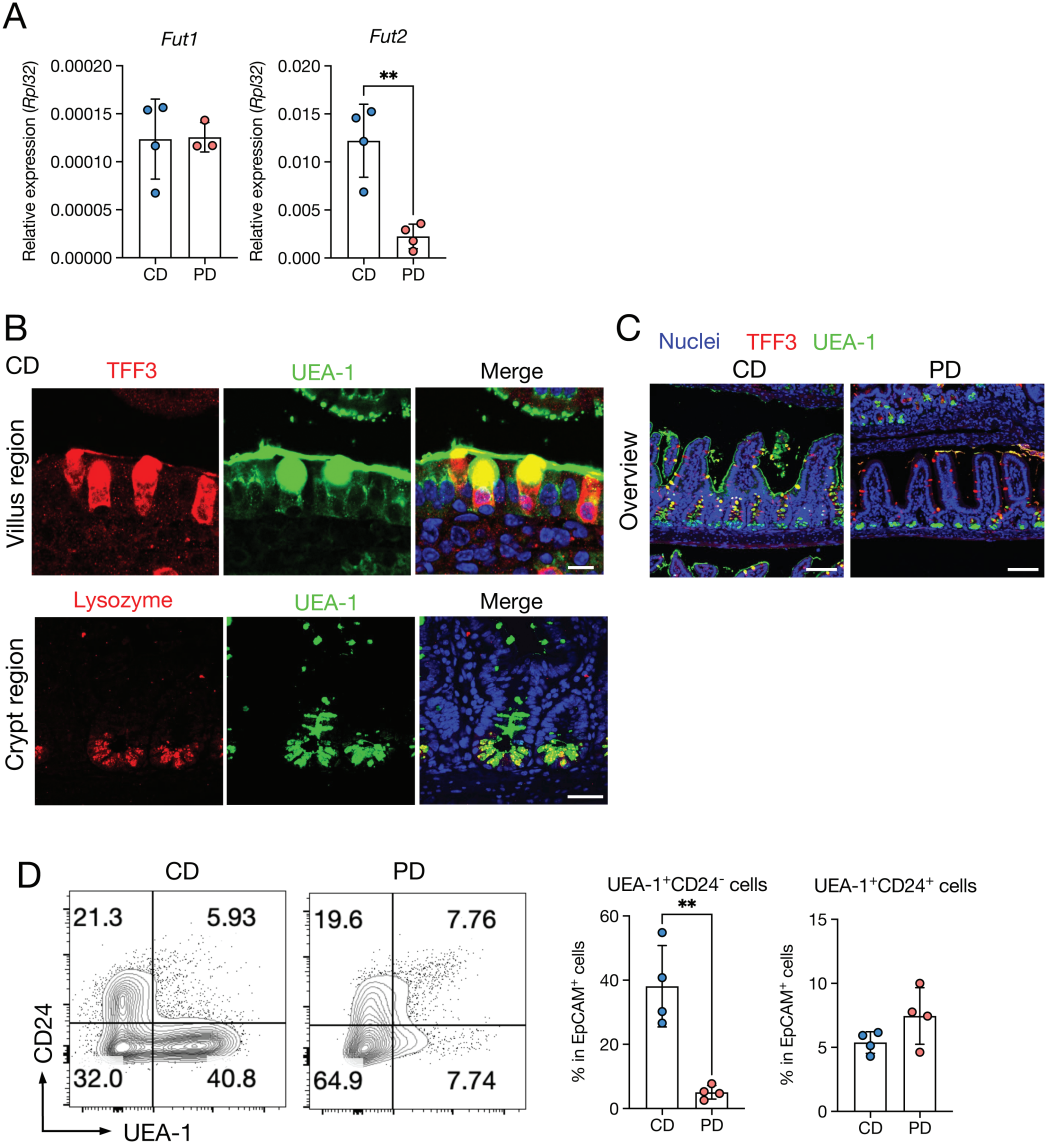
The PD suppresses epithelial fucosylation. (A) Relative mRNA expression of *Fut1* and *Fut2* in the ileal epithelium of CD- and PD-fed mice. *n* > 3 mice per group. (B) Top: Immunofluorescent images of TFF3 and UEA-1 in the villus region. Scale bars: 10 µm. Lower: Immunofluorescent images of lysozyme and UEA-1 in the crypt region. Scale bars: 40 µm. (C) Immunofluorescent images of TFF3 and UEA-1 in the ileum of CD- and PD-fed mice. Scale bars: 100 µm. (D) Representative plots of flow cytometry and percentage of UEA-1^+^CD24^−^ and UEA-1^+^CD24^+^ cell subsets in the ileum of CD- and PD-fed mice. *n* = 4 mice per group. The data represent the mean ± SD. **P< .01. *P* values were determined by unpaired *t*-test. CD, crude diet; PD, purified diet.

RNA-sequencing data demonstrated significant downregulation in the gene expression of *fucosyltransferase 2* (*Fut2*), encoding the enzyme responsible for α1,2-fucose transfer to the epithelial glycans (58, 59), in the ileum of PD-fed mice (Figs. 2A, 3A, Supplementary Table S2A). Additionally, *B3gnt7*, which mediates the fucosylation of mucin *O*-glycans in goblet cells (60), was downregulated in the PD-fed mice (Fig. 2A, Supplementary Table S2A). qPCR analysis confirmed that *Fut2* was significantly decreased in the PD group compared to the CD group, whereas *Fut1* expression was not significantly changed (Fig. 4A). We, therefore, investigated the impact of PD feeding on epithelial fucosylation using *Ulex europaeus* agglutinin-1 (UEA-1), a lectin that reacts explicitly with α(1,2)-fucose. In CD-fed mice, positive signals for UEA-1 were detected at the apical surface of the villus epithelium and in the intracellular granules of TFF3^+^ goblet cells and lysozyme^+^ Paneth cells (Fig. 4B and C), which is consistent with previous studies (59, 61). In contrast, UEA-1 staining in the villus epithelium and goblet cells was nearly absent in the PD-fed mice, although Paneth cells retained their reactivity to UEA-1. Flow cytometry also confirmed that the number of UEA-1^+^CD24^−^, but not UEA-1^+^CD24^+^, cells was significantly decreased in PD-fed mice (Fig. 4D). CD24 is a marker expressed on Paneth and enteroendocrine cells (62), suggesting that PD feeding decreases the fucosylation of enterocytes and goblet cells by downregulating Fut2 without affecting that of Paneth cells.

### The PD alters the gut microbiota

**Figure 5.**
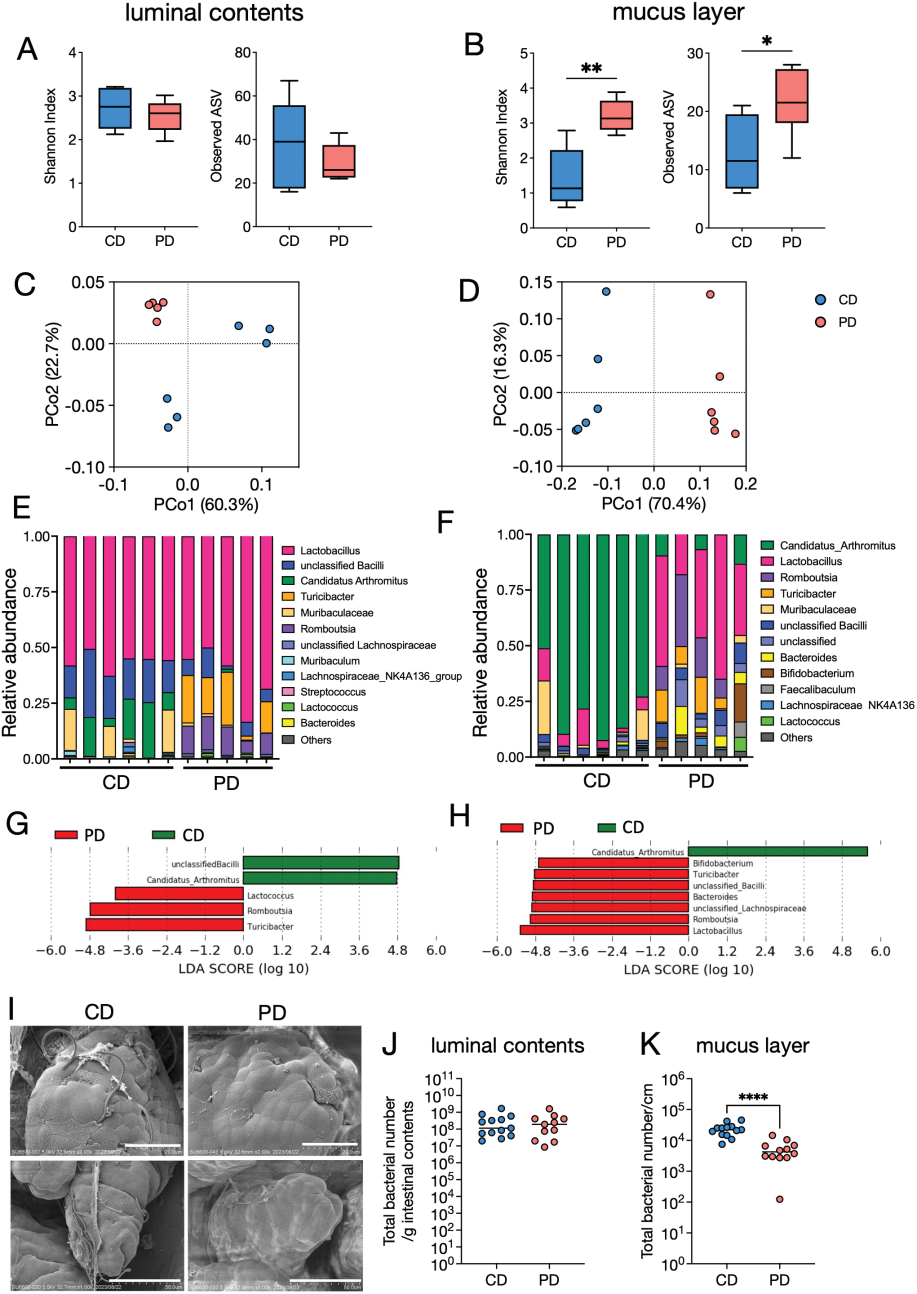
The PD alters the gut microbiota in the ileum. (A, B) α-diversities (Shannon index and observed ASVs) of the microbiota in the ileal contents (A) and ileal mucus layer (B) in CD- and PD-fed mice. (C, D) Principal coordinate analysis of weighted UniFrac distances between microbiota in the ileal contents (C) and ileal mucus layer (D). (E, F) Composition of the microbiota at the genus level in the ileal contents (E) and ileal mucus layers (F). (G, H) Discriminating taxa between CD- and PD-fed mice in the ileal contents (G) and ileal mucus layer (H) as determined using LEfSe analysis (55). (I) Scanning electron microscopy of the ileal villi from CD- or PD-fed mice. (J, K) The total bacterial load in the ileal contents (I) and ileal mucus layer (J) was analyzed using qPCR for the 16S rRNA V3–V4 region. The data represent the mean ± SD. *P < .05, **P < .01. *P* values were determined by unpaired *t*-test. CD, crude diet; PD, purified diet.

We subsequently examined the effects of the PD on the gut microbiota using 16S rRNA gene sequencing. As the gut microbiota composition differs between the luminal contents and the mucus layer (2), we analyzed the gut microbiota at both sites of the ileum. We found that α-diversity indices were significantly increased in the mucus layer of PD-fed mice, while there are no significant differences in the luminal contents (Fig. 5A and B). β-diversity determined using principal coordinate analysis (PCoA) based on weighted UniFrac distance was significantly different between the two groups both in the luminal contents [weighted UniFrac distance: *P* = .004 (PERMANOVA)] and mucus layer [weighted UniFrac distance: *P* = .003 (PERMANOVA)] (Fig. 5C and D).

Taxonomic assignment followed by linear discriminant analysis effect size (LEfSe) analysis (55) demonstrated that *Candidatus* Arthromitus, also called SFB, was abundant in the luminal contents of the CD-fed mice (Fig. 5E, G). Multiple genera, including *Lactococcus*, *Romboutsia*, and *Turicibacter*, were significantly increased in the intestinal contents of the PD group (Fig. 5E and G). SFB was dominant (accounting for 50%–80%) in the mucus layer microbiota of CD-fed mice. In contrast, SFB was almost absent in the PD-fed mice (Fig. 5F). Instead, the abundances of *Lactobacillus*, *Romboutsia*, *Turicibacter*, *Bacteroides*, and *Bifidobacterium* significantly increased in the PD group (Fig. 5F and H).

Scanning electron microscopy revealed that SFB was attached to the ileal epithelium of CD-fed mice. However, they were hardly observed in PD-fed mice (Fig. 5I). Furthermore, the total bacterial load significantly decreased in the mucus layer, despite no significant difference in the intestinal contents (Fig. 5J and K). Together, these findings indicate that PD feeding significantly affects microbial communities, particularly in the mucus layer.

### The PD does not affect SCFA levels in the ileal contents

Commensal bacteria-derived SCFAs and lactate promote epithelial turnover (22, 24). Therefore, we examined the effects of PD feeding on luminal organic acid concentrations. Acetate and butyrate were significantly decreased in the cecal contents of PD-fed mice compared with CD-fed mice, whereas propionate and lactate levels were not significantly different (Supplementary Fig. 4A–D). The levels of acetate, propionate, and butyrate in the ileal contents were marginal (approximately 1.2 µM, 0.25 µM, and 0.25 mM, respectively), whereas lactate was abundant (approximately 5.6 mM) and comparable between PD and CD groups (Supplementary Fig. 4A–D). Thus, luminal metabolites are most likely dispensable for the attenuated epithelial turnover in the ileum of PD-fed mice.

### The PD suppresses epithelial proliferation, fucosylation, and REG3γ expression by reducing SFB

SFB promotes epithelial turnover (63) in the ileum and consolidates epithelial barrier functions by inducing fucosylation (12) on the epithelial surface and the production of REG3γ (64). Therefore, we investigated whether the underrepresentation of SFB by PD feeding contributes to the reduction of epithelial proliferation and function. We obtained mice from two breeding grounds: Fuji and Inasa. We analyzed Fuji-derived BALB/c mice (Fuji mice), which possess abundant SFB upon CD feeding (Fig. 5E–I). In contrast, BALB/c mice from the Inasa breeding ground (Inasa mice) lacked the SFB (Supplementary Fig. 5A). Correspondingly, the β-diversity in the intestinal contents and mucus layer was distinct between Fuji and Inasa groups, especially upon CD feeding (Supplementary Fig. 5B and C). Taxonomic assignment revealed that the PD decreased the abundance of bacilli but increased the levels of *Faecalibaculum* in both the luminal contents and mucus layer of PD-fed Inasa mice. (Supplementary Fig. 5D and E). Consistent with the Fuji mice, the total bacterial load in the mucus layer was significantly decreased by the PD in Inasa mice (Supplementary Fig. 5F and G).

**Figure 6.**
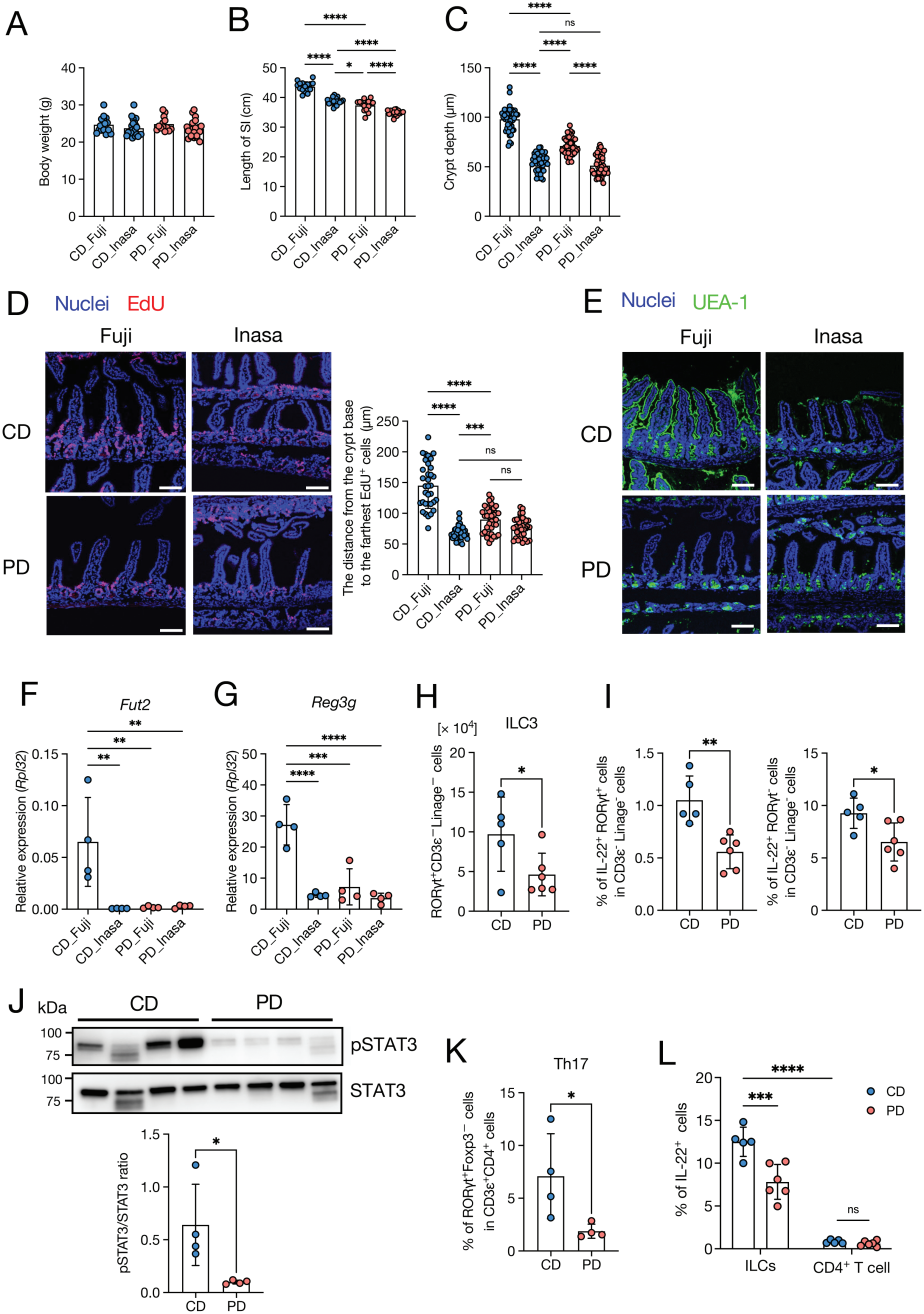
The PD affects intestinal turnover and barrier functions by reducing segmented filamentous bacteria. The effect of the PD on epithelial turnover and functions in the ileal epithelium was investigated in mice from two vendors: Fuji and Inasa. (A) Body weight, *n* ≥ 10 mice per group. (B) Small intestine length, *n* ≥ 10 mice per group. (C) Crypt depth, *n* ≥ 40 crypt regions from four individual mice per group were analyzed. (D) Representative images of EdU fluorescence and distance from the crypt base to the farthest EdU-labeled cells. Scale bars: 100 µm. *n* ≥ 30 crypt-villi regions from three individual mice per group were analyzed. (E) Immunofluorescent images of UEA-1 in the ileum. Scale bars: 100 µm. (F, G) Relative mRNA expression of *Fut2* (F) and *Reg3g* (G) in the ileal epithelium of CD- and PD-fed mice. *n* = 4 mice per group. (H) The number of ILC3 (RORγt^+^ CD3ε^−^linage^−^ cells). *n* ≥ 5 mice per group. (I) Percentage of IL-22^+^RORγt^+^ cells and IL-22^+^RORγt^−^ cell in CD3ε^−^linage^−^ cells. *n* ≥ 5 mice per group. (J) Representative immunoblot of the ileal epithelium of CD- and PD-fed Fuji mice, with detection of pSTAT3, STAT3, and β-actin (loading control). *n* = 4 mice per group. (K) Percentage of Th17 (RORγt^+^Foxp3^−^) cells in CD3ε^+^CD4^+^ cells. *n* ≥ 5 mice per group. (L) Percentage of IL-22^+^ in ILCs (CD3ε^−^linage^−^) cells and CD4^+^ T (CD3ε^+^CD4^+^ cells).The data represent the mean ± SD. *P < .05, **P < .01, ***P < .001, ****P < .0001. *P* values were determined by unpaired *t*-test (I–K), one-way ANOVA followed by Tukey’s multiple comparison test (A–D, F, G), or two-way ANOVA followed by Tukey’s multiple comparison test (H). CD, crude diet; ns, not significant; PD, purified diet.

Subsequently, we compared the influence of PD feeding on epithelial integrity in the absence (Inasa) and presence (Fuji) of SFB. Upon CD feeding, Inasa mice exhibited a shorter small intestine compared to Fuji mice. Additionally, we observed that the small-intestine length of PD-fed Inasa mice was even shorter than that of CD-fed Inasa mice, although their body weights were comparable (Fig. 6A and B). In contrast, the crypt depth and the distance from the crypt base to the farthest EdU^+^ cells were significantly decreased in PD-fed Fuji mice but not in Inasa mice (Fig. 6C and D). In addition, the crypt depth and the distance from the crypt base to the farthest EdU^+^ cells were shorter in CD-fed Inasa mice than in CD-fed Fuji mice (Fig. 6C and D), indicating that epithelial turnover was slower in Inasa mice. Lectin staining of UEA-1 showed that the villus epithelium of Fuji mice was UEA-1 positive in the CD-fed group, and PD feeding profoundly reduced the UEA-1 positive area, whereas it was UEA-1 negative in Inasa mice, even in the CD-fed group (Fig. 6E). In addition, Fut2 expression was downregulated by the PD in Fuji mice and was lower in CD-fed Inasa mice than in CD-fed Fuji mice (Fig. 6F). Furthermore, the PD suppressed *Reg3g* expression in Fuji mice, whereas no differences were observed in Inasa mice (Fig. 6G). These results correlated with the SFB abundance in the mucus layer (Supplementary Fig. 5A), suggesting that the PD suppressed epithelial turnover, fucosylation, and antimicrobial product generation by inhibiting SFB colonization. SFB induces REG3γ production by promoting IL-22 production from ILC3s and subsequent phosphorylation of STAT3 (pSTAT3) in the intestinal epithelium (65). SFB also enhances the production of IL-22 in CD4^+^ T cells by inducing Th17-cell differentiation (66). To investigate the influence of PD feeding on the ileal immune cells, we conducted flow cytometric analysis (Supplementary Fig. 6A). We observed that ILC3s were significantly decreased in the ileum in PD-fed mice compared with that in CD-fed mice (Fig. 6H). Notably, IL-22-producing CD3^−^RORγt^+^ and CD3^−^RORγt^−^ ILCs were significantly decreased in PD-fed mice (Fig. 6I, Supplementary Fig. 6B). Correspondingly, epithelial pSTAT3 levels were significantly reduced in PD-fed mice than in CD-fed Fuji mice (Fig. 6J). The abundance of ileal Th17 cells was significantly lower in PD-fed mice than in those fed the CD (Fig. 6K, Supplementary Fig. 6C), whereas these Th17 cells exhibited only marginal expression of IL-22, with its levels comparable between the two groups (Fig. 6L, Supplementary Fig. 6B). These results indicate that the PD reduces IL-22 production from ILCs and phosphorylation of STAT3 in the ileum.

## Discussion

Multiple studies have examined the relationship between dietary patterns and intestinal homeostasis, including the gut microbiota and intestinal epithelial cells (14, 67); however, most of these studies have used a PD, and few studies have focused on the difference between a PD and a CD. In the present study, we demonstrated that the PD suppresses epithelial turnover, fucosylation, and antimicrobial product generation by altering the gut microbiota.

PD-fed mice exhibited slower epithelial turnover in the ileum, whereas the difference was smaller in the duodenum. As the density of commensal bacteria is much lower in the duodenum than in the ileum (2, 68), the effects of the PD most likely result from alterations in the gut microbiota. The PD did not significantly change the luminal concentrations of SCFAs and lactate or the abundance of lactate-producing bacteria (e.g. *Lactococcus*, *Lactobacillu*s spp., and *Bifidobacterium* spp.) (69) in the ileum. Thus, gut microbe-derived organic acids are dispensable for mediating the effects of the PD. We found that the PD markedly reduced the abundance of SFB, which mainly colonized the ileum. SFB facilitates epithelial cell proliferation by producing retinoic acid (70). Activation of the retinoic acid receptor (RAR) leads to the induction of nitric oxide synthase 2 (Nos2), which inhibits colonization by *Citrobacter rodentium* (70, 71). We observed the downregulation of *Nos2* in PD-fed mice (Supplementary Table S1). Therefore, attenuated epithelial turnover in the ileum of PD-fed mice may result from the underrepresentation of the SFB. However, reduced epithelial turnover in the duodenum should be independent of SFB because it does not colonize the duodenum. However, further investigations are required to clarify the underlying mechanism through which PD feeding attenuates duodenal epithelial turnover.

Upon CD feeding, the small intestine in Inasa mice was shorter than that in Fuji mice. This phenotype may be attributed to the presence or absence of SFB in Fuji or Inasa mice, respectively. Notably, the small intestine in PD-fed Inasa mice was even shorter than that in CD-fed Inasa mice, although their body weights were comparable. It can be speculated that elongation of the small intestine upon CD feeding may result from a high intake of dietary fiber, which is well-known to delay nutrient absorption (72) and thereby elongates the small intestine (73, 74) as an adaptive response to enhance energy absorption.

In a previous study, the number of Olfm4^+^ ISCs in chow-diet-fed mice was reported to be slightly lower than that in PD-fed mice at the steady state (39). However, we did not detect any significant changes in Lgr5^+^ ISCs between CD- and PD-fed mice. This apparent discrepancy between our and previous study may be attributed to the difference in molecular markers for ISCs. Olfm4 has been used extensively as a substitute for Lgr5 to evaluate ISCs. However, recent studies suggest that Olfm4 protein may be expressed not only by ISCs but also by TA progenitor cells (75). Nonetheless, we cannot formally exclude the possibility that differences in other experimental conditions (i.e. composition of the CD used in the studies and indigenous microbial communities) may have influenced the experimental outcomes.

PD-fed mice showed attenuated expression of antimicrobial products such as *Reg3b* and *Reg3g*. The gut microbiota promotes the expression of antimicrobial products in intestinal epithelial cells, mainly by activating TLR-Myd88 signaling and STAT3 (3, 76). STAT3 is activated by IL-22 from ILC3s in response to SFB (3). Although PD feeding did not alter the expression of *Myd88*, it markedly reduced IL-22 production by ILCs and STAT3 phosphorylation in the ileum. Moreover, *Reg3g* expression was significantly decreased in SFB-free Inasa mice. These data indicate that the PD may reduce epithelial STAT3 signaling by limiting SFB colonization, leading to the downregulation of *Reg3b* and *Reg3g* in the ileum.

Lectin staining revealed fucosylation of apical glycans in enterocytes and intracellular granules in the goblet and Paneth cells in the ileum of CD-fed mice. Meanwhile, PD feeding alleviated fucosylation in enterocytes and goblet cells, but not in Paneth cells. These results reflect the differences in the enzymes responsible for fucosylation between Paneth cells and other epithelial cell subsets. Both Fut1 and Fut2 mediate fucosylation in Paneth cells (59), whereas Fut2 alone mediate this process in enterocytes and goblet cells. Notably, PD feeding downregulated *Fut2* but not *Fut1*. Therefore, the fucosylation of Paneth cells was most likely maintained by Fut1 in PD-fed mice. Certain commensal bacteria, such as *Bacteroides* (77) and SFB (12), induce epithelial fucosylation. 16S rRNA gene analysis showed that the abundance of *Bacteroides* was not decreased but rather increased in the mucus layer of PD-fed mice. As mentioned previously, PD feeding significantly decreased the abundance of SFB. SFB induce epithelial fucosylation in the ileum by facilitating IL-22 production from ILC3s (12). IL-22 induces the expression of both Fut2 and B3GNT7 by activating STAT3 (60). Our RNA-sequencing data demonstrated that *B3gnt7* gene expression was significantly downregulated in the ileum of PD-fed mice compared to that in CD-fed mice, most likely because of attenuated STAT3 signaling. Thus, PD feeding limits epithelial fucosylation by inactivating the ILC-IL-22-STAT3 signaling.

The contribution of the ILC3-IL-22 axis to intestinal stemness and epithelial regeneration has been well documented. Early studies demonstrated that ILC3-deficient Lgr5-reporter mice showed significant reduction in the number of Lgr5^+^ ISCs during methotrexate-induced intestinal damage (78), whereas at the steady state, the frequency of Lgr5^+^ ISCs remained unchanged in ILC3-deficient and -sufficient mice. Furthermore, treatment with IL-22 expanded Lgr5-GFP^high^ ISCs on small-intestinal organoids, promoting the expansion of organoids (79). Likewise, IL-22 administration increased the recovery of Lgr5^+^ ISCs and epithelial regeneration in a graft-versus-host disease mice model. In contrast, recent studies showed that treatment with IL-22 facilitated intestinal organoid growth, while reducing the number of Lgr5^+^ ISCs (80, 81). Similarly, *in vivo* administration of IL-22 reduced the number of Lgr5^+^ ISCs by inhibiting Wnt and Notch signaling (80). Thus, the role of IL-22 in intestinal stemness remains controversial. Here, we found that PD feeding significantly decreased IL-22 production by ILCs and reduced epithelial cell turnover without affecting the number of ISCs. Considering that *IL-22Ra1* is expressed by TA progenitors and IL-22 enhances TA cell proliferation (81), it is plausible that the attenuated epithelial cell turnover in PD-fed mice may result from reduced proliferation of TA cells due to the downregulation of IL-22.

Multiple lines of evidence suggest that PPARα signaling is activated during fasting in the liver to promote fatty acid oxidation and ketogenesis (56). Fasting also activates PPAR target molecules, including *Pdk4*, *Hmgcs2*, and *Fabp1* in small intestinal epithelial cells (57). We observed that PD feeding upregulated the expression of these PPARα target molecules at both transcriptional and protein levels in the ileal epithelium. HMGCS2 is a rate-limiting enzyme in ketogenesis that converts acetyl-CoA and acetoacetyl-CoA to HMG-CoA and CoA (56, 82). PDK4 is a critical enzyme that converts cellular energy from carbohydrates to lipids by inhibiting the synthesis of pyruvate to acetyl-CoA (83). FABP1, an intracellular protein highly expressed in the liver and intestines (84, 85), is essential for fatty acid uptake and basolateral secretion in differentiated enterocytes (86). Further, FABP1 interacts with PPARα and upregulates its transcriptional activity (87). Thus, a PD is thought to induce the metabolic rewiring of fatty acid oxidation and ketogenesis, which is reminiscent of the metabolic state in the intestinal epithelium of fasted mice. In addition, short-term (12 h) fasting downregulates the expression of antimicrobial products, including REG3γ, by reducing SFB (64). In the present study, we demonstrated that the PD decreased the abundance of SFB, leading to attenuated epithelial turnover, fucosylation, and generation of antimicrobial products. These data indicate that the PD also promotes a gut microbial community in the ileal mucus layer, similar to that in fasted mice. In contrast, RNA-sequencing data showed that genes involved in carbohydrate metabolism were upregulated in the duodenum of PD-fed mice, implying that the duodenal epithelium utilizes carbohydrates as a significant energy source. We speculate that most of the nutrients, especially carbohydrates, in the PD are digested and absorbed in the upper small intestine before reaching the ileum, which makes the ileum in undernutrition. SFB attachment to the ileal epithelium is suppressed by fasting (54, 64), suggesting that sufficient nutritional status in the ileal lumen by feeding is essential for SFB colonization. Thus, the PD may limit SFB colonization by making the ileum undernutrition, similar to a fasting state.

In conclusion, we found that the PD altered the gut microbiota, characterized by the underrepresentation of SFB, which led to the suppression of epithelial barrier functions such as epithelial turnover, fucosylation, and antimicrobial product generation. In addition, the PD facilitates metabolic rewiring to fatty acid oxidation and ketogenesis by activating PPARα signaling. Thus, we provide a new perspective on the importance of dietary composition in nutritional research.

### Limitation of study and direction for future study

We found that PD feeding suppresses the growth of SFB, suggesting that a particular dietary ingredient in the CD is necessary to maintain SFB in the ileum. However, it remains unknown. Furthermore, the underlying mechanism by which PD feeding attenuates epithelial proliferation and antimicrobial product expression in the duodenum has yet to be clarified.

## Supplementary Material

dxae003_suppl_Supplementary_Tables

dxae003_suppl_Supplementary_Figures

## Data Availability

The data in this study are available from the corresponding author, K.H., upon reasonable request.
