# Supplementary material for: A purified diet affects intestinal epithelial proliferation and barrier functions through gut microbial alterations
Source: Int Immunol. 2024 Jan 23;36(5):223–40. doi: 10.1093/intimm/dxae003 (PMC10989658; doi:10.1093/intimm/dxae003)
Supplement: dxae003_suppl_Supplementary_Tables [file dxae003_suppl_supplementary_tables.pdf]

Table S1. Nutritional formula of CD and PD

Diet	CD*	PD
Protein (gm%)	25.05	20
Fat (gm%)	4.77	7
Carbohydrate (gm%)	49.82 (Nitrogen Free Extract)	64
kcal/gm	3.424	4

*Average of periodic analysis in 2022

Table S2. Differentially expressed genes between CD and PD epithelium

A) List of genes downregulated in the ileum of PD-fed mice

ENSMUSG	Gene names	log2FoldChange	adjusted p-value
ENSMUSG00000074115	Saa1	-5.696143481	9.37E-16
ENSMUSG00000032068	Plet1	-5.426979882	2.39E-10
ENSMUSG00000079547	H2-DMb1	-4.941358883	2.13E-166
ENSMUSG00000079180	Mptx2	-4.757679766	4.06E-35
ENSMUSG00000068349	Gml	-4.609812713	4.54E-05
ENSMUSG00000043931	Gimap7	-4.537923337	5.37E-10
ENSMUSG00000035042	Ccl5	-4.46707985	3.73E-63
ENSMUSG00000004612	Nkg7	-4.306981062	6.72E-11
ENSMUSG00000023132	Gzma	-4.22160491	1.00E-52
ENSMUSG00000015437	Gzmb	-4.172048926	4.06E-31
ENSMUSG00000076752	Trgc2	-4.171545678	3.73E-18
ENSMUSG00000024610	Cd74	-4.134496558	6.71E-101
ENSMUSG00000073421	H2-Ab1	-4.046034784	4.80E-130
ENSMUSG00000035186	Ubd	-4.033524879	6.81E-11
ENSMUSG00000036594	H2-Aa	-3.991245868	2.67E-155
ENSMUSG00000031089	Slc6a14	-3.977614433	0.000962376
ENSMUSG00000075602	Ly6a	-3.733208013	4.87E-05
ENSMUSG00000008461	Fut1	-3.67722283	0.010643215
ENSMUSG00000037095	Lrg1	-3.618620805	0.009187279
ENSMUSG00000041193	Pla2g5	-3.618278396	2.41E-32
ENSMUSG00000001670	Tat	-3.598790085	0.012370556
ENSMUSG00000029120	Ppp2r2c	-3.558458317	0.035676384
ENSMUSG00000023931	Efhb	-3.553678148	0.038672583
ENSMUSG00000069324	Bhmt1b	-3.51065401	0.020752792
ENSMUSG00000100150	Gm19585	-3.467949416	0.000369602
ENSMUSG00000076498	Trbc2	-3.449952354	4.95E-14
ENSMUSG00000021384	Susd3	-3.449947978	0.023781545
ENSMUSG00000060586	H2-Eb1	-3.422245314	3.02E-156
ENSMUSG00000068600	Gml2	-3.39822656	0.026788152
ENSMUSG00000055978	Fut2	-3.392095642	7.12E-20
ENSMUSG00000026117	Zap70	-3.294498555	0.016319231
ENSMUSG00000036322	H2-Ea	-3.258786186	8.44E-52
ENSMUSG00000027514	Zbp1	-3.225879515	6.98E-28
ENSMUSG00000034028	Cd226	-3.192502189	0.003408158
ENSMUSG00000053977	Cd8a	-3.167102916	1.70E-16
ENSMUSG00000037649	H2-DMa	-3.146831369	1.56E-39
ENSMUSG00000022504	Ciita	-3.138441147	2.06E-33
ENSMUSG00000102418	Sh2d1b1	-3.123440834	0.005393929
ENSMUSG00000029417	Cxcl9	-3.078714532	3.12E-08
ENSMUSG00000022723	Crybg3	-3.078069681	0.000498526
ENSMUSG00000052374	Actn2	-3.072834057	0.048090968
ENSMUSG00000029204	Rhoh	-3.044497888	0.001527109
ENSMUSG00000082171	Rpl27-ps2	-3.018090992	0.001061784
ENSMUSG00000024863	Mbl2	-2.984227898	0.001191412
ENSMUSG00000020826	Nos2	-2.959327461	2.90E-29
ENSMUSG00000054169	Ceacam10	-2.946724093	1.05E-13
ENSMUSG00000040026	Saa3	-2.940018407	0.012161377
ENSMUSG00000034634	Ly6d	-2.929225817	0.000506125
ENSMUSG00000030173	Klra5	-2.929070816	4.96E-06
ENSMUSG00000071158	Klrh1	-2.91153632	3.92E-05
ENSMUSG00000090254	Gm1965	-2.901066632	0.023341681
ENSMUSG00000002033	Cd3g	-2.890936224	1.46E-10
ENSMUSG00000027636	Sla2	-2.875753379	0.043432933
ENSMUSG00000074183	Gsta1	-2.871095855	1.37E-32
ENSMUSG00000027225	Duoxa2	-2.847151659	2.67E-55
ENSMUSG00000036833	Pnpla7	-2.810597192	0.000183961
ENSMUSG00000030775	Trat1	-2.785469261	0.022779044
ENSMUSG00000060240	Cend1	-2.775522563	0.044916321
ENSMUSG00000089614	Gm23505	-2.761100601	0.003602444
ENSMUSG00000025163	Cd7	-2.757376279	5.42E-16
ENSMUSG00000040264	Gbp2b	-2.748132215	1.66E-11
ENSMUSG00000032094	Cd3d	-2.727525324	6.69E-07
ENSMUSG00000022657	Cd96	-2.725385214	0.019685121
ENSMUSG00000034271	Jdp2	-2.703438502	2.85E-06
ENSMUSG00000024677	Ms4a6b	-2.667953901	7.41E-05
ENSMUSG00000062082	Cd200r4	-2.658316935	0.000834551
ENSMUSG00000070427	Il18bp	-2.645157874	2.76E-05
ENSMUSG00000002076	Hsf2bp	-2.615445779	0.003491564
ENSMUSG00000076754	Trgv2	-2.580378469	0.003399773
ENSMUSG00000045826	Ptprcap	-2.547639917	0.026991512
ENSMUSG00000024027	Glp1r	-2.457593663	0.035533028
ENSMUSG00000068227	Il2rb	-2.453406143	1.77E-05
ENSMUSG00000076613	Ighg2b	-2.421259495	0.010450098
ENSMUSG00000054072	Iigp1	-2.41612046	0.000914034
ENSMUSG00000071715	Ncf4	-2.403228464	0.046749389
ENSMUSG00000040345	Arhgap9	-2.375618099	0.007556482
ENSMUSG00000071356	Reg3b	-2.365344216	8.36E-30
ENSMUSG00000013653	1810065E05	-2.361442719	1.70E-70
ENSMUSG00000074892	B3galt5	-2.349054996	0.000151277
ENSMUSG00000078920	Ifi47	-2.335820694	1.89E-22
ENSMUSG00000052212	Cd177	-2.335350568	2.59E-08
ENSMUSG00000030017	Reg3g	-2.322211384	5.73E-42
ENSMUSG00000078853	Igtp	-2.312359601	3.20E-24
ENSMUSG00000073418	C4b	-2.307635452	0.03056972
ENSMUSG00000028965	Tnfrsf9	-2.302952688	0.004505281
ENSMUSG00000039264	Gimap3	-2.287555087	0.005230782
ENSMUSG00000032093	Cd3e	-2.283221685	0.000509931
ENSMUSG00000038323	1700066M21	-2.276969607	0.007924553
ENSMUSG00000085696	Hoxaas3	-2.276757195	0.032407168
ENSMUSG00000076441	Ass1	-2.273287854	0.027138662
ENSMUSG00000079260	Tmppe	-2.254732547	0.046461791
ENSMUSG00000009687	Fxyd5	-2.245234726	0.003287939
ENSMUSG00000005763	Cd247	-2.235975507	0.010171617
ENSMUSG00000040328	Or2v1	-2.211428976	0.018832931
ENSMUSG00000024575	Pde6a	-2.160784675	0.019614473
ENSMUSG00000079116	Defa41	-2.145450975	6.71E-06
ENSMUSG00000083649	Rasl2-9	-2.129138465	0.004607303
ENSMUSG00000060063	Alox5ap	-2.126809887	1.07E-05
ENSMUSG00000056737	Capg	-2.126534984	4.76E-05
ENSMUSG00000020143	Dock2	-2.10983141	0.027846155
ENSMUSG00000025092	Hspa12a	-2.106306785	3.48E-25
ENSMUSG00000028270	Gbp2	-2.069727437	0.000667002
ENSMUSG00000054435	Gimap4	-2.044784807	3.51E-05
ENSMUSG00000024338	Psmb8	-2.034243938	5.00E-47
ENSMUSG00000020407	Upp1	-2.026273864	2.39E-33
ENSMUSG00000079339	Ifit1bl1	-2.012074828	1.33E-13
ENSMUSG00000021720	Rnf180	-2.00188363	0.002219141
ENSMUSG00000033213	AA467197	-1.997438642	1.96E-15
ENSMUSG00000009246	Trpm5	-1.994948232	6.91E-05
ENSMUSG00000031360	Ctps2	-1.976363866	0.021723876
ENSMUSG00000022220	Adcy4	-1.947250619	0.008698095
ENSMUSG00000060675	Plaat3	-1.945661881	1.69E-35
ENSMUSG00000027009	Itga4	-1.93724031	0.021452274
ENSMUSG00000016496	Cd274	-1.936657749	0.002402177
ENSMUSG00000079363	Gbp4	-1.935292493	0.000657803
ENSMUSG00000031101	Sash3	-1.919864637	0.030229682
ENSMUSG00000048521	Cxcr6	-1.907002458	0.009191814

B) List of genes upregulated in the ileum of PD-fed mice

ENSMUSG	Gene names	log2FoldChange	adjusted p-value
ENSMUSG00000066072	Cyp4a10	7.943393494	2.43E-44
ENSMUSG00000041536	Serpina3a	5.069578932	2.76E-37
ENSMUSG00000050201	Otop2	4.646086882	4.11E-05
ENSMUSG00000025905	Oprk1	4.62084184	1.34E-16
ENSMUSG00000027875	Hmgcs2	4.426576549	3.97E-42
ENSMUSG00000051323	Pcdh19	4.412878289	3.56E-14
ENSMUSG00000028571	Cyp2j13	4.333945351	5.21E-06
ENSMUSG00000021228	Acot3	3.951961052	0.001229375
ENSMUSG00000027801	Tm4sf4	3.682958924	1.76E-43
ENSMUSG00000106397	Gm21049	3.635771818	3.76E-19
ENSMUSG00000027961	Lrrc39	3.602948677	1.53E-10
ENSMUSG00000034687	Fras1	3.576118979	1.89E-08
ENSMUSG00000041828	Abca8a	3.498572928	0.038369936
ENSMUSG00000031355	Arhgap6	3.382653391	0.030717512
ENSMUSG00000015224	Cyp2j9	3.376844505	2.63E-11
ENSMUSG00000072949	Acot1	3.222473449	1.10E-20
ENSMUSG00000054422	Fabp1	3.2161508	2.86E-39
ENSMUSG00000024682	Cblif	3.155799704	1.35E-06
ENSMUSG00000043496	Tril	3.022521674	0.016653232
ENSMUSG00000050097	Ces2b	3.00382958	6.16E-20
ENSMUSG00000056973	Ces1d	2.929956926	2.19E-36
ENSMUSG00000062515	Fabp4	2.900609361	1.34E-07
ENSMUSG00000074639	Rdh16f2	2.863103856	2.66E-25
ENSMUSG00000020620	Abca8b	2.814454711	5.10E-07
ENSMUSG00000067229	Cyp2c66	2.792211055	1.37E-93
ENSMUSG00000050666	Vstm4	2.763625252	0.005376535
ENSMUSG00000043448	Gjc2	2.755046326	0.000900876
ENSMUSG00000026354	Lct	2.744697297	3.56E-12
ENSMUSG00000029236	Nmu	2.735867246	0.020589035
ENSMUSG00000064140	Trim38	2.733345848	6.80E-16
ENSMUSG00000035357	Pdzrn3	2.731062593	0.004052349
ENSMUSG00000056666	Retsat	2.726761554	1.10E-56
ENSMUSG00000027761	Aadac	2.702152233	8.18E-29
ENSMUSG00000051079	Rgs13	2.691247852	7.39E-06
ENSMUSG00000059824	Dbp	2.684648217	0.018943183
ENSMUSG00000006205	Htra1	2.679304944	0.018763227
ENSMUSG00000074207	Adh1	2.65732388	1.59E-168
ENSMUSG00000051748	Wfdc21	2.643202891	0.000299811
ENSMUSG00000022181	C6	2.641351214	0.004129941
ENSMUSG00000052415	Tchh	2.635110368	0.046978647
ENSMUSG00000042812	Foxf1	2.631653831	0.000365416
ENSMUSG00000021765	Fst	2.619560274	0.003693273
ENSMUSG00000025726	Slc28a1	2.554859554	2.03E-08
ENSMUSG00000056569	Mpz	2.553768527	0.00691414
ENSMUSG00000015401	Cltrn	2.496383874	0.007209135
ENSMUSG00000042750	Bex2	2.490218453	0.001765034
ENSMUSG00000027690	Slc2a2	2.489854565	1.39E-150
ENSMUSG00000047992	Dipk1c	2.483936239	0.027867911
ENSMUSG00000027887	Sypl2	2.458113197	9.51E-05
ENSMUSG00000021719	Rgs7bp	2.456541835	0.004571209
ENSMUSG00000046213	Cym	2.448790207	1.17E-128
ENSMUSG00000047414	Flrt2	2.448683097	0.001118985
ENSMUSG00000003949	Hlf	2.444638197	6.53E-11
ENSMUSG00000048218	Amigo2	2.403359965	0.002137383
ENSMUSG00000040134	Rdh7	2.390003158	7.83E-51
ENSMUSG00000034845	Plvap	2.385305656	9.41E-05
ENSMUSG00000028167	Bdh2	2.329206718	3.67E-16
ENSMUSG00000018822	Sfrp5	2.325317441	1.17E-11
ENSMUSG00000038143	Stox2	2.307661563	0.036211525
ENSMUSG00000037071	Scd1	2.29678469	0.000101389
ENSMUSG00000036856	Wnt4	2.274869811	0.03061777
ENSMUSG00000047502	Mroh7	2.2714337	0.027859551
ENSMUSG00000096971	4930556M19	2.265484162	0.041573612
ENSMUSG00000010651	Acaa1b	2.244686597	8.85E-13
ENSMUSG00000001870	Ltbp1	2.222221972	0.000129694
ENSMUSG00000045928	4933440M02	2.214588274	1.37E-07
ENSMUSG00000021226	Acot2	2.189017355	2.55E-24
ENSMUSG00000052160	Pld4	2.185599915	0.030031535
ENSMUSG00000015962	1700016C15	2.184970029	5.90E-06
ENSMUSG00000037492	Zmat4	2.178409638	2.63E-05
ENSMUSG00000091649	Phf11b	2.163982507	0.001025936
ENSMUSG00000024268	Celf4	2.155321212	0.017488323
ENSMUSG00000021792	Prxl2a	2.153241594	3.89E-198
ENSMUSG00000030048	Gkn3	2.15280481	8.30E-05
ENSMUSG00000024672	Ms4a7	2.137115388	0.000843846
ENSMUSG00000086914	Gm16124	2.132151144	0.004556121
ENSMUSG00000069835	Sat2	2.125231652	0.043161784
ENSMUSG00000024354	Slc23a1	2.117456256	0.00063384
ENSMUSG00000054932	Afp	2.117361512	1.15E-82
ENSMUSG00000031073	Fgf15	2.09216855	4.21E-166
ENSMUSG00000002944	Cd36	2.084048578	9.76E-11
ENSMUSG00000070873	Lilra5	2.082079867	0.017854923
ENSMUSG00000020787	P2rx1	2.027577266	0.014791005
ENSMUSG00000024245	Tmem178	2.027196235	0.031417028
ENSMUSG00000097203	4732419C18	2.012231164	0.000353454
ENSMUSG00000027800	Tm4sf1	2.011622185	0.008460808
ENSMUSG00000025930	Msc	2.006193891	0.001677762
ENSMUSG00000020658	Efr3b	1.986909725	2.40E-11
ENSMUSG00000046997	Spsb4	1.979495153	0.037632198
ENSMUSG00000030102	Itpr1	1.951623151	2.46E-52
ENSMUSG00000031387	Renbp	1.94426774	0.000595133
ENSMUSG00000094686	Ccl21a	1.932429686	0.032537862
ENSMUSG00000022098	Bmp1	1.931494359	1.72E-07
ENSMUSG00000057074	Ces1g	1.895909082	1.90E-20
ENSMUSG00000019577	Pdk4	1.89213252	2.03E-06
ENSMUSG00000032076	Cadm1	1.87845287	0.014054383
ENSMUSG00000021539	Lect2	1.848593132	0.000688547
ENSMUSG00000029309	Sparcl1	1.847400833	7.97E-12
ENSMUSG00000026657	Frmd4a	1.843234355	0.04447606
ENSMUSG00000086382	Chrna1os	1.835072561	0.000741664
ENSMUSG00000118449	Gm3625	1.832604863	0.013014001
ENSMUSG00000044986	Tst	1.827319197	0.000941387
ENSMUSG00000028755	Cda	1.815791626	2.08E-24
ENSMUSG00000031548	Sfrp1	1.815213991	0.006683715
ENSMUSG00000074896	Ifit3	1.811586327	7.55E-16
ENSMUSG00000020889	Nr1d1	1.809574651	4.96E-05
ENSMUSG00000064325	Hhip	1.80783527	1.57E-06
ENSMUSG00000085315	A430018G15	1.806776174	0.000657344
ENSMUSG00000022197	Pdzd2	1.796805726	0.001716018
ENSMUSG00000021730	Hcn1	1.792118188	9.89E-11
ENSMUSG00000039542	Ncam1	1.791717356	0.037281104
ENSMUSG00000050953	Gja1	1.791381829	0.013632486
ENSMUSG00000055737	Ghr	1.778874151	4.02E-10
ENSMUSG00000017737	Mmp9	1.776797104	0.016319231
ENSMUSG00000021609	Slc6a3	1.775015876	6.02E-20
ENSMUSG00000021835	Bmp4	1.765457959	1.15E-06
ENSMUSG00000035896	Rnase1	1.758763709	2.97E-08

C) List of genes downregulated in the duodenum of PD-fed mice

ENSMUSG	SYMBOL	log2FoldChange	adjusted p-value
ENSMUSG00000003053	Cyp2c29	-5.260768045	6.37E-60
ENSMUSG00000061906	Ugt2b38	-4.932813148	0.000789259
ENSMUSG00000032315	Cyp1a1	-4.73679181	1.23E-09
ENSMUSG00000029762	Akr1b8	-3.918581885	4.72E-12
ENSMUSG00000034258	Flvcr2	-3.888652897	1.16E-21
ENSMUSG00000052373	Mpp3	-3.835345042	0.001803626
ENSMUSG00000063851	Rnf183	-3.818917455	0.00518228
ENSMUSG00000026418	Tnni1	-3.696568641	0.000172223
ENSMUSG00000041193	Pla2g5	-3.57788612	2.41E-32
ENSMUSG00000025243	Slc6a20b	-3.516872202	8.21E-05
ENSMUSG00000046807	Lrrc75b	-3.497259356	0.000550758
ENSMUSG00000030775	Trat1	-3.464783403	0.022779044
ENSMUSG00000005763	Cd247	-3.460568687	0.010171617
ENSMUSG00000004151	Etv1	-3.457507378	0.00149941
ENSMUSG00000032122	Slc37a2	-3.423449275	0.003269137
ENSMUSG00000030554	Synm	-3.401387536	0.015891634
ENSMUSG00000071356	Reg3b	-3.39799283	8.36E-30
ENSMUSG00000066072	Cyp4a10	-3.317742015	2.43E-44
ENSMUSG00000025002	Cyp2c55	-3.231214919	1.46E-47
ENSMUSG00000030762	Aqp8	-3.04069013	1.56E-122
ENSMUSG00000030483	Cyp2b10	-3.002852472	1.80E-39
ENSMUSG00000078612	Fyb2	-2.971947086	1.37E-06
ENSMUSG00000002384	Bmp8b	-2.955176561	0.002773712
ENSMUSG00000067229	Cyp2c66	-2.94902256	1.37E-93
ENSMUSG00000034028	Cd226	-2.915460463	0.003408158
ENSMUSG00000047992	Dipk1c	-2.892095509	0.027867911
ENSMUSG00000055748	Gsdmc4	-2.876133524	1.02E-19
ENSMUSG00000063683	Glyat	-2.867118941	0.034896114
ENSMUSG00000027015	Cybrd1	-2.846747176	0.004117035
ENSMUSG00000042988	Notum	-2.733896188	0.000549422
ENSMUSG00000070056	Mfhas1	-2.709150754	7.35E-31
ENSMUSG00000024268	Celf4	-2.670588377	0.017488323
ENSMUSG00000074004	B3gnt6	-2.613769123	0.00659336
ENSMUSG00000029603	Dtx1	-2.558717315	0.036163918
ENSMUSG00000033847	Pla2g4c	-2.557155015	0.000187951
ENSMUSG00000030087	Klf15	-2.534767492	0.010434514
ENSMUSG00000052374	Actn2	-2.475954027	0.048090968
ENSMUSG00000068600	Gml2	-2.467200676	0.026788152
ENSMUSG00000020653	Klf11	-2.466053675	0.010733749
ENSMUSG00000037579	Kcnh3	-2.463667612	0.001725962
ENSMUSG00000056293	Gsdmc2	-2.434757082	1.06E-17
ENSMUSG00000079180	Mptx2	-2.434524819	4.06E-35
ENSMUSG00000042115	Klhdc8a	-2.427237936	0.01271405
ENSMUSG00000028435	Aqp3	-2.421756996	4.13E-07
ENSMUSG00000058135	Gstm1	-2.408345029	2.04E-42
ENSMUSG00000028680	Plk3	-2.395337822	0.003875591
ENSMUSG00000008999	Bmp7	-2.385100769	2.13E-05
ENSMUSG00000026581	Sell	-2.350676836	0.005968545
ENSMUSG00000051735	Rinl	-2.34116956	0.027753045
ENSMUSG00000022857	Tmprss15	-2.334694215	2.15E-197
ENSMUSG00000030173	Klra5	-2.267086537	4.96E-06
ENSMUSG00000050288	Fzd2	-2.210450908	0.020694027
ENSMUSG00000092570	Plut	-2.193819848	1.32E-40
ENSMUSG00000002033	Cd3g	-2.153775055	1.46E-10
ENSMUSG00000076752	Trgc2	-2.148978876	3.73E-18
ENSMUSG00000042817	Flt3	-2.138956749	0.034537167
ENSMUSG00000073424	Cyp4f15	-2.131533757	1.15E-05
ENSMUSG00000039994	Timeless	-2.116375892	0.000881497
ENSMUSG00000025726	Slc28a1	-2.113589764	2.03E-08
ENSMUSG00000048163	Selplg	-2.111991764	0.020438256
ENSMUSG00000022220	Adcy4	-2.107174946	0.008698095
ENSMUSG00000071158	Klrh1	-2.099382101	3.92E-05
ENSMUSG00000040010	Slc7a5	-2.084229474	0.029843409
ENSMUSG00000021384	Susd3	-2.082221299	0.023781545
ENSMUSG00000088990	Gm22767	-2.071533414	0.002237261
ENSMUSG00000027225	Duoxa2	-2.065160597	2.67E-55
ENSMUSG00000053977	Cd8a	-2.063035233	1.70E-16
ENSMUSG00000024533	Spire1	-2.051509258	0.018591429
ENSMUSG00000032278	Paqr5	-2.0509008	2.18E-07
ENSMUSG00000003555	Cyp17a1	-2.017705948	0.008101647
ENSMUSG00000030017	Reg3g	-2.01766772	5.73E-42
ENSMUSG00000010064	Slc38a3	-1.977354838	7.32E-05
ENSMUSG00000113204	Gm46430	-1.953777477	0.026824258
ENSMUSG00000002076	Hsf2bp	-1.95290562	0.003491564
ENSMUSG00000065208	Gm24616	-1.950267639	0.00536353
ENSMUSG00000056724	Nbeal2	-1.941459365	0.000199188
ENSMUSG00000037762	Slc16a9	-1.939885648	0.001527026
ENSMUSG00000000805	Car4	-1.92844588	2.52E-12
ENSMUSG00000120391	Gm31308	-1.917561036	9.79E-05
ENSMUSG00000109305	Smim38	-1.91220812	0.006244318
ENSMUSG00000065956	Defa37	-1.906610184	0.005908885
ENSMUSG00000024030	Abcg1	-1.903069997	0.005278011
ENSMUSG00000079114	Defa42	-1.898468141	7.51E-06
ENSMUSG00000018554	Ybx2	-1.886250771	0.017814172
ENSMUSG00000004038	Gstm3	-1.871422839	1.44E-59
ENSMUSG00000074179	Gsta5	-1.842606823	8.45E-24
ENSMUSG00000041644	Slc5a12	-1.834044825	0.039837035
ENSMUSG00000020226	Slc5a4b	-1.828250077	4.45E-09
ENSMUSG00000074604	Mgst2	-1.82437709	3.02E-16
ENSMUSG00000030089	Slc41a3	-1.810933473	1.41E-14
ENSMUSG00000050762	Prss27	-1.80537022	0.009133556
ENSMUSG00000057058	Skap1	-1.798473794	0.026318125
ENSMUSG00000051359	Ncald	-1.795311633	8.72E-15
ENSMUSG00000018862	Otop3	-1.789034163	2.77E-16
ENSMUSG00000088254	Gm24289	-1.779779938	0.000105645
ENSMUSG00000000531	Tamalin	-1.770182397	0.020698169
ENSMUSG00000021108	Prkch	-1.746597361	0.021160302
ENSMUSG00000050097	Ces2b	-1.745151217	6.16E-20
ENSMUSG00000022504	Ciita	-1.729775078	2.06E-33
ENSMUSG00000027513	Pck1	-1.728732379	1.48E-08
ENSMUSG00000035186	Ubd	-1.725064308	6.81E-11
ENSMUSG00000089960	Ugt1a1	-1.705202876	5.94E-05
ENSMUSG00000087366	Junos	-1.696849175	0.010453859
ENSMUSG00000023931	Efhb	-1.690870486	0.038672583
ENSMUSG00000037440	Vnn1	-1.683418246	4.37E-37
ENSMUSG00000009614	Sardh	-1.68273763	0.003884172
ENSMUSG00000120485	Gm35340	-1.675894063	2.97E-06
ENSMUSG00000019178	Styxl1	-1.670016576	0.001281323
ENSMUSG00000027935	Rab13	-1.666481309	0.003649798
ENSMUSG00000030165	Klrd1	-1.663874554	0.016054813
ENSMUSG00000026404	Ddx59	-1.652753435	0.024474389
ENSMUSG00000027870	Hao2	-1.651142771	0.000276627
ENSMUSG00000063522	Ly6m	-1.630010371	3.14E-20
ENSMUSG00000057074	Ces1g	-1.62814222	1.90E-20
ENSMUSG00000028262	Clca3a2	-1.623581588	6.87E-11
ENSMUSG00000023132	Gzma	-1.618708461	1.00E-52
ENSMUSG00000060675	Plaat3	-1.608729253	1.69E-35

D) List of genes upregulated in the duodenum of PD-fed mice

ENSMUSG	SYMBOL	log2FoldChange	adjusted p-value
ENSMUSG00000024266	Adad2	4.259028148	2.46E-05
ENSMUSG00000037593	Rskr	3.497563341	0.008211049
ENSMUSG00000024421	Lama3	3.262671217	1.49E-13
ENSMUSG00000046329	Slc25a23	3.139043967	0.004528787
ENSMUSG00000026249	Serpine2	3.082927776	0.046749389
ENSMUSG00000015401	Cltrn	3.049307175	0.007209135
ENSMUSG00000067235	H2-Q10	3.009335741	2.48E-39
ENSMUSG00000050953	Gja1	2.873491981	0.013632486
ENSMUSG00000003949	Hlf	2.853008368	6.53E-11
ENSMUSG00000016494	Cd34	2.729866184	0.011209708
ENSMUSG00000018648	Dusp14	2.728611716	0.000184746
ENSMUSG00000023073	Slc10a2	2.727732897	2.45E-39
ENSMUSG00000040181	Fmo1	2.714496752	0.018345782
ENSMUSG00000058331	Zfp85	2.679974887	0.012236827
ENSMUSG00000034863	Ano8	2.676383272	0.016284208
ENSMUSG00000028542	Slc6a9	2.619760536	4.38E-07
ENSMUSG00000001014	Icam4	2.613948386	0.013351547
ENSMUSG00000049555	Tmie	2.60540566	1.20E-10
ENSMUSG00000005681	Apoa2	2.596477204	9.04E-07
ENSMUSG00000043673	Kcns3	2.595005267	9.94E-13
ENSMUSG00000096696	Zfp960	2.548843475	0.048902709
ENSMUSG00000073094	Smim9	2.529552659	0.000369346
ENSMUSG00000083649	Rasl2-9	2.490842589	0.004607303
ENSMUSG00000027995	Tlr2	2.406621404	0.009715968
ENSMUSG00000022181	C6	2.379536612	0.004129941
ENSMUSG00000059631	1500035N22	2.378520129	8.72E-08
ENSMUSG00000024738	Pga5	2.369800544	0.001161853
ENSMUSG00000102418	Sh2d1b1	2.359914014	0.005393929
ENSMUSG00000032500	Dclk3	2.358942462	0.040924215
ENSMUSG00000054582	Pabpc1l	2.354761989	0.026874588
ENSMUSG00000020492	Ska2	2.298865257	0.010580194
ENSMUSG00000073557	Ppp1r12b	2.294225045	0.013807886
ENSMUSG00000032860	P2ry2	2.28608878	4.28E-13
ENSMUSG00000007594	Hapln4	2.284813183	0.011454977
ENSMUSG00000050761	Gp1bb	2.278724544	9.06E-15
ENSMUSG00000037216	Lipt1	2.266027513	0.035234992
ENSMUSG00000053835	H2-T24	2.26043167	1.57E-06
ENSMUSG00000043391	2510009E07	2.249089354	7.04E-07
ENSMUSG00000095105	Edaradd	2.225459859	8.83E-09
ENSMUSG00000078963	Hsbp1l1	2.220536376	0.01282466
ENSMUSG00000027188	Pamr1	2.194386183	0.007556482
ENSMUSG00000106352	5033403H07	2.089850136	0.000180467
ENSMUSG00000042216	Sgsm1	2.082951805	0.021540694
ENSMUSG00000051022	Hs3st1	2.074744402	0.000137127
ENSMUSG00000023345	Poc1a	2.072828124	0.009900661
ENSMUSG00000040711	Sh3pxd2b	2.060026997	0.028016101
ENSMUSG00000028976	Slc2a5	2.037553423	4.44E-135
ENSMUSG00000021719	Rgs7bp	2.03624125	0.004571209
ENSMUSG00000032816	Igdcc4	2.02814324	0.000735619
ENSMUSG00000016382	Pls3	2.013736148	0.014497077
ENSMUSG00000016239	Lonrf3	2.001847063	0.02617699
ENSMUSG00000069805	Fbp1	1.913401881	8.73E-136
ENSMUSG00000039633	Lonrf1	1.910229997	0.00415548
ENSMUSG00000042750	Bex2	1.903955696	0.001765034
ENSMUSG00000000093	Tbx2	1.900336936	0.047773101
ENSMUSG00000046658	Zfp316	1.897264741	0.032543912
ENSMUSG00000021228	Acot3	1.893501187	0.001229375
ENSMUSG00000022197	Pdzd2	1.891201864	0.001716018
ENSMUSG00000028199	Cryz	1.872741653	2.29E-05
ENSMUSG00000048332	Lhfpl6	1.864498146	0.000273719
ENSMUSG00000022435	Upk3a	1.849470461	0.009203445
ENSMUSG00000031387	Renbp	1.847096262	0.000595133
ENSMUSG00000018395	Kif3a	1.815587184	0.007556482
ENSMUSG00000026705	Klhl20	1.803859554	0.032848588
ENSMUSG00000041681	Iapp	1.798803639	0.000111281
ENSMUSG00000043629	1700019D03	1.797065085	2.77E-18
ENSMUSG00000027887	Sypl2	1.781923365	9.51E-05
ENSMUSG00000066682	Pilrb2	1.778960698	0.002476358
ENSMUSG00000096971	4930556M19	1.777246909	0.041573612
ENSMUSG00000033898	Cfhr2	1.749431048	0.006576403
ENSMUSG00000062488	Ifit3b	1.748042399	3.32E-12
ENSMUSG00000091813	Ces2h	1.746857209	0.000269614
ENSMUSG00000028307	Aldob	1.741539455	2.59E-132
ENSMUSG00000052560	Cpne8	1.734154008	7.88E-09
ENSMUSG00000074896	Ifit3	1.733797021	7.55E-16
ENSMUSG00000096965	3300005D01	1.729476929	4.47E-10
ENSMUSG00000121492	6820431F20	1.725517782	0.007786579
ENSMUSG00000020333	Acsl6	1.714984531	3.63E-13
ENSMUSG00000086727	4931428L18	1.711842524	0.027736522
ENSMUSG00000036057	Ptpn23	1.707081185	0.011620851
ENSMUSG00000048782	Insc	1.676947018	4.09E-05
ENSMUSG00000020121	Srgap1	1.656550461	0.001707449
ENSMUSG00000064225	Paqr9	1.634491525	1.23E-101
ENSMUSG00000000125	Wnt3	1.62372432	1.17E-05
ENSMUSG00000048218	Amigo2	1.621017291	0.002137383
ENSMUSG00000033715	Akr1c14	1.604684485	1.85E-11
ENSMUSG00000068245	Phf11d	1.600018204	3.20E-10
ENSMUSG00000033906	Zdhhc15	1.591255309	0.015826695
ENSMUSG00000021730	Hcn1	1.57307131	9.89E-11
ENSMUSG00000001288	Rarg	1.553802092	0.00068699
ENSMUSG00000075254	Heg1	1.552033681	0.021349288
ENSMUSG00000039395	Mreg	1.54822259	1.16E-08
ENSMUSG00000016028	Celsr1	1.540873255	0.007264573
ENSMUSG00000023094	Msrb2	1.529847111	6.61E-06
ENSMUSG00000092212	Slc22a13b	1.527964346	9.65E-151
ENSMUSG00000024245	Tmem178	1.489676606	0.031417028
ENSMUSG00000047443	Erfe	1.489371678	0.018426056
ENSMUSG00000027983	Cyp2u1	1.487147631	2.74E-12
ENSMUSG00000022545	Ercc4	1.482905201	0.002972817
ENSMUSG00000078616	Trim30c	1.482239259	0.001765034
ENSMUSG00000061887	Ssbp3	1.478936053	1.23E-10
ENSMUSG00000022799	Arhgap31	1.476568273	0.017330066
ENSMUSG00000021609	Slc6a3	1.475132408	6.02E-20
ENSMUSG00000034371	Tkfc	1.471528231	1.29E-93
ENSMUSG00000038175	Mylip	1.465652357	8.42E-17
ENSMUSG00000042978	Sbk1	1.46125559	0.002229498
ENSMUSG00000096039	D830030K20	1.458139323	8.30E-11
ENSMUSG00000098037	Gm7765	1.457269684	0.039551596
ENSMUSG00000085696	Hoxaas3	1.442762185	0.032407168
ENSMUSG00000074489	Bglap3	1.436640612	0.006665393
ENSMUSG00000027790	Sis	1.430189609	1.13E-71
ENSMUSG00000028385	Snx30	1.42666118	0.001282204
ENSMUSG00000059343	Aldoart1	1.42453907	0.043204363
ENSMUSG00000036622	Atp13a2	1.414649862	7.53E-05
ENSMUSG00000044367	Slc16a13	1.414428575	9.38E-05
ENSMUSG00000040213	Kyat3	1.412789434	0.047208109
ENSMUSG00000038702	Dsel	1.40798657	8.25E-14

ENSMUSG00000000409	Lck	-1.906068651	0.004336286
ENSMUSG00000027562	Car2	-1.901386335	4.49E-52
ENSMUSG00000030428	Ttyh1	-1.893564808	0.010275434
ENSMUSG00000096727	Psmb9	-1.891960068	2.01E-45
ENSMUSG00000020717	Pecam1	-1.89183629	0.015247343
ENSMUSG00000024371	C2	-1.879742587	1.58E-25
ENSMUSG00000027375	Mal	-1.874821646	0.043828705
ENSMUSG00000037321	Tap1	-1.867661309	8.07E-13
ENSMUSG00000029762	Akr1b8	-1.85904752	4.72E-12
ENSMUSG00000078771	Evi2a	-1.838435275	0.00296642
ENSMUSG00000040329	Il7	-1.831385476	0.002602967
ENSMUSG00000064899	Snord118	-1.821212588	0.001632331
ENSMUSG00000057058	Skap1	-1.813141859	0.026318125
ENSMUSG00000002324	Rec8	-1.811173969	5.91E-06
ENSMUSG00000049608	Gpr55	-1.791118376	0.006189469
ENSMUSG00000037145	Lypd8l	-1.790520591	2.31E-83
ENSMUSG00000021509	Slc25a48	-1.774020767	1.29E-19
ENSMUSG00000026358	Rgs1	-1.769312507	0.036349476
ENSMUSG00000070031	Sp140	-1.728138832	2.20E-06
ENSMUSG00000036459	Wtip	-1.716926268	4.33E-11
ENSMUSG00000028689	Ccdc163	-1.709170602	0.042917017
ENSMUSG00000016206	H2-M3	-1.705970883	0.029616272
ENSMUSG00000041202	Pla2g2d	-1.701976759	0.020590466
ENSMUSG00000021108	Prkch	-1.700642415	0.021160302
ENSMUSG00000070034	Sp110	-1.683957815	5.45E-05
ENSMUSG00000069874	Irgm2	-1.683425129	2.24E-12
ENSMUSG00000100593	1700119H24	-1.656944797	0.044566267
ENSMUSG00000031165	Was	-1.638981874	0.037045175
ENSMUSG00000040747	Cd53	-1.636733144	0.016556244
ENSMUSG00000072620	Slfn2	-1.633352995	3.53E-19
ENSMUSG00000068452	Duox2	-1.629601136	2.00E-119
ENSMUSG00000093661	Eif4e3	-1.612954969	7.26E-07
ENSMUSG00000039158	Akna	-1.602819847	0.009861212
ENSMUSG00000010064	Slc38a3	-1.592019033	7.32E-05
ENSMUSG00000052229	Gpr17	-1.588246586	0.008842857
ENSMUSG00000074489	Bglap3	-1.575343647	0.006665393
ENSMUSG00000073197	5730507C01	-1.574361395	1.08E-15
ENSMUSG00000076757	Trgc4	-1.567164747	0.000858235
ENSMUSG00000005947	Itgae	-1.566113015	0.013862877
ENSMUSG00000050914	Ankrd37	-1.565211856	1.49E-05
ENSMUSG00000050762	Prss27	-1.560737199	0.009133556
ENSMUSG00000029603	Dtx1	-1.55925497	0.036163918
ENSMUSG00000021403	Serpinb9b	-1.556171713	8.81E-11
ENSMUSG00000046841	Ckap4	-1.552650048	2.92E-37
ENSMUSG00000031551	Ido1	-1.549087752	7.30E-06
ENSMUSG00000076749	Trgc1	-1.541868602	0.000104307
ENSMUSG00000032033	Barx2	-1.537300474	2.42E-16
ENSMUSG00000088990	Gm22767	-1.532891076	0.002237261
ENSMUSG00000098112	Bin2	-1.530471318	0.0454463
ENSMUSG00000038058	Nod1	-1.527021497	0.031835911
ENSMUSG00000065987	Cd209b	-1.526936473	8.15E-06
ENSMUSG00000025997	Ikzf2	-1.522868104	0.026991512
ENSMUSG00000035356	Nfkbiz	-1.521862269	9.01E-14
ENSMUSG00000047517	Dmbt1	-1.511246968	1.62E-10
ENSMUSG00000026404	Ddx59	-1.501065823	0.024474389
ENSMUSG00000052131	Akr1b7	-1.49298929	6.77E-23
ENSMUSG00000068735	Trp53i11	-1.483953986	1.03E-07
ENSMUSG00000085873	Ttc39aos1	-1.480645283	0.006819693
ENSMUSG00000030707	Coro1a	-1.469633466	5.38E-05
ENSMUSG00000000531	Tamalin	-1.461885865	0.020698169
ENSMUSG00000000682	Cd52	-1.461679743	5.51E-05
ENSMUSG00000074715	Ccl28	-1.459562289	8.97E-12
ENSMUSG00000027995	Tlr2	-1.458836485	0.009715968
ENSMUSG00000120485	Gm35340	-1.45864231	2.97E-06
ENSMUSG00000032109	Nlrx1	-1.451540943	1.68E-07
ENSMUSG00000026525	Opn3	-1.451512389	9.93E-05
ENSMUSG00000064262	Gimap8	-1.449311633	0.009218264
ENSMUSG00000057948	Unc13d	-1.4459442	0.016400404
ENSMUSG00000078485	Plekhn1	-1.445442688	0.00198114
ENSMUSG00000096965	3300005D01	-1.428039631	4.47E-10
ENSMUSG00000094662	Defa36	-1.419911981	0.023237477
ENSMUSG00000092517	Art2a	-1.417074516	0.000299797
ENSMUSG00000068855	H2ac20	-1.41583486	0.038391136
ENSMUSG00000053054	Adh6a	-1.414265841	4.87E-60
ENSMUSG00000029084	Cd38	-1.407018047	5.78E-18
ENSMUSG00000040751	Lat2	-1.40696369	9.51E-05
ENSMUSG00000030220	Arhgdib	-1.39399413	0.006445494
ENSMUSG00000048163	Selplg	-1.383633004	0.020438256
ENSMUSG00000030165	Klrd1	-1.381312675	0.016054813
ENSMUSG00000039146	Ifi44l	-1.370904342	0.032942047
ENSMUSG00000085995	Gm2788	-1.357136003	4.30E-08
ENSMUSG00000042808	Gpx2	-1.353985043	2.68E-67
ENSMUSG00000031304	Il2rg	-1.348127048	0.014137151
ENSMUSG00000026509	Capn2	-1.345407384	0.026162214
ENSMUSG00000004038	Gstm3	-1.334810421	1.44E-59
ENSMUSG00000042677	Zc3h12a	-1.33261726	3.44E-17
ENSMUSG00000030263	Irag2	-1.329714585	0.003545798
ENSMUSG00000079436	Kcnj13	-1.295481589	6.61E-20
ENSMUSG00000052373	Mpp3	-1.291674432	0.001803626
ENSMUSG00000014846	Tppp3	-1.288322659	2.94E-06
ENSMUSG00000021187	Tc2n	-1.279375694	0.001350529
ENSMUSG00000038530	Rgs4	-1.27831209	0.02836377
ENSMUSG00000021749	Fam3d	-1.27732963	1.28E-33
ENSMUSG00000028480	Glipr2	-1.27523909	5.72E-21
ENSMUSG00000029923	Rab19	-1.261939194	0.00072332
ENSMUSG00000040010	Slc7a5	-1.261192521	0.029843409
ENSMUSG00000036943	Rab8b	-1.256328691	0.003773892
ENSMUSG00000038301	Snx10	-1.24010665	5.47E-09
ENSMUSG00000079445	B3gnt7	-1.229399844	0.000414182
ENSMUSG00000004665	Cnn2	-1.224680547	0.000215042
ENSMUSG00000028019	Pdgfc	-1.210405973	0.01248121
ENSMUSG00000023073	Slc10a2	-1.209260522	2.45E-39
ENSMUSG00000022226	Mcpt2	-1.208961193	0.001094271
ENSMUSG00000013846	St3gal1	-1.206585475	6.71E-05
ENSMUSG00000063851	Rnf183	-1.202543794	0.00518228
ENSMUSG00000020875	Hoxb9	-1.200318207	0.001637345
ENSMUSG00000002384	Bmp8b	-1.195577958	0.002773712
ENSMUSG00000058952	Cfi	-1.194226912	1.85E-05
ENSMUSG00000042115	Klhdc8a	-1.194192325	0.01271405
ENSMUSG00000016028	Celsr1	-1.189677569	0.007264573
ENSMUSG00000047945	Marcksl1	-1.183943765	1.34E-25
ENSMUSG00000090101	Snhg9	-1.183646211	1.08E-05
ENSMUSG00000029752	Asns	-1.181334958	2.40E-13
ENSMUSG00000009378	Slc16a12	-1.180980676	0.046206365
ENSMUSG00000044303	Cdkn2a	-1.179878114	0.015115355
ENSMUSG00000020865	Abcc3	-1.176694481	0.000579926
ENSMUSG00000021200	Asb2	-1.175894867	0.001512495
ENSMUSG00000064109	Hcst	-1.166430178	7.00E-10
ENSMUSG00000009585	Apobec3	-1.166338911	0.000667899
ENSMUSG00000072618	Gm10384	-1.166179924	0.00401388
ENSMUSG00000054582	Pabpc1l	-1.154663612	0.026874588
ENSMUSG00000091625	Lsm5	-1.153487236	0.006975797

ENSMUSG00000037211	Spry1	1.758344012	0.015209092
ENSMUSG00000040189	Odad1	1.752479374	1.86E-20
ENSMUSG00000020377	Ltc4s	1.752399276	9.63E-11
ENSMUSG00000026070	Il18r1	1.749779909	0.044795055
ENSMUSG00000022180	Slc7a8	1.744272546	1.64E-08
ENSMUSG00000029814	Igf2bp3	1.731976164	5.40E-19
ENSMUSG00000026950	Neb	1.720708946	0.004017009
ENSMUSG00000039114	Nrn1	1.71951414	0.043204363
ENSMUSG00000052914	Cyp2j6	1.711414303	6.68E-49
ENSMUSG00000003283	Hck	1.708015038	0.000113552
ENSMUSG00000023828	Slc22a3	1.701681559	2.67E-09
ENSMUSG00000059631	1500035N22	1.69882277	8.72E-08
ENSMUSG00000073094	Smim9	1.691268896	0.000369346
ENSMUSG00000039110	Mycbpap	1.689708451	0.000395588
ENSMUSG00000028356	Ambp	1.689144983	9.96E-11
ENSMUSG00000020467	Efemp1	1.68673012	0.023672623
ENSMUSG00000026621	Mtarc1	1.686366959	2.87E-06
ENSMUSG00000042116	Vwa1	1.660068952	2.58E-06
ENSMUSG00000093805	Gal3st2b	1.659144103	9.33E-07
ENSMUSG00000027698	Nceh1	1.647598905	4.72E-10
ENSMUSG00000031626	Sorbs2	1.647163441	0.000881561
ENSMUSG00000029311	Hsd17b11	1.646138563	2.89E-41
ENSMUSG00000015243	Abca1	1.644279442	8.63E-48
ENSMUSG00000049555	Tmie	1.639758253	1.20E-10
ENSMUSG00000062410	Hsd3b3	1.638665173	2.51E-09
ENSMUSG00000030155	Clec2e	1.637159413	1.06E-30
ENSMUSG00000025810	Nrp1	1.6291052	0.002285352
ENSMUSG00000024266	Adad2	1.621542361	2.46E-05
ENSMUSG00000032418	Me1	1.617579929	3.60E-20
ENSMUSG00000025243	Slc6a20b	1.609655985	8.21E-05
ENSMUSG00000112129	Pbld1	1.590694756	1.38E-37
ENSMUSG00000024620	Pdgfrb	1.587754121	0.002497937
ENSMUSG00000034528	Hsd17b13	1.584096827	5.76E-28
ENSMUSG00000087006	Gm13889	1.580672373	0.028146604
ENSMUSG00000028713	Cyp4b1	1.576789588	4.44E-42
ENSMUSG00000018752	Tnfsfm13	1.568047466	0.009471519
ENSMUSG00000047250	Ptgs1	1.5566063	0.003457643
ENSMUSG00000040998	Npnt	1.545082873	3.94E-05
ENSMUSG00000039410	Prdm16	1.542526007	2.94E-19
ENSMUSG00000097743	Gm16973	1.534202296	0.043818152
ENSMUSG00000078780	Gm5150	1.533627406	0.004342645
ENSMUSG00000035112	Wnk4	1.515613138	0.000194793
ENSMUSG00000104529	Rbakdn	1.511548443	0.0001419
ENSMUSG00000022037	Clu	1.511021244	0.00321906
ENSMUSG00000033174	Mgll	1.508467311	6.73E-09
ENSMUSG00000022912	Pros1	1.507443931	0.003138142
ENSMUSG00000032085	Tagln	1.507437239	0.002847561
ENSMUSG00000032122	Slc37a2	1.504566244	0.003269137
ENSMUSG00000022687	Boc	1.503626942	0.040791125
ENSMUSG00000022445	Cyp2d26	1.495724223	1.04E-24
ENSMUSG00000006711	D130043K22	1.481979054	2.35E-05
ENSMUSG00000046658	Zfp316	1.474313459	0.032543912
ENSMUSG00000055827	Gsdmc3	1.47412598	5.06E-13
ENSMUSG00000016382	Pls3	1.463004114	0.014497077
ENSMUSG00000027359	Slc27a2	1.461979133	1.01E-46
ENSMUSG00000064202	Spata6l	1.459774569	1.73E-18
ENSMUSG00000026726	Cubn	1.452676785	1.07E-31
ENSMUSG00000026678	Rgs5	1.451679781	0.011034641
ENSMUSG00000061878	Sphk1	1.444623639	7.83E-11
ENSMUSG00000037440	Vnn1	1.442488589	4.37E-37
ENSMUSG00000006369	Fbln1	1.438853759	2.37E-17
ENSMUSG00000005514	Por	1.436377889	3.99E-13
ENSMUSG00000018648	Dusp14	1.433446283	0.000184746
ENSMUSG00000109841	E330011O21	1.432170325	0.00365429
ENSMUSG00000053279	Aldh1a1	1.4293652	3.19E-45
ENSMUSG00000004035	Gstm7	1.428816057	7.67E-36
ENSMUSG00000045980	Tmem104	1.425020021	0.030993454
ENSMUSG00000050721	Plekho2	1.42311011	0.00839658
ENSMUSG00000067231	Cyp2c65	1.417759778	1.76E-51
ENSMUSG00000022383	Ppara	1.410390126	1.23E-34
ENSMUSG00000046908	Ltb4r1	1.406959082	0.014592683
ENSMUSG00000032715	Trib3	1.402119227	1.52E-08
ENSMUSG00000038648	Creb3l2	1.382522813	2.44E-05
ENSMUSG00000006586	Runx1t1	1.376720195	0.007757073
ENSMUSG00000019762	Iyd	1.373102947	6.05E-35
ENSMUSG00000094651	Gal3st2	1.369040451	5.75E-20
ENSMUSG00000030554	Synm	1.368283883	0.015891634
ENSMUSG00000028427	Aqp7	1.364201482	2.25E-06
ENSMUSG00000064210	Ano6	1.362062246	7.45E-15
ENSMUSG00000116673	A630089N07	1.348887286	0.012945209
ENSMUSG00000033910	Gucy1a1	1.347151554	0.041924022
ENSMUSG00000105906	Iglc1	1.337737868	8.71E-14
ENSMUSG00000022040	Ephx2	1.33539391	3.62E-85
ENSMUSG00000019775	Rgs17	1.335139057	0.000657803
ENSMUSG00000037348	Paqr7	1.334864942	1.27E-18
ENSMUSG00000026478	Lamc1	1.329658427	0.048902709
ENSMUSG00000023094	Msrb2	1.325174403	6.61E-06
ENSMUSG00000037379	Spon2	1.319991813	0.019851384
ENSMUSG00000040147	Maob	1.319676303	1.42E-08
ENSMUSG00000079057	Cyp4v3	1.319296229	2.77E-11
ENSMUSG00000014351	Gip	1.312098396	3.09E-66
ENSMUSG00000057440	Mpp7	1.308083067	6.17E-06
ENSMUSG00000029134	Plb1	1.302227355	1.24E-244
ENSMUSG00000035448	Ccr3	1.296781391	0.003140787
ENSMUSG00000092212	Slc22a13b	1.295879029	9.65E-151
ENSMUSG00000056698	Elmod3	1.2929131	3.20E-05
ENSMUSG00000015312	Gadd45b	1.289772072	0.007364716
ENSMUSG00000030087	Klf15	1.286502556	0.010434514
ENSMUSG00000025652	Tmem89	1.276460098	0.000104076
ENSMUSG00000039976	Tbc1d16	1.274709181	0.024492178
ENSMUSG00000052584	Serp2	1.27405208	0.049068391
ENSMUSG00000058317	Ube2e2	1.273895238	0.020360003
ENSMUSG00000030762	Aqp8	1.271917628	1.56E-122
ENSMUSG00000031444	F10	1.27191322	1.98E-09
ENSMUSG00000020333	Acsl6	1.271913113	3.63E-13
ENSMUSG00000108449	Gm44507	1.271912488	6.79E-06
ENSMUSG00000052631	Sh2d6	1.271912019	0.026207129
ENSMUSG00000066682	Pilrb2	1.27191109	0.002476358
ENSMUSG00000040111	Gramd1b	1.269737761	2.02E-12
ENSMUSG00000028199	Cryz	1.269560318	2.29E-05
ENSMUSG00000036186	Dipk1b	1.269320175	0.000103815
ENSMUSG00000027792	Bche	1.258995701	7.31E-60
ENSMUSG00000020604	Arsg	1.25104979	1.94E-71
ENSMUSG00000022853	Ehhadh	1.247882923	0.023344525
ENSMUSG00000025004	Cyp2c40	1.246596013	0.04426896
ENSMUSG00000086728	Man2c1os	1.244732271	0.038066421
ENSMUSG00000024507	Hsd17b4	1.244385976	2.08E-23
ENSMUSG00000035780	Ugt2a3	1.243424808	1.57E-32
ENSMUSG00000015134	Aldh1a3	1.242310631	2.74E-12
ENSMUSG00000068762	Gstm6	1.240997355	2.20E-123
ENSMUSG00000022263	Trio	1.237600905	9.32E-19
ENSMUSG00000091721	Gimd1	1.232654046	2.23E-09

ENSMUSG00000027684	Mecom	-1.605420726	3.35E-07
ENSMUSG00000015437	Gzmb	-1.600035692	4.06E-31
ENSMUSG00000025934	Gsta3	-1.572704453	1.34E-08
ENSMUSG00000020062	Slc5a8	-1.56883398	1.21E-194
ENSMUSG00000021733	Slc4a7	-1.557011316	2.48E-16
ENSMUSG00000009246	Trpm5	-1.555667206	6.91E-05
ENSMUSG00000003134	Tbc1d8	-1.537108374	0.005972119
ENSMUSG00000079071	Slc28a2b	-1.535112184	1.13E-13
ENSMUSG00000097852	4933405D12	-1.527564726	0.014260624
ENSMUSG00000039005	Tlr4	-1.520373381	0.018279328
ENSMUSG00000030402	Ppm1n	-1.514164186	5.34E-07
ENSMUSG00000041974	Spidr	-1.511648993	0.028064487
ENSMUSG00000064442	Gm26225	-1.508254119	0.029363438
ENSMUSG00000034871	Fam151a	-1.503403018	2.68E-104
ENSMUSG00000041000	Trim62	-1.496876803	0.000799537
ENSMUSG00000022346	Myc	-1.488192583	0.000512097
ENSMUSG00000014846	Tppp3	-1.4877588	2.94E-06
ENSMUSG00000051748	Wfdc21	-1.484613923	0.000299811
ENSMUSG00000041120	Nbl1	-1.484136218	2.03E-79
ENSMUSG00000039653	Baat	-1.481517273	0.010414355
ENSMUSG00000035042	Ccl5	-1.479473113	3.73E-63
ENSMUSG00000028713	Cyp4b1	-1.473937984	4.44E-42
ENSMUSG00000017697	Ada	-1.451048908	1.40E-57
ENSMUSG00000044303	Cdkn2a	-1.44939479	0.015115355
ENSMUSG00000053004	Hrh1	-1.449328972	0.001080766
ENSMUSG00000050520	Cldn8	-1.448927136	2.37E-07
ENSMUSG00000047844	Bex4	-1.448136189	2.25E-26
ENSMUSG00000061947	Serpina10	-1.447478504	2.84E-34
ENSMUSG00000051435	Fhad1	-1.445906876	0.020906802
ENSMUSG00000039781	Cep131	-1.445906324	0.032353312
ENSMUSG00000073468	Sft2d1	-1.445692767	0.00817157
ENSMUSG00000035878	Hykk	-1.444867624	0.000683235
ENSMUSG00000069324	Bhmt1b	-1.444693262	0.020752792
ENSMUSG00000100150	Gm19585	-1.439908264	0.000369602
ENSMUSG00000062082	Cd200r4	-1.437913754	0.000834551
ENSMUSG00000058952	Cfi	-1.436356233	1.85E-05
ENSMUSG00000068115	Ninl	-1.411691969	0.013006961
ENSMUSG00000056737	Capg	-1.405848244	4.76E-05
ENSMUSG00000027210	Meis2	-1.402731955	1.23E-09
ENSMUSG00000002319	Ipo4	-1.402481082	0.001672171
ENSMUSG00000055730	Ces2a	-1.390746205	7.68E-45
ENSMUSG00000031304	Il2rg	-1.390425957	0.014137151
ENSMUSG00000042808	Gpx2	-1.382348043	2.68E-67
ENSMUSG00000068452	Duox2	-1.371448166	2.00E-119
ENSMUSG00000018900	Slc22a5	-1.369577285	6.34E-17
ENSMUSG00000038732	Mboat1	-1.367020692	4.81E-54
ENSMUSG00000006342	Susd2	-1.362256738	0.000299811
ENSMUSG00000027636	Sla2	-1.337338079	0.043432933
ENSMUSG00000073421	H2-Ab1	-1.335981492	4.80E-130
ENSMUSG00000038496	Slc19a3	-1.334083615	1.53E-10
ENSMUSG00000029377	Ereg	-1.322301825	8.57E-16
ENSMUSG00000031725	Ces1f	-1.321679915	2.19E-36
ENSMUSG00000078938	Syce3	-1.317790073	1.71E-05
ENSMUSG00000021749	Fam3d	-1.310944644	1.28E-33
ENSMUSG00000037936	Scarb1	-1.303865469	1.33E-32
ENSMUSG00000099583	H3c4	-1.295477384	0.027308807
ENSMUSG00000064262	Gimap8	-1.2951483	0.009218264
ENSMUSG00000026117	Zap70	-1.294364139	0.016319231
ENSMUSG00000044676	Zfp612	-1.287675461	0.046132894
ENSMUSG00000047766	Lrrc49	-1.274100515	0.00249024
ENSMUSG00000004655	Aqp1	-1.274062932	8.85E-13
ENSMUSG00000027890	Gstm4	-1.270661744	2.83E-30
ENSMUSG00000044986	Tst	-1.267531352	0.000941387
ENSMUSG00000030725	Lipt2	-1.267041506	0.025601017
ENSMUSG00000074183	Gsta1	-1.264720059	1.37E-32
ENSMUSG00000031360	Ctps2	-1.251784526	0.021723876
ENSMUSG00000022615	Tymp	-1.249026829	1.04E-08
ENSMUSG00000046697	Enpp7	-1.245619757	1.39E-24
ENSMUSG00000028427	Aqp7	-1.243412518	2.25E-06
ENSMUSG00000064202	Spata6l	-1.241553408	1.73E-18
ENSMUSG00000054999	Naaladl1	-1.241498718	7.28E-99
ENSMUSG00000054321	Taf4b	-1.234990556	0.001361863
ENSMUSG00000054630	Ugt2b5	-1.229713475	2.79E-50
ENSMUSG00000039943	Plcb4	-1.223635962	0.04824816
ENSMUSG00000029644	Pdx1	-1.221441606	7.46E-26
ENSMUSG00000005672	Kit	-1.218790319	5.71E-09
ENSMUSG00000076749	Trgc1	-1.203532344	0.000104307
ENSMUSG00000027562	Car2	-1.185198338	4.49E-52
ENSMUSG00000048779	P2ry6	-1.18275472	0.000677353
ENSMUSG00000089614	Gm23505	-1.179699988	0.003602444
ENSMUSG00000064581	Gm25133	-1.173063351	4.02E-09
ENSMUSG00000005980	Dnase1	-1.17098317	3.55E-08
ENSMUSG00000031377	Bmx	-1.170192942	0.002159169
ENSMUSG00000021751	Acox2	-1.169035047	0.01180493
ENSMUSG00000042500	Ago4	-1.164997734	0.014262929
ENSMUSG00000073197	5730507C01	-1.164130051	1.08E-15
ENSMUSG00000040562	Gstm2	-1.161889913	1.03E-80
ENSMUSG00000037348	Paqr7	-1.161410652	1.27E-18
ENSMUSG00000059654	Reg1	-1.160727837	8.56E-92
ENSMUSG00000029311	Hsd17b11	-1.159378098	2.89E-41
ENSMUSG00000048440	Cyp4f16	-1.157119442	1.45E-11
ENSMUSG00000061825	Ces2c	-1.154032739	3.66E-24
ENSMUSG00000072949	Acot1	-1.150162779	1.10E-20
ENSMUSG00000043931	Gimap7	-1.13624975	5.37E-10
ENSMUSG00000030528	Blm	-1.13276103	0.014592683
ENSMUSG00000031101	Sash3	-1.128805027	0.030229682
ENSMUSG00000108449	Gm44507	-1.122708672	6.79E-06
ENSMUSG00000027661	Slc2a10	-1.12226519	0.009381024
ENSMUSG00000090115	Usp49	-1.114372674	0.004258887
ENSMUSG00000072618	Gm10384	-1.111575676	0.00401388
ENSMUSG00000076498	Trbc2	-1.111307397	4.95E-14
ENSMUSG00000035900	Gramd4	-1.110099134	0.007532154
ENSMUSG00000067006	Serpinb5	-1.106458663	0.010991827
ENSMUSG00000056035	Cyp3a11	-1.105984125	1.76E-88
ENSMUSG00000024640	Psat1	-1.105086686	0.000837522
ENSMUSG00000021620	Acot12	-1.098205401	3.88E-06
ENSMUSG00000022687	Boc	-1.096553933	0.040791125
ENSMUSG00000120645	Gm29688	-1.096501296	1.14E-08
ENSMUSG00000060240	Cend1	-1.087553244	0.044916321
ENSMUSG00000032033	Barx2	-1.08702627	2.42E-16
ENSMUSG00000041577	Prelp	-1.08087207	0.034196222
ENSMUSG00000033767	Tmem131l	-1.079953015	0.009364853
ENSMUSG00000067231	Cyp2c65	-1.078732502	1.76E-51
ENSMUSG00000022070	Bora	-1.077928422	3.60E-05
ENSMUSG00000026358	Rgs1	-1.077625792	0.036349476
ENSMUSG00000039745	Htatip2	-1.074902076	2.23E-15
ENSMUSG00000006313	Upk1a	-1.074657546	0.000195332
ENSMUSG00000026639	Lamb3	-1.074245205	0.000145891
ENSMUSG00000039110	Mycbpap	-1.07369817	0.000395588
ENSMUSG00000005947	Itgae	-1.069114125	0.013862877
ENSMUSG00000036594	H2-Aa	-1.068247386	2.67E-155
ENSMUSG00000028024	Enpep	-1.066768371	1.52E-36

ENSMUSG00000038473	Nos1ap	1.405857548	5.82E-08
ENSMUSG00000071604	Entrep1	1.405005426	0.014991104
ENSMUSG00000029061	Mmp23	1.40491491	6.05E-07
ENSMUSG00000051727	Kctd14	1.401409474	1.04E-23
ENSMUSG00000062044	Lmtk3	1.397563395	0.032832312
ENSMUSG00000047264	Zfp358	1.389474415	0.003174179
ENSMUSG00000070808	Bicra	1.369982985	9.18E-05
ENSMUSG00000028545	Bend5	1.369869639	8.02E-08
ENSMUSG00000028268	Gbp3	1.364245848	4.13E-06
ENSMUSG00000112805	Gm34574	1.36357506	0.001271606
ENSMUSG00000063364	3300002I08R	1.351671985	0.026788152
ENSMUSG00000045932	Ifit2	1.347012195	5.81E-05
ENSMUSG00000018547	Pip4k2b	1.344217921	0.002074464
ENSMUSG00000005397	Nid1	1.34403264	0.014772576
ENSMUSG00000040913	Fbxw4	1.339088793	0.015824669
ENSMUSG00000032109	Nlrx1	1.335482229	1.68E-07
ENSMUSG00000032076	Cadm1	1.32817826	0.014054383
ENSMUSG00000105906	Iglc1	1.319325415	8.71E-14
ENSMUSG00000032085	Tagln	1.319094007	0.002847561
ENSMUSG00000038604	Ripor1	1.31749118	0.036251442
ENSMUSG00000043496	Tril	1.31422481	0.016653232
ENSMUSG00000040998	Npnt	1.314020491	3.94E-05
ENSMUSG00000026659	Dusp12	1.306818824	2.92E-09
ENSMUSG00000025316	Banp	1.305668593	0.000291222
ENSMUSG00000038058	Nod1	1.305265394	0.031835911
ENSMUSG00000024682	Cblif	1.280250508	1.35E-06
ENSMUSG00000076934	Iglv1	1.277874465	0.001877963
ENSMUSG00000021765	Fst	1.273433819	0.003693273
ENSMUSG00000041828	Abca8a	1.268194738	0.038369936
ENSMUSG00000037989	Wnk2	1.267662841	0.000355004
ENSMUSG00000086382	Chrna1os	1.265524034	0.000741664
ENSMUSG00000020553	Pctp	1.261429195	0.030315921
ENSMUSG00000091649	Phf11b	1.259479711	0.001025936
ENSMUSG00000089774	Slc5a3	1.259303797	0.000248451
ENSMUSG00000027395	Polr1b	1.25737242	0.017297188
ENSMUSG00000039976	Tbc1d16	1.253463614	0.024492178
ENSMUSG00000037211	Spry1	1.248974472	0.015209092
ENSMUSG00000062515	Fabp4	1.243550029	1.34E-07
ENSMUSG00000033910	Gucy1a1	1.241840934	0.041924022
ENSMUSG00000037887	Dusp8	1.24090074	0.041924022
ENSMUSG00000059149	Mfsd4a	1.238033514	1.66E-08
ENSMUSG00000021962	Dcp1a	1.233896055	1.49E-05
ENSMUSG00000038506	Dcun1d2	1.230414311	0.016392718
ENSMUSG00000050395	Tnfsf15	1.22400172	1.94E-05
ENSMUSG00000003518	Dusp3	1.216191089	0.011052933
ENSMUSG00000023905	Tnfrsf12a	1.215833232	4.29E-05
ENSMUSG00000036896	C1qc	1.205754529	0.009140038
ENSMUSG00000030407	Qpctl	1.200206867	0.022839999
ENSMUSG00000039114	Nrn1	1.199979231	0.043204363
ENSMUSG00000010660	Plcd1	1.199288251	5.40E-08
ENSMUSG00000048264	Dip2c	1.195789498	0.008590019
ENSMUSG00000024620	Pdgfrb	1.191218102	0.002497937
ENSMUSG00000032915	Adgre4	1.188699182	0.016489501
ENSMUSG00000054404	Slfn5	1.188129034	0.003397837
ENSMUSG00000027956	Tmem144	1.186909579	1.47E-10
ENSMUSG00000087107	AI662270	1.184587094	0.049609771
ENSMUSG00000066571	Garre1	1.18248499	8.11E-05
ENSMUSG00000026069	Il1rl1	1.178644921	0.017557016
ENSMUSG00000062373	Tmem65	1.175773388	8.02E-08
ENSMUSG00000058230	Arhgap35	1.175282035	6.39E-05
ENSMUSG00000031548	Sfrp1	1.174239656	0.006683715
ENSMUSG00000097908	4933404O12	1.159451424	0.000358273
ENSMUSG00000035769	Xylb	1.158151803	0.000151948
ENSMUSG00000030787	Lyve1	1.153330982	0.020104265
ENSMUSG00000045294	Insig1	1.151231858	1.10E-14
ENSMUSG00000087477	Gm13822	1.148780031	0.005636551
ENSMUSG00000087433	Gm14167	1.147691865	0.015674104
ENSMUSG00000027889	Ampd2	1.145909342	0.008663014
ENSMUSG00000076613	Ighg2b	1.141935368	0.010450098
ENSMUSG00000054417	Cyp3a44	1.135218037	1.93E-09
ENSMUSG00000030107	Usp18	1.134908066	1.66E-21
ENSMUSG00000025867	Cplx2	1.13085743	0.048084422
ENSMUSG00000091811	Inafm1	1.119351759	0.000500059
ENSMUSG00000031762	Mt2	1.118107003	0.000905458
ENSMUSG00000039840	Epg5	1.118080527	0.021917203
ENSMUSG00000030051	Aplf	1.117981879	0.034913088
ENSMUSG00000023349	Clec4n	1.115512472	0.001097884
ENSMUSG00000086728	Man2c1os	1.114140834	0.038066421
ENSMUSG00000023961	Enpp4	1.10975006	0.0456917
ENSMUSG00000038831	Ralgps1	1.109138276	1.14E-09
ENSMUSG00000049690	Nckap5	1.106552268	1.20E-06
ENSMUSG00000020661	Dnmt3a	1.101487538	1.32E-06
ENSMUSG00000078650	G6pc	1.09143295	1.24E-83
ENSMUSG00000026678	Rgs5	1.088194383	0.011034641
ENSMUSG00000070327	Rnf213	1.087932958	0.001029581
ENSMUSG00000027690	Slc2a2	1.087474675	1.39E-150
ENSMUSG00000034459	Ifit1	1.072036631	7.04E-10
ENSMUSG00000019890	Nts	1.067588701	2.37E-49
ENSMUSG00000078949	R3hdml	1.066499605	0.039640743
ENSMUSG00000100826	Ipw	1.060696215	1.93E-08
ENSMUSG00000006386	Tek	1.059457274	1.07E-05
ENSMUSG00000050071	Bex1	1.055336082	1.78E-33
ENSMUSG00000002781	Tmem143	1.054295017	4.62E-06
ENSMUSG00000032769	Trpa1	1.052675563	1.73E-05
ENSMUSG00000001870	Ltbp1	1.04443298	0.000129694
ENSMUSG00000097145	9230114K14	1.041927294	0.015298951
ENSMUSG00000002250	Ppard	1.036598107	0.001191729
ENSMUSG00000036459	Wtip	1.034079425	4.33E-11
ENSMUSG00000042505	Sdhaf3	1.032296948	7.05E-08
ENSMUSG00000042684	Npl	1.030356743	0.001788343
ENSMUSG00000038059	Smim3	1.028817097	0.019239983
ENSMUSG00000046841	Ckap4	1.027819152	2.92E-37
ENSMUSG00000070574	2310016G11	1.026244544	0.028623231
ENSMUSG00000034413	Neurl1b	1.024756579	4.99E-05
ENSMUSG00000021974	Fgf9	1.022683032	0.000578364
ENSMUSG00000021140	Pcnx	1.020639091	0.007292993
ENSMUSG00000068587	Mgam	1.015688146	1.43E-05
ENSMUSG00000047921	Trappc9	1.014773098	0.036136295
ENSMUSG00000014859	E2f4	1.014607037	0.000728529
ENSMUSG00000079055	Slc8a3	1.010107877	0.018081601
ENSMUSG00000013653	1810065E05	1.008993875	1.70E-70
ENSMUSG00000026670	Uap1	1.007478253	2.54E-07
ENSMUSG00000034981	Parm1	1.007266134	1.69E-05
ENSMUSG00000031706	Rfx1	1.005577364	7.50E-07
ENSMUSG00000038530	Rgs4	1.003495601	0.02836377
ENSMUSG00000056531	Ccdc18	1.002705747	0.004966899
ENSMUSG00000097743	Gm16973	1.002616104	0.043818152
ENSMUSG00000020644	Id2	1.00224581	4.95E-24
ENSMUSG00000017737	Mmp9	1.002053026	0.016319231
ENSMUSG00000014907	Naf1	1.00054015	0.037447539
ENSMUSG00000021403	Serpinb9b	0.999112773	8.81E-11
ENSMUSG00000055320	Tead1	0.997150057	5.65E-10

ENSMUSG00000031584	Gsr	-1.149405782	4.50E-96
ENSMUSG00000033906	Zdhhc15	-1.146552755	0.015826695
ENSMUSG00000042745	Id1	-1.14556446	1.86E-18
ENSMUSG00000030107	Usp18	-1.141143083	1.66E-21
ENSMUSG00000074342	I830077J02R	-1.132581003	0.003762332
ENSMUSG00000050747	Trim15	-1.131206657	1.25E-06
ENSMUSG00000065208	Gm24616	-1.129166832	0.00536353
ENSMUSG00000029798	Herc6	-1.124166901	2.02E-09
ENSMUSG00000024124	Prss30	-1.124123171	1.98E-29
ENSMUSG00000075010	AW112010	-1.123287889	3.88E-07
ENSMUSG00000044681	Cnpy1	-1.121256436	3.51E-05
ENSMUSG00000022900	Ildr1	-1.118370306	0.001999876
ENSMUSG00000026586	Prrx1	-1.118188891	7.13E-56
ENSMUSG00000012187	Mogat1	-1.116035702	0.028413825
ENSMUSG00000029847	Slc23a4	-1.11536136	1.72E-07
ENSMUSG00000022748	Cmss1	-1.114389099	0.02142583
ENSMUSG00000021023	Prorp	-1.112332126	0.014144122
ENSMUSG00000030402	Ppm1n	-1.111857379	5.34E-07
ENSMUSG00000092570	Plut	-1.11012588	1.32E-40
ENSMUSG00000023345	Poc1a	-1.108863499	0.009900661
ENSMUSG00000021190	Lgmn	-1.105859706	1.84E-07
ENSMUSG00000085980	Gm12408	-1.105585865	0.006727072
ENSMUSG00000027454	Gins1	-1.100274526	0.015930305
ENSMUSG00000021619	Atg10	-1.099062514	5.79E-05
ENSMUSG00000032420	Nt5e	-1.098566333	2.12E-15
ENSMUSG00000105547	Iglc3	-1.096802227	0.031216573
ENSMUSG00000039470	Zdhhc2	-1.088693692	6.57E-08
ENSMUSG00000102106	2310043O21	-1.087421517	0.011305183
ENSMUSG00000031628	Casp3	-1.086943041	5.48E-104
ENSMUSG00000057346	Apol9a	-1.084041431	3.95E-21
ENSMUSG00000017446	C1qtnf1	-1.083732687	0.016450556
ENSMUSG00000079523	Tmsb10	-1.080971017	2.96E-05
ENSMUSG00000025161	Slc16a3	-1.069613582	9.15E-28
ENSMUSG00000074439	Defa5	-1.066791679	2.55E-09
ENSMUSG00000074004	B3gnt6	-1.061403843	0.00659336
ENSMUSG00000071637	Cebpd	-1.061174491	1.70E-21
ENSMUSG00000033707	Lrrc24	-1.057675233	0.01803907
ENSMUSG00000058163	Gm5431	-1.055952136	0.000455939
ENSMUSG00000073598	Smim33	-1.050607799	0.001412539
ENSMUSG00000022847	Thpo	-1.047931298	0.002805469
ENSMUSG00000033538	Casp4	-1.044997183	0.000130017
ENSMUSG00000037434	Slc30a1	-1.044639417	3.15E-20
ENSMUSG00000063206	Defa34	-1.044465617	2.76E-20
ENSMUSG00000025492	Ifitm3	-1.043052849	7.20E-05
ENSMUSG00000045871	Slitrk6	-1.041265087	4.59E-15
ENSMUSG00000102047	2010010A06	-1.040604835	0.000251934
ENSMUSG00000040253	Gbp7	-1.039795467	2.03E-08
ENSMUSG00000049047	Armcx3	-1.039153104	0.021984813
ENSMUSG00000032035	Ets1	-1.038230496	0.0002307
ENSMUSG00000050335	Lgals3	-1.037950504	2.71E-14
ENSMUSG00000005237	Dnah2	-1.037909335	2.61E-38
ENSMUSG00000027016	Zfp385b	-1.035990509	6.44E-06
ENSMUSG00000049858	Suox	-1.03529128	4.09E-51
ENSMUSG00000018623	Mmp7	-1.027185997	3.64E-10
ENSMUSG00000044827	Tlr1	-1.022674726	0.027753045
ENSMUSG00000048779	P2ry6	-1.020861659	0.000677353
ENSMUSG00000053862	Slc51b	-1.020801921	1.15E-12
ENSMUSG00000029644	Pdx1	-1.019158235	7.46E-26
ENSMUSG00000028937	Acot7	-1.018919753	6.74E-09
ENSMUSG00000075254	Heg1	-1.018894728	0.021349288
ENSMUSG00000063522	Ly6m	-1.018854434	3.14E-20
ENSMUSG00000030309	Caprin2	-1.015667798	0.001512029
ENSMUSG00000060121	Gemin2	-1.015651754	0.005317399
ENSMUSG00000029009	Mthfr	-1.013634274	0.033519392
ENSMUSG00000022227	Mcpt1	-1.007452299	0.009288642
ENSMUSG00000026417	Pigr	-1.006630326	2.07E-14
ENSMUSG00000020839	Tmigd1	-1.006530757	3.38E-68
ENSMUSG00000028699	Tspan1	-1.004870663	9.13E-47
ENSMUSG00000030407	Qpctl	-1.001928401	0.022839999
ENSMUSG00000061845	Defa35	-0.999988554	2.54E-15
ENSMUSG00000024421	Lama3	-0.999974907	1.49E-13
ENSMUSG00000042306	S100a14	-0.993862827	2.73E-07
ENSMUSG00000051735	Rinl	-0.992914175	0.027753045
ENSMUSG00000105879	Gm6204	-0.991596029	0.000809269
ENSMUSG00000026971	Itgb6	-0.991595877	3.61E-06
ENSMUSG00000080727	C920021L13	-0.986576918	0.023818348
ENSMUSG00000037419	Endod1	-0.984103107	1.20E-21
ENSMUSG00000031803	B3gnt3	-0.98348725	8.31E-13
ENSMUSG00000026483	Niban1	-0.981884552	0.012218937
ENSMUSG00000024640	Psat1	-0.979451982	0.000837522
ENSMUSG00000000440	Pparg	-0.975515524	3.31E-14
ENSMUSG00000028885	Smpdl3b	-0.973320949	1.81E-13
ENSMUSG00000027323	Rad51	-0.972105585	0.013561666
ENSMUSG00000049436	Upk1b	-0.968060761	3.28E-12
ENSMUSG00000028194	Ddah1	-0.966323917	3.41E-78
ENSMUSG00000028028	Alpk1	-0.966156608	1.94E-10
ENSMUSG00000036805	Noxa1	-0.965089407	4.43E-07
ENSMUSG00000060149	BC002059	-0.964820754	0.001291087
ENSMUSG00000088254	Gm24289	-0.963508036	0.000105645
ENSMUSG00000026104	Stat1	-0.961088993	2.20E-09
ENSMUSG00000012519	Mlkl	-0.960830026	1.49E-07
ENSMUSG00000007987	Ift22	-0.957063696	0.002178827
ENSMUSG00000039699	Batf2	-0.955783094	2.17E-10
ENSMUSG00000029086	Prom1	-0.953083801	1.95E-28
ENSMUSG00000038305	Spats2l	-0.951942451	0.003744916
ENSMUSG00000037820	Tgm2	-0.948206063	1.76E-32
ENSMUSG00000052949	Rnf157	-0.946211173	0.001859094
ENSMUSG00000018899	Irf1	-0.945185724	1.41E-21
ENSMUSG00000029530	Ccr9	-0.942144264	0.045596836
ENSMUSG00000000805	Car4	-0.941816063	2.52E-12
ENSMUSG00000026395	Ptprc	-0.938640374	1.06E-06
ENSMUSG00000024742	Fen1	-0.937685746	0.008477322
ENSMUSG00000050957	Insl6	-0.932435499	3.51E-17
ENSMUSG00000038732	Mboat1	-0.931208479	4.81E-54
ENSMUSG00000053846	Lipg	-0.931201365	8.56E-06
ENSMUSG00000062064	Slc2a7	-0.930337472	5.57E-06
ENSMUSG00000020077	Srgn	-0.928744566	0.002054823
ENSMUSG00000072974	Gm4787	-0.92455721	0.019982045
ENSMUSG00000031093	Dock11	-0.922085297	0.000483816
ENSMUSG00000031824	6430548M08	-0.919377536	1.49E-08
ENSMUSG00000040430	Pitpnc1	-0.91913153	0.004454409
ENSMUSG00000028044	Cks1b	-0.91508854	0.000152616
ENSMUSG00000049439	Cyp20a1	-0.914944321	0.005286484
ENSMUSG00000028657	Ppt1	-0.907291396	3.86E-11
ENSMUSG00000038372	Gmds	-0.907075068	2.29E-29
ENSMUSG00000029007	Agtrap	-0.906781085	7.70E-05
ENSMUSG00000037754	Ppp1r16b	-0.901918745	1.76E-08
ENSMUSG00000026880	Stom	-0.901657429	1.14E-08
ENSMUSG00000032010	Usp2	-0.900734243	0.000645534
ENSMUSG00000039005	Tlr4	-0.898612662	0.018279328
ENSMUSG00000024165	Jpt2	-0.895168548	0.01628867
ENSMUSG00000037242	Clic4	-0.889214509	7.07E-07

ENSMUSG00000026278	Bok	1.230449969	1.71E-05
ENSMUSG00000031877	Ces2g	1.226510847	1.21E-141
ENSMUSG00000036880	Acaa2	1.225615702	1.96E-69
ENSMUSG00000005320	Fgfr4	1.222241248	0.031864013
ENSMUSG00000066595	Flvcr1	1.219235877	2.57E-59
ENSMUSG00000037344	Slc12a9	1.217055082	0.004342645
ENSMUSG00000070056	Mfhas1	1.215940236	7.35E-31
ENSMUSG00000047562	Mmp10	1.215839031	3.35E-13
ENSMUSG00000023070	Rgn	1.215004885	0.000147608
ENSMUSG00000030972	Acsm5	1.213119343	5.08E-07
ENSMUSG00000025194	Abcc2	1.203702463	2.77E-11
ENSMUSG00000005360	Slc1a3	1.203562711	1.44E-17
ENSMUSG00000087477	Gm13822	1.202200903	0.005636551
ENSMUSG00000027983	Cyp2u1	1.197354748	2.74E-12
ENSMUSG00000025915	Sgk3	1.196749202	9.38E-05
ENSMUSG00000031214	Ophn1	1.18877808	0.009317429
ENSMUSG00000002289	Angptl4	1.187845714	1.93E-07
ENSMUSG00000025197	Cyp2c23	1.186803777	3.38E-10
ENSMUSG00000026832	Cytip	1.182222736	4.42E-08
ENSMUSG00000045658	Pid1	1.179174379	0.01493476
ENSMUSG00000036216	Leap2	1.178769792	9.54E-30
ENSMUSG00000022799	Arhgap31	1.177766459	0.017330066
ENSMUSG00000053835	H2-T24	1.173206439	1.57E-06
ENSMUSG00000031790	Mmp15	1.172489387	1.17E-06
ENSMUSG00000024781	Lipa	1.171843428	2.34E-40
ENSMUSG00000027187	Cat	1.167863802	8.21E-25
ENSMUSG00000032311	Nrg4	1.167349271	4.92E-09
ENSMUSG00000055053	Nfic	1.159787279	1.90E-09
ENSMUSG00000028435	Aqp3	1.157514301	4.13E-07
ENSMUSG00000026343	Gpr39	1.155326458	9.40E-05
ENSMUSG00000035311	Gnptab	1.151335805	9.31E-08
ENSMUSG00000042684	Npl	1.150170925	0.001788343
ENSMUSG00000022122	Ednrb	1.149088017	0.028830963
ENSMUSG00000034701	Neurod1	1.149003569	0.00237907
ENSMUSG00000111296	2010001M07	1.148063947	2.81E-77
ENSMUSG00000112805	Gm34574	1.143621994	0.001271606
ENSMUSG00000064105	Cnnm2	1.14286507	4.82E-07
ENSMUSG00000061898	Rbak	1.142489661	0.023715214
ENSMUSG00000046245	Pilra	1.141802701	0.040342647
ENSMUSG00000026659	Dusp12	1.138445015	2.92E-09
ENSMUSG00000029231	Pdgfra	1.137568773	5.24E-05
ENSMUSG00000047394	Odf3b	1.134913063	9.79E-08
ENSMUSG00000044676	Zfp612	1.132612348	0.046132894
ENSMUSG00000038068	Rnf144b	1.131919232	0.025963319
ENSMUSG00000049791	Fzd4	1.130178039	3.35E-06
ENSMUSG00000032841	Prr5l	1.129785282	0.031901129
ENSMUSG00000022389	Tef	1.127172925	0.041982779
ENSMUSG00000025780	Itih5	1.121303226	3.77E-06
ENSMUSG00000055114	Anxa13	1.121142388	1.74E-16
ENSMUSG00000038473	Nos1ap	1.11950326	5.82E-08
ENSMUSG00000022708	Zbtb20	1.114547025	0.001428095
ENSMUSG00000030769	Slc5a11	1.114118051	1.88E-08
ENSMUSG00000048440	Cyp4f16	1.109289372	1.45E-11
ENSMUSG00000018378	2210416O15	1.107021258	0.024422864
ENSMUSG00000054263	Lifr	1.099037019	3.22E-05
ENSMUSG00000053414	Hunk	1.094442447	0.012911808
ENSMUSG00000030340	Scnn1a	1.087444931	0.003614544
ENSMUSG00000030605	Mfge8	1.08738048	0.002308611
ENSMUSG00000060402	Chst8	1.0844176	1.37E-09
ENSMUSG00000050288	Fzd2	1.08209951	0.020694027
ENSMUSG00000078139	AK157302	1.07828863	0.030553863
ENSMUSG00000074071	Fam169b	1.076992265	0.01250621
ENSMUSG00000003555	Cyp17a1	1.075614213	0.008101647
ENSMUSG00000104011	Gm32391	1.073912221	0.036163918
ENSMUSG00000048486	Fitm2	1.070709246	0.021047305
ENSMUSG00000028383	Hsdl2	1.069652624	1.10E-06
ENSMUSG00000024747	Aldh1a7	1.067677888	1.56E-11
ENSMUSG00000027870	Hao2	1.065066901	0.000276627
ENSMUSG00000064363	ND4	1.060420012	4.41E-08
ENSMUSG00000045414	Dipk2a	1.057666854	5.05E-08
ENSMUSG00000037905	Bri3bp	1.056316389	1.08E-16
ENSMUSG00000058997	Vwa8	1.055550487	3.55E-08
ENSMUSG00000029334	Prkg2	1.055167228	1.11E-18
ENSMUSG00000030691	Fchsd2	1.051098864	3.53E-12
ENSMUSG00000030878	Cdr2	1.049004313	6.78E-26
ENSMUSG00000062044	Lmtk3	1.047231399	0.032832312
ENSMUSG00000029781	Fkbp9	1.045593324	0.005471906
ENSMUSG00000027962	Vcam1	1.04530333	0.006652296
ENSMUSG00000055748	Gsdmc4	1.04197165	1.02E-19
ENSMUSG00000041660	Bbox1	1.040207227	1.67E-41
ENSMUSG00000048332	Lhfpl6	1.040185201	0.000273719
ENSMUSG00000040102	Klhl42	1.038739473	0.046022438
ENSMUSG00000017929	B4galt5	1.034930362	6.45E-08
ENSMUSG00000025813	Homer2	1.034396067	4.08E-12
ENSMUSG00000019359	Gdpd2	1.025844343	4.61E-32
ENSMUSG00000020600	Slc7a15	1.025409831	8.49E-05
ENSMUSG00000034751	Mast4	1.024710853	1.92E-08
ENSMUSG00000086712	Mexis	1.023352281	3.26E-47
ENSMUSG00000028127	Abcd3	1.022363727	2.85E-25
ENSMUSG00000000876	Pxmp4	1.020971154	2.77E-09
ENSMUSG00000048280	Zfp738	1.020535922	0.025789335
ENSMUSG00000001313	Rnd2	1.020472991	2.40E-12
ENSMUSG00000020538	Srebf1	1.019317398	5.33E-05
ENSMUSG00000010025	Aldh3a2	1.018725796	8.22E-21
ENSMUSG00000022514	Il1rap	1.017949356	0.024161758
ENSMUSG00000046096	Mosmo	1.013530978	0.036684687
ENSMUSG00000037797	Adh4	1.013197019	1.10E-85
ENSMUSG00000038872	Zfhx3	1.011437892	0.000111517
ENSMUSG00000052763	Zfp212	1.011180811	0.000420713
ENSMUSG00000013150	Gfod2	1.009670183	0.000575867
ENSMUSG00000028494	Plin2	1.008258585	3.62E-13
ENSMUSG00000003585	Sec14l2	1.005247591	2.29E-15
ENSMUSG00000023764	Sfi1	1.002612748	0.008063469
ENSMUSG00000066026	Dhrs3	1.001734529	3.68E-05
ENSMUSG00000035877	Zhx3	0.998242423	0.007327965
ENSMUSG00000024818	Slc25a45	0.996800028	2.72E-37
ENSMUSG00000008855	Hdac5	0.996698368	0.000813439
ENSMUSG00000028088	Fmo5	0.996001034	4.58E-11
ENSMUSG00000025402	Nab2	0.994690601	0.028064487
ENSMUSG00000036231	Agr3	0.988541877	0.004943332
ENSMUSG00000036473	Tbc1d24	0.987344152	8.95E-08
ENSMUSG00000010122	Slc47a1	0.986896146	3.99E-12
ENSMUSG00000049577	Zfpm1	0.985641444	5.20E-08
ENSMUSG00000048550	Thnsl1	0.985602987	0.010310755
ENSMUSG00000030643	Rab30	0.985576678	3.50E-11
ENSMUSG00000005534	Insr	0.984562031	0.000127622
ENSMUSG00000001657	Hoxc8	0.983764206	8.06E-05
ENSMUSG00000027876	Reg4	0.978285859	5.20E-24
ENSMUSG00000020019	Ntn4	0.978107397	0.00104114
ENSMUSG00000041762	Gpr155	0.978104275	1.17E-13
ENSMUSG00000120645	Gm29688	0.977379951	1.14E-08
ENSMUSG00000048264	Dip2c	0.977224724	0.008590019

ENSMUSG00000030340	Scnn1a	-1.065351195	0.003614544
ENSMUSG00000060548	Tnfrsf19	-1.062041312	0.001725285
ENSMUSG00000020774	Aspa	-1.05689823	2.65E-10
ENSMUSG00000022003	Slc25a30	-1.05141129	0.001873809
ENSMUSG00000053279	Aldh1a1	-1.050441896	3.19E-45
ENSMUSG00000027524	Edn3	-1.046498409	6.03E-28
ENSMUSG00000025163	Cd7	-1.044900477	5.42E-16
ENSMUSG00000096948	Gm4221	-1.038350042	0.01653401
ENSMUSG00000057234	Mettl15	-1.032792336	0.006675101
ENSMUSG00000047945	Marcksl1	-1.030776774	1.34E-25
ENSMUSG00000116802	Gm5165	-1.030775199	0.007958367
ENSMUSG00000004814	Ccl24	-1.029496392	2.10E-31
ENSMUSG00000021895	Arhgef3	-1.025058451	4.71E-13
ENSMUSG00000044628	Rnf208	-1.02171302	3.94E-09
ENSMUSG00000027219	Slc28a2	-1.016660799	1.02E-17
ENSMUSG00000049134	Nrap	-1.011811168	0.040924215
ENSMUSG00000044952	Kctd21	-1.010958848	0.049075975
ENSMUSG00000027346	Gpcpd1	-1.01059145	2.35E-37
ENSMUSG00000029385	Ccng2	-1.01037391	4.43E-10
ENSMUSG00000037148	Arhgap10	-1.010131347	0.048922138
ENSMUSG00000004612	Nkg7	-1.007567245	6.72E-11
ENSMUSG00000037649	H2-DMa	-1.00529246	1.56E-39
ENSMUSG00000037679	Inf2	-1.001977124	0.005563449
ENSMUSG00000030785	Cox6a2	-0.999420635	0.023413671
ENSMUSG00000019854	Reps1	-0.998779757	1.21E-05
ENSMUSG00000022364	Tbc1d31	-0.992849237	3.61E-13
ENSMUSG00000047205	Dusp18	-0.992691797	0.020127666
ENSMUSG00000074647	Fam83c	-0.990667545	0.000459844
ENSMUSG00000039713	Plekhg5	-0.990202272	7.73E-06
ENSMUSG00000024253	Dync2li1	-0.986341133	0.015760246
ENSMUSG00000034472	Rasd2	-0.982956939	3.65E-07
ENSMUSG00000032094	Cd3d	-0.981818795	6.69E-07
ENSMUSG00000034265	Zdhhc14	-0.981159162	0.009979354
ENSMUSG00000034361	Cpne2	-0.974436652	1.75E-09
ENSMUSG00000025396	Hsd17b6	-0.973235451	8.79E-56
ENSMUSG00000085873	Ttc39aos1	-0.970700729	0.006819693
ENSMUSG00000053414	Hunk	-0.969805936	0.012911808
ENSMUSG00000039699	Batf2	-0.968982523	2.17E-10
ENSMUSG00000079260	Tmppe	-0.967763528	0.046461791
ENSMUSG00000025092	Hspa12a	-0.967202326	3.48E-25
ENSMUSG00000024381	Bin1	-0.965659773	0.000150298
ENSMUSG00000042010	Acacb	-0.964171099	0.005923203
ENSMUSG00000002944	Cd36	-0.963409015	9.76E-11
ENSMUSG00000040435	Ppp1r15a	-0.962320568	0.046438592
ENSMUSG00000028982	Slc25a33	-0.960655638	0.0010711
ENSMUSG00000025810	Nrp1	-0.9595673	0.002285352
ENSMUSG00000000409	Lck	-0.958996928	0.004336286
ENSMUSG00000071551	Akr1c19	-0.958072764	7.89E-21
ENSMUSG00000031490	Eif4ebp1	-0.954982831	0.000353571
ENSMUSG00000028521	Slc35d1	-0.954718685	0.001158736
ENSMUSG00000035547	Capn5	-0.953916517	2.44E-05
ENSMUSG00000017210	Med24	-0.949899693	0.008168715
ENSMUSG00000012187	Mogat1	-0.948676933	0.028413825
ENSMUSG00000032263	Bckdhb	-0.948455237	0.030150408
ENSMUSG00000039062	Anpep	-0.94546259	1.18E-07
ENSMUSG00000040740	Slc25a34	-0.94390793	0.000414469
ENSMUSG00000038351	Sgsm2	-0.943180253	0.038018638
ENSMUSG00000002324	Rec8	-0.943054702	5.91E-06
ENSMUSG00000074439	Defa5	-0.942511861	2.55E-09
ENSMUSG00000032420	Nt5e	-0.942279772	2.12E-15
ENSMUSG00000063142	Kcnma1	-0.941003501	0.01172516
ENSMUSG00000019577	Pdk4	-0.939700453	2.03E-06
ENSMUSG00000020143	Dock2	-0.939606888	0.027846155
ENSMUSG00000020526	Znhit3	-0.937665992	0.034194934
ENSMUSG00000030048	Gkn3	-0.934553826	8.30E-05
ENSMUSG00000061845	Defa35	-0.930513088	2.54E-15
ENSMUSG00000025780	Itih5	-0.929631679	3.77E-06
ENSMUSG00000028587	Orc1	-0.92935167	0.03167774
ENSMUSG00000021062	Rab15	-0.927790339	1.58E-10
ENSMUSG00000070691	Runx3	-0.926612393	0.011130369
ENSMUSG00000025041	Nt5c2	-0.925582298	0.000105358
ENSMUSG00000070427	Il18bp	-0.923821851	2.76E-05
ENSMUSG00000003849	Nqo1	-0.921860124	3.63E-12
ENSMUSG00000064427	Gm22748	-0.92174266	8.63E-20
ENSMUSG00000089809	A930011G23	-0.921600269	0.000130203
ENSMUSG00000032245	Cln6	-0.91815923	0.000303194
ENSMUSG00000020865	Abcc3	-0.917961867	0.000579926
ENSMUSG00000013707	Tnfaip8l2	-0.913529005	6.78E-06
ENSMUSG00000010025	Aldh3a2	-0.910066806	8.22E-21
ENSMUSG00000022696	Sidt1	-0.908193633	5.92E-09
ENSMUSG00000025037	Maoa	-0.9075185	1.01E-18
ENSMUSG00000040220	Gas8	-0.906173029	0.040524515
ENSMUSG00000056234	Ncoa4	-0.901959232	0.000550084
ENSMUSG00000029055	Plch2	-0.898710148	0.011798637
ENSMUSG00000040751	Lat2	-0.898515368	9.51E-05
ENSMUSG00000053846	Lipg	-0.898386269	8.56E-06
ENSMUSG00000027358	Bmp2	-0.895778622	1.06E-08
ENSMUSG00000027452	Acss1	-0.89082583	1.46E-13
ENSMUSG00000021565	Slc6a19	-0.890274275	6.26E-09
ENSMUSG00000026380	Tfcp2l1	-0.8824314	4.87E-15
ENSMUSG00000020899	Pfas	-0.879163515	7.72E-06
ENSMUSG00000031626	Sorbs2	-0.877673978	0.000881561
ENSMUSG00000079547	H2-DMb1	-0.873406585	2.13E-166
ENSMUSG00000031937	Vstm5	-0.872817107	0.022279962
ENSMUSG00000029134	Plb1	-0.870742862	1.24E-244
ENSMUSG00000055827	Gsdmc3	-0.870742113	5.06E-13
ENSMUSG00000020620	Abca8b	-0.870741841	5.10E-07
ENSMUSG00000022376	Adcy8	-0.870741763	8.23E-05
ENSMUSG00000032625	Thsd7a	-0.87074173	9.76E-06
ENSMUSG00000050201	Otop2	-0.870741706	4.11E-05
ENSMUSG00000009628	Tex15	-0.870741672	0.001583783
ENSMUSG00000039682	Lap3	-0.868948179	5.94E-07
ENSMUSG00000048911	Rnf24	-0.868881662	0.00083789
ENSMUSG00000025557	Slc15a1	-0.865223521	0.000789598
ENSMUSG00000038172	Ttc39b	-0.864527959	0.00017854
ENSMUSG00000046997	Spsb4	-0.864285752	0.037632198
ENSMUSG00000045658	Pid1	-0.864057939	0.01493476
ENSMUSG00000035811	Ugt2b35	-0.860483591	2.32E-35
ENSMUSG00000070704	Ugt2b36	-0.849722425	9.18E-06
ENSMUSG00000025825	Iscu	-0.849683778	0.036793326
ENSMUSG00000064140	Trim38	-0.849247739	6.80E-16
ENSMUSG00000020709	Adap2	-0.849127001	0.029880846
ENSMUSG00000060586	H2-Eb1	-0.848707076	3.02E-156
ENSMUSG00000032093	Cd3e	-0.846766263	0.000509931
ENSMUSG00000026979	Psd4	-0.842246014	0.033620746
ENSMUSG00000045282	Tmem86b	-0.841761634	3.66E-09
ENSMUSG00000044165	Bcl2l15	-0.840813582	3.98E-11
ENSMUSG00000020108	Ddit4	-0.838832791	0.014911637
ENSMUSG00000017802	Retreg3	-0.837754281	3.66E-05
ENSMUSG00000000440	Pparg	-0.835288764	3.31E-14
ENSMUSG00000029630	Cyp3a25	-0.832624975	4.92E-59
ENSMUSG00000040820	Hlcs	-0.827906135	0.022315015

ENSMUSG00000013483	Card14	0.996347004	0.006547487
ENSMUSG00000028549	Itgb3bp	0.993697455	0.03949428
ENSMUSG00000031097	Tnni2	0.991799453	0.011036514
ENSMUSG00000027375	Mal	0.989949277	0.043828705
ENSMUSG00000020641	Rsad2	0.989355596	2.51E-13
ENSMUSG00000020231	Dip2a	0.987352677	0.002463622
ENSMUSG00000029605	Oas1b	0.983990515	2.33E-08
ENSMUSG00000056665	Them6	0.983562441	0.032125241
ENSMUSG00000020647	Ncoa1	0.983161269	3.57E-07
ENSMUSG00000045237	Eola1	0.982748817	5.18E-05
ENSMUSG00000057440	Mpp7	0.979952402	6.17E-06
ENSMUSG00000032114	Slc37a4	0.975647313	2.10E-85
ENSMUSG00000033170	Card10	0.975364897	0.000607797
ENSMUSG00000030956	Fam53b	0.972632968	0.001920703
ENSMUSG00000017718	Afmid	0.970688556	0.011908367
ENSMUSG00000047822	Angptl8	0.970441144	8.25E-05
ENSMUSG00000025034	Trim8	0.969584585	0.00014056
ENSMUSG00000034634	Ly6d	0.969447736	0.000506125
ENSMUSG00000072235	Tuba1a	0.968372338	0.000544036
ENSMUSG00000011034	Slc5a1	0.966194471	1.41E-34
ENSMUSG00000023055	Calcoco1	0.965669122	0.033378418
ENSMUSG00000041237	Pklr	0.964695149	1.49E-16
ENSMUSG00000022305	Lrp12	0.961513135	6.74E-09
ENSMUSG00000029561	Oasl2	0.95781666	1.51E-06
ENSMUSG00000022651	Retnlg	0.954350859	0.017329148
ENSMUSG00000035451	Foxa1	0.954326676	1.28E-13
ENSMUSG00000037499	Nenf	0.953570794	9.02E-17
ENSMUSG00000078771	Evi2a	0.953087788	0.00296642
ENSMUSG00000017390	Aldoc	0.949840831	2.72E-20
ENSMUSG00000028944	Prkag2	0.949061555	1.41E-21
ENSMUSG00000025007	Aldh18a1	0.948812155	6.89E-21
ENSMUSG00000021506	Pitx1	0.947064547	6.00E-06
ENSMUSG00000074918	Inafm2	0.944232145	5.26E-07
ENSMUSG00000102047	2010010A06	0.944172069	0.000251934
ENSMUSG00000062352	Itgb1bp1	0.943137003	0.00173483
ENSMUSG00000022021	Diaph3	0.942739336	0.025142669
ENSMUSG00000006517	Mvd	0.941228486	4.22E-05
ENSMUSG00000079339	Ifit1bl1	0.938823654	1.33E-13
ENSMUSG00000020828	Pld2	0.935895128	0.00028098
ENSMUSG00000043789	Vwce	0.935851649	2.88E-14
ENSMUSG00000014543	Klra17	0.935553988	9.39E-07
ENSMUSG00000063239	Grm4	0.929432804	0.000248731
ENSMUSG00000021835	Bmp4	0.927709067	1.15E-06
ENSMUSG00000046245	Pilra	0.92717006	0.040342647
ENSMUSG00000073889	Il11ra1	0.924850902	0.016821364
ENSMUSG00000052229	Gpr17	0.924054804	0.008842857
ENSMUSG00000004263	Atn1	0.922961949	3.09E-05
ENSMUSG00000002489	Tiam1	0.922606615	0.00032707
ENSMUSG00000029162	Khk	0.922115007	2.67E-89
ENSMUSG00000046020	Pofut1	0.920748695	0.00173483
ENSMUSG00000074738	Fndc10	0.916562677	6.37E-14
ENSMUSG00000039146	Ifi44l	0.915292764	0.032942047
ENSMUSG00000043448	Gjc2	0.914718573	0.000900876
ENSMUSG00000005899	Smpd4	0.913021155	0.010549069
ENSMUSG00000034275	Igsf9b	0.912401367	1.74E-06
ENSMUSG00000029167	Ppargc1a	0.912274763	4.48E-33
ENSMUSG00000095193	Gm20939	0.909973059	7.54E-08
ENSMUSG00000026579	F5	0.909715776	2.67E-06
ENSMUSG00000046157	Tmem229b	0.906977665	0.003932215
ENSMUSG00000026430	Rassf5	0.903687283	0.040265882
ENSMUSG00000028689	Ccdc163	0.902616485	0.042917017
ENSMUSG00000014547	Wdfy2	0.9018164	0.024666542
ENSMUSG00000019850	Tnfaip3	0.901694442	0.038132687
ENSMUSG00000059040	Eno1b	0.900068306	2.71E-20
ENSMUSG00000000861	Bcl11a	0.898733776	2.65E-05
ENSMUSG00000040712	Camta2	0.896131416	0.001232666
ENSMUSG00000018378	2210416O15	0.893312388	0.024422864
ENSMUSG00000012519	Mlkl	0.892859664	1.49E-07
ENSMUSG00000030583	Sipa1l3	0.892242427	4.56E-06
ENSMUSG00000039474	Wfs1	0.890100481	0.001811975
ENSMUSG00000030397	Mark4	0.889377176	2.16E-05
ENSMUSG00000043252	Tmem64	0.881510926	4.25E-05
ENSMUSG00000022408	Fam83f	0.881032813	2.90E-05
ENSMUSG00000035992	Fnip1	0.87913019	0.000109679
ENSMUSG00000034057	Myrfl	0.878516057	6.26E-06
ENSMUSG00000003283	Hck	0.876075895	0.000113552
ENSMUSG00000026235	Epha4	0.875143689	5.84E-12
ENSMUSG00000036986	Pml	0.871996569	0.000474505
ENSMUSG00000039741	Bahcc1	0.871391983	0.015002271
ENSMUSG00000029554	Mad1l1	0.871206357	0.014212431
ENSMUSG00000010175	Prox1	0.864304853	4.54E-14
ENSMUSG00000078317	F8a	0.864149373	0.010305582
ENSMUSG00000061898	Rbak	0.862585032	0.023715214
ENSMUSG00000002028	Kmt2a	0.855574138	7.19E-16
ENSMUSG00000022816	Fstl1	0.854668912	0.005185075
ENSMUSG00000035692	Isg15	0.854129255	1.10E-14
ENSMUSG00000045038	Prkce	0.854019213	1.12E-09
ENSMUSG00000063524	Eno1	0.853314336	3.75E-15
ENSMUSG00000029309	Sparcl1	0.850248326	7.97E-12
ENSMUSG00000028854	Slc9a1	0.849973483	0.002881981
ENSMUSG00000021614	Vcan	0.849526913	0.024836426
ENSMUSG00000013629	Cad	0.849450721	8.26E-07
ENSMUSG00000030934	Oat	0.846477209	1.00E-06
ENSMUSG00000079685	Ulbp1	0.845531451	0.006403808
ENSMUSG00000035764	Fbxo45	0.842558913	0.002282152
ENSMUSG00000034154	Ino80	0.842446162	0.00216492
ENSMUSG00000029007	Agtrap	0.841279637	7.70E-05
ENSMUSG00000057969	Sema3b	0.840985403	0.032375091
ENSMUSG00000030921	Trim30a	0.840667036	9.79E-09
ENSMUSG00000003526	Prodh	0.839752083	4.29E-06
ENSMUSG00000020638	Cmpk2	0.838123614	1.79E-17
ENSMUSG00000026842	Abl1	0.837732632	0.000584911
ENSMUSG00000021485	Mxd3	0.837684076	0.033029568
ENSMUSG00000055737	Ghr	0.83501693	4.02E-10
ENSMUSG00000032579	Hemk1	0.834252197	0.01118386
ENSMUSG00000069808	Tlcd3a	0.833461126	0.000377942
ENSMUSG00000020387	Jade2	0.832746457	0.016803978
ENSMUSG00000052135	Foxo6	0.831746433	3.25E-13
ENSMUSG00000030103	Bhlhe40	0.831152806	0.000519872
ENSMUSG00000032232	Cgnl1	0.824986855	0.00447197
ENSMUSG00000110444	Gm10033	0.824629622	0.047844666
ENSMUSG00000026483	Niban1	0.820662135	0.012218937
ENSMUSG00000021687	Scamp1	0.810861704	7.54E-11
ENSMUSG00000041426	Hibch	0.810239465	0.000151987
ENSMUSG00000029851	Tcaf2	0.807335176	0.003449118
ENSMUSG00000040653	Ppp1r14c	0.801864973	0.01525031
ENSMUSG00000027962	Vcam1	0.801336795	0.006652296
ENSMUSG00000042035	Igsf3	0.801176025	0.000266846
ENSMUSG00000048537	Phldb1	0.799393241	3.76E-05
ENSMUSG00000056608	Chd9	0.797226931	0.013314373
ENSMUSG00000073418	C4b	0.796457681	0.03056972
ENSMUSG00000017677	Wsb1	0.796320608	0.01947398

ENSMUSG00000029095	Ablim2	-0.888412468	0.001126048
ENSMUSG00000041431	Ccnb1	-0.888241599	0.000331783
ENSMUSG00000040204	Pclaf	-0.886970519	0.000144948
ENSMUSG00000116542	Cd200l2	-0.885022882	0.013784498
ENSMUSG00000025176	Hoga1	-0.885004953	0.019869589
ENSMUSG00000041515	Irf8	-0.883610003	3.09E-15
ENSMUSG00000017697	Ada	-0.881327496	1.40E-57
ENSMUSG00000021993	Mipep	-0.880976191	0.017466762
ENSMUSG00000059363	Fxn	-0.880214292	0.038521767
ENSMUSG00000028459	Cd72	-0.880068925	0.015801549
ENSMUSG00000072082	Ccnf	-0.873341569	0.00695745
ENSMUSG00000013643	Lypd8	-0.8711037	2.88E-11
ENSMUSG00000021485	Mxd3	-0.86723113	0.033029568
ENSMUSG00000037946	Fgd3	-0.866620338	0.001136289
ENSMUSG00000019853	Hebp2	-0.863113015	0.00160016
ENSMUSG00000002055	Spag5	-0.861163843	0.000393845
ENSMUSG00000036136	Fam110c	-0.856249211	0.000604495
ENSMUSG00000097779	4833407H14	-0.854261305	1.27E-07
ENSMUSG00000038564	Ift172	-0.854071318	3.52E-12
ENSMUSG00000059149	Mfsd4a	-0.848649207	1.66E-08
ENSMUSG00000000628	Hk2	-0.846149428	3.51E-24
ENSMUSG00000065956	Defa37	-0.845138675	0.005908885
ENSMUSG00000035578	Iqcg	-0.842497844	0.001420242
ENSMUSG00000017716	Birc5	-0.839080637	1.65E-06
ENSMUSG00000028256	Odf2l	-0.834804238	5.85E-07
ENSMUSG00000001865	Cpa3	-0.834428753	0.030584039
ENSMUSG00000023505	Cdca3	-0.834065073	4.01E-05
ENSMUSG00000033307	Mif	-0.832720425	1.24E-07
ENSMUSG00000002679	Med6	-0.832318403	0.032619666
ENSMUSG00000048782	Insc	-0.829253554	4.09E-05
ENSMUSG00000030671	Pde3b	-0.829246341	0.008348828
ENSMUSG00000023393	Slc17a9	-0.828170985	0.000544034
ENSMUSG00000100714	2010308F09	-0.827390592	7.74E-08
ENSMUSG00000032397	Tipin	-0.825155759	0.013715055
ENSMUSG00000068547	Clca4a	-0.824715212	8.47E-14
ENSMUSG00000007872	Id3	-0.824305798	3.92E-11
ENSMUSG00000088252	Snord13	-0.823676837	2.01E-14
ENSMUSG00000005470	Asf1b	-0.822916117	0.015758577
ENSMUSG00000029385	Ccng2	-0.820524095	4.43E-10
ENSMUSG00000041718	Alg13	-0.813602601	0.026521418
ENSMUSG00000037542	Aldh8a1	-0.813459681	0.037401339
ENSMUSG00000032554	Trf	-0.811497345	3.28E-06
ENSMUSG00000048327	Ckap2l	-0.808389682	4.27E-06
ENSMUSG00000026430	Rassf5	-0.805802967	0.040265882
ENSMUSG00000071041	Impdh2-ps	-0.805446415	0.008400777
ENSMUSG00000074272	Ceacam1	-0.803181999	2.88E-41
ENSMUSG00000031257	Nox1	-0.803107658	0.00318856
ENSMUSG00000039795	Zfand1	-0.801137088	0.00503928
ENSMUSG00000026628	Atf3	-0.800599277	0.047056656
ENSMUSG00000025747	Tyms	-0.799822821	0.020395193
ENSMUSG00000039942	Ptger4	-0.799791227	2.92E-10
ENSMUSG00000026520	Pycr2	-0.798754257	0.000657124
ENSMUSG00000047443	Erfe	-0.79823954	0.018426056
ENSMUSG00000020415	Pttg1	-0.797986842	2.13E-10
ENSMUSG00000055312	Them7	-0.797549807	0.035513762
ENSMUSG00000006715	Gmnn	-0.796580048	3.85E-07
ENSMUSG00000022351	Sqle	-0.793826907	8.17E-20
ENSMUSG00000020846	Rflnb	-0.79272668	2.01E-05
ENSMUSG00000067367	Lyar	-0.79067152	9.14E-09
ENSMUSG00000039356	Exosc2	-0.789393844	0.000573051
ENSMUSG00000039883	Lrrc17	-0.788554585	0.026272758
ENSMUSG00000038943	Prc1	-0.788429723	8.38E-10
ENSMUSG00000035236	Scai	-0.786333837	0.04902945
ENSMUSG00000028940	Hes2	-0.786019762	0.016462958
ENSMUSG00000034570	Inpp5j	-0.782774342	3.34E-12
ENSMUSG00000028085	Gatb	-0.779689846	0.000519166
ENSMUSG00000117621	Hspe1-rs1	-0.775852951	0.000889768
ENSMUSG00000116802	Gm5165	-0.775080311	0.007958367
ENSMUSG00000037953	A4gnt	-0.774580054	0.040521671
ENSMUSG00000025245	Lztfl1	-0.774312478	0.032752455
ENSMUSG00000063236	1110038F14	-0.771234295	0.00529627
ENSMUSG00000043592	Unc5cl	-0.770620505	0.00112905
ENSMUSG00000033191	Tie1	-0.770168637	0.004349782
ENSMUSG00000027715	Ccna2	-0.769339418	1.79E-10
ENSMUSG00000031546	Gins4	-0.767570571	0.02525604
ENSMUSG00000090946	Ccdc71l	-0.764767006	0.000734215
ENSMUSG00000045273	Cenph	-0.763773542	0.006013372
ENSMUSG00000070348	Ccnd1	-0.761807194	2.67E-63
ENSMUSG00000039648	Kyat1	-0.761356775	0.000199123
ENSMUSG00000020774	Aspa	-0.760970039	2.65E-10
ENSMUSG00000006519	Cyba	-0.758825401	4.64E-18
ENSMUSG00000042726	Trafd1	-0.757498098	1.55E-09
ENSMUSG00000010830	Kdelr3	-0.756580742	1.77E-07
ENSMUSG00000028671	Gale	-0.755497066	7.57E-09
ENSMUSG00000024056	Ndc80	-0.751811725	0.007864052
ENSMUSG00000030342	Cd9	-0.750957089	3.87E-09
ENSMUSG00000074437	Defa29	-0.745045157	6.98E-17
ENSMUSG00000052485	Tmem171	-0.744548801	5.40E-08
ENSMUSG00000025287	Acot9	-0.74430275	1.22E-05
ENSMUSG00000022587	Ly6e	-0.744081244	0.024232465
ENSMUSG00000032218	Ccnb2	-0.741534358	0.016051
ENSMUSG00000037579	Kcnh3	-0.739472655	0.001725962
ENSMUSG00000031410	Nxf7	-0.73937038	0.006118009
ENSMUSG00000021250	Fos	-0.738967827	0.001948042
ENSMUSG00000054404	Slfn5	-0.738639557	0.003397837
ENSMUSG00000019942	Cdk1	-0.737812622	0.00848051
ENSMUSG00000027309	Dnaaf9	-0.730806554	1.52E-16
ENSMUSG00000048992	Prss32	-0.730335857	3.22E-15
ENSMUSG00000028415	Spink4	-0.725430843	0.000198568
ENSMUSG00000029401	Rilpl2	-0.72524991	1.37E-06
ENSMUSG00000020607	Lratd1	-0.719760703	0.01854918
ENSMUSG00000050761	Gp1bb	-0.718546855	9.06E-15
ENSMUSG00000023960	Enpp5	-0.717400128	3.10E-06
ENSMUSG00000021273	Fdft1	-0.717293874	2.13E-07
ENSMUSG00000022769	Sdf2l1	-0.714596363	0.027177921
ENSMUSG00000064581	Gm25133	-0.712861569	4.02E-09
ENSMUSG00000033107	Rnf125	-0.712048673	2.79E-06
ENSMUSG00000037722	Gnpnat1	-0.711851443	2.26E-06
ENSMUSG00000030786	Itgam	-0.710892271	0.001599213
ENSMUSG00000027306	Nusap1	-0.709244811	6.43E-07
ENSMUSG00000021998	Lcp1	-0.704728479	0.002960495
ENSMUSG00000027331	Knstrn	-0.703728915	0.014572191
ENSMUSG00000061758	Akr1b10	-0.703717307	3.36E-08
ENSMUSG00000079018	Ly6c1	-0.703486449	0.016400404
ENSMUSG00000022186	Oxct1	-0.702963705	3.51E-06
ENSMUSG00000051234	Rnf7	-0.70223657	2.33E-06
ENSMUSG00000031897	Psmb10	-0.702224015	2.06E-16
ENSMUSG00000003355	Fkbp11	-0.701867671	0.016382756
ENSMUSG00000027030	Stk39	-0.701845771	1.21E-11
ENSMUSG00000038151	Prdm1	-0.699176717	0.01152163
ENSMUSG00000001403	Ube2c	-0.698626015	8.73E-07
ENSMUSG00000074129	Rpl13a	-0.697764612	0.001667726

ENSMUSG00000052698	Tln2	0.977018408	1.53E-11
ENSMUSG00000037995	Igsf9	0.97621046	7.98E-09
ENSMUSG00000042010	Acacb	0.970446373	0.005923203
ENSMUSG00000025002	Cyp2c55	0.969267984	1.46E-47
ENSMUSG00000028944	Prkag2	0.968846094	1.41E-21
ENSMUSG00000057286	St6galnac2	0.968811937	0.014086736
ENSMUSG00000044952	Kctd21	0.965831645	0.049075975
ENSMUSG00000097852	4933405D12	0.964930621	0.014260624
ENSMUSG00000020220	Vps13d	0.958803872	1.47E-05
ENSMUSG00000028708	Mknk1	0.95862025	0.026902809
ENSMUSG00000028005	Gucy1b1	0.958192718	0.049627004
ENSMUSG00000013878	Rnf170	0.95811524	0.001649073
ENSMUSG00000074170	Plekhf1	0.957498521	7.76E-10
ENSMUSG00000032080	Apoa4	0.956922333	1.08E-93
ENSMUSG00000110573	Gm5485	0.955712643	3.30E-31
ENSMUSG00000024731	Ms4a10	0.953922716	5.87E-29
ENSMUSG00000020258	Glyctk	0.953572465	0.000299517
ENSMUSG00000030834	Abcc6	0.953031116	6.86E-08
ENSMUSG00000005442	Cic	0.952633121	8.02E-06
ENSMUSG00000032098	Treh	0.952249849	6.66E-77
ENSMUSG00000001663	Gstt1	0.951323453	3.80E-149
ENSMUSG00000038702	Dsel	0.947731629	8.25E-14
ENSMUSG00000069805	Fbp1	0.940936077	8.73E-136
ENSMUSG00000032788	Pdxk	0.939915202	3.84E-07
ENSMUSG00000020681	Ace	0.938712104	0.002512482
ENSMUSG00000035041	Creb3l3	0.938592382	3.89E-31
ENSMUSG00000021620	Acot12	0.937756984	3.88E-06
ENSMUSG00000027350	Chgb	0.936995805	1.45E-08
ENSMUSG00000038776	Ephx1	0.934047879	3.26E-47
ENSMUSG00000061959	Ces1e	0.933772211	3.72E-45
ENSMUSG00000051483	Cbr1	0.93122133	3.42E-58
ENSMUSG00000015340	Cybb	0.930943273	0.045076778
ENSMUSG00000046805	Mpeg1	0.929729457	0.002898664
ENSMUSG00000056429	Tgoln1	0.928613393	2.04E-18
ENSMUSG00000073096	Lrrc61	0.928422728	0.031199374
ENSMUSG00000032066	Bco2	0.927307916	2.32E-21
ENSMUSG00000034265	Zdhhc14	0.926990958	0.009979354
ENSMUSG00000055541	Lair1	0.926555309	3.54E-07
ENSMUSG00000022946	Dop1b	0.924617343	0.003172722
ENSMUSG00000036905	C1qb	0.922333145	0.03229553
ENSMUSG00000005677	Nr1i3	0.921772891	2.20E-40
ENSMUSG00000024887	Asah2	0.921428342	0.000354217
ENSMUSG00000003452	Bicd1	0.919007067	5.90E-07
ENSMUSG00000060336	Zfp937	0.918634519	1.58E-06
ENSMUSG00000051727	Kctd14	0.915809226	1.04E-23
ENSMUSG00000020828	Pld2	0.915480151	0.00028098
ENSMUSG00000036304	Zdhhc23	0.908786311	3.14E-05
ENSMUSG00000091780	Sco2	0.90506219	3.15E-32
ENSMUSG00000042797	Aqp11	0.901800904	4.27E-15
ENSMUSG00000030207	Fam234b	0.901204937	0.004866308
ENSMUSG00000044017	Adgrd1	0.898323036	1.45E-11
ENSMUSG00000032766	Gng11	0.89392642	9.89E-06
ENSMUSG00000037664	Cdkn1c	0.893469241	1.49E-08
ENSMUSG00000024521	Pmaip1	0.8926503	0.000464015
ENSMUSG00000025007	Aldh18a1	0.892301421	6.89E-21
ENSMUSG00000022435	Upk3a	0.891302024	0.009203445
ENSMUSG00000018500	Adora2b	0.889148196	3.62E-24
ENSMUSG00000109695	Gm31166	0.888151099	0.000262578
ENSMUSG00000027956	Tmem144	0.887036898	1.47E-10
ENSMUSG00000018920	Cxcl16	0.884433438	8.52E-07
ENSMUSG00000036622	Atp13a2	0.884388789	7.53E-05
ENSMUSG00000028600	Podn	0.882102265	0.000104636
ENSMUSG00000032978	Guca2b	0.881744898	9.66E-17
ENSMUSG00000017664	Slc35c2	0.880734559	1.11E-09
ENSMUSG00000045441	Gprin3	0.875675563	0.000100861
ENSMUSG00000022231	Sema5a	0.873926949	3.60E-15
ENSMUSG00000027397	Slc20a1	0.872356492	0.033783473
ENSMUSG00000029478	Ncor2	0.872083253	4.06E-05
ENSMUSG00000050530	Fam171a1	0.871394392	0.000286573
ENSMUSG00000022210	Dhrs4	0.870601221	1.42E-27
ENSMUSG00000032359	Ctsh	0.867844261	5.50E-30
ENSMUSG00000018919	Tm4sf5	0.86755041	9.23E-21
ENSMUSG00000035004	Igsf6	0.86589304	0.027059533
ENSMUSG00000034871	Fam151a	0.865495561	2.68E-104
ENSMUSG00000056293	Gsdmc2	0.863286725	1.06E-17
ENSMUSG00000026380	Tfcp2l1	0.85922149	4.87E-15
ENSMUSG00000002332	Dhrs1	0.857587573	3.56E-139
ENSMUSG00000049303	Syt12	0.857172389	0.011283469
ENSMUSG00000035878	Hykk	0.856626762	0.000683235
ENSMUSG00000021759	Plpp1	0.855446622	2.46E-19
ENSMUSG00000027784	Ppm1l	0.852019815	0.031823412
ENSMUSG00000049690	Nckap5	0.851026904	1.20E-06
ENSMUSG00000030890	Ilk	0.850087409	0.000668681
ENSMUSG00000068130	Zfp442	0.849242597	4.70E-08
ENSMUSG00000026170	Cyp27a1	0.847658701	0.001153652
ENSMUSG00000036256	Igfbp7	0.846072852	0.00243075
ENSMUSG00000039474	Wfs1	0.842265091	0.001811975
ENSMUSG00000061825	Ces2c	0.841971328	3.66E-24
ENSMUSG00000061436	Hipk2	0.841031523	0.000256372
ENSMUSG00000064370	CYTB	0.841020723	2.21E-05
ENSMUSG00000018042	Cyb5r3	0.840488785	8.34E-40
ENSMUSG00000038866	Zcchc2	0.839901472	0.005337033
ENSMUSG00000001138	Cnnm3	0.839684943	0.001252868
ENSMUSG00000056133	Unc93a2	0.838021443	0.001767197
ENSMUSG00000057103	Nat8f1	0.837861875	4.04E-16
ENSMUSG00000025316	Banp	0.835419074	0.000291222
ENSMUSG00000038264	Sema7a	0.834826033	0.004409113
ENSMUSG00000027381	Bcl2l11	0.834099272	0.000242631
ENSMUSG00000064345	ND2	0.834009696	0.000450124
ENSMUSG00000029377	Ereg	0.832749427	8.57E-16
ENSMUSG00000046697	Enpp7	0.831602635	1.39E-24
ENSMUSG00000029167	Ppargc1a	0.82547068	4.48E-33
ENSMUSG00000039428	Tmem135	0.824868029	5.69E-10
ENSMUSG00000001482	Def8	0.824718256	0.003952614
ENSMUSG00000024777	Ppp2r5b	0.823655441	0.016317672
ENSMUSG00000064917	Snord138	0.820596583	0.019286429
ENSMUSG00000023044	Csad	0.820128167	0.003589867
ENSMUSG00000006567	Atp7b	0.819066011	6.09E-08
ENSMUSG00000028976	Slc2a5	0.818695281	4.44E-135
ENSMUSG00000036027	Pou2af2	0.818451616	4.41E-05
ENSMUSG00000026853	Crat	0.817594689	6.42E-07
ENSMUSG00000034450	Gulo	0.816604426	0.02169546
ENSMUSG00000043789	Vwce	0.81585665	2.88E-14
ENSMUSG00000031378	Abcd1	0.815479955	0.005392166
ENSMUSG00000018593	Sparc	0.814583112	0.047318673
ENSMUSG00000032357	Tinag	0.81151114	2.46E-06
ENSMUSG00000025867	Cplx2	0.811117899	0.048084422
ENSMUSG00000020226	Slc5a4b	0.810676368	4.45E-09
ENSMUSG00000010175	Prox1	0.809115928	4.54E-14
ENSMUSG00000032081	Apoc3	0.807990259	3.97E-50
ENSMUSG00000001864	Aif1l	0.807312604	0.020343029
ENSMUSG00000034863	Ano8	0.802197797	0.016284208

ENSMUSG00000106397	Gm21049	-0.827702386	3.76E-19
ENSMUSG00000043705	Capn13	-0.82688149	6.02E-08
ENSMUSG00000025350	Rdh5	-0.826480422	0.001820682
ENSMUSG00000013150	Gfod2	-0.825369399	0.000575867
ENSMUSG00000022748	Cmss1	-0.825117811	0.02142583
ENSMUSG00000021213	Akr1c13	-0.8221652	7.96E-53
ENSMUSG00000020681	Ace	-0.815362757	0.002512482
ENSMUSG00000026113	Inpp4a	-0.813481836	0.039231689
ENSMUSG00000028737	Aldh4a1	-0.812897685	9.02E-08
ENSMUSG00000044468	Tent5c	-0.808684637	1.64E-05
ENSMUSG00000051615	Rap2a	-0.807596618	3.12E-17
ENSMUSG00000027332	Ivd	-0.80729531	0.010550001
ENSMUSG00000023057	Fabp2	-0.802759343	0.000445521
ENSMUSG00000073096	Lrrc61	-0.800406455	0.031199374
ENSMUSG00000025176	Hoga1	-0.79915615	0.019869589
ENSMUSG00000001095	Slc13a2	-0.797820877	1.56E-44
ENSMUSG00000024215	Spdef	-0.797262843	7.71E-05
ENSMUSG00000028464	Tpm2	-0.79673777	0.010603278
ENSMUSG00000065259	Snora30	-0.796645512	0.00712989
ENSMUSG00000015341	Golga7	-0.79513657	0.045992602
ENSMUSG00000026614	Slc30a10	-0.792058601	1.80E-07
ENSMUSG00000021254	Gpatch2l	-0.791519699	0.035853265
ENSMUSG00000068547	Clca4a	-0.78475555	8.47E-14
ENSMUSG00000019762	Iyd	-0.784192379	6.05E-35
ENSMUSG00000027230	Creb3l1	-0.782392042	2.04E-16
ENSMUSG00000030091	Nup210	-0.780007105	0.000841206
ENSMUSG00000021211	Akr1c12	-0.77987346	1.69E-45
ENSMUSG00000024066	Xdh	-0.778751861	1.15E-10
ENSMUSG00000023048	Prr13	-0.778375514	4.15E-10
ENSMUSG00000031824	6430548M08	-0.776884898	1.49E-08
ENSMUSG00000028836	Slc30a2	-0.775399988	2.64E-09
ENSMUSG00000032350	Gclc	-0.770731111	9.32E-15
ENSMUSG00000026687	Aldh9a1	-0.766386539	2.48E-06
ENSMUSG00000029273	Sult1d1	-0.765746082	5.00E-18
ENSMUSG00000049047	Armcx3	-0.763933159	0.021984813
ENSMUSG00000029204	Rhoh	-0.76377409	0.001527109
ENSMUSG00000022657	Cd96	-0.762166298	0.019685121
ENSMUSG00000076757	Trgc4	-0.758461762	0.000858235
ENSMUSG00000060803	Gstp1	-0.754047632	4.63E-08
ENSMUSG00000032418	Me1	-0.753869276	3.60E-20
ENSMUSG00000009828	Cilk1	-0.752132738	3.62E-06
ENSMUSG00000040026	Saa3	-0.752086721	0.012161377
ENSMUSG00000048521	Cxcr6	-0.751989376	0.009191814
ENSMUSG00000054619	Mettl7a1	-0.751934934	0.043501025
ENSMUSG00000028378	Ptgr1	-0.75163124	1.83E-09
ENSMUSG00000035780	Ugt2a3	-0.746614526	1.57E-32
ENSMUSG00000044017	Adgrd1	-0.745878506	1.45E-11
ENSMUSG00000085427	6430710C18	-0.742151382	7.06E-07
ENSMUSG00000024764	Naa40	-0.740015746	0.032048707
ENSMUSG00000033213	AA467197	-0.739697283	1.96E-15
ENSMUSG00000074406	Zfp628	-0.737808961	0.020461029
ENSMUSG00000044252	Osbpl1a	-0.737243943	2.72E-09
ENSMUSG00000024646	Cyb5a	-0.736324283	4.24E-29
ENSMUSG00000068686	Cd59b	-0.734181089	3.48E-07
ENSMUSG00000005102	Eif2ak4	-0.732772558	0.000367421
ENSMUSG00000051674	Dcun1d4	-0.73101736	4.35E-05
ENSMUSG00000022371	Col14a1	-0.730951319	6.10E-05
ENSMUSG00000063206	Defa34	-0.730541087	2.76E-20
ENSMUSG00000024070	Prkd3	-0.729938555	0.00051001
ENSMUSG00000059775	Rps26-ps1	-0.729608935	0.00013855
ENSMUSG00000020182	Ddc	-0.728299462	2.65E-38
ENSMUSG00000004356	Utp20	-0.727988204	0.000279803
ENSMUSG00000005514	Por	-0.727846509	3.99E-13
ENSMUSG00000030220	Arhgdib	-0.727180112	0.006445494
ENSMUSG00000038415	Foxq1	-0.725185709	3.11E-09
ENSMUSG00000029847	Slc23a4	-0.72502491	1.72E-07
ENSMUSG00000000958	Slc7a7	-0.723596959	0.000645705
ENSMUSG00000031165	Was	-0.721263262	0.037045175
ENSMUSG00000079445	B3gnt7	-0.721090068	0.000414182
ENSMUSG00000063354	Slc39a4	-0.720195436	9.37E-11
ENSMUSG00000049154	Fam183b	-0.719385604	3.61E-15
ENSMUSG00000010830	Kdelr3	-0.717021056	1.77E-07
ENSMUSG00000038178	Slc43a2	-0.715886662	7.71E-07
ENSMUSG00000094662	Defa36	-0.715659053	0.023237477
ENSMUSG00000018820	Zfyve27	-0.715007531	0.003153198
ENSMUSG00000015312	Gadd45b	-0.714029953	0.007364716
ENSMUSG00000057933	Gsta2	-0.711824426	3.55E-39
ENSMUSG00000074272	Ceacam1	-0.710900746	2.88E-41
ENSMUSG00000024486	Hbegf	-0.710426902	0.004739645
ENSMUSG00000054723	Vmac	-0.710402039	0.035123721
ENSMUSG00000005320	Fgfr4	-0.709584165	0.031864013
ENSMUSG00000038776	Ephx1	-0.708907559	3.26E-47
ENSMUSG00000041261	Car8	-0.708638417	1.19E-12
ENSMUSG00000003623	Crot	-0.708354327	5.00E-23
ENSMUSG00000059316	Slc27a4	-0.707645467	0.004555358
ENSMUSG00000032841	Prr5l	-0.704884194	0.031901129
ENSMUSG00000032058	Ppp2r1b	-0.704813531	0.000785267
ENSMUSG00000051177	Plcb1	-0.704556514	0.005348814
ENSMUSG00000035105	Egln3	-0.702673344	3.74E-09
ENSMUSG00000040007	Bahd1	-0.70242391	9.63E-06
ENSMUSG00000040282	Cdin1	-0.701718474	0.009280473
ENSMUSG00000036894	Rap2b	-0.700304079	0.002590358
ENSMUSG00000098112	Bin2	-0.698870636	0.0454463
ENSMUSG00000041609	Bicdl1	-0.69739222	0.008222951
ENSMUSG00000028889	Yrdc	-0.696759065	0.044654497
ENSMUSG00000029153	Ociad2	-0.696148369	3.42E-65
ENSMUSG00000003662	Ciao1	-0.69561784	0.002468415
ENSMUSG00000041361	Myzap	-0.695554949	0.000204474
ENSMUSG00000036206	Sh3bp4	-0.695055331	1.73E-09
ENSMUSG00000028607	Cpt2	-0.694047557	1.21E-09
ENSMUSG00000053411	Cbx7	-0.692680872	0.001925498
ENSMUSG00000024610	Cd74	-0.692625025	6.71E-101
ENSMUSG00000030671	Pde3b	-0.692514325	0.008348828
ENSMUSG00000029557	Mrm2	-0.692419436	0.001665156
ENSMUSG00000068735	Trp53i11	-0.690867152	1.03E-07
ENSMUSG00000003752	Itpkc	-0.690808749	5.54E-05
ENSMUSG00000024312	Wdr46	-0.690395005	0.001207778
ENSMUSG00000031584	Gsr	-0.687714313	4.50E-96
ENSMUSG00000022114	Spry2	-0.687350168	0.000586423
ENSMUSG00000075415	Fnbp1	-0.68396647	0.027059533
ENSMUSG00000009630	Ppp2cb	-0.683269223	0.012888666
ENSMUSG00000059713	Rcan3	-0.682519913	0.005690076
ENSMUSG00000024731	Ms4a10	-0.681675464	5.87E-29
ENSMUSG00000039264	Gimap3	-0.680016272	0.005230782
ENSMUSG00000058392	Rrp1b	-0.678159949	1.32E-05
ENSMUSG00000025742	Prps2	-0.67444041	0.004146123
ENSMUSG00000031714	Gab1	-0.673935283	0.001807062
ENSMUSG00000074446	Defa23	-0.673126782	0.030492662
ENSMUSG00000068349	Gml	-0.671965766	4.54E-05
ENSMUSG00000054256	Msi1	-0.670446744	0.037914731
ENSMUSG00000038812	Trmt112	-0.67027383	0.000176188
ENSMUSG00000051853	Arf3	-0.669404584	5.35E-32

ENSMUSG00000034845	Plvap	0.795963993	9.41E-05
ENSMUSG00000023341	Mx2	0.795184538	0.002020061
ENSMUSG00000109841	E330011O21	0.795072411	0.00365429
ENSMUSG00000007038	Neu1	0.792842895	1.01E-34
ENSMUSG00000074342	I830077J02R	0.789289201	0.003762332
ENSMUSG00000040957	Cables1	0.789226219	8.32E-08
ENSMUSG00000040383	Aqr	0.788349265	2.87E-05
ENSMUSG00000007029	Vars	0.787140604	9.12E-09
ENSMUSG00000032666	1700025G04	0.782536558	5.45E-12
ENSMUSG00000021336	Slc17a4	0.782315166	3.85E-18
ENSMUSG00000068246	Apol9b	0.780154631	3.34E-07
ENSMUSG00000045871	Slitrk6	0.775622167	4.59E-15
ENSMUSG00000025429	Pstpip2	0.775513157	3.56E-06
ENSMUSG00000040506	Ambra1	0.775093814	0.020694027
ENSMUSG00000067297	Ifit1bl2	0.774301055	3.38E-17
ENSMUSG00000030374	Strn4	0.773946304	4.76E-05
ENSMUSG00000074754	Smim26	0.773129117	6.57E-07
ENSMUSG00000024242	Map4k3	0.772945857	0.000568118
ENSMUSG00000087651	1500009L16	0.772141758	2.38E-07
ENSMUSG00000067825	Pex26	0.770402917	0.004611191
ENSMUSG00000035578	Iqcg	0.769647321	0.001420242
ENSMUSG00000036256	Igfbp7	0.767906561	0.00243075
ENSMUSG00000008090	Fgfrl1	0.767293062	1.91E-08
ENSMUSG00000031075	Ano1	0.765811811	6.31E-05
ENSMUSG00000026447	Pik3c2b	0.761343545	1.03E-06
ENSMUSG00000019888	Mgat4c	0.7596893	3.51E-299
ENSMUSG00000043885	Slc36a4	0.758808641	0.007327965
ENSMUSG00000029763	Exoc4	0.756671298	1.58E-05
ENSMUSG00000034112	Atp2c2	0.756319668	2.05E-05
ENSMUSG00000030905	Crym	0.755032795	5.69E-26
ENSMUSG00000027490	E2f1	0.75117062	0.045454589
ENSMUSG00000024397	Aif1	0.75057251	0.002604148
ENSMUSG00000031355	Arhgap6	0.748636742	0.030717512
ENSMUSG00000090019	Gimap1	0.748154262	0.019765081
ENSMUSG00000033161	Atp1a1	0.746810711	0.003626641
ENSMUSG00000033769	Exoc6b	0.746743272	0.001240041
ENSMUSG00000036216	Leap2	0.746246697	9.54E-30
ENSMUSG00000102252	Snrpn	0.745989211	3.50E-19
ENSMUSG00000026827	Gpd2	0.744351519	1.82E-15
ENSMUSG00000022122	Ednrb	0.743747892	0.028830963
ENSMUSG00000040253	Gbp7	0.743722782	2.03E-08
ENSMUSG00000032715	Trib3	0.742440562	1.52E-08
ENSMUSG00000020277	Pfkl	0.741515399	0.002140552
ENSMUSG00000030314	Atg7	0.739322146	4.39E-05
ENSMUSG00000079018	Ly6c1	0.737561389	0.016400404
ENSMUSG00000063663	Brwd3	0.734582847	0.000913741
ENSMUSG00000004366	Sst	0.731554294	3.37E-45
ENSMUSG00000003528	Slc25a1	0.730909898	1.43E-05
ENSMUSG00000024124	Prss30	0.729049446	1.98E-29
ENSMUSG00000028037	Ifi44	0.72705537	0.001720194
ENSMUSG00000033581	Igf2bp2	0.724689506	2.07E-07
ENSMUSG00000019966	Kitl	0.72328891	1.32E-07
ENSMUSG00000057346	Apol9a	0.722859982	3.95E-21
ENSMUSG00000055978	Fut2	0.722393553	7.12E-20
ENSMUSG00000032788	Pdxk	0.720039578	3.84E-07
ENSMUSG00000028399	Ptprd	0.718990648	1.30E-19
ENSMUSG00000021539	Lect2	0.717466964	0.000688547
ENSMUSG00000034993	Vat1	0.717057587	1.71E-05
ENSMUSG00000085888	Gm12224	0.716843182	0.049320664
ENSMUSG00000022014	Epsti1	0.715641185	4.91E-06
ENSMUSG00000064177	Ghrl	0.715257764	7.28E-51
ENSMUSG00000024563	Smad2	0.712476358	0.009196271
ENSMUSG00000019893	Ros1	0.712403332	0.000764296
ENSMUSG00000047649	Polr1g	0.711487204	0.000423217
ENSMUSG00000040270	Bach2	0.71118955	2.82E-06
ENSMUSG00000005580	Adcy9	0.709228384	0.015891634
ENSMUSG00000033949	Trim36	0.708760739	0.00888391
ENSMUSG00000024187	Fam234a	0.708527792	0.004864242
ENSMUSG00000033952	Aspm	0.706156817	2.11E-08
ENSMUSG00000024639	Gnaq	0.706021507	2.53E-09
ENSMUSG00000049940	Pgrmc2	0.704293835	5.51E-42
ENSMUSG00000029814	Igf2bp3	0.704055458	5.40E-19
ENSMUSG00000031604	Msmo1	0.70341133	0.003216875
ENSMUSG00000022475	Hdac7	0.702882784	3.79E-06
ENSMUSG00000051579	Tceal8	0.701488436	1.16E-18
ENSMUSG00000001435	Col18a1	0.699227865	0.00018191
ENSMUSG00000040268	Plekha1	0.698229946	0.021350326
ENSMUSG00000006586	Runx1t1	0.696468296	0.007757073
ENSMUSG00000017929	B4galt5	0.693636238	6.45E-08
ENSMUSG00000047423	AI837181	0.691761748	0.022880965
ENSMUSG00000046688	Tifa	0.690829085	4.48E-13
ENSMUSG00000059743	Fdps	0.689849074	6.35E-08
ENSMUSG00000027086	Fastkd1	0.686945565	0.049544718
ENSMUSG00000026103	Gls	0.685407358	7.24E-37
ENSMUSG00000020359	Phykpl	0.684976466	0.000264189
ENSMUSG00000052415	Tchh	0.683145714	0.046978647
ENSMUSG00000023764	Sfi1	0.681502114	0.008063469
ENSMUSG00000120268	Gm41032	0.681120502	0.049024244
ENSMUSG00000006711	D130043K22	0.680925518	2.35E-05
ENSMUSG00000042121	Ssh1	0.680531997	0.025501597
ENSMUSG00000030834	Abcc6	0.679664933	6.86E-08
ENSMUSG00000038244	Mical2	0.677979868	1.20E-05
ENSMUSG00000056342	Usp34	0.677915999	5.10E-07
ENSMUSG00000037845	Fdxacb1	0.677885195	0.000948397
ENSMUSG00000034218	Atm	0.677619748	0.000478021
ENSMUSG00000048826	Dact2	0.677313181	0.006386617
ENSMUSG00000062064	Slc2a7	0.673115035	5.57E-06
ENSMUSG00000001700	Gramd2b	0.67239314	1.05E-74
ENSMUSG00000047562	Mmp10	0.670957625	3.35E-13
ENSMUSG00000020173	Cobl	0.669323843	0.002351147
ENSMUSG00000026360	Rgs2	0.668726279	4.74E-05
ENSMUSG00000031591	Asah1	0.668163746	0.008127929
ENSMUSG00000033538	Casp4	0.666886081	0.000130017
ENSMUSG00000021097	Clmn	0.666617052	0.0004193
ENSMUSG00000028005	Gucy1b1	0.666118985	0.049627004
ENSMUSG00000093930	Hmgcs1	0.665674778	7.27E-14
ENSMUSG00000093661	Eif4e3	0.66525706	7.26E-07
ENSMUSG00000066362	Rps13-ps1	0.665157968	1.86E-05
ENSMUSG00000040760	Appl1	0.664938273	1.99E-05
ENSMUSG00000015627	Gata5	0.663757071	1.06E-11
ENSMUSG00000042709	Atpaf2	0.66373963	0.002489915
ENSMUSG00000109695	Gm31166	0.663735043	0.000262578
ENSMUSG00000059498	Fcgr3	0.663396615	0.02700555
ENSMUSG00000020275	Rel	0.663166401	0.026320873
ENSMUSG00000062753	Smim29	0.662528823	0.013302226
ENSMUSG00000022614	Lmf2	0.661914896	0.017029317
ENSMUSG00000032766	Gng11	0.661343515	9.89E-06
ENSMUSG00000061689	Dlgap4	0.65998899	0.019329608
ENSMUSG00000089872	Rps6kc1	0.659773964	0.03463141
ENSMUSG00000058672	Tubb2a	0.657976935	1.24E-06
ENSMUSG00000039410	Prdm16	0.657909227	2.94E-19
ENSMUSG00000027463	Slc52a3	0.655545733	9.68E-133

ENSMUSG00000036298	Slc2a13	-0.696883815	0.003022027
ENSMUSG00000039781	Cep131	-0.694013281	0.032353312
ENSMUSG00000044468	Tent5c	-0.693513236	1.64E-05
ENSMUSG00000051978	Erich1	-0.693360121	0.012849896
ENSMUSG00000024376	Epb41l4a	-0.693320589	3.61E-13
ENSMUSG00000030088	Aldh1l1	-0.692847641	1.18E-07
ENSMUSG00000074345	Tnfaip8l3	-0.692725191	0.048922138
ENSMUSG00000003721	Insig2	-0.691449316	1.12E-09
ENSMUSG00000069808	Tlcd3a	-0.690017275	0.000377942
ENSMUSG00000074440	Defa3	-0.686957017	0.04521167
ENSMUSG00000074457	S100a16	-0.686350395	7.21E-10
ENSMUSG00000020897	Aurkb	-0.685433962	0.000655942
ENSMUSG00000003038	Hmgn2	-0.685178775	0.027484367
ENSMUSG00000076437	Selenoh	-0.684294669	0.046997051
ENSMUSG00000004730	Adgre1	-0.68407279	0.005430765
ENSMUSG00000022221	Ripk3	-0.683914482	7.90E-07
ENSMUSG00000041498	Kif14	-0.68357107	0.029741042
ENSMUSG00000034311	Kif4	-0.68352885	0.012708848
ENSMUSG00000032254	Kif23	-0.683220427	2.39E-06
ENSMUSG00000015943	Bola1	-0.681801637	0.002844741
ENSMUSG00000006398	Cdc20	-0.678287574	1.27E-05
ENSMUSG00000031591	Asah1	-0.677957503	0.008127929
ENSMUSG00000052997	Uba2	-0.676508857	0.034474675
ENSMUSG00000036832	Lpar3	-0.673007098	0.034275011
ENSMUSG00000038172	Ttc39b	-0.66956579	0.00017854
ENSMUSG00000068101	Cenpm	-0.669401607	0.024176019
ENSMUSG00000027330	Cdc25b	-0.662435293	0.002775596
ENSMUSG00000051627	H1f4	-0.66201943	0.00206194
ENSMUSG00000029446	Psph	-0.660948667	0.014346646
ENSMUSG00000019872	Smpdl3a	-0.660354789	0.000144801
ENSMUSG00000031490	Eif4ebp1	-0.659530502	0.000353571
ENSMUSG00000073705	Cenps	-0.657132912	0.012945209
ENSMUSG00000038155	Gstp2	-0.656149516	0.000812053
ENSMUSG00000054717	Hmgb2	-0.65514789	0.002053144
ENSMUSG00000000093	Tbx2	-0.654649979	0.047773101
ENSMUSG00000007594	Hapln4	-0.654528232	0.011454977
ENSMUSG00000027496	Aurka	-0.654299798	0.001659308
ENSMUSG00000001014	Icam4	-0.654138131	0.013351547
ENSMUSG00000037313	Tacc3	-0.653323874	0.001418447
ENSMUSG00000005686	Ampd3	-0.652023198	0.000250308
ENSMUSG00000064225	Paqr9	-0.65193738	1.23E-101
ENSMUSG00000047230	Cldn2	-0.651333878	1.81E-22
ENSMUSG00000028545	Bend5	-0.650325643	8.02E-08
ENSMUSG00000029279	Brdt	-0.650041273	0.003677289
ENSMUSG00000063364	3300002I08R	-0.649344326	0.026788152
ENSMUSG00000096696	Zfp960	-0.648623807	0.048902709
ENSMUSG00000087213	2810408I11R	-0.645679688	0.000223392
ENSMUSG00000020544	Cox11	-0.644100104	1.49E-09
ENSMUSG00000037845	Fdxacb1	-0.643871385	0.000948397
ENSMUSG00000074088	Snrnp40	-0.642358058	0.015063984
ENSMUSG00000070372	Capza1	-0.642014466	0.021198554
ENSMUSG00000060904	Arl1	-0.640341022	0.000107409
ENSMUSG00000049882	Vcpkmt	-0.639723524	1.45E-06
ENSMUSG00000002477	Snrpd1	-0.639548701	0.013633496
ENSMUSG00000029865	Sval1	-0.638929193	7.04E-11
ENSMUSG00000037580	Gch1	-0.635899866	1.07E-09
ENSMUSG00000038256	Bcl9	-0.635187893	3.82E-09
ENSMUSG00000030413	Pglyrp1	-0.634337128	5.48E-27
ENSMUSG00000049751	Rpl36al	-0.633022037	0.008609617
ENSMUSG00000038618	Rassf7	-0.632887686	0.000151277
ENSMUSG00000024480	Ap3s1	-0.628252132	0.001627625
ENSMUSG00000012443	Kif11	-0.627774882	0.023590132
ENSMUSG00000041827	Oasl1	-0.62773662	3.56E-18
ENSMUSG00000020572	Nampt	-0.627204134	2.68E-15
ENSMUSG00000062127	Cttnbp2nl	-0.626554856	3.34E-07
ENSMUSG00000040084	Bub1b	-0.624005716	0.010898782
ENSMUSG00000019857	Asf1a	-0.623167895	0.043950555
ENSMUSG00000067006	Serpinb5	-0.623042378	0.010991827
ENSMUSG00000019320	Noxo1	-0.622943147	2.20E-11
ENSMUSG00000071266	Zfp946	-0.621260129	0.030801814
ENSMUSG00000038751	Ptk6	-0.621031825	5.14E-33
ENSMUSG00000082211	Defa27	-0.620579454	0.000547211
ENSMUSG00000030560	Ctsc	-0.620122988	2.87E-08
ENSMUSG00000058799	Nap1l1	-0.620112048	6.61E-05
ENSMUSG00000060475	Wtap	-0.620063531	0.007698532
ENSMUSG00000019979	Apaf1	-0.618672242	2.02E-05
ENSMUSG00000058908	Pla2g2a	-0.617875673	9.86E-21
ENSMUSG00000043831	Lysmd4	-0.617698915	0.02245163
ENSMUSG00000034919	Ttc22	-0.617414217	0.014223719
ENSMUSG00000072571	Tmem253	-0.615679848	4.69E-12
ENSMUSG00000018554	Ybx2	-0.613843228	0.017814172
ENSMUSG00000027907	S100a11	-0.613101479	1.65E-07
ENSMUSG00000030659	Nucb2	-0.612738322	4.79E-22
ENSMUSG00000001025	S100a6	-0.612528088	0.001001923
ENSMUSG00000029153	Ociad2	-0.612228095	3.42E-65
ENSMUSG00000074909	Ranbp6	-0.611730072	0.0014401
ENSMUSG00000005481	Ddx39a	-0.610679009	0.005868545
ENSMUSG00000057541	Pus7	-0.609809091	0.040183167
ENSMUSG00000018548	Trim37	-0.609501237	5.45E-05
ENSMUSG00000039438	Ttc36	-0.609475984	0.006308072
ENSMUSG00000024339	Tap2	-0.608981743	6.98E-05
ENSMUSG00000060743	H3f3a	-0.608780585	0.012550795
ENSMUSG00000020914	Top2a	-0.606754869	1.61E-07
ENSMUSG00000042672	Dcst1	-0.606192974	0.005544042
ENSMUSG00000032263	Bckdhb	-0.605333784	0.030150408
ENSMUSG00000037593	Rskr	-0.604897655	0.008211049
ENSMUSG00000038879	Nipal2	-0.604509268	0.000689779
ENSMUSG00000073411	H2-D1	-0.6040636	6.39E-06
ENSMUSG00000059669	Taf1b	-0.603227032	0.01654663
ENSMUSG00000029149	Krtcap3	-0.602857519	2.91E-08
ENSMUSG00000028873	Cdca8	-0.602395104	4.63E-13
ENSMUSG00000030208	Emp1	-0.602123992	0.002900676
ENSMUSG00000049411	Tmem241	-0.599837757	0.0462941
ENSMUSG00000027296	Itpka	-0.59957323	0.009451871
ENSMUSG00000024660	Incenp	-0.599166163	3.48E-05
ENSMUSG00000037190	Cyb561d2	-0.599137621	0.015240534
ENSMUSG00000010660	Plcd1	-0.599073265	5.40E-08
ENSMUSG00000030793	Pycard	-0.597685093	0.000550378
ENSMUSG00000078974	Sec61g	-0.596664542	3.16E-05
ENSMUSG00000026566	Mpzl1	-0.594207345	0.004566459
ENSMUSG00000025025	Mxi1	-0.592309055	8.52E-06
ENSMUSG00000017607	Tns4	-0.592155337	9.60E-12
ENSMUSG00000023827	Agpat4	-0.591185105	3.00E-20
ENSMUSG00000022964	Tmem50b	-0.59088266	0.000104392
ENSMUSG00000002068	Ccne1	-0.590661005	0.019377542
ENSMUSG00000079508	Apoo	-0.590466084	0.00020292
ENSMUSG00000028542	Slc6a9	-0.589663783	4.38E-07
ENSMUSG00000026005	Rpe	-0.589315094	1.11E-15
ENSMUSG00000000295	Hddc2	-0.588801042	0.009036192
ENSMUSG00000003955	Fam162a	-0.588327009	2.46E-15
ENSMUSG00000006782	Cnp	-0.587873818	0.023438406
ENSMUSG00000031848	Lsm4	-0.587472343	0.000550084

ENSMUSG00000027340	Slc23a2	0.799933111	2.61E-07
ENSMUSG00000020901	Pik3r5	0.796899151	0.006518324
ENSMUSG00000073557	Ppp1r12b	0.793714277	0.013807886
ENSMUSG00000035769	Xylb	0.793142865	0.000151948
ENSMUSG00000096948	Gm4221	0.79276282	0.01653401
ENSMUSG00000026043	Col3a1	0.791630854	9.18E-08
ENSMUSG00000061906	Ugt2b38	0.791030635	0.000789259
ENSMUSG00000038496	Slc19a3	0.791017921	1.53E-10
ENSMUSG00000003053	Cyp2c29	0.79101758	6.37E-60
ENSMUSG00000024738	Pga5	0.791016881	0.001161853
ENSMUSG00000107681	4930528H21	0.791016669	0.002775874
ENSMUSG00000120391	Gm31308	0.791016393	9.79E-05
ENSMUSG00000031377	Bmx	0.791016223	0.002159169
ENSMUSG00000098037	Gm7765	0.791016146	0.039551596
ENSMUSG00000014543	Klra17	0.791015687	9.39E-07
ENSMUSG00000041012	Cmtm8	0.791014949	1.57E-06
ENSMUSG00000032315	Cyp1a1	0.791014494	1.23E-09
ENSMUSG00000069922	Ces3a	0.791014353	0.018563328
ENSMUSG00000040653	Ppp1r14c	0.791013936	0.01525031
ENSMUSG00000040181	Fmo1	0.791012449	0.018345782
ENSMUSG00000033847	Pla2g4c	0.790159422	0.000187951
ENSMUSG00000063317	Usp31	0.789955661	0.019597778
ENSMUSG00000040697	Dnajc16	0.788544989	0.020730953
ENSMUSG00000003166	Dgcr2	0.788113883	0.00162002
ENSMUSG00000095115	Itpripl2	0.78765425	6.80E-19
ENSMUSG00000021254	Gpatch2l	0.786333923	0.035853265
ENSMUSG00000046314	Stxbp6	0.784574434	0.023023893
ENSMUSG00000021209	Ppp4r4	0.784514708	1.40E-10
ENSMUSG00000025277	Abhd6	0.783666223	3.03E-19
ENSMUSG00000022620	Arsa	0.78365211	0.001997418
ENSMUSG00000030545	Pex11a	0.783527855	5.26E-14
ENSMUSG00000025515	Muc2	0.781154475	1.60E-33
ENSMUSG00000038384	Setd1b	0.780376862	8.56E-11
ENSMUSG00000033634	Nat8f2	0.778198447	2.69E-05
ENSMUSG00000004568	Arhgef18	0.777524387	0.006349284
ENSMUSG00000022010	Tsc22d1	0.775853725	1.25E-21
ENSMUSG00000024501	Dpysl3	0.775294286	5.40E-06
ENSMUSG00000098905	Zfp953	0.774690662	0.002088176
ENSMUSG00000031381	Piga	0.770382902	0.049395139
ENSMUSG00000030168	Adipor2	0.768874223	1.11E-10
ENSMUSG00000024921	Smarca2	0.767397778	6.70E-18
ENSMUSG00000038437	Mllt6	0.766990674	1.06E-06
ENSMUSG00000026103	Gls	0.765847643	7.24E-37
ENSMUSG00000043987	Cep164	0.765367512	0.00014853
ENSMUSG00000033917	Gde1	0.764435017	6.66E-26
ENSMUSG00000043252	Tmem64	0.764012185	4.25E-05
ENSMUSG00000040249	Lrp1	0.761864162	4.02E-09
ENSMUSG00000079516	Reg3a	0.761379811	1.14E-16
ENSMUSG00000037750	Fam222b	0.7593432	3.70E-09
ENSMUSG00000004151	Etv1	0.756709638	0.00149941
ENSMUSG00000052392	Acot4	0.756238731	0.002666373
ENSMUSG00000018796	Acsl1	0.754854432	9.13E-13
ENSMUSG00000014444	Piezo1	0.754779758	8.31E-06
ENSMUSG00000024132	Eci1	0.754115286	1.53E-07
ENSMUSG00000033769	Exoc6b	0.752600947	0.001240041
ENSMUSG00000002781	Tmem143	0.752563646	4.62E-06
ENSMUSG00000018849	Wwc1	0.750116909	1.75E-07
ENSMUSG00000037709	Fam13a	0.747708632	1.71E-68
ENSMUSG00000076435	Acsf2	0.747701886	0.014387553
ENSMUSG00000055730	Ces2a	0.746279026	7.68E-45
ENSMUSG00000035847	Ids	0.746046694	0.000120486
ENSMUSG00000027944	Hax1	0.744296396	0.002652016
ENSMUSG00000102252	Snrpn	0.743979248	3.50E-19
ENSMUSG00000060206	Zfp462	0.742916947	6.72E-05
ENSMUSG00000022747	St3gal6	0.742476422	2.38E-11
ENSMUSG00000036896	C1qc	0.741937439	0.009140038
ENSMUSG00000034993	Vat1	0.738824287	1.71E-05
ENSMUSG00000043391	2510009E07I	0.738673658	7.04E-07
ENSMUSG00000023452	Pisd	0.736068279	6.78E-07
ENSMUSG00000024843	Chka	0.734585477	1.79E-08
ENSMUSG00000040407	Akap9	0.73251052	3.91E-09
ENSMUSG00000020827	Mink1	0.732251787	9.43E-05
ENSMUSG00000026249	Serpine2	0.730399346	0.046749389
ENSMUSG00000069094	Pde7a	0.729376452	0.001970294
ENSMUSG00000030956	Fam53b	0.727635455	0.001920703
ENSMUSG00000040746	Rnf167	0.726800785	3.48E-11
ENSMUSG00000036246	Gmip	0.724423498	0.000113948
ENSMUSG00000071551	Akr1c19	0.724189163	7.89E-21
ENSMUSG00000021125	Arg2	0.723576994	6.46E-37
ENSMUSG00000035245	Eogt	0.72275683	0.00066737
ENSMUSG00000055866	Per2	0.721531431	0.001379975
ENSMUSG00000079293	Clec7a	0.721082024	0.02083372
ENSMUSG00000078616	Trim30c	0.717709272	0.001765034
ENSMUSG00000036585	Fgf1	0.71629137	0.006932516
ENSMUSG00000074024	4632427E13I	0.71611798	0.009298266
ENSMUSG00000110803	Gm20275	0.714747556	0.02085123
ENSMUSG00000020387	Jade2	0.712895752	0.016803978
ENSMUSG00000002028	Kmt2a	0.71068471	7.19E-16
ENSMUSG00000086784	Isoc2a	0.70924161	1.16E-10
ENSMUSG00000002222	Rmnd5a	0.707952791	0.000500317
ENSMUSG00000039713	Plekhg5	0.707097942	7.73E-06
ENSMUSG00000037005	Xpnpep2	0.706805245	3.72E-86
ENSMUSG00000034832	Tet3	0.703864624	5.30E-09
ENSMUSG00000078938	Syce3	0.703750567	1.71E-05
ENSMUSG00000030203	Dusp16	0.701116025	0.000159141
ENSMUSG00000022615	Tymp	0.699344096	1.04E-08
ENSMUSG00000030491	Tdrd12	0.698123472	0.017139466
ENSMUSG00000035372	1810055G02	0.698122581	5.70E-06
ENSMUSG00000043311	D17H6S53E	0.697949945	0.003957525
ENSMUSG00000066306	Numa1	0.696990735	2.78E-09
ENSMUSG00000017210	Med24	0.696680839	0.008168715
ENSMUSG00000029415	Sdad1	0.696552488	0.000728793
ENSMUSG00000052336	Cx3cr1	0.693669722	0.040301779
ENSMUSG00000070436	Serpinh1	0.693663119	0.002399427
ENSMUSG00000031958	Ldhd	0.693505781	1.14E-08
ENSMUSG00000042647	Acad12	0.692295851	2.39E-06
ENSMUSG00000022419	Deptor	0.690859741	1.10E-11
ENSMUSG00000021194	Chga	0.689793442	0.017645961
ENSMUSG00000083829	Gm2199	0.689690245	0.022913951
ENSMUSG00000057234	Mettl15	0.689332097	0.006675101
ENSMUSG00000003123	Lipe	0.689172867	0.001355807
ENSMUSG00000037989	Wnk2	0.688841504	0.000355004
ENSMUSG00000045975	C2cd2	0.688273504	4.50E-11
ENSMUSG00000024050	Wiz	0.687850306	0.002660214
ENSMUSG00000057719	Sh3rf2	0.687691851	5.71E-05
ENSMUSG00000036814	Slc6a20a	0.686807054	8.82E-12
ENSMUSG00000022376	Adcy8	0.685377586	8.23E-05
ENSMUSG00000051452	Gm11437	0.685338167	0.000287512
ENSMUSG00000019927	Ube2d1	0.685128304	0.001768159
ENSMUSG00000046329	Slc25a23	0.682921392	0.004528787
ENSMUSG00000074882	Cyp2c68	0.682750217	3.18E-22
ENSMUSG00000002985	Apoe	0.682181475	0.003728077

ENSMUSG00000033191	Tie1	-0.668833667	0.004349782
ENSMUSG00000020895	Tmem107	-0.668815575	0.009959751
ENSMUSG00000069456	Rdh16	-0.668243473	1.48E-06
ENSMUSG00000008540	Mgst1	-0.668184503	0.026480238
ENSMUSG00000028068	Iqgap3	-0.667894563	0.049470033
ENSMUSG00000021236	Entpd5	-0.664552796	4.66E-07
ENSMUSG00000083829	Gm2199	-0.663988236	0.022913951
ENSMUSG00000021365	Nedd9	-0.663102653	0.010912817
ENSMUSG00000024776	Stambpl1	-0.662390528	0.004048999
ENSMUSG00000003585	Sec14l2	-0.66207549	2.29E-15
ENSMUSG00000082211	Defa27	-0.659160277	0.000547211
ENSMUSG00000038151	Prdm1	-0.659046329	0.01152163
ENSMUSG00000019942	Cdk1	-0.659033083	0.00848051
ENSMUSG00000054598	9130230L23I	-0.657762605	1.08E-09
ENSMUSG00000028617	Lrrc42	-0.657106518	4.19E-16
ENSMUSG00000045725	Prr15	-0.65493629	7.30E-15
ENSMUSG00000028965	Tnfrsf9	-0.654087492	0.004505281
ENSMUSG00000073402	Gm8909	-0.653592782	1.79E-10
ENSMUSG00000005718	Tfap4	-0.653402324	0.032457048
ENSMUSG00000071715	Ncf4	-0.65331982	0.046749389
ENSMUSG00000031327	Chic1	-0.652097688	7.52E-14
ENSMUSG00000027456	Sdcbp2	-0.651132014	1.54E-09
ENSMUSG00000003038	Hmgn2	-0.650855434	0.027484367
ENSMUSG00000068227	Il2rb	-0.650782576	1.77E-05
ENSMUSG00000028383	Hsdl2	-0.649542063	1.10E-06
ENSMUSG00000048200	Cracr2b	-0.644374967	0.007127017
ENSMUSG00000025277	Abhd6	-0.64287348	3.03E-19
ENSMUSG00000002308	Cd320	-0.642029603	2.71E-05
ENSMUSG00000022469	Rapgef3	-0.641086967	0.030121144
ENSMUSG00000040139	9430038I01R	-0.638170574	4.16E-13
ENSMUSG00000025004	Cyp2c40	-0.635934228	0.04426896
ENSMUSG00000005628	Tmod4	-0.635082501	0.002974021
ENSMUSG00000033107	Rnf125	-0.633986385	2.79E-06
ENSMUSG00000032583	Mon1a	-0.633672207	0.021467857
ENSMUSG00000020377	Ltc4s	-0.633659762	9.63E-11
ENSMUSG00000113769	5033406O09	-0.633259697	1.84E-10
ENSMUSG00000036322	H2-Ea	-0.633195255	8.44E-52
ENSMUSG00000023909	Paqr4	-0.633132768	1.07E-24
ENSMUSG00000038209	Itln1	-0.632412728	4.92E-08
ENSMUSG00000058908	Pla2g2a	-0.630736204	9.86E-21
ENSMUSG00000020901	Pik3r5	-0.628137825	0.006518324
ENSMUSG00000048473	Sult6b2	-0.627793465	7.86E-21
ENSMUSG00000029650	Slc46a3	-0.62778617	0.0001302
ENSMUSG00000061848	Gm5805	-0.626610442	0.002739169
ENSMUSG00000025950	Idh1	-0.623726747	1.14E-27
ENSMUSG00000061959	Ces1e	-0.623108417	3.72E-45
ENSMUSG00000071711	Mpst	-0.62117275	3.75E-14
ENSMUSG00000024052	Lpin2	-0.619736094	2.55E-08
ENSMUSG00000018899	Irf1	-0.619568534	1.41E-21
ENSMUSG00000019989	Enpp3	-0.618141149	7.43E-76
ENSMUSG00000026879	Gsn	-0.617643292	1.35E-83
ENSMUSG00000045136	Tubb2b	-0.617348655	9.89E-13
ENSMUSG00000054364	Rhob	-0.616863768	4.71E-05
ENSMUSG00000021125	Arg2	-0.614684625	6.46E-37
ENSMUSG00000020405	Fabp6	-0.613077547	1.51E-180
ENSMUSG00000022389	Tef	-0.60874361	0.041982779
ENSMUSG00000018919	Tm4sf5	-0.606828708	9.23E-21
ENSMUSG00000052131	Akr1b7	-0.604555907	6.77E-23
ENSMUSG00000021200	Asb2	-0.603311134	0.001512495
ENSMUSG00000015837	Sqstm1	-0.601431064	7.52E-10
ENSMUSG00000056133	Unc93a2	-0.601398071	0.001767197
ENSMUSG00000027293	Ehd4	-0.60044398	4.54E-12
ENSMUSG00000034255	Arhgap27	-0.599673673	0.025516425
ENSMUSG00000022701	Ccdc191	-0.598213246	0.006088592
ENSMUSG00000071855	Ccdc112	-0.596093618	0.012469257
ENSMUSG00000046432	Bex3	-0.595264653	0.001516644
ENSMUSG00000029446	Psph	-0.595141408	0.014346646
ENSMUSG00000037552	Plekhg2	-0.594594553	7.85E-05
ENSMUSG00000036390	Gadd45a	-0.594471023	5.86E-06
ENSMUSG00000033955	Tnks1bp1	-0.59007338	5.95E-05
ENSMUSG00000029413	Naaa	-0.587470515	0.038682435
ENSMUSG00000030761	Myo7a	-0.585912222	0.029868586
ENSMUSG00000028600	Podn	-0.584216295	0.000104636
ENSMUSG00000024027	Glp1r	-0.583368869	0.035533028
ENSMUSG00000040432	Ltb4r2	-0.583299191	3.81E-06
ENSMUSG00000022231	Sema5a	-0.581791652	3.60E-15
ENSMUSG00000030662	Ipo5	-0.579347649	1.80E-05
ENSMUSG00000036027	Pou2af2	-0.57912574	4.41E-05
ENSMUSG00000057880	Abat	-0.578197472	6.81E-12
ENSMUSG00000038252	Ncapd2	-0.577922184	0.023700542
ENSMUSG00000038155	Gstp2	-0.576912573	0.000812053
ENSMUSG00000044991	Shld1	-0.576429215	0.001907013
ENSMUSG00000051452	Gm11437	-0.57333591	0.000287512
ENSMUSG00000024131	Slc3a1	-0.572825268	0.014875225
ENSMUSG00000060279	Ap2a1	-0.572770788	0.034904457
ENSMUSG00000022844	Pdia5	-0.572011663	0.00340953
ENSMUSG00000022377	Asap1	-0.571681669	0.010334594
ENSMUSG00000030816	Rnf40	-0.570522485	0.000355903
ENSMUSG00000050213	Snip1	-0.570515979	0.031761239
ENSMUSG00000024507	Hsd17b4	-0.569642423	2.08E-23
ENSMUSG00000027439	Gzf1	-0.567246943	4.48E-08
ENSMUSG00000010307	Tmem86a	-0.567060282	0.023835012
ENSMUSG00000042367	Gjb3	-0.566736967	2.69E-25
ENSMUSG00000020261	Slc36a1	-0.563878918	8.44E-07
ENSMUSG00000022193	Psmb5	-0.563302693	0.006524472
ENSMUSG00000046562	Unc119b	-0.562874527	0.000786522
ENSMUSG00000042195	Slc35f2	-0.5628069	0.002738082
ENSMUSG00000068762	Gstm6	-0.562338354	2.20E-123
ENSMUSG00000001865	Cpa3	-0.562290826	0.030584039
ENSMUSG00000032712	Resf1	-0.561869148	8.14E-18
ENSMUSG00000048572	Tmem252	-0.561562423	5.74E-10
ENSMUSG00000022479	Vdr	-0.561400123	1.35E-10
ENSMUSG00000093805	Gal3st2b	-0.560158886	9.33E-07
ENSMUSG00000097415	AU020206	-0.559161655	0.003958051
ENSMUSG00000021975	Ints9	-0.559136989	0.04118817
ENSMUSG00000027359	Slc27a2	-0.559061193	1.01E-46
ENSMUSG00000000078	Klf6	-0.558796478	9.95E-07
ENSMUSG00000017307	Acot8	-0.557115264	0.001388687
ENSMUSG00000051510	Mafg	-0.557093892	0.000235616
ENSMUSG00000032397	Tipin	-0.555506645	0.013715055
ENSMUSG00000033318	Gstt2	-0.55470854	3.78E-09
ENSMUSG00000079440	Alpi	-0.554391369	1.89E-11
ENSMUSG00000020282	Rhbdf1	-0.552272993	0.00065325
ENSMUSG00000005470	Asf1b	-0.551239667	0.015758577
ENSMUSG00000025203	Scd2	-0.550263829	1.32E-93
ENSMUSG00000037145	Lypd8l	-0.548017935	2.31E-83
ENSMUSG00000023045	Soat2	-0.546120903	0.010575628
ENSMUSG00000074437	Defa29	-0.544345339	6.98E-17
ENSMUSG00000002332	Dhrs1	-0.54337699	3.56E-139
ENSMUSG00000029578	Wipi2	-0.542467212	0.001377849
ENSMUSG00000038525	Armc10	-0.541838584	0.035668392
ENSMUSG00000038372	Gmds	-0.541342914	2.29E-29

ENSMUSG00000016409	Nkap	0.655449176	0.048388236
ENSMUSG00000025579	Gaa	0.655379077	0.006939633
ENSMUSG00000052336	Cx3cr1	0.654421102	0.040301779
ENSMUSG00000000732	Icosl	0.654250879	0.038753168
ENSMUSG00000056493	Foxk1	0.652250115	4.27E-06
ENSMUSG00000048874	Phf3	0.650078077	4.22E-09
ENSMUSG00000017679	Ttpal	0.650059862	0.000654262
ENSMUSG00000029188	Slc34a2	0.650028255	7.32E-69
ENSMUSG00000036427	Gpi1	0.649668587	9.20E-12
ENSMUSG00000033434	Gtpbp6	0.648683049	0.037050981
ENSMUSG00000045690	Wdr89	0.64822786	0.012218937
ENSMUSG00000052212	Cd177	0.648159335	2.59E-08
ENSMUSG00000021273	Fdft1	0.647294856	2.13E-07
ENSMUSG00000027198	Ext2	0.643103921	0.001532551
ENSMUSG00000032294	Pkm	0.642922042	8.92E-52
ENSMUSG00000039047	Pigk	0.642483466	0.027252551
ENSMUSG00000005958	Ephb3	0.641233184	2.22E-11
ENSMUSG00000031983	2310022B05	0.640828214	0.017128529
ENSMUSG00000026354	Lct	0.639888004	3.56E-12
ENSMUSG00000111296	2010001M07	0.639811607	2.81E-77
ENSMUSG00000074405	Zfp865	0.63929974	0.01187514
ENSMUSG00000072082	Ccnf	0.638725212	0.00695745
ENSMUSG00000055204	Ankrd17	0.634369845	7.48E-09
ENSMUSG00000047843	Bri3	0.633874611	0.001754909
ENSMUSG00000030972	Acsm5	0.633542868	5.08E-07
ENSMUSG00000066441	Rdh11	0.633346092	1.84E-17
ENSMUSG00000004665	Cnn2	0.633063332	0.000215042
ENSMUSG00000080727	C920021L13	0.631885812	0.023818348
ENSMUSG00000029804	Herc3	0.630936416	0.00924354
ENSMUSG00000042284	Itga1	0.630920619	0.002844741
ENSMUSG00000079293	Clec7a	0.630560561	0.02083372
ENSMUSG00000009035	Tmem184b	0.630058612	0.023198941
ENSMUSG00000026131	Dst	0.630010965	0.032435817
ENSMUSG00000044906	4930503L19I	0.628827315	0.034768495
ENSMUSG00000050188	Lsm10	0.628594057	0.008309343
ENSMUSG00000028654	Mycl	0.627875394	0.003419422
ENSMUSG00000009905	Kdsr	0.626758468	3.72E-05
ENSMUSG00000020183	Cpm	0.626551007	0.000506655
ENSMUSG00000006435	Neurl1a	0.626383035	4.11E-05
ENSMUSG00000046223	Plaur	0.625082334	0.032968492
ENSMUSG00000057103	Nat8f1	0.622762331	4.04E-16
ENSMUSG00000035064	Eef2k	0.622241049	2.29E-09
ENSMUSG00000041895	Wipi1	0.621335576	0.007467407
ENSMUSG00000070034	Sp110	0.620207561	5.45E-05
ENSMUSG00000085421	4732490B19	0.619954948	0.01707099
ENSMUSG00000030894	Tpp1	0.619596895	0.000651315
ENSMUSG00000021221	Dpf3	0.619492474	0.000277657
ENSMUSG00000034285	Nipsnap1	0.618117178	9.80E-32
ENSMUSG00000022292	Rrm2b	0.617864861	0.023642109
ENSMUSG00000036333	Kidins220	0.616760627	0.001527026
ENSMUSG00000018678	Sp2	0.614774071	0.046713742
ENSMUSG00000031644	Nek1	0.614486999	0.002937568
ENSMUSG00000029009	Mthfr	0.614077591	0.033519392
ENSMUSG00000054932	Afp	0.612810456	1.15E-82
ENSMUSG00000037646	Vps13b	0.612163384	8.28E-07
ENSMUSG00000040721	Zfhx2	0.611502462	0.011847783
ENSMUSG00000020140	Lgr5	0.610581038	1.22E-05
ENSMUSG00000021990	Spata13	0.61045351	0.017029317
ENSMUSG00000017978	Cadps2	0.610444683	0.028450328
ENSMUSG00000036591	Arhgap21	0.610089218	0.041397351
ENSMUSG00000022519	Srl	0.609761533	0.009523928
ENSMUSG00000047417	Rexo1	0.608714509	2.11E-07
ENSMUSG00000026832	Cytip	0.607952178	4.42E-08
ENSMUSG00000022263	Trio	0.607628162	9.32E-19
ENSMUSG00000015745	Plekho1	0.604122384	0.016410247
ENSMUSG00000062995	Ica1	0.603934472	8.90E-05
ENSMUSG00000040795	Iqcc	0.603642791	0.017718245
ENSMUSG00000022351	Sqle	0.602871665	8.17E-20
ENSMUSG00000021508	Cxcl14	0.602241819	0.04911688
ENSMUSG00000058317	Ube2e2	0.601083778	0.020360003
ENSMUSG00000033732	Sf3b3	0.601007252	0.023833086
ENSMUSG00000031765	Mt1	0.600637023	6.60E-05
ENSMUSG00000029505	Ep400	0.600111175	1.74E-06
ENSMUSG00000046718	Bst2	0.599026349	7.26E-07
ENSMUSG00000072974	Gm4787	0.598881804	0.019982045
ENSMUSG00000053841	Txlna	0.598204032	8.60E-05
ENSMUSG00000064325	Hhip	0.597628223	1.57E-06
ENSMUSG00000022270	Retreg1	0.597290672	5.66E-18
ENSMUSG00000020717	Pecam1	0.597110806	0.015247343
ENSMUSG00000051323	Pcdh19	0.595361627	3.56E-14
ENSMUSG00000034317	Trim59	0.593547959	0.022447547
ENSMUSG00000027952	Pmvk	0.593198793	0.022019075
ENSMUSG00000030655	Smg1	0.591669736	0.030362379
ENSMUSG00000061755	Bod1l	0.590878242	5.82E-07
ENSMUSG00000026043	Col3a1	0.59057044	9.18E-08
ENSMUSG00000001211	Agpat3	0.590239407	1.57E-17
ENSMUSG00000021708	Rasgrf2	0.589994638	0.016803978
ENSMUSG00000024079	Eif2ak2	0.589425832	0.04241373
ENSMUSG00000004730	Adgre1	0.589268949	0.005430765
ENSMUSG00000038331	Satb2	0.589175591	1.71E-14
ENSMUSG00000043822	Adamtsl5	0.589090252	0.023672623
ENSMUSG00000025280	Polr3a	0.588835148	0.000337974
ENSMUSG00000096910	Zfp955b	0.587048893	0.000625905
ENSMUSG00000006717	Acot13	0.585409321	7.49E-21
ENSMUSG00000042423	Fbrs	0.585372919	0.005930344
ENSMUSG00000057672	Pkn1	0.584536332	0.001547124
ENSMUSG00000022057	Adamdec1	0.582648148	7.61E-06
ENSMUSG00000036572	Upf3b	0.582262071	2.82E-06
ENSMUSG00000033781	Asb13	0.580981395	1.47E-17
ENSMUSG00000022286	Grhl2	0.579935836	0.031809287
ENSMUSG00000085126	Gm12589	0.579504369	3.85E-13
ENSMUSG00000042096	Dao	0.578191857	4.44E-42
ENSMUSG00000036503	Rnf13	0.577997424	8.07E-05
ENSMUSG00000013846	St3gal1	0.577777113	6.71E-05
ENSMUSG00000021240	Abcd4	0.577717027	0.00046332
ENSMUSG00000040189	Odad1	0.577381941	1.86E-20
ENSMUSG00000005373	Mlxipl	0.577050278	2.11E-11
ENSMUSG00000025153	Fasn	0.576531868	1.53E-10
ENSMUSG00000038534	Osbpl7	0.575734342	1.57E-10
ENSMUSG00000034430	Zxdc	0.575670434	0.006563997
ENSMUSG00000038143	Stox2	0.574801041	0.036211525
ENSMUSG00000033355	Rtp4	0.57475587	7.75E-10
ENSMUSG00000052534	Pbx1	0.574626522	9.62E-20
ENSMUSG00000074218	Cox7a1	0.574513509	2.01E-45
ENSMUSG00000017830	Dhx58	0.574435052	7.42E-19
ENSMUSG00000030516	Tjp1	0.573674323	0.000102727
ENSMUSG00000069045	Ddx3y	0.573069954	1.16E-06
ENSMUSG00000041642	Kif21b	0.57242156	0.042995412
ENSMUSG00000045216	Hs6st1	0.571828514	1.27E-08
ENSMUSG00000022817	Itgb5	0.571564507	0.00228574
ENSMUSG00000036905	C1qb	0.571262686	0.03229553
ENSMUSG00000034930	Rtkn	0.570160118	0.005875082

ENSMUSG00000026622	Nek2	-0.587077142	0.006035309
ENSMUSG00000028567	Txndc12	-0.586537665	1.64E-05
ENSMUSG00000059182	Skap2	-0.586288986	7.88E-11
ENSMUSG00000050912	Tmem123	-0.583805659	0.004567805
ENSMUSG00000026126	Ptpn18	-0.583781662	6.38E-18
ENSMUSG00000120425	Ndufb4b	-0.581653471	1.98E-06
ENSMUSG00000029482	Aacs	-0.580447349	1.03E-11
ENSMUSG00000026281	Dtymk	-0.580233159	0.03598728
ENSMUSG00000073987	Ggh	-0.580142467	0.001737258
ENSMUSG00000001761	Smo	-0.579310098	0.035189454
ENSMUSG00000050982	Apol10a	-0.577089866	0.001991487
ENSMUSG00000079418	Atg4a	-0.576905649	3.05E-05
ENSMUSG00000022003	Slc25a30	-0.576892864	0.001873809
ENSMUSG00000056209	Npm3	-0.576694684	0.001510097
ENSMUSG00000022584	Ly6c2	-0.575279138	6.26E-08
ENSMUSG00000024030	Abcg1	-0.574982486	0.005278011
ENSMUSG00000027624	Epb41l1	-0.572600223	2.56E-12
ENSMUSG00000027018	Hat1	-0.571758387	0.001988759
ENSMUSG00000058618	Defa39	-0.571694155	0.004630545
ENSMUSG00000036768	Kif15	-0.570467716	0.000488647
ENSMUSG00000015023	Ddx19a	-0.569837125	0.018174199
ENSMUSG00000053581	Zfand2a	-0.569591555	0.021872205
ENSMUSG00000037266	Rsrp1	-0.568701964	7.36E-07
ENSMUSG00000001228	Uhrf1	-0.56821465	0.000373215
ENSMUSG00000001517	Foxm1	-0.567970707	0.000304724
ENSMUSG00000020843	Timm22	-0.567143071	0.012753338
ENSMUSG00000047466	8030462N17	-0.566218376	0.044373609
ENSMUSG00000029804	Herc3	-0.565772773	0.00924354
ENSMUSG00000051674	Dcun1d4	-0.565486344	4.35E-05
ENSMUSG00000074802	Gas2l3	-0.5649508	0.049244394
ENSMUSG00000044558	Ndrg2	-0.564280682	2.60E-08
ENSMUSG00000015083	C8g	-0.563039925	2.96E-09
ENSMUSG00000069899	Gm12166	-0.562657882	0.000321563
ENSMUSG00000044763	Trmt10c	-0.562633301	0.007005825
ENSMUSG00000031827	Cotl1	-0.562187442	0.014262929
ENSMUSG00000026418	Tnni1	-0.561750833	0.000172223
ENSMUSG00000036777	Anln	-0.560815682	0.003298138
ENSMUSG00000022297	Dbf4	-0.560769978	0.004317406
ENSMUSG00000055200	Sertad3	-0.559719963	0.00747671
ENSMUSG00000020405	Fabp6	-0.559084982	1.51E-180
ENSMUSG00000078949	R3hdml	-0.557608293	0.039640743
ENSMUSG00000037679	Inf2	-0.556974993	0.005563449
ENSMUSG00000020581	Agr2	-0.556896963	8.43E-08
ENSMUSG00000041642	Kif21b	-0.556588662	0.042995412
ENSMUSG00000026614	Slc30a10	-0.556491894	1.80E-07
ENSMUSG00000087651	1500009L16	-0.554402872	2.38E-07
ENSMUSG00000022469	Rapgef3	-0.551966451	0.030121144
ENSMUSG00000039994	Timeless	-0.551957641	0.000881497
ENSMUSG00000024902	Mrpl11	-0.551814146	4.10E-06
ENSMUSG00000022013	Dnajc15	-0.550536207	1.84E-10
ENSMUSG00000022216	Psme1	-0.549598896	3.68E-11
ENSMUSG00000079114	Defa42	-0.549502573	7.51E-06
ENSMUSG00000020492	Ska2	-0.549429212	0.010580194
ENSMUSG00000038633	Degs1	-0.547858473	1.96E-14
ENSMUSG00000035547	Capn5	-0.547119376	2.44E-05
ENSMUSG00000035212	Leprot	-0.546602354	0.044354991
ENSMUSG00000018983	E2f2	-0.54563395	4.46E-11
ENSMUSG00000064427	Gm22748	-0.545470585	8.63E-20
ENSMUSG00000037465	Klf10	-0.544263197	0.034550032
ENSMUSG00000026828	Galnt5	-0.543783447	0.000307404
ENSMUSG00000044906	4930503L19	-0.542560428	0.034768495
ENSMUSG00000027835	Pdcd10	-0.542106035	3.71E-09
ENSMUSG00000037661	Gpr160	-0.539942298	0.007350991
ENSMUSG00000001525	Tubb5	-0.536692425	3.68E-09
ENSMUSG00000057561	Eif1a	-0.53615682	0.001266079
ENSMUSG00000003526	Prodh	-0.535667081	4.29E-06
ENSMUSG00000078185	Chml	-0.53556501	0.005058815
ENSMUSG00000021774	Ube2e1	-0.534783181	0.000327924
ENSMUSG00000041959	S100a10	-0.533288861	0.003982743
ENSMUSG00000005410	Mcm5	-0.532286571	0.002920749
ENSMUSG00000030695	Aldoa	-0.531868449	5.04E-08
ENSMUSG00000028268	Gbp3	-0.531702133	4.13E-06
ENSMUSG00000068246	Apol9b	-0.531182852	3.34E-07
ENSMUSG00000078713	Tomm5	-0.529394659	0.000151342
ENSMUSG00000078612	Fyb2	-0.52724814	1.37E-06
ENSMUSG00000030603	Psmc4	-0.526160223	0.000225596
ENSMUSG00000027524	Edn3	-0.525374569	6.03E-28
ENSMUSG00000019082	Slc25a22	-0.524978378	6.08E-13
ENSMUSG00000030029	Lrig1	-0.522817163	5.93E-06
ENSMUSG00000024151	Msh2	-0.522812839	0.044569672
ENSMUSG00000032067	Pts	-0.522557545	0.006201742
ENSMUSG00000004562	Arhgef40	-0.522516213	0.01323804
ENSMUSG00000073792	Alg6	-0.522337458	0.017174658
ENSMUSG00000028484	Psip1	-0.521521734	0.000345612
ENSMUSG00000015749	Anp32e	-0.521403221	1.82E-05
ENSMUSG00000032091	Tmprss4	-0.520937947	0.000171221
ENSMUSG00000029430	Ran	-0.520876275	0.016790317
ENSMUSG00000030867	Plk1	-0.520389692	3.61E-09
ENSMUSG00000045103	Dmd	-0.519428868	0.006240103
ENSMUSG00000024539	Ptpn2	-0.518399742	0.005899217
ENSMUSG00000025076	Casp7	-0.517439548	2.44E-08
ENSMUSG00000041119	Pde9a	-0.51682455	0.000368335
ENSMUSG00000069633	Pex11g	-0.516213111	1.04E-05
ENSMUSG00000030978	Rrm1	-0.515171225	0.000293034
ENSMUSG00000023951	Vegfa	-0.513322556	0.000529776
ENSMUSG00000001095	Slc13a2	-0.513208741	1.56E-44
ENSMUSG00000048911	Rnf24	-0.512530591	0.00083789
ENSMUSG00000036983	Tfb1m	-0.511771376	0.017476017
ENSMUSG00000032182	Yipf2	-0.511527825	0.002334584
ENSMUSG00000030056	Isy1	-0.510547595	0.023618942
ENSMUSG00000068749	Psma5	-0.509313888	0.004968592
ENSMUSG00000022358	Fbxo32	-0.509302692	1.04E-24
ENSMUSG00000033467	Crlf2	-0.5092295	4.52E-05
ENSMUSG00000028334	Nans	-0.509201929	0.000107609
ENSMUSG00000040675	Mthfd1l	-0.508649215	0.004026717
ENSMUSG00000031422	Morf4l2	-0.506424998	8.27E-08
ENSMUSG00000117679	Apbb3	-0.505741169	0.031183314
ENSMUSG00000069662	Marcks	-0.505256356	1.75E-15
ENSMUSG00000063904	Dpp3	-0.504948817	0.000156903
ENSMUSG00000030751	Psma1	-0.504017897	0.000109699
ENSMUSG00000024925	Rnaseh2c	-0.501041058	0.007549987
ENSMUSG00000037279	Ovol2	-0.500841269	0.000473195
ENSMUSG00000046909	Tefm	-0.500682367	0.02678648
ENSMUSG00000024776	Stambpl1	-0.500641174	0.004048999
ENSMUSG00000041736	Tspo	-0.500465115	1.64E-07
ENSMUSG00000058290	Espl1	-0.499980297	0.010685262
ENSMUSG00000043190	Rfesd	-0.499638459	0.00015023
ENSMUSG00000037991	Rmi2	-0.499465239	0.018055568
ENSMUSG00000090258	Churc1	-0.497794042	0.035123721
ENSMUSG00000064120	Mocs1	-0.497090247	0.014551215
ENSMUSG00000106951	5930430L01	-0.496994252	0.010453859
ENSMUSG00000061232	H2-K1	-0.496663881	0.031257952

ENSMUSG00000020261	Slc36a1	0.681781812	8.44E-07
ENSMUSG00000023057	Fabp2	0.681494973	0.000445521
ENSMUSG00000060935	Tmem263	0.680802152	6.44E-19
ENSMUSG00000068551	Zfp467	0.680540506	2.08E-11
ENSMUSG00000040943	Tet2	0.679343112	0.00032707
ENSMUSG00000018820	Zfyve27	0.679299002	0.003153198
ENSMUSG00000042035	Igsf3	0.67736031	0.000266846
ENSMUSG00000026489	Coq8a	0.674963619	2.01E-05
ENSMUSG00000033854	Kcnk10	0.674260456	1.45E-18
ENSMUSG00000033313	Fbxl8	0.674024088	0.011062313
ENSMUSG00000036138	Acaa1a	0.673559664	6.47E-11
ENSMUSG00000121492	6820431F20	0.673151256	0.007786579
ENSMUSG00000015627	Gata5	0.672293565	1.06E-11
ENSMUSG00000042292	Mrtfa	0.670892096	0.02715623
ENSMUSG00000036887	C1qa	0.670173196	7.31E-06
ENSMUSG00000057604	Lmcd1	0.666873173	1.18E-10
ENSMUSG00000041415	Dicer1	0.666565963	0.003977245
ENSMUSG00000059555	Tor4a	0.665470175	0.03167935
ENSMUSG00000039568	Ubald1	0.664755008	0.004669741
ENSMUSG00000020121	Srgap1	0.664404087	0.001707449
ENSMUSG00000031886	Ces2e	0.662874491	1.40E-24
ENSMUSG00000021208	Ifi27l2b	0.662473393	2.86E-19
ENSMUSG00000020458	Rtn4	0.660665812	2.55E-25
ENSMUSG00000026389	Steap3	0.660604825	0.000666661
ENSMUSG00000054226	Tprkb	0.660477999	8.50E-11
ENSMUSG00000036368	Rmdn2	0.659211863	0.001019291
ENSMUSG00000045282	Tmem86b	0.658373885	3.66E-09
ENSMUSG00000040913	Fbxw4	0.657887795	0.015824669
ENSMUSG00000003233	Dvl3	0.657655812	0.044533113
ENSMUSG00000068323	Slc4a5	0.657546639	4.31E-17
ENSMUSG00000031575	Ash2l	0.65695327	0.000116542
ENSMUSG00000029050	Ski	0.656424532	5.07E-16
ENSMUSG00000000861	Bcl11a	0.656308421	2.65E-05
ENSMUSG00000089774	Slc5a3	0.656284614	0.000248451
ENSMUSG00000044857	Lemd2	0.655045261	0.003597092
ENSMUSG00000097392	Thoc2l	0.654491901	5.76E-07
ENSMUSG00000024621	Csf1r	0.653315977	0.00447197
ENSMUSG00000040820	Hlcs	0.653258547	0.022315015
ENSMUSG00000003119	Cdk12	0.652635096	3.75E-05
ENSMUSG00000025091	Pnliprp2	0.652030247	6.98E-15
ENSMUSG00000001700	Gramd2b	0.651601885	1.05E-74
ENSMUSG00000030201	Lrp6	0.651150118	1.61E-08
ENSMUSG00000036503	Rnf13	0.65046488	8.07E-05
ENSMUSG00000074405	Zfp865	0.650062834	0.01187514
ENSMUSG00000040997	Abhd4	0.649760028	0.000163143
ENSMUSG00000005533	Igf1r	0.649098426	2.40E-13
ENSMUSG00000120268	Gm41032	0.648905744	0.049024244
ENSMUSG00000055225	Plekha8	0.647661263	5.23E-05
ENSMUSG00000053004	Hrh1	0.646329777	0.001080766
ENSMUSG00000068245	Phf11d	0.645394399	3.20E-10
ENSMUSG00000022270	Retreg1	0.64279332	5.66E-18
ENSMUSG00000061740	Cyp2d22	0.642698774	8.80E-05
ENSMUSG00000042308	Setd1a	0.642690885	0.0410229
ENSMUSG00000045598	Zfp553	0.642389213	0.002717478
ENSMUSG00000054843	Atrnl1	0.641233343	0.000357761
ENSMUSG00000089960	Ugt1a1	0.639867299	5.94E-05
ENSMUSG00000022265	Ank	0.6386632	2.17E-11
ENSMUSG00000028517	Plpp3	0.63860228	0.002088176
ENSMUSG00000022634	Yaf2	0.637942718	0.008655707
ENSMUSG00000038418	Egr1	0.636487397	1.74E-09
ENSMUSG00000068115	Ninl	0.636463966	0.013006961
ENSMUSG00000005373	Mlxipl	0.636123818	2.11E-11
ENSMUSG00000027890	Gstm4	0.635124131	2.83E-30
ENSMUSG00000040274	Cdk6	0.63497297	1.57E-06
ENSMUSG00000061143	Maml3	0.634967611	0.00059653
ENSMUSG00000083019	Gm6222	0.633850806	0.000247227
ENSMUSG00000057069	Ero1b	0.632999368	0.027736522
ENSMUSG00000014771	Pdcd2	0.631842543	0.032537862
ENSMUSG00000106352	5033403H07	0.631340981	0.000180467
ENSMUSG00000057982	Zfp809	0.631068718	0.039896575
ENSMUSG00000073468	Sft2d1	0.63034437	0.00817157
ENSMUSG00000021221	Dpf3	0.629297885	0.000277667
ENSMUSG00000022651	Retnlg	0.628899213	0.017329148
ENSMUSG00000026692	Fmo4	0.628850593	0.005135732
ENSMUSG00000044783	A730008H23	0.628142943	0.006499982
ENSMUSG00000025059	Gk	0.628019681	0.000883349
ENSMUSG00000021336	Slc17a4	0.626877466	3.85E-18
ENSMUSG00000048874	Phf3	0.626488015	4.22E-09
ENSMUSG00000024958	Gpr137	0.626404297	0.001988759
ENSMUSG00000023913	Pla2g7	0.626253336	0.002620755
ENSMUSG00000022305	Lrp12	0.624223583	6.74E-09
ENSMUSG00000020182	Ddc	0.623436342	2.65E-38
ENSMUSG00000044367	Slc16a13	0.623184326	9.38E-05
ENSMUSG00000033943	Mga	0.622737633	0.017890285
ENSMUSG00000102752	Gm7694	0.622044073	9.12E-16
ENSMUSG00000021972	Hmbox1	0.62189958	0.048750063
ENSMUSG00000034245	Hdac11	0.621540781	1.89E-05
ENSMUSG00000026193	Fn1	0.621020628	2.57E-05
ENSMUSG00000020777	Acox1	0.620964962	8.92E-06
ENSMUSG00000020097	Sgpl1	0.620393287	0.011283469
ENSMUSG00000097571	Jpx	0.620317337	0.017353324
ENSMUSG00000039844	Rapgef1	0.619996495	0.000657803
ENSMUSG00000054676	1600014C10	0.619848853	1.62E-18
ENSMUSG00000021978	Extl3	0.618589446	0.005430103
ENSMUSG00000018574	Acadvl	0.618121068	1.94E-10
ENSMUSG00000046982	Tshz1	0.616842818	1.70E-07
ENSMUSG00000024187	Fam234a	0.616715938	0.004864242
ENSMUSG00000020427	Igfbp3	0.615646897	1.93E-07
ENSMUSG00000006435	Neurl1a	0.615618492	4.11E-05
ENSMUSG00000038175	Mylip	0.614381507	8.42E-17
ENSMUSG00000090150	Acad11	0.614321871	3.53E-12
ENSMUSG00000024424	Ttc39c	0.612860228	2.75E-21
ENSMUSG00000028603	Scp2	0.611221451	3.48E-10
ENSMUSG00000024270	Lpcat3	0.609342675	7.91E-12
ENSMUSG00000079440	Alpi	0.608466393	1.89E-11
ENSMUSG00000003623	Crot	0.607913144	5.00E-23
ENSMUSG00000055912	Tmem150a	0.607711795	0.000962376
ENSMUSG00000094840	Muc3a	0.6050466	1.93E-05
ENSMUSG00000045287	Rtn4rl1	0.604777969	5.24E-22
ENSMUSG00000074647	Fam83c	0.604739316	0.000459844
ENSMUSG00000038387	Rras	0.604100045	0.011325008
ENSMUSG00000021140	Pcnx	0.603869428	0.007292993
ENSMUSG00000041779	Tram2	0.603484106	0.000584974
ENSMUSG00000039349	C130074G19	0.603467368	5.31E-12
ENSMUSG00000017843	Ppp2r5c	0.602761734	1.72E-08
ENSMUSG00000040151	Hs2st1	0.601900297	0.002850089
ENSMUSG00000027253	Lrp4	0.601529442	0.000582014
ENSMUSG00000024254	Abcg8	0.600491517	0.003399773
ENSMUSG00000045482	Trrap	0.600343157	0.014730004
ENSMUSG00000029161	Cgref1	0.600290777	5.38E-51
ENSMUSG00000045757	Zfp764	0.59974845	0.048021844
ENSMUSG00000074093	Svip	0.598095075	1.06E-09

ENSMUSG00000029201	Ugdh	-0.54113358	4.35E-46
ENSMUSG00000028937	Acot7	-0.54082825	6.74E-09
ENSMUSG00000017144	Rnd3	-0.538627754	0.001963708
ENSMUSG00000077704	Snord89	-0.538504487	1.37E-17
ENSMUSG00000042672	Dcst1	-0.537899209	0.005544042
ENSMUSG00000022272	Myo10	-0.537849635	9.76E-06
ENSMUSG00000024608	Rps14	-0.537672223	0.001949464
ENSMUSG00000029781	Fkbp9	-0.536339638	0.005471906
ENSMUSG00000024313	Mep1b	-0.536321552	0.0048394
ENSMUSG00000055137	Sugct	-0.53601366	0.00421901
ENSMUSG00000021876	Rnase4	-0.535615302	2.02E-08
ENSMUSG00000085451	Gm40614	-0.534384773	0.001684702
ENSMUSG00000039318	Rab3gap2	-0.534253662	4.33E-10
ENSMUSG00000001082	Mfsd10	-0.532511556	1.77E-05
ENSMUSG00000052763	Zfp212	-0.53245515	0.000420713
ENSMUSG00000056666	Retsat	-0.532424098	1.10E-56
ENSMUSG00000018500	Adora2b	-0.530342209	3.62E-24
ENSMUSG00000032714	Syde1	-0.530113377	1.80E-05
ENSMUSG00000024913	Lrp5	-0.527284552	0.042971842
ENSMUSG00000032519	Slc25a38	-0.526128152	0.001928136
ENSMUSG00000064899	Snord118	-0.525045737	0.001632331
ENSMUSG00000052914	Cyp2j6	-0.524571525	6.68E-49
ENSMUSG00000033068	Pdk3	-0.52363848	1.52E-06
ENSMUSG00000098905	Zfp953	-0.523329706	0.002088176
ENSMUSG00000019979	Apaf1	-0.523313474	2.02E-05
ENSMUSG00000061536	Sec22c	-0.523292318	1.71E-09
ENSMUSG00000018417	Myo1b	-0.523031536	0.000134267
ENSMUSG00000058173	Smco4	-0.522947841	0.021524132
ENSMUSG00000049411	Tmem241	-0.522746113	0.0462941
ENSMUSG00000032802	Srxn1	-0.521926072	2.38E-09
ENSMUSG00000031357	Syap1	-0.520864132	0.000325163
ENSMUSG00000005621	Zfp592	-0.520737993	0.014355654
ENSMUSG00000043592	Unc5cl	-0.518860814	0.00112905
ENSMUSG00000049721	Gal3st1	-0.518623316	0.014387553
ENSMUSG00000031146	Plp2	-0.518514594	0.000315431
ENSMUSG00000041498	Kif14	-0.518058482	0.029741042
ENSMUSG00000017868	Sgk2	-0.516625373	0.007896575
ENSMUSG00000061414	Cracr2a	-0.516020785	1.71E-05
ENSMUSG00000015944	Castor2	-0.513419052	0.021427005
ENSMUSG00000003228	Grk5	-0.512094743	0.030771941
ENSMUSG00000069899	Gm12166	-0.510836571	0.000321563
ENSMUSG00000047496	Rnf152	-0.510707344	8.97E-21
ENSMUSG00000000876	Pxmp4	-0.509644167	2.77E-09
ENSMUSG00000035960	Apex1	-0.509621593	0.015689327
ENSMUSG00000019590	Cyb561	-0.508422854	3.34E-06
ENSMUSG00000058624	Gda	-0.508150031	4.49E-13
ENSMUSG00000035896	Rnase1	-0.508141753	2.97E-08
ENSMUSG00000004633	Chn2	-0.507733766	0.000449015
ENSMUSG00000029260	Ugt2b34	-0.507721038	7.51E-54
ENSMUSG00000024900	Cpt1a	-0.507367567	1.57E-06
ENSMUSG00000027006	Dnajc10	-0.507293491	0.000171295
ENSMUSG00000069633	Pex11g	-0.506853662	1.04E-05
ENSMUSG00000024558	Mapk4	-0.506067391	1.17E-09
ENSMUSG00000040883	Tmem205	-0.505566874	6.75E-08
ENSMUSG00000089704	Galnt2	-0.505212456	0.017850123
ENSMUSG00000096727	Psmb9	-0.503895732	2.01E-45
ENSMUSG00000021023	Prorp	-0.503065275	0.014144122
ENSMUSG00000029171	Pgm2	-0.50156338	1.20E-08
ENSMUSG00000030986	Dhx32	-0.501217644	1.30E-05
ENSMUSG00000019838	Slc16a10	-0.501189244	6.29E-06
ENSMUSG00000004931	Apba3	-0.500584199	0.037895684
ENSMUSG00000037242	Clic4	-0.498819004	7.07E-07
ENSMUSG00000041438	Utp4	-0.498763802	0.037281104
ENSMUSG00000098207	Arl14	-0.497953477	2.24E-11
ENSMUSG00000045128	Rpl18a	-0.497757915	1.14E-06
ENSMUSG00000066892	Fbxl12	-0.497708155	0.027736522
ENSMUSG00000078812	Eif5a	-0.497276351	0.001920703
ENSMUSG00000064105	Cnnm2	-0.496244267	4.82E-07
ENSMUSG00000044864	Ankrd50	-0.495944926	0.012160781
ENSMUSG00000024339	Tap2	-0.495487164	6.98E-05
ENSMUSG00000030200	Bcl2l14	-0.493281332	1.36E-19
ENSMUSG00000024747	Aldh1a7	-0.492902364	1.56E-11
ENSMUSG00000102752	Gm7694	-0.492058305	9.12E-16
ENSMUSG00000021608	Lpcat1	-0.490700472	0.003153346
ENSMUSG00000037788	Vopp1	-0.488807994	2.22E-23
ENSMUSG00000022744	Cldnd1	-0.488633138	1.32E-16
ENSMUSG00000041536	Serpina3a	-0.488557853	2.76E-37
ENSMUSG00000005410	Mcm5	-0.488005002	0.002920749
ENSMUSG00000093904	Tomm20	-0.487226219	0.001496085
ENSMUSG00000069922	Ces3a	-0.487222238	0.018563328
ENSMUSG00000040997	Abhd4	-0.486742763	0.000163143
ENSMUSG00000032594	Ip6k1	-0.486379498	0.004174213
ENSMUSG00000011179	Odc1	-0.485704008	0.002633852
ENSMUSG00000001036	Epn2	-0.485639289	0.009713512
ENSMUSG00000020072	Pbld2	-0.485225541	2.42E-24
ENSMUSG00000006641	Slc5a6	-0.485190841	2.09E-57
ENSMUSG00000044681	Cnpy1	-0.484997278	3.51E-05
ENSMUSG00000022982	Sod1	-0.484642299	6.50E-09
ENSMUSG00000034785	Dio1	-0.484509251	5.80E-05
ENSMUSG00000026413	Pkp1	-0.48416152	5.97E-05
ENSMUSG00000026209	Dnpep	-0.483524766	0.001037584
ENSMUSG00000024778	Fas	-0.483425203	2.14E-05
ENSMUSG00000036814	Slc6a20a	-0.482882355	8.82E-12
ENSMUSG00000034570	Inpp5j	-0.482733462	3.34E-12
ENSMUSG00000022210	Dhrs4	-0.482727538	1.42E-27
ENSMUSG00000026657	Frmd4a	-0.48142708	0.04447606
ENSMUSG00000003032	Klf4	-0.481305551	5.40E-13
ENSMUSG00000013275	Slc41a1	-0.480929277	9.96E-11
ENSMUSG00000040584	Abcb1a	-0.480498299	1.12E-09
ENSMUSG00000027551	Zfp64	-0.479956146	0.000940773
ENSMUSG00000054199	Gon4l	-0.479821964	0.001940984
ENSMUSG00000023232	Serinc2	-0.478727663	7.29E-08
ENSMUSG00000001517	Foxm1	-0.477643605	0.000304724
ENSMUSG00000040964	Arhgef10l	-0.476464578	1.82E-10
ENSMUSG00000052631	Sh2d6	-0.476194062	0.026207129
ENSMUSG00000035000	Dpp4	-0.476186066	7.96E-12
ENSMUSG00000039048	Foxred1	-0.474295475	0.000818399
ENSMUSG00000026389	Steap3	-0.474024495	0.000666661
ENSMUSG00000032035	Ets1	-0.473958272	0.0002307
ENSMUSG00000018623	Mmp7	-0.473532948	3.64E-10
ENSMUSG00000079516	Reg3a	-0.47348578	1.14E-16
ENSMUSG00000021550	2210016F16	-0.472816577	3.53E-06
ENSMUSG00000032014	Oaf	-0.471701107	0.035217753
ENSMUSG00000052485	Tmem171	-0.470866212	5.40E-08
ENSMUSG00000025337	Sbds	-0.46718885	1.84E-22
ENSMUSG00000058022	Adtrp	-0.465995814	3.49E-09
ENSMUSG00000019951	Bltp3b	-0.465436674	1.07E-05
ENSMUSG00000022353	Mtss1	-0.465085136	0.026569206
ENSMUSG00000046733	Gprc5a	-0.464810234	0.021149436
ENSMUSG00000040687	Madd	-0.464403301	0.000127325
ENSMUSG00000001576	Ergic1	-0.463587174	7.05E-05
ENSMUSG00000031628	Casp3	-0.463560661	5.48E-104

ENSMUSG00000052539	Magi3	0.570107687	2.29E-06
ENSMUSG00000042507	Mideas	0.569962236	0.011009046
ENSMUSG00000008855	Hdac5	0.569954754	0.000813439
ENSMUSG00000034271	Jdp2	0.569285885	2.85E-06
ENSMUSG00000021416	Eci3	0.568673471	6.03E-19
ENSMUSG00000037390	Muc3	0.568162528	3.39E-05
ENSMUSG00000035168	Tanc1	0.56775413	4.31E-05
ENSMUSG00000044501	Zfp758	0.567553568	1.08E-05
ENSMUSG00000019849	Prep	0.567092759	0.000867088
ENSMUSG00000020955	Ap4s1	0.566968046	0.040894926
ENSMUSG00000026566	Mpzl1	0.566580639	0.004566459
ENSMUSG00000028329	Xpa	0.566154115	0.0391561
ENSMUSG00000035569	Ankrd11	0.565125263	1.49E-10
ENSMUSG00000018217	Pmp22	0.56478778	1.50E-21
ENSMUSG00000032193	Ldlr	0.564142447	1.02E-12
ENSMUSG00000069094	Pde7a	0.562959943	0.001970294
ENSMUSG00000032402	Smad3	0.562435642	0.000400374
ENSMUSG00000021196	Pfkp	0.560214218	0.006849382
ENSMUSG00000035722	Abca7	0.55987301	0.044092504
ENSMUSG00000032570	Atp2c1	0.559237077	0.000233569
ENSMUSG00000001419	Mef2d	0.559029794	3.60E-09
ENSMUSG00000029528	Pxn	0.558571283	0.006328797
ENSMUSG00000033068	Entpd6	0.557863382	0.009325215
ENSMUSG00000034453	Polr3b	0.557159162	0.030134072
ENSMUSG00000026870	Cutal	0.554421653	9.03E-08
ENSMUSG00000068566	Myadm	0.554198979	6.11E-13
ENSMUSG00000036123	Slc9a3	0.553846055	1.95E-16
ENSMUSG00000000628	Hk2	0.553704642	3.51E-24
ENSMUSG00000036461	Elf1	0.55316274	0.011223877
ENSMUSG00000023247	Guca2a	0.553120488	3.81E-59
ENSMUSG00000049504	Proser1	0.55274687	0.021149436
ENSMUSG00000055932	Fto	0.552509732	0.0294161
ENSMUSG00000045441	Gprin3	0.55210231	0.000100861
ENSMUSG00000020458	Rtn4	0.551127612	2.55E-25
ENSMUSG00000032479	Map4	0.550405088	5.54E-10
ENSMUSG00000029236	Nmu	0.550225412	0.020589035
ENSMUSG00000060487	Samd5	0.548667048	5.19E-15
ENSMUSG00000048027	Rgmb	0.548369026	6.39E-05
ENSMUSG00000068876	Cgn	0.545885118	1.34E-10
ENSMUSG00000064373	Selenop	0.544899486	3.03E-12
ENSMUSG00000056919	Cep162	0.544889724	0.001684702
ENSMUSG00000061751	Kalrn	0.544841328	1.23E-05
ENSMUSG00000027720	Il2	0.544717847	1.23E-07
ENSMUSG00000032536	Trak1	0.544478973	2.86E-08
ENSMUSG00000040697	Dnajc16	0.543522126	0.020730953
ENSMUSG00000074024	4632427E13	0.541101746	0.009298266
ENSMUSG00000032068	Plet1	0.540822719	2.39E-10
ENSMUSG00000041272	Tox	0.540465542	6.94E-06
ENSMUSG00000038866	Zcchc2	0.540388761	0.005337033
ENSMUSG00000018126	Baiap2l2	0.540047769	6.45E-09
ENSMUSG00000024222	Fkbp5	0.53902792	1.07E-06
ENSMUSG00000041235	Chd7	0.536924607	3.15E-07
ENSMUSG00000050148	Ubqln2	0.536864456	0.015597078
ENSMUSG00000001098	Kctd10	0.536795222	8.45E-17
ENSMUSG00000057666	Gapdh	0.536551226	1.46E-16
ENSMUSG00000060336	Zfp937	0.536433799	1.58E-06
ENSMUSG00000022587	Ly6e	0.534233778	0.024232465
ENSMUSG00000031672	Got2	0.53407494	0.001611584
ENSMUSG00000044707	Ccnjl	0.533739329	0.028393642
ENSMUSG00000029735	Tpk1	0.533057171	0.001234225
ENSMUSG00000020137	Thap2	0.531085498	0.010812507
ENSMUSG00000022641	Bbx	0.530555074	0.000950705
ENSMUSG00000064215	Ifi27	0.530119782	3.33E-21
ENSMUSG00000023800	Tiam2	0.530061326	0.001469193
ENSMUSG00000031790	Mmp15	0.53001974	1.17E-06
ENSMUSG00000035448	Ccr3	0.529883381	0.003140787
ENSMUSG00000087119	Atg4a-ps	0.529585615	0.019520074
ENSMUSG00000025757	Hspa4l	0.529431217	0.004132785
ENSMUSG00000021376	Tpmt	0.528042098	3.12E-23
ENSMUSG00000026627	Pacc1	0.527861915	0.000775647
ENSMUSG00000073412	Lst1	0.527640713	0.041782234
ENSMUSG00000024843	Chka	0.526902258	1.79E-08
ENSMUSG00000025764	Jade1	0.526342532	0.026491757
ENSMUSG00000002413	Braf	0.526226433	0.005842777
ENSMUSG00000029397	Rchy1	0.525944959	0.01555894
ENSMUSG00000026104	Stat1	0.5259314	2.20E-09
ENSMUSG00000010066	Cacna2d2	0.525260534	0.02404627
ENSMUSG00000021748	Pdhb	0.523755872	8.52E-11
ENSMUSG00000032357	Tinag	0.522998716	2.46E-06
ENSMUSG00000033294	Noc4l	0.522160154	0.005110189
ENSMUSG00000094686	Ccl21a	0.520156666	0.032537862
ENSMUSG00000054414	Slc30a7	0.519684061	0.025601017
ENSMUSG00000047710	Champ1	0.518757695	0.000846162
ENSMUSG00000032121	Tmem218	0.518494023	0.025030331
ENSMUSG00000006205	Htra1	0.517609696	0.018763227
ENSMUSG00000030155	Clec2e	0.516919987	1.06E-30
ENSMUSG00000005125	Ndrg1	0.516241868	0.002352582
ENSMUSG00000034574	Daam1	0.514907924	7.77E-07
ENSMUSG00000039217	Il18	0.514904494	1.02E-57
ENSMUSG00000021244	Ylpm1	0.514157228	8.81E-09
ENSMUSG00000027457	Snph	0.513830577	0.000248451
ENSMUSG00000033306	Lpp	0.513015842	5.11E-06
ENSMUSG00000022708	Zbtb20	0.512946544	0.001428095
ENSMUSG00000051950	B3glct	0.512529085	1.28E-07
ENSMUSG00000000776	Polr3d	0.512315368	2.80E-05
ENSMUSG00000032410	Xrn1	0.511939146	0.00063772
ENSMUSG00000021051	Ppp2r5e	0.511917057	0.013515925
ENSMUSG00000028670	Lypla2	0.51177962	0.008747152
ENSMUSG00000024797	Vps51	0.511094568	0.0087029
ENSMUSG00000023224	Serping1	0.511050576	0.025814206
ENSMUSG00000033159	Cnppd1	0.510109545	0.00366575
ENSMUSG00000037379	Spon2	0.510087623	0.019851384
ENSMUSG00000026749	Nek6	0.51004127	0.026185282
ENSMUSG00000032902	Slc16a1	0.509862269	1.46E-06
ENSMUSG00000061758	Akr1b10	0.508470598	3.36E-08
ENSMUSG00000058454	Dhcr7	0.508137601	0.001294384
ENSMUSG00000039914	Coq10a	0.50800472	0.018226652
ENSMUSG00000001467	Cyp51	0.507929337	0.000355903
ENSMUSG00000057230	Aak1	0.507380773	0.045652398
ENSMUSG00000021831	Ero1a	0.507131632	0.009410071
ENSMUSG00000061393	Acvr2b	0.505600829	0.010006049
ENSMUSG00000034269	Setd5	0.504452912	0.000421334
ENSMUSG00000025915	Sgk3	0.504035771	9.38E-05
ENSMUSG00000031633	Slc25a4	0.503784008	8.46E-25
ENSMUSG00000038888	Ctu1	0.503330576	0.033620746
ENSMUSG00000017713	Tha1	0.503103403	0.000598829
ENSMUSG00000032867	Fbxw8	0.501959003	0.001187139
ENSMUSG00000033713	Foxn3	0.501231493	6.11E-13
ENSMUSG00000037262	Kin	0.500279201	0.047903534
ENSMUSG00000025161	Slc16a3	0.499189724	9.15E-28
ENSMUSG00000052584	Serp2	0.498581864	0.049068391
ENSMUSG00000061474	Mrps36	0.497907565	2.21E-05

ENSMUSG00000022391	Rangap1	-0.495349301	1.79E-11
ENSMUSG00000018395	Kif3a	-0.494141487	0.007556482
ENSMUSG00000002820	Atg4d	-0.494070606	1.71E-11
ENSMUSG00000056529	Ptafr	-0.493585711	1.92E-13
ENSMUSG00000070730	Rmdn3	-0.493221938	1.00E-11
ENSMUSG00000037601	Nme1	-0.492891729	0.007066435
ENSMUSG00000031930	Wwp2	-0.491746703	0.0023727
ENSMUSG00000007041	Clic1	-0.49150105	3.51E-15
ENSMUSG00000018678	Sp2	-0.491070459	0.046713742
ENSMUSG00000031948	Kars	-0.490717891	0.013622843
ENSMUSG00000034480	Diaph2	-0.490400093	0.003440188
ENSMUSG00000021025	Nfkbia	-0.490376883	1.90E-12
ENSMUSG00000028555	Ttc39a	-0.489841607	2.33E-05
ENSMUSG00000030095	Tmem43	-0.489838254	0.001990421
ENSMUSG00000074179	Gsta5	-0.489500568	8.45E-24
ENSMUSG00000022148	Fyb	-0.488128975	9.61E-13
ENSMUSG00000020918	Kat2a	-0.488009764	0.039880051
ENSMUSG00000109324	Prmt1	-0.486807817	0.007309413
ENSMUSG00000026307	Scly	-0.484821145	1.27E-06
ENSMUSG00000020330	Hmmr	-0.484605038	8.37E-05
ENSMUSG00000027712	Anxa5	-0.48407599	0.01856162
ENSMUSG00000019763	Rmnd1	-0.483972393	0.006937704
ENSMUSG00000042489	Clspn	-0.483482036	0.000296132
ENSMUSG00000007034	Slc44a4	-0.482684202	2.61E-38
ENSMUSG00000055401	Fbxo6	-0.482550632	4.15E-06
ENSMUSG00000060073	Psma3	-0.482426758	0.003544068
ENSMUSG00000003814	Calr	-0.482021359	0.000231859
ENSMUSG00000020526	Znhit3	-0.481992086	0.034194934
ENSMUSG00000034636	Zyg11b	-0.481847691	0.001308283
ENSMUSG00000038936	Sccpdh	-0.481607492	0.000198862
ENSMUSG00000024645	Timm21	-0.48090183	5.51E-06
ENSMUSG00000025934	Gsta3	-0.480692247	1.34E-08
ENSMUSG00000021109	Hif1a	-0.480311891	0.00053532
ENSMUSG00000015652	Steap1	-0.479764714	0.001340911
ENSMUSG00000021553	Slc28a3	-0.47942243	0.017811691
ENSMUSG00000024668	Sdhaf2	-0.478868728	7.73E-08
ENSMUSG00000027333	Smox	-0.478835576	4.87E-18
ENSMUSG00000028212	Ccne2	-0.478804075	0.000404731
ENSMUSG00000095813	Defa-ps1	-0.478503017	0.009631029
ENSMUSG00000030677	Kif22	-0.4781321	0.002125223
ENSMUSG00000033114	Slc35d2	-0.477190422	9.23E-09
ENSMUSG00000029102	Hgfac	-0.476926632	3.91E-09
ENSMUSG00000034317	Trim59	-0.476900881	0.022447547
ENSMUSG00000089862	Umad1	-0.476689205	0.017709793
ENSMUSG00000026581	Sell	-0.475375402	0.005968545
ENSMUSG00000054200	Ffar4	-0.475344096	0.000477061
ENSMUSG00000036206	Sh3bp4	-0.47523364	1.73E-09
ENSMUSG00000001467	Cyp51	-0.475171757	0.000355903
ENSMUSG00000034607	Pof1b	-0.474995247	2.04E-07
ENSMUSG00000023800	Tiam2	-0.474600565	0.001469193
ENSMUSG00000020737	Jpt1	-0.473992343	0.01172516
ENSMUSG00000023965	Fbxl17	-0.473353253	0.049868443
ENSMUSG00000066892	Fbxl12	-0.472385274	0.027736522
ENSMUSG00000057137	Tmem140	-0.472055403	2.14E-06
ENSMUSG00000069793	Slfn9	-0.46985936	0.001670766
ENSMUSG00000053644	Aldh7a1	-0.469378405	0.006511955
ENSMUSG00000019039	Dalrd3	-0.469233766	0.04222603
ENSMUSG00000043668	Tox3	-0.469145758	6.86E-08
ENSMUSG00000025545	Clybl	-0.468793149	1.66E-06
ENSMUSG00000063235	Ptpmt1	-0.468587693	2.58E-08
ENSMUSG00000030842	Lamtor1	-0.468265005	0.000155204
ENSMUSG00000062070	Pgk1	-0.466707592	4.77E-05
ENSMUSG00000004032	Gstm5	-0.466556617	0.021524132
ENSMUSG00000002768	Mea1	-0.466490967	0.006145375
ENSMUSG00000025534	Gusb	-0.466170486	0.000365595
ENSMUSG00000027006	Dnajc10	-0.465706867	0.000171295
ENSMUSG00000019773	Fbxo5	-0.464393198	0.00520707
ENSMUSG00000031974	Abcb10	-0.464181814	8.64E-11
ENSMUSG00000028635	Edn2	-0.463139432	1.10E-14
ENSMUSG00000034422	Parp14	-0.462755092	0.01889159
ENSMUSG00000019088	Dnase1l1	-0.462692963	0.000343535
ENSMUSG00000032058	Ppp2r1b	-0.462646734	0.000785267
ENSMUSG00000037072	Selenof	-0.462564425	0.000173235
ENSMUSG00000007036	Abhd16a	-0.462332139	4.82E-06
ENSMUSG00000031146	Plp2	-0.462153727	0.000315431
ENSMUSG00000004864	Mapk13	-0.461576978	0.006063852
ENSMUSG00000030492	Slc7a9	-0.460833294	0.000761763
ENSMUSG00000057506	Bloc1s2	-0.460324129	0.033119485
ENSMUSG00000035699	Slc51a	-0.460246244	1.76E-59
ENSMUSG00000029322	Plac8	-0.459633846	5.14E-33
ENSMUSG00000039183	Nubp2	-0.45957034	0.008339738
ENSMUSG00000041351	Rap1gap	-0.459142773	0.024885058
ENSMUSG00000026914	Psmd14	-0.458157971	0.000241646
ENSMUSG00000031557	Plekha2	-0.457651699	0.012156349
ENSMUSG00000019868	Vta1	-0.457132498	0.000150254
ENSMUSG00000027160	Ccdc34	-0.456596224	0.000681991
ENSMUSG00000061762	Tac1	-0.45659428	0.007967163
ENSMUSG00000028793	Rnf19b	-0.456471921	0.008520681
ENSMUSG00000038721	Hoxb7	-0.456294374	2.30E-05
ENSMUSG00000039105	Atp6v1g1	-0.456026241	0.016332964
ENSMUSG00000004980	Hnrnpa2b1	-0.455564047	0.001156317
ENSMUSG00000052906	Ubxn8	-0.455227064	0.039504754
ENSMUSG00000034192	Lsm3	-0.455012329	3.25E-08
ENSMUSG00000069565	Dazap1	-0.453517888	0.037436518
ENSMUSG00000021556	Golm1	-0.453267218	5.24E-24
ENSMUSG00000032596	Uba7	-0.453023968	0.023476369
ENSMUSG00000040964	Arhgef10l	-0.452190823	1.82E-10
ENSMUSG00000039616	Mocos	-0.451463416	3.73E-07
ENSMUSG00000073399	Trim40	-0.45064548	0.001581644
ENSMUSG00000010914	Pdhx	-0.450259753	6.40E-07
ENSMUSG00000035493	Tgfbi	-0.449925214	1.13E-05
ENSMUSG00000040432	Ltb4r2	-0.449638455	3.81E-06
ENSMUSG00000026333	Gin1	-0.449558696	2.22E-05
ENSMUSG00000049134	Nrap	-0.448968793	0.040924215
ENSMUSG00000071180	Smim15	-0.448249782	2.04E-06
ENSMUSG00000029465	Arpc3	-0.447912866	4.90E-05
ENSMUSG00000100826	Ipw	-0.447695031	1.93E-08
ENSMUSG00000061132	Blnk	-0.447302137	0.003621224
ENSMUSG00000016946	Kctd5	-0.446773868	0.015668953
ENSMUSG00000028648	Ndufs5	-0.446154469	4.56E-06
ENSMUSG00000028673	Fuca1	-0.445890574	1.47E-06
ENSMUSG00000032786	Alas1	-0.443006097	0.000587982
ENSMUSG00000020062	Slc5a8	-0.442649247	1.21E-194
ENSMUSG00000038497	Tmco3	-0.442625784	1.33E-06
ENSMUSG00000003849	Nqo1	-0.44228033	3.63E-12
ENSMUSG00000063239	Grm4	-0.441953964	0.000248731
ENSMUSG00000002731	Prkra	-0.441873379	0.006173151
ENSMUSG00000044734	Serpinb1a	-0.441535118	0.001995062
ENSMUSG00000006313	Upk1a	-0.441420817	0.000195332
ENSMUSG00000057092	Fxyd3	-0.440648352	0.000161492
ENSMUSG00000040767	Snrnp25	-0.440339209	0.000120824
ENSMUSG00000052298	Cdc42se2	-0.439685986	0.028516241

ENSMUSG00000010095	Slc3a2	0.597933533	0.000658285
ENSMUSG00000047730	Fcgbp	0.597464111	9.10E-10
ENSMUSG00000046753	Ccdc66	0.596903266	0.000241571
ENSMUSG00000059326	Csf2ra	0.596655251	1.79E-14
ENSMUSG00000025085	Ablim1	0.595824945	0.004398787
ENSMUSG00000079017	Ifi27l2a	0.595739655	9.90E-10
ENSMUSG00000040412	Elapor1	0.594895909	2.08E-11
ENSMUSG00000004565	Pnpla6	0.594269353	0.023958135
ENSMUSG00000000957	Mmp14	0.593763053	0.003730007
ENSMUSG00000050174	Nudt6	0.593677419	9.68E-05
ENSMUSG00000001288	Rarg	0.592917574	0.00068699
ENSMUSG00000039234	Sec24d	0.590626251	5.82E-21
ENSMUSG00000052713	Zfp608	0.589167788	0.003006681
ENSMUSG00000015597	Zfp318	0.588722665	0.000110198
ENSMUSG00000047409	Ctdspl	0.588346733	1.09E-27
ENSMUSG00000048000	Gigyf2	0.588220342	0.00021241
ENSMUSG00000028152	Tspan5	0.587649662	0.00851043
ENSMUSG00000031445	Proz	0.585785404	8.92E-05
ENSMUSG00000058331	Zfp85	0.585604089	0.012236827
ENSMUSG00000018377	Vezf1	0.585103487	0.001832741
ENSMUSG00000027198	Ext2	0.584671918	0.001532551
ENSMUSG00000027341	Tmem230	0.584464606	5.47E-06
ENSMUSG00000058756	Thra	0.583998052	0.025815408
ENSMUSG00000044628	Rnf208	0.583906481	3.94E-09
ENSMUSG00000021775	Nr1d2	0.582715664	0.000743791
ENSMUSG00000026317	Cln8	0.581908856	6.82E-12
ENSMUSG00000064215	Ifi27	0.581456261	3.33E-21
ENSMUSG00000040270	Bach2	0.580520684	2.82E-06
ENSMUSG00000021823	Vcl	0.578841266	2.74E-05
ENSMUSG00000027809	Etfdh	0.578084373	4.75E-05
ENSMUSG00000029647	Pan3	0.577340992	1.77E-05
ENSMUSG00000035168	Tanc1	0.577185687	4.31E-05
ENSMUSG00000040447	Spns2	0.57642562	4.00E-10
ENSMUSG00000030725	Lipt2	0.576148703	0.025601017
ENSMUSG00000028229	Rmdn1	0.575778615	9.26E-26
ENSMUSG00000022000	Zc3h13	0.573690951	0.029140189
ENSMUSG00000040761	Spen	0.571375768	1.24E-07
ENSMUSG00000023232	Serinc2	0.570917521	7.29E-08
ENSMUSG00000021143	Pacs2	0.570753077	0.001316979
ENSMUSG00000041417	Pik3r1	0.570032123	0.000513393
ENSMUSG00000037949	Ano10	0.569719963	0.000206709
ENSMUSG00000032528	Vipr1	0.569521967	0.00043244
ENSMUSG00000037552	Plekhg2	0.569412261	7.85E-05
ENSMUSG00000030761	Myo7a	0.569237365	0.029868586
ENSMUSG00000057637	Prdm2	0.568615463	0.003113836
ENSMUSG00000024140	Epas1	0.56802932	0.020981507
ENSMUSG00000030583	Sipa1l3	0.56645644	4.56E-06
ENSMUSG00000009614	Sardh	0.566237931	0.003884172
ENSMUSG00000027854	Sike1	0.564881048	0.00040374
ENSMUSG00000010307	Tmem86a	0.563820709	0.023835012
ENSMUSG00000007817	Zmiz1	0.562902845	8.98E-11
ENSMUSG00000060600	Eno3	0.562205231	0.000378771
ENSMUSG00000086040	Wipf3	0.562195554	0.021404014
ENSMUSG00000032500	Dclk3	0.562071731	0.040924215
ENSMUSG00000034801	Sos2	0.561912014	0.009038371
ENSMUSG00000121513	Ptprg	0.561565423	0.01070431
ENSMUSG00000023055	Calcoco1	0.560413536	0.033378418
ENSMUSG00000018415	Gid4	0.559900067	0.000609393
ENSMUSG00000038150	Ormdl3	0.559845969	1.97E-12
ENSMUSG00000053898	Ech1	0.559099321	2.93E-10
ENSMUSG00000034930	Rtkn	0.557929734	0.005875082
ENSMUSG00000032485	Scap	0.557032904	3.90E-05
ENSMUSG00000039270	Megf9	0.557026399	1.08E-08
ENSMUSG00000038538	Ubn2	0.556870451	0.000187236
ENSMUSG00000095385	D630033O11	0.556612099	0.005267069
ENSMUSG00000002565	Scin	0.556554925	1.69E-07
ENSMUSG00000028607	Cpt2	0.55654129	1.21E-09
ENSMUSG00000044702	Palb2	0.556369449	0.000564159
ENSMUSG00000025745	Hadha	0.55576435	8.66E-05
ENSMUSG00000045725	Prr15	0.555603365	7.30E-15
ENSMUSG00000025762	Larp1b	0.555020381	0.007133454
ENSMUSG00000024604	Rbm22	0.554736272	0.006849382
ENSMUSG00000001034	Mapk7	0.553989848	0.015258993
ENSMUSG00000014075	Dynlt2b	0.553972915	5.54E-11
ENSMUSG00000031993	Snx19	0.553639877	0.022501547
ENSMUSG00000032536	Trak1	0.553365802	2.86E-08
ENSMUSG00000032454	Rbp2	0.553075153	1.49E-78
ENSMUSG00000001855	Nup214	0.552886342	0.008133624
ENSMUSG00000036644	Tbc1d9b	0.551810977	0.000271423
ENSMUSG00000028565	Nfia	0.551237576	3.08E-08
ENSMUSG00000028106	Rprd2	0.550865784	0.000229063
ENSMUSG00000029179	Zcchc4	0.548317414	0.026731623
ENSMUSG00000030214	Plbd1	0.548125302	0.004005103
ENSMUSG00000055204	Ankrd17	0.545915684	7.48E-09
ENSMUSG00000061751	Kalrn	0.545218705	1.23E-05
ENSMUSG00000044501	Zfp758	0.544985569	1.08E-05
ENSMUSG00000033715	Akr1c14	0.544975082	1.85E-11
ENSMUSG00000024968	Rcor2	0.54280132	0.036570332
ENSMUSG00000014361	Mertk	0.542019942	0.017809877
ENSMUSG00000064177	Ghrl	0.541487918	7.28E-51
ENSMUSG00000034430	Zxdc	0.541245426	0.006563997
ENSMUSG00000008822	Acyp1	0.54120034	1.57E-05
ENSMUSG00000078517	Emc1	0.540480187	7.36E-05
ENSMUSG00000005580	Adcy9	0.54043864	0.015891634
ENSMUSG00000050395	Tnfsf15	0.540410148	1.94E-05
ENSMUSG00000022521	Crebbp	0.540227126	1.16E-09
ENSMUSG00000031438	Rnf128	0.539828263	9.71E-07
ENSMUSG00000064341	ND1	0.539665517	0.028045232
ENSMUSG00000075706	Gpx4	0.539630633	0.014690733
ENSMUSG00000045107	Saysd1	0.539394974	0.014292265
ENSMUSG00000001911	Nfix	0.539025795	0.001474982
ENSMUSG00000061313	Ddhd2	0.538707009	1.86E-12
ENSMUSG00000036333	Kidins220	0.538699382	0.001527026
ENSMUSG00000056602	Fry	0.538550649	0.005681935
ENSMUSG00000033478	Fhip2a	0.538533404	0.005975048
ENSMUSG00000032666	1700025G04	0.538400327	5.45E-12
ENSMUSG00000049940	Pgrmc2	0.538114643	5.51E-42
ENSMUSG00000005951	Shpk	0.536330721	1.08E-05
ENSMUSG00000037692	Ahdc1	0.535929213	0.000354105
ENSMUSG00000038298	Pdzk1	0.535498104	5.73E-11
ENSMUSG00000103711	Tstd1	0.53522774	2.67E-10
ENSMUSG00000031732	Phlpp2	0.534820461	3.19E-05
ENSMUSG00000026360	Rgs2	0.534611432	4.74E-05
ENSMUSG00000003458	Ncstn	0.534304867	0.011223877
ENSMUSG00000020661	Dnmt3a	0.532849855	1.32E-06
ENSMUSG00000031642	Sh3rf1	0.532707013	1.79E-10
ENSMUSG00000042810	Krba1	0.532002751	5.65E-06
ENSMUSG00000038244	Mical2	0.531865529	1.20E-05
ENSMUSG00000020105	Lrig3	0.5316438	8.49E-08
ENSMUSG00000023905	Tnfrsf12a	0.530777083	4.29E-05
ENSMUSG00000023236	Scg5	0.530386545	0.028911631
ENSMUSG00000025034	Trim8	0.529359577	0.00014056

ENSMUSG00000020918	Kat2a	-0.463463766	0.039880051
ENSMUSG00000047501	Cldn4	-0.462273765	0.00016321
ENSMUSG00000036606	Plxnb2	-0.461461867	2.19E-11
ENSMUSG00000025158	Rfng	-0.461263614	1.40E-05
ENSMUSG00000037266	Rsrp1	-0.460882208	7.36E-07
ENSMUSG00000040767	Snrnp25	-0.459651281	0.000120824
ENSMUSG00000054072	Iigp1	-0.459339532	0.000914034
ENSMUSG00000036138	Acaa1a	-0.459094697	6.47E-11
ENSMUSG00000047180	Neurl3	-0.4589756	0.00116571
ENSMUSG00000024338	Psmb8	-0.458678335	5.00E-47
ENSMUSG00000020309	Chac2	-0.45848031	2.87E-05
ENSMUSG00000026632	Tatdn3	-0.457916445	5.06E-05
ENSMUSG00000026509	Capn2	-0.45770237	0.026162214
ENSMUSG00000022434	Fam118a	-0.457121662	2.86E-05
ENSMUSG00000030681	Mvp	-0.456402724	2.46E-26
ENSMUSG00000027331	Knstrn	-0.455784777	0.014572191
ENSMUSG00000048186	Bend7	-0.455255286	5.39E-49
ENSMUSG00000041515	Irf8	-0.454200085	3.09E-15
ENSMUSG00000035828	Pim3	-0.454111587	0.000176905
ENSMUSG00000017493	Igfbp4	-0.453980098	3.94E-05
ENSMUSG00000064120	Mocs1	-0.453445317	0.014551215
ENSMUSG00000045975	C2cd2	-0.452907549	4.50E-11
ENSMUSG00000025997	Ikzf2	-0.452192812	0.026991512
ENSMUSG00000030056	Isy1	-0.452095799	0.023618942
ENSMUSG00000028709	Mob3c	-0.451983109	0.014260989
ENSMUSG00000075520	Malrd1	-0.451155127	7.41E-29
ENSMUSG00000015224	Cyp2j9	-0.450955682	2.63E-11
ENSMUSG00000024818	Slc25a45	-0.450108829	2.72E-37
ENSMUSG00000002963	Pnkp	-0.450034648	0.003493378
ENSMUSG00000023886	Smoc2	-0.450033511	0.009580071
ENSMUSG00000028708	Mknk1	-0.448834682	0.026902809
ENSMUSG00000020229	Slc5a4a	-0.445797412	2.13E-08
ENSMUSG00000029165	Agbl5	-0.445659586	0.002914419
ENSMUSG00000022433	Csnk1e	-0.445625963	0.042765575
ENSMUSG00000060615	Ang4	-0.444976654	1.69E-12
ENSMUSG00000037542	Aldh8a1	-0.444842539	0.037401339
ENSMUSG00000018697	Aatf	-0.444291932	0.005351994
ENSMUSG00000029502	Golga3	-0.444002253	0.000355004
ENSMUSG00000020019	Ntn4	-0.443843946	0.00104114
ENSMUSG00000020634	Ubxn2a	-0.443560645	0.021533336
ENSMUSG00000049580	Tsku	-0.443039805	0.004377194
ENSMUSG00000042524	Sun2	-0.442932295	0.006284639
ENSMUSG00000034445	Cyb561a3	-0.442242234	0.016054813
ENSMUSG00000019564	Arid3a	-0.442224765	0.042971842
ENSMUSG00000037190	Cyb561d2	-0.441503547	0.015240534
ENSMUSG00000031093	Dock11	-0.439754691	0.000483816
ENSMUSG00000037664	Cdkn1c	-0.439306418	1.49E-08
ENSMUSG00000006345	Ggt1	-0.438922994	3.31E-10
ENSMUSG00000026950	Neb	-0.438398034	0.004017009
ENSMUSG00000121584	Zftraf1	-0.438344601	0.020785279
ENSMUSG00000074457	S100a16	-0.438152996	7.21E-10
ENSMUSG00000031822	Gse1	-0.437623561	1.19E-05
ENSMUSG00000053931	Cnn3	-0.437395027	0.003572122
ENSMUSG00000028664	Ephb2	-0.435948379	7.05E-05
ENSMUSG00000032300	1700017B05	-0.434965107	0.002672609
ENSMUSG00000035944	Ttc38	-0.434238484	0.002128791
ENSMUSG00000000142	Axin2	-0.432953836	4.37E-25
ENSMUSG00000055976	Cldn23	-0.431118658	0.000421334
ENSMUSG00000015357	Clpx	-0.429220743	0.00544832
ENSMUSG00000057561	Eif1a	-0.4285874	0.001266079
ENSMUSG00000060427	Zfp868	-0.428437152	0.0011826
ENSMUSG00000029247	Paics	-0.428426589	0.011182021
ENSMUSG00000026880	Stom	-0.427620468	1.14E-08
ENSMUSG00000025140	Pycr1	-0.426153659	0.000683781
ENSMUSG00000032724	Abtb2	-0.425527304	0.044995924
ENSMUSG00000027163	Commd9	-0.425413021	0.028139588
ENSMUSG00000039364	Sectm1b	-0.425339395	7.16E-06
ENSMUSG00000045826	Ptprcap	-0.424464624	0.026991512
ENSMUSG00000066595	Flvcr1	-0.424408062	2.57E-59
ENSMUSG00000010095	Slc3a2	-0.423132272	0.000658285
ENSMUSG00000069601	Ank3	-0.422756874	0.000748388
ENSMUSG00000078670	Fam174b	-0.422603234	4.95E-10
ENSMUSG00000021139	Gm20498	-0.422216194	0.048865101
ENSMUSG00000039208	Metrnl	-0.421894424	0.00060125
ENSMUSG00000005625	Psmd4	-0.421835884	2.83E-08
ENSMUSG00000033596	Rfwd3	-0.421470173	0.024473224
ENSMUSG00000060802	B2m	-0.42120901	2.46E-07
ENSMUSG00000056602	Fry	-0.419929578	0.005681935
ENSMUSG00000088252	Snord13	-0.419481633	2.01E-14
ENSMUSG00000040463	Mybbp1a	-0.419410156	0.029212476
ENSMUSG00000001663	Gstt1	-0.418533742	3.80E-149
ENSMUSG00000027227	Sord	-0.418504256	1.15E-07
ENSMUSG00000003721	Insig2	-0.417819838	1.12E-09
ENSMUSG00000078713	Tomm5	-0.417365417	0.000151342
ENSMUSG00000060791	Gmfg	-0.416879248	7.46E-06
ENSMUSG00000024414	Mrpl27	-0.41614513	2.28E-07
ENSMUSG00000026617	Bpnt1	-0.415788819	1.44E-98
ENSMUSG00000051627	H1f4	-0.415617321	0.00206194
ENSMUSG00000027907	S100a11	-0.414990559	1.65E-07
ENSMUSG00000032046	Abhd12	-0.414887857	0.000536892
ENSMUSG00000034789	Rab24	-0.414863844	0.020679189
ENSMUSG00000063236	1110038F14I	-0.414527485	0.00529627
ENSMUSG00000042462	Dctpp1	-0.414338853	6.18E-05
ENSMUSG00000028088	Fmo5	-0.413759982	4.58E-11
ENSMUSG00000003868	Ruvbl2	-0.41354955	0.040095291
ENSMUSG00000025792	Slc25a10	-0.413309963	0.007445477
ENSMUSG00000029530	Ccr9	-0.413308076	0.045596836
ENSMUSG00000016495	Plgrkt	-0.412808639	0.009772702
ENSMUSG00000015970	Chdh	-0.412448581	8.66E-05
ENSMUSG00000120992	D17H6S56E-	-0.412178405	7.00E-14
ENSMUSG00000026618	Iars2	-0.41200995	4.93E-08
ENSMUSG00000025362	Rps26	-0.411658165	2.22E-10
ENSMUSG00000036768	Kif15	-0.41129145	0.000488647
ENSMUSG00000029821	Gsdme	-0.410992226	3.82E-96
ENSMUSG00000029922	Mkrn1	-0.410924806	4.48E-08
ENSMUSG00000045071	E130308A19	-0.41087804	0.001768159
ENSMUSG00000053644	Aldh7a1	-0.410875973	0.006511955
ENSMUSG00000025616	Usp16	-0.41083012	1.81E-06
ENSMUSG00000032602	Slc25a20	-0.410589292	0.013172615
ENSMUSG00000041202	Pla2g2d	-0.410333786	0.020590466
ENSMUSG00000033684	Qsox1	-0.409895691	1.44E-18
ENSMUSG00000002014	Ssr4	-0.409842962	1.10E-05
ENSMUSG00000008136	Fhl2	-0.409811672	0.007128531
ENSMUSG00000028416	Bag1	-0.409212427	8.64E-05
ENSMUSG00000030717	Nupr1	-0.408222713	3.31E-34
ENSMUSG00000061286	Exosc5	-0.407976875	0.036793326
ENSMUSG00000027371	Fahd2a	-0.407937759	1.46E-05
ENSMUSG00000010045	Tmem115	-0.407886032	0.048090968
ENSMUSG00000010054	Tusc2	-0.407391083	7.88E-06
ENSMUSG00000032010	Usp2	-0.407321835	0.000645534
ENSMUSG00000026003	Acadl	-0.406949316	2.27E-16
ENSMUSG00000029727	Cyp3a13	-0.406273573	9.75E-109

ENSMUSG00000038056	Kmt2c	0.497308124	0.00059979
ENSMUSG00000058729	Lin9	0.495906553	8.51E-05
ENSMUSG00000024098	Twsg1	0.493871741	0.003722434
ENSMUSG00000034422	Parp14	0.493331704	0.01889159
ENSMUSG00000021143	Pacs2	0.49316324	0.001316979
ENSMUSG00000045962	Wnk1	0.491927318	3.38E-09
ENSMUSG00000023861	Mpc1	0.491725346	3.92E-09
ENSMUSG00000005533	Igf1r	0.490953338	2.40E-13
ENSMUSG00000024298	Zfp871	0.48978575	0.005817429
ENSMUSG00000026356	Dars	0.489773115	0.001057081
ENSMUSG00000030304	Ergic2	0.488829942	5.31E-08
ENSMUSG00000074715	Ccl28	0.487353333	8.97E-12
ENSMUSG00000001999	Blvra	0.48532747	0.013841793
ENSMUSG00000030706	Mrpl48	0.484415276	1.96E-07
ENSMUSG00000037921	Ddx60	0.484013185	1.90E-27
ENSMUSG00000005951	Shpk	0.483100019	1.08E-05
ENSMUSG00000036499	Eea1	0.482820219	7.22E-10
ENSMUSG00000038418	Egr1	0.482080201	1.74E-09
ENSMUSG00000022827	Rabl3	0.48160386	0.014528976
ENSMUSG00000047045	Tmem164	0.481408	4.28E-10
ENSMUSG00000085995	Gm2788	0.480543285	4.30E-08
ENSMUSG00000033883	Zfp267	0.480167669	0.036480425
ENSMUSG00000120425	Ndufb4b	0.478815472	1.98E-06
ENSMUSG00000001418	Glmp	0.478051773	0.002491715
ENSMUSG00000097101	1810034E14I	0.47748244	0.008513972
ENSMUSG00000006538	Ihh	0.47620861	0.01134154
ENSMUSG00000068856	Sf3b4	0.4762048	4.41E-05
ENSMUSG00000051413	Plagl2	0.475854964	0.049406237
ENSMUSG00000046159	Chrm3	0.47559834	0.008001785
ENSMUSG00000020427	Igfbp3	0.47532972	1.93E-07
ENSMUSG00000023019	Gpd1	0.474495652	1.67E-39
ENSMUSG00000041301	Cftr	0.47357091	2.76E-11
ENSMUSG00000029417	Cxcl9	0.472456296	3.12E-08
ENSMUSG00000036246	Gmip	0.471892634	0.000113948
ENSMUSG00000003119	Cdk12	0.471842017	3.75E-05
ENSMUSG00000022246	Rai14	0.471511527	0.018215079
ENSMUSG00000024050	Wiz	0.470509139	0.002660214
ENSMUSG00000041351	Rap1gap	0.4702906	0.024885058
ENSMUSG00000021540	Smad5	0.470147374	0.000205881
ENSMUSG00000046185	Zfp84	0.469970983	1.72E-05
ENSMUSG00000033373	Fntb	0.46971115	2.47E-07
ENSMUSG00000028967	Errfi1	0.4696735	4.88E-05
ENSMUSG00000021978	Extl3	0.469142516	0.005430103
ENSMUSG00000046213	Cym	0.468779486	1.17E-128
ENSMUSG00000019791	Hint3	0.468490836	3.02E-09
ENSMUSG00000014351	Gip	0.468046645	3.09E-66
ENSMUSG00000026313	Hdac4	0.467987209	0.00401388
ENSMUSG00000032062	2310030G06	0.46786538	0.007489876
ENSMUSG00000055912	Tmem150a	0.467721689	0.000962376
ENSMUSG00000071654	Uqcc3	0.466830182	0.000505953
ENSMUSG00000022957	Itsn1	0.466421628	4.70E-05
ENSMUSG00000035203	Epn1	0.466175935	0.028016101
ENSMUSG00000085980	Gm12408	0.465981461	0.006727072
ENSMUSG00000008822	Acyp1	0.465849213	1.57E-05
ENSMUSG00000078636	Gm7336	0.465193577	2.62E-06
ENSMUSG00000020917	Acly	0.464774625	3.92E-05
ENSMUSG00000002565	Scin	0.463711596	1.69E-07
ENSMUSG00000020883	Fbxl20	0.463638797	0.011413888
ENSMUSG00000035239	Neu3	0.463082747	1.18E-22
ENSMUSG00000060600	Eno3	0.463051111	0.000378771
ENSMUSG00000032349	Elovl5	0.462503588	7.01E-23
ENSMUSG00000055541	Lair1	0.461641578	3.54E-07
ENSMUSG00000038371	Sbf2	0.461431889	0.042758846
ENSMUSG00000078439	Smim24	0.461300581	9.16E-82
ENSMUSG00000044702	Palb2	0.460831792	0.000564159
ENSMUSG00000045934	Mtmr11	0.460548969	5.34E-11
ENSMUSG00000097222	Gata6os	0.460344019	0.001122192
ENSMUSG00000056598	Drc3	0.460164182	0.000691367
ENSMUSG00000002968	Med25	0.459829888	0.000240937
ENSMUSG00000027420	Bfsp1	0.459033555	0.00205409
ENSMUSG00000027180	Fbxo3	0.458760069	0.001649242
ENSMUSG00000024727	Trpm6	0.458430797	2.98E-31
ENSMUSG00000035021	Baz1a	0.458035775	2.82E-06
ENSMUSG00000042647	Acad12	0.457985371	2.39E-06
ENSMUSG00000022394	L3mbtl2	0.457372907	0.044291234
ENSMUSG00000051314	Ffar2	0.45688449	4.97E-05
ENSMUSG00000079419	Ms4a6c	0.456645374	0.020961664
ENSMUSG00000033237	Arid2	0.45659643	0.001153339
ENSMUSG00000043162	Pyurf	0.456505282	4.77E-05
ENSMUSG00000031444	F10	0.456402047	1.98E-09
ENSMUSG00000095325	Zfp870	0.456066758	4.50E-07
ENSMUSG00000019852	Arfgef3	0.45464005	3.97E-23
ENSMUSG00000033863	Klf9	0.454000446	4.34E-15
ENSMUSG00000039414	Heatr5b	0.453874105	0.01070431
ENSMUSG00000025278	Flnb	0.453624388	2.21E-09
ENSMUSG00000091721	Gimd1	0.453484628	2.23E-09
ENSMUSG00000000394	Gcg	0.453380375	4.31E-05
ENSMUSG00000043683	Fem1a	0.452898624	0.011680706
ENSMUSG00000076617	Ighm	0.452582277	0.027363156
ENSMUSG00000006378	Gcat	0.452492405	1.79E-25
ENSMUSG00000047875	Gpr157	0.451406137	7.74E-16
ENSMUSG00000034621	Gpatch8	0.451026153	0.005990325
ENSMUSG00000041920	Slc16a6	0.450984141	5.17E-126
ENSMUSG00000097203	4732419C18	0.450877361	0.000353454
ENSMUSG00000042700	Sipa1l1	0.450393646	1.83E-06
ENSMUSG00000026116	Tmem131	0.450160746	3.93E-08
ENSMUSG00000063953	Amd2	0.45006734	0.011130369
ENSMUSG00000025197	Cyp2c23	0.449804943	3.38E-10
ENSMUSG00000031529	Tnks	0.449543705	0.001280563
ENSMUSG00000021241	Isca2	0.449391626	1.51E-07
ENSMUSG00000035754	Wdr18	0.448894809	0.031528933
ENSMUSG00000040481	Bptf	0.4487305	1.82E-14
ENSMUSG00000021124	Vti1b	0.448705425	2.87E-06
ENSMUSG00000060477	Irak2	0.448396502	0.009001843
ENSMUSG00000015340	Cybb	0.448222769	0.045076778
ENSMUSG00000024085	Man2a1	0.447804141	0.000397208
ENSMUSG00000029860	Zyx	0.447619868	0.004059296
ENSMUSG00000050705	2310061I04R	0.447393806	0.048716002
ENSMUSG00000036214	Polr1has	0.447093647	9.27E-07
ENSMUSG00000051768	Xrcc1	0.446127928	0.033771177
ENSMUSG00000036438	Calm2	0.446024537	0.017199492
ENSMUSG00000039477	Tnrc18	0.445500018	1.74E-07
ENSMUSG00000033917	Gde1	0.445353151	6.66E-26
ENSMUSG00000020272	Stk10	0.445295688	0.000377942
ENSMUSG00000099632	2900093K20	0.445107957	0.034904457
ENSMUSG00000031832	Taf1c	0.445080423	0.012161377
ENSMUSG00000036887	C1qa	0.443732927	7.31E-06
ENSMUSG00000036019	Tmtc2	0.441789221	0.029203545
ENSMUSG00000025571	Tnrc6c	0.44147429	7.22E-08
ENSMUSG00000024974	Smc3	0.441023661	7.15E-13
ENSMUSG00000038564	Ift172	0.44086743	3.52E-12
ENSMUSG00000085315	A430018G15	0.440678289	0.000657344

ENSMUSG00000046733	Gprc5a	-0.43892976	0.021149436
ENSMUSG00000026701	Prdx6	-0.43889273	9.46E-07
ENSMUSG00000046756	Mrps7	-0.438687103	0.000987671
ENSMUSG00000028367	Txn1	-0.437734162	2.70E-15
ENSMUSG00000029338	Antxr2	-0.437237232	2.14E-10
ENSMUSG00000027248	Pdia3	-0.436179582	0.002761641
ENSMUSG00000024181	Mrpl28	-0.435810525	1.71E-05
ENSMUSG00000022848	Slc49a4	-0.435646581	0.041164597
ENSMUSG00000030157	Clec2d	-0.435361768	0.002699393
ENSMUSG00000056612	Ppp1r14b	-0.435340077	6.15E-05
ENSMUSG00000050188	Lsm10	-0.435076771	0.008309343
ENSMUSG00000029730	Mcm7	-0.434790494	0.002040617
ENSMUSG00000060802	B2m	-0.434224091	2.46E-07
ENSMUSG00000032009	Sesn3	-0.433827196	1.54E-06
ENSMUSG00000046718	Bst2	-0.433739288	7.26E-07
ENSMUSG00000064036	Mro	-0.4325259	0.012370556
ENSMUSG00000024963	Dnajc4	-0.432170257	0.000550684
ENSMUSG00000029815	Malsu1	-0.432123457	0.000163342
ENSMUSG00000029270	Dipk1a	-0.431943144	0.001811975
ENSMUSG00000041957	Pkp2	-0.431765819	0.00183989
ENSMUSG00000023072	Cep89	-0.43149025	0.031886289
ENSMUSG00000054766	Set	-0.430587702	7.29E-05
ENSMUSG00000026708	Cenpl	-0.430279956	0.023076627
ENSMUSG00000046432	Bex3	-0.43015796	0.001516644
ENSMUSG00000027952	Pmvk	-0.430137294	0.022019075
ENSMUSG00000042524	Sun2	-0.42996062	0.006284639
ENSMUSG00000042396	Rbm7	-0.429556358	3.36E-09
ENSMUSG00000036781	Rps27l	-0.42733978	0.000498526
ENSMUSG00000031672	Got2	-0.427018345	0.001611584
ENSMUSG00000040883	Tmem205	-0.426865623	6.75E-08
ENSMUSG00000034932	Mrpl54	-0.426458171	3.36E-10
ENSMUSG00000060615	Ang4	-0.42639021	1.69E-12
ENSMUSG00000004099	Dnmt1	-0.426341711	0.000252785
ENSMUSG00000026675	Hsd17b7	-0.425353126	0.000657318
ENSMUSG00000035944	Ttc38	-0.424219144	0.002128791
ENSMUSG00000038009	Dnajc22	-0.423957798	0.000142323
ENSMUSG00000030538	Cib1	-0.42384852	0.026818078
ENSMUSG00000033565	Rbfox2	-0.423613172	0.002194184
ENSMUSG00000026784	Pdss1	-0.423116351	1.09E-05
ENSMUSG00000027828	Ssr3	-0.422152663	0.000623364
ENSMUSG00000015112	Slc25a13	-0.421916205	1.79E-19
ENSMUSG00000026049	Tex30	-0.421427095	0.029257646
ENSMUSG00000027195	Hsd17b12	-0.4211593	0.001033397
ENSMUSG00000013629	Cad	-0.421117419	8.26E-07
ENSMUSG00000022824	Muc13	-0.4209403	0.023220419
ENSMUSG00000028656	Cap1	-0.420234968	0.000212026
ENSMUSG00000060791	Gmfg	-0.420074747	7.46E-06
ENSMUSG00000049932	H2ax	-0.419938437	0.02594076
ENSMUSG00000049382	Krt8	-0.419783416	3.59E-15
ENSMUSG00000021432	Slc35b3	-0.419772277	0.018903441
ENSMUSG00000016319	Slc25a5	-0.418927945	2.44E-08
ENSMUSG00000035069	Oma1	-0.418457404	0.000583543
ENSMUSG00000029554	Mad1l1	-0.418148807	0.014212431
ENSMUSG00000047501	Cldn4	-0.417665818	0.00016321
ENSMUSG00000051319	Mtln	-0.41760592	0.007412826
ENSMUSG00000039016	Timm8b	-0.417264106	6.64E-10
ENSMUSG00000021024	Psma6	-0.416767644	0.000391629
ENSMUSG00000078153	Psme2b	-0.416744506	1.85E-11
ENSMUSG00000040466	Blvrb	-0.41669996	9.45E-06
ENSMUSG00000026238	Ptma	-0.416586476	0.001807062
ENSMUSG00000025894	Aasdhppt	-0.414443518	0.003327901
ENSMUSG00000040771	Oard1	-0.414000903	0.029872325
ENSMUSG00000036813	Entpd8	-0.413997259	2.07E-13
ENSMUSG00000026277	Stk25	-0.413900162	0.015602812
ENSMUSG00000060679	Mrps9	-0.413769117	0.000227434
ENSMUSG00000027076	Timm10	-0.413443909	1.32E-10
ENSMUSG00000029650	Slc46a3	-0.412979649	0.0001302
ENSMUSG00000070661	Rnf186	-0.412892313	2.81E-27
ENSMUSG00000040213	Kyat3	-0.412371448	0.047208109
ENSMUSG00000047881	Rell1	-0.41222737	1.37E-40
ENSMUSG00000054702	Ap1s3	-0.412099989	7.55E-51
ENSMUSG00000022370	Mrpl13	-0.41182272	1.12E-07
ENSMUSG00000038213	Tapbpl	-0.410939555	0.000189635
ENSMUSG00000046223	Plaur	-0.410465679	0.032968492
ENSMUSG00000041426	Hibch	-0.409745	0.000151987
ENSMUSG00000027293	Ehd4	-0.40918157	4.54E-12
ENSMUSG00000018446	C1qbp	-0.408199213	0.022814218
ENSMUSG00000042606	Hirip3	-0.407338796	0.026751239
ENSMUSG00000053317	Sec61b	-0.406944462	0.036229616
ENSMUSG00000059714	Flot1	-0.406841443	0.022087364
ENSMUSG00000067274	Rplp0	-0.406522151	0.008059402
ENSMUSG00000028098	Rnf115	-0.406181489	0.039284188
ENSMUSG00000040385	Ppp1ca	-0.405858795	0.041138171
ENSMUSG00000007850	Hnrnph1	-0.405817167	0.0177659
ENSMUSG00000021905	Dph3	-0.405758013	5.29E-06
ENSMUSG00000017146	Brca1	-0.405428516	0.019746485
ENSMUSG00000048970	C1galt1c1	-0.404543241	0.003983908
ENSMUSG00000028680	Plk3	-0.404480065	0.003875591
ENSMUSG00000029267	Mtf2	-0.404091702	0.037824587
ENSMUSG00000006289	Osgep	-0.40380101	0.006125863
ENSMUSG00000023942	Slc29a1	-0.403595745	0.013507646
ENSMUSG00000060143	Gm10076	-0.403551839	0.004904039
ENSMUSG00000046688	Tifa	-0.402722262	4.48E-13
ENSMUSG00000022377	Asap1	-0.402581935	0.010334594
ENSMUSG00000068523	Gng5	-0.402083065	0.032121894
ENSMUSG00000073676	Hspe1	-0.401953723	0.001333094
ENSMUSG00000020873	Slc35b1	-0.401771174	0.000478555
ENSMUSG00000020571	Pdia6	-0.401454058	9.56E-12
ENSMUSG00000001627	Ifrd1	-0.40135905	7.53E-11
ENSMUSG00000028333	Anp32b	-0.401212453	1.52E-09
ENSMUSG00000041261	Car8	-0.401089496	1.19E-12
ENSMUSG00000020272	Stk10	-0.399935942	0.000377942
ENSMUSG00000032381	Ciao2a	-0.399364709	2.15E-11
ENSMUSG00000032479	Map4	-0.398391176	5.54E-10
ENSMUSG00000028898	Trnau1ap	-0.398384232	0.00174785
ENSMUSG00000079197	Psme2	-0.398217958	1.99E-07
ENSMUSG00000048572	Tmem252	-0.397927637	5.74E-10
ENSMUSG00000026970	Rbms1	-0.397815462	0.011538857
ENSMUSG00000056671	Prelid2	-0.397467368	0.022525236
ENSMUSG00000068747	Sort1	-0.396605356	6.10E-18
ENSMUSG00000019888	Mgat4c	-0.395492799	3.51E-299
ENSMUSG00000002963	Pnkp	-0.395276972	0.003493378
ENSMUSG00000053931	Cnn3	-0.395108508	0.003572122
ENSMUSG00000042129	Rassf4	-0.395097229	3.61E-13
ENSMUSG00000026202	Tuba4a	-0.393896344	4.47E-22
ENSMUSG00000027566	Psma7	-0.393854336	9.31E-08
ENSMUSG00000023861	Mpc1	-0.393681194	3.92E-09
ENSMUSG00000101031	Ms4a12	-0.393436471	2.63E-09
ENSMUSG00000085126	Gm12589	-0.393117853	3.85E-13
ENSMUSG00000007029	Vars	-0.392619965	9.12E-09
ENSMUSG00000022844	Pdia5	-0.392174026	0.00340953
ENSMUSG00000069744	Psmb3	-0.391550245	0.003781037

ENSMUSG00000096188	Cmtm4	0.52907271	9.83E-12
ENSMUSG00000024388	Myo7b	0.52861613	0.006930624
ENSMUSG00000039145	Camk1d	0.528176684	1.71E-20
ENSMUSG00000048126	Col6a3	0.527835322	0.005612682
ENSMUSG00000029673	Auts2	0.526066272	5.01E-06
ENSMUSG00000042638	Gucy2c	0.525269409	0.000688803
ENSMUSG00000024426	Atat1	0.524867496	8.66E-05
ENSMUSG00000038745	Nlrp6	0.524686986	0.000365283
ENSMUSG00000019890	Nts	0.524292562	2.37E-49
ENSMUSG00000031633	Slc25a4	0.524193484	8.46E-25
ENSMUSG00000021185	Dglucy	0.524043693	1.86E-54
ENSMUSG00000038976	Ppp1r9b	0.523830572	0.005901646
ENSMUSG00000059713	Rcan3	0.523616778	0.005690076
ENSMUSG00000052062	Pard3b	0.52349952	2.73E-15
ENSMUSG00000024947	Men1	0.523482486	0.003375855
ENSMUSG00000041235	Chd7	0.52340666	3.15E-07
ENSMUSG00000056162	Cndp1	0.522801928	0.047670499
ENSMUSG00000027739	Rab33b	0.521959716	0.024466871
ENSMUSG00000009035	Tmem184b	0.521858263	0.023198941
ENSMUSG00000027765	P2ry1	0.52132296	0.012876154
ENSMUSG00000021376	Tpmt	0.521136129	3.12E-23
ENSMUSG00000051316	Taf7	0.520214664	0.000471846
ENSMUSG00000054874	Pcnx3	0.519792526	0.00030282
ENSMUSG00000026918	Brd3	0.519290167	4.77E-05
ENSMUSG00000024778	Fas	0.518996353	2.14E-05
ENSMUSG00000023367	Tmem176a	0.518488273	0.018577836
ENSMUSG00000029994	Anxa4	0.51761099	4.36E-35
ENSMUSG00000023247	Guca2a	0.516346752	3.81E-59
ENSMUSG00000022057	Adamdec1	0.516257723	7.61E-06
ENSMUSG00000090213	Peds1	0.515811508	0.000367741
ENSMUSG00000051285	Pcmtd1	0.515629956	0.008499754
ENSMUSG00000041720	Pi4ka	0.515324916	0.005248254
ENSMUSG00000034947	Tmem106a	0.515158326	0.000552727
ENSMUSG00000056124	B4galt6	0.514630551	1.51E-10
ENSMUSG00000035413	Tmem98	0.514605082	0.000148222
ENSMUSG00000050777	Tmem37	0.514440128	2.07E-24
ENSMUSG00000027508	Pag1	0.51279411	0.009055767
ENSMUSG00000005836	Gata6	0.512573578	6.02E-07
ENSMUSG00000052560	Cpne8	0.512372819	7.88E-09
ENSMUSG00000033355	Rtp4	0.511908836	7.75E-10
ENSMUSG00000051615	Rap2a	0.511500521	3.12E-17
ENSMUSG00000028173	Wls	0.511478435	4.18E-05
ENSMUSG00000044352	Sowaha	0.511429933	1.27E-23
ENSMUSG00000035437	Rabgap1	0.510877974	0.000412556
ENSMUSG00000018501	Ncor1	0.510772512	0.013862877
ENSMUSG00000010609	Psen2	0.509187033	0.02359653
ENSMUSG00000015846	Rxra	0.509058176	3.26E-09
ENSMUSG00000031447	Lamp1	0.508639365	9.09E-11
ENSMUSG00000028799	Zfp362	0.508521336	0.026659763
ENSMUSG00000054723	Vmac	0.507192666	0.035123721
ENSMUSG00000022614	Lmf2	0.507014039	0.017029317
ENSMUSG00000028532	Cachd1	0.506494828	9.23E-17
ENSMUSG00000070047	Fat1	0.505942395	1.36E-13
ENSMUSG00000074305	Peak1	0.505712206	0.004234774
ENSMUSG00000041000	Trim62	0.505570502	0.000799537
ENSMUSG00000021097	Clmn	0.505554572	0.0004193
ENSMUSG00000022982	Sod1	0.505227181	6.50E-09
ENSMUSG00000006307	Kmt2b	0.504581155	0.001159611
ENSMUSG00000016283	H2-M2	0.504262255	0.0044366
ENSMUSG00000059866	Tnip2	0.503721092	0.025082927
ENSMUSG00000026924	Sec16a	0.503666438	0.006270663
ENSMUSG00000008136	Fhl2	0.503317077	0.007128531
ENSMUSG00000050786	Ccdc126	0.502250485	0.015845996
ENSMUSG00000085451	Gm40614	0.501978637	0.001684702
ENSMUSG00000040711	Sh3pxd2b	0.501944211	0.028016101
ENSMUSG00000025937	Lactb2	0.50153926	0.004497466
ENSMUSG00000074406	Zfp628	0.501086787	0.020461029
ENSMUSG00000027015	Cybrd1	0.501003312	0.004117035
ENSMUSG00000022751	Nit2	0.500305327	1.74E-09
ENSMUSG00000028082	Sh3d19	0.499597239	0.014335662
ENSMUSG00000064367	ND5	0.498200153	0.00118019
ENSMUSG00000029413	Naaa	0.497063181	0.038682435
ENSMUSG00000048109	Rbm15	0.496691446	0.035111488
ENSMUSG00000026399	Cd55	0.496388697	0.000128063
ENSMUSG00000032501	Trib1	0.495310651	2.62E-39
ENSMUSG00000025579	Gaa	0.495151365	0.006939633
ENSMUSG00000025047	Pdcd11	0.494953288	0.019765081
ENSMUSG00000047388	Atmin	0.494808872	8.58E-07
ENSMUSG00000030397	Mark4	0.493562159	2.16E-05
ENSMUSG00000023022	Lima1	0.493393562	0.00209822
ENSMUSG00000006538	Ihh	0.493261762	0.01134154
ENSMUSG00000062098	Btbd3	0.493038082	0.018768285
ENSMUSG00000070704	Ugt2b36	0.492863039	9.18E-06
ENSMUSG00000029162	Khk	0.492513969	2.67E-89
ENSMUSG00000042423	Fbrs	0.491353067	0.005930344
ENSMUSG00000028015	Ctso	0.490963563	1.13E-11
ENSMUSG00000030616	Sytl2	0.490129962	1.25E-08
ENSMUSG00000069729	Arid1b	0.490080764	0.00015871
ENSMUSG00000034413	Neurl1b	0.489801571	4.99E-05
ENSMUSG00000024727	Trpm6	0.489356488	2.98E-31
ENSMUSG00000028743	Akr7a5	0.488857419	6.92E-05
ENSMUSG00000028327	Stra6l	0.488740834	0.029706774
ENSMUSG00000038990	Cables2	0.488614504	0.001385068
ENSMUSG00000074994	Qser1	0.488454426	5.18E-07
ENSMUSG00000046876	Atxn1	0.488291544	6.53E-11
ENSMUSG00000051306	Usp42	0.488141727	0.03038984
ENSMUSG00000036565	Ttyh3	0.488064072	2.88E-18
ENSMUSG00000037622	Wdtc1	0.488018384	0.000183543
ENSMUSG00000033065	Pfkm	0.487887429	0.010977594
ENSMUSG00000025192	Entpd7	0.487772427	3.76E-06
ENSMUSG00000026131	Dst	0.487117585	0.032435817
ENSMUSG00000032816	Igdcc4	0.486994102	0.000735619
ENSMUSG00000039405	Prss23	0.486962094	1.00E-37
ENSMUSG00000063382	Bcl9l	0.486928235	0.000414182
ENSMUSG00000005125	Ndrg1	0.486568944	0.002352582
ENSMUSG00000021508	Cxcl14	0.484422554	0.04911688
ENSMUSG00000043940	Wdfy3	0.484313792	0.002093169
ENSMUSG00000022812	Gsk3b	0.483682968	9.83E-06
ENSMUSG00000042680	Garem1	0.483257645	7.73E-22
ENSMUSG00000041638	Gcn1	0.481646252	0.006223857
ENSMUSG00000009566	Fpgs	0.481496477	0.001428095
ENSMUSG00000036687	Tmem184a	0.481331003	0.031247801
ENSMUSG00000006362	Cbfa2t3	0.480356804	1.30E-07
ENSMUSG00000037487	Ubr5	0.48017772	4.53E-10
ENSMUSG00000020329	Polrmt	0.478946269	0.036190166
ENSMUSG00000042605	Atxn2	0.47891271	0.001150677
ENSMUSG00000063077	Kif1b	0.478899057	1.42E-09
ENSMUSG00000042744	Hectd4	0.478414024	0.017284334
ENSMUSG00000036985	Zdhhc9	0.47811731	0.006650662
ENSMUSG00000040511	Pvr	0.477878373	0.008462844
ENSMUSG00000021416	Eci3	0.477753638	6.03E-19
ENSMUSG00000067235	H2-Q10	0.476832649	2.48E-39

ENSMUSG00000036718	Micall2	-0.405915706	0.001765034
ENSMUSG00000035561	Aldh1b1	-0.404894901	3.93E-29
ENSMUSG00000014074	Rnf168	-0.404366689	0.00107887
ENSMUSG00000022235	Cmbl	-0.403504494	1.78E-05
ENSMUSG00000005103	Wdr1	-0.402697748	5.48E-20
ENSMUSG00000021250	Fos	-0.402666515	0.001948042
ENSMUSG00000032554	Trf	-0.402520384	3.28E-06
ENSMUSG00000027698	Nceh1	-0.402261665	4.72E-10
ENSMUSG00000036880	Acaa2	-0.400992182	1.96E-69
ENSMUSG00000036372	Tmem258	-0.400751322	0.015298986
ENSMUSG00000022180	Slc7a8	-0.400749152	1.64E-08
ENSMUSG00000020422	Tns3	-0.400066236	3.58E-08
ENSMUSG00000026994	Galnt3	-0.399181997	0.015694482
ENSMUSG00000030822	Prr14	-0.399079024	0.006212389
ENSMUSG00000031938	4931406C07	-0.39739348	1.37E-14
ENSMUSG00000025075	Habp2	-0.396413114	6.03E-07
ENSMUSG00000025544	Tm9sf2	-0.395216782	0.000162157
ENSMUSG00000073399	Trim40	-0.394870786	0.001581644
ENSMUSG00000060475	Wtap	-0.393607675	0.007698532
ENSMUSG00000020423	Btg2	-0.39358371	0.002679488
ENSMUSG00000034931	Dhx8	-0.393512089	0.000714428
ENSMUSG00000030539	Sema4b	-0.393203752	1.86E-06
ENSMUSG00000016206	H2-M3	-0.393185223	0.029616272
ENSMUSG00000041747	Utp15	-0.393058317	0.000180467
ENSMUSG00000007987	Ift22	-0.392797103	0.002178827
ENSMUSG00000040613	Apobec1	-0.392675358	4.83E-12
ENSMUSG00000037321	Tap1	-0.39235788	8.07E-13
ENSMUSG00000038240	Pdss2	-0.392349056	0.000457891
ENSMUSG00000116542	Cd200l2	-0.392241123	0.013784498
ENSMUSG00000006373	Pgrmc1	-0.392032575	6.76E-16
ENSMUSG00000029084	Cd38	-0.391608542	5.78E-18
ENSMUSG00000057719	Sh3rf2	-0.391140499	5.71E-05
ENSMUSG00000034450	Gulo	-0.390915004	0.02169546
ENSMUSG00000009585	Apobec3	-0.390623895	0.000667899
ENSMUSG00000021209	Ppp4r4	-0.390431043	1.40E-10
ENSMUSG00000078485	Plekhn1	-0.389743373	0.00198114
ENSMUSG00000027679	Dnajc19	-0.389669005	6.24E-07
ENSMUSG00000025194	Abcc2	-0.389467445	2.77E-11
ENSMUSG00000040945	Rcc2	-0.389342601	2.81E-26
ENSMUSG00000006299	Aamp	-0.389176166	0.005762682
ENSMUSG00000087022	9130024F11	-0.389026848	8.21E-05
ENSMUSG00000020826	Nos2	-0.388871612	2.90E-29
ENSMUSG00000015083	C8g	-0.388081987	2.96E-09
ENSMUSG00000031378	Abcd1	-0.387518069	0.005392166
ENSMUSG00000029120	Ppp2r2c	-0.387472804	0.035676384
ENSMUSG00000029445	Hpd	-0.386487779	0.004556115
ENSMUSG00000025409	Mbd6	-0.385990824	0.000223559
ENSMUSG00000058290	Espl1	-0.385804971	0.010685262
ENSMUSG00000033386	Frrs1	-0.385211302	4.46E-34
ENSMUSG00000056529	Ptafr	-0.385105356	1.92E-13
ENSMUSG00000002778	Kdelr1	-0.384419074	2.73E-10
ENSMUSG00000021906	Oxnad1	-0.384216717	2.27E-13
ENSMUSG00000037005	Xpnpep2	-0.383997413	3.72E-86
ENSMUSG00000024660	Incenp	-0.383830761	3.48E-05
ENSMUSG00000073411	H2-D1	-0.383701659	6.39E-06
ENSMUSG00000028656	Cap1	-0.38309077	0.000212026
ENSMUSG00000006736	Tspan31	-0.382794869	5.52E-05
ENSMUSG00000061762	Tac1	-0.382129637	0.007967163
ENSMUSG00000018659	Pnpo	-0.381109455	0.000316536
ENSMUSG00000041431	Ccnb1	-0.381054057	0.000331783
ENSMUSG00000031950	Gabarapl2	-0.380673817	5.42E-07
ENSMUSG00000032959	Pebp1	-0.380634329	0.011620851
ENSMUSG00000023004	Tuba1b	-0.380357835	0.001240041
ENSMUSG00000028102	Pex11b	-0.380153461	0.003419422
ENSMUSG00000058254	Tspan7	-0.380070773	1.85E-10
ENSMUSG00000020372	Rack1	-0.378662649	0.048021844
ENSMUSG00000118449	Gm3625	-0.378450902	0.013014001
ENSMUSG00000041362	Shtn1	-0.378307889	0.004063834
ENSMUSG00000051306	Usp42	-0.378273081	0.03038984
ENSMUSG00000079507	H2-Q1	-0.378039603	0.000937765
ENSMUSG00000055866	Per2	-0.376793209	0.001379975
ENSMUSG00000028207	Asph	-0.376567753	0.000594212
ENSMUSG00000056629	Fkbp2	-0.376198422	0.010618603
ENSMUSG00000038751	Ptk6	-0.375468386	5.14E-33
ENSMUSG00000036120	Rfxank	-0.37519041	0.034538216
ENSMUSG00000002625	Akap8l	-0.3748616	0.005549479
ENSMUSG00000030432	Rpl28	-0.374663101	1.69E-05
ENSMUSG00000003355	Fkbp11	-0.374630011	0.016382756
ENSMUSG00000026355	Mcm6	-0.374121491	0.002640015
ENSMUSG00000022108	Itm2b	-0.373081748	4.36E-12
ENSMUSG00000017837	Nkiras2	-0.372656975	0.001746391
ENSMUSG00000040204	Pclaf	-0.372406623	0.000144948
ENSMUSG00000075304	Sp5	-0.372377856	3.40E-06
ENSMUSG00000028992	Nmnat1	-0.371825414	8.29E-16
ENSMUSG00000039450	Dcxr	-0.371346184	0.011620851
ENSMUSG00000069917	Hba-a2	-0.371161688	0.041598768
ENSMUSG00000024335	Brd2	-0.370893514	0.000344449
ENSMUSG00000022769	Sdf2l1	-0.370893315	0.027177921
ENSMUSG00000036975	Tmem177	-0.370729649	0.008362682
ENSMUSG00000031551	Ido1	-0.370635859	7.30E-06
ENSMUSG00000046722	Cdc42se1	-0.370455036	0.034301603
ENSMUSG00000101031	Ms4a12	-0.37023352	2.63E-09
ENSMUSG00000048373	Fgfbp1	-0.368668515	7.61E-25
ENSMUSG00000021048	Mthfd1	-0.368307166	9.26E-05
ENSMUSG00000028860	Sytl1	-0.367843376	0.00066948
ENSMUSG00000001138	Cnnm3	-0.367808337	0.001252868
ENSMUSG00000031974	Abcb10	-0.367375349	8.64E-11
ENSMUSG00000040264	Gbp2b	-0.367125538	1.66E-11
ENSMUSG00000028603	Scp2	-0.367086396	3.48E-10
ENSMUSG00000032182	Yipf2	-0.366964058	0.002334584
ENSMUSG00000014867	Surf4	-0.366943249	2.16E-09
ENSMUSG00000040174	Alkbh3	-0.366538339	2.77E-05
ENSMUSG00000023829	Slc22a1	-0.366529424	1.25E-15
ENSMUSG00000073043	Atoh1	-0.366372922	0.000105556
ENSMUSG00000021518	Ptdss1	-0.365931531	3.80E-36
ENSMUSG00000029470	P2rx4	-0.365795986	0.004271155
ENSMUSG00000038991	Txndc5	-0.365773058	2.70E-06
ENSMUSG00000035847	Ids	-0.365425602	0.000120486
ENSMUSG00000030095	Tmem43	-0.365405951	0.001990421
ENSMUSG00000050627	Gpd1l	-0.365243792	1.92E-09
ENSMUSG00000026520	Pycr2	-0.364673271	0.000657124
ENSMUSG00000032679	Cd59a	-0.364249969	0.048790081
ENSMUSG00000004207	Psap	-0.364092441	0.00021099
ENSMUSG00000026202	Tuba4a	-0.363934217	4.47E-22
ENSMUSG00000021996	Esd	-0.363248541	8.36E-09
ENSMUSG00000020658	Efr3b	-0.363238705	2.40E-11
ENSMUSG00000041079	Rwdd2b	-0.363079056	0.000234264
ENSMUSG00000012443	Kif11	-0.363013248	0.023590132
ENSMUSG00000035236	Scai	-0.362946829	0.04902945
ENSMUSG00000027810	Eif2a	-0.362573134	0.000599811
ENSMUSG00000028885	Smpdl3b	-0.36220569	1.81E-13
ENSMUSG00000041718	Alg13	-0.361943815	0.026521418

ENSMUSG00000037344	Slc12a9	0.439654107	0.004342645
ENSMUSG00000025930	Msc	0.439229956	0.001677762
ENSMUSG00000030157	Clec2d	0.437534805	0.002699393
ENSMUSG00000031149	Praf2	0.437026951	4.57E-05
ENSMUSG00000047735	Samd9l	0.43695902	1.49E-10
ENSMUSG00000027639	Samhd1	0.434611847	2.01E-05
ENSMUSG00000034957	Cebpa	0.433821309	1.59E-06
ENSMUSG00000029646	Cdx2	0.43367661	0.033237767
ENSMUSG00000026568	Mpc2	0.432325223	3.65E-21
ENSMUSG00000029004	Kmt2e	0.432096097	1.76E-12
ENSMUSG00000006005	Tpr	0.431476826	1.26E-08
ENSMUSG00000000131	Xpo6	0.43146017	9.31E-06
ENSMUSG00000117284	Gm7072	0.431381275	0.013251376
ENSMUSG00000067942	Zfp160	0.431094296	0.019310557
ENSMUSG00000022629	Kif21a	0.430705459	2.83E-10
ENSMUSG00000037025	Foxa2	0.430472934	0.036544717
ENSMUSG00000020817	Rabep1	0.430156203	5.90E-06
ENSMUSG00000027002	Nckap1	0.429836009	0.001750757
ENSMUSG00000033854	Kcnk10	0.429697595	1.45E-18
ENSMUSG00000078920	Ifi47	0.429249317	1.89E-22
ENSMUSG00000055053	Nfic	0.42915742	1.90E-09
ENSMUSG00000029596	Sdsl	0.429113071	1.76E-06
ENSMUSG00000031960	Aars	0.428279244	0.000532479
ENSMUSG00000029190	D5Ertd579e	0.427956096	9.19E-13
ENSMUSG00000051079	Rgs13	0.427924631	7.39E-06
ENSMUSG00000120113	Fam120aos	0.427877448	0.005286484
ENSMUSG00000024947	Men1	0.427754809	0.003375855
ENSMUSG00000063273	Naa15	0.427024908	0.001708753
ENSMUSG00000031431	Tsc22d3	0.426792891	1.03E-07
ENSMUSG00000048922	Cdca2	0.426216303	0.006235582
ENSMUSG00000037279	Ovol2	0.426063276	0.000473195
ENSMUSG00000026576	Atp1b1	0.425524439	2.50E-07
ENSMUSG00000071064	Zfp827	0.425219149	9.83E-05
ENSMUSG00000063179	Pstk	0.424994661	2.41E-05
ENSMUSG00000068130	Zfp442	0.424347658	4.70E-08
ENSMUSG00000068267	Cenpb	0.423889143	0.013102438
ENSMUSG00000032403	2300009A05	0.423819114	0.001737794
ENSMUSG00000031286	Glt28d2	0.423259843	0.002832508
ENSMUSG00000028356	Ambp	0.422321786	9.96E-11
ENSMUSG00000024997	Prdx3	0.422025093	7.89E-06
ENSMUSG00000039262	Prrc2b	0.421126106	0.002106378
ENSMUSG00000033065	Pfkm	0.420396627	0.010977594
ENSMUSG00000023960	Enpp5	0.419857539	3.10E-06
ENSMUSG00000068141	Gm10232	0.41971611	0.031417028
ENSMUSG00000042744	Hectd4	0.41963286	0.017284334
ENSMUSG00000070713	Hmgn2-ps	0.418985083	0.037299163
ENSMUSG00000038305	Spats2l	0.418053805	0.003744916
ENSMUSG00000040479	Dgkz	0.417938714	1.12E-09
ENSMUSG00000004952	Rasa4	0.417792398	0.030199975
ENSMUSG00000078185	Chml	0.416764688	0.005058815
ENSMUSG00000029482	Aacs	0.416696581	1.03E-11
ENSMUSG00000009376	Met	0.416443377	0.021765742
ENSMUSG00000038507	Parp12	0.416230509	2.19E-13
ENSMUSG00000021494	Ddx41	0.416166912	0.009364853
ENSMUSG00000024292	Cyp4f14	0.415941988	0.001024626
ENSMUSG00000025047	Pdcd11	0.415761615	0.019765081
ENSMUSG00000017478	Zc3h18	0.415717895	0.000424472
ENSMUSG00000031996	Aplp2	0.415382109	0.001557951
ENSMUSG00000051285	Pcmtd1	0.415243357	0.008499754
ENSMUSG00000052609	Plekhg3	0.414673365	0.000829264
ENSMUSG00000078570	1110065P20	0.414247598	4.84E-06
ENSMUSG00000033429	Mcee	0.412434284	0.008082177
ENSMUSG00000025762	Larp1b	0.412249979	0.007133454
ENSMUSG00000042608	Stk40	0.41189733	3.22E-05
ENSMUSG00000037465	Klf10	0.411249403	0.034550032
ENSMUSG00000020978	Klhdc2	0.411138807	1.42E-07
ENSMUSG00000040111	Gramd1b	0.410251775	2.02E-12
ENSMUSG00000030335	Mrpl51	0.410123844	0.001085329
ENSMUSG00000100593	1700119H24	0.410091913	0.044566267
ENSMUSG00000032621	Srek1	0.409765861	0.000767964
ENSMUSG00000003352	Cacnb3	0.40969228	3.59E-05
ENSMUSG00000039842	Mcph1	0.409093638	0.000121982
ENSMUSG00000030102	Itpr1	0.40885476	2.46E-52
ENSMUSG00000030849	Fgfr2	0.408294709	0.017722889
ENSMUSG00000078780	Gm5150	0.408022893	0.004342645
ENSMUSG00000022797	Tfrc	0.407935129	0.002726922
ENSMUSG00000032978	Guca2b	0.407529337	9.66E-17
ENSMUSG00000036990	Otud4	0.406759836	8.37E-11
ENSMUSG00000003452	Bicd1	0.406581209	5.90E-07
ENSMUSG00000046410	Kcnk6	0.405388726	7.37E-05
ENSMUSG00000024661	Fth1	0.405180809	2.60E-83
ENSMUSG00000024921	Smarca2	0.404915767	6.70E-18
ENSMUSG00000040483	Xaf1	0.404908218	1.36E-20
ENSMUSG00000049422	Chchd10	0.404749532	2.18E-34
ENSMUSG00000111080	Gm20300	0.404699521	0.004892759
ENSMUSG00000046982	Tshz1	0.404222924	1.70E-07
ENSMUSG00000020105	Lrig3	0.403637242	8.49E-08
ENSMUSG00000025245	Lztfl1	0.403177296	0.032752455
ENSMUSG00000000295	Hddc2	0.40291011	0.009036192
ENSMUSG00000029461	Fam168a	0.402841788	0.020154342
ENSMUSG00000036580	Spart	0.402636657	5.89E-09
ENSMUSG00000030231	Plekha5	0.402275014	0.002866355
ENSMUSG00000063253	Scoc	0.402072574	2.10E-26
ENSMUSG00000030094	Xpc	0.402009085	0.028064487
ENSMUSG00000038002	Cramp1	0.401174779	2.22E-05
ENSMUSG00000038020	Rapgefl1	0.401113764	0.040524515
ENSMUSG00000027739	Rab33b	0.400634539	0.024466871
ENSMUSG00000037071	Scd1	0.40060795	0.000101389
ENSMUSG00000068580	Zfyve19	0.400296927	0.000941387
ENSMUSG00000027667	Zfp639	0.399952671	0.018241225
ENSMUSG00000017188	Coa3	0.399576003	2.33E-08
ENSMUSG00000035473	Galm	0.399474106	1.13E-38
ENSMUSG00000025790	Slco3a1	0.399219384	0.000250395
ENSMUSG00000032525	Nktr	0.398969412	0.01401643
ENSMUSG00000035529	Prdm4	0.398185062	0.028233022
ENSMUSG00000040407	Akap9	0.397604939	3.91E-09
ENSMUSG00000024378	Stard4	0.397538657	9.73E-20
ENSMUSG00000042590	Ipo11	0.396178895	0.049061965
ENSMUSG00000027272	Ubr1	0.396030593	0.027019826
ENSMUSG00000041782	Lad1	0.395729479	8.38E-05
ENSMUSG00000066640	Fbxl18	0.394390141	0.000430141
ENSMUSG00000014075	Dynlt2b	0.394343882	5.54E-11
ENSMUSG00000003810	Mast2	0.393915777	2.01E-07
ENSMUSG00000028261	Ndufaf4	0.392422541	2.57E-12
ENSMUSG00000022521	Crebbp	0.392265859	1.16E-09
ENSMUSG00000050592	Fam78a	0.392090794	0.018881599
ENSMUSG00000015962	1700016C15	0.390814656	5.90E-06
ENSMUSG00000034158	Lrrc58	0.390445678	0.000789598
ENSMUSG00000042043	Tbca	0.390378301	1.34E-07
ENSMUSG00000008575	Nfib	0.389335077	4.44E-06
ENSMUSG00000032309	Fbxo22	0.389243564	0.000102949
ENSMUSG00000018377	Vezf1	0.38919442	0.001832741

ENSMUSG00000024313	Mep1b	-0.390680986	0.0048394
ENSMUSG00000026319	Relch	-0.390409195	0.048517154
ENSMUSG00000042195	Slc35f2	-0.390302397	0.002738082
ENSMUSG00000113769	5033406O09	-0.390220275	1.84E-10
ENSMUSG00000047844	Bex4	-0.389982719	2.25E-26
ENSMUSG00000029474	Rnf34	-0.38978548	0.00029908
ENSMUSG00000032575	Manf	-0.388912663	0.002983244
ENSMUSG00000032589	Bsn	-0.38871653	6.60E-05
ENSMUSG00000022792	Yars2	-0.388209639	0.04409293
ENSMUSG00000039670	Oxld1	-0.388171959	0.00144598
ENSMUSG00000022434	Fam118a	-0.388115622	2.86E-05
ENSMUSG00000030062	Rpn1	-0.387696233	3.45E-05
ENSMUSG00000006699	Cdc42	-0.387391729	5.13E-07
ENSMUSG00000030717	Nupr1	-0.386889355	3.31E-34
ENSMUSG00000028312	Smc2	-0.38584498	8.11E-05
ENSMUSG00000040339	Eeig2	-0.385789601	6.34E-34
ENSMUSG00000020464	Pnpt1	-0.38548452	0.002881478
ENSMUSG00000027007	Itprid2	-0.385403253	0.002676969
ENSMUSG00000026014	Raph1	-0.384839596	4.70E-14
ENSMUSG00000002881	Nab1	-0.383265618	0.005668452
ENSMUSG00000054161	Fam83e	-0.383070603	1.16E-28
ENSMUSG00000028749	Pla2g2f	-0.382004081	0.01057116
ENSMUSG00000118894	Gm24424	-0.381904149	0.019336282
ENSMUSG00000064181	Rab3ip	-0.381791913	1.78E-07
ENSMUSG00000032199	Polr2m	-0.381711891	0.009282081
ENSMUSG00000023939	Mrpl14	-0.381092459	0.000141232
ENSMUSG00000035900	Gramd4	-0.380592949	0.007532154
ENSMUSG00000042225	Ammecr1	-0.380082439	3.62E-07
ENSMUSG00000041272	Tox	-0.379456066	6.94E-06
ENSMUSG00000035775	Krt20	-0.379232564	0.002424176
ENSMUSG00000003868	Ruvbl2	-0.3788898	0.040095291
ENSMUSG00000031156	Slc35a2	-0.378467354	3.55E-08
ENSMUSG00000024590	Lmnb1	-0.378416464	2.50E-20
ENSMUSG00000025357	Dgka	-0.37768828	3.59E-11
ENSMUSG00000118038	Gm9895	-0.377417445	0.000477359
ENSMUSG00000010406	Mrpl52	-0.376998702	1.53E-07
ENSMUSG00000014633	Cmc2	-0.37539048	0.030539428
ENSMUSG00000002489	Tiam1	-0.374160928	0.00032707
ENSMUSG00000042272	Sestd1	-0.373878873	0.004143347
ENSMUSG00000021832	Psmc6	-0.373202108	0.00416813
ENSMUSG00000036513	Commd2	-0.37297022	5.09E-06
ENSMUSG00000038209	Itln1	-0.372905418	4.92E-08
ENSMUSG00000034789	Rab24	-0.372836768	0.020679189
ENSMUSG00000019818	Cd164	-0.372356318	6.04E-10
ENSMUSG00000030610	Det1	-0.372133695	0.001473462
ENSMUSG00000038332	Sesn1	-0.370814492	0.03888568
ENSMUSG00000078566	Bnip3	-0.370056332	0.000462906
ENSMUSG00000020018	Snrpf	-0.36997036	0.018684488
ENSMUSG00000026222	Sp100	-0.369747929	1.30E-17
ENSMUSG00000019432	Ddx39b	-0.369687753	0.000785021
ENSMUSG00000021748	Pdhb	-0.368855239	8.52E-11
ENSMUSG00000018965	Ywhah	-0.368567446	0.040353325
ENSMUSG00000023908	Pkmyt1	-0.368551832	0.010111192
ENSMUSG00000067219	Nipal1	-0.368128431	4.62E-13
ENSMUSG00000060208	Defa17	-0.367779817	0.035080957
ENSMUSG00000030089	Slc41a3	-0.36762395	1.41E-14
ENSMUSG00000021831	Ero1a	-0.367410848	0.009410071
ENSMUSG00000031429	Psmd10	-0.367037989	0.000141134
ENSMUSG00000023886	Smoc2	-0.366852165	0.009580071
ENSMUSG00000033998	Kcnk1	-0.366823168	2.43E-11
ENSMUSG00000004880	Lbr	-0.366623884	0.000264466
ENSMUSG00000068011	Mkrn2os	-0.366244004	0.00103785
ENSMUSG00000033813	Tcea1	-0.36622276	0.002840411
ENSMUSG00000054091	1810037I17F	-0.365855181	5.97E-06
ENSMUSG00000050043	Tmx2	-0.365648565	0.00107871
ENSMUSG00000051413	Plagl2	-0.365546664	0.049406237
ENSMUSG00000020476	Dbnl	-0.365412724	9.13E-06
ENSMUSG00000006641	Slc5a6	-0.365282989	2.09E-57
ENSMUSG00000032261	Sh3bgrl2	-0.365203108	8.66E-50
ENSMUSG00000028693	Nasp	-0.364738867	0.000165444
ENSMUSG00000021670	Hmgcr	-0.364516246	4.69E-10
ENSMUSG00000022971	Ifnar2	-0.364012198	0.000229063
ENSMUSG00000026107	Nabp1	-0.363295301	0.000382748
ENSMUSG00000025366	Esyt1	-0.362780238	0.03367502
ENSMUSG00000038507	Parp12	-0.362521658	2.19E-13
ENSMUSG00000002102	Psmc3	-0.362396009	0.002678155
ENSMUSG00000035692	Isg15	-0.362319805	1.10E-14
ENSMUSG00000024725	Ostf1	-0.362306594	0.036777447
ENSMUSG00000025817	Nudt5	-0.362254012	0.001599213
ENSMUSG00000032548	Slco2a1	-0.362140344	1.49E-09
ENSMUSG00000031922	Cep57	-0.362025222	0.024589241
ENSMUSG00000047205	Dusp18	-0.359652124	0.020127666
ENSMUSG00000062867	Impdh2	-0.359340089	4.83E-08
ENSMUSG00000034349	Smc4	-0.359326974	0.002717496
ENSMUSG00000055681	Cope	-0.35888941	7.16E-06
ENSMUSG00000075229	Mix23	-0.358848202	1.70E-05
ENSMUSG00000031163	Glod5	-0.358387644	4.42E-28
ENSMUSG00000024712	Rfk	-0.357990643	0.000365595
ENSMUSG00000113902	Ndufb1	-0.35772393	2.72E-06
ENSMUSG00000039745	Htatip2	-0.357277545	2.23E-15
ENSMUSG00000027808	Serp1	-0.356658403	0.000500317
ENSMUSG00000114003	Gm9616	-0.356418987	0.03757242
ENSMUSG00000036989	Adam10	-0.356368973	4.66E-08
ENSMUSG00000063953	Amd2	-0.355007038	0.011130369
ENSMUSG00000002014	Ssr4	-0.354841548	1.10E-05
ENSMUSG00000028639	Ybx1	-0.35446455	0.006788561
ENSMUSG00000023909	Paqr4	-0.353905274	1.07E-24
ENSMUSG00000041084	Ostc	-0.353509518	0.000103815
ENSMUSG00000024795	Kif20b	-0.353195464	1.79E-06
ENSMUSG00000044707	Ccnjl	-0.353194681	0.028393642
ENSMUSG00000027395	Polr1b	-0.351465813	0.017297188
ENSMUSG00000032908	Sgpp2	-0.351188732	0.021396842
ENSMUSG00000024331	Dsc2	-0.350232438	0.000569235
ENSMUSG00000061111	Mcrip1	-0.350178469	0.043204363
ENSMUSG00000033200	Tpsg1	-0.349542661	0.012744647
ENSMUSG00000027286	Lrrc57	-0.349321846	6.92E-07
ENSMUSG00000032046	Abhd12	-0.349304269	0.000536892
ENSMUSG00000023805	Synj2	-0.349089938	0.000299517
ENSMUSG00000065259	Snora30	-0.348362967	0.00712989
ENSMUSG00000003382	Etv3	-0.348294178	0.004127172
ENSMUSG00000021893	Capn7	-0.348251133	0.000327057
ENSMUSG00000070923	Klhl9	-0.347744375	7.14E-09
ENSMUSG00000025645	Ccdc51	-0.347733322	0.000657803
ENSMUSG00000024486	Hbegf	-0.347340574	0.004739645
ENSMUSG00000021054	Sgpp1	-0.347067403	0.001245323
ENSMUSG00000059734	Ndufs8	-0.347022685	1.56E-06
ENSMUSG00000039456	Morc3	-0.346888506	0.005520237
ENSMUSG00000040170	Fmo2	-0.346744634	6.39E-09
ENSMUSG00000030662	Ipo5	-0.346379338	1.80E-05
ENSMUSG00000027108	Ola1	-0.346289067	0.036211525
ENSMUSG00000014226	Cacybp	-0.345953803	0.004743943
ENSMUSG00000002345	Borcs8	-0.345914476	0.022362873

ENSMUSG00000001435	Col18a1	0.476460479	0.00018191
ENSMUSG00000039678	Tbc1d13	0.476302794	0.014029269
ENSMUSG00000021277	Traf3	0.475968662	0.019982045
ENSMUSG00000021751	Acox2	0.475761686	0.01180493
ENSMUSG00000040740	Slc25a34	0.475629644	0.000414469
ENSMUSG00000005615	Pcyt1a	0.474855598	2.93E-15
ENSMUSG00000027272	Ubr1	0.474524179	0.027019826
ENSMUSG00000020868	Xylt2	0.474128817	0.001967403
ENSMUSG00000059409	Ppp2r5d	0.472866694	4.71E-08
ENSMUSG00000026269	Rnpepl1	0.471781532	0.001623468
ENSMUSG00000070574	2310016G11	0.47140989	0.028623231
ENSMUSG00000021900	Btd	0.470971362	0.026185226
ENSMUSG00000004846	Plod3	0.470772362	0.042233477
ENSMUSG00000039100	Marchf6	0.470561177	9.78E-07
ENSMUSG00000062624	Cyp2c67	0.470296325	0.007954786
ENSMUSG00000040505	Abcg5	0.469462689	9.88E-12
ENSMUSG00000048826	Dact2	0.469137077	0.006386617
ENSMUSG00000034248	Slc25a37	0.468716115	4.34E-13
ENSMUSG00000004814	Ccl24	0.468522974	2.10E-31
ENSMUSG00000025571	Tnrc6c	0.468458657	7.22E-08
ENSMUSG00000005621	Zfp592	0.468320953	0.014355654
ENSMUSG00000071855	Ccdc112	0.467803735	0.012469257
ENSMUSG00000044042	Fmn1	0.467433549	2.78E-05
ENSMUSG00000020277	Pfkl	0.466631764	0.002140552
ENSMUSG00000033671	Cep350	0.466524958	0.003242197
ENSMUSG00000030747	Dgat2	0.466493151	9.58E-07
ENSMUSG00000031706	Rfx1	0.465726578	7.50E-07
ENSMUSG00000070643	Sox13	0.465013074	7.45E-15
ENSMUSG00000028464	Tpm2	0.464214695	0.010603278
ENSMUSG00000004366	Sst	0.464077717	3.37E-45
ENSMUSG00000005142	Man2b1	0.463918993	1.20E-05
ENSMUSG00000029004	Kmt2e	0.463708812	1.76E-12
ENSMUSG00000008575	Nfib	0.463345165	4.44E-06
ENSMUSG00000039218	Srrm2	0.463168685	4.27E-09
ENSMUSG00000031502	Col4a1	0.462620797	0.007133387
ENSMUSG00000031832	Taf1c	0.462609122	0.012161377
ENSMUSG00000053205	Styx	0.46227777	0.031809287
ENSMUSG00000070808	Bicra	0.460913886	9.18E-05
ENSMUSG00000020140	Lgr5	0.459879904	1.22E-05
ENSMUSG00000060012	Kif13b	0.459808079	0.034275011
ENSMUSG00000037992	Rara	0.459649613	0.030229682
ENSMUSG00000022048	Dpysl2	0.459503701	0.000855807
ENSMUSG00000023914	Mep1a	0.459435357	9.65E-151
ENSMUSG00000030629	Zfand6	0.45825131	1.58E-09
ENSMUSG00000039782	Cpeb2	0.457471898	0.015669224
ENSMUSG00000028655	Mfsd2a	0.457322154	1.83E-15
ENSMUSG00000021127	Zfp36l1	0.457292868	0.000231027
ENSMUSG00000045886	Pam16l	0.45717433	0.001267821
ENSMUSG00000017493	Igfbp4	0.457065456	3.94E-05
ENSMUSG00000028278	Rragd	0.45587056	6.73E-17
ENSMUSG00000046020	Pofut1	0.455334235	0.00173483
ENSMUSG00000027546	Atp9a	0.455245618	9.73E-07
ENSMUSG00000071203	Naip5	0.455105022	1.70E-29
ENSMUSG00000074738	Fndc10	0.454777737	6.37E-14
ENSMUSG00000073889	Il11ra1	0.454713802	0.016821364
ENSMUSG00000019838	Slc16a10	0.45454814	6.29E-06
ENSMUSG00000031788	Kifc3	0.453800713	0.003092098
ENSMUSG00000027371	Fahd2a	0.453745722	1.46E-05
ENSMUSG00000037098	Rab11fip3	0.452880347	0.024529872
ENSMUSG00000042535	Gtpbp1	0.452722786	0.000126167
ENSMUSG00000027188	Pamr1	0.452160579	0.007556482
ENSMUSG00000018334	Ksr1	0.452055985	0.000118806
ENSMUSG00000028636	Ppcs	0.451451383	0.000706544
ENSMUSG00000054469	Lclat1	0.450813096	5.31E-06
ENSMUSG00000040782	Cop1	0.450517555	0.007216364
ENSMUSG00000004071	Cdip1	0.450190952	8.22E-25
ENSMUSG00000031996	Aplp2	0.449786349	0.001557951
ENSMUSG00000038831	Ralgps1	0.449636947	1.14E-09
ENSMUSG00000022371	Col14a1	0.448943763	6.10E-05
ENSMUSG00000047710	Champ1	0.44884667	0.000846162
ENSMUSG00000021690	Jmy	0.448543706	0.000711404
ENSMUSG00000035632	Cnot3	0.448464586	0.00421901
ENSMUSG00000021665	Hexb	0.448299502	0.000391653
ENSMUSG00000028709	Mob3c	0.44806256	0.014260989
ENSMUSG00000022479	Vdr	0.44767009	1.35E-10
ENSMUSG00000039841	Zfp800	0.447496899	0.006223857
ENSMUSG00000042096	Dao	0.447330739	4.44E-42
ENSMUSG00000050520	Cldn8	0.446856349	2.37E-07
ENSMUSG00000058886	Deaf1	0.446667773	0.031270982
ENSMUSG00000021417	Eci2	0.446320749	0.001073064
ENSMUSG00000034453	Polr3b	0.445470151	0.030134072
ENSMUSG00000033427	Upb1	0.445280118	1.56E-33
ENSMUSG00000032449	Slc25a36	0.445020799	5.73E-11
ENSMUSG00000040687	Madd	0.444907956	0.000127325
ENSMUSG00000010045	Tmem115	0.443787369	0.048090968
ENSMUSG00000058672	Tubb2a	0.443674662	1.24E-06
ENSMUSG00000033308	Dpyd	0.443579039	3.75E-05
ENSMUSG00000030852	Tacc2	0.44340345	2.63E-06
ENSMUSG00000076617	Ighm	0.442738215	0.027363156
ENSMUSG00000032083	Apoa1	0.442017598	2.28E-11
ENSMUSG00000031983	2310022B05I	0.441923531	0.017128529
ENSMUSG00000038914	Dido1	0.441661675	0.040505462
ENSMUSG00000023019	Gpd1	0.441532628	1.67E-39
ENSMUSG00000038611	Phrf1	0.441498113	0.024130871
ENSMUSG00000054199	Gon4l	0.441145323	0.001940984
ENSMUSG00000028161	Ppp3ca	0.440508555	4.79E-12
ENSMUSG00000042472	Zfp410	0.439651902	1.32E-07
ENSMUSG00000097222	Gata6os	0.439548802	0.001122192
ENSMUSG00000031451	Gas6	0.439319934	1.30E-11
ENSMUSG00000039089	L3mbtl3	0.43926801	0.040776418
ENSMUSG00000025041	Nt5c2	0.439254106	0.000105358
ENSMUSG00000029188	Slc34a2	0.439206506	7.32E-69
ENSMUSG00000024393	Prrc2a	0.438833542	3.79E-05
ENSMUSG00000058022	Adtrp	0.438695729	3.49E-09
ENSMUSG00000068284	Usf3	0.437825191	0.001926304
ENSMUSG00000069917	Hba-a2	0.43777305	0.041598768
ENSMUSG00000000385	Tmprss2	0.437572533	9.72E-21
ENSMUSG00000028920	Fbxo42	0.437415406	0.007848567
ENSMUSG00000028977	Casz1	0.43609108	1.14E-07
ENSMUSG00000014426	Map3k4	0.435251502	0.026725332
ENSMUSG00000075704	Txnrd2	0.434916197	0.006032896
ENSMUSG00000042367	Gjb3	0.434270881	2.69E-25
ENSMUSG00000021518	Ptdss1	0.432613329	3.80E-36
ENSMUSG00000036968	Cnpy4	0.431779556	0.046330648
ENSMUSG00000049354	Dcaf7	0.431562564	0.00128914
ENSMUSG00000033059	Pygb	0.431311751	0.001504114
ENSMUSG00000042359	Osbpl6	0.431275991	1.13E-10
ENSMUSG00000035569	Ankrd11	0.431200671	1.49E-10
ENSMUSG00000047866	Lonp2	0.430626952	4.54E-12
ENSMUSG00000045576	St7l	0.430136167	0.003982036
ENSMUSG00000034108	Ccs	0.430056304	0.005977972
ENSMUSG00000037022	Mmaa	0.429728957	3.43E-09

ENSMUSG00000051483	Cbr1	-0.36156249	3.42E-58
ENSMUSG00000032218	Ccnb2	-0.360958627	0.016051
ENSMUSG00000002983	Relb	-0.360407563	5.64E-22
ENSMUSG00000027333	Smox	-0.360360254	4.87E-18
ENSMUSG00000059714	Flot1	-0.360033468	0.022087364
ENSMUSG00000069744	Psmb3	-0.359963356	0.003781037
ENSMUSG00000042595	Fam199x	-0.359916516	3.04E-05
ENSMUSG00000024866	Acy3	-0.359895666	4.33E-10
ENSMUSG00000002055	Spag5	-0.359488993	0.000393845
ENSMUSG00000025484	Bet1l	-0.358683411	1.78E-05
ENSMUSG00000060376	Bckdha	-0.358227034	0.00129736
ENSMUSG00000036943	Rab8b	-0.358114911	0.003773892
ENSMUSG00000032067	Pts	-0.358031867	0.006201742
ENSMUSG00000027533	Fabp5	-0.357708326	7.23E-05
ENSMUSG00000047407	Tgif1	-0.357675551	3.19E-07
ENSMUSG00000030545	Pex11a	-0.357614699	5.26E-14
ENSMUSG00000042129	Rassf4	-0.357521213	3.61E-13
ENSMUSG00000026971	Itgb6	-0.357041835	3.61E-06
ENSMUSG00000028124	Gclm	-0.35692372	6.95E-12
ENSMUSG00000031930	Wwp2	-0.354272344	0.0023727
ENSMUSG00000019868	Vta1	-0.354091492	0.000150254
ENSMUSG00000020097	Sgpl1	-0.353748861	0.011283469
ENSMUSG00000043003	Rasef	-0.353416464	0.029334695
ENSMUSG00000027323	Rad51	-0.35228921	0.013561666
ENSMUSG00000020520	Galnt10	-0.352251533	0.012322259
ENSMUSG00000028671	Gale	-0.352052122	7.57E-09
ENSMUSG00000042487	Leo1	-0.352049219	0.00686984
ENSMUSG00000026692	Fmo4	-0.35154586	0.005135732
ENSMUSG00000021379	Id4	-0.351406802	0.002088176
ENSMUSG00000026289	Atg16l1	-0.350919022	0.001333094
ENSMUSG00000020018	Snrpf	-0.350667491	0.018684488
ENSMUSG00000026688	Mgst3	-0.350422484	1.11E-16
ENSMUSG00000073705	Cenps	-0.350243525	0.012945209
ENSMUSG00000023505	Cdca3	-0.35022289	4.01E-05
ENSMUSG00000046062	Ppp1r15b	-0.350205934	1.29E-06
ENSMUSG00000030298	Sec13	-0.350146855	3.63E-14
ENSMUSG00000024150	Mcfd2	-0.349765119	1.88E-09
ENSMUSG00000050335	Lgals3	-0.349760829	2.71E-14
ENSMUSG00000045928	4933440M02	-0.34930469	1.37E-07
ENSMUSG00000028405	Aco1	-0.349296443	0.001654081
ENSMUSG00000030281	Il17rc	-0.347617844	7.16E-09
ENSMUSG00000021484	Lman2	-0.347541769	0.005513403
ENSMUSG00000079480	Pin4	-0.347494236	0.002995628
ENSMUSG00000042472	Zfp410	-0.347412278	1.32E-07
ENSMUSG00000019066	Rab3d	-0.347348429	3.02E-16
ENSMUSG00000024132	Eci1	-0.34701758	1.53E-07
ENSMUSG00000039100	Marchf6	-0.346483165	9.78E-07
ENSMUSG00000027506	Tpd52	-0.346375898	0.004273545
ENSMUSG00000028691	Prdx1	-0.346349786	0.006194536
ENSMUSG00000028256	Odf2l	-0.346233406	5.85E-07
ENSMUSG00000046908	Ltb4r1	-0.346193739	0.014592683
ENSMUSG00000023259	Slc26a6	-0.346101433	2.23E-19
ENSMUSG00000019558	Slc6a8	-0.346099205	0.016400404
ENSMUSG00000052406	Rexo4	-0.345921805	0.037490416
ENSMUSG00000030823	9130019O22	-0.34580237	0.000591956
ENSMUSG00000010122	Slc47a1	-0.345512479	3.99E-12
ENSMUSG00000042712	Tceal9	-0.345173164	3.37E-21
ENSMUSG00000047250	Ptgs1	-0.345038575	0.003457643
ENSMUSG00000024074	Crim1	-0.344469097	2.93E-05
ENSMUSG00000056899	Immp2l	-0.344092687	1.08E-05
ENSMUSG00000020733	Nherf1	-0.343676282	2.62E-07
ENSMUSG00000014243	Zswim7	-0.343613214	8.84E-07
ENSMUSG00000031156	Slc35a2	-0.343554589	3.55E-08
ENSMUSG00000029186	Pi4k2b	-0.343095661	8.38E-10
ENSMUSG00000029777	Gars	-0.342865395	5.66E-05
ENSMUSG00000021798	Ldb3	-0.341974906	1.03E-13
ENSMUSG00000061950	Ppp4r1	-0.341690645	0.001581674
ENSMUSG00000033634	Nat8f2	-0.341617438	2.69E-05
ENSMUSG00000044860	Gm1123	-0.340632347	4.99E-07
ENSMUSG00000034757	Tmub2	-0.340394843	0.026608554
ENSMUSG00000039953	Clstn1	-0.340363775	1.76E-12
ENSMUSG00000074259	Gramd2a	-0.339877487	0.00254277
ENSMUSG00000024590	Lmnb1	-0.339789046	2.50E-20
ENSMUSG00000039910	Cited2	-0.339397737	0.000100871
ENSMUSG00000041845	Rhod	-0.339055536	4.64E-10
ENSMUSG00000070643	Sox13	-0.338276898	7.45E-15
ENSMUSG00000030707	Coro1a	-0.337737749	5.38E-05
ENSMUSG00000003235	Eif2b5	-0.336782089	0.021976795
ENSMUSG00000028415	Spink4	-0.336651245	0.000198568
ENSMUSG00000038828	Tmem214	-0.336241349	0.001099444
ENSMUSG00000037926	Ssh2	-0.336063832	0.000414242
ENSMUSG00000027775	Mfsd1	-0.335804445	3.08E-05
ENSMUSG00000013878	Rnf170	-0.334989846	0.001649073
ENSMUSG00000029076	Sdf4	-0.334968198	3.03E-09
ENSMUSG00000025239	Limd1	-0.334719692	3.19E-20
ENSMUSG00000074207	Adh1	-0.334451258	1.59E-168
ENSMUSG00000044768	Macir	-0.334356546	0.00460451
ENSMUSG00000025467	Prap1	-0.333726427	5.66E-85
ENSMUSG00000024425	Ndfip1	-0.333566586	0.017649881
ENSMUSG00000067274	Rplp0	-0.33331866	0.008059402
ENSMUSG00000019822	Smpd2	-0.333267414	0.006475576
ENSMUSG00000045636	Mtus1	-0.333259051	0.010166753
ENSMUSG00000020444	Guk1	-0.333196608	4.59E-28
ENSMUSG00000040329	Il7	-0.332883904	0.002602967
ENSMUSG00000019158	Tmem160	-0.332554482	1.10E-33
ENSMUSG00000073802	Cdkn2b	-0.332553259	4.20E-05
ENSMUSG00000025793	Hgs	-0.332352623	0.001014632
ENSMUSG00000002064	Sdf2	-0.3322098	0.000274522
ENSMUSG00000046865	Fbl	-0.332185954	0.025541177
ENSMUSG00000020250	Txnrd1	-0.331730311	1.37E-09
ENSMUSG00000004035	Gstm7	-0.33171763	7.67E-36
ENSMUSG00000030082	Sec61a1	-0.331620156	1.49E-05
ENSMUSG00000022226	Mcpt2	-0.33116878	0.001094271
ENSMUSG00000025747	Tyms	-0.330897089	0.020395193
ENSMUSG00000031621	Isx	-0.330202962	2.79E-06
ENSMUSG00000019278	Dpep1	-0.329929111	1.01E-17
ENSMUSG00000001436	Slc19a1	-0.329571816	2.30E-10
ENSMUSG00000055301	Adh7	-0.329484572	3.21E-24
ENSMUSG00000003346	Abhd17a	-0.328257456	5.08E-06
ENSMUSG00000015711	Prune1	-0.328172169	1.31E-10
ENSMUSG00000034636	Zyg11b	-0.327632457	0.001308283
ENSMUSG00000032226	Gcnt3	-0.327507589	8.39E-13
ENSMUSG00000074345	Tnfaip8l3	-0.32748873	0.048922138
ENSMUSG00000026278	Bok	-0.327295084	1.71E-05
ENSMUSG00000025130	P4hb	-0.327024412	1.23E-36
ENSMUSG00000049382	Krt8	-0.326581056	3.59E-15
ENSMUSG00000058997	Vwa8	-0.3265088	3.55E-08
ENSMUSG00000049339	Retreg2	-0.326415838	0.007640862
ENSMUSG00000054843	Atrnl1	-0.326215255	0.000357761
ENSMUSG00000019866	Crybg1	-0.325867589	6.60E-26
ENSMUSG00000025076	Casp7	-0.325564885	2.44E-08
ENSMUSG00000025025	Mxi1	-0.325310823	8.52E-06

ENSMUSG00000027712	Anxa5	0.388417822	0.01856162
ENSMUSG00000031922	Cep57	0.388267856	0.024589241
ENSMUSG00000021756	Il6st	0.388064548	0.008551288
ENSMUSG00000051989	Smim11	0.387142508	0.007585796
ENSMUSG00000039542	Ncam1	0.384872989	0.037281104
ENSMUSG00000048970	C1galt1c1	0.384576972	0.003983908
ENSMUSG00000023805	Synj2	0.384484408	0.000299517
ENSMUSG00000092274	Neat1	0.383956781	0.000681713
ENSMUSG00000100801	Gm15459	0.383614676	0.034609858
ENSMUSG00000038369	Ncoa6	0.383071378	0.000898932
ENSMUSG00000030579	Tyrobp	0.382703618	0.00039658
ENSMUSG00000109324	Prmt1	0.382630921	0.007309413
ENSMUSG00000035245	Eogt	0.381469294	0.00066737
ENSMUSG00000027035	Cers6	0.381269591	0.000509931
ENSMUSG00000028053	Ash1l	0.381129035	6.96E-06
ENSMUSG00000035958	Tdp2	0.381116907	5.97E-13
ENSMUSG00000032572	Col6a4	0.380778416	3.94E-06
ENSMUSG00000022636	Alcam	0.380185429	0.001539384
ENSMUSG00000024691	Fam111a	0.380059301	4.56E-07
ENSMUSG00000019861	Gopc	0.379612573	0.001761033
ENSMUSG00000034160	Ogt	0.379404918	0.000336715
ENSMUSG00000037536	Fbxo34	0.379366305	0.004647615
ENSMUSG00000039795	Zfand1	0.3787636	0.00503928
ENSMUSG00000039294	Cybc1	0.378567703	0.001986562
ENSMUSG00000026608	Kctd3	0.378511237	0.022802322
ENSMUSG00000030691	Fchsd2	0.37838334	3.53E-12
ENSMUSG00000059866	Tnip2	0.378182884	0.025082927
ENSMUSG00000003123	Lipe	0.378175093	0.001355807
ENSMUSG00000070661	Rnf186	0.378112369	2.81E-27
ENSMUSG00000038323	1700066M21	0.37592	0.007924553
ENSMUSG00000016283	H2-M2	0.375869612	0.0044366
ENSMUSG00000036528	Ppfibp2	0.375576619	0.039422707
ENSMUSG00000063382	Bcl9l	0.375541433	0.000414182
ENSMUSG00000014633	Cmc2	0.375281123	0.030539428
ENSMUSG00000042292	Mrtfa	0.375198132	0.02715623
ENSMUSG00000027961	Lrrc39	0.374958646	1.53E-10
ENSMUSG00000020659	Cbll1	0.37489096	0.000550758
ENSMUSG00000027620	Rbm39	0.374822442	0.000877095
ENSMUSG00000079508	Apoo	0.374087837	0.00020292
ENSMUSG00000023243	Kcnk5	0.374016438	0.000424806
ENSMUSG00000018381	Abi3	0.373682008	5.68E-09
ENSMUSG00000022765	Snap29	0.373581558	0.002476358
ENSMUSG00000019857	Asf1a	0.373338623	0.043950555
ENSMUSG00000002228	Ppm1j	0.373000954	1.31E-41
ENSMUSG00000030747	Dgat2	0.372322206	9.58E-07
ENSMUSG00000047888	Tnrc6b	0.372317948	0.003032508
ENSMUSG00000036698	Ago2	0.372088643	7.62E-09
ENSMUSG00000020580	Rock2	0.372087091	0.00179514
ENSMUSG00000021549	Rasa1	0.372056616	0.026991512
ENSMUSG00000078139	AK157302	0.372032653	0.030553863
ENSMUSG00000028760	Eif4g3	0.371993646	3.02E-07
ENSMUSG00000001157	Gmcl1	0.371914672	0.011181699
ENSMUSG00000038459	Abhd17c	0.370346125	0.011213722
ENSMUSG00000020839	Tmigd1	0.369799268	3.38E-68
ENSMUSG00000035161	Ints6	0.369727243	0.015084918
ENSMUSG00000032352	Lrrc1	0.369638044	6.85E-06
ENSMUSG00000020538	Srebf1	0.369347075	5.33E-05
ENSMUSG00000030852	Tacc2	0.369242142	2.63E-06
ENSMUSG00000029439	Sfswap	0.369108025	0.000112419
ENSMUSG00000020009	Ifngr1	0.368818069	0.024872783
ENSMUSG00000031245	Hmgn5	0.368675907	8.17E-10
ENSMUSG00000027487	Cdk5rap1	0.368408075	0.012287356
ENSMUSG00000000355	Mcts1	0.367568759	4.16E-05
ENSMUSG00000112129	Pbld1	0.367308824	1.38E-37
ENSMUSG00000039195	Bbln	0.367199	0.001897836
ENSMUSG00000041779	Tram2	0.366698085	0.000584974
ENSMUSG00000026932	Nacc2	0.366693013	0.001046018
ENSMUSG00000042251	Pm20d1	0.365798988	0.009160416
ENSMUSG00000030207	Fam234b	0.365591127	0.004866308
ENSMUSG00000038497	Tmco3	0.365261856	1.33E-06
ENSMUSG00000029221	Slc30a9	0.364637593	0.000841206
ENSMUSG00000078676	Casc3	0.364067147	0.003556087
ENSMUSG00000032883	Acsl3	0.364066749	4.29E-07
ENSMUSG00000070939	Tgfbrap1	0.36406637	0.000617831
ENSMUSG00000058163	Gm5431	0.363695318	0.000455939
ENSMUSG00000001983	Taco1	0.363655679	2.58E-06
ENSMUSG00000034832	Tet3	0.363127991	5.30E-09
ENSMUSG00000022994	Adcy6	0.362859998	1.10E-06
ENSMUSG00000000282	Mnt	0.362355711	0.011983913
ENSMUSG00000027428	Rbbp9	0.361445515	0.031357622
ENSMUSG00000020766	Galk1	0.361017293	4.39E-16
ENSMUSG00000044709	Gemin7	0.361000579	0.004459285
ENSMUSG00000034610	Tut4	0.360879154	2.61E-05
ENSMUSG00000022568	Scrib	0.360674844	0.001731794
ENSMUSG00000040209	Zfp704	0.360528542	1.79E-08
ENSMUSG00000042613	Pbxip1	0.358176475	0.010630951
ENSMUSG00000028433	Ubap2	0.358008272	3.01E-10
ENSMUSG00000037992	Rara	0.357843198	0.030229682
ENSMUSG00000024501	Dpysl3	0.35755667	5.40E-06
ENSMUSG00000022565	Plec	0.357366686	2.45E-12
ENSMUSG00000032532	Cck	0.356768593	3.70E-150
ENSMUSG00000022098	Bmp1	0.356548447	1.72E-07
ENSMUSG00000074466	Gm15417	0.356290693	0.000132731
ENSMUSG00000037095	Lrg1	0.355927445	0.009187279
ENSMUSG00000042369	Rbm45	0.355752454	0.000636341
ENSMUSG00000061436	Hipk2	0.355644359	0.000256372
ENSMUSG00000060261	Gtf2i	0.355135214	8.06E-05
ENSMUSG00000059824	Dbp	0.353740547	0.018943183
ENSMUSG00000074802	Gas2l3	0.353086172	0.049244394
ENSMUSG00000059495	Arhgef12	0.352625244	0.02018339
ENSMUSG00000015943	Bola1	0.352546682	0.002844741
ENSMUSG00000030647	Ndufc2	0.352525166	4.77E-12
ENSMUSG00000026504	Sdccag8	0.352476791	1.38E-06
ENSMUSG00000061992	Gm4953	0.352250436	5.92E-15
ENSMUSG00000041763	Tpp2	0.352138072	3.70E-05
ENSMUSG00000027875	Hmgcs2	0.352017126	3.97E-42
ENSMUSG00000041130	Zfp598	0.351818374	9.32E-05
ENSMUSG00000072620	Slfn2	0.351583055	3.53E-19
ENSMUSG00000048550	Thnsl1	0.351509762	0.010310755
ENSMUSG00000038607	Gng10	0.351299045	0.003693273
ENSMUSG00000062098	Btbd3	0.35117891	0.018768285
ENSMUSG00000028470	Hint2	0.351062166	6.37E-11
ENSMUSG00000035637	Grhpr	0.351055925	1.93E-14
ENSMUSG00000021807	Rtraf	0.351023368	0.016632808
ENSMUSG00000066894	Vsig10	0.350435736	1.09E-19
ENSMUSG00000031546	Gins4	0.350041716	0.02525604
ENSMUSG00000096950	Gm9530	0.349608436	0.020813317
ENSMUSG00000008859	Rala	0.34960605	0.000202213
ENSMUSG00000043998	Mgat2	0.349353563	1.11E-09
ENSMUSG00000029599	Ddx54	0.348763176	1.00E-06
ENSMUSG00000066036	Ubr4	0.348583059	1.26E-05
ENSMUSG00000029102	Hgfac	0.348499036	3.91E-09

ENSMUSG00000025144	Cenpx	-0.345893955	6.34E-05
ENSMUSG00000022360	Atad2	-0.345388925	0.000185654
ENSMUSG00000081684	Rps2-ps13	-0.34530682	0.045962863
ENSMUSG00000060373	Hnrnpc	-0.344888959	0.000952451
ENSMUSG00000084957	Bbip1	-0.344510942	0.000109372
ENSMUSG00000005779	Psmb4	-0.344410703	0.000123042
ENSMUSG00000096950	Gm9530	-0.343967722	0.020813317
ENSMUSG00000027456	Sdcbp2	-0.343592405	1.54E-09
ENSMUSG00000040128	Pnrc1	-0.343567481	6.22E-13
ENSMUSG00000055239	Kcmf1	-0.343452185	0.000156065
ENSMUSG00000024730	Ms4a8a	-0.34318369	2.18E-05
ENSMUSG00000070284	Gmppb	-0.343117853	1.49E-09
ENSMUSG00000027384	Ndufaf5	-0.343023932	3.50E-06
ENSMUSG00000032171	Pin1	-0.342750719	0.005636551
ENSMUSG00000020917	Acly	-0.342473402	3.92E-05
ENSMUSG00000033589	Reep4	-0.342260311	2.62E-10
ENSMUSG00000024253	Dync2li1	-0.341904214	0.015760246
ENSMUSG00000093930	Hmgcs1	-0.34184031	7.27E-14
ENSMUSG00000028745	Capzb	-0.341637468	7.55E-10
ENSMUSG00000036372	Tmem258	-0.340479002	0.015298986
ENSMUSG00000030706	Mrpl48	-0.34010071	1.96E-07
ENSMUSG00000022559	Fbxl6	-0.340092761	0.008222951
ENSMUSG00000120252	Gm31504	-0.338953233	0.000744549
ENSMUSG00000046598	Bdh1	-0.33819861	2.81E-16
ENSMUSG00000020459	Mtif2	-0.337713287	2.64E-06
ENSMUSG00000030532	Hddc3	-0.337706525	5.18E-15
ENSMUSG00000028678	Kif2c	-0.337617143	0.010798597
ENSMUSG00000071604	Entrep1	-0.33761443	0.014991104
ENSMUSG00000072115	Ang	-0.337421743	3.60E-06
ENSMUSG00000032459	Mrps22	-0.337053102	0.005762682
ENSMUSG00000031357	Syap1	-0.336747603	0.000325163
ENSMUSG00000022797	Tfrc	-0.336577073	0.002726922
ENSMUSG00000038312	Edem2	-0.33630552	0.004998049
ENSMUSG00000023031	Cela1	-0.335999656	3.10E-07
ENSMUSG00000027366	Sppl2a	-0.335688852	9.05E-07
ENSMUSG00000026879	Gsn	-0.335370748	1.35E-83
ENSMUSG00000015533	Itga2	-0.334877728	2.91E-10
ENSMUSG00000004945	Tmem242	-0.334536094	1.66E-07
ENSMUSG00000016252	Atp5e	-0.33376147	4.21E-13
ENSMUSG00000028889	Yrdc	-0.333111037	0.044654497
ENSMUSG00000031309	Rps6ka3	-0.332845157	8.17E-05
ENSMUSG00000022545	Ercc4	-0.332620716	0.002972817
ENSMUSG00000035242	Oaz1	-0.33243443	1.98E-11
ENSMUSG00000020183	Cpm	-0.332193633	0.000506655
ENSMUSG00000045136	Tubb2b	-0.331930538	9.89E-13
ENSMUSG00000022014	Epsti1	-0.331897722	4.91E-06
ENSMUSG00000023004	Tuba1b	-0.331430443	0.001240041
ENSMUSG00000032802	Srxn1	-0.331411362	2.38E-09
ENSMUSG00000027513	Pck1	-0.331394289	1.48E-08
ENSMUSG00000024208	Uqcc2	-0.331214659	0.001008049
ENSMUSG00000020386	Sar1b	-0.330985971	2.47E-16
ENSMUSG00000032615	Nt5m	-0.330866792	0.001662336
ENSMUSG00000041120	Nbl1	-0.330614029	2.03E-79
ENSMUSG00000024513	Mbd2	-0.330206407	0.046498405
ENSMUSG00000055943	Emc7	-0.330121849	0.01138942
ENSMUSG00000028841	Cnksr1	-0.329456836	0.020355098
ENSMUSG00000014243	Zswim7	-0.329315639	8.84E-07
ENSMUSG00000001833	Septin7	-0.329314129	0.000649255
ENSMUSG00000019139	Isyna1	-0.329146358	0.001226979
ENSMUSG00000016308	Ube2a	-0.329018775	0.012960757
ENSMUSG00000028896	Rcc1	-0.328873574	1.02E-06
ENSMUSG00000025190	Got1	-0.32820993	1.79E-13
ENSMUSG00000002325	Irf9	-0.327094716	8.62E-10
ENSMUSG00000000088	Cox5a	-0.326639438	2.56E-26
ENSMUSG00000043629	1700019D03	-0.326614393	2.77E-18
ENSMUSG00000003161	Sri	-0.326474693	1.58E-09
ENSMUSG00000032456	Nmnat3	-0.325830702	4.39E-16
ENSMUSG00000032301	Psma4	-0.325620558	0.000194134
ENSMUSG00000028274	Rngtt	-0.32560423	0.003045526
ENSMUSG00000001082	Mfsd10	-0.325476337	1.77E-05
ENSMUSG00000019066	Rab3d	-0.325233969	3.02E-16
ENSMUSG00000016257	Prelid3b	-0.324888947	2.87E-08
ENSMUSG00000026638	Irf6	-0.32358388	0.002084516
ENSMUSG00000029992	Gfpt1	-0.323349502	1.28E-32
ENSMUSG00000058407	Txndc9	-0.323243505	0.002717478
ENSMUSG00000013483	Card14	-0.323186834	0.006547487
ENSMUSG00000027810	Eif2a	-0.322814152	0.000599811
ENSMUSG00000031919	Tmed6	-0.322794985	0.001023102
ENSMUSG00000020778	Ten1	-0.322395045	0.008325527
ENSMUSG00000042505	Sdhaf3	-0.32199338	7.05E-08
ENSMUSG00000038607	Gng10	-0.321830889	0.003693273
ENSMUSG00000033792	Atp7a	-0.321167137	0.000127399
ENSMUSG00000004460	Dnajb11	-0.320967123	0.017673494
ENSMUSG00000032519	Slc25a38	-0.320637914	0.001928136
ENSMUSG00000004849	Ap1s1	-0.320235361	0.000117374
ENSMUSG00000030770	Parva	-0.320196047	0.00025828
ENSMUSG00000074444	Defa30	-0.319287946	1.70E-05
ENSMUSG00000017057	Il13ra1	-0.31920785	9.51E-06
ENSMUSG00000038217	Tlcd2	-0.319074237	1.54E-11
ENSMUSG00000031353	Rbbp7	-0.319038275	0.001707272
ENSMUSG00000011958	Bnip2	-0.318787241	0.021077511
ENSMUSG00000035704	Alg8	-0.318458363	0.007777756
ENSMUSG00000031609	Sap30	-0.317980206	0.027859551
ENSMUSG00000039285	Azi2	-0.317894047	0.002357239
ENSMUSG00000019173	Rab5c	-0.317893207	3.24E-08
ENSMUSG00000021216	Tubal3	-0.317090613	8.18E-05
ENSMUSG00000025075	Habp2	-0.316775308	6.03E-07
ENSMUSG00000030298	Sec13	-0.316209054	3.63E-14
ENSMUSG00000041301	Cftr	-0.316149569	2.76E-11
ENSMUSG00000044533	Rps2	-0.316110776	0.013315496
ENSMUSG00000033021	Gmppa	-0.316071633	0.021765742
ENSMUSG00000022571	Pycrl	-0.316030643	8.09E-11
ENSMUSG00000040174	Alkbh3	-0.315751871	2.77E-05
ENSMUSG00000042460	C1galt1	-0.315748569	0.00116571
ENSMUSG00000056394	Lig1	-0.315564547	1.69E-08
ENSMUSG00000037685	Atp8a1	-0.315332501	0.003905009
ENSMUSG00000030647	Ndufc2	-0.315250178	4.77E-12
ENSMUSG00000022706	Mrpl40	-0.31510991	0.046836484
ENSMUSG00000034906	Ncaph	-0.314369213	0.012436677
ENSMUSG00000079685	Ulbp1	-0.314074024	0.006403808
ENSMUSG00000009630	Ppp2cb	-0.314005944	0.012888666
ENSMUSG00000021591	Glrx	-0.313287633	0.025919558
ENSMUSG00000029221	Slc30a9	-0.313153639	0.000841206
ENSMUSG00000013822	Elof1	-0.312909344	1.65E-05
ENSMUSG00000037070	Rbmxl1	-0.312444731	0.011726217
ENSMUSG00000028969	Cdk5	-0.312167919	0.000109372
ENSMUSG00000024608	Rps14	-0.311968732	0.001949464
ENSMUSG00000028409	Smu1	-0.311856559	0.001324359
ENSMUSG00000040455	Usp45	-0.311693734	0.048517154
ENSMUSG00000021981	Cab39l	-0.3111107	0.000675742
ENSMUSG00000020956	Dtd2	-0.310878391	0.01539186
ENSMUSG00000040631	Dok4	-0.310863944	2.17E-13

ENSMUSG00000036698	Ago2	0.429261959	7.62E-09
ENSMUSG00000035247	Hectd1	0.429102266	7.87E-16
ENSMUSG00000118376	Gm9926	0.428722708	0.00287052
ENSMUSG00000030126	Tmcc1	0.428633725	0.039558338
ENSMUSG00000039765	Cc2d2a	0.428518562	8.39E-07
ENSMUSG00000049800	Sertad2	0.428411335	0.005393929
ENSMUSG00000026074	Map4k4	0.427722725	6.07E-07
ENSMUSG00000024169	Ift140	0.426874326	0.026863897
ENSMUSG00000058325	Dock1	0.426807211	1.28E-10
ENSMUSG00000041164	Zmiz2	0.426543633	1.01E-05
ENSMUSG00000114358	Gm5200	0.426541381	6.33E-07
ENSMUSG00000022235	Cmbl	0.426194637	1.78E-05
ENSMUSG00000040481	Bptf	0.426060321	1.82E-14
ENSMUSG00000027463	Slc52a3	0.425613482	9.68E-133
ENSMUSG00000041895	Wipi1	0.42514456	0.007467407
ENSMUSG00000050711	Scg2	0.42501821	0.005961378
ENSMUSG00000045005	Fzd5	0.424580845	8.16E-18
ENSMUSG00000055013	Agap1	0.42374521	7.16E-09
ENSMUSG00000020198	Ap3d1	0.423433003	2.11E-07
ENSMUSG00000034218	Atm	0.423175538	0.000478021
ENSMUSG00000009681	Bcr	0.422955002	0.00167826
ENSMUSG00000003154	Foxj2	0.422597415	0.009317429
ENSMUSG00000045664	Cdc42ep2	0.422370075	0.020461029
ENSMUSG00000029173	Sepsecs	0.422287494	0.001539384
ENSMUSG00000024298	Zfp871	0.422239043	0.005817429
ENSMUSG00000047371	Zfp768	0.422043972	0.002892207
ENSMUSG00000047888	Tnrc6b	0.421823691	0.003032508
ENSMUSG00000041702	Btbd7	0.421614571	0.00030282
ENSMUSG00000021375	Kif13a	0.421475151	8.53E-14
ENSMUSG00000038658	Ric1	0.420387312	0.030584039
ENSMUSG00000034994	Eef2	0.419630146	1.56E-19
ENSMUSG00000024589	Nedd4l	0.419477556	1.58E-10
ENSMUSG00000045095	Magi1	0.418611669	0.000499552
ENSMUSG00000064442	Gm26225	0.418585269	0.029363438
ENSMUSG00000044465	Fhip1b	0.418523282	0.000149937
ENSMUSG00000032114	Slc37a4	0.417769854	2.10E-85
ENSMUSG00000057134	Ado	0.417462302	2.38E-05
ENSMUSG00000022565	Plec	0.417400485	2.45E-12
ENSMUSG00000045038	Prkce	0.417313922	1.12E-09
ENSMUSG00000021392	Nol8	0.417313548	0.044533113
ENSMUSG00000028465	Tln1	0.417128646	0.019964999
ENSMUSG00000021974	Fgf9	0.416702504	0.000578364
ENSMUSG00000042390	Gatad2b	0.415885173	1.79E-05
ENSMUSG00000041831	Sytl3	0.415557248	0.037730465
ENSMUSG00000050103	Agmo	0.415235484	8.23E-09
ENSMUSG00000054321	Taf4b	0.41452159	0.001361863
ENSMUSG00000037499	Nenf	0.414462884	9.02E-17
ENSMUSG00000028992	Nmnat1	0.414277313	8.29E-16
ENSMUSG00000020638	Cmpk2	0.413662774	1.79E-17
ENSMUSG00000025856	Pdgfa	0.413556352	0.018492819
ENSMUSG00000039477	Tnrc18	0.413313749	1.74E-07
ENSMUSG00000018412	Kansl1	0.412893414	0.011096963
ENSMUSG00000025609	Mkln1	0.412728831	4.78E-06
ENSMUSG00000020137	Thap2	0.412224216	0.010812507
ENSMUSG00000023224	Serping1	0.412084429	0.025814206
ENSMUSG00000019854	Reps1	0.411777469	1.21E-05
ENSMUSG00000036155	Mgat5	0.41173944	0.001437345
ENSMUSG00000034889	Cactin	0.411557557	0.000970805
ENSMUSG00000036281	Snapc4	0.411556521	0.024274076
ENSMUSG00000066621	Tecpr1	0.411111355	0.000741664
ENSMUSG00000040007	Bahd1	0.410558673	9.63E-06
ENSMUSG00000018401	Mtmr4	0.410422819	0.04399908
ENSMUSG00000015839	Nfe2l2	0.410365302	5.81E-79
ENSMUSG00000034659	Tmem109	0.410348971	0.001937543
ENSMUSG00000050592	Fam78a	0.410132138	0.018881599
ENSMUSG00000040703	Cyp2s1	0.409645299	3.23E-05
ENSMUSG00000031149	Praf2	0.409571606	4.57E-05
ENSMUSG00000026547	Tagln2	0.408818684	0.001404727
ENSMUSG00000031902	Nfatc3	0.407542255	2.73E-05
ENSMUSG00000039414	Heatr5b	0.406974074	0.01070431
ENSMUSG00000034269	Setd5	0.406858441	0.000421334
ENSMUSG00000043154	Ppp2r3a	0.406081238	4.62E-11
ENSMUSG00000036918	Ttc7	0.405483915	0.009317429
ENSMUSG00000025225	Nfkb2	0.404793728	1.50E-06
ENSMUSG00000029684	Wasl	0.404633351	2.74E-32
ENSMUSG00000039529	Atp8b1	0.403412295	6.53E-23
ENSMUSG00000021065	Fut8	0.403344563	3.85E-06
ENSMUSG00000034908	Sidt2	0.402995082	0.003377762
ENSMUSG00000037791	Phf12	0.402428789	0.000278762
ENSMUSG00000044365	Cxxc4	0.402427667	3.04E-06
ENSMUSG00000018707	Dync1h1	0.402288164	8.90E-05
ENSMUSG00000034371	Tkfc	0.402137748	1.29E-93
ENSMUSG00000052534	Pbx1	0.40211681	9.62E-20
ENSMUSG00000066894	Vsig10	0.401952135	1.09E-19
ENSMUSG00000040498	Igsf23	0.401937831	1.20E-11
ENSMUSG00000050271	Prag1	0.401726377	0.006974247
ENSMUSG00000025220	Oga	0.401523096	0.001836723
ENSMUSG00000045248	Med26	0.401469618	0.001395375
ENSMUSG00000052539	Magi3	0.401304757	2.29E-06
ENSMUSG00000038020	Rapgefl1	0.400861483	0.040524515
ENSMUSG00000005628	Tmod4	0.400388076	0.002974021
ENSMUSG00000025792	Slc25a10	0.400211246	0.007445477
ENSMUSG00000015806	Qdpr	0.400170988	2.46E-06
ENSMUSG00000026827	Gpd2	0.400161941	1.82E-15
ENSMUSG00000037600	Kdf1	0.39975303	0.010643215
ENSMUSG00000003948	Mmd	0.399720251	0.0001174
ENSMUSG00000099583	H3c4	0.399623449	0.027308807
ENSMUSG00000010277	Bltp2	0.399612551	0.032442581
ENSMUSG00000003346	Abhd17a	0.399214135	5.08E-06
ENSMUSG00000045410	Akr1e1	0.399170505	6.87E-18
ENSMUSG00000008348	Ubc	0.398972918	0.007368425
ENSMUSG00000025486	Sirt3	0.398760471	5.08E-07
ENSMUSG00000045665	Mfsd5	0.398379773	0.023962331
ENSMUSG00000006638	Abhd1	0.397975485	4.24E-24
ENSMUSG00000060716	Plekhh1	0.397558769	0.011223877
ENSMUSG00000031540	Kat6a	0.396907329	0.000657803
ENSMUSG00000013707	Tnfaip8l2	0.396647835	6.78E-06
ENSMUSG00000033610	Pank1	0.39650947	0.008244926
ENSMUSG00000037270	Bltp1	0.394898247	0.004946374
ENSMUSG00000028519	Dab1	0.394850735	0.041862076
ENSMUSG00000029630	Cyp3a25	0.39420599	4.92E-59
ENSMUSG00000049339	Retreg2	0.39390809	0.007640862
ENSMUSG00000029291	Rufy3	0.393868342	0.031320364
ENSMUSG00000034485	Uaca	0.393827965	2.08E-11
ENSMUSG00000020821	Kif1c	0.393423185	3.89E-12
ENSMUSG00000027428	Rbbp9	0.393361477	0.031357622
ENSMUSG00000026798	Coq4	0.393111878	3.07E-08
ENSMUSG00000025436	Atp23	0.39159204	0.00545803
ENSMUSG00000025396	Hsd17b6	0.391486825	8.79E-56
ENSMUSG00000073481	Mtarc2	0.391426998	9.37E-163
ENSMUSG00000028003	Lrat	0.391406437	1.52E-29
ENSMUSG00000071064	Zfp827	0.391165477	9.83E-05

ENSMUSG00000070348	Ccnd1	-0.324710957	2.67E-63
ENSMUSG00000038503	Mesd	-0.323860658	0.023590132
ENSMUSG00000027552	E2f5	-0.323708199	0.014233943
ENSMUSG00000030744	Rps3	-0.323686601	0.029491611
ENSMUSG00000057069	Ero1b	-0.323679693	0.027736522
ENSMUSG00000034473	Sec22a	-0.323624635	2.44E-05
ENSMUSG00000021928	Ebpl	-0.323462141	0.048646406
ENSMUSG00000104445	Rhbg	-0.323332693	0.005445631
ENSMUSG00000027555	Car13	-0.323196403	0.002240767
ENSMUSG00000034248	Slc25a37	-0.323055907	4.34E-13
ENSMUSG00000067787	Blcap	-0.322700272	0.009711665
ENSMUSG00000032216	Nedd4	-0.32264922	5.76E-10
ENSMUSG00000031168	Ebp	-0.322503392	0.000131971
ENSMUSG00000079197	Psme2	-0.32238024	1.99E-07
ENSMUSG00000060961	Slc4a4	-0.321186076	1.75E-10
ENSMUSG00000026887	Mrrf	-0.321055014	0.003310363
ENSMUSG00000021998	Lcp1	-0.320282378	0.002960495
ENSMUSG00000039081	Zfp503	-0.320175974	1.08E-14
ENSMUSG00000029730	Mcm7	-0.319695303	0.002040617
ENSMUSG00000009569	Mrtfb	-0.319675539	5.87E-05
ENSMUSG00000040147	Maob	-0.319638877	1.42E-08
ENSMUSG00000020023	Tmcc3	-0.319608525	3.55E-13
ENSMUSG00000037787	Coa8	-0.319145161	0.00075467
ENSMUSG00000028678	Kif2c	-0.318447102	0.010798597
ENSMUSG00000037075	Rnf139	-0.317953039	2.00E-05
ENSMUSG00000037600	Kdf1	-0.317775932	0.010643215
ENSMUSG00000026939	Tmem141	-0.317137298	0.019720579
ENSMUSG00000019873	Reep3	-0.31686186	0.002486837
ENSMUSG00000026839	Upp2	-0.316271851	0.006939633
ENSMUSG00000005481	Ddx39a	-0.316040941	0.005868545
ENSMUSG00000061666	Gdpd1	-0.315762348	4.37E-89
ENSMUSG00000024644	Cndp2	-0.315560827	9.91E-14
ENSMUSG00000030769	Slc5a11	-0.31543237	1.88E-08
ENSMUSG00000095079	Igha	-0.315359585	7.83E-06
ENSMUSG00000030879	Mrpl17	-0.314909668	1.06E-09
ENSMUSG00000047881	Rell1	-0.314629931	1.37E-40
ENSMUSG00000026201	Stk16	-0.314228386	0.000649602
ENSMUSG00000033047	Eif3l	-0.314152748	0.022767552
ENSMUSG00000024180	Pgap6	-0.31412622	4.85E-11
ENSMUSG00000001665	Gstt3	-0.313655514	0.004129941
ENSMUSG00000057092	Fxyd3	-0.313164392	0.000161492
ENSMUSG00000028044	Cks1b	-0.313155101	0.000152616
ENSMUSG00000019969	Psen1	-0.312638716	0.002222725
ENSMUSG00000015094	Npdc1	-0.312577915	0.005348605
ENSMUSG00000029416	Slc15a4	-0.312442984	0.001469359
ENSMUSG00000033021	Gmppa	-0.312425562	0.021765742
ENSMUSG00000055200	Sertad3	-0.312353339	0.00747671
ENSMUSG00000030551	Nr2f2	-0.312195469	3.84E-21
ENSMUSG00000043668	Tox3	-0.311396466	6.86E-08
ENSMUSG00000018761	Mpdu1	-0.311171609	2.19E-11
ENSMUSG00000074129	Rpl13a	-0.311127143	0.001667726
ENSMUSG00000070730	Rmdn3	-0.31106772	1.00E-11
ENSMUSG00000059325	Hopx	-0.310821115	7.52E-12
ENSMUSG00000024487	Yipf5	-0.310751878	1.70E-14
ENSMUSG00000020897	Aurkb	-0.310588923	0.000655942
ENSMUSG00000085148	Mir22hg	-0.31048544	1.06E-08
ENSMUSG00000031311	Nono	-0.310350831	0.003877031
ENSMUSG00000020788	Atp2a3	-0.310152492	2.28E-09
ENSMUSG00000025102	3110040N11	-0.309785452	0.010951921
ENSMUSG00000040151	Hs2st1	-0.309413893	0.002850089
ENSMUSG00000024493	Lars	-0.309015799	0.001765034
ENSMUSG00000066900	Suds3	-0.30895783	2.94E-06
ENSMUSG00000029545	Acads	-0.308858826	0.004406103
ENSMUSG00000020467	Efemp1	-0.30849821	0.023672623
ENSMUSG00000052271	Bhlha15	-0.307581264	9.16E-06
ENSMUSG00000081534	Slc48a1	-0.307038484	0.001700455
ENSMUSG00000004040	Stat3	-0.305815369	0.000148257
ENSMUSG00000032280	Tle3	-0.305078724	3.91E-23
ENSMUSG00000030978	Rrm1	-0.30495028	0.000293034
ENSMUSG00000021226	Acot2	-0.304631353	2.55E-24
ENSMUSG00000000743	Chmp1a	-0.304405746	0.007806352
ENSMUSG00000078619	Smarcd2	-0.303439398	0.002697978
ENSMUSG00000031078	Cttn	-0.303260988	6.29E-32
ENSMUSG00000040297	Suco	-0.302657776	0.001290253
ENSMUSG00000005442	Cic	-0.302072069	8.02E-06
ENSMUSG00000037991	Rmi2	-0.300925108	0.018055568
ENSMUSG00000038766	Gabpb2	-0.300876707	6.91E-08
ENSMUSG00000021417	Eci2	-0.30063616	0.001073064
ENSMUSG00000007872	Id3	-0.300057077	3.92E-11
ENSMUSG00000053390	Zfp952	-0.299885154	1.59E-05
ENSMUSG00000053113	Socs3	-0.299219601	0.000194357
ENSMUSG00000026589	Sec16b	-0.299041966	1.25E-05
ENSMUSG00000045664	Cdc42ep2	-0.298656886	0.020461029
ENSMUSG00000036775	Decr2	-0.298568698	0.001576695
ENSMUSG00000043587	Pxylp1	-0.29809749	0.016054813
ENSMUSG00000015749	Anp32e	-0.297667576	1.82E-05
ENSMUSG00000034311	Kif4	-0.297499181	0.012708848
ENSMUSG00000039159	Ube2h	-0.297429108	3.59E-05
ENSMUSG00000020777	Acox1	-0.297047286	8.92E-06
ENSMUSG00000005362	Crbn	-0.296982583	0.015600001
ENSMUSG00000017831	Rab5a	-0.296662414	0.021538676
ENSMUSG00000090700	Cyp4f40	-0.296457992	4.23E-22
ENSMUSG00000102106	2310043O21	-0.29599627	0.011305183
ENSMUSG00000031819	Emc8	-0.295841059	0.004454409
ENSMUSG00000052997	Uba2	-0.295237899	0.034474675
ENSMUSG00000069520	Tmem19	-0.294639051	0.000242749
ENSMUSG00000032261	Sh3bgrl2	-0.294633681	8.66E-50
ENSMUSG00000031921	Terf2	-0.29362195	0.004874025
ENSMUSG00000069895	Atxn1l	-0.292842732	0.015630553
ENSMUSG00000057137	Tmem140	-0.292504762	2.14E-06
ENSMUSG00000022664	Slc35a5	-0.292413513	0.020832341
ENSMUSG00000022186	Oxct1	-0.292372429	3.51E-06
ENSMUSG00000040414	Slc25a28	-0.291957125	0.008520681
ENSMUSG00000061878	Sphk1	-0.291692765	7.83E-11
ENSMUSG00000039988	Ankrd13c	-0.291565683	0.01149584
ENSMUSG00000025817	Nudt5	-0.291487492	0.001599213
ENSMUSG00000030413	Pglyrp1	-0.291472948	5.48E-27
ENSMUSG00000066798	Zbtb6	-0.290730844	0.001250781
ENSMUSG00000045503	Sys1	-0.29034319	0.013314323
ENSMUSG00000037313	Tacc3	-0.290272004	0.001418447
ENSMUSG00000026553	Copa	-0.289630912	0.019989731
ENSMUSG00000002289	Angptl4	-0.289299005	1.93E-07
ENSMUSG00000005871	Apc	-0.288714117	2.05E-05
ENSMUSG00000026519	Tmem63a	-0.288523917	3.38E-05
ENSMUSG00000111709	Gm3776	-0.288407575	1.25E-36
ENSMUSG00000025980	Hspd1	-0.288214273	0.009410388
ENSMUSG00000062908	Acadm	-0.288005507	1.12E-06
ENSMUSG00000024672	Ms4a7	-0.287966529	0.000843846
ENSMUSG00000029649	Pomp	-0.287388442	0.003713724
ENSMUSG00000019579	Mydgf	-0.286637358	0.027264864
ENSMUSG00000052632	Asap2	-0.286473138	2.00E-11
ENSMUSG00000034308	Sdr42e1	-0.285817564	0.023198941

ENSMUSG00000031788	Kifc3	0.348373694	0.003092098
ENSMUSG00000026088	Mitd1	0.348135415	2.72E-09
ENSMUSG00000040249	Lrp1	0.348105542	4.02E-09
ENSMUSG00000017453	Pipox	0.347606599	8.58E-29
ENSMUSG00000031575	Ash2l	0.346912195	0.000116542
ENSMUSG00000030019	Fbxl14	0.346814529	0.007259845
ENSMUSG00000003863	Ppfia3	0.34671968	7.72E-09
ENSMUSG00000050914	Ankrd37	0.345837057	1.49E-05
ENSMUSG00000041238	Rbbp8	0.345823128	0.01990862
ENSMUSG00000029632	Ndufa4	0.345545249	1.35E-08
ENSMUSG00000021208	Ifi27l2b	0.344823331	2.86E-19
ENSMUSG00000022462	Slc38a2	0.344075561	5.82E-11
ENSMUSG00000032405	Pias1	0.344032419	0.002243948
ENSMUSG00000030532	Hddc3	0.34390424	5.18E-15
ENSMUSG00000025465	Echs1	0.343589062	1.59E-11
ENSMUSG00000030126	Tmcc1	0.343455283	0.039558338
ENSMUSG00000063317	Usp31	0.342843301	0.019597778
ENSMUSG00000035623	Rsf1	0.342818274	0.0083493
ENSMUSG00000028106	Rprd2	0.34233476	0.000229063
ENSMUSG00000066026	Dhrs3	0.341634484	3.68E-05
ENSMUSG00000039218	Srrm2	0.341388924	4.27E-09
ENSMUSG00000048251	Bcl11b	0.341114652	8.52E-17
ENSMUSG00000040274	Cdk6	0.340988643	1.57E-06
ENSMUSG00000015806	Qdpr	0.340974926	2.46E-06
ENSMUSG00000037210	Fam193a	0.340475849	7.37E-05
ENSMUSG00000085939	Cd63-ps	0.340294026	8.78E-13
ENSMUSG00000017760	Ctsa	0.340249333	0.032549775
ENSMUSG00000018239	Zcchc10	0.339812954	0.000991987
ENSMUSG00000028251	Tstd3	0.3398127	0.045460277
ENSMUSG00000018931	Natd1	0.339771082	0.00751999
ENSMUSG00000039967	Zfp292	0.338844125	0.038452778
ENSMUSG00000021127	Zfp36l1	0.338727666	0.000231027
ENSMUSG00000040423	Rc3h1	0.338067636	0.047125216
ENSMUSG00000041417	Pik3r1	0.338041467	0.000513393
ENSMUSG00000035401	Emsy	0.337662776	0.040590545
ENSMUSG00000047388	Atmin	0.337588749	8.58E-07
ENSMUSG00000042638	Gucy2c	0.33706754	0.000688803
ENSMUSG00000028706	Nsun4	0.33683292	0.000129291
ENSMUSG00000021156	Zmynd11	0.336598224	0.047844666
ENSMUSG00000024535	Snx24	0.336555803	0.039809059
ENSMUSG00000022443	Myh9	0.336294567	8.03E-08
ENSMUSG00000055917	Zfp277	0.336223411	0.000594613
ENSMUSG00000069729	Arid1b	0.336191911	0.00015871
ENSMUSG00000005836	Gata6	0.335979188	6.02E-07
ENSMUSG00000116673	A630089N07	0.335767414	0.012945209
ENSMUSG00000035770	Dync1li2	0.33511295	0.027674198
ENSMUSG00000040761	Spen	0.334965888	1.24E-07
ENSMUSG00000045316	Fahd1	0.334553788	1.74E-24
ENSMUSG00000038909	Kat7	0.333914365	0.021533336
ENSMUSG00000034201	Gas2l1	0.333839941	7.72E-06
ENSMUSG00000048126	Col6a3	0.333758901	0.005612682
ENSMUSG00000083380	Ndufb4c	0.333429344	2.10E-16
ENSMUSG00000029673	Auts2	0.333388409	5.01E-06
ENSMUSG00000040048	Ndufb10	0.333142651	7.74E-14
ENSMUSG00000036093	Arl5a	0.332747227	2.32E-20
ENSMUSG00000039671	Zmynd8	0.331378962	0.000408024
ENSMUSG00000039841	Zfp800	0.33116584	0.006223857
ENSMUSG00000037031	Tspan15	0.330833786	0.021907569
ENSMUSG00000020232	Hmg20b	0.330803142	1.97E-08
ENSMUSG00000027016	Zfp385b	0.330471788	6.44E-06
ENSMUSG00000040843	Tiprl	0.330420537	0.032391014
ENSMUSG00000015846	Rxra	0.330228719	3.26E-09
ENSMUSG00000000631	Myo18a	0.329718652	8.41E-06
ENSMUSG00000001855	Nup214	0.328927167	0.008133624
ENSMUSG00000063296	Tmem117	0.32846416	6.11E-82
ENSMUSG00000027341	Tmem230	0.328011702	5.47E-06
ENSMUSG00000036197	Gxylt1	0.327934146	0.000833849
ENSMUSG00000063870	Chd4	0.327882782	1.55E-05
ENSMUSG00000031778	Cx3cl1	0.327736117	2.18E-05
ENSMUSG00000068184	Ndufaf2	0.327697668	0.00489906
ENSMUSG00000004748	Mtfp1	0.326698494	1.05E-15
ENSMUSG00000027398	Il1b	0.326440412	0.021441111
ENSMUSG00000049303	Syt12	0.326145634	0.011283469
ENSMUSG00000030739	Myh14	0.326091942	6.86E-11
ENSMUSG00000021290	Atp5mpl	0.325959906	2.37E-06
ENSMUSG00000049076	Acap2	0.325920142	0.018915017
ENSMUSG00000063235	Ptpmt1	0.325468342	2.58E-08
ENSMUSG00000023965	Fbxl17	0.325045585	0.049868443
ENSMUSG00000001100	Poldip2	0.324951688	0.000940742
ENSMUSG00000022255	Mtdh	0.324615535	1.59E-07
ENSMUSG00000022755	Adgrg7	0.324605106	7.44E-06
ENSMUSG00000097571	Jpx	0.324315239	0.017353324
ENSMUSG00000055723	Rras2	0.324204141	0.013025101
ENSMUSG00000029618	Ocm	0.323538116	1.48E-68
ENSMUSG00000029202	Pds5a	0.323479956	4.16E-05
ENSMUSG00000028032	Papss1	0.32345536	2.18E-26
ENSMUSG00000021670	Hmgcr	0.323171916	4.69E-10
ENSMUSG00000029759	Pon3	0.322801612	1.23E-06
ENSMUSG00000056569	Mpz	0.322448195	0.00691414
ENSMUSG00000063229	Ldha	0.322439067	5.57E-29
ENSMUSG00000036352	Ubac1	0.32211452	9.12E-11
ENSMUSG00000022223	Sdr39u1	0.32186852	0.000242749
ENSMUSG00000018398	Septin8	0.321708021	3.24E-16
ENSMUSG00000028145	Them4	0.321439444	2.27E-11
ENSMUSG00000070031	Sp140	0.321266022	2.20E-06
ENSMUSG00000026112	Coa5	0.3203721	0.037298003
ENSMUSG00000021583	Erap1	0.319969266	0.02423143
ENSMUSG00000034243	Golgb1	0.319812595	9.99E-13
ENSMUSG00000028278	Rragd	0.318838002	6.73E-17
ENSMUSG00000015247	Nipsnap3b	0.318603266	0.000355004
ENSMUSG00000029815	Malsu1	0.318381139	0.000163342
ENSMUSG00000068706	Gm10250	0.316722115	1.26E-20
ENSMUSG00000026478	Lamc1	0.316413711	0.048902709
ENSMUSG00000090000	Ier3ip1	0.316371808	0.000326758
ENSMUSG00000024790	Sac3d1	0.315779324	4.27E-07
ENSMUSG00000002608	Ccdc97	0.315706686	0.0085051
ENSMUSG00000040339	Eeig2	0.31464926	6.34E-34
ENSMUSG00000024387	Csnk2b	0.314531277	0.001502127
ENSMUSG00000011257	Pabpc4	0.314383331	3.25E-10
ENSMUSG00000028655	Mfsd2a	0.314292158	1.83E-15
ENSMUSG00000022900	Ildr1	0.3136975	0.001999876
ENSMUSG00000041203	Trir	0.313561737	0.025844537
ENSMUSG00000022812	Gsk3b	0.313510047	9.83E-06
ENSMUSG00000036585	Fgf1	0.312991856	0.006932516
ENSMUSG00000031231	Cox7b	0.312969946	3.74E-11
ENSMUSG00000034681	Rnps1	0.312727301	0.0303846
ENSMUSG00000026796	Niban2	0.312201716	2.69E-07
ENSMUSG00000027519	Rab22a	0.312151933	0.021790683
ENSMUSG00000029705	Cux1	0.312137362	1.17E-13
ENSMUSG00000053581	Zfand2a	0.311634498	0.021872205
ENSMUSG00000021720	Rnf180	0.310835664	0.002219141
ENSMUSG00000024077	Strn	0.310763131	1.38E-06

ENSMUSG00000042507	Mideas	-0.310762682	0.011009046
ENSMUSG00000050323	Ndufaf6	-0.310537296	0.013421324
ENSMUSG00000024691	Fam111a	-0.310094593	4.56E-07
ENSMUSG00000031938	4931406C07	-0.308337862	1.37E-14
ENSMUSG00000020017	Hal	-0.308062984	5.11E-05
ENSMUSG00000040659	Efhd2	-0.307487694	5.00E-22
ENSMUSG00000056629	Fkbp2	-0.306365534	0.010618603
ENSMUSG00000096255	Dynlt1b	-0.305844063	0.000234715
ENSMUSG00000015759	Cnih1	-0.305762257	5.30E-06
ENSMUSG00000027552	E2f5	-0.305454936	0.014233943
ENSMUSG00000028413	B4galt1	-0.305447328	4.08E-16
ENSMUSG00000027551	Zfp64	-0.305444552	0.000940773
ENSMUSG00000039737	Prkrip1	-0.305405292	0.000281226
ENSMUSG00000015671	Psma2	-0.305002828	0.01229255
ENSMUSG00000031799	Tpm4	-0.304386964	0.006838564
ENSMUSG00000042029	Ncapg2	-0.304305065	0.03892037
ENSMUSG00000031231	Cox7b	-0.304145782	3.74E-11
ENSMUSG00000028719	Cmpk1	-0.304142608	7.37E-06
ENSMUSG00000005362	Crbn	-0.304016906	0.015600001
ENSMUSG00000005087	Cd44	-0.3039344	0.001807316
ENSMUSG00000021457	Syk	-0.303729992	0.000505261
ENSMUSG00000001666	Ddt	-0.303634494	5.88E-08
ENSMUSG00000057649	Brd9	-0.303522884	0.00010538
ENSMUSG00000070777	Ceacam20	-0.30328878	5.61E-11
ENSMUSG00000057858	Fam204a	-0.303236518	4.73E-06
ENSMUSG00000024340	Btnl2	-0.302858519	0.000365595
ENSMUSG00000048373	Fgfbp1	-0.302775142	7.61E-25
ENSMUSG00000071014	Ndufb6	-0.302578004	1.88E-09
ENSMUSG00000034854	Mfsd12	-0.30235202	0.024100058
ENSMUSG00000045838	Ccdc9b	-0.30200962	0.03692031
ENSMUSG00000030921	Trim30a	-0.301950908	9.79E-09
ENSMUSG00000038812	Trmt112	-0.301705752	0.000176188
ENSMUSG00000024105	Themis3	-0.301699693	7.62E-05
ENSMUSG00000003309	Ap1m2	-0.301608309	0.000509684
ENSMUSG00000035559	Mpv17l2	-0.30073965	0.000814668
ENSMUSG00000028037	Ifi44	-0.299982786	0.001720194
ENSMUSG00000039195	Bbln	-0.299968457	0.001897836
ENSMUSG00000029513	Prkab1	-0.299278376	9.09E-20
ENSMUSG00000032245	Cln6	-0.299227742	0.000303194
ENSMUSG00000026558	Uck2	-0.299172128	4.94E-06
ENSMUSG00000020390	Ube2b	-0.298912721	1.69E-08
ENSMUSG00000027357	Crls1	-0.298889676	0.044354991
ENSMUSG00000038518	Jarid2	-0.298838904	5.91E-10
ENSMUSG00000063524	Eno1	-0.29875563	3.75E-15
ENSMUSG00000038503	Mesd	-0.298511876	0.023590132
ENSMUSG00000014859	E2f4	-0.298407176	0.000728529
ENSMUSG00000024556	Me2	-0.297687052	2.55E-11
ENSMUSG00000006050	Sra1	-0.297370615	1.26E-10
ENSMUSG00000046865	Fbl	-0.297105488	0.025541177
ENSMUSG00000017713	Tha1	-0.296554217	0.000598829
ENSMUSG00000042682	Selenok	-0.296523896	0.020343029
ENSMUSG00000032126	Hmbs	-0.296479942	0.03149149
ENSMUSG00000025759	Mfsd8	-0.296372704	0.02769699
ENSMUSG00000056116	H2-T22	-0.29627461	0.009414367
ENSMUSG00000075702	Selenom	-0.296146437	0.017645423
ENSMUSG00000066800	Rnasel	-0.295938956	4.14E-06
ENSMUSG00000022841	Ap2m1	-0.295336609	0.000975717
ENSMUSG00000048200	Cracr2b	-0.295284083	0.007127017
ENSMUSG00000059325	Hopx	-0.295212738	7.52E-12
ENSMUSG00000066362	Rps13-ps1	-0.294622675	1.86E-05
ENSMUSG00000058729	Lin9	-0.294546405	8.51E-05
ENSMUSG00000030612	Mrpl46	-0.29441911	8.02E-10
ENSMUSG00000041355	Ssr2	-0.294188153	0.001026968
ENSMUSG00000005981	Trap1	-0.293839273	0.027247136
ENSMUSG00000036894	Rap2b	-0.293647924	0.002590358
ENSMUSG00000003863	Ppfia3	-0.293541797	7.72E-09
ENSMUSG00000039208	Metrnl	-0.29329123	0.00060125
ENSMUSG00000071528	Atp5md	-0.293260643	1.57E-10
ENSMUSG00000006998	Psmd2	-0.292677109	0.000747486
ENSMUSG00000021917	Spcs1	-0.292479355	0.023607887
ENSMUSG00000063787	Chchd1	-0.292458724	2.93E-05
ENSMUSG00000030019	Fbxl14	-0.292264369	0.007259845
ENSMUSG00000028247	Coq3	-0.292030749	0.002269371
ENSMUSG00000025027	Xpnpep1	-0.291549865	1.43E-25
ENSMUSG00000030061	Uba3	-0.290921061	3.45E-05
ENSMUSG00000054598	9130230L23	-0.290103323	1.08E-09
ENSMUSG00000030282	Cmas	-0.29006023	0.004257107
ENSMUSG00000008475	Arpc5	-0.28989225	0.010850966
ENSMUSG00000031819	Emc8	-0.289875718	0.004454409
ENSMUSG00000031604	Msmo1	-0.289538677	0.003216875
ENSMUSG00000029066	Mrpl20	-0.289033343	2.01E-08
ENSMUSG00000090136	Gm10177	-0.288822416	1.06E-06
ENSMUSG00000031776	Arl2bp	-0.288535912	0.049113022
ENSMUSG00000029462	Vps29	-0.288521358	9.06E-05
ENSMUSG00000019973	Cops3	-0.287900197	3.94E-05
ENSMUSG00000032468	Armc8	-0.287536102	0.003552609
ENSMUSG00000053411	Cbx7	-0.287436263	0.001925498
ENSMUSG00000017221	Psmd3	-0.287398971	0.001035531
ENSMUSG00000026187	Xrcc5	-0.287092339	0.001978751
ENSMUSG00000025616	Usp16	-0.287032957	1.81E-06
ENSMUSG00000053510	Nrd1	-0.286905735	0.001972629
ENSMUSG00000058267	Mrps14	-0.286575203	0.016040978
ENSMUSG00000042093	Ndufv2	-0.285942393	9.66E-19
ENSMUSG00000061718	Ppp1r1b	-0.285601429	0.001765034
ENSMUSG00000023048	Prr13	-0.285384429	4.15E-10
ENSMUSG00000054662	Ano9	-0.284692071	0.030150408
ENSMUSG00000110444	Gm10033	-0.284684504	0.047844666
ENSMUSG00000028737	Aldh4a1	-0.284632348	9.02E-08
ENSMUSG00000055320	Tead1	-0.28457715	5.65E-10
ENSMUSG00000020108	Ddit4	-0.284277897	0.014911637
ENSMUSG00000021962	Dcp1a	-0.28410759	1.49E-05
ENSMUSG00000038225	Primpol	-0.283922885	0.009959751
ENSMUSG00000059743	Fdps	-0.283683997	6.35E-08
ENSMUSG00000042444	Mindy2	-0.283265504	0.000324791
ENSMUSG00000051223	Bzw1	-0.283184019	0.043079913
ENSMUSG00000025429	Pstpip2	-0.283123706	3.56E-06
ENSMUSG00000058569	Tmed9	-0.283004326	1.83E-14
ENSMUSG00000091957	Rps2-ps10	-0.282683277	0.033964931
ENSMUSG00000070713	Hmgn2-ps	-0.282254165	0.037299163
ENSMUSG00000024576	Csnk1a1	-0.2822415	5.48E-07
ENSMUSG00000025204	Ndufb8	-0.282014124	1.08E-21
ENSMUSG00000002329	Mdp1	-0.281951678	7.05E-12
ENSMUSG00000026600	Soat1	-0.281897481	0.009144356
ENSMUSG00000018217	Pmp22	-0.281702985	1.50E-21
ENSMUSG00000031173	Otc	-0.281471868	6.14E-06
ENSMUSG00000025630	Hprt	-0.28084104	0.000491529
ENSMUSG00000058013	Septin11	-0.280034265	7.75E-06
ENSMUSG00000028654	Mycl	-0.280021584	0.003419422
ENSMUSG00000021684	Pde8b	-0.279513888	2.10E-06
ENSMUSG00000027940	Tpm3	-0.279470185	0.005704977
ENSMUSG00000035960	Apex1	-0.279214196	0.015689327
ENSMUSG00000020328	Nudcd2	-0.279176182	0.011324528

ENSMUSG00000033909	Usp36	0.390697019	0.001926304
ENSMUSG00000025278	Flnb	0.390676553	2.21E-09
ENSMUSG00000087119	Atg4a-ps	0.390043346	0.019520074
ENSMUSG00000022574	Naprt	0.38998159	1.82E-07
ENSMUSG00000062866	Phactr2	0.389783909	8.34E-07
ENSMUSG00000021028	Mbip	0.38941862	0.01152163
ENSMUSG00000021782	Dlg5	0.389339156	1.21E-06
ENSMUSG00000032902	Slc16a1	0.38906607	1.46E-06
ENSMUSG00000028124	Gclm	0.388523826	6.95E-12
ENSMUSG00000027510	Rbm38	0.388008566	0.015007255
ENSMUSG00000022856	Tmem41a	0.3878719	0.041177463
ENSMUSG00000003660	Snrnp200	0.387559646	0.001116991
ENSMUSG00000031767	Nudt7	0.387527177	1.43E-21
ENSMUSG00000028405	Aco1	0.38735982	0.001654081
ENSMUSG00000017679	Ttpal	0.387090678	0.000654262
ENSMUSG00000037390	Muc3	0.387086366	3.39E-05
ENSMUSG00000042207	Kdm5b	0.386882235	0.02409627
ENSMUSG00000029426	Scarb2	0.386753138	0.0234826
ENSMUSG00000038351	Sgsm2	0.386649057	0.038018638
ENSMUSG00000000881	Dlg3	0.386396065	0.042395818
ENSMUSG00000032121	Tmem218	0.385866711	0.025030331
ENSMUSG00000037210	Fam193a	0.384889504	7.37E-05
ENSMUSG00000019864	Rtn4ip1	0.384804215	0.014092674
ENSMUSG00000018900	Slc22a5	0.384010281	6.34E-17
ENSMUSG00000024866	Acy3	0.383905186	4.33E-10
ENSMUSG00000027665	Pik3ca	0.383751021	0.02841654
ENSMUSG00000033373	Fntb	0.383499618	2.47E-07
ENSMUSG00000048920	Fkrp	0.383286336	0.002321765
ENSMUSG00000038174	Hycc2	0.383153728	5.51E-07
ENSMUSG00000039967	Zfp292	0.382089635	0.038452778
ENSMUSG00000015341	Golga7	0.382089302	0.045992602
ENSMUSG00000027790	Sis	0.381996836	1.13E-71
ENSMUSG00000010067	Rassf1	0.381902659	0.029311492
ENSMUSG00000034157	Cipc	0.381458372	0.000182056
ENSMUSG00000037674	Rfx7	0.381134039	0.000412556
ENSMUSG00000029090	Adgra3	0.380844146	0.029061948
ENSMUSG00000042628	Zfyve1	0.380620942	0.01962715
ENSMUSG00000034647	Ankrd12	0.380510632	2.01E-08
ENSMUSG00000019989	Ptprk	0.380294992	1.35E-07
ENSMUSG00000030741	Spns1	0.380278873	0.017283857
ENSMUSG00000078676	Casc3	0.380230693	0.003556087
ENSMUSG00000026987	Baz2b	0.379951667	0.00726097
ENSMUSG00000032340	Neo1	0.378900119	1.91E-07
ENSMUSG00000025198	Erlin1	0.378659118	5.90E-09
ENSMUSG00000021411	Pxdc1	0.378087494	8.22E-10
ENSMUSG00000026176	Ctdsp1	0.377935845	1.54E-07
ENSMUSG00000021540	Smad5	0.377603723	0.000205881
ENSMUSG00000020659	Cbll1	0.377580975	0.000550758
ENSMUSG00000029502	Golga3	0.377141238	0.000355004
ENSMUSG00000022364	Tbc1d31	0.376795956	3.61E-13
ENSMUSG00000036019	Tmtc2	0.376099545	0.029203545
ENSMUSG00000072825	Cep170b	0.375830159	1.05E-05
ENSMUSG00000029672	Fam3c	0.375634261	0.006365424
ENSMUSG00000024810	Il33	0.375512846	2.68E-12
ENSMUSG00000068876	Cgn	0.37529258	1.34E-10
ENSMUSG00000033781	Asb13	0.375241944	1.47E-17
ENSMUSG00000021975	Ints9	0.375033113	0.04118817
ENSMUSG00000063663	Brwd3	0.375014633	0.000913741
ENSMUSG00000043019	Edem3	0.373933427	7.53E-09
ENSMUSG00000028223	Decr1	0.373771895	7.57E-15
ENSMUSG00000034957	Cebpa	0.373386792	1.59E-06
ENSMUSG00000029705	Cux1	0.37316345	1.17E-13
ENSMUSG00000030779	Rbbp6	0.372868191	4.09E-12
ENSMUSG00000038369	Ncoa6	0.37205914	0.000898932
ENSMUSG00000002625	Akap8l	0.371747733	0.005549479
ENSMUSG00000034390	Cmip	0.371657805	0.031417028
ENSMUSG00000022816	Fstl1	0.371384929	0.005185075
ENSMUSG00000004655	Aqp1	0.371148277	8.85E-13
ENSMUSG00000054889	Dsp	0.371110133	4.59E-11
ENSMUSG00000039046	Usp6nl	0.370234262	0.009456619
ENSMUSG00000000631	Myo18a	0.370196748	8.41E-06
ENSMUSG00000026663	Atf6	0.369603466	1.58E-06
ENSMUSG00000028053	Ash1l	0.369541718	6.96E-06
ENSMUSG00000049658	Bdp1	0.369121696	1.47E-06
ENSMUSG00000051910	Sox6	0.368756885	0.019982045
ENSMUSG00000025314	Ptprj	0.368495592	1.70E-05
ENSMUSG00000024381	Bin1	0.368255303	0.000150298
ENSMUSG00000021990	Spata13	0.368150853	0.017029317
ENSMUSG00000034300	Fam53c	0.367728651	0.000600221
ENSMUSG00000045071	E130308A19	0.367704651	0.001768159
ENSMUSG00000044433	Camsap3	0.367615542	4.08E-11
ENSMUSG00000028385	Snx30	0.367420803	0.001282204
ENSMUSG00000033948	Zswim5	0.367080427	0.03047745
ENSMUSG00000074182	Znhit6	0.36627484	0.041138171
ENSMUSG00000039450	Dcxr	0.36599662	0.011620851
ENSMUSG00000017311	Pyy	0.365794398	1.64E-103
ENSMUSG00000029550	Sppl3	0.365680112	1.86E-11
ENSMUSG00000029545	Acads	0.364909895	0.004406103
ENSMUSG00000019158	Tmem160	0.364851955	1.10E-33
ENSMUSG00000020447	Npc1l1	0.36451388	1.55E-06
ENSMUSG00000041920	Slc16a6	0.36413695	5.17E-126
ENSMUSG00000039952	Dag1	0.363933032	2.82E-05
ENSMUSG00000035105	Egln3	0.363738611	3.74E-09
ENSMUSG00000019194	Cops6	0.363545639	0.008181493
ENSMUSG00000068134	Zfp120	0.363167834	6.65E-05
ENSMUSG00000096546	Smlr1	0.362620414	8.38E-37
ENSMUSG00000071659	Hnrnpul2	0.36242782	0.000164264
ENSMUSG00000066640	Fbxl18	0.361904061	0.000430141
ENSMUSG00000045932	Ifit2	0.361492442	5.81E-05
ENSMUSG00000054733	Msra	0.361191245	5.27E-18
ENSMUSG00000036528	Ppfibp2	0.361029296	0.039422707
ENSMUSG00000058655	Eif4b	0.360004823	7.57E-10
ENSMUSG00000021506	Pitx1	0.359654231	6.00E-06
ENSMUSG00000054256	Msi1	0.35848683	0.037914731
ENSMUSG00000037216	Lipt1	0.358160134	0.035234992
ENSMUSG00000013152	Hccs	0.357909076	7.00E-14
ENSMUSG00000023915	Tnfrsf21	0.357736131	0.000575101
ENSMUSG00000014905	Dnajb9	0.357690092	7.16E-09
ENSMUSG00000040918	Slc19a2	0.357585311	5.72E-06
ENSMUSG00000047264	Zfp358	0.357065035	0.003174179
ENSMUSG00000064351	COX1	0.356624117	3.07E-06
ENSMUSG00000031229	Atrx	0.356197446	2.35E-07
ENSMUSG00000074754	Smim26	0.356186443	6.57E-07
ENSMUSG00000046159	Chrm3	0.355790386	0.008001785
ENSMUSG00000032579	Hemk1	0.355760511	0.01118386
ENSMUSG00000020238	Ncln	0.355380278	0.039703869
ENSMUSG00000021496	Ube2d-ps	0.355353703	1.95E-05
ENSMUSG00000022994	Adcy6	0.354937509	1.10E-06
ENSMUSG00000021944	Gata4	0.354903989	2.33E-08
ENSMUSG00000059456	Ptk2b	0.35483764	0.008001785
ENSMUSG00000078942	Naip6	0.35470272	8.23E-07
ENSMUSG00000021366	Hivep1	0.354254524	6.04E-05

ENSMUSG00000000385	Tmprss2	-0.284623811	9.72E-21
ENSMUSG00000024140	Epas1	-0.284474823	0.020981507
ENSMUSG00000052396	Mogat2	-0.284050269	4.00E-07
ENSMUSG00000023031	Cela1	-0.283866212	3.10E-07
ENSMUSG00000039131	Gipc2	-0.28374876	2.49E-05
ENSMUSG00000090266	Mettl23	-0.283478586	0.016355893
ENSMUSG00000032763	Ilvbl	-0.28298577	0.005636538
ENSMUSG00000029710	Ephb4	-0.282376822	0.013151243
ENSMUSG00000033054	Npat	-0.282366264	0.016355893
ENSMUSG00000043153	Crppa	-0.282307452	0.006959974
ENSMUSG00000027820	Mme	-0.282249019	5.61E-91
ENSMUSG00000034300	Fam53c	-0.281899953	0.000600221
ENSMUSG00000028692	Akr1a1	-0.281665876	0.000567593
ENSMUSG00000021025	Nfkbia	-0.281403845	1.90E-12
ENSMUSG00000010914	Pdhx	-0.280927459	6.40E-07
ENSMUSG00000003154	Foxj2	-0.280545889	0.009317429
ENSMUSG00000058618	Defa39	-0.280269548	0.004630545
ENSMUSG00000024556	Me2	-0.279990305	2.55E-11
ENSMUSG00000033313	Fbxl8	-0.279705302	0.011062313
ENSMUSG00000071266	Zfp946	-0.279523221	0.030801814
ENSMUSG00000018548	Trim37	-0.279437386	5.45E-05
ENSMUSG00000041607	Mbp	-0.278885111	0.002866831
ENSMUSG00000078974	Sec61g	-0.278349128	3.16E-05
ENSMUSG00000002108	Nr1h3	-0.277416079	0.000142457
ENSMUSG00000048007	Timm8a1	-0.277172516	0.001773946
ENSMUSG00000023072	Cep89	-0.277131315	0.031886289
ENSMUSG00000006699	Cdc42	-0.277124005	5.13E-07
ENSMUSG00000027801	Tm4sf4	-0.277105118	1.76E-43
ENSMUSG00000042535	Gtpbp1	-0.276708329	0.000126167
ENSMUSG00000095385	D630033O11	-0.27629779	0.005267069
ENSMUSG00000068855	H2ac20	-0.276092328	0.038391136
ENSMUSG00000026070	Il18r1	-0.27556837	0.044795055
ENSMUSG00000059669	Taf1b	-0.275540527	0.01654663
ENSMUSG00000022575	Gsdmd	-0.275444544	6.17E-10
ENSMUSG00000110573	Gm5485	-0.274573546	3.30E-31
ENSMUSG00000032231	Anxa2	-0.274090831	0.004462294
ENSMUSG00000030084	Plxna1	-0.273477141	7.50E-05
ENSMUSG00000034042	Gpbp1l1	-0.273154691	1.66E-07
ENSMUSG00000027610	Gss	-0.272966285	0.000726248
ENSMUSG00000002812	Flii	-0.272388485	9.31E-07
ENSMUSG00000021877	Arf4	-0.272155645	5.65E-17
ENSMUSG00000026628	Atf3	-0.272029518	0.047056656
ENSMUSG00000042570	Mier2	-0.271963314	0.024554323
ENSMUSG00000071637	Cebpd	-0.271586577	1.70E-21
ENSMUSG00000018574	Acadvl	-0.271389028	1.94E-10
ENSMUSG00000037946	Fgd3	-0.271357135	0.001136289
ENSMUSG00000009566	Fpgs	-0.271104618	0.001428095
ENSMUSG00000028101	Pias3	-0.271100753	0.007362687
ENSMUSG00000023828	Slc22a3	-0.270427032	2.67E-09
ENSMUSG00000052837	Junb	-0.270407435	0.000411437
ENSMUSG00000030748	Il4ra	-0.270348834	0.000913741
ENSMUSG00000029407	Uso1	-0.269973434	3.80E-11
ENSMUSG00000042116	Vwa1	-0.26944157	2.58E-06
ENSMUSG00000037072	Selenof	-0.269307902	0.000173235
ENSMUSG00000011884	Gltp	-0.269190805	2.06E-18
ENSMUSG00000019143	Hars2	-0.268999141	6.25E-05
ENSMUSG00000046096	Mosmo	-0.268568374	0.036684687
ENSMUSG00000024340	Btnl2	-0.268465914	0.000365595
ENSMUSG00000031537	Ikbkb	-0.268372683	0.000385907
ENSMUSG00000074444	Defa30	-0.268265076	1.70E-05
ENSMUSG00000058799	Nap1l1	-0.268209843	6.61E-05
ENSMUSG00000018765	Fxr2	-0.268166889	0.019993235
ENSMUSG00000062127	Cttnbp2nl	-0.268131308	3.34E-07
ENSMUSG00000019996	Map7	-0.267857113	0.033029568
ENSMUSG00000038745	Nlrp6	-0.267534121	0.000365283
ENSMUSG00000031438	Rnf128	-0.267473804	9.71E-07
ENSMUSG00000028270	Gbp2	-0.267364454	0.000667002
ENSMUSG00000026111	Unc50	-0.267235598	0.016400404
ENSMUSG00000007041	Clic1	-0.266724528	3.51E-15
ENSMUSG00000029185	Fam114a1	-0.266617806	4.30E-05
ENSMUSG00000024217	Snrpc	-0.266308401	0.000599179
ENSMUSG00000024665	Fads2	-0.266046542	3.93E-15
ENSMUSG00000036813	Entpd8	-0.265961942	2.07E-13
ENSMUSG00000070473	Cldn3	-0.26578858	0.008968959
ENSMUSG00000022221	Ripk3	-0.265709292	7.90E-07
ENSMUSG00000020476	Dbnl	-0.265509469	9.13E-06
ENSMUSG00000074182	Znhit6	-0.265418786	0.041138171
ENSMUSG00000117924	Tmem223	-0.265030393	0.010561826
ENSMUSG00000074882	Cyp2c68	-0.265018613	3.18E-22
ENSMUSG00000022551	Cyc1	-0.264995397	2.09E-16
ENSMUSG00000009575	Cbx5	-0.26479193	1.54E-14
ENSMUSG00000028098	Rnf115	-0.264374867	0.039284188
ENSMUSG00000020846	Rflnb	-0.264161405	2.01E-05
ENSMUSG00000022546	Gpt	-0.264123539	3.76E-10
ENSMUSG00000079658	Eloc	-0.264114579	0.001657279
ENSMUSG00000002635	Pdcd2l	-0.263936994	0.001774511
ENSMUSG00000023262	Acy1	-0.263881682	8.74E-07
ENSMUSG00000020149	Rab1a	-0.26383850 1	2.36E-06
ENSMUSG00000091269	Gm6682	-0.263606133	0.001659308
ENSMUSG00000042225	Ammecr1	-0.2633566	3.62E-07
ENSMUSG00000057375	Yipf1	-0.263004005	0.002666373
ENSMUSG00000040747	Cd53	-0.262233542	0.016556244
ENSMUSG00000029179	Zcchc4	-0.262126562	0.026731623
ENSMUSG00000041836	Ptpre	-0.262111596	0.001086641
ENSMUSG00000056394	Lig1	-0.262090189	1.69E-08
ENSMUSG00000037722	Gnpnat1	-0.261662296	2.26E-06
ENSMUSG00000030275	Etnk1	-0.26143115	2.78E-06
ENSMUSG00000020089	Ppa1	-0.261116518	3.27E-77
ENSMUSG00000026558	Uck2	-0.260482855	4.94E-06
ENSMUSG00000039193	Nlrc4	-0.26018675	3.34E-08
ENSMUSG00000039826	Trub2	-0.260176719	4.57E-14
ENSMUSG00000072825	Cep170b	-0.259658947	1.05E-05
ENSMUSG00000026187	Xrcc5	-0.259595095	0.001978751
ENSMUSG00000039254	Pomt1	-0.259084171	0.034874097
ENSMUSG00000024371	C2	-0.258994562	1.58E-25
ENSMUSG00000042489	Clspn	-0.258646903	0.000296132
ENSMUSG00000036620	Mgat4b	-0.258130591	1.30E-15
ENSMUSG00000034607	Pof1b	-0.2579498	2.04E-07
ENSMUSG00000028063	Lmna	-0.257849228	1.46E-12
ENSMUSG00000022742	Cpox	-0.257778681	6.75E-08
ENSMUSG00000043501	Lgals2	-0.257687945	0.035681706
ENSMUSG00000095567	Noc2l	-0.25747371	2.80E-07
ENSMUSG00000030751	Psma1	-0.257461132	0.000109699
ENSMUSG00000045273	Cenph	-0.257157509	0.006013372
ENSMUSG00000020829	Slc46a1	-0.25688879	3.56E-107
ENSMUSG00000006599	Gtf2h1	-0.256426983	0.008170186
ENSMUSG00000035112	Wnk4	-0.256114872	0.000194793
ENSMUSG00000091780	Sco2	-0.256108894	3.15E-32
ENSMUSG00000033379	Atp6v0b	-0.255991954	8.60E-11
ENSMUSG00000019088	Dnase1l1	-0.255373212	0.000343535
ENSMUSG00000029430	Ran	-0.255346988	0.016790317
ENSMUSG00000027305	Ndufaf1	-0.255220669	0.019880491

ENSMUSG00000025401	Myo1a	0.309474324	1.32E-07
ENSMUSG00000032311	Nrg4	0.309386749	4.92E-09
ENSMUSG00000042606	Hirip3	0.308959169	0.026751239
ENSMUSG00000042342	Tmem161a	0.308803838	0.020981507
ENSMUSG00000028977	Casz1	0.308529364	1.14E-07
ENSMUSG00000078202	Nrarp	0.308008666	3.97E-06
ENSMUSG00000064138	Fam172a	0.308007836	2.32E-10
ENSMUSG00000035725	Prkx	0.307987436	0.014141363
ENSMUSG00000002731	Prkra	0.307684731	0.006173151
ENSMUSG00000030695	Aldoa	0.307562079	5.04E-08
ENSMUSG00000023236	Scg5	0.307243316	0.028911631
ENSMUSG00000020956	Dtd2	0.307217293	0.01539186
ENSMUSG00000031502	Col4a1	0.307194642	0.007133387
ENSMUSG00000029334	Prkg2	0.306753463	1.11E-18
ENSMUSG00000034485	Uaca	0.30664724	2.08E-11
ENSMUSG00000049232	Tigd2	0.306046673	0.037733281
ENSMUSG00000045757	Zfp764	0.305833667	0.048021844
ENSMUSG00000026621	Mtarc1	0.305776896	2.87E-06
ENSMUSG00000029249	Rest	0.305660918	2.01E-06
ENSMUSG00000026159	Agfg1	0.30559211	0.000340741
ENSMUSG00000032016	Lyrm2	0.305488666	0.000464429
ENSMUSG00000059363	Fxn	0.30546374	0.038521767
ENSMUSG00000031068	Glrx3	0.305450982	0.048820618
ENSMUSG00000030614	Tmem126b	0.305221466	0.002785078
ENSMUSG00000035047	Kri1	0.305177362	0.00051638
ENSMUSG00000018849	Wwc1	0.305162877	1.75E-07
ENSMUSG00000038990	Cables2	0.304785847	0.001385068
ENSMUSG00000037152	Ndufc1	0.304718357	2.12E-27
ENSMUSG00000020780	Srp68	0.30455091	0.001205993
ENSMUSG00000049577	Zfpm1	0.304425855	5.20E-08
ENSMUSG00000055553	Kxd1	0.303627669	0.044021187
ENSMUSG00000066233	Tmem42	0.303418709	4.81E-05
ENSMUSG00000062931	Zfp938	0.30338274	0.003742216
ENSMUSG00000026385	Dbi	0.302957156	2.52E-07
ENSMUSG00000024208	Uqcc2	0.302803404	0.001008049
ENSMUSG00000028381	Ugcg	0.302387357	0.001765034
ENSMUSG00000031862	Atp13a1	0.302280891	0.025108957
ENSMUSG00000026600	Soat1	0.302140921	0.009144356
ENSMUSG00000031229	Atrx	0.301924259	2.35E-07
ENSMUSG00000021194	Chga	0.301856718	0.017645961
ENSMUSG00000021843	Ktn1	0.301280249	0.003490042
ENSMUSG00000037531	Mrpl47	0.301022708	0.000972538
ENSMUSG00000034647	Ankrd12	0.300739168	2.01E-08
ENSMUSG00000039046	Usp6nl	0.300448261	0.009456619
ENSMUSG00000024777	Ppp2r5b	0.30019945	0.016317672
ENSMUSG00000027893	Ahcyl1	0.299489483	6.67E-42
ENSMUSG00000044792	Isca1	0.299470273	2.77E-06
ENSMUSG00000033174	Mgll	0.299431285	6.73E-09
ENSMUSG00000028517	Plpp3	0.299390876	0.002088176
ENSMUSG00000031442	Mcf2l	0.299356712	9.81E-11
ENSMUSG00000104623	Gm6394	0.298878532	0.001546549
ENSMUSG00000039183	Nubp2	0.298798878	0.008339738
ENSMUSG00000054342	Kcnn4	0.296928351	2.73E-10
ENSMUSG00000044881	Coa4	0.296603415	0.000506125
ENSMUSG00000031007	Atp6ap2	0.296555396	0.000324815
ENSMUSG00000036026	Tmem63b	0.296483465	1.03E-05
ENSMUSG00000057554	Lgals8	0.296438697	1.80E-07
ENSMUSG00000024413	Npc1	0.295750909	0.000284656
ENSMUSG00000016256	Ctsz	0.295568367	0.003282074
ENSMUSG00000078671	Chd2	0.295477976	0.002315008
ENSMUSG00000021684	Pde8b	0.295453529	2.10E-06
ENSMUSG00000104529	Rbakdn	0.294865659	0.0001419
ENSMUSG00000038481	Cdk19	0.294700021	1.05E-09
ENSMUSG00000034926	Dhcr24	0.294617305	4.96E-14
ENSMUSG00000019864	Rtn4ip1	0.294214867	0.014092674
ENSMUSG00000034708	Grn	0.294091389	0.003388521
ENSMUSG00000046027	Stard5	0.293837512	4.11E-05
ENSMUSG00000033998	Kcnk1	0.293562172	2.43E-11
ENSMUSG00000025525	Apool	0.293495295	8.05E-06
ENSMUSG00000037754	Ppp1r16b	0.293361059	1.76E-08
ENSMUSG00000007656	Arpp19	0.293329781	9.93E-07
ENSMUSG00000024483	Ankhd1	0.293291956	2.37E-05
ENSMUSG00000020402	Vdac1	0.293162846	0.022825865
ENSMUSG00000041037	Irgq	0.292825984	0.032945195
ENSMUSG00000083019	Gm6222	0.292625303	0.000247227
ENSMUSG00000024073	Birc6	0.29230234	1.62E-05
ENSMUSG00000006932	Ctnnb1	0.291972432	2.44E-15
ENSMUSG00000051910	Sox6	0.291823921	0.019982045
ENSMUSG00000032081	Apoc3	0.291496396	3.97E-50
ENSMUSG00000074211	Sdhaf1	0.2913112	1.88E-08
ENSMUSG00000030004	Nat8	0.291274165	1.04E-36
ENSMUSG00000039852	Rere	0.290793963	6.61E-15
ENSMUSG00000037458	Azin1	0.290651169	6.23E-27
ENSMUSG00000031352	Hccs	0.290640968	7.00E-14
ENSMUSG00000020988	L2hgdh	0.290540181	3.09E-20
ENSMUSG00000053293	Pom121	0.290362011	5.59E-05
ENSMUSG00000042133	Ppig	0.290318464	0.006373669
ENSMUSG00000062410	Hsd3b3	0.29017717	2.51E-09
ENSMUSG00000040502	Marchf9	0.289986925	0.003442564
ENSMUSG00000027247	Arhgap1	0.289894758	5.25E-22
ENSMUSG00000014361	Mertk	0.28972238	0.017809877
ENSMUSG00000032870	Smap2	0.289647419	0.031769501
ENSMUSG00000022477	Aco2	0.289605798	5.10E-11
ENSMUSG00000021460	Auh	0.289516094	1.36E-06
ENSMUSG00000050666	Vstm4	0.288788576	0.005376535
ENSMUSG00000049295	Zfp219	0.288253766	0.00120847
ENSMUSG00000064181	Rab3ip	0.288062414	1.78E-07
ENSMUSG00000025486	Sirt3	0.287862007	5.08E-07
ENSMUSG00000024587	Nars	0.287700087	1.26E-14
ENSMUSG00000002985	Apoe	0.286929641	0.003728077
ENSMUSG00000021792	Prxl2a	0.286908136	3.89E-198
ENSMUSG00000021149	Gtpbp4	0.286519092	0.011885359
ENSMUSG00000005360	Slc1a3	0.286463491	1.44E-17
ENSMUSG00000017664	Slc35c2	0.286313075	1.11E-09
ENSMUSG00000091625	Lsm5	0.286236084	0.006975797
ENSMUSG00000022961	Son	0.286093395	4.07E-06
ENSMUSG00000074829	2010315B03	0.285944342	1.52E-08
ENSMUSG00000001089	Luzp1	0.28593579	1.78E-09
ENSMUSG00000028161	Ppp3ca	0.285532422	4.79E-12
ENSMUSG00000026150	Mff	0.28551244	0.002231949
ENSMUSG00000026176	Ctdsp1	0.285422621	1.54E-07
ENSMUSG00000022906	Parp9	0.285413256	2.33E-08
ENSMUSG00000028282	Casp8ap2	0.285155107	0.001765034
ENSMUSG00000020017	Hal	0.284841076	5.11E-05
ENSMUSG00000008035	Mid1ip1	0.284817194	0.000665654
ENSMUSG00000027340	Slc23a2	0.284611818	2.61E-07
ENSMUSG00000030604	Zfp626	0.284041999	0.015514179
ENSMUSG00000018974	Sart3	0.283834576	0.024748481
ENSMUSG00000029426	Scarb2	0.283771668	0.0234826
ENSMUSG00000018736	Ndel1	0.283660522	0.006042998
ENSMUSG00000021114	Atp6v1d	0.283549609	0.009414367
ENSMUSG00000044042	Fmn1	0.283048281	2.78E-05

ENSMUSG00000029017	Pmpcb	-0.278836221	7.47E-06
ENSMUSG00000017390	Aldoc	-0.27857776	2.72E-20
ENSMUSG00000024844	Banf1	-0.278457879	1.13E-05
ENSMUSG00000023020	Cox14	-0.277829038	4.79E-09
ENSMUSG00000031527	Eri1	-0.277750615	0.001595691
ENSMUSG00000063888	Rpl7l1	-0.277733833	0.002166604
ENSMUSG00000016833	Mrps18c	-0.277705248	0.000809269
ENSMUSG00000002233	Rhoc	-0.277611065	1.26E-37
ENSMUSG00000033751	Gadd45gip1	-0.276981837	0.013630866
ENSMUSG00000003032	Klf4	-0.27692384	5.40E-13
ENSMUSG00000021040	Slirp	-0.276838407	0.002407021
ENSMUSG00000025465	Echs1	-0.276789454	1.59E-11
ENSMUSG00000025888	Casp1	-0.276697409	2.15E-08
ENSMUSG00000015880	Ncapg	-0.276441073	0.007399736
ENSMUSG00000021906	Oxnad1	-0.27614174	2.27E-13
ENSMUSG00000042570	Mier2	-0.275928413	0.024554323
ENSMUSG00000028165	Cisd2	-0.275742491	0.000157942
ENSMUSG00000056153	Socs6	-0.27535043	2.79E-06
ENSMUSG00000018921	Pelp1	-0.274627872	0.000308515
ENSMUSG00000048537	Phldb1	-0.274610857	3.76E-05
ENSMUSG00000025742	Prps2	-0.273065538	0.004146123
ENSMUSG00000029713	Gnb2	-0.273063315	0.00011614
ENSMUSG00000000708	Kat2b	-0.273030508	0.00432081
ENSMUSG00000074918	Inafm2	-0.272826038	5.26E-07
ENSMUSG00000052056	Zfp217	-0.272806038	0.017524734
ENSMUSG00000054474	Thnsl2	-0.27274104	0.003879406
ENSMUSG00000042962	Gm5436	-0.272716529	2.19E-09
ENSMUSG00000032202	Rab27a	-0.272690668	0.029014665
ENSMUSG00000028617	Lrrc42	-0.272206183	4.19E-16
ENSMUSG00000028779	Pef1	-0.272198655	1.11E-05
ENSMUSG00000041859	Mcm3	-0.271671664	0.000144759
ENSMUSG00000070394	Tmem256	-0.271630518	4.29E-05
ENSMUSG00000029447	Cct6a	-0.271532181	0.002059865
ENSMUSG00000026618	Iars2	-0.271385064	4.93E-08
ENSMUSG00000062963	Ufc1	-0.271062292	0.009811712
ENSMUSG00000073616	Cops9	-0.271012085	2.75E-08
ENSMUSG00000020899	Pfas	-0.270610832	7.72E-06
ENSMUSG00000041688	Amot	-0.270461107	0.00071503
ENSMUSG00000028010	Gar1	-0.270343232	1.02E-05
ENSMUSG00000115338	Pnp	-0.269873085	1.00E-30
ENSMUSG00000078812	Eif5a	-0.269850251	0.001920703
ENSMUSG00000018339	Gpx3	-0.269710216	7.35E-11
ENSMUSG00000038302	Afg1l	-0.269399014	0.016212652
ENSMUSG00000029472	Anapc5	-0.269318127	0.041061966
ENSMUSG00000029649	Pomp	-0.269233977	0.003713724
ENSMUSG00000029038	Ssu72	-0.269117377	4.64E-09
ENSMUSG00000039193	Nlrc4	-0.269023616	3.34E-08
ENSMUSG00000019851	Perp	-0.268776569	0.048314041
ENSMUSG00000031286	Glt28d2	-0.267996781	0.002832508
ENSMUSG00000021640	Naip1	-0.267852983	2.56E-12
ENSMUSG00000024387	Csnk2b	-0.267495737	0.001502127
ENSMUSG00000021764	Ndufs4	-0.266789476	2.43E-08
ENSMUSG00000025140	Pycr1	-0.266546567	0.000683781
ENSMUSG00000053119	Chmp3	-0.266521815	0.001207157
ENSMUSG00000021969	Zdhhc20	-0.266498264	1.21E-09
ENSMUSG00000047617	Paxx	-0.265664319	1.61E-08
ENSMUSG00000025289	Prdx4	-0.265558978	0.001842335
ENSMUSG00000028998	Tomm7	-0.265044548	0.014678503
ENSMUSG00000020205	Phlda1	-0.264830916	0.006705753
ENSMUSG00000044894	Uqcrq	-0.264442048	1.38E-11
ENSMUSG00000031960	Aars	-0.264151485	0.000532479
ENSMUSG00000063179	Pstk	-0.264075732	2.41E-05
ENSMUSG00000025940	Tmem70	-0.263875806	4.89E-06
ENSMUSG00000029198	Grpel1	-0.263855814	2.10E-09
ENSMUSG00000030483	Cyp2b10	-0.263840813	1.80E-39
ENSMUSG00000031516	Dctn6	-0.263677682	0.014948642
ENSMUSG00000018697	Aatf	-0.263584018	0.005351994
ENSMUSG00000029068	Ccnl2	-0.263432573	1.09E-05
ENSMUSG00000001156	Mxd1	-0.263091446	0.000326425
ENSMUSG00000021427	Ssr1	-0.263006911	0.032469343
ENSMUSG00000038690	Atp5j2	-0.262591038	5.93E-12
ENSMUSG00000032540	Abhd5	-0.262513391	6.86E-11
ENSMUSG00000022793	B4galt4	-0.262274676	9.94E-10
ENSMUSG00000056234	Ncoa4	-0.26217607	0.000550084
ENSMUSG00000021266	Wars1	-0.261703794	1.24E-08
ENSMUSG00000021573	Tppp	-0.261581366	7.03E-05
ENSMUSG00000034424	Gcsh	-0.261561093	0.002277266
ENSMUSG00000024993	Dennd10	-0.261139508	0.037824587
ENSMUSG00000029055	Plch2	-0.260544004	0.011798637
ENSMUSG00000032198	Dock6	-0.260220711	0.016708449
ENSMUSG00000091269	Gm6682	-0.259700216	0.001659308
ENSMUSG00000054619	Mettl7a1	-0.259664583	0.043501025
ENSMUSG00000034445	Cyb561a3	-0.2585762	0.016054813
ENSMUSG00000045160	Bola3	-0.258576112	4.18E-28
ENSMUSG00000060961	Slc4a4	-0.258526398	1.75E-10
ENSMUSG00000029610	Aimp2	-0.257774597	0.011347984
ENSMUSG00000104368	2010103J01R	-0.257556579	4.00E-14
ENSMUSG00000021731	Mrps30	-0.257462421	6.24E-07
ENSMUSG00000003033	Ap1m1	-0.257262898	0.049255473
ENSMUSG00000033186	Mzt1	-0.256783647	0.032357396
ENSMUSG00000047735	Samd9l	-0.256755588	1.49E-10
ENSMUSG00000027506	Tpd52	-0.2566684	0.004273545
ENSMUSG00000034610	Tut4	-0.256586023	2.61E-05
ENSMUSG00000046722	Cdc42se1	-0.256154614	0.034301603
ENSMUSG00000027082	Tfpi	-0.255630806	6.20E-25
ENSMUSG00000015247	Nipsnap3b	-0.255446025	0.000355004
ENSMUSG00000022205	Sub1	-0.255086251	0.000502241
ENSMUSG00000024215	Spdef	-0.254839363	7.71E-05
ENSMUSG00000028587	Orc1	-0.25467169	0.03167774
ENSMUSG00000070691	Runx3	-0.254088449	0.011130369
ENSMUSG00000022285	Ywhaz	-0.253912015	3.50E-11
ENSMUSG00000052305	Hbb-b1	-0.253585087	0.000179124
ENSMUSG00000035811	Ugt2b35	-0.253420557	2.32E-35
ENSMUSG00000022906	Parp9	-0.25338118	2.33E-08
ENSMUSG00000028156	Eif4e	-0.252634353	0.000543331
ENSMUSG00000033386	Frrs1	-0.252590698	4.46E-34
ENSMUSG00000050002	Idnk	-0.252303515	4.94E-09
ENSMUSG00000083992	Gm11478	-0.251978738	0.044235433
ENSMUSG00000027200	Sema6d	-0.251777743	0.033189239
ENSMUSG00000031934	Panx1	-0.251284374	2.35E-11
ENSMUSG00000027001	Dusp19	-0.250947036	0.005464815
ENSMUSG00000021458	Aopep	-0.250901236	0.047070601
ENSMUSG00000029632	Ndufa4	-0.250882621	1.35E-08
ENSMUSG00000021235	Coq6	-0.250457735	1.72E-05
ENSMUSG00000000563	Atp5pb	-0.250193761	5.29E-13
ENSMUSG00000039653	Baat	-0.250081697	0.010414355
ENSMUSG00000106918	Mrpl33	-0.250059917	0.007110926
ENSMUSG00000060548	Tnfrsf19	-0.25003132	0.001725285
ENSMUSG00000024535	Snx24	-0.2495161	0.039809059
ENSMUSG00000024854	Pold4	-0.249322311	0.009358511
ENSMUSG00000021930	Spryd7	-0.249268114	6.51E-15
ENSMUSG00000019998	Stx7	-0.249162816	1.57E-16

ENSMUSG00000019795	Pcmt1	0.353932681	4.82E-07
ENSMUSG00000028860	Sytl1	0.353625516	0.00066948
ENSMUSG00000067813	Xkr9	0.353525668	0.001082899
ENSMUSG00000061414	Cracr2a	0.353502347	1.71E-05
ENSMUSG00000038459	Abhd17c	0.353495475	0.011213722
ENSMUSG00000026589	Sec16b	0.353493073	1.25E-05
ENSMUSG00000020817	Rabep1	0.353101821	5.90E-06
ENSMUSG00000019996	Map7	0.353036714	0.033029568
ENSMUSG00000021456	Fbp2	0.352968383	5.47E-09
ENSMUSG00000034308	Sdr42e1	0.352940259	0.023198941
ENSMUSG00000022555	Dgat1	0.352610058	5.75E-07
ENSMUSG00000033054	Npat	0.352479587	0.016355893
ENSMUSG00000026235	Epha4	0.351889759	5.84E-12
ENSMUSG00000064373	Selenop	0.35155338	3.03E-12
ENSMUSG00000022641	Bbx	0.351266456	0.000950705
ENSMUSG00000028861	Mrps15	0.350499785	7.29E-10
ENSMUSG00000094483	Purb	0.350265631	0.003392795
ENSMUSG00000024044	Epb41l3	0.349711306	0.005030987
ENSMUSG00000024286	Ccny	0.349466095	0.02202254
ENSMUSG00000038949	Cnst	0.349001081	0.002387076
ENSMUSG00000007880	Arid1a	0.348407381	2.85E-09
ENSMUSG00000059498	Fcgr3	0.348394846	0.02700555
ENSMUSG00000048154	Kmt2d	0.34837777	0.020988512
ENSMUSG00000024219	Anks1	0.347993872	0.022047487
ENSMUSG00000021278	Amn	0.347637099	5.28E-09
ENSMUSG00000098207	Arl14	0.347629617	2.24E-11
ENSMUSG00000049606	Zfp644	0.347001976	0.00045216
ENSMUSG00000003746	Man1a	0.346921265	0.003745688
ENSMUSG00000004266	Ptpn6	0.34648027	0.014273307
ENSMUSG00000014547	Wdfy2	0.346132708	0.024666542
ENSMUSG00000024233	Spire1	0.346058626	0.018591429
ENSMUSG00000039087	Rreb1	0.345569095	0.000288229
ENSMUSG00000019873	Reep3	0.345527485	0.002486837
ENSMUSG00000034940	Synrg	0.34528617	3.69E-05
ENSMUSG00000034403	Pja1	0.344830843	2.64E-07
ENSMUSG00000027889	Ampd2	0.34458991	0.008663014
ENSMUSG00000053390	Zfp952	0.344309705	1.59E-05
ENSMUSG00000074218	Cox7a1	0.344145913	2.01E-45
ENSMUSG00000036501	Fam13b	0.344108426	0.04423346
ENSMUSG00000055670	Zzef1	0.343860682	0.025987712
ENSMUSG00000001604	Tcea3	0.343664251	1.55E-06
ENSMUSG00000029030	Tprgl	0.343385689	0.03548192
ENSMUSG00000034525	Ice1	0.343271981	0.0012208
ENSMUSG00000034931	Dhx8	0.342697419	0.000714428
ENSMUSG00000017453	Pipox	0.342131563	8.58E-29
ENSMUSG00000021687	Scamp1	0.341720213	7.54E-11
ENSMUSG00000031534	Smim19	0.341431074	5.85E-07
ENSMUSG00000035722	Abca7	0.341377159	0.044092504
ENSMUSG00000027984	Hadh	0.340953551	1.73E-32
ENSMUSG00000032599	Ip6k2	0.340914768	2.63E-05
ENSMUSG00000009905	Kdsr	0.340664482	3.72E-05
ENSMUSG00000047027	Rgmb	0.340398803	6.39E-05
ENSMUSG00000049154	Fam183b	0.340325742	3.61E-15
ENSMUSG00000019947	Arid5b	0.33998057	0.002315008
ENSMUSG00000079071	Slc28a2b	0.338574402	1.13E-13
ENSMUSG00000001418	Glmp	0.338296706	0.002491715
ENSMUSG00000070003	Ssbp4	0.338126278	5.55E-05
ENSMUSG00000021488	Nsd1	0.337848852	1.82E-05
ENSMUSG00000028037	Ifit3b	0.337505468	3.32E-12
ENSMUSG00000024079	Eif2ak2	0.337040415	0.04241373
ENSMUSG00000026385	Dbi	0.3369279	2.52E-07
ENSMUSG00000038331	Satb2	0.336061365	1.71E-14
ENSMUSG00000039031	Arhgap18	0.3357801	0.007910639
ENSMUSG00000024990	Rbp4	0.33562337	0.029213504
ENSMUSG00000040502	Marchf9	0.33536734	0.003442564
ENSMUSG00000024900	Cpt1a	0.335134602	1.57E-06
ENSMUSG00000024247	Pkdcc	0.335060069	0.024420475
ENSMUSG00000031834	Pik3r2	0.334902315	0.000783947
ENSMUSG00000004610	Etfb	0.334777877	5.65E-12
ENSMUSG00000038485	Socs7	0.33461967	0.00026345
ENSMUSG00000031105	Slc25a14	0.334288305	0.031298191
ENSMUSG00000031753	Cog4	0.33400987	0.005163594
ENSMUSG00000032594	Ip6k1	0.33342791	0.004174213
ENSMUSG00000063253	Scoc	0.332865327	2.10E-26
ENSMUSG00000029470	P2rx4	0.332755868	0.004271155
ENSMUSG00000097318	1700007L15	0.332711371	0.000497488
ENSMUSG00000096910	Zfp955b	0.332459832	0.000625905
ENSMUSG00000034785	Dio1	0.332416502	5.80E-05
ENSMUSG00000032119	Hinfp	0.331824149	0.027957546
ENSMUSG00000002748	Baz1b	0.331646919	3.98E-07
ENSMUSG00000026579	F5	0.331271184	2.67E-06
ENSMUSG00000024241	Sos1	0.331022877	0.019527073
ENSMUSG00000037936	Scarb1	0.33046997	1.33E-32
ENSMUSG00000071506	Tmem139	0.329839524	7.36E-11
ENSMUSG00000041846	Ppp4r3a	0.329519623	0.018101556
ENSMUSG00000038712	Mindy1	0.329514517	1.71E-15
ENSMUSG00000044254	Pcsk9	0.329486416	5.23E-28
ENSMUSG00000024743	Syt7	0.329403252	1.93E-14
ENSMUSG00000096145	Vkorc1	0.329314049	2.25E-15
ENSMUSG00000057706	Mex3b	0.328529411	4.15E-07
ENSMUSG00000009628	Tex15	0.328472494	0.001583783
ENSMUSG00000022141	Nipbl	0.328378959	1.08E-06
ENSMUSG00000020988	L2hgdh	0.328259006	3.09E-20
ENSMUSG00000005268	Prlr	0.327972775	2.99E-05
ENSMUSG00000061689	Dlgap4	0.327957374	0.019329608
ENSMUSG00000085683	Tmem238l	0.327956375	2.27E-07
ENSMUSG00000028292	Rars2	0.327056587	0.000598243
ENSMUSG00000061589	Dot1l	0.326987424	0.00736463
ENSMUSG00000047417	Rexo1	0.326487832	2.11E-07
ENSMUSG00000021244	Ylpm1	0.326350706	8.81E-09
ENSMUSG00000020173	Cobl	0.326285962	0.002351147
ENSMUSG00000030314	Atg7	0.326282925	4.39E-05
ENSMUSG00000028096	Gpr89	0.32605109	1.66E-13
ENSMUSG00000001225	Slc26a3	0.32551284	2.38E-07
ENSMUSG00000029165	Agbl5	0.32543446	0.002914419
ENSMUSG00000025764	Jade1	0.325383643	0.026491757
ENSMUSG00000060279	Ap2a1	0.324943901	0.034904457
ENSMUSG00000033295	Ptprf	0.324934933	2.49E-06
ENSMUSG00000028233	Tgs1	0.324906109	0.034983118
ENSMUSG00000025207	Sema4g	0.324763722	0.037365145
ENSMUSG00000090700	Cyp4f40	0.324677966	4.23E-22
ENSMUSG00000026116	Tmem131	0.324274243	3.93E-08
ENSMUSG00000052707	Tnrc6a	0.324225642	0.00394793
ENSMUSG00000025203	Scd2	0.324098878	1.32E-93
ENSMUSG00000038056	Kmt2c	0.324091046	0.00059979
ENSMUSG00000023904	Hcfc1r1	0.324049009	0.005442206
ENSMUSG00000048351	Coa7	0.324036929	0.002210487
ENSMUSG00000027935	Rab13	0.323716893	0.003649798
ENSMUSG00000074102	Rbm15b	0.32320429	5.97E-07
ENSMUSG00000042251	Pm20d1	0.323196324	0.009160416
ENSMUSG00000025790	Slco3a1	0.323192995	0.000250395
ENSMUSG00000049504	Proser1	0.323035375	0.021149436

ENSMUSG00000020321	Mdh1	-0.255133638	3.69E-09
ENSMUSG00000086841	2410006H16	-0.254907496	0.001499301
ENSMUSG00000001604	Tcea3	-0.254871784	1.55E-06
ENSMUSG00000004264	Phb2	-0.254805937	0.002246801
ENSMUSG00000026110	Mgat4a	-0.254223999	1.46E-15
ENSMUSG00000002741	Ykt6	-0.25414751	5.03E-06
ENSMUSG00000019189	Rnf145	-0.253889932	2.64E-12
ENSMUSG00000026726	Cubn	-0.25380455	1.07E-31
ENSMUSG00000007837	Prrg2	-0.253766775	6.09E-09
ENSMUSG00000017311	Pyy	-0.253589896	1.64E-103
ENSMUSG00000017221	Psmd3	-0.253262791	0.001035531
ENSMUSG00000006567	Atp7b	-0.252789103	6.09E-08
ENSMUSG00000024892	Pcx	-0.252714357	0.004761597
ENSMUSG00000029038	Ssu72	-0.252143524	4.64E-09
ENSMUSG00000052557	Gan	-0.251226944	9.07E-10
ENSMUSG00000028393	Alad	-0.250954645	9.00E-10
ENSMUSG00000030659	Nucb2	-0.250566706	4.79E-22
ENSMUSG00000034127	Tspan8	-0.250508614	2.29E-20
ENSMUSG00000024091	Vapa	-0.250450515	5.53E-10
ENSMUSG00000049800	Sertad2	-0.250258091	0.005393929
ENSMUSG00000078154	Gm12184	-0.24981763	0.018979183
ENSMUSG00000021286	Zfyve21	-0.249720764	0.016307069
ENSMUSG00000040128	Pnrc1	-0.249554653	6.22E-13
ENSMUSG00000115338	Pnp	-0.249193381	1.00E-30
ENSMUSG00000029864	Gstk1	-0.248748742	9.88E-47
ENSMUSG00000058569	Tmed9	-0.248508371	1.83E-14
ENSMUSG00000039285	Azi2	-0.24822696	0.002357239
ENSMUSG00000026333	Gin1	-0.248099372	2.22E-05
ENSMUSG00000039765	Cc2d2a	-0.247503773	8.39E-07
ENSMUSG00000062646	Ganc	-0.24746885	5.51E-05
ENSMUSG00000025364	Pa2g4	-0.246290453	0.013327827
ENSMUSG00000052397	Ezr	-0.246139786	6.22E-17
ENSMUSG00000019494	Cops6	-0.24590827	0.008181493
ENSMUSG00000027637	Rab5if	-0.245894797	8.79E-19
ENSMUSG00000027384	Ndufaf5	-0.245767317	3.50E-06
ENSMUSG00000022554	Hgh1	-0.245562095	0.017104211
ENSMUSG00000028127	Abcd3	-0.24543961	2.85E-25
ENSMUSG00000071369	Map3k5	-0.24521145	2.42E-05
ENSMUSG00000032119	Hinfp	-0.245156843	0.027957546
ENSMUSG00000040385	Ppp1ca	-0.24480389	0.041138171
ENSMUSG00000017716	Birc5	-0.244675566	1.65E-06
ENSMUSG00000024069	Slc30a6	-0.244515476	0.000971218
ENSMUSG00000078517	Emc1	-0.244247548	7.36E-05
ENSMUSG00000056938	Acbd4	-0.243406461	2.58E-14
ENSMUSG00000001270	Ckb	-0.243079081	2.14E-131
ENSMUSG00000022253	Nadk2	-0.243033219	4.96E-14
ENSMUSG00000010609	Psen2	-0.242629576	0.02359653
ENSMUSG00000027955	Gask1b	-0.242577039	1.61E-16
ENSMUSG00000021235	Coq6	-0.242505075	1.72E-05
ENSMUSG00000029726	Mepce	-0.242498152	0.036183086
ENSMUSG00000028890	Mtf1	-0.241885053	8.79E-15
ENSMUSG00000038150	Ormdl3	-0.241789731	1.97E-12
ENSMUSG00000067150	Xpo5	-0.241750067	0.009380488
ENSMUSG00000095026	Gm3336	-0.241487162	3.89E-08
ENSMUSG00000067219	Nipal1	-0.241397101	4.62E-13
ENSMUSG00000035069	Oma1	-0.24089192	0.000583543
ENSMUSG00000049489	Ccnq	-0.240318461	0.009451458
ENSMUSG00000061232	H2-K1	-0.240132617	0.031257952
ENSMUSG00000058407	Txndc9	-0.240131219	0.002717478
ENSMUSG00000026914	Psmd14	-0.239371397	0.000241646
ENSMUSG00000026798	Coq4	-0.239343359	3.07E-08
ENSMUSG00000029552	Tes	-0.239003649	0.011356575
ENSMUSG00000065987	Cd209b	-0.238870857	8.15E-06
ENSMUSG00000054766	Set	-0.238827609	7.29E-05
ENSMUSG00000057604	Lmcd1	-0.238702445	1.18E-10
ENSMUSG00000035356	Nfkbiz	-0.238307799	9.01E-14
ENSMUSG00000044627	Swi5	-0.237902266	1.66E-07
ENSMUSG00000018848	Rars	-0.237891503	0.020603866
ENSMUSG00000024875	Yif1a	-0.237798985	1.33E-15
ENSMUSG00000039349	C130074G19	-0.237765614	5.31E-12
ENSMUSG00000030795	Fus	-0.237699034	7.76E-05
ENSMUSG00000063856	Gpx1	-0.236583241	5.88E-25
ENSMUSG00000026883	Dab2ip	-0.235961109	1.35E-08
ENSMUSG00000029415	Sdad1	-0.235893584	0.000728793
ENSMUSG00000047446	Arl4a	-0.235497064	0.012876154
ENSMUSG00000034584	Exph5	-0.2354458	2.75E-12
ENSMUSG00000015932	Dstn	-0.235346254	8.50E-15
ENSMUSG00000030225	Dera	-0.235282569	1.50E-06
ENSMUSG00000036299	BC031181	-0.234959065	3.55E-07
ENSMUSG00000020737	Jpt1	-0.234591697	0.01172516
ENSMUSG00000027678	Ncoa3	-0.234405066	0.000879971
ENSMUSG00000039286	Fndc3b	-0.234258795	0.000805829
ENSMUSG00000070972	Dnajc25	-0.234110979	0.006435437
ENSMUSG00000074909	Ranbp6	-0.233889174	0.0014401
ENSMUSG00000040659	Efhd2	-0.233292921	5.00E-22
ENSMUSG00000017801	Mlx	-0.233171825	2.03E-10
ENSMUSG00000030613	Ccdc90b	-0.232975759	1.27E-10
ENSMUSG00000025439	Clns1a	-0.232657421	4.80E-05
ENSMUSG00000038879	Nipal2	-0.232551151	0.000689779
ENSMUSG00000103711	Tstd1	-0.23245047	2.67E-10
ENSMUSG00000024424	Ttc39c	-0.232346997	2.75E-21
ENSMUSG00000033427	Upb1	-0.232105533	1.56E-33
ENSMUSG00000046753	Ccdc66	-0.232060546	0.000241571
ENSMUSG00000018572	Phf23	-0.231901272	0.023972146
ENSMUSG00000068101	Cenpm	-0.231881587	0.024176019
ENSMUSG00000048351	Coa7	-0.231328473	0.002210487
ENSMUSG00000001630	Stk38l	-0.230519301	0.000874659
ENSMUSG00000053898	Ech1	-0.230263632	2.93E-10
ENSMUSG00000042460	C1galt1	-0.230133406	0.00116571
ENSMUSG00000022048	Dpysl2	-0.230028512	0.000855807
ENSMUSG00000020873	Slc35b1	-0.229541543	0.000478555
ENSMUSG00000020577	Tspan13	-0.229532599	1.14E-16
ENSMUSG00000042396	Rbm7	-0.22952873	3.36E-09
ENSMUSG00000017950	Hnf4a	-0.229398088	2.51E-11
ENSMUSG00000046909	Tefm	-0.22939432	0.02678648
ENSMUSG00000027968	Larp7	-0.229389207	8.43E-05
ENSMUSG00000105547	Iglc3	-0.228970209	0.031216573
ENSMUSG00000004610	Etfb	-0.228893562	5.65E-12
ENSMUSG00000030309	Caprin2	-0.228759338	0.001512029
ENSMUSG00000026821	Ralgds	-0.228644611	2.14E-06
ENSMUSG00000026708	Cenpl	-0.228543947	0.023076627
ENSMUSG00000030208	Emp1	-0.228310225	0.002900676
ENSMUSG00000046861	Hectd3	-0.228084817	0.004531913
ENSMUSG00000024958	Gpr137	-0.227544316	0.001988759
ENSMUSG00000099881	2810013P06	-0.227004503	0.031298191
ENSMUSG00000006519	Cyba	-0.22700336	4.64E-18
ENSMUSG00000052310	Slc39a1	-0.226181555	0.014625219
ENSMUSG00000004446	Bid	-0.226146581	9.23E-09
ENSMUSG00000095115	Itpripl2	-0.225357796	6.80E-19
ENSMUSG00000036834	Plch1	-0.224952398	0.031622732
ENSMUSG00000047731	Wbp1l	-0.224752873	0.003621695
ENSMUSG00000062526	Mppe1	-0.224733682	3.93E-09

ENSMUSG00000003423	Pih1d1	0.282977425	0.000145005
ENSMUSG00000031753	Cog4	0.282929663	0.005163594
ENSMUSG00000025498	Irf7	0.282520234	1.79E-08
ENSMUSG00000037007	Zfp113	0.282369409	0.04575066
ENSMUSG00000020029	Nudt4	0.282332667	5.09E-36
ENSMUSG00000070777	Ceacam20	0.282305465	5.61E-11
ENSMUSG00000022463	Srebf2	0.282189473	8.49E-05
ENSMUSG00000048920	Fkrp	0.281895197	0.002321765
ENSMUSG00000020782	Llgl2	0.28169041	0.000617831
ENSMUSG00000018593	Sparc	0.281567758	0.047318673
ENSMUSG00000045455	Gm9797	0.281128547	0.005001184
ENSMUSG00000006369	Fbln1	0.28101994	2.37E-17
ENSMUSG00000070372	Capza1	0.281009488	0.021198554
ENSMUSG00000026279	Thap4	0.280847789	1.71E-12
ENSMUSG00000055415	Atp10b	0.28039257	0.000117056
ENSMUSG00000024231	Cul2	0.280011903	0.006493082
ENSMUSG00000068391	Chrac1	0.279581956	6.18E-05
ENSMUSG00000057400	Ces1c	0.279410991	1.00E-05
ENSMUSG00000021079	Timm9	0.278925182	3.00E-10
ENSMUSG00000021577	Sdha	0.278909044	3.75E-05
ENSMUSG00000038733	Wdr26	0.278804794	1.39E-06
ENSMUSG00000036850	Mrpl41	0.278750315	0.001365656
ENSMUSG00000027936	Crtc2	0.278405736	0.041129844
ENSMUSG00000030770	Parva	0.278153919	0.00025828
ENSMUSG00000039158	Akna	0.277992562	0.009861212
ENSMUSG00000037236	Matr3	0.277515548	9.86E-05
ENSMUSG00000042810	Krba1	0.277422554	5.65E-06
ENSMUSG00000041736	Tspo	0.277089857	1.64E-10
ENSMUSG00000040054	Baz2a	0.276409194	0.001320004
ENSMUSG00000059409	Ppp2r5d	0.27638688	4.71E-08
ENSMUSG00000031958	Ldhd	0.276158972	1.14E-08
ENSMUSG00000020827	Mink1	0.276127315	9.43E-05
ENSMUSG00000007850	Hnrnph1	0.275849752	0.0177659
ENSMUSG00000023039	Krt7	0.275546252	0.00017484
ENSMUSG00000044224	Dnajc21	0.275004226	0.005817429
ENSMUSG00000078546	Zfp995	0.274931627	0.004839236
ENSMUSG00000009030	Pdcl	0.274695191	0.012849896
ENSMUSG00000106951	5930430L01	0.274363265	0.010453859
ENSMUSG00000061315	Naca	0.27401294	0.022397199
ENSMUSG00000037709	Fam13a	0.273939206	1.71E-68
ENSMUSG00000006818	Sod2	0.273860647	0.000144226
ENSMUSG00000021936	Mapk8	0.273726243	2.08E-17
ENSMUSG00000025937	Lactb2	0.273620547	0.004497466
ENSMUSG00000034751	Mast4	0.272999361	1.92E-08
ENSMUSG00000037270	Bltp1	0.272980198	0.004946374
ENSMUSG00000006362	Cbfa2t3	0.272643575	1.30E-07
ENSMUSG00000062691	Cebpzos	0.272606045	7.91E-12
ENSMUSG00000042962	Gm5436	0.272335167	2.19E-09
ENSMUSG00000033105	Lss	0.272175911	0.008348828
ENSMUSG00000049090	Ptgr3	0.271640348	0.000369346
ENSMUSG00000026170	Cyp27a1	0.271542691	0.001153652
ENSMUSG00000031642	Sh3rf1	0.271243997	1.79E-10
ENSMUSG00000022000	Zc3h13	0.271020825	0.029140189
ENSMUSG00000068134	Zfp120	0.270668361	6.65E-05
ENSMUSG00000021606	Ndufs6	0.270274374	2.38E-09
ENSMUSG00000020022	Ndufa12	0.269836893	1.30E-22
ENSMUSG00000044945	Tmem242	0.269808786	1.66E-07
ENSMUSG00000045095	Magi1	0.269275578	0.000499552
ENSMUSG00000040455	Usp45	0.269095669	0.048517154
ENSMUSG00000094483	Purb	0.268610869	0.003392795
ENSMUSG00000025512	Chid1	0.26851287	1.84E-07
ENSMUSG00000054309	Cpsf3	0.26846787	0.004392996
ENSMUSG00000041012	Cmtm8	0.268417652	1.57E-06
ENSMUSG00000041831	Sytl3	0.268112718	0.037730465
ENSMUSG00000039697	Ncoa7	0.267879672	5.39E-45
ENSMUSG00000019139	Isyna1	0.26733035	0.001226979
ENSMUSG00000068323	Slc4a5	0.267213078	4.31E-17
ENSMUSG00000032066	Bco2	0.266951541	2.32E-21
ENSMUSG00000031201	Brcc3	0.266751706	0.044091305
ENSMUSG00000041720	Pi4ka	0.266584124	0.005248254
ENSMUSG00000029722	Agfg2	0.266018284	0.008168715
ENSMUSG00000040134	Rdh7	0.265816664	7.83E-51
ENSMUSG00000046598	Bdh1	0.265784974	2.81E-16
ENSMUSG00000019907	Ppp1r12a	0.265775287	5.98E-08
ENSMUSG00000021102	Glrx5	0.265715429	3.80E-13
ENSMUSG00000059273	Zc3h4	0.265403138	0.022826568
ENSMUSG00000060613	Cyp2c70	0.264985523	7.35E-05
ENSMUSG00000018339	Gpx3	0.264885539	7.35E-11
ENSMUSG00000029267	Mtf2	0.26460258	0.037824587
ENSMUSG00000066406	Akap13	0.26442816	7.28E-18
ENSMUSG00000027342	Pcna	0.264335506	0.000916739
ENSMUSG00000020664	Dld	0.264312807	3.38E-17
ENSMUSG00000079215	Zfp664	0.263671131	8.11E-05
ENSMUSG00000005142	Man2b1	0.263578642	1.20E-05
ENSMUSG00000045598	Zfp553	0.263406613	0.002717478
ENSMUSG00000039263	Npepl1	0.263087795	0.007368425
ENSMUSG00000049882	Vcpkmt	0.2630491	1.45E-06
ENSMUSG00000029550	Sppl3	0.262624074	1.86E-11
ENSMUSG00000005087	Cd44	0.262542329	0.001807316
ENSMUSG00000101523	Csnk2a1-ps3	0.262480901	0.000485922
ENSMUSG00000001833	Septin7	0.262404286	0.000649255
ENSMUSG00000031534	Smim19	0.261400474	5.85E-07
ENSMUSG00000000184	Ccnd2	0.261209261	1.51E-15
ENSMUSG00000032187	Smarca4	0.26077863	5.21E-10
ENSMUSG00000038145	Snrk	0.26077825	0.021948434
ENSMUSG00000028334	Nans	0.260299163	0.000107609
ENSMUSG00000025630	Hprt	0.259858126	0.000491529
ENSMUSG00000019947	Arid5b	0.259231732	0.002315008
ENSMUSG00000036304	Zdhhc23	0.259190522	3.14E-05
ENSMUSG00000036565	Ttyh3	0.259164855	2.88E-18
ENSMUSG00000025813	Homer2	0.258775112	4.08E-12
ENSMUSG00000039670	Oxld1	0.258737444	0.00144598
ENSMUSG00000022350	Washc5	0.258411718	8.23E-05
ENSMUSG00000054065	Pkp3	0.25815846	2.63E-09
ENSMUSG00000053470	Kdm3a	0.258020712	0.00288652
ENSMUSG00000024151	Msh2	0.25786764	0.004569672
ENSMUSG00000046761	Fam83h	0.257820616	1.54E-08
ENSMUSG00000019971	Cep290	0.25772557	0.019315693
ENSMUSG00000026083	Eif5b	0.257541716	2.62E-08
ENSMUSG00000033307	Mif	0.257464523	1.24E-07
ENSMUSG00000053012	Krcc1	0.257249067	9.30E-06
ENSMUSG00000021109	Hif1a	0.256993177	0.00053532
ENSMUSG00000041025	Iffo2	0.256896445	0.049412373
ENSMUSG00000061759	Armt1	0.256890443	8.34E-06
ENSMUSG00000002957	Ap2a2	0.256752779	0.000535213
ENSMUSG00000050428	Fbxo46	0.256315236	4.22E-05
ENSMUSG00000033629	Hacd3	0.255629187	0.021605147
ENSMUSG00000089862	Umad1	0.255205085	0.017709793
ENSMUSG00000044791	Setd2	0.254974271	0.010159526
ENSMUSG00000048756	Foxo3	0.254180597	0.000191803
ENSMUSG00000029270	Dipk1a	0.253690277	0.001811975
ENSMUSG00000022973	Synj1	0.253565087	0.005459893

ENSMUSG00000030744	Rps3	-0.249112331	0.029491611
ENSMUSG00000032373	Car12	-0.248972493	5.32E-12
ENSMUSG00000004455	Ppp1cc	-0.248587945	0.014704092
ENSMUSG00000057497	Fam136a	-0.247996826	5.49E-10
ENSMUSG00000020946	Gosr2	-0.247950812	1.60E-06
ENSMUSG00000023243	Kcnk5	-0.247928476	0.000424806
ENSMUSG00000041881	Ndufa7	-0.24787677	6.97E-13
ENSMUSG00000097354	2310001H17	-0.247516949	0.025986568
ENSMUSG00000021496	Pcbd2	-0.247113267	0.000105556
ENSMUSG00000031765	Mt1	-0.247100487	6.60E-05
ENSMUSG00000028295	Smim8	-0.246971816	1.11E-05
ENSMUSG00000045503	Sys1	-0.246933352	0.013314323
ENSMUSG00000038252	Ncapd2	-0.246743168	0.023700542
ENSMUSG00000032246	Calml4	-0.246501425	5.54E-10
ENSMUSG00000030986	Dhx32	-0.246439219	1.30E-05
ENSMUSG00000005161	Prdx2	-0.246127111	5.37E-07
ENSMUSG00000038388	Pals2	-0.245998619	5.27E-10
ENSMUSG00000025487	Psmd13	-0.245851296	0.005133579
ENSMUSG00000044627	Swi5	-0.245710159	1.66E-07
ENSMUSG00000060487	Samd5	-0.245602773	5.19E-15
ENSMUSG00000044469	Tnfaip8l1	-0.24543714	1.50E-05
ENSMUSG00000042670	Immp1l	-0.245062742	0.008919567
ENSMUSG00000021967	Mrpl57	-0.245043291	0.005246525
ENSMUSG00000068566	Myadm	-0.245008145	6.11E-13
ENSMUSG00000025103	Btbd1	-0.24476666	0.046388134
ENSMUSG00000029397	Rchy1	-0.244764175	0.01555894
ENSMUSG00000026434	Nucks1	-0.243715998	3.56E-12
ENSMUSG00000029171	Pgm2	-0.242865374	1.20E-08
ENSMUSG00000045991	Onecut2	-0.24234659	2.21E-107
ENSMUSG00000022021	Diaph3	-0.242280562	0.025142669
ENSMUSG00000001436	Slc19a1	-0.242086555	2.30E-10
ENSMUSG00000031007	Atp6ap2	-0.242047832	0.000324815
ENSMUSG00000002010	Idh3g	-0.241483514	4.89E-10
ENSMUSG00000030788	Rnf141	-0.240795475	0.001376976
ENSMUSG00000020372	Rack1	-0.239908333	0.048021844
ENSMUSG00000024799	Tm7sf2	-0.239903575	2.18E-05
ENSMUSG00000079658	Eloc	-0.239815348	0.001657279
ENSMUSG00000051256	Jagn1	-0.239744714	0.012640472
ENSMUSG00000001289	Pfdn5	-0.23929784	0.001457869
ENSMUSG00000026568	Mpc2	-0.239142846	3.65E-21
ENSMUSG00000019579	Mydgf	-0.238571694	0.027264864
ENSMUSG00000028159	Dapp1	-0.238199325	0.01161518
ENSMUSG00000027305	Ndufaf1	-0.238057728	0.019880491
ENSMUSG00000042712	Tceal9	-0.238056598	3.37E-21
ENSMUSG00000057762	Gm6169	-0.237813242	0.04605327
ENSMUSG00000045237	Eola1	-0.237204522	5.18E-05
ENSMUSG00000005102	Eif2ak4	-0.236843396	0.000367421
ENSMUSG00000038845	Phb	-0.23677449	4.52E-05
ENSMUSG00000020225	Tmbim4	-0.236589026	0.000944136
ENSMUSG00000024885	Aldh3b1	-0.236347536	0.002794355
ENSMUSG00000018848	Rars	-0.236339125	0.020603866
ENSMUSG00000039294	Cybc1	-0.236291592	0.001986562
ENSMUSG00000032300	1700017B05	-0.236096597	0.002672609
ENSMUSG00000030846	Tial1	-0.23609483	0.000550221
ENSMUSG00000022817	Itgb5	-0.23491479	0.00228574
ENSMUSG00000032278	Paqr5	-0.234818457	2.18E-07
ENSMUSG00000014907	Naf1	-0.23480894	0.037447539
ENSMUSG00000035885	Cox8a	-0.234691138	4.79E-09
ENSMUSG00000061666	Gdpd1	-0.234529401	4.37E-89
ENSMUSG00000021809	Nudt13	-0.234339576	5.37E-05
ENSMUSG00000019952	Poc1b	-0.233762876	3.67E-05
ENSMUSG00000029918	Mrps33	-0.23362867	4.54E-13
ENSMUSG00000089872	Rps6kc1	-0.233553201	0.03463141
ENSMUSG00000062825	Actg1	-0.232964718	0.047736318
ENSMUSG00000022890	Atp5j	-0.232812054	8.38E-05
ENSMUSG00000022781	Pak2	-0.232695121	0.000226371
ENSMUSG00000036438	Calm2	-0.23260388	0.017199492
ENSMUSG00000032625	Thsd7a	-0.23221588	9.76E-06
ENSMUSG00000019054	Fis1	-0.232184181	2.61E-05
ENSMUSG00000066798	Zbtb6	-0.232126605	0.001250781
ENSMUSG00000020048	Hsp90b1	-0.232122234	1.79E-09
ENSMUSG00000022807	Osbpl11	-0.231784654	0.007142108
ENSMUSG00000029048	Rer1	-0.231072615	8.04E-05
ENSMUSG00000021876	Rnase4	-0.230901962	2.02E-08
ENSMUSG00000024985	Tcf7l2	-0.230900299	8.63E-06
ENSMUSG00000045659	Plekha7	-0.230667541	0.047755611
ENSMUSG00000027901	Dennd2d	-0.230479021	8.45E-13
ENSMUSG00000040613	Apobec1	-0.230318465	4.83E-12
ENSMUSG00000002416	Ndufb2	-0.23024329	5.31E-16
ENSMUSG00000025138	Sirt7	-0.230182723	0.007848567
ENSMUSG00000034320	Slc26a2	-0.22962304	5.27E-83
ENSMUSG00000031201	Brcc3	-0.229538673	0.044091305
ENSMUSG00000003423	Pih1d1	-0.229289684	0.000145005
ENSMUSG00000028760	Eif4g3	-0.228995985	3.02E-07
ENSMUSG00000030275	Etnk1	-0.228745166	2.78E-06
ENSMUSG00000054405	Dnajc8	-0.228307012	0.001661489
ENSMUSG00000052087	Rgs14	-0.228233414	4.18E-08
ENSMUSG00000020477	Mrps24	-0.227730373	4.81E-06
ENSMUSG00000119584	Rn18s	-0.227621621	0.021790683
ENSMUSG00000046804	Phgr1	-0.22726928	4.53E-11
ENSMUSG00000020023	Tmcc3	-0.226864895	3.55E-13
ENSMUSG00000024360	Etf1	-0.226845408	0.001456417
ENSMUSG00000024966	Stip1	-0.226568745	0.009622221
ENSMUSG00000009575	Cbx5	-0.226314194	1.54E-14
ENSMUSG00000038582	Pptc7	-0.226039279	0.000637478
ENSMUSG00000035953	Pip4p1	-0.225969385	0.028064487
ENSMUSG00000009013	Dynll1	-0.225801354	0.000930772
ENSMUSG00000043091	Tuba1c	-0.225795524	7.15E-09
ENSMUSG00000096001	Gpr15lg	-0.225454979	1.97E-13
ENSMUSG00000029199	Lias	-0.225336429	1.32E-08
ENSMUSG00000036751	Cox6b1	-0.224955294	7.68E-14
ENSMUSG00000024317	Rnf138	-0.224530312	0.03430665
ENSMUSG00000021290	Atp5mpl	-0.224490777	2.37E-06
ENSMUSG00000022070	Bora	-0.223632431	3.60E-05
ENSMUSG00000025612	Bach1	-0.223603574	0.034751082
ENSMUSG00000042111	Ccdc115	-0.223577226	0.008130464
ENSMUSG00000004535	Tax1bp1	-0.223393578	0.001297722
ENSMUSG00000058254	Tspan7	-0.223123891	1.85E-10
ENSMUSG00000059995	Atxn7l3	-0.222893929	0.016422899
ENSMUSG00000060002	Chpt1	-0.222859981	6.12E-136
ENSMUSG00000043885	Slc36a4	-0.222801486	0.007327965
ENSMUSG00000041361	Myzap	-0.222534775	0.000204474
ENSMUSG00000041488	Stx3	-0.222328764	0.00904923
ENSMUSG00000040112	Mrps35	-0.222204781	6.24E-06
ENSMUSG00000026994	Galnt3	-0.222090226	0.015694482
ENSMUSG00000024066	Xdh	-0.221890038	1.15E-10
ENSMUSG00000075467	Dnlz	-0.221835125	0.008394779
ENSMUSG00000036632	Alg5	-0.221723623	0.001926304
ENSMUSG00000037152	Ndufc1	-0.221711482	2.12E-27
ENSMUSG00000024222	Fkbp5	-0.221547027	1.07E-06
ENSMUSG00000041926	Rnpep	-0.221162333	0.000268604
ENSMUSG00000026500	Cox20	-0.220961808	0.035315011

ENSMUSG00000029723	Spacdr	0.322689361	0.000453139
ENSMUSG00000024841	Eif1ad	0.322539311	8.22E-26
ENSMUSG00000037331	Larp1	0.322371017	3.75E-11
ENSMUSG00000022292	Rrm2b	0.322112221	0.023642109
ENSMUSG00000034168	Irf2bpl	0.321979105	5.08E-05
ENSMUSG00000024597	Slc12a2	0.321753145	4.55E-13
ENSMUSG00000001983	Taco1	0.321395877	2.58E-06
ENSMUSG00000016494	Cd34	0.32089177	0.011209708
ENSMUSG00000002546	Golga2	0.320731283	0.000257149
ENSMUSG00000046447	Camk2n1	0.319891697	0.006145375
ENSMUSG00000040760	Appl1	0.319301987	1.99E-05
ENSMUSG00000038708	Golga4	0.319251009	1.71E-05
ENSMUSG00000051224	Tceanc	0.318943221	0.006328797
ENSMUSG00000033306	Lpp	0.31893941	5.11E-06
ENSMUSG00000026932	Nacc2	0.318934855	0.001046018
ENSMUSG00000040220	Gas8	0.318885724	0.040524515
ENSMUSG00000021928	Ebpl	0.318388899	0.048646406
ENSMUSG00000024558	Mapk4	0.318170002	1.17E-09
ENSMUSG00000071054	Safb	0.317698525	0.003153346
ENSMUSG00000074064	Mlycd	0.317264288	7.77E-06
ENSMUSG00000032667	Pon2	0.317209748	3.74E-21
ENSMUSG00000025026	Add3	0.316750751	2.11E-17
ENSMUSG00000033863	Klf9	0.315525354	4.34E-15
ENSMUSG00000031712	Il15	0.315514848	5.27E-07
ENSMUSG00000031906	Smpd3	0.315262409	1.06E-11
ENSMUSG00000002957	Ap2a2	0.315220098	0.000535213
ENSMUSG00000037536	Fbxo34	0.31488732	0.004647615
ENSMUSG00000033055	Ankrd54	0.314514426	0.00018191
ENSMUSG00000030094	Xpc	0.313875139	0.028064487
ENSMUSG00000024294	Mib1	0.313774869	0.008567265
ENSMUSG00000020859	Spag9	0.313682355	0.002908508
ENSMUSG00000027086	Fastkd1	0.313239169	0.049544718
ENSMUSG00000043162	Pyurf	0.312818694	4.77E-05
ENSMUSG00000063142	Kcnma1	0.312523933	0.01172516
ENSMUSG00000025497	Cdhr5	0.312428318	0.0021332
ENSMUSG00000044252	Osbpl1a	0.312300539	2.72E-09
ENSMUSG00000059273	Zc3h4	0.311962558	0.022826568
ENSMUSG00000034903	Cobll1	0.311621032	0.002850089
ENSMUSG00000039741	Bahcc1	0.310662803	0.015002271
ENSMUSG00000020780	Srp68	0.310487875	0.001205993
ENSMUSG00000017478	Zc3h18	0.310194448	0.000424472
ENSMUSG00000048473	Sult6b2	0.310133869	7.86E-21
ENSMUSG00000073424	Cyp4f15	0.310119424	1.15E-05
ENSMUSG00000032572	Col6a4	0.310118705	3.94E-06
ENSMUSG00000043673	Kcns3	0.310118547	9.94E-13
ENSMUSG00000032769	Trpa1	0.310117948	1.73E-05
ENSMUSG00000015714	Cers2	0.309876724	0.02204387
ENSMUSG00000021957	Tkt	0.309546191	3.72E-07
ENSMUSG00000017830	Dhx58	0.309489598	7.42E-19
ENSMUSG00000025137	Pcyt2	0.309316704	6.88E-07
ENSMUSG00000079020	Slc45a4	0.309212735	0.002210559
ENSMUSG00000029605	Oas1b	0.308997142	2.33E-08
ENSMUSG00000020454	Eif4enif1	0.308922248	0.016634244
ENSMUSG00000042121	Ssh1	0.308440501	0.025501597
ENSMUSG00000077450	Rab11b	0.308419168	0.000180503
ENSMUSG00000087366	Junos	0.308394932	0.010453859
ENSMUSG00000015961	Adss	0.308293043	6.81E-11
ENSMUSG00000032724	Abtb2	0.308228729	0.044995924
ENSMUSG00000046027	Stard5	0.308067524	4.11E-05
ENSMUSG00000074733	Zfp950	0.307745986	0.018289775
ENSMUSG00000017718	Afmid	0.307680425	0.011908367
ENSMUSG00000073436	Eme2	0.307589824	0.002862161
ENSMUSG00000039953	Clstn1	0.307517412	1.76E-12
ENSMUSG00000029811	Aoc1	0.307452795	1.14E-05
ENSMUSG00000075419	Dolk	0.307423309	0.047172472
ENSMUSG00000029063	Nadk	0.30729217	0.007115351
ENSMUSG00000000282	Mnt	0.306799567	0.011983913
ENSMUSG00000030766	Arhgap17	0.306370182	0.041101873
ENSMUSG00000042178	Armc5	0.306132687	4.35E-05
ENSMUSG00000067369	Trmt2b	0.305757781	2.23E-08
ENSMUSG00000020766	Galk1	0.305437969	4.39E-16
ENSMUSG00000022983	Scaf4	0.305358322	3.04E-07
ENSMUSG00000026069	Il1rl1	0.305326615	0.017557016
ENSMUSG00000010066	Cacna2d2	0.304501736	0.02404627
ENSMUSG00000045216	Hs6st1	0.304469986	1.27E-08
ENSMUSG00000068856	Sf3b4	0.303926717	4.41E-05
ENSMUSG00000025732	Mcrip2	0.303797993	3.89E-10
ENSMUSG00000003199	Mpnd	0.303410404	0.010688445
ENSMUSG00000031853	Map3k21	0.303206025	0.000172357
ENSMUSG00000024397	Aif1	0.302784032	0.002604148
ENSMUSG00000059654	Reg1	0.302200046	8.56E-92
ENSMUSG00000052712	BC004004	0.302035374	0.038407852
ENSMUSG00000028917	Plekhm2	0.301760279	0.048034937
ENSMUSG00000034663	Bmp2k	0.301733238	4.55E-10
ENSMUSG00000024483	Ankhd1	0.301630887	2.37E-05
ENSMUSG00000020829	Slc46a1	0.301608367	3.56E-107
ENSMUSG00000028479	Gne	0.301580403	0.014104411
ENSMUSG00000018547	Pip4k2b	0.301153497	0.002074464
ENSMUSG00000021371	Mcur1	0.301044607	0.001955254
ENSMUSG00000071654	Uqcc3	0.300842325	0.000505953
ENSMUSG00000037025	Foxa2	0.300507115	0.036544717
ENSMUSG00000033685	Ucp2	0.299869092	4.37E-06
ENSMUSG00000063646	Jakmip1	0.298923303	0.015500911
ENSMUSG00000026883	Dab2ip	0.298781044	1.35E-08
ENSMUSG00000025019	Lcor	0.298050802	2.83E-06
ENSMUSG00000029599	Ddx54	0.297066121	1.00E-06
ENSMUSG00000051177	Plcb1	0.296927903	0.005348814
ENSMUSG00000041644	Slc5a12	0.296854269	0.039837035
ENSMUSG00000020850	Prpf8	0.29660033	0.000140648
ENSMUSG00000069601	Ank3	0.29657817	0.000748388
ENSMUSG00000003316	Glg1	0.296545339	1.51E-10
ENSMUSG00000056268	Dennd1b	0.296492033	0.004474675
ENSMUSG00000025782	Taf3	0.296386314	0.000248451
ENSMUSG00000003068	Stk11	0.296223611	0.027507502
ENSMUSG00000020099	Unc5b	0.296049209	1.68E-09
ENSMUSG00000031621	Isx	0.295825409	2.79E-06
ENSMUSG00000020091	Eif4ebp2	0.295820701	3.08E-20
ENSMUSG00000040584	Abcb1a	0.29572438	1.12E-09
ENSMUSG00000079470	Utp14b	0.29554943	0.003315543
ENSMUSG00000022895	Ets2	0.295417017	1.85E-05
ENSMUSG00000023169	Slc38a1	0.295045723	0.001492697
ENSMUSG00000095105	Edaradd	0.294843659	8.83E-09
ENSMUSG00000030278	Cidec	0.294612	1.28E-07
ENSMUSG00000039701	Usp53	0.294365988	0.005257668
ENSMUSG00000020196	Cabin1	0.294344253	0.016519329
ENSMUSG00000029313	Aff1	0.294217191	3.40E-08
ENSMUSG00000038084	Opa1	0.294030776	0.026925643
ENSMUSG00000032217	Rnf111	0.293809209	0.02202254
ENSMUSG00000002968	Med25	0.29380598	0.000240937
ENSMUSG00000019806	Aig1	0.293759419	0.000658285
ENSMUSG00000022246	Rai14	0.293202712	0.018215079
ENSMUSG00000030035	Wbp1	0.293109101	0.039111291

ENSMUSG00000048992	Prss32	-0.224418204	3.22E-15
ENSMUSG00000034168	Irf2bpl	-0.22435992	5.08E-05
ENSMUSG00000048534	Jaml	-0.224049358	3.63E-12
ENSMUSG00000068749	Psma5	-0.222614422	0.004968592
ENSMUSG00000060373	Hnrnpc	-0.222555157	0.000952451
ENSMUSG00000024384	Iws1	-0.222316795	0.001546016
ENSMUSG00000022853	Ehhadh	-0.222149178	0.023344525
ENSMUSG00000060716	Plekhh1	-0.221951095	0.011223877
ENSMUSG00000059182	Skap2	-0.221609381	7.88E-11
ENSMUSG00000037580	Gch1	-0.221283861	1.07E-09
ENSMUSG00000030788	Rnf141	-0.220958731	0.001376976
ENSMUSG00000038365	Fbxo25	-0.220711537	0.004424072
ENSMUSG00000058546	Rpl23a	-0.22070762	0.000223612
ENSMUSG00000018401	Mtmr4	-0.220649528	0.04399908
ENSMUSG00000050777	Tmem37	-0.220469549	2.07E-24
ENSMUSG00000072115	Ang	-0.220453292	3.60E-06
ENSMUSG00000028563	Tm2d1	-0.220262468	0.002397492
ENSMUSG00000023904	Hcfc1r1	-0.220217837	0.005442206
ENSMUSG00000024966	Stip1	-0.219951272	0.009622221
ENSMUSG00000004849	Ap1s1	-0.219855329	0.000117374
ENSMUSG00000018559	Ctdnep1	-0.219651028	0.000385292
ENSMUSG00000015176	Nolc1	-0.219571134	0.031546335
ENSMUSG00000027184	Caprin1	-0.218896485	0.042573484
ENSMUSG00000032786	Alas1	-0.217579911	0.000587982
ENSMUSG00000024863	Mbl2	-0.217468088	0.001191412
ENSMUSG00000038437	Mllt6	-0.216992954	1.06E-06
ENSMUSG00000026031	Cflar	-0.216589285	0.047840264
ENSMUSG00000034006	Slc66a2	-0.21617702	0.03195251
ENSMUSG00000090935	Synj2bp	-0.215557735	2.46E-05
ENSMUSG00000032388	Spg21	-0.215528613	0.027159598
ENSMUSG00000046417	Lrrc75a	-0.215180672	0.004147725
ENSMUSG00000020841	Cpd	-0.215158926	3.20E-12
ENSMUSG00000032098	Treh	-0.214916227	6.66E-77
ENSMUSG00000028419	Chmp5	-0.214751102	0.007532518
ENSMUSG00000021411	Pxdc1	-0.214566938	8.22E-10
ENSMUSG00000053253	Ndfip2	-0.214432465	5.70E-05
ENSMUSG00000037519	Ppfia1	-0.213370241	0.001088741
ENSMUSG00000095813	Defa-ps1	-0.213041334	0.009631029
ENSMUSG00000004562	Arhgef40	-0.212864696	0.01323804
ENSMUSG00000025487	Psmd13	-0.21215724	0.005133579
ENSMUSG00000039616	Mocos	-0.211782968	3.73E-07
ENSMUSG00000054277	Arfgap3	-0.210809311	2.87E-05
ENSMUSG00000002395	Use1	-0.210004382	0.002468176
ENSMUSG00000046324	Ermp1	-0.209914329	0.047106589
ENSMUSG00000029319	Coq2	-0.209745755	0.000390206
ENSMUSG00000028988	Ctnnbip1	-0.209702853	0.013063757
ENSMUSG00000029811	Aoc1	-0.209681306	1.14E-05
ENSMUSG00000070003	Ssbp4	-0.209234618	5.55E-05
ENSMUSG00000059278	Naa38	-0.208781889	0.00039526
ENSMUSG00000038803	Ost4	-0.208554004	7.60E-05
ENSMUSG00000042506	Usp22	-0.208413267	0.000193735
ENSMUSG00000029178	Klf3	-0.208406309	1.73E-11
ENSMUSG00000031320	Rps4x	-0.208404107	1.63E-09
ENSMUSG00000029994	Anxa4	-0.208200294	4.36E-35
ENSMUSG00000020775	Mrpl38	-0.208035602	0.009167341
ENSMUSG00000001864	Aif1l	-0.207617317	0.020343029
ENSMUSG00000026547	Tagln2	-0.207255793	0.001404727
ENSMUSG00000052062	Pard3b	-0.207170937	2.73E-15
ENSMUSG00000030766	Arhgap17	-0.207139898	0.041101873
ENSMUSG00000048076	Arf1	-0.207119779	0.012849896
ENSMUSG00000037851	Iars	-0.206912563	0.021047305
ENSMUSG00000021500	Ddx46	-0.206907132	2.26E-05
ENSMUSG00000078945	Naip2	-0.206746882	0.005131288
ENSMUSG00000078153	Psme2b	-0.206474647	1.85E-11
ENSMUSG00000024354	Slc23a1	-0.206361882	0.00063384
ENSMUSG00000036368	Rmdn2	-0.205987246	0.001019291
ENSMUSG00000030616	Sytl2	-0.205952463	1.25E-08
ENSMUSG00000045103	Dmd	-0.205892148	0.006240103
ENSMUSG00000032290	Ptpn9	-0.205828048	3.84E-08
ENSMUSG00000062866	Phactr2	-0.205781955	8.34E-07
ENSMUSG00000027187	Cat	-0.205674765	8.21E-25
ENSMUSG00000090136	Gm10177	-0.205628458	1.06E-06
ENSMUSG00000028332	Hemgn	-0.205368149	3.44E-09
ENSMUSG00000036281	Snapc4	-0.205248304	0.024274076
ENSMUSG00000049922	Slc35c1	-0.20505228	0.000794929
ENSMUSG00000004980	Hnrnpa2b1	-0.204717579	0.001156317
ENSMUSG00000038312	Edem2	-0.204642781	0.004998049
ENSMUSG00000006638	Abhd1	-0.204562187	4.24E-24
ENSMUSG00000023914	Mep1a	-0.204265761	9.65E-151
ENSMUSG00000042349	Ikbke	-0.204101484	2.74E-13
ENSMUSG00000036513	Commd2	-0.203325179	5.09E-06
ENSMUSG00000038384	Setd1b	-0.203051469	8.56E-11
ENSMUSG00000063888	Rpl7l1	-0.203018425	0.002166604
ENSMUSG00000027001	Dusp19	-0.202883018	0.005464815
ENSMUSG00000070873	Lilra5	-0.202792331	0.017854923
ENSMUSG00000036186	Dipk1b	-0.202588618	0.000103815
ENSMUSG00000052712	BC004004	-0.20255989	0.038407852
ENSMUSG00000028255	Clca1	-0.202442578	2.01E-11
ENSMUSG00000028413	B4galt1	-0.20243702	4.08E-16
ENSMUSG00000032413	Rasa2	-0.202375679	0.011202067
ENSMUSG00000020225	Tmbim4	-0.202319012	0.000944136
ENSMUSG00000009378	Slc16a12	-0.202100255	0.046206365
ENSMUSG00000059005	Hnrnpa3	-0.202041302	1.36E-05
ENSMUSG00000017057	Il13ra1	-0.201949161	9.51E-06
ENSMUSG00000024044	Epb41l3	-0.201835803	0.005030987
ENSMUSG00000041660	Bbox1	-0.20153582	1.67E-41
ENSMUSG00000033308	Dpyd	-0.201241247	3.75E-05
ENSMUSG00000026853	Crat	-0.201155316	6.42E-07
ENSMUSG00000020868	Xylt2	-0.200967847	0.001967403
ENSMUSG00000026828	Galnt5	-0.20080219	0.000307404
ENSMUSG00000029918	Mrps33	-0.200670805	4.54E-13
ENSMUSG00000016319	Slc25a5	-0.200473173	2.44E-08
ENSMUSG00000054435	Gimap4	-0.200068342	3.51E-05
ENSMUSG00000021556	Golm1	-0.199481484	5.24E-24
ENSMUSG00000030282	Cmas	-0.199412888	0.004257107
ENSMUSG00000045659	Plekha7	-0.199072149	0.047755611
ENSMUSG00000079659	Tmem243	-0.198926501	0.000344906
ENSMUSG00000033392	Clasp2	-0.197997216	0.001063144
ENSMUSG00000041765	Ubac2	-0.197917425	0.049040342
ENSMUSG00000028158	Mttp	-0.197349618	3.05E-39
ENSMUSG00000056429	Tgoln1	-0.197230739	2.04E-18
ENSMUSG00000039356	Exosc2	-0.197043102	0.000573051
ENSMUSG00000044783	A730008H23	-0.196951076	0.006499982
ENSMUSG00000021972	Hmbox1	-0.196731672	0.048750063
ENSMUSG00000041625	Ggact	-0.195886126	2.96E-06
ENSMUSG00000030591	Psmd8	-0.195759362	0.014788109
ENSMUSG00000022559	Fbxl6	-0.195222031	0.008222951
ENSMUSG00000087687	Pet100	-0.195101813	2.29E-08
ENSMUSG00000043866	Taf10	-0.194997421	0.049383084
ENSMUSG00000026623	Lpgat1	-0.194892573	2.14E-20
ENSMUSG00000009647	Mcu	-0.194882578	0.004831708
ENSMUSG00000015889	Lta4h	-0.194794395	0.037050981

ENSMUSG00000005881	Ergic3	0.253467899	1.96E-05
ENSMUSG00000046139	Patl1	0.253003887	0.045071816
ENSMUSG00000032178	Ilf3	0.252732758	0.000345574
ENSMUSG00000050697	Prkaa1	0.252648662	0.001108449
ENSMUSG00000021280	Exoc3l4	0.252623039	7.99E-32
ENSMUSG00000028894	Inpp5b	0.25255843	3.21E-06
ENSMUSG00000026126	Ptpn18	0.252460086	6.38E-18
ENSMUSG00000024844	Banf1	0.252331622	1.13E-05
ENSMUSG00000021939	Ctsb	0.251572656	5.39E-24
ENSMUSG00000016496	Cd274	0.251519532	0.002402177
ENSMUSG00000038582	Pptc7	0.251425531	0.000637478
ENSMUSG00000052812	Atad2b	0.251340509	0.009122095
ENSMUSG00000025261	Huwe1	0.250976745	0.000251276
ENSMUSG00000004934	Pias4	0.249776849	0.044943342
ENSMUSG00000032596	Uba7	0.249425801	0.023476369
ENSMUSG00000021893	Capn7	0.249353063	0.000327057
ENSMUSG00000043940	Wdfy3	0.249044973	0.002093169
ENSMUSG00000007817	Zmiz1	0.248782803	8.98E-11
ENSMUSG00000039219	Arid4b	0.247862264	0.002214606
ENSMUSG00000090258	Churc1	0.247422175	0.035123721
ENSMUSG00000026222	Sp100	0.247242976	1.30E-17
ENSMUSG00000005268	Prlr	0.247213195	2.99E-05
ENSMUSG00000029050	Ski	0.247193314	5.07E-16
ENSMUSG00000025137	Pcyt2	0.247091938	6.88E-07
ENSMUSG00000028249	Sdcbp	0.246936418	0.042130774
ENSMUSG00000021520	Uqcrb	0.246920996	0.000127633
ENSMUSG00000064210	Ano6	0.246824838	7.45E-15
ENSMUSG00000025103	Btbd1	0.246015846	0.046388134
ENSMUSG00000023089	Ndufa5	0.245904944	1.43E-14
ENSMUSG00000058006	Mdn1	0.245762668	0.015238699
ENSMUSG00000039205	Ciz1	0.24543471	0.008543078
ENSMUSG00000006392	Med8	0.245330155	0.007058908
ENSMUSG00000052698	Tln2	0.244679775	1.53E-11
ENSMUSG00000006315	Tmem147	0.244649585	3.30E-06
ENSMUSG00000028649	Macf1	0.24454946	0.008083509
ENSMUSG00000057388	Mrpl18	0.244542326	1.75E-09
ENSMUSG00000107283	Mpv17	0.244313042	0.000238775
ENSMUSG00000003435	Supt5	0.244305525	0.038999003
ENSMUSG00000002804	Nudt14	0.244184688	6.47E-05
ENSMUSG00000048280	Zfp738	0.243986459	0.025789335
ENSMUSG00000058240	Cryzl1	0.243681631	3.34E-06
ENSMUSG00000031217	Efnb1	0.243352169	1.01E-05
ENSMUSG00000039304	Tnfsf10	0.243343747	7.99E-06
ENSMUSG00000025515	Muc2	0.243333714	1.60E-33
ENSMUSG00000019082	Slc25a22	0.24331982	6.08E-13
ENSMUSG00000027274	Mkks	0.243309077	9.17E-05
ENSMUSG00000054808	Actn4	0.243299717	0.000860537
ENSMUSG00000001794	Capns1	0.243116779	0.000262041
ENSMUSG00000044393	Dsg2	0.243012059	5.06E-18
ENSMUSG00000042293	Gm5617	0.242728781	0.006497768
ENSMUSG00000021868	Ppif	0.242589603	1.78E-06
ENSMUSG00000050552	Lamtor4	0.242567437	0.001174015
ENSMUSG00000006705	Pknox1	0.24240181	0.002271463
ENSMUSG00000043923	Cenatac	0.242228505	4.88E-05
ENSMUSG00000045838	Ccdc9b	0.241436429	0.03692031
ENSMUSG00000064109	Hcst	0.241417087	7.00E-10
ENSMUSG00000015605	Srf	0.241188924	1.03E-06
ENSMUSG00000032475	Nck1	0.240877327	0.002305727
ENSMUSG00000029467	Atp2a2	0.240764587	8.20E-13
ENSMUSG00000079559	Pou2af3	0.240730809	0.000332109
ENSMUSG00000072571	Tmem253	0.240449202	4.69E-12
ENSMUSG00000015023	Ddx19a	0.24037763	0.018174199
ENSMUSG00000020608	Smc6	0.240307553	5.38E-06
ENSMUSG00000018999	Slc35b4	0.240081507	0.001727824
ENSMUSG00000068747	Sort1	0.239860005	6.10E-18
ENSMUSG00000044221	Grsf1	0.239794258	0.041878331
ENSMUSG00000031931	Ankrd49	0.239601876	0.039244718
ENSMUSG00000020167	Tcf3	0.239512039	0.000126874
ENSMUSG00000004558	Ndrg2	0.239212622	2.60E-08
ENSMUSG00000023367	Tmem176a	0.238719213	0.018577836
ENSMUSG00000021690	Jmy	0.23863343	0.000711404
ENSMUSG00000017485	Top2b	0.238580601	0.011929613
ENSMUSG00000029752	Asns	0.238258805	2.40E-13
ENSMUSG00000051469	Zfp24	0.237396124	0.001502534
ENSMUSG00000032246	Calml4	0.237211168	5.54E-10
ENSMUSG00000021456	Fbp2	0.237020347	5.47E-09
ENSMUSG00000060121	Gemin2	0.237004	0.005317399
ENSMUSG00000025165	Sectm1a	0.236944459	2.24E-10
ENSMUSG00000001247	Lsr	0.23680748	3.67E-16
ENSMUSG00000024290	Rock1	0.236783543	2.25E-06
ENSMUSG00000020572	Nampt	0.236662441	2.68E-15
ENSMUSG00000063904	Dpp3	0.236601077	0.000156903
ENSMUSG00000027447	Cst3	0.236470985	2.32E-05
ENSMUSG00000006589	Aprt	0.236315704	1.40E-25
ENSMUSG00000024038	Ndufv3	0.236279502	7.44E-10
ENSMUSG00000020523	Fam114a2	0.236254255	1.75E-10
ENSMUSG00000070436	Serpinh1	0.236090707	0.002399427
ENSMUSG00000026701	Prdx6	0.235661897	9.46E-07
ENSMUSG00000024810	Il33	0.235651569	2.68E-12
ENSMUSG00000052214	Opa3	0.235605166	0.009846511
ENSMUSG00000028648	Ndufs5	0.235286896	4.56E-06
ENSMUSG00000002052	Supt6	0.234821202	0.005311537
ENSMUSG00000026353	Ubxn4	0.234721639	0.022591247
ENSMUSG00000031527	Eri1	0.234382256	0.001595691
ENSMUSG00000029405	G3bp2	0.234316011	0.00085329
ENSMUSG00000026860	Sh3glb2	0.234296289	0.037997814
ENSMUSG00000034701	Neurod1	0.234190522	0.00237907
ENSMUSG00000037098	Rab11fip3	0.23386517	0.024529872
ENSMUSG00000027076	Timm10	0.233862986	1.32E-10
ENSMUSG00000062963	Ufc1	0.233739106	0.009811712
ENSMUSG00000033326	Kdm4a	0.233429585	1.43E-05
ENSMUSG00000028367	Txn1	0.233291073	2.70E-15
ENSMUSG00000027018	Hat1	0.232935572	0.001988759
ENSMUSG00000046949	Nqo2	0.232608724	0.01634875
ENSMUSG00000024991	Eif3a	0.232493586	6.81E-11
ENSMUSG00000038034	Igsf8	0.232303265	0.003199626
ENSMUSG00000047730	Fcgbp	0.232197847	9.10E-10
ENSMUSG00000022358	Fbxo32	0.232137583	1.04E-24
ENSMUSG00000070738	Dgkd	0.232073187	1.17E-08
ENSMUSG00000055013	Agap1	0.231713705	7.16E-09
ENSMUSG00000019210	Atp6v1e1	0.231693937	3.19E-10
ENSMUSG00000021573	Tppp	0.231453583	7.03E-05
ENSMUSG00000028917	Plekhm2	0.231158446	0.048034937
ENSMUSG00000028465	Tln1	0.231100646	0.019964999
ENSMUSG00000029701	Rbm28	0.231051883	0.00391872
ENSMUSG00000062825	Actg1	0.230945817	0.047736318
ENSMUSG00000024604	Rbm22	0.230747598	0.006849382
ENSMUSG00000038332	Sesn1	0.230305154	0.03888568
ENSMUSG00000000682	Cd52	0.229766331	5.51E-05
ENSMUSG00000054662	Ano9	0.229751944	0.030150408
ENSMUSG00000061731	Ext1	0.229743413	3.55E-09
ENSMUSG00000028070	Naxe	0.22967054	0.00042001

ENSMUSG00000054428	Atpif1	-0.220901396	2.11E-10
ENSMUSG00000015217	Hmgb3	-0.220458216	0.016029763
ENSMUSG00000021460	Auh	-0.220432111	1.36E-06
ENSMUSG00000028675	Pnrc2	-0.220178581	0.002863014
ENSMUSG00000019866	Crybg1	-0.220171458	6.60E-26
ENSMUSG00000068184	Ndufaf2	-0.218747569	0.00489906
ENSMUSG00000063694	Cycs	-0.218732287	3.64E-09
ENSMUSG00000000159	Igsf5	-0.218566349	1.49E-05
ENSMUSG00000022400	Rbx1	-0.21833049	0.000657318
ENSMUSG00000024231	Cul2	-0.218114912	0.006493082
ENSMUSG00000000171	Sdhd	-0.217816256	1.41E-05
ENSMUSG00000022680	Pdxdc1	-0.217706023	0.004589407
ENSMUSG00000035232	Pdk3	-0.21604257	1.52E-06
ENSMUSG00000028024	Enpep	-0.216008389	1.52E-36
ENSMUSG00000021196	Pfkp	-0.215927593	0.006849382
ENSMUSG00000022329	Stk3	-0.21518377	9.03E-19
ENSMUSG00000006315	Tmem147	-0.215084561	3.30E-06
ENSMUSG00000020163	Uqcr11	-0.214822826	8.06E-14
ENSMUSG00000020580	Rock2	-0.214309636	0.00179514
ENSMUSG00000005804	Bloc1s6	-0.214131754	0.001546299
ENSMUSG00000031246	Sh3bgrl	-0.213953635	4.43E-14
ENSMUSG00000074829	2010315B03	-0.213757982	1.52E-08
ENSMUSG00000025237	Parp6	-0.213672861	0.013715055
ENSMUSG00000038671	Arfrp1	-0.213577943	4.25E-06
ENSMUSG00000043003	Rasef	-0.213529808	0.029334695
ENSMUSG00000021939	Ctsb	-0.213400675	5.39E-24
ENSMUSG00000014551	Mrps25	-0.212973732	0.001012552
ENSMUSG00000044080	S100a1	-0.212579078	8.34E-29
ENSMUSG00000000544	Gpa33	-0.212185662	0.013351547
ENSMUSG00000022698	Naa50	-0.212007908	0.016708234
ENSMUSG00000002949	Timm44	-0.211655467	4.58E-05
ENSMUSG00000038379	Ttk	-0.211459506	0.047780096
ENSMUSG00000070327	Rnf213	-0.211186986	0.001029581
ENSMUSG00000062753	Smim29	-0.211184485	0.013302226
ENSMUSG00000062619	2310039H08	-0.211060694	4.18E-05
ENSMUSG00000018239	Zcchc10	-0.21089287	0.000991987
ENSMUSG00000002043	Trappc6a	-0.210698226	0.000292422
ENSMUSG00000038240	Pdss2	-0.210431299	0.000457891
ENSMUSG00000048922	Cdca2	-0.210255209	0.006235582
ENSMUSG00000087260	Lamtor5	-0.210039843	0.008556454
ENSMUSG00000085421	4732490B19	-0.209784301	0.01707099
ENSMUSG00000017950	Hnf4a	-0.208898424	2.51E-11
ENSMUSG00000015672	Mrpl32	-0.208727671	1.29E-06
ENSMUSG00000025651	Uqcrc1	-0.208291773	4.38E-07
ENSMUSG00000055302	Mrfap1	-0.208122461	2.65E-13
ENSMUSG00000039943	Plcb4	-0.208032696	0.04824816
ENSMUSG00000031505	Naxd	-0.207771066	0.009364853
ENSMUSG00000021500	Ddx46	-0.207591187	2.26E-05
ENSMUSG00000031891	Hsd11b2	-0.207415111	1.85E-05
ENSMUSG00000024913	Lrp5	-0.207264267	0.042971842
ENSMUSG00000034006	Slc66a2	-0.207023851	0.03195251
ENSMUSG00000028102	Pex11b	-0.206955692	0.003419422
ENSMUSG00000037531	Mrpl47	-0.206769018	0.000972538
ENSMUSG00000003235	Eif2b5	-0.206352519	0.021976795
ENSMUSG00000021361	Tmem14c	-0.205859141	2.08E-11
ENSMUSG00000010601	Apol7a	-0.205622175	1.66E-13
ENSMUSG00000037361	Sf3b6	-0.205530884	0.015154516
ENSMUSG00000069208	Zfp825	-0.205505576	0.011382329
ENSMUSG00000030057	Cnbp	-0.205343687	0.000463455
ENSMUSG00000022677	Cep20	-0.205183056	0.000137983
ENSMUSG00000025498	Irf7	-0.205011609	1.79E-08
ENSMUSG00000026234	Ncl	-0.204847661	2.96E-12
ENSMUSG00000044600	Smim7	-0.204779026	4.43E-07
ENSMUSG00000021608	Lpcat1	-0.204623008	0.003153346
ENSMUSG00000026526	Fh1	-0.204312173	1.15E-12
ENSMUSG00000034574	Daam1	-0.204136703	7.77E-07
ENSMUSG00000026149	Tm4sf20	-0.204093916	1.07E-46
ENSMUSG00000117284	Gm7072	-0.204014723	0.013251376
ENSMUSG00000038525	Armc10	-0.203623759	0.035668392
ENSMUSG00000031950	Gabarapl2	-0.203444164	5.42E-07
ENSMUSG00000034211	Mrps17	-0.203203083	0.004349782
ENSMUSG00000026820	Ptges2	-0.203063768	6.11E-13
ENSMUSG00000037089	Slc35b2	-0.202934452	6.81E-07
ENSMUSG00000029821	Gsdme	-0.202742351	3.82E-96
ENSMUSG00000054364	Rhob	-0.202694487	4.71E-05
ENSMUSG00000050856	Atp5k	-0.202604767	2.01E-11
ENSMUSG00000075700	Selenot	-0.202558493	0.000509792
ENSMUSG00000041238	Rbbp8	-0.202427224	0.01990862
ENSMUSG00000063903	Klk1	-0.202425251	1.25E-25
ENSMUSG00000008540	Mgst1	-0.202150021	0.026480238
ENSMUSG00000022191	Drosha	-0.20175047	0.00141052
ENSMUSG00000060733	Ipmk	-0.201563105	4.17E-30
ENSMUSG00000050199	Lgr4	-0.201431729	2.95E-21
ENSMUSG00000059534	Uqcr10	-0.20115535	6.56E-14
ENSMUSG00000040850	Psme4	-0.201062201	0.022279962
ENSMUSG00000003531	Dgcr6	-0.200536976	6.88E-09
ENSMUSG00000034024	Cct2	-0.200501305	1.18E-06
ENSMUSG00000061286	Exosc5	-0.200486412	0.036793326
ENSMUSG00000019982	Myb	-0.200396563	1.62E-10
ENSMUSG00000023723	Mrps23	-0.199997178	3.64E-10
ENSMUSG00000027332	Ivd	-0.199670747	0.010550001
ENSMUSG00000074446	Defa23	-0.199605175	0.030492662
ENSMUSG00000031885	Cbfb	-0.199259028	0.00038681
ENSMUSG00000041040	Fam117b	-0.199223489	1.25E-16
ENSMUSG00000020232	Hmg20b	-0.198716659	1.97E-08
ENSMUSG00000060512	0610040J01F	-0.198376802	0.000997231
ENSMUSG00000046434	Hnrnpa1	-0.198009338	3.20E-05
ENSMUSG00000021112	Pals1	-0.197842464	0.009430858
ENSMUSG00000035958	Tdp2	-0.197733752	5.97E-13
ENSMUSG00000015932	Dstn	-0.197063877	8.50E-15
ENSMUSG00000036850	Mrpl41	-0.197028372	0.001365656
ENSMUSG00000025260	Hsd17b10	-0.196969332	0.001706765
ENSMUSG00000031644	Nek1	-0.196966836	0.002937568
ENSMUSG00000022111	Uchl3	-0.196700408	0.030070943
ENSMUSG00000040471	Ggt6	-0.196556445	1.69E-05
ENSMUSG00000024098	Twsg1	-0.19643444	0.003722434
ENSMUSG00000035726	Supt16	-0.196365739	4.41E-05
ENSMUSG00000015002	Efr3a	-0.196334042	2.33E-14
ENSMUSG00000032014	Oaf	-0.196220822	0.035217753
ENSMUSG00000025980	Hspd1	-0.196169957	0.009410388
ENSMUSG00000037110	Ralgapa2	-0.19606053	4.29E-05
ENSMUSG00000018381	Abi3	-0.196025135	5.68E-09
ENSMUSG00000030884	Uqcrc2	-0.195765536	2.59E-11
ENSMUSG00000031068	Glrx3	-0.195747027	0.048820618
ENSMUSG00000030528	Blm	-0.195571692	0.014592683
ENSMUSG00000033955	Tnks1bp1	-0.195493807	5.95E-05
ENSMUSG00000041733	Coq5	-0.194819851	0.007882978
ENSMUSG00000015837	Sqstm1	-0.194503129	7.52E-10
ENSMUSG00000020952	Scfd1	-0.194364665	0.000353135
ENSMUSG00000031812	Map1lc3b	-0.194238936	0.049500279
ENSMUSG00000049760	Micos13	-0.194149666	1.07E-09
ENSMUSG00000069020	Urm1	-0.194039989	0.000855807

ENSMUSG00000002052	Supt6	0.292798413	0.005311537
ENSMUSG00000078671	Chd2	0.292678639	0.002315008
ENSMUSG00000024491	Rbm27	0.292677579	0.000240937
ENSMUSG00000066043	Phactr4	0.291725168	0.023651903
ENSMUSG00000036986	Pml	0.291706134	0.000474505
ENSMUSG00000083380	Ndufb4c	0.29145782	2.10E-16
ENSMUSG00000048546	Tob2	0.291449129	0.006877541
ENSMUSG00000113204	Gm46430	0.291165059	0.026824258
ENSMUSG00000021156	Zmynd11	0.291036592	0.047844666
ENSMUSG00000021868	Ppif	0.291007446	1.78E-06
ENSMUSG00000031442	Mcf2l	0.289761062	9.81E-11
ENSMUSG00000021895	Arhgef3	0.289194374	4.71E-13
ENSMUSG00000079215	Zfp664	0.289114076	8.11E-05
ENSMUSG00000039220	Ppp1r10	0.289037605	0.001025322
ENSMUSG00000024002	Brd4	0.288878179	1.05E-09
ENSMUSG00000025006	Sorbs1	0.288333553	0.020694027
ENSMUSG00000040843	Tiprl	0.288156439	0.032391014
ENSMUSG00000040785	Ttc3	0.287787106	0.0149099
ENSMUSG00000002342	Tmem161a	0.287255578	0.020981507
ENSMUSG00000034765	Dusp5	0.287177206	4.29E-05
ENSMUSG00000061755	Bod1l	0.287045524	5.82E-07
ENSMUSG00000059495	Arhgef12	0.286990162	0.02018339
ENSMUSG00000066880	Zfp617	0.28679093	0.000423217
ENSMUSG00000029560	Snx8	0.286547849	0.003735951
ENSMUSG00000027955	Gask1b	0.286286494	1.61E-16
ENSMUSG00000035828	Pim3	0.285789838	0.000176905
ENSMUSG00000027452	Acss1	0.285364265	1.46E-13
ENSMUSG00000073725	Lmbrd1	0.285342055	0.016279203
ENSMUSG00000036775	Decr2	0.284739983	0.001576695
ENSMUSG00000020818	Mfsd11	0.2843752	0.046910347
ENSMUSG00000029381	Shroom3	0.283893612	1.46E-05
ENSMUSG00000040483	Xaf1	0.283729479	1.36E-20
ENSMUSG00000039515	Ptpa	0.283041299	0.004520609
ENSMUSG00000054630	Ugt2b5	0.282855117	2.79E-50
ENSMUSG00000022961	Son	0.282565863	4.07E-06
ENSMUSG00000006599	Gtf2h1	0.282218087	0.008170186
ENSMUSG00000063296	Tmem117	0.282185236	6.11E-82
ENSMUSG00000032602	Slc25a20	0.28212936	0.013172615
ENSMUSG00000028158	Mttp	0.282064656	3.05E-39
ENSMUSG00000018076	Med13l	0.281665623	5.81E-11
ENSMUSG00000029082	Bst1	0.281621202	5.52E-20
ENSMUSG00000031488	Rab11fip1	0.281201328	1.33E-08
ENSMUSG00000022892	App	0.281037554	5.19E-27
ENSMUSG00000033557	Fam20b	0.280799395	6.93E-05
ENSMUSG00000059447	Hadhb	0.280784892	6.00E-06
ENSMUSG00000083837	Gm14427	0.280494062	0.000994978
ENSMUSG00000022094	Slc39a14	0.280469124	3.48E-11
ENSMUSG00000058454	Dhcr7	0.280417809	0.001294384
ENSMUSG00000059775	Rps26-ps1	0.280335368	0.00013855
ENSMUSG00000020955	Ap4s1	0.280171131	0.040894926
ENSMUSG00000030718	Ppme1	0.280129539	0.000612051
ENSMUSG00000024070	Prkd3	0.279974711	0.00051001
ENSMUSG00000026749	Nek6	0.279747943	0.026185282
ENSMUSG00000036769	Wdr44	0.279668705	0.019720579
ENSMUSG00000041530	Ago1	0.27927066	0.001617074
ENSMUSG00000025477	Inpp5a	0.279203943	7.44E-10
ENSMUSG00000031924	Cyb5b	0.278924661	9.96E-33
ENSMUSG00000013997	Nit1	0.278870128	0.038076999
ENSMUSG00000039262	Prrc2b	0.278458191	0.002106378
ENSMUSG00000038145	Snrk	0.278442535	0.021948434
ENSMUSG00000047407	Tgif1	0.278414462	3.19E-07
ENSMUSG00000001211	Agpat3	0.277758413	1.57E-17
ENSMUSG00000028868	Wasf2	0.277520216	3.26E-09
ENSMUSG00000020640	Itsn2	0.277446427	0.002532728
ENSMUSG00000024566	Atp9b	0.277139817	0.005349933
ENSMUSG00000066571	Garre1	0.276692829	8.11E-05
ENSMUSG00000046157	Tmem229b	0.276554684	0.003932215
ENSMUSG00000017831	Rab5a	0.276328543	0.021538676
ENSMUSG00000042363	Lgalsl	0.276178663	4.47E-09
ENSMUSG00000026842	Abl1	0.275960929	0.000584911
ENSMUSG00000042625	Safb2	0.275705533	0.000423217
ENSMUSG00000035725	Prkx	0.275620451	0.014141363
ENSMUSG00000069833	Ahnak	0.275614305	6.47E-05
ENSMUSG00000026028	Trak2	0.275513627	0.016771673
ENSMUSG00000047030	Spata2	0.275432634	4.72E-05
ENSMUSG00000029335	Bmp3	0.275286306	1.81E-09
ENSMUSG00000021096	Ppm1a	0.275226648	0.001433497
ENSMUSG00000034584	Exph5	0.275132141	2.75E-12
ENSMUSG00000021213	Akr1c13	0.275112247	7.96E-53
ENSMUSG00000025404	R3hdm2	0.275019575	0.011041446
ENSMUSG00000028967	Errfi1	0.274857212	4.88E-05
ENSMUSG00000022636	Alcam	0.274844823	0.001539384
ENSMUSG00000026090	Cracdl	0.274484092	0.00046851
ENSMUSG00000026781	Acbd5	0.274069715	3.81E-19
ENSMUSG00000023262	Acy1	0.273417082	8.74E-07
ENSMUSG00000040712	Camta2	0.273360968	0.001232666
ENSMUSG00000032309	Fbxo22	0.273159948	0.000102949
ENSMUSG00000041773	Enc1	0.273099325	0.007088321
ENSMUSG00000032239	Rp9	0.27230153	0.049400636
ENSMUSG00000039852	Rere	0.27213628	6.61E-15
ENSMUSG00000024981	Acsl5	0.272033191	2.10E-32
ENSMUSG00000043822	Adamtsl5	0.271566834	0.023672623
ENSMUSG00000064138	Fam172a	0.271300937	2.32E-10
ENSMUSG00000027288	Zfp106	0.270997743	0.000146124
ENSMUSG00000028266	Lmo4	0.269708222	1.98E-06
ENSMUSG00000022095	Fhip2b	0.269696629	0.026192841
ENSMUSG00000034708	Grn	0.269683592	0.003388521
ENSMUSG00000005871	Apc	0.269169071	2.05E-05
ENSMUSG00000020409	Slu7	0.268907724	1.53E-15
ENSMUSG00000027519	Rab22a	0.268322373	0.021790683
ENSMUSG00000047423	AI837181	0.267510698	0.022880965
ENSMUSG00000038848	Ythdf1	0.266771261	0.000453027
ENSMUSG00000047613	A430005L14	0.266442341	5.59E-05
ENSMUSG00000002608	Ccdc97	0.265995221	0.0085051
ENSMUSG00000022710	Usp7	0.265711122	7.40E-06
ENSMUSG00000037960	Card19	0.265662296	0.020154342
ENSMUSG00000031838	Ifi30	0.265653471	1.76E-13
ENSMUSG00000015092	Edf1	0.265432672	1.96E-09
ENSMUSG00000022755	Adgrg7	0.265058769	7.44E-06
ENSMUSG00000066441	Rdh11	0.264635756	1.84E-17
ENSMUSG00000050965	Prkca	0.264228058	0.012516318
ENSMUSG00000026113	Inpp4a	0.263710749	0.039231689
ENSMUSG00000024248	Cox7a2l	0.263670687	7.83E-05
ENSMUSG00000057522	Spop	0.26312107	0.000110085
ENSMUSG00000018166	Erbb3	0.262992667	8.20E-13
ENSMUSG00000041241	Mul1	0.26272764	4.85E-13
ENSMUSG00000009646	Pla2g12b	0.262329324	0.000361624
ENSMUSG00000034471	Caskin2	0.262274911	1.53E-07
ENSMUSG00000040721	Zfhx2	0.262053407	0.011847783
ENSMUSG00000063870	Chd4	0.262033917	1.55E-05
ENSMUSG00000028780	Sema3c	0.261940814	4.13E-05
ENSMUSG00000050428	Fbxo46	0.261910685	4.22E-05

ENSMUSG00000031482	Slc25a15	-0.194730796	2.71E-06
ENSMUSG00000027828	Ssr3	-0.194721916	0.000623364
ENSMUSG00000002949	Timm44	-0.19465321	4.58E-05
ENSMUSG00000029447	Cct6a	-0.194481489	0.002059865
ENSMUSG00000022365	Derl1	-0.194180093	2.05E-10
ENSMUSG00000027078	Ube2l6	-0.194158653	5.37E-13
ENSMUSG00000033542	Arhgef5	-0.19407562	0.0067032
ENSMUSG00000026672	Optn	-0.194049937	0.00016076
ENSMUSG00000043831	Lysmd4	-0.19390211	0.02245163
ENSMUSG00000030029	Lrig1	-0.193821941	5.93E-06
ENSMUSG00000022090	Pdlim2	-0.193654943	0.022628139
ENSMUSG00000021884	Hacl1	-0.193499399	0.001978751
ENSMUSG00000047517	Dmbt1	-0.193491311	1.62E-10
ENSMUSG00000025366	Esyt1	-0.193460873	0.03367502
ENSMUSG00000030610	Det1	-0.193010348	0.001473462
ENSMUSG00000097779	4833407H14	-0.192360319	1.27E-07
ENSMUSG00000118038	Gm9895	-0.191693188	0.000477359
ENSMUSG00000030867	Plk1	-0.191489559	3.61E-09
ENSMUSG00000041605	Inava	-0.191480718	7.16E-20
ENSMUSG00000040188	Scamp2	-0.191418314	4.66E-11
ENSMUSG00000037573	Tob1	-0.19134591	4.50E-08
ENSMUSG00000029030	Tprgl	-0.191150146	0.03548192
ENSMUSG00000006050	Sra1	-0.191108746	1.26E-10
ENSMUSG00000004455	Ppp1cc	-0.190799717	0.014704092
ENSMUSG00000074748	Atxn7l3b	-0.190733868	7.95E-07
ENSMUSG00000001482	Def8	-0.190340886	0.003952614
ENSMUSG00000025410	Dctn2	-0.190031022	0.022569002
ENSMUSG00000045165	AI467606	-0.189697191	0.004120191
ENSMUSG00000024925	Rnaseh2c	-0.189340661	0.007549987
ENSMUSG00000022216	Psme1	-0.189133427	3.68E-11
ENSMUSG00000026039	Sgo2a	-0.189072961	0.010299182
ENSMUSG00000039737	Prkrip1	-0.18890231	0.000281226
ENSMUSG00000015536	Mocs2	-0.188879688	1.69E-24
ENSMUSG00000064036	Mro	-0.188255183	0.012370556
ENSMUSG00000043019	Edem3	-0.188151599	7.53E-09
ENSMUSG00000021024	Psma6	-0.187919248	0.000391629
ENSMUSG00000005779	Psmb4	-0.187904131	0.000123042
ENSMUSG00000109901	Chmp1b	-0.187853129	0.000103018
ENSMUSG00000036781	Rps27l	-0.187607996	0.000498526
ENSMUSG00000026417	Pigr	-0.187599113	2.07E-14
ENSMUSG00000052298	Cdc42se2	-0.187503525	0.028516241
ENSMUSG00000032567	Aste1	-0.186852934	0.029599358
ENSMUSG00000040520	Manea	-0.186849947	4.65E-05
ENSMUSG00000028693	Nasp	-0.186812293	0.000165444
ENSMUSG00000008461	Fut1	-0.186775192	0.010643215
ENSMUSG00000019982	Myb	-0.186392213	1.62E-10
ENSMUSG00000027309	Dnaaf9	-0.185769229	1.52E-16
ENSMUSG00000026922	Agpat2	-0.185613086	7.57E-05
ENSMUSG00000057367	Birc2	-0.185263666	0.001685219
ENSMUSG00000038528	Mfsd4b5	-0.184935624	0.01070431
ENSMUSG00000069020	Urm1	-0.184665196	0.000855807
ENSMUSG00000035441	Myo1d	-0.184661146	1.83E-20
ENSMUSG00000037033	Clca3b	-0.184176515	3.66E-60
ENSMUSG00000019863	Qrsl1	-0.184058493	0.003535704
ENSMUSG00000021947	Cryl1	-0.183962525	1.26E-16
ENSMUSG00000079418	Atg4a	-0.183833648	3.05E-05
ENSMUSG00000015405	Ace2	-0.183593722	0.001211575
ENSMUSG00000029149	Krtcap3	-0.183561734	2.91E-08
ENSMUSG00000043190	Rfesd	-0.183266195	0.00015023
ENSMUSG00000066306	Numa1	-0.182535343	2.78E-09
ENSMUSG00000024990	Rbp4	-0.182278007	0.029213504
ENSMUSG00000021072	Tmx1	-0.182111932	2.82E-06
ENSMUSG00000049858	Suox	-0.182093766	4.09E-51
ENSMUSG00000043311	D17H6S53E	-0.181528978	0.003957525
ENSMUSG00000004285	Atp6v1f	-0.181178302	0.000158318
ENSMUSG00000028003	Lrat	-0.181107091	1.52E-29
ENSMUSG00000004270	Lpcat3	-0.181045383	7.91E-12
ENSMUSG00000037742	Eef1a1	-0.181023077	7.59E-06
ENSMUSG00000096255	Dynlt1b	-0.180887894	0.000234715
ENSMUSG00000047139	Cd24a	-0.18071233	3.90E-09
ENSMUSG00000041375	Ccdc9	-0.18056177	0.014800023
ENSMUSG00000034765	Dusp5	-0.179886207	4.29E-05
ENSMUSG00000073639	Rab18	-0.17972003	0.004754158
ENSMUSG00000047617	Paxx	-0.179549617	1.61E-08
ENSMUSG00000020946	Gosr2	-0.179512291	1.60E-06
ENSMUSG00000070426	Rnf121	-0.17919401	3.89E-10
ENSMUSG00000059323	Tonsl	-0.179144661	0.004696182
ENSMUSG00000022668	Gtpbp8	-0.178893726	0.000119674
ENSMUSG00000024885	Aldh3b1	-0.178616204	0.002794355
ENSMUSG00000074261	Erich4	-0.178607324	1.72E-09
ENSMUSG00000035227	Spcs2	-0.178413127	1.30E-10
ENSMUSG00000033610	Pank1	-0.178085485	0.008244926
ENSMUSG00000034192	Lsm3	-0.177962966	3.25E-08
ENSMUSG00000045414	Dipk2a	-0.177436078	5.05E-08
ENSMUSG00000114003	Gm9616	-0.177424828	0.03757242
ENSMUSG00000022400	Rbx1	-0.177423516	0.000657318
ENSMUSG00000016308	Ube2a	-0.177293152	0.012960757
ENSMUSG00000037661	Gpr160	-0.177222202	0.007350991
ENSMUSG00000059923	Grb2	-0.176837455	0.045766349
ENSMUSG00000031485	Plpbp	-0.176710194	0.048561538
ENSMUSG00000027879	Sec22b	-0.176672088	1.14E-21
ENSMUSG00000090101	Snhg9	-0.175851724	1.08E-05
ENSMUSG00000055302	Mrfap1	-0.175638005	2.65E-13
ENSMUSG00000034889	Cactin	-0.1753701	0.000970805
ENSMUSG00000020091	Eif4ebp2	-0.175196296	3.08E-20
ENSMUSG00000060703	Cd302	-0.175101455	1.20E-14
ENSMUSG00000028755	Cda	-0.175045497	2.08E-24
ENSMUSG00000009646	Pla2g12b	-0.174603264	0.000361624
ENSMUSG00000063511	Snrnp70	-0.174165601	0.000127623
ENSMUSG00000022620	Arsa	-0.174154641	0.001997418
ENSMUSG00000038267	Slc22a23	-0.174055419	4.78E-10
ENSMUSG00000024197	Plin3	-0.174005136	2.66E-16
ENSMUSG00000029152	Ociad1	-0.173800816	7.01E-08
ENSMUSG00000022574	Naprt	-0.173472377	1.82E-07
ENSMUSG00000030865	Chp2	-0.173462061	7.62E-24
ENSMUSG00000037960	Card19	-0.173433658	0.020154342
ENSMUSG00000034616	Ssh3	-0.173426696	0.005730713
ENSMUSG00000025745	Hadha	-0.173268266	8.66E-05
ENSMUSG00000015305	Sash1	-0.172956424	0.000119653
ENSMUSG00000032288	Imp3	-0.172917173	0.000706544
ENSMUSG00000006095	Tbcb	-0.172869266	0.040265882
ENSMUSG00000098332	Pigbos1	-0.172858011	0.022526406
ENSMUSG00000028019	Pdgfc	-0.172849007	0.01248121
ENSMUSG00000043091	Tuba1c	-0.172121871	7.15E-09
ENSMUSG00000058267	Mrps14	-0.171772825	0.016040978
ENSMUSG00000032220	Myo1e	-0.171719278	9.11E-09
ENSMUSG00000042625	Safb2	-0.171389422	0.000423217
ENSMUSG00000059447	Hadhb	-0.17136041	6.00E-06
ENSMUSG00000019763	Rmnd1	-0.171095818	0.006937704
ENSMUSG00000032359	Ctsh	-0.170783798	5.50E-30
ENSMUSG00000075702	Selenom	-0.170780504	0.017645423
ENSMUSG00000020198	Ap3d1	-0.170636717	2.11E-07

ENSMUSG00000087006	Gm13889	0.229463786	0.028146604
ENSMUSG00000061111	Mcrip1	0.229034787	0.043204363
ENSMUSG00000033565	Rbfox2	0.228827422	0.002194184
ENSMUSG00000021619	Atg10	0.228615247	5.79E-05
ENSMUSG00000021650	Ptcd2	0.228312911	0.045106406
ENSMUSG00000076441	Ass1	0.228009869	0.027138662
ENSMUSG00000038084	Opa1	0.227880934	0.026925643
ENSMUSG00000001761	Smo	0.227784774	0.035189454
ENSMUSG00000064363	ND4	0.227748395	4.41E-08
ENSMUSG00000027496	Aurka	0.226987859	0.001659308
ENSMUSG00000053329	Gatd3a	0.226801593	0.0001302
ENSMUSG00000045983	Eif4g1	0.226480709	0.008852901
ENSMUSG00000024247	Pkdcc	0.226412182	0.024420475
ENSMUSG00000069833	Ahnak	0.226317873	6.47E-05
ENSMUSG00000033577	Myo6	0.226022999	4.94E-09
ENSMUSG00000029338	Antxr2	0.225917831	2.14E-10
ENSMUSG00000002768	Mea1	0.225519289	0.006145375
ENSMUSG00000025888	Casp1	0.22543904	2.15E-08
ENSMUSG00000003660	Snrnp200	0.225267338	0.001116991
ENSMUSG00000029381	Shroom3	0.225244121	1.46E-05
ENSMUSG00000034994	Eef2	0.225020124	1.56E-19
ENSMUSG00000035311	Gnptab	0.22498957	9.31E-08
ENSMUSG00000022329	Stk3	0.224807576	9.03E-19
ENSMUSG00000019689	Fmc1	0.224311716	3.45E-13
ENSMUSG00000036617	Etl4	0.224175595	2.94E-07
ENSMUSG00000021054	Sgpp1	0.224118945	0.001245323
ENSMUSG00000022037	Clu	0.224074486	0.00321906
ENSMUSG00000034875	Nudt19	0.223731763	0.000194646
ENSMUSG00000060224	Pyroxd2	0.223160801	0.030084456
ENSMUSG00000044080	S100a1	0.223043858	8.34E-29
ENSMUSG00000024963	Dnajc4	0.222396147	0.000550684
ENSMUSG00000039148	Sart1	0.22227877	0.015593789
ENSMUSG00000030629	Zfand6	0.221838095	1.58E-09
ENSMUSG00000038690	Atp5j2	0.221795867	5.93E-12
ENSMUSG00000046836	Brox	0.221547768	0.001925028
ENSMUSG00000018677	Slc25a39	0.221289615	0.004225507
ENSMUSG00000033590	Myo5c	0.220998526	1.57E-14
ENSMUSG00000038668	Lpar1	0.220946356	9.83E-09
ENSMUSG00000049960	Mrps16	0.220909191	3.31E-13
ENSMUSG00000020003	Pex7	0.220788564	0.004490301
ENSMUSG00000025509	Pnpla2	0.220714491	0.008168715
ENSMUSG00000032065	Tex12	0.220555898	0.000201348
ENSMUSG00000019775	Rgs17	0.220439287	0.000657803
ENSMUSG00000030428	Ttyh1	0.220109829	0.010275434
ENSMUSG00000075706	Gpx4	0.220026698	0.014690733
ENSMUSG00000036687	Tmem184a	0.219821509	0.031247801
ENSMUSG00000039087	Rreb1	0.219582226	0.000288229
ENSMUSG00000023913	Pla2g7	0.219208499	0.002620755
ENSMUSG00000028920	Fbxo42	0.219126595	0.007848567
ENSMUSG00000027422	Rrbp1	0.219008023	1.10E-06
ENSMUSG00000021782	Dlg5	0.218959296	1.21E-06
ENSMUSG00000031386	Hcfc1	0.218808189	0.000425898
ENSMUSG00000007338	Mrpl49	0.218715836	2.28E-05
ENSMUSG00000034157	Cipc	0.21850572	0.000182056
ENSMUSG00000004565	Pnpla6	0.218451634	0.023958135
ENSMUSG00000037791	Phf12	0.218383881	0.000278762
ENSMUSG00000120252	Gm31504	0.218003323	0.000744549
ENSMUSG00000117621	Hspe1-rs1	0.217925578	0.000889768
ENSMUSG00000028447	Dctn3	0.217797334	0.001345616
ENSMUSG00000002325	Irf9	0.217411151	8.62E-10
ENSMUSG00000025178	Pi4k2a	0.21731798	0.000492092
ENSMUSG00000052040	Klf13	0.217295709	1.25E-09
ENSMUSG00000024219	Anks1	0.217284853	0.022047487
ENSMUSG00000005225	Plekha8	0.216766318	5.23E-05
ENSMUSG00000009681	Bcr	0.216718242	0.00167826
ENSMUSG00000005534	Insr	0.216639011	0.000127622
ENSMUSG00000041530	Ago1	0.216399431	0.001617074
ENSMUSG00000039089	L3mbtl3	0.216300245	0.040776418
ENSMUSG00000026565	Pou2f1	0.216100643	3.35E-06
ENSMUSG00000071659	Hnrnpul2	0.215742721	0.000164264
ENSMUSG00000026149	Tm4sf20	0.215609791	1.07E-46
ENSMUSG00000030265	Kras	0.215567514	4.44E-09
ENSMUSG00000020778	Ten1	0.215365233	0.008325527
ENSMUSG00000044254	Pcsk9	0.214893275	5.23E-28
ENSMUSG00000024732	Ccdc86	0.214837791	0.010158039
ENSMUSG00000030786	Itgam	0.214748956	0.001599213
ENSMUSG00000030643	Rab30	0.213842979	3.50E-11
ENSMUSG00000006390	Elovl1	0.213828923	4.14E-08
ENSMUSG00000021033	Gstz1	0.213045469	2.52E-09
ENSMUSG00000021636	Marveld2	0.212921981	0.000651315
ENSMUSG00000026473	Glul	0.212389473	2.04E-05
ENSMUSG00000017291	Taok1	0.212037164	9.43E-07
ENSMUSG00000020315	Sptbn1	0.21190088	5.80E-14
ENSMUSG00000024491	Rbm27	0.211853053	0.000240937
ENSMUSG00000041571	Selenow	0.211750388	1.28E-07
ENSMUSG00000041773	Enc1	0.211707115	0.007088321
ENSMUSG00000034667	Xpot	0.211609072	0.003819374
ENSMUSG00000041164	Zmiz2	0.211540421	1.01E-05
ENSMUSG00000029066	Mrpl20	0.211334193	2.01E-08
ENSMUSG00000026510	Trp53bp2	0.211310917	0.04525241
ENSMUSG00000030452	Nipa2	0.211251962	1.37E-06
ENSMUSG00000048707	Tprn	0.210792312	4.65E-06
ENSMUSG00000034480	Diaph2	0.210447273	0.003440188
ENSMUSG00000018412	Kansl1	0.210432056	0.011096963
ENSMUSG00000025260	Hsd17b10	0.210090697	0.001706765
ENSMUSG00000024002	Brd4	0.210004169	1.05E-09
ENSMUSG00000062981	Mrpl42	0.209845813	4.90E-05
ENSMUSG00000034801	Sos2	0.209773572	0.009038371
ENSMUSG00000080893	Ndufa12-ps	0.209420875	6.81E-12
ENSMUSG00000020220	Vps13d	0.209220929	1.47E-05
ENSMUSG00000022419	Deptor	0.209191315	1.10E-11
ENSMUSG00000025612	Bach1	0.209147095	0.034751082
ENSMUSG00000054889	Dsp	0.209073084	4.59E-11
ENSMUSG00000056758	Hmga2	0.208638872	0.013520105
ENSMUSG00000079941	Cox5b-ps	0.208206304	3.94E-19
ENSMUSG00000013523	Bcas1	0.207664894	1.18E-19
ENSMUSG00000050931	Sgms2	0.207069316	0.017488323
ENSMUSG00000028771	Ptpn12	0.206928775	0.001049906
ENSMUSG00000029560	Snx8	0.206826646	0.003735951
ENSMUSG00000048118	Arid4a	0.206809978	2.32E-09
ENSMUSG00000010601	Apol7a	0.206806685	1.66E-13
ENSMUSG00000022217	Emc9	0.206480168	9.88E-07
ENSMUSG00000046876	Atxn1	0.206221673	6.53E-11
ENSMUSG00000027534	Snx16	0.20616878	0.001586423
ENSMUSG00000032411	Tfdp2	0.206125931	0.036600532
ENSMUSG00000042428	Mgat3	0.205890889	0.000410973
ENSMUSG00000027673	Ndufb5	0.205885442	3.43E-09
ENSMUSG00000022533	Atp13a3	0.205850987	1.01E-16
ENSMUSG00000048701	Ccdc6	0.2058502	0.000270554
ENSMUSG00000026753	Ppp6c	0.20579076	0.036167935
ENSMUSG00000054381	Zfp747	0.205555314	0.003482977
ENSMUSG00000037904	Ankrd9	0.205517069	0.027573307

ENSMUSG00000033318	Gstt2	-0.193807956	3.78E-09
ENSMUSG00000018417	Myo1b	-0.193509105	0.000134267
ENSMUSG00000025428	Atp5a1	-0.193224228	1.80E-33
ENSMUSG00000025917	Cops5	-0.193072565	0.000612087
ENSMUSG00000052459	Atp6v1a	-0.192981771	8.19E-05
ENSMUSG00000031154	Otud5	-0.192761148	0.027736522
ENSMUSG00000022551	Cyc1	-0.192529467	2.09E-16
ENSMUSG00000079941	Cox5b-ps	-0.192246264	3.94E-19
ENSMUSG00000010057	Nprl2	-0.191978415	0.00035654
ENSMUSG00000018858	Mrpl58	-0.191819795	3.01E-09
ENSMUSG00000034459	Ifit1	-0.191790481	7.04E-10
ENSMUSG00000023944	Hsp90ab1	-0.191656668	0.000277912
ENSMUSG00000042369	Rbm45	-0.191535852	0.000636341
ENSMUSG00000038195	Rilp	-0.191506028	3.61E-05
ENSMUSG00000002280	Ciao3	-0.191441969	0.024637667
ENSMUSG00000041438	Utp4	-0.191328526	0.037281104
ENSMUSG00000021477	Ctsl	-0.191293717	1.12E-32
ENSMUSG00000049960	Mrps16	-0.191263154	3.31E-13
ENSMUSG00000030225	Dera	-0.190957463	1.50E-06
ENSMUSG00000027219	Slc28a2	-0.190313729	1.02E-17
ENSMUSG00000002635	Pdcd2l	-0.190265021	0.001774511
ENSMUSG00000041143	Tmco4	-0.190233636	3.70E-06
ENSMUSG00000030879	Mrpl17	-0.190148993	1.06E-09
ENSMUSG00000024899	Papss2	-0.189711812	8.85E-35
ENSMUSG00000055723	Rras2	-0.189360706	0.013025101
ENSMUSG00000003051	Elf3	-0.189159639	0.013686283
ENSMUSG00000079559	Pou2af3	-0.188925881	0.000332109
ENSMUSG00000024048	Myl12a	-0.188850362	0.025812037
ENSMUSG00000027111	Itga6	-0.188565021	0.007524523
ENSMUSG00000055839	Elob	-0.187934548	0.019982045
ENSMUSG00000003352	Cacnb3	-0.187654486	3.59E-05
ENSMUSG00000030704	Rab6a	-0.18689856	7.72E-13
ENSMUSG00000021062	Rab15	-0.186897151	1.58E-10
ENSMUSG00000019852	Arfgef3	-0.186879952	3.97E-23
ENSMUSG00000028436	Dcaf12	-0.186874798	0.016255009
ENSMUSG00000030663	1110004F10I	-0.186810214	0.006796763
ENSMUSG00000025544	Tm9sf2	-0.186745826	0.000162157
ENSMUSG00000048707	Tprn	-0.186640821	4.65E-06
ENSMUSG00000066839	Ecsit	-0.186376846	4.68E-08
ENSMUSG00000026510	Trp53bp2	-0.186244578	0.04525241
ENSMUSG00000027342	Pcna	-0.185525163	0.000916739
ENSMUSG00000037287	Tbcel	-0.185343428	0.000697929
ENSMUSG00000036299	BC031181	-0.185196466	3.55E-07
ENSMUSG00000056941	Commd7	-0.185012187	0.006017453
ENSMUSG00000032567	Aste1	-0.184771044	0.029599358
ENSMUSG00000042216	Sgsm1	-0.184761347	0.021540694
ENSMUSG00000032413	Rasa2	-0.184692619	0.011202067
ENSMUSG00000029561	Oasl2	-0.184680699	1.51E-06
ENSMUSG00000026097	Ormdl1	-0.184539944	3.82E-05
ENSMUSG00000014856	Tmem208	-0.184297125	3.45E-07
ENSMUSG00000043153	Crppa	-0.184093042	0.006959974
ENSMUSG00000047638	Nr1h4	-0.184044678	0.005000842
ENSMUSG00000038683	Pak1ip1	-0.184041671	4.49E-06
ENSMUSG00000059278	Naa38	-0.183804516	0.00039526
ENSMUSG00000028894	Inpp5b	-0.183645415	3.21E-06
ENSMUSG00000034566	Atp5h	-0.183635328	1.36E-11
ENSMUSG00000021379	Id4	-0.18328432	0.002088176
ENSMUSG00000020076	Ddx50	-0.182891108	0.002355971
ENSMUSG00000057110	Cntrl	-0.182344303	0.000595133
ENSMUSG00000062075	Lmnb2	-0.181953779	0.003677289
ENSMUSG00000015357	Clpx	-0.181433277	0.00544832
ENSMUSG00000027469	Tpx2	-0.18119378	2.29E-07
ENSMUSG00000025525	Apool	-0.181142569	8.05E-06
ENSMUSG00000016534	Lamp2	-0.180840403	3.18E-06
ENSMUSG00000021036	Sptlc2	-0.180505126	0.010630951
ENSMUSG00000020267	Hint1	-0.180408179	1.08E-09
ENSMUSG00000022477	Aco2	-0.180323166	5.10E-11
ENSMUSG00000062352	Itgb1bp1	-0.180137845	0.00173483
ENSMUSG00000039914	Coq10a	-0.180042024	0.018226652
ENSMUSG00000025781	Atp5c1	-0.179816902	6.29E-11
ENSMUSG00000037373	Ctbp1	-0.179479925	3.50E-05
ENSMUSG00000022820	Ndufb4	-0.179360617	1.71E-10
ENSMUSG00000028461	Ccdc107	-0.179078667	1.42E-12
ENSMUSG00000024145	Pigf	-0.178862851	0.010522271
ENSMUSG00000031878	Nae1	-0.178842333	2.64E-07
ENSMUSG00000035451	Foxa1	-0.178366242	1.28E-13
ENSMUSG00000062169	Cnih4	-0.178342233	8.41E-09
ENSMUSG00000041215	Yeats2	-0.178246664	0.022893675
ENSMUSG00000061518	Cox5b	-0.178072762	8.43E-15
ENSMUSG00000074261	Erich4	-0.177977616	1.72E-09
ENSMUSG00000022673	Mcm4	-0.17771125	0.003174862
ENSMUSG00000075701	Selenos	-0.177161532	0.00031883
ENSMUSG00000055976	Cldn23	-0.177054963	0.000421334
ENSMUSG00000033938	Ndufb7	-0.176993096	4.63E-14
ENSMUSG00000026154	Sdhaf4	-0.176984049	6.99E-07
ENSMUSG00000032299	Commd4	-0.176789203	0.026572224
ENSMUSG00000021051	Ppp2r5e	-0.176662961	0.013515925
ENSMUSG00000028063	Lmna	-0.176646742	1.46E-12
ENSMUSG00000000001	Gnai3	-0.176459325	0.007755676
ENSMUSG00000027968	Larp7	-0.176274363	8.43E-05
ENSMUSG00000069516	Lyz2	-0.175722585	0.015095697
ENSMUSG00000043501	Lgals2	-0.175225242	0.035681706
ENSMUSG00000050627	Gpd1l	-0.175211879	1.92E-09
ENSMUSG00000036873	2410004B18	-0.175114146	0.020113341
ENSMUSG00000052214	Opa3	-0.174870905	0.009846511
ENSMUSG00000026605	Cenpf	-0.174647858	2.84E-07
ENSMUSG00000024150	Mcfd2	-0.17447581	1.88E-09
ENSMUSG00000004815	Dgkq	-0.174425727	1.37E-06
ENSMUSG00000038717	Atp5l	-0.173827281	1.88E-09
ENSMUSG00000030335	Mrpl51	-0.173775813	0.001085329
ENSMUSG00000005103	Wdr1	-0.173561449	5.48E-20
ENSMUSG00000022554	Hgh1	-0.173406442	0.017104211
ENSMUSG00000046516	Cox17	-0.173216455	9.27E-07
ENSMUSG00000020782	Llgl2	-0.173122143	0.000617831
ENSMUSG00000018379	Srsf1	-0.172319426	7.87E-08
ENSMUSG00000021484	Lman2	-0.172258331	0.005513403
ENSMUSG00000007815	Rhoa	-0.172144174	0.007261257
ENSMUSG00000022124	Fbxl3	-0.172075941	0.000923024
ENSMUSG00000037788	Vopp1	-0.17203011	2.22E-23
ENSMUSG00000039013	Siglecf	-0.171935137	9.07E-06
ENSMUSG00000041203	Trir	-0.171612629	0.025844537
ENSMUSG00000049550	Clip1	-0.171174037	4.45E-05
ENSMUSG00000038822	Hace1	-0.171057183	0.022671839
ENSMUSG00000007338	Mrpl49	-0.170992499	2.28E-05
ENSMUSG00000019966	Kitl	-0.170992172	1.32E-07
ENSMUSG00000025421	Hdhd2	-0.170790563	9.66E-06
ENSMUSG00000032915	Adgre4	-0.170777993	0.016489501
ENSMUSG00000021139	Gm20498	-0.170777491	0.048865101
ENSMUSG00000079055	Slc8a3	-0.17077685	0.018081601
ENSMUSG00000037762	Slc16a9	-0.170767735	0.001527026
ENSMUSG00000075232	Amd1	-0.170707571	0.002123934
ENSMUSG00000026355	Mcm6	-0.170597985	0.002640015

ENSMUSG00000009907	Vps4b	0.261789221	0.00241387
ENSMUSG00000052040	Klf13	0.261786712	1.25E-09
ENSMUSG00000039148	Sart1	0.261775971	0.015593789
ENSMUSG00000042377	Fam83g	0.261392141	1.29E-06
ENSMUSG00000032525	Nktr	0.261388227	0.01401643
ENSMUSG00000021474	Sfxn1	0.261192573	9.27E-08
ENSMUSG00000067942	Zfp160	0.260582405	0.019310557
ENSMUSG00000061536	Sec22c	0.260302561	1.71E-09
ENSMUSG00000032349	Elovl5	0.260271596	7.01E-23
ENSMUSG00000038371	Sbf2	0.260185223	0.042758846
ENSMUSG00000030534	Vps33b	0.260108745	0.001944919
ENSMUSG00000004748	Mtfp1	0.259810656	1.05E-15
ENSMUSG00000078439	Smim24	0.259630751	9.16E-82
ENSMUSG00000034472	Rasd2	0.259556617	3.65E-07
ENSMUSG00000020229	Slc5a4a	0.259509945	2.13E-08
ENSMUSG00000020074	Ccar1	0.259469631	4.54E-08
ENSMUSG00000035642	Aamdc	0.259430559	4.28E-15
ENSMUSG00000059316	Slc27a4	0.259404318	0.004555358
ENSMUSG00000033713	Foxn3	0.259347385	6.11E-13
ENSMUSG00000046897	Zfp740	0.25916945	0.041346824
ENSMUSG00000028145	Them4	0.258613624	2.27E-11
ENSMUSG00000038059	Smim3	0.258441781	0.019239983
ENSMUSG00000034707	Gns	0.258419154	0.000919104
ENSMUSG00000022433	Csnk1e	0.258241586	0.042765575
ENSMUSG00000031592	Pcm1	0.257148976	0.029111456
ENSMUSG00000023829	Slc22a1	0.257046196	1.25E-15
ENSMUSG00000015405	Ace2	0.256636649	0.001211575
ENSMUSG00000026398	Nr5a2	0.255947461	3.09E-20
ENSMUSG00000021772	Nkiras1	0.255836862	1.04E-14
ENSMUSG00000029851	Tcaf2	0.255775563	0.003449118
ENSMUSG00000027035	Cers6	0.255313238	0.000509931
ENSMUSG00000015305	Sash1	0.255177559	0.000119653
ENSMUSG00000031839	Hsbp1	0.2549632	0.000367421
ENSMUSG00000031714	Gab1	0.254937282	0.001807062
ENSMUSG00000001151	Pcnt	0.254360979	7.28E-10
ENSMUSG00000037106	Fer1l6	0.254195151	9.54E-10
ENSMUSG00000039835	Nhsl1	0.254098402	6.04E-05
ENSMUSG00000033392	Clasp2	0.253955758	0.001063144
ENSMUSG00000029860	Zyx	0.253813732	0.004059296
ENSMUSG00000025165	Sectm1a	0.253661125	2.24E-10
ENSMUSG00000032741	Tpcn1	0.253651255	0.000253703
ENSMUSG00000029439	Sfswap	0.253346163	0.000112419
ENSMUSG00000037031	Tspan15	0.253257867	0.021907569
ENSMUSG00000030096	Slc6a6	0.252991503	3.31E-11
ENSMUSG00000025401	Myo1a	0.25266692	1.32E-07
ENSMUSG00000038909	Kat7	0.252484711	0.021533336
ENSMUSG00000033047	Eif3l	0.252429923	0.022767552
ENSMUSG00000039633	Lonrf1	0.252358358	0.00415548
ENSMUSG00000020532	Acaca	0.252014063	1.71E-05
ENSMUSG00000035992	Fnip1	0.251777487	0.000109679
ENSMUSG00000078317	F8a	0.251772007	0.010305582
ENSMUSG00000000567	Sox9	0.251750584	1.26E-12
ENSMUSG00000072946	Ptgr2	0.251147177	3.59E-12
ENSMUSG00000020864	Ankrd40	0.250916197	0.000826805
ENSMUSG00000020029	Nudt4	0.250426714	5.09E-36
ENSMUSG00000019362	D8Ertd738e	0.250261296	0.000800747
ENSMUSG00000024091	Vapa	0.249838928	5.53E-10
ENSMUSG00000022973	Synj1	0.24974943	0.005459893
ENSMUSG00000056665	Them6	0.249491912	0.032125241
ENSMUSG00000036120	Rfxank	0.249371274	0.034538216
ENSMUSG00000029189	Sel1l3	0.249203231	6.42E-14
ENSMUSG00000042293	Gm5617	0.248958631	0.006497768
ENSMUSG00000036499	Eea1	0.248245647	7.22E-10
ENSMUSG00000032652	Crebl2	0.248194794	0.010485435
ENSMUSG00000076431	Sox4	0.248056776	3.39E-15
ENSMUSG00000025280	Polr3a	0.247999738	0.000337974
ENSMUSG00000039157	Eeig1	0.247888854	0.004998145
ENSMUSG00000000275	Trim25	0.247750029	0.001877963
ENSMUSG00000039473	Ubn1	0.247535849	4.89E-08
ENSMUSG00000033542	Arhgef5	0.247410947	0.0067032
ENSMUSG00000011752	Pgam1	0.246625608	0.000128523
ENSMUSG00000032178	Ilf3	0.246595181	0.000345574
ENSMUSG00000061859	Patj	0.246566336	1.11E-05
ENSMUSG00000048277	Syngr2	0.246511279	1.24E-07
ENSMUSG00000032411	Tfdp2	0.246119776	0.036600532
ENSMUSG00000037887	Dusp8	0.246118229	0.041924022
ENSMUSG00000024404	Riok3	0.245632123	0.001218359
ENSMUSG00000110841	Gpx4-ps2	0.245609316	2.81E-48
ENSMUSG00000031862	Atp13a1	0.245600175	0.025108957
ENSMUSG00000037646	Vps13b	0.245593708	8.28E-07
ENSMUSG00000038828	Tmem214	0.245464769	0.001099444
ENSMUSG00000045316	Fahd1	0.245262724	1.74E-24
ENSMUSG00000030516	Tjp1	0.244956951	0.000102727
ENSMUSG00000021708	Rasgrf2	0.244907482	0.016803978
ENSMUSG00000068299	Nat8f4	0.244851734	0.000486813
ENSMUSG00000040365	Trim41	0.244641149	0.000332109
ENSMUSG00000058230	Arhgap35	0.24455333	6.39E-05
ENSMUSG00000047731	Wbp1l	0.244407843	0.003621695
ENSMUSG00000000826	Dnajc5	0.243922538	0.003608153
ENSMUSG00000040459	Arglu1	0.243651868	9.87E-06
ENSMUSG00000025350	Rdh5	0.242755624	0.001820682
ENSMUSG00000060538	Tmem219	0.242753327	1.36E-06
ENSMUSG00000032827	Ppp1r9a	0.242456299	0.000412672
ENSMUSG00000029131	Dnajb6	0.242234402	0.007864052
ENSMUSG00000035621	Midn	0.242231013	1.06E-11
ENSMUSG00000005980	Dnase1	0.241732776	3.55E-08
ENSMUSG00000028101	Pias3	0.241328314	0.007362687
ENSMUSG00000062014	Gmfb	0.241322102	0.000147745
ENSMUSG00000024619	Cdx1	0.241218539	1.94E-26
ENSMUSG00000021589	Rhobtb3	0.241008913	2.30E-30
ENSMUSG00000024292	Cyp4f14	0.240884461	0.001024626
ENSMUSG00000033326	Kdm4a	0.240267536	1.43E-05
ENSMUSG00000040225	Prrc2c	0.24014822	3.93E-07
ENSMUSG00000022286	Grhl2	0.239938798	0.031809287
ENSMUSG00000015656	Hspa8	0.239678383	0.000460382
ENSMUSG00000028080	Lrba	0.239675173	0.019730174
ENSMUSG00000003810	Mast2	0.239522714	2.01E-07
ENSMUSG00000048007	Timm8a1	0.239497099	0.001773946
ENSMUSG00000033075	Senp1	0.239376934	2.92E-05
ENSMUSG00000012535	Tnpo3	0.239028656	0.007699726
ENSMUSG00000039304	Tnfsf10	0.239015403	7.99E-06
ENSMUSG00000044709	Gemin7	0.238506301	0.004459285
ENSMUSG00000035473	Galm	0.238319732	1.13E-38
ENSMUSG00000005672	Kit	0.238124681	5.71E-09
ENSMUSG00000017760	Ctsa	0.238046361	0.032549775
ENSMUSG00000045098	Kmt5b	0.237342462	0.003462674
ENSMUSG00000025786	Zdhhc3	0.237285632	0.000922699
ENSMUSG00000036975	Tmem177	0.237182862	0.008362682
ENSMUSG00000051339	2900026A02	0.237091086	1.75E-12
ENSMUSG00000071369	Map3k5	0.236844674	2.42E-05
ENSMUSG00000065979	Cpped1	0.236702426	3.43E-12
ENSMUSG00000050552	Lamtor4	0.23630524	0.001174015

ENSMUSG00000104368	2010103J01F	-0.170563175	4.00E-14
ENSMUSG00000020267	Hint1	-0.170510217	1.08E-09
ENSMUSG00000028059	Arhgef2	-0.170494909	0.00358307
ENSMUSG00000014932	Yes1	-0.170406735	8.90E-05
ENSMUSG00000050891	Tatdn1	-0.170341907	0.049500279
ENSMUSG00000025732	Mcrip2	-0.169823827	3.89E-10
ENSMUSG00000019806	Aig1	-0.1697871	0.000658285
ENSMUSG00000041220	Elovl6	-0.169746966	0.00055278
ENSMUSG00000005354	Txn2	-0.169695052	1.66E-08
ENSMUSG00000097354	2310001H17	-0.169238034	0.025986568
ENSMUSG00000025528	2010106E10I	-0.169173227	6.92E-09
ENSMUSG00000016534	Lamp2	-0.168988117	3.18E-06
ENSMUSG00000020219	Timm13	-0.168984842	6.20E-05
ENSMUSG00000020877	Scrn2	-0.168804827	0.008654496
ENSMUSG00000020133	2310011J03F	-0.168375946	0.000192737
ENSMUSG00000078681	Tm2d3	-0.168082959	0.000509931
ENSMUSG00000021823	Vcl	-0.168011047	2.74E-05
ENSMUSG00000058715	Fcer1g	-0.168001397	0.000194731
ENSMUSG00000025545	Clybl	-0.167416299	1.66E-06
ENSMUSG00000051234	Rnf7	-0.167294295	2.33E-06
ENSMUSG00000026321	Tnfrsf11a	-0.167216702	9.01E-11
ENSMUSG00000121513	Ptprg	-0.167144644	0.01070431
ENSMUSG00000019578	Ubxn6	-0.167101766	0.002340762
ENSMUSG00000054717	Hmgb2	-0.166867277	0.002053144
ENSMUSG00000019761	Krt10	-0.166843885	0.029801642
ENSMUSG00000059326	Csf2ra	-0.166734793	1.79E-14
ENSMUSG00000031934	Panx1	-0.166211598	2.35E-11
ENSMUSG00000075010	AW112010	-0.166102563	3.88E-07
ENSMUSG00000004535	Tax1bp1	-0.166058729	0.001297722
ENSMUSG00000031516	Dctn6	-0.165143696	0.014948642
ENSMUSG00000035357	Pdzrn3	-0.165130553	0.004052349
ENSMUSG00000038943	Prc1	-0.164797503	8.38E-10
ENSMUSG00000001225	Slc26a3	-0.164640333	2.38E-07
ENSMUSG00000071655	Ubxn1	-0.164555551	0.033221993
ENSMUSG00000027067	Ssrp1	-0.164502422	1.17E-08
ENSMUSG00000023044	Csad	-0.164319816	0.003589867
ENSMUSG00000034282	Evpl	-0.164209989	8.52E-19
ENSMUSG00000045775	Slc16a5	-0.163885647	0.007886387
ENSMUSG00000024959	Bad	-0.163725638	3.71E-10
ENSMUSG00000070923	Klhl9	-0.163031198	7.14E-09
ENSMUSG00000040943	Tet2	-0.163027983	0.00032707
ENSMUSG00000031609	Sap30	-0.162961017	0.027859551
ENSMUSG00000030491	Tdrd12	-0.162793742	0.017139466
ENSMUSG00000026849	Tor1a	-0.162693596	1.09E-11
ENSMUSG00000039701	Usp53	-0.162542394	0.005257668
ENSMUSG00000063903	Klk1	-0.162523771	1.25E-25
ENSMUSG00000023452	Pisd	-0.162072206	6.78E-07
ENSMUSG00000013822	Elof1	-0.162036866	1.65E-05
ENSMUSG00000022191	Drosha	-0.161795318	0.00141052
ENSMUSG00000003200	Sh3gl1	-0.161550525	0.009214125
ENSMUSG00000053317	Sec61b	-0.161519897	0.036229616
ENSMUSG00000015143	Actn1	-0.161463707	7.61E-08
ENSMUSG00000033478	Fhip2a	-0.161459517	0.005975048
ENSMUSG00000030890	Ilk	-0.161329263	0.000668681
ENSMUSG00000026281	Dtymk	-0.161255694	0.03598728
ENSMUSG00000020142	Slc1a4	-0.161037371	2.10E-05
ENSMUSG00000057110	Cntrl	-0.160998827	0.000595133
ENSMUSG00000042308	Setd1a	-0.160767967	0.0410229
ENSMUSG00000000326	Comt	-0.160585082	0.013773667
ENSMUSG00000024105	Themis3	-0.160560103	7.62E-05
ENSMUSG00000039063	Echdc3	-0.160024439	0.009601643
ENSMUSG00000034659	Tmem109	-0.159841697	0.001937543
ENSMUSG00000038342	Mlxip	-0.159757313	0.036211525
ENSMUSG00000031410	Nxf7	-0.159276832	0.006118009
ENSMUSG00000118376	Gm9926	-0.159053161	0.00287052
ENSMUSG00000015671	Psma2	-0.158992691	0.01229255
ENSMUSG00000032563	Mrpl3	-0.1589468	0.000260491
ENSMUSG00000076609	Igkc	-0.158420963	1.95E-09
ENSMUSG00000030492	Slc7a9	-0.158289568	0.000761763
ENSMUSG00000021840	Mapk1ip1l	-0.157668184	3.88E-13
ENSMUSG00000025198	Erlin1	-0.157421669	5.90E-09
ENSMUSG00000055850	Rnf181	-0.157319665	0.025456237
ENSMUSG00000002068	Ccne1	-0.157213231	0.019377542
ENSMUSG00000025162	Csnk1d	-0.157016729	8.31E-05
ENSMUSG00000006304	Arpc2	-0.156685126	0.002529459
ENSMUSG00000030793	Pycard	-0.156563717	0.000550378
ENSMUSG00000022673	Mcm4	-0.15651864	0.003174862
ENSMUSG00000020454	Eif4enif1	-0.156500235	0.016634244
ENSMUSG00000002820	Atg4d	-0.15639778	1.71E-11
ENSMUSG00000034908	Sidt2	-0.15631466	0.003377762
ENSMUSG00000033444	Specc1l	-0.156159255	0.00398425
ENSMUSG00000038068	Rnf144b	-0.15610816	0.025963319
ENSMUSG00000025068	Gsto1	-0.156051943	6.38E-22
ENSMUSG00000032314	Etfa	-0.155743813	1.16E-10
ENSMUSG00000010406	Mrpl52	-0.155716493	1.53E-07
ENSMUSG00000022698	Naa50	-0.155337792	0.016708234
ENSMUSG00000028552	Eps15	-0.155298415	0.000252564
ENSMUSG00000006651	Aplp1	-0.155274372	0.010654996
ENSMUSG00000059811	Atl2	-0.155268301	1.51E-13
ENSMUSG00000055681	Cope	-0.155239027	7.16E-06
ENSMUSG00000001025	S100a6	-0.155083351	0.001001923
ENSMUSG00000037797	Adh4	-0.154750558	1.10E-85
ENSMUSG00000028555	Ttc39a	-0.154490216	2.33E-05
ENSMUSG00000003062	Stard3nl	-0.154027806	0.017189204
ENSMUSG00000011752	Pgam1	-0.153994972	0.000128523
ENSMUSG00000015714	Cers2	-0.153974891	0.02204387
ENSMUSG00000027248	Pdia3	-0.153872956	0.002761641
ENSMUSG00000022706	Mrpl40	-0.15373714	0.046836484
ENSMUSG00000115987	Vps28	-0.153670304	6.87E-07
ENSMUSG00000024712	Rfk	-0.153297094	0.000365595
ENSMUSG00000022514	Il1rap	-0.152810199	0.024161758
ENSMUSG00000027510	Rbm38	-0.15229251	0.015007255
ENSMUSG00000036315	Polr1h	-0.152127017	0.037303577
ENSMUSG00000006289	Osgep	-0.151913032	0.006125863
ENSMUSG00000054702	Ap1s3	-0.151737706	7.55E-51
ENSMUSG00000034663	Bmp2k	-0.151719824	4.55E-10
ENSMUSG00000030189	Ybx3	-0.151609301	5.10E-19
ENSMUSG00000022295	Atp6v1c1	-0.151542079	9.63E-05
ENSMUSG00000021190	Lgmn	-0.1515016	1.84E-07
ENSMUSG00000024887	Asah2	-0.151455199	0.000354217
ENSMUSG00000041837	Pdcd7	-0.150902954	0.043831375
ENSMUSG00000022383	Ppara	-0.150792025	1.23E-34
ENSMUSG00000087260	Lamtor5	-0.150612276	0.008556454
ENSMUSG00000040706	Agmat	-0.150507588	4.41E-32
ENSMUSG00000021754	Map3k1	-0.150472358	2.75E-06
ENSMUSG00000020483	Dynll2	-0.15033718	0.02430588
ENSMUSG00000052833	Sae1	-0.149994921	0.001842466
ENSMUSG00000057522	Spop	-0.149797415	0.000110085
ENSMUSG00000024730	Ms4a8a	-0.149504526	2.18E-05
ENSMUSG00000045886	Pam16l	-0.149351822	0.001267821
ENSMUSG00000029161	Cgref1	-0.149335318	5.38E-51
ENSMUSG00000075701	Selenos	-0.149033487	0.00031883

ENSMUSG00000029173	Sepsecs	0.205351644	0.001539384
ENSMUSG00000030138	Bms1	0.20528975	1.93E-05
ENSMUSG00000022111	Uchl3	0.205021228	0.030070943
ENSMUSG00000089847	Timm10b	0.204138237	0.000182727
ENSMUSG00000004508	Gab2	0.203939267	1.28E-05
ENSMUSG00000005198	Polr2a	0.20370872	1.12E-10
ENSMUSG00000015013	Trappc2l	0.203646995	9.01E-17
ENSMUSG00000055312	Them7	0.203577611	0.035513762
ENSMUSG00000052305	Hbb-b1	0.203539925	0.000179124
ENSMUSG00000044894	Uqcrq	0.203246783	1.38E-11
ENSMUSG00000028295	Smim8	0.203185231	1.11E-05
ENSMUSG00000020607	Lratd1	0.202555082	0.01854918
ENSMUSG00000038379	Ttk	0.20250344	0.047780096
ENSMUSG00000035247	Hectd1	0.20250122	7.87E-16
ENSMUSG00000087213	2810408I11F	0.202147732	0.000223392
ENSMUSG00000003316	Glg1	0.2018049	1.51E-10
ENSMUSG00000045410	Akr1e1	0.201785464	6.87E-18
ENSMUSG00000060002	Chpt1	0.201726147	6.12E-136
ENSMUSG00000008958	Vps72	0.201274985	0.000527685
ENSMUSG00000003948	Mmd	0.2012423	0.0001174
ENSMUSG00000046314	Stxbp6	0.201195093	0.023023893
ENSMUSG00000029798	Herc6	0.200677676	2.02E-09
ENSMUSG00000037622	Wdtc1	0.200492601	0.000183543
ENSMUSG00000030067	Foxp1	0.200277388	4.84E-07
ENSMUSG00000031309	Rps6ka3	0.19974611	8.17E-05
ENSMUSG00000060012	Kif13b	0.199737533	0.034275011
ENSMUSG00000031533	Mrps31	0.19950698	2.04E-06
ENSMUSG00000004071	Cdip1	0.199445639	8.22E-25
ENSMUSG00000021716	Srek1ip1	0.198998417	1.72E-09
ENSMUSG00000022141	Nipbl	0.198820652	1.08E-06
ENSMUSG00000006219	Fblim1	0.19868372	7.40E-16
ENSMUSG00000032423	Syncrip	0.198275417	9.25E-08
ENSMUSG00000061589	Dot1l	0.198001998	0.00736463
ENSMUSG00000086040	Wipf3	0.197910647	0.021404014
ENSMUSG00000034906	Ncaph	0.19786763	0.012436677
ENSMUSG00000029196	Tada2b	0.197201072	0.010668865
ENSMUSG00000028996	Rbp7	0.19716812	8.40E-10
ENSMUSG00000025731	Mettl26	0.196306974	9.87E-07
ENSMUSG00000027876	Reg4	0.196294351	5.20E-24
ENSMUSG00000070544	Top1	0.196177521	4.92E-11
ENSMUSG00000056851	Pcbp2	0.195898762	0.005793103
ENSMUSG00000067713	Prkag1	0.195890515	0.000505261
ENSMUSG00000028969	Cdk5	0.195877916	0.000109372
ENSMUSG00000041846	Ppp4r3a	0.19585005	0.018101556
ENSMUSG00000024645	Timm21	0.19582761	5.51E-06
ENSMUSG00000020380	Rad50	0.195566807	1.98E-11
ENSMUSG00000091387	Gcnt4	0.195329541	8.14E-08
ENSMUSG00000001569	Nom1	0.195321818	0.004336286
ENSMUSG00000028007	Snx7	0.195207915	4.19E-06
ENSMUSG00000024725	Ostf1	0.19520644	0.036777447
ENSMUSG00000022792	Yars2	0.195196182	0.04409293
ENSMUSG00000028458	Tesk1	0.194991096	0.001114266
ENSMUSG00000022617	Chkb	0.19473299	6.82E-07
ENSMUSG00000021277	Traf3	0.194370332	0.019982045
ENSMUSG00000027357	Crls1	0.194010955	0.044354991
ENSMUSG00000022013	Dnajc15	0.19388403	1.84E-10
ENSMUSG00000056537	Rlim	0.19362825	0.01990862
ENSMUSG00000038400	Pmepa1	0.193606549	0.001759583
ENSMUSG00000003531	Dgcr6	0.192852277	6.88E-09
ENSMUSG00000022865	Cxadr	0.192163058	0.000513672
ENSMUSG00000054405	Dnajc8	0.192040078	0.001661489
ENSMUSG00000038388	Pals2	0.191901749	5.27E-10
ENSMUSG00000010097	Nxf1	0.191758839	0.024420475
ENSMUSG00000038671	Arfrp1	0.19160176	4.25E-06
ENSMUSG00000000168	Dlat	0.191503949	6.09E-19
ENSMUSG00000063171	Rps4l	0.191467405	1.14E-06
ENSMUSG00000061461	Smim20	0.191129625	3.31E-05
ENSMUSG00000024286	Ccny	0.190494323	0.02202254
ENSMUSG00000015337	Endog	0.190478765	6.86E-08
ENSMUSG00000019998	Stx7	0.190431988	1.57E-16
ENSMUSG00000028861	Mrps15	0.190335131	7.29E-10
ENSMUSG00000026107	Nabp1	0.189925495	0.000382748
ENSMUSG00000027475	Kif3b	0.189646591	0.034301603
ENSMUSG00000006456	Rbm14	0.189637902	0.001527893
ENSMUSG00000037958	Nsrp1	0.189542741	0.00312632
ENSMUSG00000038095	Sbno1	0.189535685	0.000642374
ENSMUSG00000037953	A4gnt	0.189373698	0.040521671
ENSMUSG00000032637	Atxn2l	0.189296847	0.017576169
ENSMUSG00000024392	Bag6	0.189270422	0.031791721
ENSMUSG00000030612	Mrpl46	0.189210682	8.02E-10
ENSMUSG00000028069	Gpatch4	0.189176092	0.005724025
ENSMUSG00000015839	Nfe2l2	0.189009661	5.81E-79
ENSMUSG00000026526	Fh1	0.188813404	1.15E-12
ENSMUSG00000031838	Ifi30	0.188547977	1.76E-13
ENSMUSG00000064370	CYTB	0.188536897	2.21E-05
ENSMUSG00000038721	Hoxb7	0.188440997	2.30E-05
ENSMUSG00000023020	Cox14	0.188352932	4.79E-09
ENSMUSG00000020736	Nt5c	0.188319631	8.00E-05
ENSMUSG00000025019	Lcor	0.188143586	2.83E-06
ENSMUSG00000029095	Ablim2	0.188053532	0.001126048
ENSMUSG00000043962	Thrap3	0.187973934	4.59E-07
ENSMUSG00000077450	Rab11b	0.187909814	0.000180503
ENSMUSG00000057963	Itpk1	0.187701203	0.000434715
ENSMUSG00000024780	Cdc37l1	0.187490639	9.15E-06
ENSMUSG00000040322	Slc25a24	0.187458615	4.81E-26
ENSMUSG00000028862	Map3k6	0.187257241	3.04E-97
ENSMUSG00000040328	Or2v1	0.187194109	0.018832931
ENSMUSG00000004667	Polr2e	0.187194061	0.003457643
ENSMUSG00000021432	Slc35b3	0.187014733	0.018903441
ENSMUSG00000022820	Ndufb4	0.186999711	1.71E-10
ENSMUSG00000034349	Smc4	0.186936376	0.002717496
ENSMUSG00000054422	Fabp1	0.186865084	2.86E-39
ENSMUSG00000060402	Chst8	0.18668052	1.37E-09
ENSMUSG00000021457	Syk	0.186007802	0.000505261
ENSMUSG00000025940	Tmem70	0.185990222	4.89E-06
ENSMUSG00000047414	Flrt2	0.185849991	0.001118985
ENSMUSG00000071014	Ndufb6	0.185788017	1.88E-09
ENSMUSG00000031879	Ciao2b	0.185747764	1.21E-05
ENSMUSG00000038412	Higd1a	0.185381837	1.83E-20
ENSMUSG00000032384	Csnk1g1	0.185347235	0.00099799
ENSMUSG00000035151	Elmod2	0.185291908	0.004398878
ENSMUSG00000025190	Got1	0.185027806	1.79E-13
ENSMUSG00000014177	Tvp23b	0.184993077	0.000385632
ENSMUSG00000069565	Dazap1	0.184372014	0.037436518
ENSMUSG00000027007	Itprid2	0.184298939	0.002676969
ENSMUSG00000020283	Pex13	0.184252386	3.61E-05
ENSMUSG00000036769	Wdr44	0.183836062	0.019720579
ENSMUSG00000028822	Tmem50a	0.183778851	0.000810597
ENSMUSG00000025856	Pdgfa	0.183464952	0.018492819
ENSMUSG00000024036	Slc37a1	0.183450897	2.95E-05
ENSMUSG00000028233	Tgs1	0.183204204	0.034983118
ENSMUSG00000032217	Rnf111	0.183027414	0.02202254

ENSMUSG00000023045	Soat2	-0.170548581	0.010575628
ENSMUSG00000034275	Igsf9b	-0.170533375	1.74E-06
ENSMUSG00000020888	Dvl2	-0.170454308	0.002847561
ENSMUSG00000020775	Mrpl38	-0.17031611	0.009167341
ENSMUSG00000028771	Ptpn12	-0.170219465	0.001049906
ENSMUSG00000093483	AA465934	-0.169578009	0.002381984
ENSMUSG00000009633	G0s2	-0.169495363	3.79E-12
ENSMUSG00000031327	Chic1	-0.168909268	7.52E-14
ENSMUSG00000042406	Atf4	-0.168051411	0.003907827
ENSMUSG00000029777	Gars	-0.167963533	5.66E-05
ENSMUSG00000007038	Neu1	-0.16793638	1.01E-34
ENSMUSG00000022193	Psmb5	-0.16790658	0.006524472
ENSMUSG00000037573	Tob1	-0.167460889	4.50E-08
ENSMUSG00000022217	Emc9	-0.167058862	9.88E-07
ENSMUSG00000052681	Rap1b	-0.166852439	0.000408695
ENSMUSG00000032562	Gnai2	-0.166749759	0.001209862
ENSMUSG00000033170	Card10	-0.16639821	0.000607797
ENSMUSG00000031879	Ciao2b	-0.166307197	1.21E-05
ENSMUSG00000031831	Dnaaf1	-0.166089326	0.039951206
ENSMUSG00000047875	Gpr157	-0.165654946	7.74E-16
ENSMUSG00000039682	Lap3	-0.165400338	5.94E-07
ENSMUSG00000024146	Cript	-0.165136798	8.75E-09
ENSMUSG00000021614	Vcan	-0.164967389	0.024836426
ENSMUSG00000058546	Rpl23a	-0.164712882	0.000223612
ENSMUSG00000053965	Pde5a	-0.16466347	1.29E-20
ENSMUSG00000018286	Psmb6	-0.164603416	0.004856104
ENSMUSG00000042590	Ipo11	-0.164553484	0.049061965
ENSMUSG00000020664	Dld	-0.164178815	3.38E-17
ENSMUSG00000002379	Ndufa11	-0.163960235	6.05E-11
ENSMUSG00000034361	Cpne2	-0.16375996	1.75E-09
ENSMUSG00000024425	Ndfip1	-0.16318626	0.017649881
ENSMUSG00000058392	Rrp1b	-0.162842343	1.32E-05
ENSMUSG00000021629	Slc30a5	-0.162836723	7.92E-07
ENSMUSG00000014769	Psmb1	-0.162123776	0.020113341
ENSMUSG00000051355	Commd1	-0.161900371	0.000227116
ENSMUSG00000041220	Elovl6	-0.161596495	0.00055278
ENSMUSG00000045294	Insig1	-0.160294793	1.10E-14
ENSMUSG00000024067	Dpy30	-0.160193645	0.007258447
ENSMUSG00000058126	Tpm3-rs7	-0.160049909	0.009164354
ENSMUSG00000041132	N4bp2l1	-0.159903863	4.44E-23
ENSMUSG00000001100	Poldip2	-0.159853569	0.000940742
ENSMUSG00000000581	C1d	-0.159696319	2.11E-05
ENSMUSG00000068391	Chrac1	-0.159648676	6.18E-05
ENSMUSG00000038160	Atg5	-0.159544276	1.37E-10
ENSMUSG00000038722	Bud31	-0.158872154	6.22E-05
ENSMUSG00000013593	Ndufs2	-0.158582985	3.61E-25
ENSMUSG00000025393	Atp5b	-0.158397363	4.09E-18
ENSMUSG00000022184	Fbxo4	-0.158287849	0.012733301
ENSMUSG00000047446	Arl4a	-0.15827495	0.012876154
ENSMUSG00000059040	Eno1b	-0.158139992	2.71E-20
ENSMUSG00000032231	Anxa2	-0.157822011	0.004462294
ENSMUSG00000025968	Ndufs1	-0.157616903	5.29E-14
ENSMUSG00000047554	Tmem41b	-0.157485186	3.89E-05
ENSMUSG00000025495	Ptdss2	-0.157340502	0.025576878
ENSMUSG00000038642	Ctss	-0.156805142	0.000338135
ENSMUSG00000121584	Zftraf1	-0.156563301	0.020785279
ENSMUSG00000015289	Lage3	-0.15596331	0.001464773
ENSMUSG00000027679	Dnajc19	-0.155539978	6.24E-07
ENSMUSG00000050608	Micos10	-0.15549687	1.37E-12
ENSMUSG00000027775	Mfsd1	-0.15510431	3.08E-05
ENSMUSG00000017868	Sgk2	-0.155045809	0.007896575
ENSMUSG00000002475	Abhd3	-0.154865399	3.05E-05
ENSMUSG00000027540	Ptpn1	-0.154849769	0.000700134
ENSMUSG00000022635	Zcrb1	-0.154756945	0.002005285
ENSMUSG00000087687	Pet100	-0.154724951	2.29E-08
ENSMUSG00000027287	Snap23	-0.154589927	0.002896972
ENSMUSG00000038422	Hdhd3	-0.154533562	4.93E-26
ENSMUSG00000032932	Hspa13	-0.154476261	0.002339371
ENSMUSG00000063856	Gpx1	-0.154304684	5.88E-25
ENSMUSG00000013275	Slc41a1	-0.15427658	9.96E-11
ENSMUSG00000024613	Tcof1	-0.154213807	7.16E-06
ENSMUSG00000075486	Commd6	-0.154115106	5.82E-09
ENSMUSG00000031386	Hcfc1	-0.153990213	0.000425898
ENSMUSG00000020444	Guk1	-0.153905779	4.59E-28
ENSMUSG00000020368	Canx	-0.153904535	2.16E-53
ENSMUSG00000026775	Yme1l1	-0.153885268	6.79E-08
ENSMUSG00000019143	Hars2	-0.15375409	6.25E-05
ENSMUSG00000035529	Prdm4	-0.153565146	0.028233022
ENSMUSG00000098332	Pigbos1	-0.15351941	0.022526406
ENSMUSG00000015804	Med28	-0.153517567	0.000353459
ENSMUSG00000004934	Pias4	-0.153400875	0.044943342
ENSMUSG00000062270	Morf4l1	-0.153380928	0.001867921
ENSMUSG00000047139	Cd24a	-0.152239987	3.90E-09
ENSMUSG00000022452	Smdt1	-0.152085461	3.42E-10
ENSMUSG00000042700	Sipa1l1	-0.151787153	1.83E-06
ENSMUSG00000022338	Eny2	-0.151605551	1.59E-08
ENSMUSG00000022354	Ndufb9	-0.151326969	6.11E-13
ENSMUSG00000024587	Nars	-0.151276258	1.26E-14
ENSMUSG00000062981	Mrpl42	-0.151163922	4.90E-05
ENSMUSG00000016239	Lonrf3	-0.150983673	0.02617699
ENSMUSG00000027349	Fam98b	-0.150543998	0.000465022
ENSMUSG00000020142	Slc1a4	-0.150515011	2.10E-05
ENSMUSG00000032905	Atg12	-0.150443202	5.59E-06
ENSMUSG00000032123	Dpagt1	-0.150432645	1.08E-07
ENSMUSG00000037458	Azin1	-0.150334205	6.23E-27
ENSMUSG00000032065	Tex12	-0.15014043	0.000201348
ENSMUSG00000066406	Akap13	-0.150138372	7.28E-18
ENSMUSG00000084786	Ubl5	-0.149809722	7.86E-05
ENSMUSG00000032551	1110059G10	-0.149798937	0.000428276
ENSMUSG00000027210	Meis2	-0.149669247	1.23E-09
ENSMUSG00000027180	Fbxo3	-0.149494703	0.001649242
ENSMUSG00000079480	Pin4	-0.149422427	0.002995628
ENSMUSG00000071662	Polr2g	-0.148943062	0.000821776
ENSMUSG00000015668	Pdzd11	-0.14885644	0.000307315
ENSMUSG00000022617	Chkb	-0.148811999	6.82E-07
ENSMUSG00000036620	Mgat4b	-0.148624042	1.30E-15
ENSMUSG00000030452	Nipa2	-0.147996501	1.37E-06
ENSMUSG00000044393	Dsg2	-0.14775574	5.06E-18
ENSMUSG00000061731	Ext1	-0.147378744	3.55E-09
ENSMUSG00000020803	Txndc17	-0.147162243	0.002180427
ENSMUSG00000023043	Krt18	-0.146665996	4.36E-25
ENSMUSG00000041697	Cox6a1	-0.146471508	2.48E-37
ENSMUSG00000024197	Plin3	-0.146241431	2.66E-16
ENSMUSG00000027244	Atg13	-0.146066291	0.011428504
ENSMUSG00000003031	Cdkn1b	-0.146028027	0.00418167
ENSMUSG00000029269	Sult1b1	-0.145845717	1.45E-25
ENSMUSG00000030189	Ybx3	-0.145817327	5.10E-19
ENSMUSG00000029580	Actb	-0.145735838	4.38E-06
ENSMUSG00000021716	Srek1ip1	-0.145734587	1.72E-09
ENSMUSG00000025971	Maip1	-0.145697025	0.001905016
ENSMUSG00000054808	Actn4	-0.145416704	0.000860537
ENSMUSG00000063229	Ldha	-0.145139315	5.57E-29

ENSMUSG00000022957	Itsn1	0.236083206	4.70E-05
ENSMUSG00000041362	Shtn1	0.235914586	0.004063834
ENSMUSG00000034681	Rnps1	0.23579621	0.0303846
ENSMUSG00000097145	9230114K14	0.235682819	0.015298951
ENSMUSG00000032403	2300009A05	0.235373717	0.001737794
ENSMUSG00000024858	Grk2	0.235199804	0.002383016
ENSMUSG00000035401	Emsy	0.234656829	0.040590545
ENSMUSG00000034926	Dhcr24	0.23443901	4.96E-14
ENSMUSG00000038178	Slc43a2	0.234417201	7.71E-07
ENSMUSG00000061759	Armt1	0.234064852	8.34E-06
ENSMUSG00000028552	Eps15	0.233979455	0.000252564
ENSMUSG00000023973	Cnpy3	0.233950997	0.008151847
ENSMUSG00000027678	Ncoa3	0.233877669	0.000879971
ENSMUSG00000032280	Tle3	0.233749901	3.91E-23
ENSMUSG00000017376	Nlk	0.233584375	0.024814147
ENSMUSG00000016520	Lnx2	0.233124421	0.023590132
ENSMUSG00000041440	Gk5	0.233069197	0.015898488
ENSMUSG00000032624	Eml4	0.232806283	0.001590207
ENSMUSG00000034342	Cbl	0.232702114	0.00173483
ENSMUSG00000039910	Cited2	0.232695873	0.000100871
ENSMUSG00000030374	Strn4	0.231849425	4.76E-05
ENSMUSG00000035161	Ints6	0.23160803	0.015084918
ENSMUSG00000053289	Ddx10	0.231601855	0.001001706
ENSMUSG00000038116	Phf20	0.231121808	0.012154123
ENSMUSG00000042210	Abhd14a	0.230247458	0.000159987
ENSMUSG00000021843	Ktn1	0.230107729	0.003490042
ENSMUSG00000031668	Eif2ak3	0.229592734	0.002938088
ENSMUSG00000051864	Tbc1d22a	0.22920141	0.00755563
ENSMUSG00000044167	Foxo1	0.228990626	0.000111502
ENSMUSG00000025326	Ube3a	0.228941577	0.012696823
ENSMUSG00000026915	Strbp	0.228923953	0.004057186
ENSMUSG00000032727	Mier3	0.228811088	0.002513061
ENSMUSG00000013662	Atad1	0.228808518	0.006225177
ENSMUSG00000006395	Hyi	0.228679066	0.037292703
ENSMUSG00000022800	Fyttd1	0.228562004	0.001642739
ENSMUSG00000052915	Msl1	0.228277514	4.71E-05
ENSMUSG00000037280	Galnt6	0.227715066	0.040134099
ENSMUSG00000038580	Sct	0.227673884	0.033838908
ENSMUSG00000042675	Ypel3	0.227453371	0.033612746
ENSMUSG00000034258	Flvcr2	0.227442011	1.16E-21
ENSMUSG00000004931	Apba3	0.227307447	0.037895684
ENSMUSG00000032253	Phip	0.22690756	1.07E-05
ENSMUSG00000022016	Akap11	0.22668714	0.000294483
ENSMUSG00000030138	Bms1	0.226173736	1.93E-05
ENSMUSG00000027787	Nmd3	0.225460024	0.001192276
ENSMUSG00000057342	Sphk2	0.225272394	2.14E-06
ENSMUSG00000033416	Gucd1	0.225062646	5.75E-07
ENSMUSG00000042133	Ppig	0.224567206	0.006373669
ENSMUSG00000045624	Esf1	0.224071552	4.31E-05
ENSMUSG00000034109	Golim4	0.224062132	2.74E-06
ENSMUSG00000005718	Tfap4	0.223929741	0.032457048
ENSMUSG00000027993	Trim2	0.223765964	3.33E-18
ENSMUSG00000062908	Acadm	0.223552543	1.12E-06
ENSMUSG00000020978	Klhdc2	0.223481578	1.42E-07
ENSMUSG00000063273	Naa15	0.223234436	0.001708753
ENSMUSG00000049287	Iba57	0.223080394	0.000371729
ENSMUSG00000015970	Chdh	0.22269334	8.66E-05
ENSMUSG00000030103	Bhlhe40	0.222379121	0.000519872
ENSMUSG00000028261	Ndufaf4	0.22201384	2.57E-12
ENSMUSG00000033596	Rfwd3	0.220768881	0.024473224
ENSMUSG00000033159	Cnppd1	0.220628138	0.00366575
ENSMUSG00000110344	Smim36	0.220499571	0.00017335
ENSMUSG00000027630	Tbl1xr1	0.220187653	5.17E-08
ENSMUSG00000043683	Fem1a	0.220050548	0.011680706
ENSMUSG00000005198	Polr2a	0.219883609	1.12E-10
ENSMUSG00000029634	Rnf6	0.21981976	0.009088058
ENSMUSG00000056342	Usp34	0.218920897	5.10E-07
ENSMUSG00000005148	Klf5	0.218637524	3.77E-15
ENSMUSG00000028042	Zbtb7b	0.218339264	5.68E-07
ENSMUSG00000043067	Dpy19l1	0.218078644	7.46E-05
ENSMUSG00000020087	Tysnd1	0.218046482	0.015577625
ENSMUSG00000039697	Ncoa7	0.217779234	5.39E-45
ENSMUSG00000028307	Aldob	0.217497487	2.59E-132
ENSMUSG00000021365	Nedd9	0.217420017	0.010912817
ENSMUSG00000027263	Tubgcp4	0.217000096	0.00388279
ENSMUSG00000039840	Epg5	0.216966462	0.021917203
ENSMUSG00000028458	Tesk1	0.216688394	0.001114266
ENSMUSG00000054128	H2-T3	0.215749981	9.39E-10
ENSMUSG00000017688	Hnf4g	0.215664234	1.83E-15
ENSMUSG00000074698	Csnk2a1	0.215457006	1.19E-16
ENSMUSG00000003228	Grk5	0.215438628	0.030771941
ENSMUSG00000031570	Plpp5	0.214964657	0.003550684
ENSMUSG00000009569	Mrtfb	0.214724668	5.87E-05
ENSMUSG00000048897	Zfp710	0.214417282	9.67E-06
ENSMUSG00000038619	Ensa	0.214310721	0.002436046
ENSMUSG00000040520	Manea	0.214122993	4.65E-05
ENSMUSG00000057969	Sema3b	0.2138059	0.032375091
ENSMUSG00000028988	Ctnnbip1	0.213752241	0.013063757
ENSMUSG00000034382	AI661453	0.213559094	0.007791961
ENSMUSG00000030067	Foxp1	0.213437188	4.84E-07
ENSMUSG00000041609	Bicdl1	0.213059143	0.008222951
ENSMUSG00000025575	Cant1	0.212876013	2.12E-16
ENSMUSG00000031916	Cog8	0.212650051	0.000104471
ENSMUSG00000042305	Tmem183a	0.212569931	0.023265812
ENSMUSG00000029822	Osbpl3	0.212459276	0.035420084
ENSMUSG00000054999	Naaladl1	0.212452526	7.28E-99
ENSMUSG00000022553	Maf1	0.212231757	0.005006973
ENSMUSG00000019951	Bltp3b	0.212203283	1.07E-05
ENSMUSG00000028434	Epb41l4b	0.212190623	0.002760537
ENSMUSG00000008763	Man1a2	0.211827001	0.035305034
ENSMUSG00000052632	Asap2	0.211708182	2.00E-11
ENSMUSG00000031458	Coprs	0.211610653	6.13E-06
ENSMUSG00000022219	Cideb	0.211608195	0.004938028
ENSMUSG00000044791	Setd2	0.211488637	0.010159526
ENSMUSG00000055932	Fto	0.211333307	0.0294161
ENSMUSG00000078440	Dohh	0.211198118	0.001070734
ENSMUSG00000032966	Fkbp1a	0.211135267	0.003710054
ENSMUSG00000025195	Dnmbp	0.211072552	2.77E-05
ENSMUSG00000031217	Efnb1	0.211034344	1.01E-05
ENSMUSG00000027639	Samhd1	0.210878312	2.01E-05
ENSMUSG00000026039	Sgo2a	0.210700259	0.010299182
ENSMUSG00000025812	Pard3	0.210558993	7.14E-05
ENSMUSG00000035561	Aldh1b1	0.210300244	3.93E-29
ENSMUSG00000029032	Arhgef16	0.210116053	0.018036017
ENSMUSG00000091337	Eid1	0.210037309	0.015500911
ENSMUSG00000030172	Erc1	0.209849093	0.002354117
ENSMUSG00000024335	Brd2	0.209433005	0.000344449
ENSMUSG00000004263	Atn1	0.209374311	3.09E-05
ENSMUSG00000047045	Tmem164	0.209095784	4.28E-10
ENSMUSG00000030530	Furin	0.208862399	0.001944818
ENSMUSG00000089764	Gm16580	0.208095645	0.01650575
ENSMUSG00000036427	Gpi1	0.20724656	9.20E-12

ENSMUSG00000060073	Psma3	-0.148884291	0.003544068
ENSMUSG00000020888	Dvl2	-0.148766722	0.002847561
ENSMUSG00000038217	Tlcd2	-0.148675674	1.54E-11
ENSMUSG00000028013	Ppa2	-0.148403759	0.000654058
ENSMUSG00000026933	Camsap1	-0.147995692	0.036763495
ENSMUSG00000020787	P2rx1	-0.147972242	0.014791005
ENSMUSG00000034854	Mfsd12	-0.147903217	0.024100058
ENSMUSG00000042737	Dpm3	-0.147835285	0.00017594
ENSMUSG00000015342	Xk	-0.147787999	0.0014401
ENSMUSG00000014771	Pdcd2	-0.147741224	0.032537862
ENSMUSG00000029713	Gnb2	-0.147568861	0.00011614
ENSMUSG00000064367	ND5	-0.147142534	0.00118019
ENSMUSG00000034424	Gcsh	-0.146772827	0.002277266
ENSMUSG00000018171	Vmp1	-0.146729006	0.000209098
ENSMUSG00000063646	Jakmip1	-0.146671222	0.015500911
ENSMUSG00000036777	Anln	-0.146515011	0.003298138
ENSMUSG00000027160	Ccdc34	-0.14626942	0.000681991
ENSMUSG00000036646	Man1b1	-0.146250343	0.009877911
ENSMUSG00000027514	Zbp1	-0.146169432	6.98E-28
ENSMUSG00000036109	Mbnl3	-0.14593976	0.021126102
ENSMUSG00000015290	Ubl4a	-0.145939095	0.029006606
ENSMUSG00000031447	Lamp1	-0.145536321	9.09E-11
ENSMUSG00000053819	Camk2d	-0.145350722	2.97E-15
ENSMUSG00000052906	Ubxn8	-0.145275612	0.039504754
ENSMUSG00000060143	Gm10076	-0.144646084	0.004904039
ENSMUSG00000068523	Gng5	-0.144616004	0.032121894
ENSMUSG00000079057	Cyp4v3	-0.144435595	2.77E-11
ENSMUSG00000044365	Cxxc4	-0.144057128	3.04E-06
ENSMUSG00000026622	Nek2	-0.14401511	0.006035309
ENSMUSG00000036199	Ndufa13	-0.143847193	7.31E-15
ENSMUSG00000041881	Ndufa7	-0.143815388	6.97E-13
ENSMUSG00000070002	Ell	-0.143783604	2.63E-05
ENSMUSG00000012350	Ehf	-0.143665939	1.86E-33
ENSMUSG00000024993	Dennd10	-0.143508148	0.037824587
ENSMUSG00000040618	Pck2	-0.143488392	0.029008988
ENSMUSG00000028484	Psip1	-0.14331927	0.000345612
ENSMUSG00000018415	Gid4	-0.143296972	0.000609393
ENSMUSG00000026664	Phyh	-0.14325687	1.87E-40
ENSMUSG00000034917	Tjp3	-0.143213655	6.69E-15
ENSMUSG00000028873	Cdca8	-0.143067379	4.63E-13
ENSMUSG00000032458	Copb2	-0.142705352	0.001129268
ENSMUSG00000026586	Prrx1	-0.142679969	7.13E-56
ENSMUSG00000024576	Csnk1a1	-0.142623292	5.48E-07
ENSMUSG00000036644	Tbc1d9b	-0.14248997	0.000271423
ENSMUSG00000020621	Rdh14	-0.142416111	0.008358582
ENSMUSG00000039678	Tbc1d13	-0.142386853	0.014029269
ENSMUSG00000001323	Srr	-0.142312458	0.002938991
ENSMUSG00000050732	Vamp8	-0.142297354	3.69E-08
ENSMUSG00000025497	Cdhr5	-0.142238168	0.0021332
ENSMUSG00000003778	Brd8	-0.14220303	0.00310048
ENSMUSG00000048170	Mcmbp	-0.142173816	3.05E-08
ENSMUSG00000022391	Rangap1	-0.142058595	1.79E-11
ENSMUSG00000026234	Ncl	-0.141885416	2.96E-12
ENSMUSG00000096188	Cmtm4	-0.141620184	9.83E-12
ENSMUSG00000011114	Tbrg1	-0.141497817	1.79E-10
ENSMUSG00000091537	Tma7	-0.141447567	1.83E-12
ENSMUSG00000015652	Steap1	-0.141299308	0.001340911
ENSMUSG00000031902	Nfatc3	-0.141084963	2.73E-05
ENSMUSG00000040605	Bace2	-0.140890413	5.54E-07
ENSMUSG00000002661	Alkbh7	-0.140889541	8.54E-05
ENSMUSG00000034343	Ube2f	-0.140486561	3.24E-06
ENSMUSG00000025144	Cenpx	-0.14032491	6.34E-05
ENSMUSG00000002580	Mien1	-0.140305389	4.30E-10
ENSMUSG00000039648	Kyat1	-0.139326323	0.000199123
ENSMUSG00000041132	N4bp2l1	-0.139225142	4.44E-23
ENSMUSG00000037280	Galnt6	-0.139190961	0.040134099
ENSMUSG00000022824	Muc13	-0.138964638	0.023220419
ENSMUSG00000025971	Maip1	-0.138638309	0.001905016
ENSMUSG00000039886	Tmem120a	-0.138263394	2.98E-10
ENSMUSG00000002297	Dbf4	-0.138251127	0.004317406
ENSMUSG00000073792	Alg6	-0.137895164	0.017174658
ENSMUSG00000000568	Hnrnpd	-0.137873933	1.83E-05
ENSMUSG00000041355	Ssr2	-0.137453024	0.001026968
ENSMUSG00000031458	Coprs	-0.136931741	6.13E-06
ENSMUSG00000030878	Cdr2	-0.13688602	6.78E-26
ENSMUSG00000038683	Pak1ip1	-0.136795664	4.49E-06
ENSMUSG00000024750	Zfand5	-0.136780536	0.002202407
ENSMUSG00000018567	Gabarap	-0.136621048	0.000238251
ENSMUSG00000042812	Foxf1	-0.136552997	0.000365416
ENSMUSG00000022681	Ntan1	-0.136494582	0.000353334
ENSMUSG00000020409	Slu7	-0.136460249	1.53E-15
ENSMUSG00000014769	Psmb1	-0.136360695	0.020113341
ENSMUSG00000022800	Fyttd1	-0.136237943	0.001642739
ENSMUSG00000039515	Ptpa	-0.136056158	0.004520609
ENSMUSG00000035632	Cnot3	-0.135906502	0.00421901
ENSMUSG00000060063	Alox5ap	-0.1356197	1.07E-05
ENSMUSG00000015542	Nat9	-0.135611297	1.59E-06
ENSMUSG00000021474	Sfxn1	-0.13534915	9.27E-08
ENSMUSG00000018379	Srsf1	-0.135200235	7.87E-08
ENSMUSG00000016946	Kctd5	-0.135171375	0.015668953
ENSMUSG00000024169	Ift140	-0.134992466	0.026863897
ENSMUSG00000062580	Timm17a	-0.134946802	3.64E-05
ENSMUSG00000022124	Fbxl3	-0.134890533	0.000923024
ENSMUSG00000052160	Pld4	-0.134842779	0.030031535
ENSMUSG00000049608	Gpr55	-0.134779754	0.006189469
ENSMUSG00000090150	Acad11	-0.134572098	3.53E-12
ENSMUSG00000074064	Mlycd	-0.134386864	7.77E-06
ENSMUSG00000005469	Prkaca	-0.134236032	0.000734215
ENSMUSG00000004789	Dlst	-0.134167451	5.48E-13
ENSMUSG00000038712	Mindy1	-0.134136869	1.71E-15
ENSMUSG00000056671	Prelid2	-0.134131934	0.022525236
ENSMUSG00000026991	Pkp4	-0.134060952	3.46E-07
ENSMUSG00000018920	Cxcl16	-0.133949251	8.52E-07
ENSMUSG00000050786	Ccdc126	-0.133815261	0.015845996
ENSMUSG00000058587	Tmod3	-0.133518059	0.0030253
ENSMUSG00000021496	Pcbd2	-0.13341747	0.000105556
ENSMUSG00000038174	Hycc2	-0.133330308	5.51E-07
ENSMUSG00000029472	Anapc5	-0.13320339	0.041061966
ENSMUSG00000048327	Ckap2l	-0.133111002	4.27E-06
ENSMUSG00000028138	Adh5	-0.133038696	6.01E-27
ENSMUSG00000027864	Ptgfrn	-0.133037358	4.43E-05
ENSMUSG00000023070	Rgn	-0.13293107	0.000147608
ENSMUSG00000032501	Trib1	-0.132757315	2.62E-39
ENSMUSG00000039270	Megf9	-0.132726562	1.08E-08
ENSMUSG00000020821	Kif1c	-0.132604376	3.89E-12
ENSMUSG00000044827	Tlr1	-0.132499423	0.027753045
ENSMUSG00000026307	Scly	-0.132371727	1.27E-06
ENSMUSG00000041119	Pde9a	-0.13235442	0.000368335
ENSMUSG00000031878	Nae1	-0.131605918	2.64E-07
ENSMUSG00000028699	Tspan1	-0.131322212	9.13E-47
ENSMUSG00000097184	4632428C04	-0.131219388	0.009779296
ENSMUSG00000061904	Slc25a3	-0.131113189	2.72E-18

ENSMUSG00000020706	Ftsj3	0.182969665	0.0024207
ENSMUSG00000026638	Irf6	0.182904262	0.002084516
ENSMUSG00000026974	Zmynd19	0.182807953	0.017452383
ENSMUSG00000022450	Ndufa6	0.182801367	8.38E-10
ENSMUSG00000026667	Uhmk1	0.182215879	5.81E-05
ENSMUSG00000038256	Bcl9	0.18208394	3.82E-09
ENSMUSG00000024854	Pold4	0.18192507	0.009358511
ENSMUSG00000013662	Atad1	0.181461866	0.006225177
ENSMUSG00000059734	Ndufs8	0.181237031	1.56E-06
ENSMUSG00000035762	Tmem161b	0.180927907	4.16E-07
ENSMUSG00000079017	Ifi27l2a	0.180313341	9.90E-10
ENSMUSG00000064351	COX1	0.180154455	3.07E-06
ENSMUSG00000084883	Ccdc85c	0.179944597	0.046449504
ENSMUSG00000031163	Glod5	0.179778962	4.42E-28
ENSMUSG00000028565	Nfia	0.179722336	3.08E-08
ENSMUSG00000051671	Coa6	0.179709863	0.000473174
ENSMUSG00000029086	Prom1	0.179530687	1.95E-28
ENSMUSG00000040865	Ino80d	0.179506675	2.90E-06
ENSMUSG00000068284	Usf3	0.179427118	0.001926304
ENSMUSG00000043445	Pgp	0.179184606	1.75E-10
ENSMUSG00000037601	Nme1	0.179161495	0.007066435
ENSMUSG00000027200	Sema6d	0.179030918	0.033189239
ENSMUSG00000040046	Tph1	0.178775753	4.25E-05
ENSMUSG00000027173	Depdc7	0.178575737	0.000519157
ENSMUSG00000044352	Sowaha	0.178393224	1.27E-23
ENSMUSG00000022437	Samm50	0.178222347	0.008000815
ENSMUSG00000039523	Cep104	0.177931137	0.010270764
ENSMUSG00000049091	Sephs2	0.177870587	4.68E-11
ENSMUSG00000018796	Acsl1	0.177259752	9.13E-13
ENSMUSG00000034947	Tmem106a	0.176926225	0.000552727
ENSMUSG00000022387	Brd1	0.175996538	5.10E-07
ENSMUSG00000055044	Pdlim1	0.175914413	3.92E-27
ENSMUSG00000000275	Trim25	0.175808975	0.001877963
ENSMUSG00000029610	Aimp2	0.175697194	0.011347984
ENSMUSG00000033685	Ucp2	0.175471919	4.37E-06
ENSMUSG00000000278	Scpep1	0.175378713	3.18E-07
ENSMUSG00000049401	Ogfr	0.175327763	0.020461029
ENSMUSG00000020447	Npc1l1	0.175281324	1.55E-06
ENSMUSG00000026343	Gpr39	0.175140852	9.40E-05
ENSMUSG00000005698	Ctcf	0.175048344	0.000327504
ENSMUSG00000000563	Atp5pb	0.175045036	5.29E-13
ENSMUSG00000021553	Slc28a3	0.174714403	0.017811691
ENSMUSG00000022354	Ndufb9	0.174653262	6.11E-13
ENSMUSG00000029462	Vps29	0.174621348	9.06E-05
ENSMUSG00000025759	Mfsd8	0.174478837	0.02769699
ENSMUSG00000016757	Ttll12	0.174299605	0.001377849
ENSMUSG00000066839	Ecsit	0.174109289	4.68E-08
ENSMUSG00000061136	Prpf40a	0.17393031	0.011553751
ENSMUSG00000032254	Kif23	0.173906079	2.39E-06
ENSMUSG00000030615	Tmem126a	0.173739282	2.34E-08
ENSMUSG00000057143	Trim12c	0.173713728	9.35E-05
ENSMUSG00000049421	Zfp260	0.173544091	0.036163918
ENSMUSG00000069792	Wfdc17	0.173416499	8.32E-06
ENSMUSG00000056076	Eif3b	0.173305385	1.08E-06
ENSMUSG00000053094	Tmem248	0.173153544	0.010951921
ENSMUSG00000094410	Zbed6	0.173054437	0.001192621
ENSMUSG00000074102	Rbm15b	0.172386636	5.97E-07
ENSMUSG00000000544	Gpa33	0.172338653	0.013351547
ENSMUSG00000038170	Pde4dip	0.172101501	0.000727724
ENSMUSG00000038619	Ensa	0.17187789	0.002436046
ENSMUSG00000034341	Wbp2	0.171825399	5.67E-05
ENSMUSG00000024846	Cst6	0.17158323	0.041240361
ENSMUSG00000027792	Bche	0.171288563	7.31E-60
ENSMUSG00000000581	C1d	0.170897966	2.11E-05
ENSMUSG00000021377	Dek	0.170789492	0.000146036
ENSMUSG00000032667	Pon2	0.170749194	3.74E-21
ENSMUSG00000096546	Smlr1	0.17049519	8.38E-37
ENSMUSG00000033909	Usp36	0.170217178	0.001926304
ENSMUSG00000041143	Tmco4	0.170174502	3.70E-06
ENSMUSG00000023921	Mmut	0.170112243	0.00177606
ENSMUSG00000031299	Pdha1	0.16968708	2.95E-05
ENSMUSG00000040785	Ttc3	0.169558222	0.0149099
ENSMUSG00000037257	Aagab	0.169376351	0.000871688
ENSMUSG00000036918	Ttc7	0.169346085	0.009317429
ENSMUSG00000034566	Atp5h	0.16932234	1.36E-11
ENSMUSG00000018965	Ywhah	0.169319432	0.040353325
ENSMUSG00000037287	Tbcel	0.169022276	0.000697929
ENSMUSG00000042520	Ubap2l	0.168904577	0.001897836
ENSMUSG00000020451	Limk2	0.168884193	7.30E-07
ENSMUSG00000020124	Usp15	0.168829686	0.001111645
ENSMUSG00000024181	Mrpl28	0.168731377	1.71E-05
ENSMUSG00000030660	Pik3c2a	0.168508716	1.15E-10
ENSMUSG00000024096	Ralbp1	0.16841126	0.003563409
ENSMUSG00000026219	Trip12	0.168303896	0.004081927
ENSMUSG00000036693	Nop14	0.167942704	0.036894846
ENSMUSG00000032299	Commd4	0.167704151	0.026572224
ENSMUSG00000052713	Zfp608	0.167688869	0.003006681
ENSMUSG00000057738	Sptan1	0.167669969	3.54E-09
ENSMUSG00000008475	Arpc5	0.167504685	0.010850966
ENSMUSG00000028599	Tnfrsf1b	0.167487693	3.21E-05
ENSMUSG00000074994	Qser1	0.167478992	5.18E-07
ENSMUSG00000036504	Phpt1	0.167255812	0.005297176
ENSMUSG00000061288	Taok3	0.167084172	3.96E-12
ENSMUSG00000055670	Zzef1	0.166789034	0.025987712
ENSMUSG00000070284	Gmppb	0.166584177	1.49E-09
ENSMUSG00000014226	Cacybp	0.166524024	0.004743943
ENSMUSG00000045438	Cox19	0.166384085	3.80E-05
ENSMUSG00000028062	Lamtor2	0.166225022	0.002333436
ENSMUSG00000028790	Khdrbs1	0.166211176	0.008698054
ENSMUSG00000027984	Hadh	0.1659465	1.73E-32
ENSMUSG00000005656	Snx6	0.165695865	0.000478555
ENSMUSG00000055065	Ddx17	0.165501042	0.045454589
ENSMUSG00000035898	Uba6	0.165366754	0.018622287
ENSMUSG00000022856	Tmem41a	0.165179833	0.041177463
ENSMUSG00000020180	Snrpd3	0.164944885	0.009838726
ENSMUSG00000022227	Mcpt1	0.164942124	0.009288642
ENSMUSG00000021665	Hexb	0.164857017	0.000391653
ENSMUSG00000092417	Gpank1	0.164722145	3.31E-09
ENSMUSG00000034427	Myo15b	0.164519211	3.04E-06
ENSMUSG00000031827	Cotl1	0.16429483	0.014262929
ENSMUSG00000117679	Apbb3	0.163818304	0.031183314
ENSMUSG00000000881	Dlg3	0.163760782	0.042395818
ENSMUSG00000073598	Smim33	0.163695479	0.001412539
ENSMUSG00000032018	Sc5d	0.163681563	0.035711247
ENSMUSG00000020604	Arsg	0.163631237	1.94E-71
ENSMUSG00000015812	Gnrh1	0.163584697	0.014262929
ENSMUSG00000025492	Ifitm3	0.163520803	7.20E-05
ENSMUSG00000009076	Zmat5	0.163302172	0.015857572
ENSMUSG00000035011	Zbtb7a	0.16316357	4.68E-11
ENSMUSG00000021967	Mrpl57	0.162587716	0.005246525
ENSMUSG00000040225	Prrc2c	0.162525472	3.93E-07
ENSMUSG00000033467	Crlf2	0.162470054	4.52E-05

ENSMUSG00000047822	Angptl8	-0.143827762	8.25E-05
ENSMUSG00000061315	Naca	-0.143406203	0.022397199
ENSMUSG00000040945	Rcc2	-0.143301255	2.81E-26
ENSMUSG00000015013	Trappc2l	-0.142776899	9.01E-17
ENSMUSG00000007812	Zfp655	-0.142727369	0.017899446
ENSMUSG00000027533	Fabp5	-0.142670798	7.23E-05
ENSMUSG00000052833	Sae1	-0.142206474	0.001842466
ENSMUSG00000032328	Tmem30a	-0.142014913	1.68E-11
ENSMUSG00000030082	Sec61a1	-0.141837418	1.49E-05
ENSMUSG00000026447	Pik3c2b	-0.141789862	1.03E-06
ENSMUSG00000029622	Arpc1b	-0.141613101	0.00360538
ENSMUSG00000032437	Stt3b	-0.141541281	6.86E-07
ENSMUSG00000025903	Lypla1	-0.141526245	4.31E-07
ENSMUSG00000041237	Pklr	-0.141441269	1.49E-16
ENSMUSG00000029014	Dnajc2	-0.14135809	2.51E-05
ENSMUSG00000035754	Wdr18	-0.141197835	0.031528933
ENSMUSG00000020250	Txnrd1	-0.140672041	1.37E-09
ENSMUSG00000049038	Mterf2	-0.140593534	9.87E-15
ENSMUSG00000025351	Cd63	-0.140121637	1.98E-05
ENSMUSG00000020402	Vdac1	-0.140021579	0.022825865
ENSMUSG00000026192	Atic	-0.139894013	0.01177418
ENSMUSG00000035151	Elmod2	-0.139803931	0.004398878
ENSMUSG00000020911	Krt19	-0.139515093	5.11E-46
ENSMUSG00000022519	Srl	-0.139394069	0.009523928
ENSMUSG00000000568	Hnrnpd	-0.139219666	1.83E-05
ENSMUSG00000062931	Zfp938	-0.138827205	0.003742216
ENSMUSG00000067825	Pex26	-0.13879845	0.004611191
ENSMUSG00000053286	Trmt1l	-0.138787574	0.002944447
ENSMUSG00000028013	Ppa2	-0.138787039	0.000654058
ENSMUSG00000003134	Tbc1d8	-0.138445515	0.005972119
ENSMUSG00000029394	Cdk2ap1	-0.138438197	8.14E-06
ENSMUSG00000039879	Heca	-0.137721785	5.97E-10
ENSMUSG00000024346	Pfdn1	-0.137523779	0.005937245
ENSMUSG00000019370	Calm3	-0.137504425	0.010434852
ENSMUSG00000115987	Vps28	-0.136151784	6.87E-07
ENSMUSG00000053329	Gatd3a	-0.136073408	0.0001302
ENSMUSG00000015745	Plekho1	-0.135962256	0.016410247
ENSMUSG00000003518	Dusp3	-0.135891213	0.011052933
ENSMUSG00000073155	Stmp1	-0.135447385	1.87E-16
ENSMUSG00000004667	Polr2e	-0.135399441	0.003457643
ENSMUSG00000046062	Ppp1r15b	-0.135259068	1.29E-06
ENSMUSG00000002804	Nudt14	-0.134995988	6.47E-05
ENSMUSG00000021696	Elovl7	-0.134691497	1.87E-12
ENSMUSG00000015290	Ubl4a	-0.134590431	0.029006606
ENSMUSG00000029710	Ephb4	-0.134498204	0.013151243
ENSMUSG00000001576	Ergic1	-0.133883279	7.05E-05
ENSMUSG00000009549	Srp14	-0.133368791	0.00396542
ENSMUSG00000026896	Ifih1	-0.133259271	1.62E-08
ENSMUSG00000046727	Cystm1	-0.133258791	2.21E-16
ENSMUSG00000071415	Rpl23	-0.133171316	0.000435042
ENSMUSG00000024646	Cyb5a	-0.133118655	4.24E-29
ENSMUSG00000029922	Mkrn1	-0.132877746	4.48E-08
ENSMUSG00000050697	Prkaa1	-0.132630593	0.001108449
ENSMUSG00000052151	Plpp2	-0.132619468	0.000126081
ENSMUSG00000061136	Prpf40a	-0.132432246	0.011553751
ENSMUSG00000009863	Sdhb	-0.131751585	5.40E-32
ENSMUSG00000028572	Hook1	-0.131629507	0.005157732
ENSMUSG00000078681	Tm2d3	-0.131449654	0.000509931
ENSMUSG00000063171	Rps4l	-0.13134942	1.14E-06
ENSMUSG00000042737	Dpm3	-0.13113374	0.00017594
ENSMUSG00000033105	Lss	-0.130832059	0.008348828
ENSMUSG00000085148	Mir22hg	-0.130810756	1.06E-08
ENSMUSG00000024583	Txnl1	-0.130692785	0.004215918
ENSMUSG00000038803	Ost4	-0.130626378	7.60E-05
ENSMUSG00000033590	Myo5c	-0.130570024	1.57E-14
ENSMUSG00000019710	Mrpl24	-0.130487007	6.94E-06
ENSMUSG00000036073	Galt	-0.13038644	0.020006738
ENSMUSG00000030104	Edem1	-0.130203776	0.000336271
ENSMUSG00000028007	Snx7	-0.130024728	4.19E-06
ENSMUSG00000036377	Cracd	-0.130009705	6.07E-10
ENSMUSG00000029642	Polr1d	-0.129722688	0.000774023
ENSMUSG00000026895	Ndufa8	-0.128764701	1.84E-10
ENSMUSG00000068921	Dap3	-0.128759164	0.000913741
ENSMUSG00000031314	Taf1	-0.128744987	0.006488642
ENSMUSG00000031762	Mt2	-0.128677558	0.000905458
ENSMUSG00000021585	Cast	-0.128517994	2.30E-12
ENSMUSG00000015094	Npdc1	-0.128354941	0.005348605
ENSMUSG00000030660	Pik3c2a	-0.12829071	1.15E-10
ENSMUSG00000041037	Irgq	-0.127825869	0.032945195
ENSMUSG00000017778	Cox7c	-0.127804113	1.73E-10
ENSMUSG00000035000	Dpp4	-0.127741529	7.96E-12
ENSMUSG00000061904	Slc25a3	-0.127471337	2.72E-18
ENSMUSG00000019189	Rnf145	-0.127466165	2.64E-12
ENSMUSG00000035227	Spcs2	-0.127285959	1.30E-10
ENSMUSG00000027954	Efna1	-0.127030027	5.94E-08
ENSMUSG00000054342	Kcnn4	-0.126546407	2.73E-10
ENSMUSG00000046324	Ermp1	-0.126520642	0.047106589
ENSMUSG00000052397	Ezr	-0.126397595	6.22E-17
ENSMUSG00000025364	Pa2g4	-0.126200455	0.013327827
ENSMUSG00000033294	Noc4l	-0.125999737	0.005110189
ENSMUSG00000049091	Sephs2	-0.125972815	4.68E-11
ENSMUSG00000024750	Zfand5	-0.125840154	0.002202407
ENSMUSG00000046668	Cxxc5	-0.125794739	0.000183961
ENSMUSG00000062456	Rpl9-ps6	-0.125505761	0.001927044
ENSMUSG00000040728	Esrp1	-0.125190307	3.82E-13
ENSMUSG00000027406	Idh3b	-0.125062302	6.30E-15
ENSMUSG00000085042	Abhd11os	-0.124895362	3.50E-06
ENSMUSG00000031529	Tnks	-0.124871271	0.001280563
ENSMUSG00000027472	Pdrg1	-0.124726117	0.000692529
ENSMUSG00000044991	Shld1	-0.124400442	0.001907013
ENSMUSG00000001098	Kctd10	-0.12431419	8.45E-17
ENSMUSG00000039774	Galnt12	-0.124301673	2.21E-18
ENSMUSG00000022681	Ntan1	-0.124027869	0.000353334
ENSMUSG00000020585	Laptm4a	-0.123964222	9.83E-07
ENSMUSG00000021494	Ddx41	-0.123683322	0.009364853
ENSMUSG00000025509	Pnpla2	-0.123582881	0.008168715
ENSMUSG00000077704	Snord89	-0.123357056	1.37E-17
ENSMUSG00000027637	Rab5if	-0.122994158	8.79E-19
ENSMUSG00000052962	Mrpl35	-0.12256937	0.002259251
ENSMUSG00000029148	Nrbp1	-0.122405571	0.001002166
ENSMUSG00000020358	Hnrnpab	-0.122283175	0.001345076
ENSMUSG00000078546	Zfp995	-0.121631276	0.004839236
ENSMUSG00000022241	Tars	-0.121605611	0.025814688
ENSMUSG00000035429	Ptprh	-0.121549257	0.000245171
ENSMUSG00000024085	Man2a1	-0.121494196	0.000397208
ENSMUSG00000038398	Upf3a	-0.121114488	0.014132997
ENSMUSG00000022575	Gsdmd	-0.121076529	6.17E-10
ENSMUSG00000024217	Snrpc	-0.120979016	0.000599179
ENSMUSG00000072235	Tuba1a	-0.120743986	0.000544036
ENSMUSG00000028452	Vcp	-0.120726798	5.32E-05
ENSMUSG00000031278	Acsl4	-0.120632291	0.042971842
ENSMUSG00000070738	Dgkd	-0.120405486	1.17E-08

ENSMUSG00000053094	Tmem248	0.20714003	0.010951921
ENSMUSG00000024073	Birc6	0.207044235	1.62E-05
ENSMUSG00000093904	Tomm20	0.206468816	0.001496085
ENSMUSG00000035064	Eef2k	0.206254846	2.29E-09
ENSMUSG00000048756	Foxo3	0.206098581	0.000191803
ENSMUSG00000024498	Tcerg1	0.20587954	8.86E-06
ENSMUSG00000028862	Map3k6	0.205686977	3.04E-97
ENSMUSG00000066036	Ubr4	0.20558538	1.26E-05
ENSMUSG00000028833	Ncdn	0.205253721	0.004002798
ENSMUSG00000032604	Qars	0.205068636	1.36E-12
ENSMUSG00000055065	Ddx17	0.204922712	0.045454589
ENSMUSG00000037519	Ppfia1	0.204319151	0.001088741
ENSMUSG00000068686	Cd59b	0.203864425	3.48E-07
ENSMUSG00000020910	Adprm	0.203723173	0.000234352
ENSMUSG00000039671	Zmynd8	0.203212431	0.000408024
ENSMUSG00000021242	Npc2	0.202459846	0.022767552
ENSMUSG00000032547	Ryk	0.20243759	3.70E-17
ENSMUSG00000033228	Scaf11	0.201879729	0.000657344
ENSMUSG00000018974	Sart3	0.201485276	0.024748481
ENSMUSG00000039395	Mreg	0.201393161	1.16E-08
ENSMUSG00000041782	Lad1	0.201047245	8.38E-05
ENSMUSG00000051989	Smim11	0.200901082	0.007585796
ENSMUSG00000040435	Ppp1r15a	0.200317216	0.046438592
ENSMUSG00000001569	Nom1	0.200168228	0.004336286
ENSMUSG00000033629	Hacd3	0.20004463	0.021605147
ENSMUSG00000024807	Syvn1	0.200020015	8.18E-05
ENSMUSG00000068587	Mgam	0.199753712	1.43E-05
ENSMUSG00000006219	Fblim1	0.199704435	7.40E-16
ENSMUSG00000032047	Acat1	0.199695805	1.86E-52
ENSMUSG00000032288	Imp3	0.199679163	0.000706544
ENSMUSG00000020315	Sptbn1	0.199625065	5.80E-14
ENSMUSG00000057672	Pkn1	0.199407546	0.001547124
ENSMUSG00000056493	Foxk1	0.199327892	4.27E-06
ENSMUSG00000001157	Gmcl1	0.199127057	0.011181699
ENSMUSG00000040054	Baz2a	0.199016695	0.001320004
ENSMUSG00000005958	Ephb3	0.198897964	2.22E-11
ENSMUSG00000038733	Wdr26	0.198732031	1.39E-06
ENSMUSG00000050390	C77080	0.198590077	7.88E-08
ENSMUSG00000015711	Prune1	0.198209372	1.31E-10
ENSMUSG00000000303	Cdh1	0.198120218	1.46E-10
ENSMUSG00000075520	Malrd1	0.197701525	7.41E-29
ENSMUSG00000056938	Acbd4	0.197583625	2.58E-14
ENSMUSG00000027534	Snx16	0.197376743	0.001586423
ENSMUSG00000020621	Rdh14	0.197066658	0.008358582
ENSMUSG00000022742	Cpox	0.197048548	6.75E-08
ENSMUSG00000032570	Atp2c1	0.197032049	0.000233569
ENSMUSG00000034621	Gpatch8	0.196776926	0.005990325
ENSMUSG00000000394	Gcg	0.196708374	4.31E-05
ENSMUSG00000070939	Tgfbrap1	0.196593843	0.000617831
ENSMUSG00000011179	Odc1	0.196367137	0.002633852
ENSMUSG00000066233	Tmem42	0.196229852	4.81E-05
ENSMUSG00000026110	Mgat4a	0.196173446	1.46E-15
ENSMUSG00000045438	Cox19	0.196165219	3.80E-05
ENSMUSG00000038965	Ube2l3	0.196159429	0.035699997
ENSMUSG00000038260	Trpm4	0.196158697	0.000332361
ENSMUSG00000058301	Upf1	0.195981679	0.029220285
ENSMUSG00000030472	Ceacam18	0.195450565	2.34E-46
ENSMUSG00000029701	Rbm28	0.195393449	0.00391872
ENSMUSG00000025868	Higd2a	0.195385419	1.37E-06
ENSMUSG00000006717	Acot13	0.195317548	7.49E-21
ENSMUSG00000058006	Mdn1	0.195189007	0.015238699
ENSMUSG00000029422	Rsrc2	0.195099586	0.016141346
ENSMUSG00000029186	Pi4k2b	0.194624487	8.38E-10
ENSMUSG00000036617	Etl4	0.194376878	2.94E-07
ENSMUSG00000025409	Mbd6	0.194281444	0.000223559
ENSMUSG00000004207	Psap	0.194176254	0.00021099
ENSMUSG00000011960	Ccnt1	0.193760296	0.0315752
ENSMUSG00000020986	Sec23a	0.193522251	4.23E-06
ENSMUSG00000034880	Mrpl34	0.192799114	7.30E-15
ENSMUSG00000046139	Patl1	0.192522417	0.045071816
ENSMUSG00000106864	Gtf3c2	0.191799867	0.010612663
ENSMUSG00000036450	Hif1an	0.191755412	0.007579951
ENSMUSG00000030748	Il4ra	0.190794902	0.000913741
ENSMUSG00000026754	Golga1	0.190657599	1.29E-13
ENSMUSG00000029505	Ep400	0.190446456	1.74E-06
ENSMUSG00000022546	Gpt	0.190154191	3.76E-10
ENSMUSG00000034158	Lrrc58	0.19003398	0.000789598
ENSMUSG00000029378	Areg	0.190031385	0.000584974
ENSMUSG00000029202	Pds5a	0.190026531	4.16E-05
ENSMUSG00000019810	Fuca2	0.189652485	1.88E-10
ENSMUSG00000062526	Mppe1	0.189171961	3.93E-09
ENSMUSG00000028822	Tmem50a	0.18914444	0.000810597
ENSMUSG00000032582	Rbm6	0.188886721	1.21E-05
ENSMUSG00000025408	Ddit3	0.188561703	6.61E-07
ENSMUSG00000030934	Oat	0.188541621	1.00E-06
ENSMUSG00000036057	Ptpn23	0.188254605	0.011620851
ENSMUSG00000030652	Coq7	0.188054105	0.001296421
ENSMUSG00000062929	Cfl2	0.18800626	0.014344397
ENSMUSG00000009076	Zmat5	0.187921999	0.015857572
ENSMUSG00000027304	Rtf1	0.187793222	9.76E-06
ENSMUSG00000027555	Car13	0.187732948	0.002240767
ENSMUSG00000027820	Mme	0.187605837	5.61E-91
ENSMUSG00000078636	Gm7336	0.18756901	2.62E-06
ENSMUSG00000040297	Suco	0.187398691	0.001290253
ENSMUSG00000042202	Slc35e2	0.187287256	0.023242209
ENSMUSG00000051853	Arf3	0.187279541	5.35E-32
ENSMUSG00000022387	Brd1	0.186867578	5.10E-07
ENSMUSG00000014077	Chp1	0.186750993	0.002308611
ENSMUSG00000028412	Slc44a1	0.186723367	9.08E-24
ENSMUSG00000055553	Kxd1	0.186710541	0.044021187
ENSMUSG00000033735	Spr	0.186566567	1.17E-05
ENSMUSG00000054309	Cpsf3	0.186239935	0.004392996
ENSMUSG00000052609	Plekhg3	0.186188998	0.000829264
ENSMUSG00000006517	Mvd	0.185996262	4.22E-05
ENSMUSG00000039219	Arid4b	0.185914396	0.002214606
ENSMUSG00000020671	Rab10	0.18584704	1.24E-07
ENSMUSG00000033237	Arid2	0.185670229	0.001153339
ENSMUSG00000033767	Tmem131l	0.185142523	0.009364853
ENSMUSG00000028996	Rbp7	0.184654819	8.40E-10
ENSMUSG00000025037	Maoa	0.184533461	1.01E-18
ENSMUSG00000032737	Inppl1	0.184486786	0.000311144
ENSMUSG00000051469	Zfp24	0.184078517	0.001502534
ENSMUSG00000032410	Xrn1	0.183709566	0.00063772
ENSMUSG00000056758	Hmga2	0.18358312	0.013520105
ENSMUSG00000050931	Sgms2	0.183234567	0.017488323
ENSMUSG00000026617	Bpnt1	0.183190827	1.44E-98
ENSMUSG00000030866	Ern2	0.18306485	9.57E-10
ENSMUSG00000027782	Kpna4	0.182475677	0.007506413
ENSMUSG00000020647	Ncoa1	0.18192266	3.57E-07
ENSMUSG00000032018	Sc5d	0.181813964	0.035711247
ENSMUSG00000031391	L1cam	0.181271287	7.91E-12
ENSMUSG00000050705	2310061I04R	0.181178076	0.048716002

ENSMUSG00000026028	Trak2	-0.131041885	0.016771673
ENSMUSG00000029822	Osbpl3	-0.13084965	0.035420084
ENSMUSG00000028463	Car9	-0.130692209	0.005196348
ENSMUSG00000032741	Tpcn1	-0.130315286	0.000253703
ENSMUSG00000025027	Xpnpep1	-0.12987053	1.43E-25
ENSMUSG00000027244	Atg13	-0.129851713	0.011428504
ENSMUSG00000024981	Acsl5	-0.129783487	2.10E-32
ENSMUSG00000024498	Tcerg1	-0.129735598	8.86E-06
ENSMUSG00000031913	Vps4a	-0.129383732	0.035299273
ENSMUSG00000058446	Znrf2	-0.12936174	3.10E-06
ENSMUSG00000021662	Arhgef28	-0.129004501	0.000187268
ENSMUSG00000020307	Cdc34	-0.128903202	0.001502892
ENSMUSG00000005804	Bloc1s6	-0.128801025	0.001546299
ENSMUSG00000029147	Ppm1g	-0.128690526	0.029605337
ENSMUSG00000038914	Dido1	-0.128282169	0.040505462
ENSMUSG00000039568	Ubald1	-0.128126045	0.004669741
ENSMUSG00000040675	Mthfd1l	-0.128035906	0.004026717
ENSMUSG00000017715	Pgs1	-0.127964	0.034162871
ENSMUSG00000031105	Slc25a14	-0.127735598	0.031298191
ENSMUSG00000022040	Ephx2	-0.12768944	3.62E-85
ENSMUSG00000015112	Slc25a13	-0.127665599	1.79E-19
ENSMUSG00000020911	Krt19	-0.127654852	5.11E-46
ENSMUSG00000039640	Mrpl12	-0.127612908	6.01E-19
ENSMUSG00000019505	Ubb	-0.127536929	1.55E-05
ENSMUSG00000071528	Atp5md	-0.127304441	1.57E-10
ENSMUSG00000022244	Amacr	-0.12723082	1.48E-06
ENSMUSG00000028672	Hmgcl	-0.127124616	5.38E-09
ENSMUSG00000040511	Pvr	-0.127054651	0.008462844
ENSMUSG00000031993	Snx19	-0.127045665	0.022501547
ENSMUSG00000044734	Serpinb1a	-0.126987558	0.001995062
ENSMUSG00000021832	Psmc6	-0.126946699	0.00416813
ENSMUSG00000038039	Gcc2	-0.126819212	0.000123318
ENSMUSG00000064341	ND1	-0.126521717	0.028045232
ENSMUSG00000060935	Tmem263	-0.126395509	6.44E-19
ENSMUSG00000034345	Gtf2h5	-0.125819812	1.04E-10
ENSMUSG00000031799	Tpm4	-0.125437973	0.006838564
ENSMUSG00000019173	Rab5c	-0.125362258	3.24E-08
ENSMUSG00000010651	Acaa1b	-0.125235935	8.85E-13
ENSMUSG00000069835	Sat2	-0.125091567	0.043161784
ENSMUSG00000069662	Marcks	-0.125034542	1.75E-15
ENSMUSG00000024668	Sdhaf2	-0.124663143	7.73E-08
ENSMUSG00000026317	Cln8	-0.124300336	6.82E-12
ENSMUSG00000024613	Tcof1	-0.1235487	7.16E-06
ENSMUSG00000033916	Chmp2a	-0.123149482	0.0009401
ENSMUSG00000026767	Mindy3	-0.123110743	3.14E-05
ENSMUSG00000027809	Etfdh	-0.122923543	4.75E-05
ENSMUSG00000065990	Aurkaip1	-0.122832998	2.40E-05
ENSMUSG00000030278	Cidec	-0.122671628	1.28E-07
ENSMUSG00000042569	Dhrs7b	-0.122659249	4.43E-07
ENSMUSG00000020087	Tysnd1	-0.122513667	0.015577625
ENSMUSG00000025652	Tmem89	-0.122344076	0.000104076
ENSMUSG00000067288	Rps28	-0.122215804	0.033438689
ENSMUSG00000029198	Grpel1	-0.122015891	2.10E-09
ENSMUSG00000055435	Maf	-0.121458841	1.27E-12
ENSMUSG00000028636	Ppcs	-0.12137559	0.000706544
ENSMUSG00000015755	Tab2	-0.121078339	1.79E-06
ENSMUSG00000004530	Coro1c	-0.121050339	7.64E-21
ENSMUSG00000037331	Larp1	-0.121046482	3.75E-11
ENSMUSG00000037470	Uggt1	-0.120943014	0.000533909
ENSMUSG00000015461	Atf6b	-0.120870615	7.15E-05
ENSMUSG00000031877	Ces2g	-0.1204385	1.21E-141
ENSMUSG00000037692	Ahdc1	-0.120152185	0.000354105
ENSMUSG00000028337	Coro2a	-0.119942544	2.04E-13
ENSMUSG00000002102	Psmc3	-0.119701946	0.002678155
ENSMUSG00000024968	Rcor2	-0.11965409	0.036570332
ENSMUSG00000010376	Nedd8	-0.119603962	2.57E-05
ENSMUSG00000075704	Txnrd2	-0.1192402	0.006032896
ENSMUSG00000000001	Gnai3	-0.118976826	0.007755676
ENSMUSG00000061411	Nol4l	-0.118810485	0.000434132
ENSMUSG00000054161	Fam83e	-0.118549343	1.16E-28
ENSMUSG00000005981	Trap1	-0.118319577	0.027247136
ENSMUSG00000044469	Tnfaip8l1	-0.11831531	1.50E-05
ENSMUSG00000060992	Copz1	-0.118182736	0.002269788
ENSMUSG00000001525	Tubb5	-0.117840177	3.68E-09
ENSMUSG00000029535	Triap1	-0.117632372	0.004892759
ENSMUSG00000040746	Rnf167	-0.117511285	3.48E-11
ENSMUSG00000026275	Ppp1r7	-0.117497472	0.000626073
ENSMUSG00000037197	Rbm17	-0.117484308	0.012536388
ENSMUSG00000031214	Ophn1	-0.116914851	0.009317429
ENSMUSG00000051730	Mettl5	-0.116687428	1.90E-05
ENSMUSG00000001627	Ifrd1	-0.116519735	7.53E-11
ENSMUSG00000029136	Rbks	-0.115820563	0.000271351
ENSMUSG00000031391	L1cam	-0.115495204	7.91E-12
ENSMUSG00000067367	Lyar	-0.115281463	9.14E-09
ENSMUSG00000053886	Sh2d4a	-0.115207136	0.011137244
ENSMUSG00000034403	Pja1	-0.115102137	2.64E-07
ENSMUSG00000018740	Slc25a35	-0.114965988	1.30E-05
ENSMUSG00000058351	Uqcc5	-0.114789339	0.004119802
ENSMUSG00000035699	Slc51a	-0.114770454	1.76E-59
ENSMUSG00000038116	Phf20	-0.114693565	0.012154123
ENSMUSG00000003233	Dvl3	-0.114681466	0.044533113
ENSMUSG00000021969	Zdhhc20	-0.114536264	1.21E-09
ENSMUSG00000035559	Mpv17l2	-0.114357736	0.000814668
ENSMUSG00000031392	Irak1	-0.114307098	0.012161377
ENSMUSG00000001847	Rac1	-0.113687158	4.47E-15
ENSMUSG00000042719	Naa25	-0.11368303	1.44E-07
ENSMUSG00000029063	Nadk	-0.113669547	0.007115351
ENSMUSG00000010607	Pigyl	-0.113645725	6.04E-07
ENSMUSG00000031601	Cnot7	-0.113554631	0.000413512
ENSMUSG00000022723	Crybg3	-0.11338915	0.000498526
ENSMUSG00000047638	Nr1h4	-0.112893923	0.005000842
ENSMUSG00000031906	Smpd3	-0.112754348	1.06E-11
ENSMUSG00000062300	Nectin2	-0.112752552	0.00033336
ENSMUSG00000024867	Pip5k1b	-0.112659314	0.000248211
ENSMUSG00000041278	Ttc1	-0.112576007	0.041926597
ENSMUSG00000027808	Serp1	-0.111675992	0.000500317
ENSMUSG00000029378	Areg	-0.111628922	0.000584974
ENSMUSG00000028799	Zfp362	-0.111360411	0.026659763
ENSMUSG00000030754	Copb1	-0.111299288	9.07E-15
ENSMUSG00000031059	Ndufb11	-0.111264744	5.44E-08
ENSMUSG00000027349	Fam98b	-0.111140397	0.000465022
ENSMUSG00000001440	Kpnb1	-0.111004803	0.000224619
ENSMUSG00000026078	Pdcl3	-0.11076309	0.000358273
ENSMUSG00000041133	Smc1a	-0.110701037	1.69E-08
ENSMUSG00000017615	Tnfaip1	-0.110619492	0.027753045
ENSMUSG00000028333	Anp32b	-0.110410937	1.52E-09
ENSMUSG00000013997	Nit1	-0.110287515	0.038076999
ENSMUSG00000018166	Erbb3	-0.11015018	8.20E-13
ENSMUSG00000041313	Slc7a1	-0.109854739	0.001925584
ENSMUSG00000045312	Lhfpl2	-0.109695873	5.42E-17
ENSMUSG00000032604	Qars	-0.109614875	1.36E-12
ENSMUSG00000020386	Sar1b	-0.109254052	2.47E-16

ENSMUSG00000027472	Pdrg1	0.162403322	0.000692529
ENSMUSG00000016833	Mrps18c	0.162395511	0.000809269
ENSMUSG00000040459	Arglu1	0.162316712	9.87E-06
ENSMUSG00000031540	Kat6a	0.162308219	0.000657803
ENSMUSG00000089824	Rbm12	0.162223225	0.02841654
ENSMUSG00000069793	Slfn9	0.161607372	0.001670766
ENSMUSG00000023393	Slc17a9	0.161426685	0.000544034
ENSMUSG00000020803	Txndc17	0.161204278	0.002180427
ENSMUSG00000036810	Cnep1r1	0.161079146	2.37E-18
ENSMUSG00000029290	Zfp326	0.160989867	0.01855676
ENSMUSG00000026525	Opn3	0.160654473	9.93E-05
ENSMUSG00000034211	Mrps17	0.160612752	0.004349782
ENSMUSG00000032582	Rbm6	0.160350415	1.21E-05
ENSMUSG00000031818	Cox4i1	0.1602155	5.91E-11
ENSMUSG00000041827	Oasl1	0.160169337	3.56E-18
ENSMUSG00000121496	Ube2d-ps	0.160113222	1.95E-05
ENSMUSG00000047728	Ly6g2	0.160027238	0.005495235
ENSMUSG00000019978	Epb41l2	0.160022968	5.56E-10
ENSMUSG00000025979	Mob4	0.159934855	2.95E-05
ENSMUSG00000026675	Hsd17b7	0.159933572	0.000657318
ENSMUSG00000037608	Bclaf1	0.15960506	3.71E-08
ENSMUSG00000025287	Acot9	0.159353119	1.22E-05
ENSMUSG00000060512	0610040J01R	0.159307394	0.000997231
ENSMUSG00000045665	Mfsd5	0.15903864	0.023962331
ENSMUSG00000032080	Apoa4	0.158820204	1.08E-93
ENSMUSG00000073481	Mtarc2	0.158699832	9.37E-163
ENSMUSG00000030780	Rusf1	0.158621356	0.032271422
ENSMUSG00000048249	Crebrf	0.158225722	0.001209363
ENSMUSG00000027286	Lrrc57	0.158209391	6.92E-07
ENSMUSG00000003299	Mrpl4	0.158031305	2.88E-07
ENSMUSG00000060147	Serpinb6a	0.158027339	0.002761641
ENSMUSG00000019370	Calm3	0.157928152	0.010434852
ENSMUSG00000041241	Mul1	0.157288193	4.85E-13
ENSMUSG00000019818	Cd164	0.156928675	6.04E-10
ENSMUSG00000027177	Hipk3	0.156922032	0.002492325
ENSMUSG00000027367	Stard7	0.15655683	0.049769829
ENSMUSG00000061313	Ddhd2	0.156513627	1.86E-12
ENSMUSG00000024740	Ddb1	0.156319101	1.22E-06
ENSMUSG00000003955	Fam162a	0.156256761	2.46E-15
ENSMUSG00000032737	Inppl1	0.155940188	0.000311144
ENSMUSG00000048154	Kmt2d	0.155475043	0.020988512
ENSMUSG00000028901	Gmeb1	0.155229195	0.030644703
ENSMUSG00000020074	Ccar1	0.155108413	4.54E-08
ENSMUSG00000024942	Capn1	0.154959302	0.025146021
ENSMUSG00000021809	Nudt13	0.154853148	5.37E-05
ENSMUSG00000032727	Mier3	0.154153833	0.002513061
ENSMUSG00000032612	Usp4	0.154076741	0.000634374
ENSMUSG00000110803	Gm20275	0.153911646	0.02085123
ENSMUSG00000025085	Ablim1	0.153788933	0.004398787
ENSMUSG00000030677	Kif22	0.153756411	0.002125223
ENSMUSG00000049287	Iba57	0.153519195	0.000371729
ENSMUSG00000029322	Plac8	0.153368798	5.14E-33
ENSMUSG00000020982	Nemf	0.153232274	0.034162871
ENSMUSG00000009863	Sdhb	0.153211236	5.40E-32
ENSMUSG00000009079	Ewsr1	0.153061218	1.61E-07
ENSMUSG00000038708	Golga4	0.152999495	1.71E-05
ENSMUSG00000030980	Knop1	0.152948948	7.71E-05
ENSMUSG00000050711	Scg2	0.15280775	0.005961378
ENSMUSG00000076431	Sox4	0.15277681	3.39E-15
ENSMUSG00000060743	H3f3a	0.152758299	0.012550795
ENSMUSG00000039347	Atp6v0e2	0.152681283	2.00E-07
ENSMUSG00000024754	Cemip2	0.151568501	9.41E-05
ENSMUSG00000036959	Bcorl1	0.15143564	0.000579926
ENSMUSG00000004846	Plod3	0.151108403	0.042233477
ENSMUSG00000061859	Patj	0.150841621	1.11E-05
ENSMUSG00000034867	Ankrd27	0.150791728	0.020820407
ENSMUSG00000028868	Wasf2	0.150776582	3.26E-09
ENSMUSG00000019797	Mtres1	0.150775914	9.72E-05
ENSMUSG00000024480	Ap3s1	0.150372849	0.001627625
ENSMUSG00000034880	Mrpl34	0.150273907	7.30E-15
ENSMUSG00000022094	Slc39a14	0.150261052	3.48E-11
ENSMUSG00000031886	Ces2e	0.150259948	1.40E-24
ENSMUSG00000055239	Kcmf1	0.150246073	0.000156065
ENSMUSG00000024589	Nedd4l	0.149931574	1.58E-10
ENSMUSG00000020889	Nr1d1	0.149688705	4.96E-05
ENSMUSG00000030704	Rab6a	0.14951135	7.72E-13
ENSMUSG00000107681	4930528H21	0.149138363	0.002775874
ENSMUSG00000049606	Zfp644	0.149077051	0.00045216
ENSMUSG00000013643	Lypd8	0.149056185	2.88E-11
ENSMUSG00000003099	Ppp5c	0.148919672	0.000509931
ENSMUSG00000019795	Pcmt1	0.148784805	4.82E-07
ENSMUSG00000049791	Fzd4	0.148757537	3.35E-06
ENSMUSG00000037674	Rfx7	0.148643611	0.000412556
ENSMUSG00000061518	Cox5b	0.148570469	8.43E-15
ENSMUSG00000014294	Ndufa2	0.148560069	3.46E-08
ENSMUSG00000032212	Sltm	0.148467687	7.16E-12
ENSMUSG00000029401	Rilpl2	0.148403795	1.37E-06
ENSMUSG00000027356	Fermt1	0.148389968	1.43E-05
ENSMUSG00000074578	Zfas1	0.14790581	0.000334922
ENSMUSG00000045594	Glb1	0.147870063	4.87E-08
ENSMUSG00000018334	Ksr1	0.147857657	0.000118806
ENSMUSG00000011958	Bnip2	0.147826755	0.021077511
ENSMUSG00000009013	Dynll1	0.147795494	0.000930772
ENSMUSG00000056383	AI987944	0.147650421	0.000882417
ENSMUSG00000019809	Pex3	0.147521272	1.22E-10
ENSMUSG00000063787	Chchd1	0.146920046	2.93E-05
ENSMUSG00000021709	Erbin	0.146844884	7.56E-06
ENSMUSG00000026927	Entr1	0.146672275	0.008734464
ENSMUSG00000024254	Abcg8	0.146484705	0.003399773
ENSMUSG00000044950	Pwwp2a	0.146417207	0.005727498
ENSMUSG00000031545	Gpat4	0.146018444	0.000181809
ENSMUSG00000039879	Heca	0.145485535	5.97E-10
ENSMUSG00000062234	Gak	0.145451426	0.000474525
ENSMUSG00000031803	B3gnt3	0.145358218	8.31E-13
ENSMUSG00000032340	Neo1	0.145163737	1.91E-07
ENSMUSG00000022175	Lrp10	0.144536883	5.02E-05
ENSMUSG00000018858	Mrpl58	0.144098903	3.01E-09
ENSMUSG00000109336	Samd4b	0.143778301	1.36E-07
ENSMUSG00000020098	Pcbd1	0.143764928	0.045115873
ENSMUSG00000029199	Lias	0.143410038	1.32E-08
ENSMUSG00000022205	Sub1	0.143124739	0.000502241
ENSMUSG00000022793	B4galt4	0.143114443	9.94E-10
ENSMUSG00000028684	Urod	0.142817513	9.52E-08
ENSMUSG00000032047	Acat1	0.142203653	1.86E-52
ENSMUSG00000005510	Ndufs3	0.142185863	1.51E-05
ENSMUSG00000042377	Fam83g	0.142130254	1.29E-06
ENSMUSG00000047371	Zfp768	0.142079994	0.002892207
ENSMUSG00000019054	Fis1	0.142058508	2.61E-05
ENSMUSG00000036473	Tbc1d24	0.14201229	8.95E-08
ENSMUSG00000033059	Pygb	0.141920933	0.001504114
ENSMUSG00000002748	Baz1b	0.141642463	3.98E-07
ENSMUSG00000022710	Usp7	0.141263605	7.40E-06

ENSMUSG00000034744	Nagk	-0.119980848	0.016050128
ENSMUSG00000040822	1700123O20	-0.119976768	0.001268452
ENSMUSG00000031537	Ikbkb	-0.119728899	0.000385907
ENSMUSG00000047989	Ino80c	-0.119204617	0.005856738
ENSMUSG00000009079	Ewsr1	-0.119203171	1.61E-07
ENSMUSG00000011257	Pabpc4	-0.119173512	3.25E-10
ENSMUSG00000006378	Gcat	-0.119064001	1.79E-25
ENSMUSG00000063882	Uqcrh	-0.118775296	5.82E-14
ENSMUSG00000041079	Rwdd2b	-0.118675292	0.000234264
ENSMUSG00000026767	Mindy3	-0.118624935	3.14E-05
ENSMUSG00000033379	Atp6v0b	-0.118150288	8.60E-11
ENSMUSG00000023259	Slc26a6	-0.117912679	2.23E-19
ENSMUSG00000006705	Pknox1	-0.117804183	0.002271463
ENSMUSG00000032330	Cox7a2	-0.117795434	5.98E-11
ENSMUSG00000029098	Acox3	-0.117300279	0.005836197
ENSMUSG00000039131	Gipc2	-0.117256575	2.49E-05
ENSMUSG00000008090	Fgfrl1	-0.117239613	1.91E-08
ENSMUSG00000040795	Iqcc	-0.117217258	0.017718245
ENSMUSG00000017801	Mlx	-0.116761706	2.03E-10
ENSMUSG00000020184	Mdm2	-0.116587942	1.35E-06
ENSMUSG00000050891	Tatdn1	-0.116012288	0.049500279
ENSMUSG00000004356	Utp20	-0.115987152	0.000279803
ENSMUSG00000027642	Rpn2	-0.115849986	2.62E-07
ENSMUSG00000015461	Atf6b	-0.115663675	7.15E-05
ENSMUSG00000033916	Chmp2a	-0.115266277	0.0009401
ENSMUSG00000086841	2410006H16	-0.114839072	0.001499301
ENSMUSG00000026254	Eif4e2	-0.114490207	1.33E-06
ENSMUSG00000104623	Gm6394	-0.114244897	0.001546549
ENSMUSG00000037740	Mrps26	-0.114062367	0.002335977
ENSMUSG00000031299	Pdha1	-0.113952824	2.95E-05
ENSMUSG00000032763	Ilvbl	-0.11390619	0.005636538
ENSMUSG00000081051	Gm15427	-0.113634418	0.005867786
ENSMUSG00000037904	Ankrd9	-0.113472333	0.027573307
ENSMUSG00000111966	Gm5781	-0.113445238	6.94E-05
ENSMUSG00000021048	Mthfd1	-0.113229224	9.26E-05
ENSMUSG00000027367	Stard7	-0.112695808	0.049769829
ENSMUSG00000029722	Agfg2	-0.112642425	0.008168715
ENSMUSG00000025512	Chid1	-0.112590894	1.84E-07
ENSMUSG00000089847	Timm10b	-0.112448392	0.000182727
ENSMUSG00000015937	Macroh2a1	-0.112075304	0.004329772
ENSMUSG00000028282	Casp8ap2	-0.111908519	0.001765034
ENSMUSG00000031601	Cnot7	-0.111720287	0.000413512
ENSMUSG00000032051	Fdx1	-0.111664664	5.68E-12
ENSMUSG00000063275	Hacd1	-0.111312917	0.00040136
ENSMUSG00000026078	Pdcl3	-0.111292188	0.000358273
ENSMUSG00000024953	Prdx5	-0.111179024	1.16E-28
ENSMUSG00000035852	Misp	-0.111085343	0.030909149
ENSMUSG00000028382	Ptbp3	-0.110955985	2.54E-10
ENSMUSG00000028392	Bspry	-0.11084773	7.67E-14
ENSMUSG00000029247	Paics	-0.110721196	0.011182021
ENSMUSG00000026087	Mrpl30	-0.110616412	0.005908101
ENSMUSG00000039531	Zup1	-0.110507208	0.009380488
ENSMUSG00000023349	Clec4n	-0.110258137	0.001097884
ENSMUSG00000032673	Prorsd1	-0.110199352	0.003129031
ENSMUSG00000002767	Mrpl2	-0.110154372	0.005032346
ENSMUSG00000026927	Entr1	-0.110065444	0.008734464
ENSMUSG00000069792	Wfdc17	-0.110039377	8.32E-06
ENSMUSG00000025981	Coq10b	-0.109948741	0.037733281
ENSMUSG00000025394	Epcam	-0.109909049	1.57E-14
ENSMUSG00000021241	Isca2	-0.109843893	1.51E-07
ENSMUSG00000056724	Nbeal2	-0.109790391	0.000199188
ENSMUSG00000028729	Ebna1bp2	-0.109567935	0.003057355
ENSMUSG00000045312	Lhfpl2	-0.109080548	5.42E-17
ENSMUSG00000013523	Bcas1	-0.109004885	1.18E-19
ENSMUSG00000017188	Coa3	-0.108815257	2.33E-08
ENSMUSG00000025178	Pi4k2a	-0.108361244	0.000492092
ENSMUSG00000025958	Creb1	-0.108207937	1.03E-06
ENSMUSG00000024180	Pgap6	-0.108101749	4.85E-11
ENSMUSG00000014177	Tvp23b	-0.107403825	0.000385632
ENSMUSG00000029203	Ube2k	-0.107369892	0.022615484
ENSMUSG00000034473	Sec22a	-0.106962353	2.44E-05
ENSMUSG00000028070	Naxe	-0.10683728	0.00042001
ENSMUSG00000006390	Elovl1	-0.106781199	4.14E-08
ENSMUSG00000031818	Cox4i1	-0.106753242	5.91E-11
ENSMUSG00000035764	Fbxo45	-0.106233105	0.002282152
ENSMUSG00000022956	Atp5o	-0.105865567	9.09E-12
ENSMUSG00000030281	Il17rc	-0.105728313	7.16E-09
ENSMUSG00000058624	Gda	-0.105609502	4.49E-13
ENSMUSG00000021180	Rps6ka5	-0.105230934	0.004120191
ENSMUSG00000034112	Atp2c2	-0.105109704	2.05E-05
ENSMUSG00000040046	Tph1	-0.105030977	4.25E-05
ENSMUSG00000065990	Aurkaip1	-0.10493815	2.40E-05
ENSMUSG00000037926	Ssh2	-0.104933296	0.000414242
ENSMUSG00000041577	Prelp	-0.104708434	0.034196222
ENSMUSG00000026887	Mrrf	-0.104399025	0.003310363
ENSMUSG00000044881	Coa4	-0.10395498	0.000506125
ENSMUSG00000029076	Sdf4	-0.103600184	3.03E-09
ENSMUSG00000022223	Sdr39u1	-0.10339966	0.000242749
ENSMUSG00000031605	Klhl2	-0.10304241	0.008322076
ENSMUSG00000054277	Arfgap3	-0.102852558	2.87E-05
ENSMUSG00000058076	Sdhc	-0.102166815	1.88E-22
ENSMUSG00000028599	Tnfrsf1b	-0.102115098	3.21E-05
ENSMUSG00000019822	Smpd2	-0.102111816	0.006475576
ENSMUSG00000073639	Rab18	-0.101932983	0.004754158
ENSMUSG00000028416	Bag1	-0.101902215	8.64E-05
ENSMUSG00000016510	Mtif3	-0.101746539	2.39E-10
ENSMUSG00000042349	Ikbke	-0.101375008	2.74E-13
ENSMUSG00000048186	Bend7	-0.101360456	5.39E-49
ENSMUSG00000041845	Rhod	-0.101295856	4.64E-10
ENSMUSG00000001105	Ift20	-0.10109267	1.71E-10
ENSMUSG00000024661	Fth1	-0.100832823	2.60E-83
ENSMUSG00000010097	Nxf1	-0.100832541	0.024420475
ENSMUSG00000024754	Cemip2	-0.100799113	9.41E-05
ENSMUSG00000022339	Ebag9	-0.100732222	0.040771107
ENSMUSG00000046807	Lrrc75b	-0.100154362	0.000550758
ENSMUSG00000051730	Mettl5	-0.10006267	1.90E-05
ENSMUSG00000040706	Agmat	-0.099927341	4.41E-32
ENSMUSG00000031097	Tnni2	-0.099509582	0.011036514
ENSMUSG00000078619	Smarcd2	-0.099301793	0.002697978
ENSMUSG00000001794	Capns1	-0.099266491	0.000262041
ENSMUSG00000045817	Zfp36l2	-0.099231441	0.029801642
ENSMUSG00000030754	Copb1	-0.09914277	9.07E-15
ENSMUSG00000029319	Coq2	-0.098939625	0.000390206
ENSMUSG00000011884	Gltp	-0.098919609	2.06E-18
ENSMUSG00000052738	Suclg1	-0.09871838	5.48E-12
ENSMUSG00000003778	Brd8	-0.098523014	0.00310048
ENSMUSG00000056531	Ccdc18	-0.09852176	0.004966899
ENSMUSG00000023967	Mrps18a	-0.098414524	0.029214919
ENSMUSG00000074578	Zfas1	-0.098350112	0.000334922
ENSMUSG00000024131	Slc3a1	-0.098210833	0.014875225
ENSMUSG00000037608	Bclaf1	-0.098201564	3.71E-08
ENSMUSG00000031197	Vbp1	-0.098177488	0.04345514
ENSMUSG00000020283	Pex13	0.181011816	3.61E-05
ENSMUSG00000095079	Igha	0.180839366	7.83E-06
ENSMUSG00000035770	Dync1li2	0.180782919	0.027674198
ENSMUSG00000020721	Helz	0.180775214	0.000968606
ENSMUSG00000024242	Map4k3	0.180686316	0.000568118
ENSMUSG00000020072	Pbld2	0.179940454	2.42E-24
ENSMUSG00000023845	Lnpep	0.179885816	0.000391058
ENSMUSG00000026860	Sh3glb2	0.179874414	0.037997814
ENSMUSG00000024944	Arl2	0.179778533	0.017726467
ENSMUSG00000026313	Hdac4	0.179528286	0.00401388
ENSMUSG00000047921	Trappc9	0.179513878	0.036136295
ENSMUSG00000045636	Mtus1	0.17944684	0.010166753
ENSMUSG00000000355	Mcts1	0.179424858	4.16E-05
ENSMUSG00000030935	Acsm3	0.179295109	0.04217825
ENSMUSG00000045962	Wnk1	0.179016089	3.38E-09
ENSMUSG00000095193	Gm20939	0.178191843	7.54E-08
ENSMUSG00000026031	Cflar	0.17782192	0.047840264
ENSMUSG00000029249	Rest	0.17771281	2.01E-06
ENSMUSG00000027285	Haus2	0.177694466	0.000337974
ENSMUSG00000030865	Chp2	0.177443587	7.62E-24
ENSMUSG00000026627	Pacc1	0.177324815	0.000775647
ENSMUSG00000036580	Spart	0.177174268	5.89E-09
ENSMUSG00000020608	Smc6	0.177057174	5.38E-06
ENSMUSG00000042323	Pbrm1	0.17703124	0.000223559
ENSMUSG00000031778	Cx3cl1	0.176962086	2.18E-05
ENSMUSG00000019969	Psen1	0.176930621	0.002222725
ENSMUSG00000048170	Mcmbp	0.176847925	3.05E-08
ENSMUSG00000022507	Hapstr1	0.176650113	0.044348297
ENSMUSG00000002015	Bcap31	0.176473345	4.11E-06
ENSMUSG00000044792	Isca1	0.176057142	2.77E-06
ENSMUSG00000060261	Gtf2i	0.176038008	8.06E-05
ENSMUSG00000027447	Cst3	0.175237516	2.32E-05
ENSMUSG00000035637	Grhpr	0.175076351	1.93E-14
ENSMUSG00000024764	Naa40	0.174970794	0.032048707
ENSMUSG00000018171	Vmp1	0.174962731	0.000209098
ENSMUSG00000090115	Usp49	0.174716338	0.004258887
ENSMUSG00000038366	Lasp1	0.174154193	1.60E-05
ENSMUSG00000089824	Rbm12	0.173378455	0.02841654
ENSMUSG00000036504	Phpt1	0.17335865	0.005297176
ENSMUSG00000047728	Ly6g2	0.173346572	0.005495235
ENSMUSG00000028329	Xpa	0.173075912	0.0391561
ENSMUSG00000027315	Spint1	0.173025938	0.000584911
ENSMUSG00000024639	Gnaq	0.172890096	2.53E-09
ENSMUSG00000026003	Acadl	0.172507282	2.27E-16
ENSMUSG00000000125	Wnt3	0.172265903	1.17E-05
ENSMUSG00000036606	Plxnb2	0.172087468	2.19E-11
ENSMUSG00000057143	Trim12c	0.171950288	9.35E-05
ENSMUSG00000020788	Atp2a3	0.171722845	2.28E-09
ENSMUSG00000073402	Gm8909	0.171598016	1.79E-10
ENSMUSG00000021565	Slc6a19	0.171429727	6.26E-09
ENSMUSG00000028470	Hint2	0.171358752	6.37E-11
ENSMUSG00000030739	Myh14	0.171206355	6.86E-11
ENSMUSG00000025528	2010106E10	0.170987034	6.92E-09
ENSMUSG00000060376	Bckdha	0.170928569	0.00129736
ENSMUSG00000038604	Ripor1	0.169992078	0.036251442
ENSMUSG00000026353	Ubxn4	0.169704943	0.022591247
ENSMUSG00000022752	Tomm70a	0.169498094	7.18E-29
ENSMUSG00000027457	Snph	0.169476356	0.000248451
ENSMUSG00000032679	Cd59a	0.168957426	0.048790081
ENSMUSG00000061474	Mrps36	0.168702311	2.21E-05
ENSMUSG00000027475	Kif3b	0.168474543	0.034301603
ENSMUSG00000035203	Epn1	0.168332274	0.028016101
ENSMUSG00000026640	Plxna2	0.168191944	0.000446959
ENSMUSG00000042284	Itga1	0.168120591	0.002844741
ENSMUSG00000029290	Zfp326	0.168006625	0.01855676
ENSMUSG00000030774	Pak1	0.16796997	7.49E-08
ENSMUSG00000022744	Cldnd1	0.167580582	1.32E-16
ENSMUSG00000041997	Tlk1	0.167465353	3.79E-06
ENSMUSG00000024782	Ak3	0.167390272	1.57E-06
ENSMUSG00000044950	Pwwp2a	0.167298199	0.005727498
ENSMUSG00000030787	Lyve1	0.167075588	0.020104265
ENSMUSG00000040865	Ino80d	0.166845748	2.90E-06
ENSMUSG00000038506	Dcun1d2	0.166808378	0.016392718
ENSMUSG00000005698	Ctcf	0.166625044	0.000327504
ENSMUSG00000026189	Pecr	0.16610173	0.003069527
ENSMUSG00000021662	Arhgef28	0.166046161	0.000187268
ENSMUSG00000030213	Atf7ip	0.166037784	0.015247343
ENSMUSG00000020009	Ifngr1	0.166005441	0.024872783
ENSMUSG00000017837	Nkiras2	0.165782556	0.001746391
ENSMUSG00000036352	Ubac1	0.165640283	9.12E-11
ENSMUSG00000002778	Kdelr1	0.165475421	2.73E-10
ENSMUSG00000034867	Ankrd27	0.164889216	0.020820407
ENSMUSG00000031158	Timm17b	0.164730716	1.66E-06
ENSMUSG00000051495	Irf2bp2	0.16461798	3.21E-08
ENSMUSG00000021884	Hacl1	0.164502132	0.001978751
ENSMUSG00000045775	Slc16a5	0.164345477	0.007886387
ENSMUSG00000029455	Aldh2	0.164191751	8.80E-08
ENSMUSG00000038013	Wipf2	0.163902534	0.000782135
ENSMUSG00000006299	Aamp	0.163833442	0.005762682
ENSMUSG00000048251	Bcl11b	0.163648306	8.52E-17
ENSMUSG00000109336	Samd4b	0.163587451	1.36E-07
ENSMUSG00000017485	Top2b	0.163249215	0.011929613
ENSMUSG00000020359	Phykpl	0.163200025	0.000264189
ENSMUSG00000029726	Mepce	0.163063181	0.036183086
ENSMUSG00000032290	Ptpn9	0.163022158	3.84E-08
ENSMUSG00000020877	Scrn2	0.162924969	0.008654496
ENSMUSG00000022114	Spry2	0.162647655	0.000586423
ENSMUSG00000029330	Cds1	0.162196092	0.000498281
ENSMUSG00000026991	Pkp4	0.161426114	3.46E-07
ENSMUSG00000028255	Clca1	0.161298997	2.01E-11
ENSMUSG00000031822	Gse1	0.161096818	1.19E-05
ENSMUSG00000109644	0610005C13	0.161067822	5.87E-14
ENSMUSG00000037470	Uggt1	0.160895837	0.000533909
ENSMUSG00000053841	Txlna	0.160715219	8.60E-05
ENSMUSG00000033949	Trim36	0.16047646	0.00888391
ENSMUSG00000001552	Jup	0.16043991	1.38E-14
ENSMUSG00000024974	Smc3	0.160077845	7.15E-13
ENSMUSG00000029145	Eif2b4	0.159999686	0.012467134
ENSMUSG00000007837	Prrg2	0.159956857	6.09E-09
ENSMUSG00000087433	Gm14167	0.159790415	0.015674104
ENSMUSG00000001280	Sp1	0.158909636	3.30E-11
ENSMUSG00000087165	2010001A14	0.15884190	3.45E-14
ENSMUSG00000036188	Ankmy2	0.158563133	0.001303135
ENSMUSG00000030822	Prr14	0.158274845	0.006212389
ENSMUSG00000049106	Dcaf5	0.158050785	0.019448091
ENSMUSG00000029185	Fam114a1	0.156984933	4.30E-05
ENSMUSG00000004054	Map3k11	0.156808108	0.036177317
ENSMUSG00000031835	Mbtps1	0.156386916	0.012964896
ENSMUSG00000021236	Entpd5	0.156326617	4.66E-07
ENSMUSG00000032532	Cck	0.155471755	3.70E-150
ENSMUSG00000058173	Smco4	0.155041292	0.021524132
ENSMUSG00000049090	Ptgr3	0.15478928	0.000369346
ENSMUSG00000037022	Mmaa	-0.108978412	3.43E-09
ENSMUSG00000032083	Apoa1	-0.108954302	2.28E-11
ENSMUSG00000020592	Sdc1	-0.108791537	4.25E-15
ENSMUSG00000043733	Ptpn11	-0.108624188	0.027753045
ENSMUSG00000038398	Upf3a	-0.108568846	0.014132997
ENSMUSG00000075467	Dnlz	-0.1083404	0.008394779
ENSMUSG00000026754	Golga1	-0.1082496	1.29E-13
ENSMUSG00000020864	Ankrd40	-0.107997561	0.000826805
ENSMUSG00000015002	Efr3a	-0.107931091	2.33E-14
ENSMUSG00000038485	Socs7	-0.107773647	0.00026345
ENSMUSG00000027009	Itga4	-0.107517885	0.021452274
ENSMUSG00000032547	Ryk	-0.10741763	3.70E-17
ENSMUSG00000028745	Capzb	-0.107378896	7.55E-10
ENSMUSG00000042644	Itpr3	-0.107347629	0.000111671
ENSMUSG00000028029	Aimp1	-0.106955638	0.023165591
ENSMUSG00000028466	Creb3	-0.106787873	3.25E-12
ENSMUSG00000071415	Rpl23	-0.106655593	0.000435042
ENSMUSG00000050957	Insl6	-0.106388388	3.51E-17
ENSMUSG00000001156	Mxd1	-0.106218735	0.000326425
ENSMUSG00000044533	Rps2	-0.106087001	0.013315496
ENSMUSG00000036450	Hif1an	-0.106068982	0.007579951
ENSMUSG00000042699	Dhx9	-0.105983918	0.000326848
ENSMUSG00000029776	Hibadh	-0.105819008	7.77E-08
ENSMUSG00000029269	Sult1b1	-0.105621702	1.45E-25
ENSMUSG00000044465	Fhip1b	-0.105581884	0.000149937
ENSMUSG00000043061	Tmem18	-0.105403947	0.00010538
ENSMUSG00000042272	Sestd1	-0.105123199	0.004143347
ENSMUSG00000023277	Twf2	-0.104230492	0.002820919
ENSMUSG00000001918	Slc1a5	-0.104020701	2.95E-25
ENSMUSG00000027835	Pdcd10	-0.104019976	3.71E-09
ENSMUSG00000033022	Cdo1	-0.103604499	1.70E-05
ENSMUSG00000027088	Phospho2	-0.103592546	6.76E-06
ENSMUSG00000062456	Rpl9-ps6	-0.103405752	0.001927044
ENSMUSG00000027800	Tm4sf1	-0.103231146	0.008460808
ENSMUSG00000039145	Camk1d	-0.103165049	1.71E-20
ENSMUSG00000030605	Mfge8	-0.103095244	0.002308611
ENSMUSG00000030472	Ceacam18	-0.103024896	2.34E-46
ENSMUSG00000017631	Abr	-0.102829516	0.025960939
ENSMUSG00000031831	Dnaaf1	-0.102686722	0.039951206
ENSMUSG00000028434	Epb41l4b	-0.102320321	0.002760537
ENSMUSG00000025016	Tm9sf3	-0.102068987	1.80E-10
ENSMUSG00000022564	Grina	-0.101948924	0.027905181
ENSMUSG00000002477	Snrpd1	-0.101847561	0.013633496
ENSMUSG00000022283	Pabpc1	-0.101782553	3.11E-16
ENSMUSG00000061559	Skic8	-0.101613921	0.001862014
ENSMUSG00000020952	Scfd1	-0.101440845	0.000353135
ENSMUSG00000028809	Srrm1	-0.101382157	8.21E-05
ENSMUSG00000028657	Ppt1	-0.101006427	3.86E-11
ENSMUSG00000021707	Dhfr	-0.100708493	1.89E-10
ENSMUSG00000085795	Zfp703	-0.100687549	2.04E-06
ENSMUSG00000033400	Agl	-0.100213477	0.002485741
ENSMUSG00000033845	Mrpl15	-0.100203482	0.000550197
ENSMUSG00000004880	Lbr	-0.100016418	0.000264466
ENSMUSG00000057342	Sphk2	-0.099339421	2.14E-06
ENSMUSG00000008206	Cers4	-0.099078264	0.026280643
ENSMUSG00000038780	Smurf1	-0.099065956	0.022628139
ENSMUSG00000043067	Dpy19l1	-0.098907999	7.46E-05
ENSMUSG00000072946	Ptgr2	-0.098613319	3.59E-12
ENSMUSG00000037905	Bri3bp	-0.098605358	1.08E-16
ENSMUSG00000022257	Laptm4b	-0.09848198	3.20E-10
ENSMUSG00000036078	Sigmar1	-0.0977252	0.009537598
ENSMUSG00000078578	Ube2d3	-0.097522691	0.00030282
ENSMUSG00000025402	Nab2	-0.097243134	0.028064487
ENSMUSG00000030741	Spns1	-0.096890634	0.017283857
ENSMUSG00000025981	Coq10b	-0.096687438	0.037733281
ENSMUSG00000062619	2310039H08	-0.09666966	4.18E-05
ENSMUSG00000024805	Pcgf5	-0.0966672	9.92E-05
ENSMUSG00000050103	Agmo	-0.096574839	8.23E-09
ENSMUSG00000029723	Spacdr	-0.096290418	0.000453139
ENSMUSG00000022555	Dgat1	-0.096239401	5.75E-07
ENSMUSG00000006398	Cdc20	-0.096221373	1.27E-05
ENSMUSG00000042306	S100a14	-0.096115252	2.73E-07
ENSMUSG00000040570	Rundc3b	-0.09594097	0.043818152
ENSMUSG00000035726	Supt16	-0.09585591	4.41E-05
ENSMUSG00000041992	Rapgef5	-0.095394694	0.000651647
ENSMUSG00000025510	Cd151	-0.095330754	0.000130874
ENSMUSG00000096001	Gpr15lg	-0.094912931	1.97E-13
ENSMUSG00000037373	Ctbp1	-0.094865543	3.50E-05
ENSMUSG00000047989	Ino80c	-0.094794413	0.005856738
ENSMUSG00000039844	Rapgef1	-0.094729859	0.000657803
ENSMUSG00000031995	St14	-0.094664094	0.000734215
ENSMUSG00000028010	Gar1	-0.094044899	1.02E-05
ENSMUSG00000020850	Prpf8	-0.093793796	0.000140648
ENSMUSG00000079363	Gbp4	-0.093790714	0.000657803
ENSMUSG00000085683	Tmem238l	-0.093604738	2.32E-07
ENSMUSG00000032504	Pdcd6ip	-0.09360329	1.50E-08
ENSMUSG00000032908	Sgpp2	-0.093566354	0.021396842
ENSMUSG00000055943	Emc7	-0.093072294	0.01138942
ENSMUSG00000019320	Noxo1	-0.093035921	2.20E-11
ENSMUSG00000026192	Atic	-0.093028757	0.01177418
ENSMUSG00000028847	Trappc3	-0.092851147	0.024835519
ENSMUSG00000008730	Hipk1	-0.092543255	1.03E-08
ENSMUSG00000032185	Carm1	-0.091785188	0.007445477
ENSMUSG00000021822	Plau	-0.0914948	0.001590207
ENSMUSG00000001034	Mapk7	-0.091467753	0.015258993
ENSMUSG00000036188	Ankmy2	-0.09116992	0.001303135
ENSMUSG00000028410	Dnaja1	-0.091061923	0.007233847
ENSMUSG00000021022	Ppp2r3c	-0.090871034	0.000519166
ENSMUSG00000049932	H2ax	-0.090779051	0.02594076
ENSMUSG00000022584	Ly6c2	-0.090701714	6.26E-08
ENSMUSG00000083992	Gm11478	-0.09049699	0.044235433
ENSMUSG00000028194	Ddah1	-0.0903863	3.41E-78
ENSMUSG00000062312	Erbb2	-0.090357654	0.001394536
ENSMUSG00000001666	Ddt	-0.090191481	5.88E-08
ENSMUSG00000018042	Cyb5r3	-0.089937344	8.34E-40
ENSMUSG00000020859	Spag9	-0.08957895	0.002908508
ENSMUSG00000067149	Jchain	-0.089551028	4.39E-14
ENSMUSG00000022848	Slc49a4	-0.089288691	0.041164597
ENSMUSG00000037820	Tgm2	-0.089184406	1.76E-32
ENSMUSG00000062397	Zfp706	-0.088703435	0.002298702
ENSMUSG00000024998	Plce1	-0.088578741	9.87E-14
ENSMUSG00000031712	Il15	-0.088275239	5.27E-07
ENSMUSG00000043154	Ppp2r3a	-0.088181701	4.62E-11
ENSMUSG00000003458	Ncstn	-0.087994382	0.011223877
ENSMUSG00000073436	Eme2	-0.087851434	0.002862161
ENSMUSG00000027624	Epb41l1	-0.08752697	2.56E-12
ENSMUSG00000059263	Usp47	-0.08721246	0.002223072
ENSMUSG00000006676	Usp19	-0.086925345	0.04447606
ENSMUSG00000031897	Psmb10	-0.086894277	2.06E-16
ENSMUSG00000032009	Sesn3	-0.086521385	1.54E-06
ENSMUSG00000032051	Fdx1	-0.086289975	5.68E-12
ENSMUSG00000054200	Ffar4	-0.086145961	0.000477061
ENSMUSG00000039016	Timm8b	0.141021639	6.64E-10
ENSMUSG00000075232	Amd1	0.141009776	0.002123934
ENSMUSG00000038301	Snx10	0.140411452	5.47E-09
ENSMUSG00000035506	Slc12a8	0.139734255	0.000807097
ENSMUSG00000035877	Zhx3	0.139730014	0.007327965
ENSMUSG00000032198	Dock6	0.139687027	0.016708449
ENSMUSG00000054823	Nsd3	0.139665445	0.002368838
ENSMUSG00000094651	Gal3st2	0.139628886	5.75E-20
ENSMUSG00000020238	Ncln	0.139604126	0.039703869
ENSMUSG00000027782	Kpna4	0.139567095	0.007506413
ENSMUSG00000040084	Bub1b	0.139374709	0.010898782
ENSMUSG00000036985	Zdhhc9	0.139334517	0.006650662
ENSMUSG00000030534	Vps33b	0.139163034	0.001944919
ENSMUSG00000040112	Mrps35	0.138454456	6.24E-06
ENSMUSG00000028964	Park7	0.137905224	0.000456189
ENSMUSG00000010067	Rassf1	0.137858674	0.029311492
ENSMUSG00000026663	Atf6	0.137843743	1.58E-06
ENSMUSG00000027993	Trim2	0.137723342	3.33E-18
ENSMUSG00000020611	Gna13	0.137654066	0.001757206
ENSMUSG00000027761	Aadac	0.137402735	8.18E-29
ENSMUSG00000022330	Osr2	0.137344916	4.46E-66
ENSMUSG00000024908	Ppp6r3	0.137323452	0.042758846
ENSMUSG00000000088	Cox5a	0.137166469	2.56E-26
ENSMUSG00000079470	Utp14b	0.136975806	0.003315543
ENSMUSG00000020818	Mfsd11	0.136950227	0.046910347
ENSMUSG00000016510	Mtif3	0.136751392	2.39E-10
ENSMUSG00000032112	Trappc4	0.136471013	7.91E-09
ENSMUSG00000029330	Cds1	0.136342864	0.000498281
ENSMUSG00000032449	Slc25a36	0.136323046	5.73E-11
ENSMUSG00000109644	0610005C13	0.136202741	5.87E-14
ENSMUSG00000029191	Rfc1	0.136125242	8.01E-15
ENSMUSG00000008348	Ubc	0.136123089	0.007368425
ENSMUSG00000045394	Epcam	0.135730466	1.57E-14
ENSMUSG00000018921	Pelp1	0.13569095	0.000308515
ENSMUSG00000020758	Itgb4	0.135212663	3.20E-10
ENSMUSG00000032551	1110059G10	0.135013981	0.000428276
ENSMUSG00000028673	Fuca1	0.134453146	1.47E-06
ENSMUSG00000017843	Ppp2r5c	0.134326362	1.72E-08
ENSMUSG00000031608	Galnt7	0.134253286	0.000101095
ENSMUSG00000030763	Lcmt1	0.134214122	2.60E-10
ENSMUSG00000028156	Eif4e	0.133475298	0.000543331
ENSMUSG00000086784	Isoc2a	0.133424977	1.16E-10
ENSMUSG00000054428	Atpif1	0.133403344	2.11E-10
ENSMUSG00000032615	Nt5m	0.132863591	0.001662336
ENSMUSG00000030263	Irag2	0.132640726	0.003545798
ENSMUSG00000092564	BC051226	0.132586209	0.008244926
ENSMUSG00000021640	Naip1	0.132576218	2.56E-12
ENSMUSG00000015961	Adss	0.13241048	6.81E-11
ENSMUSG00000003068	Stk11	0.132385862	0.027507502
ENSMUSG00000004568	Arhgef18	0.1320051	0.006349284
ENSMUSG00000019188	H13	0.131945202	0.000987671
ENSMUSG00000075486	Commd6	0.13189136	5.82E-09
ENSMUSG00000041757	Plekha6	0.131814612	2.96E-13
ENSMUSG00000021696	Elovl7	0.13156172	1.87E-12
ENSMUSG00000061718	Ppp1r1b	0.131398109	0.001765034
ENSMUSG00000027997	Casp6	0.131343382	3.10E-82
ENSMUSG00000004815	Dgkq	0.131120943	1.37E-06
ENSMUSG00000063882	Uqcrh	0.13098023	5.82E-14
ENSMUSG00000025195	Dnmbp	0.13088739	2.77E-05
ENSMUSG00000022360	Atad2	0.130278448	0.000185654
ENSMUSG00000030530	Furin	0.129846148	0.001944818
ENSMUSG00000028677	Rnf220	0.129478828	0.007092492
ENSMUSG00000051864	Tbc1d22a	0.129398539	0.00755563
ENSMUSG00000079020	Slc45a4	0.129258352	0.002210559
ENSMUSG00000027715	Ccna2	0.129112266	1.79E-10
ENSMUSG00000026189	Pecr	0.128945322	0.003069527
ENSMUSG00000036639	Nudt1	0.12874423	0.001347641
ENSMUSG00000032301	Psma4	0.128178012	0.000194134
ENSMUSG00000049550	Clip1	0.12809659	4.45E-05
ENSMUSG00000029090	Adgra3	0.128022704	0.029061948
ENSMUSG00000096145	Vkorc1	0.127775295	2.25E-15
ENSMUSG00000037533	Rapgef6	0.127652331	0.04217825
ENSMUSG00000078429	Ctdsp2	0.127033349	7.43E-22
ENSMUSG00000046447	Camk2n1	0.126860162	0.006145375
ENSMUSG00000020910	Adprm	0.126714727	0.000234352
ENSMUSG00000002679	Med6	0.12635656	0.032619666
ENSMUSG00000040365	Trim41	0.126337992	0.000332109
ENSMUSG00000021866	Anxa11	0.12627779	3.67E-05
ENSMUSG00000021327	Zkscan3	0.126175976	6.90E-05
ENSMUSG00000015672	Mrpl32	0.126017448	1.29E-06
ENSMUSG00000071054	Safb	0.125642159	0.003153346
ENSMUSG00000031314	Taf1	0.125498471	0.006488642
ENSMUSG00000036873	2410004B18	0.125436199	0.020113341
ENSMUSG00000042444	Mindy2	0.12541738	0.000324791
ENSMUSG00000038009	Dnajc22	0.125237641	0.000142323
ENSMUSG00000027406	Idh3b	0.125036788	6.30E-15
ENSMUSG00000022847	Thpo	0.124802723	0.002805469
ENSMUSG00000034928	Rnf44	0.12474874	0.000360812
ENSMUSG00000050608	Micos10	0.124380234	1.37E-12
ENSMUSG00000020075	Ddx21	0.124298824	1.54E-06
ENSMUSG00000034245	Hdac11	0.124281087	1.89E-05
ENSMUSG00000038658	Ric1	0.12425048	0.030584039
ENSMUSG00000038518	Jarid2	0.124048675	5.91E-10
ENSMUSG00000094840	Muc3a	0.123952421	1.93E-05
ENSMUSG00000055024	Ep300	0.123936807	5.79E-05
ENSMUSG00000026605	Cenpf	0.123915364	2.84E-07
ENSMUSG00000022841	Ap2m1	0.123900126	0.000975717
ENSMUSG00000021957	Tkt	0.123808498	3.72E-07
ENSMUSG00000008763	Man1a2	0.123793988	0.035305034
ENSMUSG00000038880	Mrps34	0.123641507	9.87E-09
ENSMUSG00000034744	Nagk	0.123458413	0.016050128
ENSMUSG00000038538	Ubn2	0.123350061	0.000187236
ENSMUSG00000063358	Mapk1	0.123248465	5.15E-06
ENSMUSG00000022680	Pdxdc1	0.123133387	0.004589407
ENSMUSG00000020329	Polrmt	0.12294179	0.036190166
ENSMUSG00000059534	Uqcr10	0.12293902	6.56E-14
ENSMUSG00000024566	Atp9b	0.122912186	0.005349933
ENSMUSG00000075602	Ly6a	0.122894197	4.87E-05
ENSMUSG00000037434	Slc30a1	0.122600289	3.15E-20
ENSMUSG00000046516	Cox17	0.122499383	9.27E-07
ENSMUSG00000118894	Gm24424	0.122462787	0.019336282
ENSMUSG00000023022	Lima1	0.122280708	0.00209822
ENSMUSG00000030935	Acsm3	0.122147188	0.04217825
ENSMUSG00000026090	Cracdl	0.121949415	0.00046851
ENSMUSG00000023845	Lnpep	0.121947487	0.000391058
ENSMUSG00000038717	Atp5l	0.121857799	1.88E-09
ENSMUSG00000020516	Rps6kb1	0.12177400	0.004630545
ENSMUSG00000020459	Mtif2	0.121729526	2.64E-06
ENSMUSG00000013663	Pten	0.121651769	0.03978833
ENSMUSG00000027650	Tti1	0.12156548	4.06E-05
ENSMUSG00000042605	Atxn2	0.121476063	0.001150677
ENSMUSG00000023967	Mrps18a	0.121407724	0.029214919

ENSMUSG00000005510	Ndufs3	-0.097719406	1.51E-05
ENSMUSG00000018923	Med11	-0.097652505	0.000735635
ENSMUSG00000029192	Tbc1d14	-0.097615477	0.0234826
ENSMUSG00000029486	Mrpl1	-0.097343537	3.95E-07
ENSMUSG00000021607	Mrpl36	-0.097300887	5.97E-05
ENSMUSG00000015812	Gnrh1	-0.097254711	0.014262929
ENSMUSG00000026933	Camsap1	-0.09695368	0.036763495
ENSMUSG00000034247	Plekhm1	-0.096876545	0.007364716
ENSMUSG00000021733	Slc4a7	-0.096709986	2.48E-16
ENSMUSG00000049422	Chchd10	-0.096113304	2.18E-34
ENSMUSG00000025439	Clns1a	-0.096058669	4.80E-05
ENSMUSG00000055436	Srsf11	-0.095912879	0.011034641
ENSMUSG00000000776	Polr3d	-0.095871844	2.80E-05
ENSMUSG00000074195	Clca4b	-0.095494908	2.05E-12
ENSMUSG00000044018	Mrpl50	-0.095452759	0.005297974
ENSMUSG00000036810	Cnep1r1	-0.095422652	2.37E-18
ENSMUSG00000039047	Pigk	-0.095382028	0.027252551
ENSMUSG00000051671	Coa6	-0.095291051	0.000473174
ENSMUSG00000020115	Tbk1	-0.095062902	0.007256752
ENSMUSG00000021377	Dek	-0.094941872	0.000146036
ENSMUSG00000067847	Romo1	-0.094842071	0.000122598
ENSMUSG00000031600	Vps37a	-0.094683197	0.001546549
ENSMUSG00000022136	Dnajc3	-0.094626588	0.000116484
ENSMUSG00000021211	Akr1c12	-0.094446681	1.69E-45
ENSMUSG00000020180	Snrpd3	-0.094281446	0.009838726
ENSMUSG00000019978	Epb41l2	-0.094142701	5.56E-10
ENSMUSG00000022108	Itm2b	-0.093662482	4.36E-12
ENSMUSG00000002108	Nr1h3	-0.093459671	0.000142457
ENSMUSG00000006932	Ctnnb1	-0.09340949	2.44E-15
ENSMUSG00000032423	Syncrip	-0.092914494	9.25E-08
ENSMUSG00000021576	Pdcd6	-0.092890788	9.76E-11
ENSMUSG00000024384	Iws1	-0.092754435	0.001546016
ENSMUSG00000071078	Nr2c2ap	-0.092600443	0.033620746
ENSMUSG00000021756	Il6st	-0.092564654	0.008551288
ENSMUSG00000024493	Lars	-0.092419527	0.001765034
ENSMUSG00000029407	Uso1	-0.092193611	3.80E-11
ENSMUSG00000047766	Lrrc49	-0.091904969	0.00249024
ENSMUSG00000037533	Rapgef6	-0.091747376	0.04217825
ENSMUSG00000021550	2210016F16I	-0.091404158	3.53E-06
ENSMUSG00000037075	Rnf139	-0.091388539	2.00E-05
ENSMUSG00000076934	Iglv1	-0.091387449	0.001877963
ENSMUSG00000024228	Nudt12	-0.090797726	6.88E-05
ENSMUSG00000006676	Usp19	-0.090706041	0.04447606
ENSMUSG00000073002	Vamp5	-0.090637653	6.61E-15
ENSMUSG00000067288	Rps28	-0.090520084	0.033438689
ENSMUSG00000070544	Top1	-0.09039899	4.92E-11
ENSMUSG00000032959	Pebp1	-0.090333507	0.011620851
ENSMUSG00000031533	Mrps31	-0.090308238	2.04E-06
ENSMUSG00000028563	Tm2d1	-0.089907646	0.002397492
ENSMUSG00000045128	Rpl18a	-0.089641866	1.14E-06
ENSMUSG00000032405	Pias1	-0.089199379	0.002243948
ENSMUSG00000023079	Gtf2ird1	-0.088886099	0.022794622
ENSMUSG00000018927	Ccl6	-0.088792096	0.003641836
ENSMUSG00000003435	Supt5	-0.088748105	0.038999003
ENSMUSG00000018659	Pnpo	-0.088693807	0.000316536
ENSMUSG00000057666	Gapdh	-0.088548445	1.46E-16
ENSMUSG00000092564	BC051226	-0.088516754	0.008244926
ENSMUSG00000022394	L3mbtl2	-0.088466052	0.044291234
ENSMUSG00000028228	Cpne3	-0.088455853	0.040412823
ENSMUSG00000028790	Khdrbs1	-0.088445889	0.008698054
ENSMUSG00000049580	Tsku	-0.08830201	0.004377194
ENSMUSG00000031320	Rps4x	-0.088243789	1.63E-09
ENSMUSG00000024997	Prdx3	-0.088216998	7.89E-06
ENSMUSG00000021840	Mapk1ip1l	-0.088161831	3.88E-13
ENSMUSG00000038342	Mlxip	-0.087787888	0.036211525
ENSMUSG00000008958	Vps72	-0.087702355	0.000527685
ENSMUSG00000018999	Slc35b4	-0.087543924	0.001727824
ENSMUSG00000001542	Ell2	-0.087316668	3.03E-06
ENSMUSG00000062382	Ftl1-ps2	-0.086775582	0.000348845
ENSMUSG00000044715	Gskip	-0.086474864	0.001192276
ENSMUSG00000024038	Ndufv3	-0.086232908	7.44E-10
ENSMUSG00000041625	Ggact	-0.085905627	2.96E-06
ENSMUSG00000021709	Erbin	-0.085763004	7.56E-06
ENSMUSG00000001918	Slc1a5	-0.085456541	2.95E-25
ENSMUSG00000039640	Mrpl12	-0.08545244	6.01E-19
ENSMUSG00000038170	Pde4dip	-0.085318764	0.000727724
ENSMUSG00000026499	Acbd3	-0.085116008	1.02E-05
ENSMUSG00000002741	Ykt6	-0.085096142	5.03E-06
ENSMUSG00000034282	Evpl	-0.085075828	8.52E-19
ENSMUSG00000020309	Chac2	-0.084993206	2.87E-05
ENSMUSG00000069456	Rdh16	-0.084680838	1.48E-06
ENSMUSG00000044165	Bcl2l15	-0.084455021	3.98E-11
ENSMUSG00000027346	Gpcpd1	-0.08398287	2.35E-37
ENSMUSG00000022024	Sugt1	-0.083980046	8.85E-05
ENSMUSG00000061207	Stk19	-0.083837794	0.020311805
ENSMUSG00000027479	Mapre1	-0.083797754	3.98E-06
ENSMUSG00000027163	Commd9	-0.083716632	0.028139588
ENSMUSG00000022337	Emc2	-0.08327067	2.38E-08
ENSMUSG00000009030	Pdcl	-0.083144279	0.012849896
ENSMUSG00000052396	Mogat2	-0.083138885	4.00E-07
ENSMUSG00000020863	Luc7l3	-0.082875562	7.33E-07
ENSMUSG00000021606	Ndufs6	-0.082171302	2.38E-09
ENSMUSG00000025362	Rps26	-0.082080217	2.22E-10
ENSMUSG00000079494	Nat8f5	-0.082060666	2.08E-24
ENSMUSG00000022408	Fam83f	-0.082017937	2.90E-05
ENSMUSG00000040562	Gstm2	-0.081901232	1.03E-80
ENSMUSG00000002580	Mien1	-0.081721966	4.30E-10
ENSMUSG00000025510	Cd151	-0.08120344	0.000130874
ENSMUSG00000028955	Vamp3	-0.081101616	0.000446012
ENSMUSG00000020634	Ubxn2a	-0.080863959	0.021533336
ENSMUSG00000041313	Slc7a1	-0.080856142	0.001925584
ENSMUSG00000043962	Thrap3	-0.080684949	4.59E-07
ENSMUSG00000028179	Cth	-0.080436865	1.31E-08
ENSMUSG00000084883	Ccdc85c	-0.08000335	0.046449504
ENSMUSG00000028964	Park7	-0.079990813	0.000456189
ENSMUSG00000021610	Clptm1l	-0.079778557	0.04962789
ENSMUSG00000039842	Mcph1	-0.079732359	0.000121982
ENSMUSG00000022475	Hdac7	-0.079647238	3.79E-06
ENSMUSG00000030615	Tmem126a	-0.079494713	2.34E-08
ENSMUSG00000074466	Gm15417	-0.079452438	0.000132731
ENSMUSG00000090035	Galnt4	-0.079189061	1.08E-07
ENSMUSG00000026504	Sdccag8	-0.079162883	1.38E-06
ENSMUSG00000079427	Mthfsl	-0.079076015	0.000291267
ENSMUSG00000026565	Pou2f1	-0.078917606	3.35E-06
ENSMUSG00000010376	Nedd8	-0.078444732	2.57E-05
ENSMUSG00000031634	Ufsp2	-0.078321764	0.009307836
ENSMUSG00000067713	Prkag1	-0.078232561	0.000505261
ENSMUSG00000025016	Tm9sf3	-0.077852846	1.80E-10
ENSMUSG00000027177	Hipk3	-0.07770621	0.002492325
ENSMUSG00000014867	Surf4	-0.077620485	2.16E-09
ENSMUSG00000078578	Ube2d3	-0.077562432	0.00030282
ENSMUSG00000036197	Gxylt1	-0.077433565	0.000833849

ENSMUSG00000042500	Ago4	0.154342593	0.014262929
ENSMUSG00000031662	Snx20	0.154234262	2.58E-13
ENSMUSG00000032402	Smad3	0.153854757	0.000400374
ENSMUSG00000031608	Galnt7	0.153682623	0.000101095
ENSMUSG00000009545	Kcnq1	0.153151702	1.87E-11
ENSMUSG00000022564	Grina	0.153074762	0.027905181
ENSMUSG00000058351	Uqcc5	0.152893393	0.004119802
ENSMUSG00000034928	Rnf44	0.152820055	0.000360812
ENSMUSG00000062646	Ganc	0.152765053	5.51E-05
ENSMUSG00000024644	Cndp2	0.152551408	9.91E-14
ENSMUSG00000089704	Galnt2	0.152541197	0.017850123
ENSMUSG00000019564	Arid3a	0.152447724	0.042971842
ENSMUSG00000034757	Tmub2	0.152196043	0.026608554
ENSMUSG00000024991	Eif3a	0.151918152	6.81E-11
ENSMUSG00000042608	Stk40	0.151827912	3.22E-05
ENSMUSG00000029201	Ugdh	0.151410242	4.35E-46
ENSMUSG00000028854	Slc9a1	0.151234444	0.002881981
ENSMUSG00000038127	Ccdc50	0.151189483	0.001485771
ENSMUSG00000027487	Cdk5rap1	0.151141	0.012287356
ENSMUSG00000031913	Vps4a	0.150944322	0.035299273
ENSMUSG00000008206	Cers4	0.150872502	0.026280643
ENSMUSG00000032612	Usp4	0.150865251	0.000634374
ENSMUSG00000090266	Mettl23	0.150809088	0.016355893
ENSMUSG00000051154	Commd3	0.150644715	0.000548183
ENSMUSG00000068040	Tm9sf4	0.150397119	0.000601369
ENSMUSG00000032293	Ireb2	0.150267748	6.00E-09
ENSMUSG00000028684	Urod	0.150117686	9.52E-08
ENSMUSG00000027363	Usp8	0.149159264	0.032834201
ENSMUSG00000003534	Ddr1	0.14906359	1.15E-07
ENSMUSG00000022568	Scrib	0.148827821	0.001731794
ENSMUSG00000006005	Tpr	0.148815496	1.26E-08
ENSMUSG00000026773	Pfkfb3	0.14876918	0.001717134
ENSMUSG00000009376	Met	0.148724658	0.021765742
ENSMUSG00000030579	Tyrobp	0.148495402	0.00039658
ENSMUSG00000053293	Pom121	0.148221208	5.59E-05
ENSMUSG00000048118	Arid4a	0.148188287	2.32E-09
ENSMUSG00000020423	Btg2	0.148181301	0.002679488
ENSMUSG00000030655	Smg1	0.147798981	0.030362379
ENSMUSG00000026956	Uap1l1	0.147685279	1.64E-13
ENSMUSG00000022629	Kif21a	0.147217908	2.83E-10
ENSMUSG00000029759	Pon3	0.145272714	1.23E-06
ENSMUSG00000042719	Naa25	0.144715708	1.44E-07
ENSMUSG00000017707	Serinc3	0.144423208	9.83E-06
ENSMUSG00000062691	Cebpzos	0.144202839	7.91E-12
ENSMUSG00000042198	Chchd7	0.144101568	0.000274522
ENSMUSG00000028809	Srrm1	0.144022573	8.21E-05
ENSMUSG00000005506	Celf1	0.143824041	4.60E-06
ENSMUSG00000062373	Tmem65	0.1435678	8.02E-08
ENSMUSG00000029196	Tada2b	0.143326386	0.010668865
ENSMUSG00000017291	Taok1	0.143060698	9.43E-07
ENSMUSG00000049076	Acap2	0.143027256	0.018915017
ENSMUSG00000120992	D17H6S56E-	0.142844847	7.00E-14
ENSMUSG00000068267	Cenpb	0.14274831	0.013102438
ENSMUSG00000019863	Qrsl1	0.142668519	0.003535704
ENSMUSG00000022443	Myh9	0.142445979	8.03E-08
ENSMUSG00000019188	H13	0.142433202	0.000987671
ENSMUSG00000041028	Ghitm	0.142247044	1.64E-11
ENSMUSG00000029152	Ociad1	0.141335232	7.01E-08
ENSMUSG00000024290	Rock1	0.14124656	2.25E-06
ENSMUSG00000031770	Herpud1	0.141066635	0.000352936
ENSMUSG00000003464	Pex19	0.141059244	0.00019839
ENSMUSG00000021072	Tmx1	0.140973134	2.82E-06
ENSMUSG00000027420	Bfsp1	0.140891883	0.00205409
ENSMUSG00000026974	Zmynd19	0.140511581	0.017452383
ENSMUSG00000036572	Upf3b	0.139785011	2.82E-06
ENSMUSG00000028378	Ptgr1	0.139634545	1.83E-09
ENSMUSG00000032899	Styk1	0.13921841	0.001398362
ENSMUSG00000019210	Atp6v1e1	0.139171274	3.19E-10
ENSMUSG00000004952	Rasa4	0.139104706	0.030199975
ENSMUSG00000032220	Myo1e	0.138979148	9.11E-09
ENSMUSG00000039461	Tcta	0.138936533	4.71E-07
ENSMUSG00000025226	Fbxl15	0.138901415	1.19E-05
ENSMUSG00000034201	Gas2l1	0.138894776	7.72E-06
ENSMUSG00000028062	Lamtor2	0.138774709	0.002333436
ENSMUSG00000020124	Usp15	0.138747636	0.001111645
ENSMUSG00000029135	Fosl2	0.138469677	2.00E-20
ENSMUSG00000037815	Ctnna1	0.138379829	2.52E-07
ENSMUSG00000029461	Fam168a	0.138215047	0.020154342
ENSMUSG00000009995	Tafazzin	0.13801248	1.47E-05
ENSMUSG00000043587	Pxylp1	0.137863529	0.016054813
ENSMUSG00000019738	Polr2i	0.137647482	0.013063757
ENSMUSG00000107002	Ncbp2as2	0.13756014	0.002692275
ENSMUSG00000031479	Vps36	0.137358412	0.008256463
ENSMUSG00000049401	Ogfr	0.137052503	0.020461029
ENSMUSG00000051768	Xrcc1	0.136995278	0.033771177
ENSMUSG00000111080	Gm20300	0.136959971	0.004892759
ENSMUSG00000046707	Csnk2a2	0.136549303	0.006157097
ENSMUSG00000036591	Arhgap21	0.136376622	0.041397351
ENSMUSG00000032714	Syde1	0.136209741	1.80E-05
ENSMUSG00000090935	Synj2bp	0.136119028	2.46E-05
ENSMUSG00000000958	Slc7a7	0.136106347	0.000645705
ENSMUSG00000053580	Tanc2	0.135932433	0.014942979
ENSMUSG00000022560	Slc52a2	0.135834134	0.013807886
ENSMUSG00000019861	Gopc	0.135716576	0.001761033
ENSMUSG00000012350	Ehf	0.135600569	1.86E-33
ENSMUSG00000028664	Ephb2	0.135235695	7.05E-05
ENSMUSG00000049489	Ccnq	0.135219462	0.009451458
ENSMUSG00000000308	Ckmt1	0.135136542	2.09E-57
ENSMUSG00000016757	Ttll12	0.13477173	0.001377849
ENSMUSG00000022696	Sidt1	0.13471069	5.92E-09
ENSMUSG00000029207	Apbb2	0.134595875	8.95E-06
ENSMUSG00000006010	Odr4	0.134048547	0.000816903
ENSMUSG00000041130	Zfp598	0.13389204	9.32E-05
ENSMUSG00000062312	Erbb2	0.133591451	0.001394536
ENSMUSG00000031029	Eif3f	0.133430614	1.35E-13
ENSMUSG00000032212	Sltm	0.133269993	7.16E-12
ENSMUSG00000044864	Ankrd50	0.133079913	0.012160781
ENSMUSG00000024695	Zfp91	0.132741377	0.048165505
ENSMUSG00000017631	Abr	0.132519881	0.025960939
ENSMUSG00000031921	Terf2	0.132160491	0.004874025
ENSMUSG00000052812	Atad2b	0.131937917	0.009122095
ENSMUSG00000020098	Pcbd1	0.130863715	0.045115873
ENSMUSG00000055024	Ep300	0.13062217	5.79E-05
ENSMUSG00000054611	Kdm2a	0.1306109	3.91E-06
ENSMUSG00000022668	Gtpbp8	0.13043084	0.000119674
ENSMUSG00000025417	Pip4k2c	0.130137661	2.33E-05
ENSMUSG00000022214	Dcaf11	0.129747897	0.000370682
ENSMUSG00000027589	Pcmtd2	0.129564586	0.000302619
ENSMUSG00000070972	Dnajc25	0.129285346	0.006435437
ENSMUSG00000099881	2810013P06	0.129276863	0.031298191
ENSMUSG00000038982	Bloc1s5	0.129135722	0.01436856
ENSMUSG00000041992	Rapgef5	0.128947264	0.000651647

ENSMUSG00000083837	Gm14427	-0.086066451	0.000994978
ENSMUSG00000020440	Arf5	-0.085887108	4.07E-29
ENSMUSG00000052337	Immt	-0.085557186	9.78E-25
ENSMUSG00000020358	Hnrnpab	-0.085447192	0.001345076
ENSMUSG00000058126	Tpm3-rs7	-0.085359695	0.009164354
ENSMUSG00000018923	Med11	-0.08516469	0.000735635
ENSMUSG00000032091	Tmprss4	-0.084447722	0.000171221
ENSMUSG00000041926	Rnpep	-0.084316605	0.000268604
ENSMUSG00000020099	Unc5b	-0.084277809	1.68E-09
ENSMUSG00000075595	Zfp652	-0.084054592	0.003787764
ENSMUSG00000057286	St6galnac2	-0.083896566	0.014086736
ENSMUSG00000028571	Cyp2j13	-0.083862848	5.21E-06
ENSMUSG00000025208	Mrpl43	-0.08385977	2.77E-05
ENSMUSG00000028635	Edn2	-0.08366338	1.10E-14
ENSMUSG00000020671	Rab10	-0.083564377	1.24E-07
ENSMUSG00000020330	Hmmr	-0.083335618	8.37E-05
ENSMUSG00000019179	Mdh2	-0.082968346	5.96E-92
ENSMUSG00000014301	Pam16	-0.082759357	1.93E-07
ENSMUSG00000047394	Odf3b	-0.082748999	9.79E-08
ENSMUSG00000003051	Elf3	-0.082525587	0.013686283
ENSMUSG00000053768	Chchd3	-0.082523447	9.25E-16
ENSMUSG00000030061	Uba3	-0.08249123	3.45E-05
ENSMUSG00000056162	Cndp1	-0.082419387	0.047670499
ENSMUSG00000004394	Tmed4	-0.082395537	6.19E-18
ENSMUSG00000053600	Zfp472	-0.082341585	0.011451514
ENSMUSG00000031662	Snx20	-0.082296514	2.58E-13
ENSMUSG00000030062	Rpn1	-0.082058594	3.45E-05
ENSMUSG00000024404	Riok3	-0.082002403	0.001218359
ENSMUSG00000029655	N4bp2l2	-0.081931439	2.68E-06
ENSMUSG00000032399	Rpl4	-0.081900762	4.55E-16
ENSMUSG00000040430	Pitpnc1	-0.081668742	0.004454409
ENSMUSG00000051495	Irf2bp2	-0.081550323	3.21E-08
ENSMUSG00000024165	Jpt2	-0.081331969	0.01628867
ENSMUSG00000004864	Mapk13	-0.080921531	0.006063852
ENSMUSG00000071180	Smim15	-0.080891064	2.04E-06
ENSMUSG00000026773	Pfkfb3	-0.080822942	0.001717134
ENSMUSG00000041126	H2az2	-0.080698295	0.000245425
ENSMUSG00000040505	Abcg5	-0.079865818	9.88E-12
ENSMUSG00000034342	Cbl	-0.079813505	0.00173483
ENSMUSG00000020532	Acaca	-0.079778312	1.71E-05
ENSMUSG00000058886	Deaf1	-0.079715591	0.031270982
ENSMUSG00000022747	St3gal6	-0.079714403	2.38E-11
ENSMUSG00000034932	Mrpl54	-0.079611156	3.36E-10
ENSMUSG00000038213	Tapbpl	-0.079560086	0.000189635
ENSMUSG00000022971	Ifnar2	-0.079328201	0.000229063
ENSMUSG00000029642	Polr1d	-0.079298216	0.000774023
ENSMUSG00000031246	Sh3bgrl	-0.07895552	4.43E-14
ENSMUSG00000019432	Ddx39b	-0.078895714	0.000785021
ENSMUSG00000007036	Abhd16a	-0.07887803	4.82E-06
ENSMUSG00000033416	Gucd1	-0.078853922	5.75E-07
ENSMUSG00000010277	Bltp2	-0.078659425	0.032442581
ENSMUSG00000039157	Eeig1	-0.078357782	0.004998145
ENSMUSG00000005674	Tomm40l	-0.078341229	0.000217454
ENSMUSG00000063694	Cycs	-0.078308174	3.64E-09
ENSMUSG00000048546	Tob2	-0.078303683	0.006877541
ENSMUSG00000032348	Gsta4	-0.078303062	1.11E-28
ENSMUSG00000027765	P2ry1	-0.07801923	0.012876154
ENSMUSG00000021180	Rps6ka5	-0.077957412	0.004120191
ENSMUSG00000057706	Mex3b	-0.077914745	4.15E-07
ENSMUSG00000031488	Rab11fip1	-0.077678957	1.33E-08
ENSMUSG00000029203	Ube2k	-0.077631134	0.022615484
ENSMUSG00000042363	Lgalsl	-0.077179961	4.47E-09
ENSMUSG00000020986	Sec23a	-0.07713566	4.23E-06
ENSMUSG00000030201	Lrp6	-0.077062059	1.61E-08
ENSMUSG00000006678	Pola1	-0.077053744	0.005994317
ENSMUSG00000025702	Marchf8	-0.076682855	7.12E-05
ENSMUSG00000027540	Ptpn1	-0.076590216	0.000700134
ENSMUSG00000020156	Pwwp3a	-0.076363567	0.000950705
ENSMUSG00000050271	Prag1	-0.076277648	0.006974247
ENSMUSG00000042207	Kdm5b	-0.076252392	0.02409627
ENSMUSG00000045248	Med26	-0.076140014	0.001395375
ENSMUSG00000026074	Map4k4	-0.075933078	6.07E-07
ENSMUSG00000053289	Ddx10	-0.075677913	0.001001706
ENSMUSG00000050912	Tmem123	-0.075640005	0.004567805
ENSMUSG00000052087	Rgs14	-0.075430459	4.18E-08
ENSMUSG00000023169	Slc38a1	-0.075377148	0.001492697
ENSMUSG00000079523	Tmsb10	-0.075275783	2.96E-05
ENSMUSG00000079111	Kdelr2	-0.07458729	0.000124785
ENSMUSG00000034471	Caskin2	-0.074541499	1.53E-07
ENSMUSG00000021981	Cab39l	-0.074512469	0.000675742
ENSMUSG00000044715	Gskip	-0.074209555	0.001192276
ENSMUSG00000051223	Bzw1	-0.074153882	0.043079913
ENSMUSG00000032562	Gnai2	-0.074049997	0.001209862
ENSMUSG00000054474	Thnsl2	-0.073905183	0.003879406
ENSMUSG00000028820	Sfpq	-0.07324055	1.42E-09
ENSMUSG00000029145	Eif2b4	-0.07323313	0.012467134
ENSMUSG00000074698	Csnk2a1	-0.07309359	1.19E-16
ENSMUSG00000031154	Otud5	-0.073046304	0.027736522
ENSMUSG00000034101	Ctnnd1	-0.072774787	1.59E-10
ENSMUSG00000031173	Otc	-0.072706554	6.14E-06
ENSMUSG00000058013	Septin11	-0.072554832	7.75E-06
ENSMUSG00000038936	Sccpdh	-0.072384204	0.000198862
ENSMUSG00000027111	Itga6	-0.072198288	0.007524523
ENSMUSG00000002475	Abhd3	-0.072183756	3.05E-05
ENSMUSG00000029455	Aldh2	-0.072092775	8.80E-08
ENSMUSG00000068921	Dap3	-0.072055801	0.000913741
ENSMUSG00000038580	Sct	-0.072025541	0.033838908
ENSMUSG00000005078	Jkamp	-0.071955015	0.004735836
ENSMUSG00000030286	Emc3	-0.071918737	0.010654996
ENSMUSG00000024521	Pmaip1	-0.07183005	0.000464015
ENSMUSG00000038387	Rras	-0.071466971	0.011325008
ENSMUSG00000053580	Tanc2	-0.071236941	0.014942979
ENSMUSG00000046668	Cxxc5	-0.071225414	0.000183961
ENSMUSG00000024899	Papss2	-0.071200245	8.85E-35
ENSMUSG00000031429	Psmd10	-0.071184575	0.000141134
ENSMUSG00000027330	Cdc25b	-0.071016359	0.002775596
ENSMUSG00000027546	Atp9a	-0.070553025	9.73E-07
ENSMUSG00000017778	Cox7c	-0.069823623	1.73E-10
ENSMUSG00000075419	Dolk	-0.069737769	0.047172472
ENSMUSG00000025173	Wdr45b	-0.069701985	0.027059533
ENSMUSG00000040703	Cyp2s1	-0.069700416	3.23E-05
ENSMUSG00000035493	Tgfbi	-0.069431959	1.13E-05
ENSMUSG00000034973	Dop1a	-0.069105966	0.011657083
ENSMUSG00000024953	Prdx5	-0.06878549	1.16E-28
ENSMUSG00000100714	2010308F09I	-0.068733651	7.74E-08
ENSMUSG00000019810	Fuca2	-0.068513708	1.88E-10
ENSMUSG00000074649	BC029722	-0.068469852	8.00E-07
ENSMUSG00000028459	Cd72	-0.068223776	0.015801549
ENSMUSG00000040359	Ufl1	-0.068220804	0.001061784
ENSMUSG00000104011	Gm32391	-0.068039526	0.036163918
ENSMUSG00000044167	Foxo1	-0.067572799	0.000111502
ENSMUSG00000073155	Stmp1	-0.067538547	1.87E-16

ENSMUSG00000005609	Ctr9	0.121129163	0.023972146
ENSMUSG00000025220	Oga	0.121004937	0.001836723
ENSMUSG00000042682	Selenok	0.12100272	0.020343029
ENSMUSG00000054611	Kdm2a	0.120888378	3.91E-06
ENSMUSG00000022751	Nit2	0.120833128	1.74E-09
ENSMUSG00000029335	Bmp3	0.120684857	1.81E-09
ENSMUSG00000029313	Aff1	0.120040945	3.40E-08
ENSMUSG00000067813	Xkr9	0.119870053	0.001082899
ENSMUSG00000019889	Ptprk	0.119803068	1.35E-07
ENSMUSG00000074305	Peak1	0.119693802	0.004234774
ENSMUSG00000012114	Med15	0.119515094	8.81E-05
ENSMUSG00000028266	Lmo4	0.119387364	1.98E-06
ENSMUSG00000042406	Atf4	0.119260688	0.003907827
ENSMUSG00000042492	Tbc1d10b	0.119244852	0.031622732
ENSMUSG00000032239	Rp9	0.119166867	0.049400636
ENSMUSG00000067071	Hes6	0.119146084	2.66E-05
ENSMUSG00000002379	Ndufa11	0.119078948	6.05E-11
ENSMUSG00000021477	Ctsl	0.119066686	1.12E-32
ENSMUSG00000050747	Trim15	0.119058286	1.25E-06
ENSMUSG00000004070	Hmox2	0.118705005	0.000829264
ENSMUSG00000029478	Ncor2	0.118609717	4.06E-05
ENSMUSG00000036073	Galt	0.118496536	0.020006738
ENSMUSG00000049436	Upk1b	0.118305344	3.28E-12
ENSMUSG00000001158	Snrnp27	0.118243777	4.44E-06
ENSMUSG00000020903	Stx8	0.118161517	0.019730174
ENSMUSG00000019710	Mrpl24	0.118069547	6.94E-06
ENSMUSG00000001891	Ugp2	0.117893807	0.001240041
ENSMUSG00000030214	Plbd1	0.11784646	0.004005103
ENSMUSG00000024985	Tcf7l2	0.117821176	8.63E-06
ENSMUSG00000033228	Scaf11	0.117641508	0.000657344
ENSMUSG00000058325	Dock1	0.117628773	1.28E-10
ENSMUSG00000028008	Asic5	0.117358204	0.009523928
ENSMUSG00000010358	Ifi35	0.117165849	1.73E-25
ENSMUSG00000042210	Abhd14a	0.117138703	0.000159987
ENSMUSG00000026260	Ndufa10	0.116574781	5.01E-16
ENSMUSG00000018585	Atox1	0.116512194	4.40E-07
ENSMUSG00000018509	Cenpv	0.116257164	8.51E-05
ENSMUSG00000000957	Mmp14	0.11613668	0.003730007
ENSMUSG00000034707	Gns	0.116072401	0.000919104
ENSMUSG00000063952	Brpf3	0.115998989	0.000170658
ENSMUSG00000007564	Ppp2r1a	0.115764424	0.010690033
ENSMUSG00000024621	Csf1r	0.11570526	0.00447197
ENSMUSG00000026319	Relch	0.1156212	0.048517154
ENSMUSG00000024799	Tm7sf2	0.115504852	2.18E-05
ENSMUSG00000047554	Tmem41b	0.115240846	3.89E-05
ENSMUSG00000028247	Coq3	0.115115187	0.002269371
ENSMUSG00000074093	Svip	0.114739642	1.06E-09
ENSMUSG00000046434	Hnrnpa1	0.114598674	3.20E-05
ENSMUSG00000022571	Pycrl	0.114416684	8.09E-11
ENSMUSG00000034640	Tiparp	0.11372985	8.23E-10
ENSMUSG00000018707	Dync1h1	0.113678205	8.90E-05
ENSMUSG00000056698	Elmod3	0.11317917	3.20E-05
ENSMUSG00000029229	Chic2	0.112998631	0.025136461
ENSMUSG00000028149	Rap1gds1	0.11278963	0.014625219
ENSMUSG00000091337	Eid1	0.112754531	0.015500911
ENSMUSG00000007034	Slc44a4	0.112653829	2.61E-38
ENSMUSG00000038949	Cnst	0.112628221	0.002387076
ENSMUSG00000004032	Gstm5	0.112512482	0.021524132
ENSMUSG00000050621	Rps27rt	0.112447164	0.000578538
ENSMUSG00000026896	Ifih1	0.112156008	1.62E-08
ENSMUSG00000031848	Lsm4	0.111925589	0.000550084
ENSMUSG00000029135	Fosl2	0.111758398	2.00E-20
ENSMUSG00000030884	Uqcrc2	0.111659479	2.59E-11
ENSMUSG00000028494	Plin2	0.11158064	3.62E-13
ENSMUSG00000025868	Higd2a	0.111563057	1.37E-06
ENSMUSG00000018076	Med13l	0.11143202	5.81E-11
ENSMUSG00000029291	Rufy3	0.11109417	0.031320364
ENSMUSG00000020153	Ndufs7	0.111071951	1.77E-09
ENSMUSG00000049751	Rpl36al	0.110923312	0.008609617
ENSMUSG00000003420	Fcgrt	0.11076317	7.71E-07
ENSMUSG00000026895	Ndufa8	0.110611182	1.84E-10
ENSMUSG00000029131	Dnajb6	0.110042924	0.007864052
ENSMUSG00000038335	Tsr1	0.109901284	3.62E-05
ENSMUSG00000031770	Herpud1	0.109851865	0.000352936
ENSMUSG00000027599	Armc1	0.109746228	0.012869605
ENSMUSG00000025466	Fuom	0.109726905	1.49E-15
ENSMUSG00000023827	Agpat4	0.109628284	3.00E-20
ENSMUSG00000020258	Glyctk	0.109569631	0.000299517
ENSMUSG00000075229	Mix23	0.109420851	1.70E-05
ENSMUSG00000031479	Vps36	0.109009559	0.008256463
ENSMUSG00000021585	Cast	0.108753999	2.30E-12
ENSMUSG00000057649	Brd9	0.10841523	0.00010538
ENSMUSG00000025404	R3hdm2	0.108218477	0.011041446
ENSMUSG00000015759	Cnih1	0.108174118	5.30E-06
ENSMUSG00000028896	Rcc1	0.10802806	1.02E-06
ENSMUSG00000022052	Ppp2r2a	0.107986995	2.31E-05
ENSMUSG00000015217	Hmgb3	0.107744755	0.016029763
ENSMUSG00000053286	Trmt1l	0.107704945	0.002944447
ENSMUSG00000030846	Tial1	0.107604476	0.000550221
ENSMUSG00000087165	2010001A14	0.107589373	3.45E-14
ENSMUSG00000032381	Ciao2a	0.107483391	2.15E-11
ENSMUSG00000030779	Rbbp6	0.107319071	4.09E-12
ENSMUSG00000052748	Swt1	0.107287128	2.78E-05
ENSMUSG00000113198	Gm6988	0.106985176	0.007850221
ENSMUSG00000022110	Sucla2	0.106971445	2.47E-14
ENSMUSG00000028082	Sh3d19	0.106715852	0.014335662
ENSMUSG00000009545	Kcnq1	0.106680272	1.87E-11
ENSMUSG00000035642	Aamdc	0.106602574	4.28E-15
ENSMUSG00000024048	Myl12a	0.106466612	0.025812037
ENSMUSG00000032540	Abhd5	0.106444803	6.86E-11
ENSMUSG00000016252	Atp5e	0.105891539	4.21E-13
ENSMUSG00000022184	Fbxo4	0.105614605	0.012733301
ENSMUSG00000027296	Itpka	0.10559456	0.009451871
ENSMUSG00000022561	Gpaa1	0.10556982	1.66E-09
ENSMUSG00000000308	Ckmt1	0.105552803	2.09E-57
ENSMUSG00000044600	Smim7	0.105300408	4.43E-07
ENSMUSG00000064345	ND2	0.105258338	0.000450124
ENSMUSG00000021408	Ripk1	0.104998206	0.019682395
ENSMUSG00000086712	Mexis	0.104863553	3.26E-47
ENSMUSG00000025326	Ube3a	0.104799289	0.012696823
ENSMUSG00000024099	Ndufv2	0.104797954	9.66E-19
ENSMUSG00000031556	Tm2d2	0.104713803	0.004759227
ENSMUSG00000039234	Sec24d	0.104690635	5.82E-21
ENSMUSG00000055114	Anxa13	0.104425924	1.74E-16
ENSMUSG00000033557	Fam20b	0.104416287	6.93E-05
ENSMUSG00000002222	Rmnd5a	0.104239948	0.000500317
ENSMUSG00000028479	Gne	0.104140592	0.014104411
ENSMUSG00000001739	Cldn15	0.104105032	9.23E-06
ENSMUSG00000021185	Dglucy	0.103990675	1.86E-54
ENSMUSG00000034109	Golim4	0.103826318	2.74E-06
ENSMUSG00000047030	Spata2	0.103763521	4.72E-05
ENSMUSG00000033114	Slc35d2	0.103667055	9.23E-09

ENSMUSG00000027746	Ufm1	-0.077384425	1.65E-07
ENSMUSG00000074794	Arrdc3	-0.077264382	7.20E-06
ENSMUSG00000026698	Pigc	-0.07702305	8.40E-06
ENSMUSG00000047496	Rnf152	-0.076857362	8.97E-21
ENSMUSG00000021877	Arf4	-0.07665896	5.65E-17
ENSMUSG00000018585	Atox1	-0.076543212	4.40E-07
ENSMUSG00000031725	Ces1f	-0.075939662	2.19E-36
ENSMUSG00000018481	Appbp2	-0.075481738	0.017726204
ENSMUSG00000026705	Klhl20	-0.074925042	0.032848588
ENSMUSG00000028706	Nsun4	-0.074856794	0.000129291
ENSMUSG00000035047	Kri1	-0.074381936	0.00051638
ENSMUSG00000025504	Eps8l2	-0.074114512	0.014917691
ENSMUSG00000120995	Gm51425	-0.073743309	0.000103279
ENSMUSG00000024847	Aip	-0.073590737	2.12E-08
ENSMUSG00000040722	Scamp5	-0.073576287	1.24E-27
ENSMUSG00000087590	Epb41l4aos	-0.073247404	0.031263462
ENSMUSG00000074259	Gramd2a	-0.07295673	0.00254277
ENSMUSG00000006456	Rbm14	-0.072945892	0.001527893
ENSMUSG00000039254	Pomt1	-0.072586925	0.034874097
ENSMUSG00000059923	Grb2	-0.072484048	0.045766349
ENSMUSG00000041278	Ttc1	-0.07239505	0.041926597
ENSMUSG00000029763	Exoc4	-0.072370868	1.58E-05
ENSMUSG00000051314	Ffar2	-0.07236675	4.97E-05
ENSMUSG00000000732	Icosl	-0.072141148	0.038753168
ENSMUSG00000027358	Bmp2	-0.071995226	1.06E-08
ENSMUSG00000032059	Alg9	-0.071529808	0.047208109
ENSMUSG00000018770	Atp5g3	-0.070885811	2.10E-18
ENSMUSG00000004285	Atp6v1f	-0.070424325	0.000158318
ENSMUSG00000038888	Ctu1	-0.070347138	0.033620746
ENSMUSG00000045328	Cenpe	-0.070339819	1.04E-10
ENSMUSG00000031090	Nadsyn1	-0.070286587	9.62E-07
ENSMUSG00000020133	2310011J03F	-0.070229867	0.000192737
ENSMUSG00000054065	Pkp3	-0.070172042	2.63E-09
ENSMUSG00000111709	Gm3776	-0.070152013	1.25E-36
ENSMUSG00000020570	Sypl	-0.070120677	0.000411072
ENSMUSG00000001158	Snrnp27	-0.070050406	4.44E-06
ENSMUSG00000036834	Plch1	-0.069510045	0.031622732
ENSMUSG00000003923	Tfam	-0.069086538	0.000788207
ENSMUSG00000118052	Gm50432	-0.06892733	4.80E-25
ENSMUSG00000047180	Neurl3	-0.068853534	0.00116571
ENSMUSG00000020553	Pctp	-0.06866472	0.030315921
ENSMUSG00000028982	Slc25a33	-0.068550571	0.0010711
ENSMUSG00000018126	Baiap2l2	-0.068475596	6.45E-09
ENSMUSG00000028820	Sfpq	-0.068273009	1.42E-09
ENSMUSG00000033400	Agl	-0.067964345	0.002485741
ENSMUSG00000026088	Mitd1	-0.067740509	2.72E-09
ENSMUSG00000021022	Ppp2r3c	-0.067271423	0.000519166
ENSMUSG00000038528	Mfsd4b5	-0.066939482	0.01070431
ENSMUSG00000109305	Smim38	-0.066929698	0.006244318
ENSMUSG00000042462	Dctpp1	-0.066809287	6.18E-05
ENSMUSG00000049624	Slc17a5	-0.066549194	8.45E-19
ENSMUSG00000026688	Mgst3	-0.066492609	1.11E-16
ENSMUSG00000043866	Taf10	-0.066488105	0.049383084
ENSMUSG00000087022	9130024F11I	-0.066306921	8.21E-05
ENSMUSG00000001036	Epn2	-0.066303155	0.009713512
ENSMUSG00000039081	Zfp503	-0.065977856	1.08E-14
ENSMUSG00000006057	Atp5g1	-0.065944053	0.001452938
ENSMUSG00000034875	Nudt19	-0.065765829	0.000194646
ENSMUSG00000053012	Krcc1	-0.065473345	9.30E-06
ENSMUSG00000015605	Srf	-0.065359196	1.03E-06
ENSMUSG00000020153	Ndufs7	-0.0652732	1.77E-09
ENSMUSG00000061992	Gm4953	-0.065252522	5.92E-15
ENSMUSG00000028393	Alad	-0.065023605	9.00E-10
ENSMUSG00000038462	Uqcrfs1	-0.064860694	5.70E-27
ENSMUSG00000021114	Atp6v1d	-0.064844062	0.009414367
ENSMUSG00000031168	Ebp	-0.064714306	0.000131971
ENSMUSG00000018761	Mpdu1	-0.064544227	2.19E-11
ENSMUSG00000054381	Zfp747	-0.064355935	0.003482977
ENSMUSG00000021280	Exoc3l4	-0.064061968	7.99E-32
ENSMUSG00000059208	Hnrnpm	-0.064027435	2.77E-07
ENSMUSG00000036529	Sbf1	-0.06402511	0.000154689
ENSMUSG00000051022	Hs3st1	-0.063328388	0.000137127
ENSMUSG00000032265	Tent5a	-0.063236869	0.000199618
ENSMUSG00000039263	Npepl1	-0.062843883	0.007368425
ENSMUSG00000029999	Tgfa	-0.062511559	0.014346646
ENSMUSG00000037742	Eef1a1	-0.062319729	7.59E-06
ENSMUSG00000042817	Flt3	-0.0621854	0.034537167
ENSMUSG00000074211	Sdhaf1	-0.062065274	1.88E-08
ENSMUSG00000030980	Knop1	-0.062059212	7.71E-05
ENSMUSG00000031004	Mki67	-0.062000338	3.79E-05
ENSMUSG00000041629	Fam104a	-0.061844272	0.000133295
ENSMUSG00000059343	Aldoart1	-0.061663709	0.043204363
ENSMUSG00000005674	Tomm40l	-0.061639097	0.000217454
ENSMUSG00000020520	Galnt10	-0.061472829	0.012322259
ENSMUSG00000066415	Msl2	-0.061206276	0.032969589
ENSMUSG00000029136	Rbks	-0.061187962	0.000271351
ENSMUSG00000029178	Klf3	-0.06111479	1.73E-11
ENSMUSG00000054417	Cyp3a44	-0.060628696	1.93E-09
ENSMUSG00000043923	Cenatac	-0.0600562	4.88E-05
ENSMUSG00000030823	9130019O22	-0.060014583	0.000591956
ENSMUSG00000032504	Pdcd6ip	-0.059624186	1.50E-08
ENSMUSG00000032475	Nck1	-0.059608839	0.002305727
ENSMUSG00000018567	Gabarap	-0.059535566	0.000238251
ENSMUSG00000027439	Gzf1	-0.05935992	4.48E-08
ENSMUSG00000051435	Fhad1	-0.059336604	0.020906802
ENSMUSG00000017670	Elmo2	-0.059301936	0.036531955
ENSMUSG00000017144	Rnd3	-0.05905996	0.001963708
ENSMUSG00000020156	Pwwp3a	-0.059046458	0.000950705
ENSMUSG00000012422	Tmem167	-0.059031613	1.09E-16
ENSMUSG00000002308	Cd320	-0.058806001	2.71E-05
ENSMUSG00000037148	Arhgap10	-0.058792287	0.048922138
ENSMUSG00000033335	Dnm2	-0.058536529	1.43E-08
ENSMUSG00000001440	Kpnb1	-0.058392725	0.000224619
ENSMUSG00000033577	Myo6	-0.058292541	4.94E-09
ENSMUSG00000024414	Mrpl27	-0.057979918	2.28E-07
ENSMUSG00000034917	Tjp3	-0.057929017	6.69E-15
ENSMUSG00000006589	Aprt	-0.057887341	1.40E-25
ENSMUSG00000117924	Tmem223	-0.057799211	0.010561826
ENSMUSG00000028419	Chmp5	-0.057776369	0.007532518
ENSMUSG00000109901	Chmp1b	-0.057696767	0.000103018
ENSMUSG00000031937	Vstm5	-0.05764877	0.022279962
ENSMUSG00000020022	Ndufa12	-0.057607369	1.30E-22
ENSMUSG00000004633	Chn2	-0.057597123	0.000449015
ENSMUSG00000015342	Xk	-0.057516283	0.0014401
ENSMUSG00000027398	Il1b	-0.057460664	0.021441111
ENSMUSG00000085939	Cd63-ps	-0.057411748	8.78E-13
ENSMUSG00000040463	Mybbp1a	-0.057045539	0.029212476
ENSMUSG00000033684	Qsox1	-0.056828106	1.44E-18
ENSMUSG00000074604	Mgst2	-0.056718655	3.02E-16
ENSMUSG00000025239	Limd1	-0.05671717	3.19E-20
ENSMUSG00000020321	Mdh1	-0.05657835	3.69E-09
ENSMUSG00000028447	Dctn3	-0.05652661	0.001345616

ENSMUSG00000028394	Pole3	0.128476384	0.002159169
ENSMUSG00000005882	Uqcc1	0.128407131	6.63E-13
ENSMUSG00000046185	Zfp84	0.128212648	1.72E-05
ENSMUSG00000032860	P2ry2	0.128158073	4.28E-13
ENSMUSG00000043998	Mgat2	0.128029689	1.11E-09
ENSMUSG00000005566	Trim28	0.128014997	0.000807109
ENSMUSG00000020523	Fam114a2	0.127809598	1.75E-10
ENSMUSG00000020736	Nt5c	0.127696047	8.00E-05
ENSMUSG00000032352	Lrrc1	0.127653485	6.85E-06
ENSMUSG00000032621	Srek1	0.127621147	0.000767964
ENSMUSG00000074748	Atxn7l3b	0.127614422	7.95E-07
ENSMUSG00000044134	Pheta1	0.127567028	6.55E-08
ENSMUSG00000022533	Atp13a3	0.127491644	1.01E-16
ENSMUSG00000018740	Slc25a35	0.127169533	1.30E-05
ENSMUSG00000000131	Xpo6	0.126572557	9.31E-06
ENSMUSG00000078650	G6pc	0.126561057	1.24E-83
ENSMUSG00000094410	Zbed6	0.126348694	0.001192621
ENSMUSG00000026608	Kctd3	0.126018706	0.022802322
ENSMUSG00000055296	Tmem245	0.125879918	0.011831041
ENSMUSG00000036202	Rif1	0.125867381	0.002047968
ENSMUSG00000039886	Tmem120a	0.125687641	2.98E-10
ENSMUSG00000052271	Bhlha15	0.125355724	9.16E-06
ENSMUSG00000025261	Huwe1	0.12529037	0.000251276
ENSMUSG00000024665	Fads2	0.125077367	3.93E-15
ENSMUSG00000021798	Ldb3	0.124949563	1.03E-13
ENSMUSG00000028180	Zranb2	0.124679859	0.001266844
ENSMUSG00000074649	BC029722	0.124614996	8.00E-07
ENSMUSG00000028138	Adh5	0.124020755	6.01E-27
ENSMUSG00000038880	Mrps34	0.123516838	9.87E-09
ENSMUSG00000027650	Tti1	0.122697378	4.06E-05
ENSMUSG00000022244	Amacr	0.122623423	1.48E-06
ENSMUSG00000023852	Chd1	0.122596309	0.002773712
ENSMUSG00000029260	Ugt2b34	0.121932721	7.51E-54
ENSMUSG00000056899	Immp2l	0.121865054	1.08E-05
ENSMUSG00000009647	Mcu	0.121842158	0.004831708
ENSMUSG00000017119	Nbr1	0.121485246	0.000107037
ENSMUSG00000073412	Lst1	0.121265162	0.041782234
ENSMUSG00000022295	Atp6v1c1	0.121202553	9.63E-05
ENSMUSG00000034160	Ogt	0.120674755	0.000336715
ENSMUSG00000028149	Rap1gds1	0.120577289	0.014625219
ENSMUSG00000030780	Rusf1	0.120556455	0.032271422
ENSMUSG00000029727	Cyp3a13	0.120491834	9.75E-109
ENSMUSG00000034255	Arhgap27	0.120486749	0.025516425
ENSMUSG00000067071	Hes6	0.120196526	2.66E-05
ENSMUSG00000033161	Atp1a1	0.120142509	0.003626641
ENSMUSG00000030905	Crym	0.120072543	5.69E-26
ENSMUSG00000029646	Cdx2	0.120050865	0.033237767
ENSMUSG00000025130	P4hb	0.119405251	1.23E-36
ENSMUSG00000024378	Stard4	0.119294163	9.73E-20
ENSMUSG00000026413	Pkp1	0.119285026	5.97E-05
ENSMUSG00000046814	Gchfr	0.119241118	4.89E-13
ENSMUSG00000032279	Idh3a	0.118919839	3.48E-17
ENSMUSG00000002413	Braf	0.118751964	0.005842777
ENSMUSG00000019590	Cyb561	0.118741429	3.34E-06
ENSMUSG00000030763	Lcmt1	0.118504772	2.60E-10
ENSMUSG00000035048	Anapc13	0.118424822	0.001240041
ENSMUSG00000006740	Kif5b	0.118280569	0.003931984
ENSMUSG00000022701	Ccdc191	0.118109006	0.006088592
ENSMUSG00000001755	Coasy	0.118086334	4.08E-06
ENSMUSG00000024456	Diaph1	0.117913793	2.17E-08
ENSMUSG00000045983	Eif4g1	0.11782998	0.008852901
ENSMUSG00000024474	Ik	0.117823534	0.014622793
ENSMUSG00000029557	Mrm2	0.117804632	0.001665156
ENSMUSG00000028890	Mtf1	0.117445805	8.79E-15
ENSMUSG00000056598	Drc3	0.116967002	0.000691367
ENSMUSG00000025921	Rdh10	0.116950909	7.95E-10
ENSMUSG00000024392	Bag6	0.116819576	0.031791721
ENSMUSG00000039166	Akap7	0.116658382	0.002448445
ENSMUSG00000041133	Smc1a	0.116195131	1.69E-08
ENSMUSG00000031078	Cttn	0.116162681	6.29E-32
ENSMUSG00000044285	Ubb-ps	0.115987145	6.86E-06
ENSMUSG00000026632	Tatdn3	0.115896046	5.06E-05
ENSMUSG00000051232	Tmem199	0.11544892	0.003149999
ENSMUSG00000045165	AI467606	0.115355538	0.004120191
ENSMUSG00000053886	Sh2d4a	0.115287933	0.011137244
ENSMUSG00000022330	Osr2	0.115238921	4.46E-66
ENSMUSG00000025466	Fuom	0.114934362	1.49E-15
ENSMUSG00000020307	Cdc34	0.114604584	0.001502892
ENSMUSG00000003420	Fcgrt	0.114587567	7.71E-07
ENSMUSG00000054823	Nsd3	0.114433674	0.002368838
ENSMUSG00000015176	Nolc1	0.11418092	0.031546335
ENSMUSG00000019179	Mdh2	0.114097367	5.96E-92
ENSMUSG00000070780	Rbm47	0.113694827	0.009359754
ENSMUSG00000034088	Hdlbp	0.113549918	0.015555348
ENSMUSG00000020611	Gna13	0.113453441	0.001757206
ENSMUSG00000020644	Id2	0.112928504	4.95E-24
ENSMUSG00000032870	Smap2	0.112757938	0.031769501
ENSMUSG00000026839	Upp2	0.112629784	0.006939633
ENSMUSG00000018509	Cenpv	0.112462464	8.51E-05
ENSMUSG00000022255	Mtdh	0.11243019	1.59E-07
ENSMUSG00000020895	Tmem107	0.112245324	0.009959751
ENSMUSG00000028399	Ptprd	0.1119419	1.30E-19
ENSMUSG00000014932	Yes1	0.111928248	8.90E-05
ENSMUSG00000025068	Gsto1	0.111807845	6.38E-22
ENSMUSG00000030681	Mvp	0.111548247	2.46E-26
ENSMUSG00000027620	Rbm39	0.111465	0.000877095
ENSMUSG00000020530	Ggnbp2	0.111420037	0.020002825
ENSMUSG00000012114	Med15	0.111352965	8.81E-05
ENSMUSG00000040479	Dgkz	0.111091961	1.12E-09
ENSMUSG00000022663	Atg3	0.11103465	0.046384458
ENSMUSG00000057367	Birc2	0.110615667	0.001685219
ENSMUSG00000041763	Tpp2	0.110245476	3.70E-05
ENSMUSG00000026179	Pnkd	0.110023898	2.03E-08
ENSMUSG00000027661	Slc2a10	0.109920163	0.009381024
ENSMUSG00000034427	Myo15b	0.10980466	3.04E-06
ENSMUSG00000024797	Vps51	0.109303834	0.0087029
ENSMUSG00000031955	Bcar1	0.109193087	0.000928109
ENSMUSG00000022185	Acin1	0.109185955	2.07E-11
ENSMUSG00000101795	Gm5835	0.109089025	3.94E-11
ENSMUSG00000037999	Arap2	0.108287243	1.33E-07
ENSMUSG00000000078	Klf6	0.10826938	9.95E-07
ENSMUSG00000009828	Cilk1	0.108229269	3.62E-06
ENSMUSG00000022561	Gpaa1	0.108166544	1.66E-09
ENSMUSG00000039063	Echdc3	0.107908439	0.009601643
ENSMUSG00000051510	Mafg	0.107902884	0.000235616
ENSMUSG00000075415	Fnbp1	0.107399635	0.027059533
ENSMUSG00000025731	Mettl26	0.106983782	9.87E-07
ENSMUSG00000025702	Marchf8	0.106815835	7.12E-05
ENSMUSG00000025484	Bet1l	0.106755709	1.78E-05
ENSMUSG00000030286	Emc3	0.106638758	0.010654996
ENSMUSG00000005656	Snx6	0.106195091	0.000478555
ENSMUSG00000018572	Phf23	0.106082767	0.023972146

ENSMUSG00000022214	Dcaf11	-0.067357763	0.000370682
ENSMUSG00000003199	Mpnd	-0.067128484	0.010688445
ENSMUSG00000051316	Taf7	-0.066993705	0.000471846
ENSMUSG00000024955	Esrra	-0.06683428	5.68E-07
ENSMUSG00000026499	Acbd3	-0.066821048	1.02E-05
ENSMUSG00000031839	Hsbp1	-0.066765003	0.000367421
ENSMUSG00000023942	Slc29a1	-0.066604272	0.013507646
ENSMUSG00000027285	Haus2	-0.066425968	0.000337974
ENSMUSG00000021361	Tmem14c	-0.066277562	2.08E-11
ENSMUSG00000041997	Tlk1	-0.066051745	3.79E-06
ENSMUSG00000002416	Ndufb2	-0.065992921	5.31E-16
ENSMUSG00000026784	Pdss1	-0.065933785	1.09E-05
ENSMUSG00000027566	Psma7	-0.065623153	9.31E-08
ENSMUSG00000028898	Trnau1ap	-0.065607065	0.00174785
ENSMUSG00000024474	Ik	-0.06556538	0.014622793
ENSMUSG00000027263	Tubgcp4	-0.06544696	0.00388279
ENSMUSG00000034525	Ice1	-0.065222869	0.0012208
ENSMUSG00000057762	Gm6169	-0.065210446	0.04605327
ENSMUSG00000020585	Laptm4a	-0.065202865	9.83E-07
ENSMUSG00000025357	Dgka	-0.065185393	3.59E-11
ENSMUSG00000074088	Snrnp40	-0.065161029	0.015063984
ENSMUSG00000031004	Mki67	-0.065143178	3.79E-05
ENSMUSG00000035840	Lysmd3	-0.064883756	0.000639615
ENSMUSG00000020914	Top2a	-0.064817866	1.61E-07
ENSMUSG00000022445	Cyp2d26	-0.064592374	1.04E-24
ENSMUSG00000068551	Zfp467	-0.064500117	2.08E-11
ENSMUSG00000076435	Acsf2	-0.064481821	0.014387553
ENSMUSG00000047824	Pygo2	-0.064255924	0.000122924
ENSMUSG00000059456	Ptk2b	-0.064197669	0.008001785
ENSMUSG00000021576	Pdcd6	-0.064008354	9.76E-11
ENSMUSG00000038298	Pdzk1	-0.06356216	5.73E-11
ENSMUSG00000037706	Cd81	-0.063541287	2.43E-08
ENSMUSG00000027937	Jtb	-0.063513148	4.35E-05
ENSMUSG00000038982	Bloc1s5	-0.063386393	0.01436856
ENSMUSG00000037606	Osbpl5	-0.063169091	0.000846162
ENSMUSG00000058301	Upf1	-0.063167919	0.029220285
ENSMUSG00000031029	Eif3f	-0.06276816	1.35E-13
ENSMUSG00000060733	Ipmk	-0.062686387	4.17E-30
ENSMUSG00000030536	Iqgap1	-0.062464385	0.025806961
ENSMUSG00000002881	Nab1	-0.062356654	0.005668452
ENSMUSG00000051232	Tmem199	-0.062243839	0.003149999
ENSMUSG00000054128	H2-T3	-0.06218797	9.39E-10
ENSMUSG00000035372	1810055G02	-0.062095051	5.70E-06
ENSMUSG00000041415	Dicer1	-0.062077705	0.003977245
ENSMUSG00000044134	Pheta1	-0.061764818	6.55E-08
ENSMUSG00000025237	Parp6	-0.061432539	0.013715055
ENSMUSG00000027454	Gins1	-0.061431619	0.015930305
ENSMUSG00000026924	Sec16a	-0.061275542	0.006270663
ENSMUSG00000032481	Smarcc1	-0.060858656	1.50E-06
ENSMUSG00000030213	Atf7ip	-0.060818317	0.015247343
ENSMUSG00000030560	Ctsc	-0.060775354	2.87E-08
ENSMUSG00000003309	Ap1m2	-0.060727283	0.000509684
ENSMUSG00000015880	Ncapg	-0.060664846	0.007399736
ENSMUSG00000036155	Mgat5	-0.06064809	0.001437345
ENSMUSG00000003464	Pex19	-0.060620131	0.00019839
ENSMUSG00000012535	Tnpo3	-0.060602775	0.007699726
ENSMUSG00000028042	Zbtb7b	-0.060110512	5.68E-07
ENSMUSG00000041638	Gcn1	-0.060036023	0.006223857
ENSMUSG00000031445	Proz	-0.0600267	8.92E-05
ENSMUSG00000119584	Rn18s	-0.059903912	0.021790683
ENSMUSG00000027746	Ufm1	-0.059678692	1.65E-07
ENSMUSG00000020521	Rnft1	-0.05949409	0.032828358
ENSMUSG00000084957	Bbip1	-0.059455699	0.000109372
ENSMUSG00000028743	Akr7a5	-0.059376802	6.92E-05
ENSMUSG00000050043	Tmx2	-0.058980021	0.00107871
ENSMUSG00000049553	Polr1a	-0.058890196	1.67E-05
ENSMUSG00000032575	Manf	-0.058869069	0.002983244
ENSMUSG00000015134	Aldh1a3	-0.05845268	2.74E-12
ENSMUSG00000074300	Smim31	-0.058285722	8.27E-24
ENSMUSG00000032673	Prorsd1	-0.05825934	0.003129031
ENSMUSG00000035852	Misp	-0.057977335	0.030909149
ENSMUSG00000029071	Dvl1	-0.057974578	0.000510606
ENSMUSG00000081051	Gm15427	-0.057765733	0.005867786
ENSMUSG00000046727	Cystm1	-0.057524904	2.21E-16
ENSMUSG00000051355	Commd1	-0.057417248	0.000227116
ENSMUSG00000029148	Nrbp1	-0.057007646	0.001002166
ENSMUSG00000063077	Kif1b	-0.056534538	1.42E-09
ENSMUSG00000031158	Timm17b	-0.056053415	1.66E-06
ENSMUSG00000063172	Ift25	-0.056016522	0.04423346
ENSMUSG00000031570	Plpp5	-0.055818525	0.003550684
ENSMUSG00000060904	Arl1	-0.055736569	0.000107409
ENSMUSG00000042029	Ncapg2	-0.055406333	0.038920372
ENSMUSG00000037916	Ndufv1	-0.055329094	1.82E-13
ENSMUSG00000051319	Mtln	-0.055287549	0.007412826
ENSMUSG00000029275	Gfi1	-0.055205661	0.031129781
ENSMUSG00000050965	Prkca	-0.054608378	0.012516318
ENSMUSG00000037999	Arap2	-0.054359545	1.33E-07
ENSMUSG00000026269	Rnpepl1	-0.053843243	0.001623468
ENSMUSG00000022912	Pros1	-0.053030065	0.003138142
ENSMUSG00000000826	Dnajc5	-0.052913212	0.003608153
ENSMUSG00000036983	Tfb1m	-0.052813452	0.017476017
ENSMUSG00000036529	Sbf1	-0.052135017	0.000154689
ENSMUSG00000041440	Gk5	-0.052088404	0.015898488
ENSMUSG00000023043	Krt18	-0.052068877	4.36E-25
ENSMUSG00000038848	Ythdf1	-0.052025375	0.000453027
ENSMUSG00000035041	Creb3l3	-0.051737031	3.89E-31
ENSMUSG00000019362	D8Ertd738e	-0.051680132	0.000800747
ENSMUSG00000041957	Pkp2	-0.051478795	0.00183989
ENSMUSG00000020205	Phlda1	-0.051368901	0.006705753
ENSMUSG00000057506	Bloc1s2	-0.051315437	0.033119485
ENSMUSG00000025138	Sirt7	-0.05118887	0.007848567
ENSMUSG00000020115	Tbk1	-0.051188039	0.007256752
ENSMUSG00000027082	Tfpi	-0.051168695	6.20E-25
ENSMUSG00000028096	Gpr89	-0.051098627	1.66E-13
ENSMUSG00000038195	Rilp	-0.050553532	3.61E-05
ENSMUSG00000003923	Tfam	-0.050374879	0.000788207
ENSMUSG00000028843	Sh3bgrl3	-0.050276515	8.00E-07
ENSMUSG00000005677	Nr1i3	-0.050254446	2.20E-40
ENSMUSG00000007812	Zfp655	-0.050197346	0.017899446
ENSMUSG00000021144	Mta1	-0.049885705	0.001320277
ENSMUSG00000047466	8030462N17	-0.04973839	0.044373609
ENSMUSG00000026097	Ormdl1	-0.049522393	3.82E-05
ENSMUSG00000047866	Lonp2	-0.048921803	4.54E-12
ENSMUSG00000052392	Acot4	-0.048880031	0.002666373
ENSMUSG00000070780	Rbm47	-0.048762468	0.009359754
ENSMUSG00000034320	Slc26a2	-0.048518652	5.27E-83
ENSMUSG00000028567	Txndc12	-0.04843503	1.64E-05
ENSMUSG00000009687	Fxyd5	-0.048130688	0.003287939
ENSMUSG00000034382	AI661453	-0.047855284	0.007791961
ENSMUSG00000060227	Golm2	-0.047669021	3.00E-05
ENSMUSG00000054263	Lifr	-0.046818487	3.22E-05
ENSMUSG00000028719	Cmpk1	-0.0468024	7.37E-06

ENSMUSG00000024317	Rnf138	0.103362976	0.03430665
ENSMUSG00000110344	Smim36	0.102793112	0.00017335
ENSMUSG00000024513	Mbd2	0.102385797	0.046498405
ENSMUSG00000009995	Tafazzin	0.102198366	1.47E-05
ENSMUSG00000028229	Rmdn1	0.102158137	9.26E-26
ENSMUSG00000029028	Lrrc47	0.102066156	0.011874495
ENSMUSG00000027954	Efna1	0.101170649	5.94E-08
ENSMUSG00000027350	Chgb	0.100936007	1.45E-08
ENSMUSG00000078622	Ccdc47	0.100913456	0.007901452
ENSMUSG00000054676	1600014C10	0.10073757	1.62E-18
ENSMUSG00000007827	Ankrd26	0.100438524	0.009790995
ENSMUSG00000042202	Slc35e2	0.100324899	0.023242209
ENSMUSG00000041702	Btbd7	0.10024226	0.00030282
ENSMUSG00000114358	Gm5200	0.10017749	6.33E-07
ENSMUSG00000054226	Tprkb	0.100136822	8.50E-11
ENSMUSG00000002846	Timmdc1	0.100047555	0.013911135
ENSMUSG00000018481	Appbp2	0.099705375	0.017726204
ENSMUSG00000029106	Add1	0.099459714	2.03E-09
ENSMUSG00000049624	Slc17a5	0.09900655	8.45E-19
ENSMUSG00000014551	Mrps25	0.098930237	0.001012552
ENSMUSG00000022338	Eny2	0.098399419	1.59E-08
ENSMUSG00000002280	Ciao3	0.098291491	0.024637667
ENSMUSG00000015804	Med28	0.098281587	0.000353459
ENSMUSG00000025477	Inpp5a	0.098256773	7.44E-10
ENSMUSG00000113902	Ndufb1	0.097750808	2.72E-06
ENSMUSG00000015668	Pdzd11	0.097592928	0.000307315
ENSMUSG00000057637	Prdm2	0.097545187	0.003113836
ENSMUSG00000039753	Fbxl5	0.097359709	4.14E-08
ENSMUSG00000015656	Hspa8	0.097280714	0.000460382
ENSMUSG00000027303	Ptpra	0.097137302	0.048797284
ENSMUSG00000015937	Macroh2a1	0.096602992	0.004329772
ENSMUSG00000056612	Ppp1r14b	0.096052988	6.15E-05
ENSMUSG00000002767	Mrpl2	0.095728913	0.005032346
ENSMUSG00000028212	Ccne2	0.095690737	0.000404731
ENSMUSG00000006395	Hyi	0.095677492	0.037292703
ENSMUSG00000024241	Sos1	0.095619317	0.019527073
ENSMUSG00000026698	Pigc	0.095454662	8.40E-06
ENSMUSG00000042726	Trafd1	0.095393721	1.55E-09
ENSMUSG00000040447	Spns2	0.095257904	4.00E-10
ENSMUSG00000050530	Fam171a1	0.095193647	0.000286573
ENSMUSG00000022370	Mrpl13	0.095136305	1.12E-07
ENSMUSG00000028675	Pnrc2	0.095087997	0.002863014
ENSMUSG00000031634	Ufsp2	0.094853113	0.009307836
ENSMUSG00000024914	Drap1	0.094666665	0.024215576
ENSMUSG00000070697	Utp3	0.094314839	0.003453114
ENSMUSG00000019738	Polr2i	0.093956684	0.013063757
ENSMUSG00000041959	S100a10	0.093793434	0.003982743
ENSMUSG00000027646	Src	0.093772275	0.001157494
ENSMUSG00000031955	Bcar1	0.093757707	0.000928109
ENSMUSG00000028327	Stra6l	0.093612073	0.029706774
ENSMUSG00000040722	Scamp5	0.093587931	1.24E-27
ENSMUSG00000050856	Atp5k	0.093303672	2.01E-11
ENSMUSG00000051154	Commd3	0.093169648	0.000548183
ENSMUSG00000090035	Galnt4	0.093143717	1.08E-07
ENSMUSG00000047230	Cldn2	0.093106931	1.81E-22
ENSMUSG00000069208	Zfp825	0.093009521	0.011382329
ENSMUSG00000020600	Slc7a15	0.092744971	8.49E-05
ENSMUSG00000033589	Reep4	0.09265411	2.62E-10
ENSMUSG00000026987	Baz2b	0.092609356	0.00726097
ENSMUSG00000042390	Gatad2b	0.092118272	1.79E-05
ENSMUSG00000041488	Stx3	0.092065335	0.00904923
ENSMUSG00000027508	Pag1	0.092035098	0.009055767
ENSMUSG00000052915	Msl1	0.091858197	4.71E-05
ENSMUSG00000000827	Tpd52l2	0.091842259	0.003759036
ENSMUSG00000060208	Defa17	0.09145158	0.035080957
ENSMUSG00000041215	Yeats2	0.091433804	0.022893675
ENSMUSG00000028779	Pef1	0.091217835	1.11E-05
ENSMUSG00000025534	Gusb	0.090926329	0.000365595
ENSMUSG00000035268	Pkig	0.090894872	0.000960297
ENSMUSG00000026915	Strbp	0.090887322	0.004057186
ENSMUSG00000020570	Sypl	0.090832009	0.000411072
ENSMUSG00000028461	Ccdc107	0.090678161	1.42E-12
ENSMUSG00000045991	Onecut2	0.090645161	2.21E-107
ENSMUSG00000023235	Ccl25	0.090146321	0.000194839
ENSMUSG00000046805	Mpeg1	0.090125606	0.002898664
ENSMUSG00000026640	Plxna2	0.089860699	0.000446959
ENSMUSG00000031333	Abcb7	0.089857293	0.001190587
ENSMUSG00000023755	Rhebl1	0.089769105	1.43E-05
ENSMUSG00000022337	Emc2	0.089577754	2.38E-08
ENSMUSG00000028028	Alpk1	0.089550769	1.94E-10
ENSMUSG00000028015	Ctso	0.089484176	1.13E-11
ENSMUSG00000029622	Arpc1b	0.089397312	0.00360538
ENSMUSG00000041870	Ankrd13a	0.089114583	1.73E-11
ENSMUSG00000026489	Coq8a	0.088885234	2.01E-05
ENSMUSG00000038762	Abcf1	0.088804823	2.16E-05
ENSMUSG00000110926	Gm5917	0.088742276	0.038452778
ENSMUSG00000034602	Mon2	0.088695194	0.02836377
ENSMUSG00000038872	Zfhx3	0.088623139	0.000111517
ENSMUSG00000040412	Elapor1	0.088588448	2.08E-11
ENSMUSG00000013593	Ndufs2	0.088377135	3.61E-25
ENSMUSG00000025204	Ndufb8	0.088133574	1.08E-21
ENSMUSG00000028064	Sema4a	0.087849212	4.52E-06
ENSMUSG00000020863	Luc7l3	0.087601908	7.33E-07
ENSMUSG00000029032	Arhgef16	0.087147449	0.018036017
ENSMUSG00000061132	Blnk	0.087143536	0.003621224
ENSMUSG00000030096	Slc6a6	0.087118527	3.31E-11
ENSMUSG00000035674	Ndufa3	0.086957971	6.19E-12
ENSMUSG00000019851	Perp	0.086914226	0.048314041
ENSMUSG00000033940	Brk1	0.086754386	0.005311537
ENSMUSG00000024841	Eif1ad	0.086508934	8.22E-26
ENSMUSG00000045817	Zfp36l2	0.086242204	0.029801642
ENSMUSG00000005615	Pcyt1a	0.085907533	2.93E-15
ENSMUSG00000040918	Slc19a2	0.08579442	5.72E-06
ENSMUSG00000003031	Cdkn1b	0.085770521	0.00418167
ENSMUSG00000046897	Zfp740	0.085685407	0.041346824
ENSMUSG00000001552	Jup	0.085520443	1.38E-14
ENSMUSG00000029923	Rab19	0.085511517	0.00072332
ENSMUSG00000023951	Vegfa	0.085289483	0.000529776
ENSMUSG00000057948	Unc13d	0.084802523	0.016400404
ENSMUSG00000024781	Lipa	0.084621489	2.34E-40
ENSMUSG00000024145	Pigf	0.084306238	0.010522271
ENSMUSG00000045576	St7l	0.084139246	0.003982036
ENSMUSG00000020923	Ubtf	0.083829607	0.001666561
ENSMUSG00000003746	Man1a	0.083688961	0.003745688
ENSMUSG00000042677	Zc3h12a	0.08366888	3.44E-17
ENSMUSG00000001632	Brpf1	0.083566602	2.33E-06
ENSMUSG00000030842	Lamtor1	0.083369835	0.000155204
ENSMUSG00000062929	Cfl2	0.083330131	0.014344397
ENSMUSG00000024456	Diaph1	0.083053498	2.17E-08
ENSMUSG00000053205	Styx	0.083049952	0.031809287
ENSMUSG00000031451	Gas6	0.082932201	1.30E-11
ENSMUSG00000034203	Chchd4	0.082846084	1.84E-10

ENSMUSG00000031715	Smarca5	-0.056304087	0.005351987
ENSMUSG00000039217	Il18	-0.056187655	1.02E-57
ENSMUSG00000048076	Arf1	-0.055965793	0.012849896
ENSMUSG00000078670	Fam174b	-0.05596248	4.95E-10
ENSMUSG00000019797	Mtres1	-0.055641971	9.72E-05
ENSMUSG00000019761	Krt10	-0.055398074	0.029801642
ENSMUSG00000032376	Usp3	-0.054614092	0.00058818
ENSMUSG00000002064	Sdf2	-0.054397629	0.000274522
ENSMUSG00000028803	Nipal3	-0.054378395	0.004483969
ENSMUSG00000097908	4933404O12	-0.054345389	0.000358273
ENSMUSG00000026672	Optn	-0.054298749	0.00016076
ENSMUSG00000019971	Cep290	-0.054260749	0.019315693
ENSMUSG00000034127	Tspan8	-0.054225984	2.29E-20
ENSMUSG00000027247	Arhgap1	-0.053782302	5.25E-22
ENSMUSG00000015143	Actn1	-0.053702786	7.61E-08
ENSMUSG00000070493	Chchd2	-0.053328553	3.59E-09
ENSMUSG00000023456	Tpi1	-0.053109222	9.89E-24
ENSMUSG00000028069	Gpatch4	-0.052922342	0.005724025
ENSMUSG00000067149	Jchain	-0.052835094	4.39E-14
ENSMUSG00000022110	Sucla2	-0.052791471	2.47E-14
ENSMUSG00000026615	Eprs	-0.052591428	2.06E-06
ENSMUSG00000032563	Mrpl3	-0.052404617	0.000260491
ENSMUSG00000025793	Hgs	-0.052337769	0.001014632
ENSMUSG00000034057	Myrfl	-0.052313884	6.26E-06
ENSMUSG00000017715	Pgs1	-0.052100878	0.034162871
ENSMUSG00000030091	Nup210	-0.05202765	0.000841206
ENSMUSG00000011034	Slc5a1	-0.051982507	1.41E-34
ENSMUSG00000055044	Pdlim1	-0.051852629	3.92E-27
ENSMUSG00000036398	Ppp1r11	-0.051750862	6.99E-10
ENSMUSG00000014294	Ndufa2	-0.051550512	3.46E-08
ENSMUSG00000036371	Serbp1	-0.051235749	0.011213722
ENSMUSG00000092203	1110038B12	-0.051193065	0.000242296
ENSMUSG00000038039	Gcc2	-0.051183291	0.000123318
ENSMUSG00000040242	Fgfr1op2	-0.050839336	5.59E-05
ENSMUSG00000038650	Rnh1	-0.050839006	5.83E-07
ENSMUSG00000032216	Nedd4	-0.05050838	5.76E-10
ENSMUSG00000039630	Hnrnpu	-0.050468451	4.44E-06
ENSMUSG00000019791	Hint3	-0.050228305	3.02E-09
ENSMUSG00000003662	Ciao1	-0.049988722	0.002468415
ENSMUSG00000078570	1110065P20	-0.049975889	4.84E-06
ENSMUSG00000079111	Kdelr2	-0.049917436	0.000124785
ENSMUSG00000025173	Wdr45b	-0.049566295	0.027059533
ENSMUSG00000034345	Gtf2h5	-0.048916923	1.04E-10
ENSMUSG00000073043	Atoh1	-0.048741866	0.000105556
ENSMUSG00000005625	Psmd4	-0.048573011	2.83E-08
ENSMUSG00000038991	Txndc5	-0.048565949	2.70E-06
ENSMUSG00000029552	Tes	-0.048209591	0.011356575
ENSMUSG00000021144	Mta1	-0.048175762	0.001320277
ENSMUSG00000030849	Fgfr2	-0.04817113	0.017722889
ENSMUSG00000029467	Atp2a2	-0.047992964	8.20E-13
ENSMUSG00000019689	Fmc1	-0.047847971	3.45E-13
ENSMUSG00000047649	Polr1g	-0.047726889	0.000423217
ENSMUSG00000029416	Slc15a4	-0.047563413	0.001469359
ENSMUSG00000007656	Arpp19	-0.047253223	9.93E-07
ENSMUSG00000005899	Smpd4	-0.047207467	0.010549069
ENSMUSG00000056919	Cep162	-0.047003583	0.001684702
ENSMUSG00000037916	Ndufv1	-0.046905478	1.82E-13
ENSMUSG00000021822	Plau	-0.046872994	0.001590207
ENSMUSG00000043510	Hscb	-0.046760685	0.000377612
ENSMUSG00000040506	Ambra1	-0.046759487	0.020694027
ENSMUSG00000026664	Phyh	-0.046685784	1.87E-40
ENSMUSG00000061848	Gm5805	-0.046185047	0.002739169
ENSMUSG00000025158	Rfng	-0.045777093	1.40E-05
ENSMUSG00000033883	Zfp267	-0.045756149	0.036480425
ENSMUSG00000029191	Rfc1	-0.045678944	8.01E-15
ENSMUSG00000006304	Arpc2	-0.045647509	0.002529459
ENSMUSG00000034341	Wbp2	-0.045636174	5.67E-05
ENSMUSG00000002395	Use1	-0.045557827	0.002468176
ENSMUSG00000001999	Blvra	-0.045440494	0.013841793
ENSMUSG00000026979	Psd4	-0.045151463	0.033620746
ENSMUSG00000020219	Timm13	-0.044559574	6.20E-05
ENSMUSG00000031782	Coq9	-0.044519788	3.31E-11
ENSMUSG00000001089	Luzp1	-0.044370397	1.78E-09
ENSMUSG00000027067	Ssrp1	-0.044183349	1.17E-08
ENSMUSG00000027002	Nckap1	-0.043993577	0.001750757
ENSMUSG00000024312	Wdr46	-0.043955413	0.001207778
ENSMUSG00000040725	Hnrnpul1	-0.0435356	0.000296927
ENSMUSG00000032399	Rpl4	-0.04349275	4.55E-16
ENSMUSG00000025381	Cnpy2	-0.043360491	0.034034709
ENSMUSG00000032388	Spg21	-0.043013976	0.027159598
ENSMUSG00000028549	Itgb3bp	-0.042818668	0.03949428
ENSMUSG00000036990	Otud4	-0.042642417	8.37E-11
ENSMUSG00000019897	Ccdc59	-0.042419526	1.69E-15
ENSMUSG00000028337	Coro2a	-0.042260661	2.04E-13
ENSMUSG00000002319	Ipo4	-0.041825518	0.001672171
ENSMUSG00000063511	Snrnp70	-0.041771175	0.000127623
ENSMUSG00000031010	Usp9x	-0.041692172	0.002400918
ENSMUSG00000023089	Ndufa5	-0.04153868	1.43E-14
ENSMUSG00000040605	Bace2	-0.041361818	5.54E-07
ENSMUSG00000027997	Casp6	-0.041324596	3.10E-82
ENSMUSG00000015536	Mocs2	-0.041095332	1.69E-24
ENSMUSG00000024052	Lpin2	-0.041087517	2.55E-08
ENSMUSG00000044408	Sptssa	-0.040802018	4.42E-06
ENSMUSG00000062580	Timm17a	-0.040683921	3.64E-05
ENSMUSG00000048249	Crebrf	-0.040598187	0.001209363
ENSMUSG00000041836	Ptpre	-0.040493394	0.001086641
ENSMUSG00000019849	Prep	-0.040400423	0.000867088
ENSMUSG00000053453	Thoc7	-0.04027414	6.26E-06
ENSMUSG00000024683	Mrpl16	-0.040019796	8.90E-05
ENSMUSG00000026946	Nmi	-0.040009463	0.003139158
ENSMUSG00000006498	Ptbp1	-0.039622038	4.17E-05
ENSMUSG00000010358	Ifi35	-0.03944764	1.73E-25
ENSMUSG00000013539	Tango2	-0.039420714	0.001895881
ENSMUSG00000003200	Sh3gl1	-0.039267956	0.009214125
ENSMUSG00000061665	Cd2ap	-0.039256318	2.35E-19
ENSMUSG00000031392	Irak1	-0.03903454	0.012161377
ENSMUSG00000037787	Coa8	-0.038996042	0.00075467
ENSMUSG00000058587	Tmod3	-0.038835213	0.0030253
ENSMUSG00000020089	Ppa1	-0.038616032	3.27E-77
ENSMUSG00000036315	Polr1h	-0.038574315	0.037303577
ENSMUSG00000021076	Actr10	-0.03845324	0.043327932
ENSMUSG00000068580	Zfyve19	-0.038430538	0.000941387
ENSMUSG00000030614	Tmem126b	-0.038371211	0.002785078
ENSMUSG00000042569	Dhrs7b	-0.038156004	4.43E-07
ENSMUSG00000047454	Gphn	-0.038086252	6.44E-06
ENSMUSG00000034616	Ssh3	-0.037933092	0.005730713
ENSMUSG00000014313	Cox6c	-0.037901237	2.39E-10
ENSMUSG00000044860	Gm1123	-0.03763569	4.99E-07
ENSMUSG00000020380	Rad50	-0.037091875	1.98E-11
ENSMUSG00000001419	Mef2d	-0.036943241	3.60E-09
ENSMUSG00000028251	Tstd3	-0.036911364	0.045460277
ENSMUSG00000031931	Ankrd49	-0.036703935	0.039244718

ENSMUSG00000091811	Inafm1	0.106021835	0.000500059
ENSMUSG00000033952	Aspm	0.105896048	2.11E-08
ENSMUSG00000015542	Nat9	0.105849991	1.59E-06
ENSMUSG00000039048	Foxred1	0.105656403	0.000818399
ENSMUSG00000029273	Sult1d1	0.105072078	5.00E-18
ENSMUSG00000032366	Tpm1	0.105044718	0.000464979
ENSMUSG00000036718	Micall2	0.105033962	0.001765034
ENSMUSG00000038095	Sbno1	0.104685363	0.000642374
ENSMUSG00000028962	Slc4a2	0.103986348	0.037509467
ENSMUSG00000037958	Nsrp1	0.103880933	0.00312632
ENSMUSG00000051359	Ncald	0.103284362	8.72E-15
ENSMUSG00000045690	Wdr89	0.10229602	0.012218937
ENSMUSG00000004897	Hdgf	0.102143025	3.14E-10
ENSMUSG00000029061	Mmp23	0.101815598	6.05E-07
ENSMUSG00000034602	Mon2	0.10112431	0.02836377
ENSMUSG00000027893	Ahcyl1	0.100615876	6.67E-42
ENSMUSG00000020758	Itgb4	0.100139415	3.20E-10
ENSMUSG00000045854	Lyrm2	0.099915129	0.000464429
ENSMUSG00000027227	Sord	0.099907048	1.15E-07
ENSMUSG00000040891	Foxa3	0.09983263	4.24E-09
ENSMUSG00000042709	Atpaf2	0.099709042	0.002489915
ENSMUSG00000040359	Ufl1	0.099677382	0.001061784
ENSMUSG00000024769	Cdc42bpg	0.099675173	5.10E-08
ENSMUSG00000022462	Slc38a2	0.099481579	5.82E-11
ENSMUSG00000021549	Rasa1	0.099454415	0.026991512
ENSMUSG00000029229	Chic2	0.099345436	0.025136461
ENSMUSG00000020903	Stx8	0.09925534	0.019730174
ENSMUSG00000022130	Tgds	0.099218494	2.74E-05
ENSMUSG00000021327	Zkscan3	0.099063511	6.90E-05
ENSMUSG00000023755	Rhebl1	0.098813889	1.43E-05
ENSMUSG00000026032	Ndufb3	0.098634255	3.89E-12
ENSMUSG00000059586	Nsmce2	0.098481877	0.045720225
ENSMUSG00000067150	Xpo5	0.098068679	0.009380488
ENSMUSG00000032187	Smarca4	0.097938584	5.21E-10
ENSMUSG00000026083	Eif5b	0.097785117	2.62E-08
ENSMUSG00000025979	Mob4	0.097776416	2.95E-05
ENSMUSG00000015755	Tab2	0.097327144	1.79E-06
ENSMUSG00000078963	Hsbp1l1	0.097057337	0.01282466
ENSMUSG00000040383	Aqr	0.096954865	2.87E-05
ENSMUSG00000033940	Brk1	0.096343427	0.005311537
ENSMUSG00000003299	Mrpl4	0.09614875	2.88E-07
ENSMUSG00000035898	Uba6	0.095910229	0.018622287
ENSMUSG00000038415	Foxq1	0.095857528	3.11E-09
ENSMUSG00000031311	Nono	0.095631597	0.003877031
ENSMUSG00000042520	Ubap2l	0.095403275	0.001897836
ENSMUSG00000041439	Mfsd6	0.095271433	3.55E-09
ENSMUSG00000030551	Nr2f2	0.094944197	3.84E-21
ENSMUSG00000020653	Klf11	0.094519108	0.010733749
ENSMUSG00000044026	Slc35g1	0.094460933	2.10E-06
ENSMUSG00000044496	2510039O18	0.094455374	9.53E-05
ENSMUSG00000010054	Tusc2	0.094342656	7.88E-06
ENSMUSG00000026473	Glul	0.094100359	2.04E-05
ENSMUSG00000031075	Ano1	0.093826147	6.31E-05
ENSMUSG00000003099	Ppp5c	0.093603001	0.000509931
ENSMUSG00000017677	Wsb1	0.093374225	0.01947398
ENSMUSG00000026639	Lamb3	0.093370374	0.000145891
ENSMUSG00000058216	Gstp3	0.093211927	0.016410247
ENSMUSG00000032867	Fbxw8	0.092841962	0.001187139
ENSMUSG00000003809	Gcdh	0.092583599	2.44E-05
ENSMUSG00000024077	Strn	0.09256599	1.38E-06
ENSMUSG00000040433	Zbtb38	0.092442426	0.018278672
ENSMUSG00000037257	Aagab	0.092404579	0.000871688
ENSMUSG00000036693	Nop14	0.092080836	0.036894846
ENSMUSG00000019989	Enpp3	0.092033251	7.43E-76
ENSMUSG00000005078	Jkamp	0.092005575	0.004735836
ENSMUSG00000022450	Ndufa6	0.091911657	8.38E-10
ENSMUSG00000028064	Sema4a	0.091868029	4.52E-06
ENSMUSG00000035268	Pkig	0.09177235	0.000960297
ENSMUSG00000060427	Zfp868	0.091705673	0.0011826
ENSMUSG00000038014	Fam120a	0.091455828	1.36E-07
ENSMUSG00000025060	Slk	0.091359931	9.32E-05
ENSMUSG00000005893	Nr2c2	0.091239177	0.000260491
ENSMUSG00000063683	Glyat	0.09122166	0.034896114
ENSMUSG00000049421	Zfp260	0.091166653	0.036163918
ENSMUSG00000040188	Scamp2	0.090917873	4.66E-11
ENSMUSG00000027303	Ptpra	0.090560988	0.048797284
ENSMUSG00000027864	Ptgfrn	0.090530832	4.43E-05
ENSMUSG00000049721	Gal3st1	0.090222893	0.014387553
ENSMUSG00000031995	St14	0.090217793	0.000734215
ENSMUSG00000021124	Vti1b	0.09016116	2.87E-06
ENSMUSG00000037706	Cd81	0.090067371	2.43E-08
ENSMUSG00000048490	Nrip1	0.089999419	0.003274848
ENSMUSG00000014763	Fam120b	0.089872758	0.038203567
ENSMUSG00000015889	Lta4h	0.089628062	0.037050981
ENSMUSG00000022272	Myo10	0.089469807	9.76E-06
ENSMUSG00000053600	Zfp472	0.089341056	0.011451514
ENSMUSG00000071652	Ints5	0.089183833	0.002222725
ENSMUSG00000040282	Cdin1	0.089106556	0.009280473
ENSMUSG00000004070	Hmox2	0.088990169	0.000829264
ENSMUSG00000025494	Sigirr	0.088918282	0.022849969
ENSMUSG00000026112	Coa5	0.088540426	0.037298003
ENSMUSG00000041025	Iffo2	0.088513179	0.049412373
ENSMUSG00000058135	Gstm1	0.088376995	2.04E-42
ENSMUSG00000010607	Pigyl	0.088376732	6.04E-07
ENSMUSG00000075595	Zfp652	0.088355762	0.003787764
ENSMUSG00000035021	Baz1a	0.087969868	2.82E-06
ENSMUSG00000038481	Cdk19	0.087127814	1.05E-09
ENSMUSG00000016427	Ndufa1	0.086992254	1.32E-17
ENSMUSG00000025153	Fasn	0.086855583	1.53E-10
ENSMUSG00000032175	Tyk2	0.086762157	0.01040779
ENSMUSG00000063354	Slc39a4	0.086747822	9.37E-11
ENSMUSG00000038762	Abcf1	0.086631086	2.16E-05
ENSMUSG00000074300	Smim31	0.086599252	8.27E-24
ENSMUSG00000032590	Apeh	0.086580001	0.004087097
ENSMUSG00000021754	Map3k1	0.086370024	2.75E-06
ENSMUSG00000038274	Fau	0.086333901	2.06E-05
ENSMUSG00000034203	Chchd4	0.086274448	1.84E-10
ENSMUSG00000014301	Pam16	0.086138645	1.93E-07
ENSMUSG00000010608	Rbm25	0.086055372	2.88E-13
ENSMUSG00000028677	Rnf220	0.086047642	0.007092492
ENSMUSG00000053253	Ndfip2	0.085331199	5.70E-05
ENSMUSG00000021866	Anxa11	0.085245197	3.67E-05
ENSMUSG00000034981	Parm1	0.085103306	1.69E-05
ENSMUSG00000030004	Nat8	0.085080894	1.04E-36
ENSMUSG00000029864	Gstk1	0.08500552	9.88E-47
ENSMUSG00000030465	Psd3	0.084752032	0.00067234
ENSMUSG00000022865	Cxadr	0.08473577	0.000513672
ENSMUSG00000024853	Sf3b2	0.084460484	1.24E-06
ENSMUSG00000025208	Mrpl43	0.0841879	2.77E-05
ENSMUSG00000042510	AA986860	0.084091512	3.90E-17
ENSMUSG00000028068	Iqgap3	0.083922817	0.049470033
ENSMUSG00000060224	Pyroxd2	0.083886667	0.030084456

ENSMUSG00000059518	Znhit1	-0.046739863	1.69E-06
ENSMUSG00000030172	Erc1	-0.046497713	0.002354117
ENSMUSG00000028803	Nipal3	-0.045963149	0.004483969
ENSMUSG00000021000	Mia2	-0.045255587	7.50E-18
ENSMUSG00000011960	Ccnt1	-0.045240579	0.0315752
ENSMUSG00000027944	Hax1	-0.0451999	0.002652016
ENSMUSG00000003345	Csnk1g2	-0.044870833	0.016400404
ENSMUSG00000047613	A430005L14	-0.044609327	5.59E-05
ENSMUSG00000034024	Cct2	-0.044409969	1.18E-06
ENSMUSG00000026014	Raph1	-0.044366647	4.70E-14
ENSMUSG00000073002	Vamp5	-0.044208525	6.61E-15
ENSMUSG00000034390	Cmip	-0.044192467	0.031417028
ENSMUSG00000025436	Atp23	-0.043760111	0.00545803
ENSMUSG00000023456	Tpi1	-0.043546417	9.89E-24
ENSMUSG00000028409	Smu1	-0.043490996	0.001324359
ENSMUSG00000044030	Irf2bp1	-0.043336385	8.83E-05
ENSMUSG00000006998	Psmd2	-0.042980921	0.000747486
ENSMUSG00000071041	Impdh2-ps	-0.042878722	0.008400777
ENSMUSG00000034687	Fras1	-0.042836219	1.89E-08
ENSMUSG00000021591	Glrx	-0.042731552	0.025919558
ENSMUSG00000031853	Map3k21	-0.042689912	0.000172357
ENSMUSG00000021905	Dph3	-0.042412425	5.29E-06
ENSMUSG00000024944	Arl2	-0.042213766	0.017726467
ENSMUSG00000025786	Zdhhc3	-0.041868119	0.000922699
ENSMUSG00000018286	Psmb6	-0.041720782	0.004856104
ENSMUSG00000027366	Sppl2a	-0.041444625	9.05E-07
ENSMUSG00000033671	Cep350	-0.041384813	0.003242197
ENSMUSG00000038462	Uqcrfs1	-0.041232256	5.70E-27
ENSMUSG00000039529	Atp8b1	-0.041134146	6.53E-23
ENSMUSG00000017688	Hnf4g	-0.041127379	1.83E-15
ENSMUSG00000026304	Rab17	-0.041092986	0.000775028
ENSMUSG00000065979	Cpped1	-0.040877766	3.43E-12
ENSMUSG00000030088	Aldh1l1	-0.040779501	1.18E-07
ENSMUSG00000064917	Snord138	-0.040726339	0.019286429
ENSMUSG00000029048	Rer1	-0.040715452	8.04E-05
ENSMUSG00000032548	Slco2a1	-0.04043701	1.49E-09
ENSMUSG00000038302	Afg1l	-0.04037428	0.016212652
ENSMUSG00000032092	Mpzl2	-0.040277912	0.000132731
ENSMUSG00000029999	Tgfa	-0.040233515	0.014346646
ENSMUSG00000026398	Nr5a2	-0.040138769	3.09E-20
ENSMUSG00000028557	Rnf11	-0.039949778	0.000444132
ENSMUSG00000038206	Fbxo8	-0.039543154	0.01149584
ENSMUSG00000048486	Fitm2	-0.039398017	0.021047305
ENSMUSG00000017607	Tns4	-0.039293363	9.60E-12
ENSMUSG00000020077	Srgn	-0.039287592	0.002054823
ENSMUSG00000029169	Dhx15	-0.038936337	0.00010504
ENSMUSG00000022095	Fhip2b	-0.038661673	0.026192841
ENSMUSG00000025495	Ptdss2	-0.038602174	0.025576878
ENSMUSG00000056228	Cars2	-0.038586467	0.019777922
ENSMUSG00000074884	Serf2	-0.038559996	4.23E-14
ENSMUSG00000084786	Ubl5	-0.038408447	7.86E-05
ENSMUSG00000020184	Mdm2	-0.03814221	1.35E-06
ENSMUSG00000050174	Nudt6	-0.038129691	9.68E-05
ENSMUSG00000053119	Chmp3	-0.037616556	0.001207157
ENSMUSG00000022148	Fyb	-0.037427591	9.61E-13
ENSMUSG00000037949	Ano10	-0.0374171	0.000206709
ENSMUSG00000090213	Peds1	-0.03739568	0.000367741
ENSMUSG00000028639	Ybx1	-0.037317872	0.006788561
ENSMUSG00000034108	Ccs	-0.037286802	0.005977972
ENSMUSG00000031916	Cog8	-0.037254278	0.000104471
ENSMUSG00000032373	Car12	-0.037118853	5.32E-12
ENSMUSG00000032624	Eml4	-0.037102451	0.001590207
ENSMUSG00000039782	Cpeb2	-0.036710792	0.015669224
ENSMUSG00000058076	Sdhc	-0.036676821	1.88E-22
ENSMUSG00000053965	Pde5a	-0.03665179	1.29E-20
ENSMUSG00000028152	Tspan5	-0.036649992	0.00851043
ENSMUSG00000000303	Cdh1	-0.036401832	1.46E-10
ENSMUSG00000003809	Gcdh	-0.036183911	2.44E-05
ENSMUSG00000107002	Ncbp2as2	-0.035807622	0.002692275
ENSMUSG00000031776	Arl2bp	-0.035705652	0.049113022
ENSMUSG00000020407	Upp1	-0.03568549	2.39E-33
ENSMUSG00000066150	Slc31a1	-0.035567045	4.12E-05
ENSMUSG00000028532	Cachd1	-0.035378441	9.23E-17
ENSMUSG00000033918	Parl	-0.035327974	0.005554182
ENSMUSG00000000171	Sdhd	-0.035015066	1.41E-05
ENSMUSG00000026781	Acbd5	-0.035009692	3.81E-19
ENSMUSG00000037419	Endod1	-0.03464573	1.20E-21
ENSMUSG00000036398	Ppp1r11	-0.034633243	6.99E-10
ENSMUSG00000024902	Mrpl11	-0.034544812	4.10E-06
ENSMUSG00000025381	Cnpy2	-0.034467192	0.034034709
ENSMUSG00000039835	Nhsl1	-0.034360838	6.04E-05
ENSMUSG00000074794	Arrdc3	-0.034151135	7.20E-06
ENSMUSG00000061207	Stk19	-0.034058697	0.020311805
ENSMUSG00000028394	Pole3	-0.033998876	0.002159169
ENSMUSG00000034247	Plekhm1	-0.033768634	0.007364716
ENSMUSG00000014444	Piezo1	-0.033690423	8.31E-06
ENSMUSG00000021371	Mcur1	-0.033339647	0.001955254
ENSMUSG00000021187	Tc2n	-0.033241477	0.001350529
ENSMUSG00000003037	Rab8a	-0.033172409	3.60E-12
ENSMUSG00000027195	Hsd17b12	-0.033159079	0.001033397
ENSMUSG00000040728	Esrp1	-0.033150061	3.82E-13
ENSMUSG00000054091	1810037I17R	-0.032947834	5.97E-06
ENSMUSG00000049799	Lrrc19	-0.032854601	7.24E-08
ENSMUSG00000032599	Ip6k2	-0.032839941	2.63E-05
ENSMUSG00000022136	Dnajc3	-0.03275465	0.000116484
ENSMUSG00000037685	Atp8a1	-0.032670462	0.003905009
ENSMUSG00000027381	Bcl2l11	-0.032394684	0.000242631
ENSMUSG00000018752	Tnfsfm13	-0.032393187	0.009471519
ENSMUSG00000051256	Jagn1	-0.032362898	0.012640472
ENSMUSG00000067847	Romo1	-0.032291199	0.000122598
ENSMUSG00000024583	Txnl1	-0.032281372	0.004215918
ENSMUSG00000086914	Gm16124	-0.032190658	0.004556121
ENSMUSG00000045005	Fzd5	-0.03208999	8.16E-18
ENSMUSG00000039630	Hnrnpu	-0.032086548	4.44E-06
ENSMUSG00000039405	Prss23	-0.031996159	1.00E-37
ENSMUSG00000025059	Gk	-0.031714794	0.000883349
ENSMUSG00000028382	Ptbp3	-0.031708105	2.54E-10
ENSMUSG00000028749	Pla2g2f	-0.031523018	0.01057116
ENSMUSG00000021772	Nkiras1	-0.031340223	1.04E-14
ENSMUSG00000085042	Abhd11os	-0.03108265	3.50E-06
ENSMUSG00000074170	Plekhf1	-0.031064371	7.76E-10
ENSMUSG00000042680	Garem1	-0.030872753	7.73E-22
ENSMUSG00000039461	Tcta	-0.030865059	4.71E-07
ENSMUSG00000052151	Plpp2	-0.030524267	0.000126081
ENSMUSG00000074071	Fam169b	-0.030073719	0.01250621
ENSMUSG00000059995	Atxn7l3	-0.029972184	0.016422899
ENSMUSG00000026175	Vil1	-0.029733537	0.003888009
ENSMUSG00000038013	Wipf2	-0.02962735	0.000782135
ENSMUSG00000029207	Apbb2	-0.029610642	8.95E-06
ENSMUSG00000038615	Nfe2l1	-0.02932314	3.46E-06
ENSMUSG00000037815	Ctnna1	-0.029107648	2.52E-07
ENSMUSG00000028841	Cnksr1	-0.028796094	0.020355098

ENSMUSG00000015243	Abca1	0.082831311	8.63E-48
ENSMUSG00000025351	Cd63	0.082649435	1.98E-05
ENSMUSG00000038225	Primpol	0.082547993	0.009959751
ENSMUSG00000033075	Senp1	0.082462838	2.92E-05
ENSMUSG00000037110	Ralgapa2	0.082390985	4.29E-05
ENSMUSG00000021488	Nsd1	0.08235641	1.82E-05
ENSMUSG00000042178	Armc5	0.082350338	4.35E-05
ENSMUSG00000031197	Vbp1	0.082281589	0.04345514
ENSMUSG00000028392	Bspry	0.081956064	7.67E-14
ENSMUSG00000034022	Cpsf1	0.081928918	1.10E-05
ENSMUSG00000015289	Lage3	0.081848195	0.001464773
ENSMUSG00000035258	Abi3bp	0.081789652	1.42E-12
ENSMUSG00000022265	Ank	0.081672655	2.17E-11
ENSMUSG00000026434	Nucks1	0.081468174	3.56E-12
ENSMUSG00000027108	Ola1	0.081320629	0.036211525
ENSMUSG00000017146	Brca1	0.081320173	0.019746485
ENSMUSG00000048109	Rbm15	0.081269852	0.035111488
ENSMUSG00000028274	Rngtt	0.081205322	0.003045526
ENSMUSG00000015501	Hivep2	0.081188466	0.001781606
ENSMUSG00000097318	1700007L15I	0.081132535	0.000497488
ENSMUSG00000092517	Art2a	0.080819276	0.000299797
ENSMUSG00000068299	Nat8f4	0.080695826	0.000486813
ENSMUSG00000071203	Naip5	0.080690707	1.70E-29
ENSMUSG00000035704	Alg8	0.080573055	0.007777756
ENSMUSG00000066324	Bpnt2	0.080156566	1.41E-17
ENSMUSG00000038650	Rnh1	0.080130068	5.83E-07
ENSMUSG00000035212	Leprot	0.080109507	0.044354991
ENSMUSG00000026946	Nmi	0.080106938	0.003139158
ENSMUSG00000030738	Eif3c	0.079598024	3.21E-15
ENSMUSG00000039105	Atp6v1g1	0.079449303	0.016332964
ENSMUSG00000010057	Nprl2	0.079442821	0.00035654
ENSMUSG00000049439	Cyp20a1	0.079387182	0.005286484
ENSMUSG00000038633	Degs1	0.079174234	1.96E-14
ENSMUSG00000060679	Mrps9	0.078984872	0.000227434
ENSMUSG00000033335	Dnm2	0.07891627	1.43E-08
ENSMUSG00000001755	Coasy	0.078813402	4.08E-06
ENSMUSG00000023723	Mrps23	0.078807927	3.64E-10
ENSMUSG00000078853	Igtp	0.078485123	3.20E-24
ENSMUSG00000037995	Igsf9	0.07812798	7.98E-09
ENSMUSG00000025782	Taf3	0.077984459	0.000248451
ENSMUSG00000060149	BC002059	0.07760568	0.001291087
ENSMUSG00000005148	Klf5	0.077393346	3.77E-15
ENSMUSG00000045107	Saysd1	0.077274593	0.014292265
ENSMUSG00000045624	Esf1	0.077149995	4.31E-05
ENSMUSG00000041040	Fam117b	0.076781457	1.25E-16
ENSMUSG00000049038	Mterf2	0.076688913	9.87E-15
ENSMUSG00000052459	Atp6v1a	0.076578418	8.19E-05
ENSMUSG00000005506	Celf1	0.076550886	4.60E-06
ENSMUSG00000033707	Lrrc24	0.076505625	0.01803907
ENSMUSG00000071078	Nr2c2ap	0.076381469	0.033620746
ENSMUSG00000021993	Mipep	0.076367972	0.017466762
ENSMUSG00000038642	Ctss	0.076204108	0.000338135
ENSMUSG00000076437	Selenoh	0.076001569	0.046997051
ENSMUSG00000034919	Ttc22	0.075811954	0.014223719
ENSMUSG00000038976	Ppp1r9b	0.075776932	0.005901646
ENSMUSG00000019872	Smpdl3a	0.075577682	0.000144801
ENSMUSG00000053453	Thoc7	0.075488882	6.26E-06
ENSMUSG00000031592	Pcm1	0.075435685	0.029111456
ENSMUSG00000023973	Cnpy3	0.07525966	0.008151847
ENSMUSG00000022553	Maf1	0.075218953	0.005006973
ENSMUSG00000032171	Pin1	0.075150302	0.005636551
ENSMUSG00000036377	Cracd	0.07512263	6.07E-10
ENSMUSG00000060538	Tmem219	0.075042146	1.36E-06
ENSMUSG00000022895	Ets2	0.074928631	1.85E-05
ENSMUSG00000024807	Syvn1	0.074833445	8.18E-05
ENSMUSG00000041028	Ghitm	0.07469638	1.64E-11
ENSMUSG00000031353	Rbbp7	0.074695556	0.001707272
ENSMUSG00000050323	Ndufaf6	0.074628826	0.013421324
ENSMUSG00000033813	Tcea1	0.07436707	0.002840411
ENSMUSG00000029192	Tbc1d14	0.074354364	0.0234826
ENSMUSG00000031600	Vps37a	0.073623587	0.001546549
ENSMUSG00000007836	Hnrnpa0	0.073457584	4.47E-16
ENSMUSG00000044285	Ubb-ps	0.07343503	6.86E-06
ENSMUSG00000078572	Ndufaf8	0.073330578	5.71E-06
ENSMUSG00000025781	Atp5c1	0.073007251	6.29E-11
ENSMUSG00000029865	Sval1	0.072923731	7.04E-11
ENSMUSG00000028793	Rnf19b	0.072881527	0.008520681
ENSMUSG00000029486	Mrpl1	0.072857787	3.95E-07
ENSMUSG00000076754	Trgv2	0.072851531	0.003399773
ENSMUSG00000042510	AA986860	0.072765039	3.90E-17
ENSMUSG00000027668	Mfn1	0.072719144	0.000894211
ENSMUSG00000024927	Rela	0.072492432	0.00771235
ENSMUSG00000018983	E2f2	0.072460583	4.46E-11
ENSMUSG00000019373	Cops3	0.072433969	3.94E-05
ENSMUSG00000028312	Smc2	0.072423038	8.11E-05
ENSMUSG00000026005	Rpe	0.072273047	1.11E-15
ENSMUSG00000045098	Kmt5b	0.071923187	0.003462674
ENSMUSG00000052962	Mrpl35	0.071803718	0.002259251
ENSMUSG00000027315	Spint1	0.071686088	0.000584911
ENSMUSG00000092203	1110038B12	0.071453542	0.000242296
ENSMUSG00000019773	Fbxo5	0.071346607	0.00520707
ENSMUSG00000025207	Sema4g	0.071299502	0.037365145
ENSMUSG00000106864	Gtf3c2	0.071292173	0.010612663
ENSMUSG00000040370	Etfrf1	0.071105441	3.04E-09
ENSMUSG00000055436	Srsf11	0.071067423	0.011034641
ENSMUSG00000073987	Ggh	0.070944717	0.001737258
ENSMUSG00000014763	Fam120b	0.070935009	0.038203567
ENSMUSG00000030718	Ppme1	0.070887362	0.000612051
ENSMUSG00000110841	Gpx4-ps2	0.070754871	2.81E-48
ENSMUSG00000037487	Ubr5	0.070747922	4.53E-10
ENSMUSG00000029017	Pmpcb	0.070741841	7.47E-06
ENSMUSG00000033055	Ankrd54	0.070735237	0.00018191
ENSMUSG00000021279	Cdc42bpb	0.070590437	1.76E-05
ENSMUSG00000025408	Ddit3	0.070553759	6.61E-07
ENSMUSG00000030057	Cnbp	0.070406352	0.000463455
ENSMUSG00000054469	Lclat1	0.070402574	5.31E-06
ENSMUSG00000034903	Cobll1	0.070368713	0.002850089
ENSMUSG00000032437	Stt3b	0.070061266	6.86E-07
ENSMUSG00000031557	Plekha2	0.069797623	0.012156349
ENSMUSG00000038260	Trpm4	0.069750274	0.000332361
ENSMUSG00000046707	Csnk2a2	0.069594254	0.006157097
ENSMUSG00000040242	Fgfr1op2	0.069466011	5.59E-05
ENSMUSG00000005882	Uqcc1	0.069390701	6.63E-13
ENSMUSG00000026154	Sdhaf4	0.069358232	6.99E-07
ENSMUSG00000025968	Ndufs1	0.069312844	5.29E-14
ENSMUSG00000032126	Hmbs	0.068945913	0.031491493
ENSMUSG00000017386	Traf4	0.068661366	0.004569192
ENSMUSG00000020328	Nudcd2	0.068588783	0.011324528
ENSMUSG00000038366	Lasp1	0.068566618	1.60E-05
ENSMUSG00000055839	Elob	0.068339854	0.019982045
ENSMUSG00000027787	Nmd3	0.068069419	0.001192276
ENSMUSG00000031381	Piga	0.067980068	0.049395139

ENSMUSG00000071076	Jund	-0.036483154	4.33E-07
ENSMUSG00000038034	Igsf8	-0.036243799	0.003199626
ENSMUSG00000057963	Itpk1	-0.036231921	0.000434715
ENSMUSG00000021936	Mapk8	-0.036092566	2.08E-17
ENSMUSG00000021583	Erap1	-0.03599518	0.02423143
ENSMUSG00000019961	Tmpo	-0.035864981	0.049233269
ENSMUSG00000029147	Ppm1g	-0.035749538	0.029605337
ENSMUSG00000037606	Osbpl5	-0.035710005	0.000846162
ENSMUSG00000008035	Mid1ip1	-0.035644575	0.000665654
ENSMUSG00000020190	Mknk2	-0.035114087	0.00037279
ENSMUSG00000028843	Sh3bgrl3	-0.0346052	8.00E-07
ENSMUSG00000028847	Trappc3	-0.034575322	0.024835519
ENSMUSG00000022884	Eif4a2	-0.034299433	1.79E-08
ENSMUSG00000020577	Tspan13	-0.033870338	1.14E-16
ENSMUSG00000042613	Pbxip1	-0.033608514	0.010630951
ENSMUSG00000092417	Gpank1	-0.033538899	3.31E-09
ENSMUSG00000001847	Rac1	-0.033422135	4.47E-15
ENSMUSG00000071655	Ubxn1	-0.033206405	0.033221993
ENSMUSG00000050621	Rps27rt	-0.03318853	0.000578538
ENSMUSG00000021947	Cryl1	-0.033032693	1.26E-16
ENSMUSG00000042595	Fam199x	-0.032759997	3.04E-05
ENSMUSG00000060477	Irak2	-0.032619058	0.009001843
ENSMUSG00000070473	Cldn3	-0.032386932	0.008968959
ENSMUSG00000015575	Atp6v0e	-0.032243016	4.88E-10
ENSMUSG00000068141	Gm10232	-0.032105024	0.031417028
ENSMUSG00000057400	Ces1c	-0.032038821	1.00E-05
ENSMUSG00000043061	Tmem18	-0.031935247	0.00010538
ENSMUSG00000053768	Chchd3	-0.031852417	9.25E-16
ENSMUSG00000032062	2310030G06	-0.031523491	0.007489876
ENSMUSG00000020982	Nemf	-0.031419412	0.034162871
ENSMUSG00000033444	Specc1l	-0.03128924	0.00398425
ENSMUSG00000043445	Pgp	-0.031265504	1.75E-10
ENSMUSG00000034640	Tiparp	-0.030926059	8.23E-10
ENSMUSG00000061613	U2af1	-0.030727317	8.87E-09
ENSMUSG00000041685	Fcho2	-0.029946859	0.013594725
ENSMUSG00000030432	Rpl28	-0.029790727	1.69E-05
ENSMUSG00000006736	Tspan31	-0.029720459	5.52E-05
ENSMUSG00000040209	Zfp704	-0.029571342	1.79E-08
ENSMUSG00000021996	Esd	-0.029413689	8.36E-09
ENSMUSG00000027173	Depdc7	-0.029297625	0.000519157
ENSMUSG00000087107	AI662270	-0.029012611	0.049609771
ENSMUSG00000089809	A930011G23	-0.028973292	0.000130203
ENSMUSG00000023235	Ccl25	-0.028758909	0.000194839
ENSMUSG00000041571	Selenow	-0.027851908	1.28E-07
ENSMUSG00000068706	Gm10250	-0.027687255	1.26E-20
ENSMUSG00000024960	Plcb3	-0.027616281	0.002032081
ENSMUSG00000002228	Ppm1j	-0.027444164	1.31E-41
ENSMUSG00000021408	Ripk1	-0.027432384	0.019682395
ENSMUSG00000052837	Junb	-0.027232774	0.000411437
ENSMUSG00000031059	Ndufb11	-0.027184464	5.44E-08
ENSMUSG00000030869	Ndufab1	-0.027061484	4.47E-09
ENSMUSG00000027673	Ndufb5	-0.026771381	3.43E-09
ENSMUSG00000034667	Xpot	-0.0261608	0.003819374
ENSMUSG00000052456	Get3	-0.026099661	0.043831375
ENSMUSG00000021102	Glrx5	-0.026000054	3.80E-13
ENSMUSG00000091387	Gcnt4	-0.025906934	8.14E-08
ENSMUSG00000047824	Pygo2	-0.025739337	0.000122924
ENSMUSG00000034285	Nipsnap1	-0.025539007	9.80E-32
ENSMUSG00000036078	Sigmar1	-0.02524691	0.009537598
ENSMUSG00000040618	Pck2	-0.025187562	0.029008988
ENSMUSG00000036639	Nudt1	-0.025007872	0.001347641
ENSMUSG00000022463	Srebf2	-0.024949519	8.49E-05
ENSMUSG00000005354	Txn2	-0.02485418	1.66E-08
ENSMUSG00000026961	Lrrc26	-0.024731555	0.000127622
ENSMUSG00000028059	Arhgef2	-0.024725194	0.00358307
ENSMUSG00000024846	Cst6	-0.024706921	0.041240361
ENSMUSG00000023921	Mmut	-0.024610588	0.00177606
ENSMUSG00000004446	Bid	-0.024373605	9.23E-09
ENSMUSG00000013701	Timm23	-0.024101282	0.003236373
ENSMUSG00000036199	Ndufa13	-0.023988629	7.31E-15
ENSMUSG00000097101	1810034E14I	-0.023849008	0.008513972
ENSMUSG00000052428	Tmco1	-0.023694264	0.00935203
ENSMUSG00000080893	Ndufa12-ps	-0.023296732	6.81E-12
ENSMUSG00000029596	Sdsl	-0.022902227	1.76E-06
ENSMUSG00000026159	Agfg1	-0.02258297	0.000340741
ENSMUSG00000040957	Cables1	-0.022543838	8.32E-08
ENSMUSG00000051950	B3glct	-0.022448586	1.28E-07
ENSMUSG00000036860	Mrpl55	-0.021998126	7.27E-11
ENSMUSG00000036123	Slc9a3	-0.021870579	1.95E-16
ENSMUSG00000024096	Ralbp1	-0.021846907	0.003563409
ENSMUSG00000029655	N4bp2l2	-0.0218165	2.68E-06
ENSMUSG00000030824	Nucb1	-0.021790694	0.000220689
ENSMUSG00000057388	Mrpl18	-0.021480292	1.75E-09
ENSMUSG00000060227	Golm2	-0.021364692	3.00E-05
ENSMUSG00000070426	Rnf121	-0.021073114	3.89E-10
ENSMUSG00000034154	Ino80	-0.0209827	0.00216492
ENSMUSG00000028901	Gmeb1	-0.020755784	0.030644703
ENSMUSG00000037921	Ddx60	-0.020725321	1.90E-27
ENSMUSG00000022253	Nadk2	-0.020667369	4.96E-14
ENSMUSG00000043760	Pkhd1	-0.020567767	0.000845579
ENSMUSG00000023039	Krt7	-0.020502333	0.00017484
ENSMUSG00000056035	Cyp3a11	-0.020182486	1.76E-88
ENSMUSG00000026304	Rab17	-0.020147468	0.000775028
ENSMUSG00000024069	Slc30a6	-0.02008883	0.000971218
ENSMUSG00000062995	Ica1	-0.019976953	8.90E-05
ENSMUSG00000020641	Rsad2	-0.01954149	2.51E-13
ENSMUSG00000031796	Cfap20	-0.019513824	0.00027929
ENSMUSG00000027356	Fermt1	-0.019311606	1.43E-05
ENSMUSG00000046410	Kcnk6	-0.01910312	7.37E-05
ENSMUSG00000073802	Cdkn2b	-0.018652281	4.20E-05
ENSMUSG00000016256	Ctsz	-0.018452479	0.003282074
ENSMUSG00000056076	Eif3b	-0.018282757	1.08E-06
ENSMUSG00000047843	Bri3	-0.018190618	0.001754909
ENSMUSG00000079426	Arpc4	-0.01816622	0.000422469
ENSMUSG00000042492	Tbc1d10b	-0.017938511	0.031622732
ENSMUSG00000057880	Abat	-0.017799824	6.81E-12
ENSMUSG00000030051	Aplf	-0.017748932	0.034913088
ENSMUSG00000021577	Sdha	-0.017540604	3.75E-05
ENSMUSG00000016495	Plgrkt	-0.017408633	0.009772702
ENSMUSG00000097184	4632428C04	-0.017397529	0.009779296
ENSMUSG00000015944	Castor2	-0.017309279	0.021427005
ENSMUSG00000032294	Pkm	-0.017304345	8.92E-52
ENSMUSG00000055137	Sugct	-0.017155369	0.00421901
ENSMUSG00000017615	Tnfaip1	-0.017077718	0.027753045
ENSMUSG00000027078	Ube2l6	-0.016677433	5.37E-13
ENSMUSG00000042978	Sbk1	-0.016619879	0.002229498
ENSMUSG00000018604	Tbx3	-0.016576345	0.000400006
ENSMUSG00000020275	Rel	-0.016464542	0.026320873
ENSMUSG00000078154	Gm12184	-0.016152825	0.018979183
ENSMUSG00000050213	Snip1	-0.015974075	0.031761239
ENSMUSG00000042699	Dhx9	-0.015563086	0.000326848
ENSMUSG00000046861	Hectd3	-0.015554824	0.004531913

ENSMUSG00000021240	Abcd4	0.083833219	0.00046332
ENSMUSG00000039826	Trub2	0.083783053	4.57E-14
ENSMUSG00000022353	Mtss1	0.083417	0.026569206
ENSMUSG00000027668	Mfn1	0.083398712	0.000894211
ENSMUSG00000032481	Smarcc1	0.08337909	1.50E-06
ENSMUSG00000022346	Myc	0.083273984	0.000512097
ENSMUSG00000038495	Otud7b	0.083144155	0.035739845
ENSMUSG00000057738	Sptan1	0.0830172	3.54E-09
ENSMUSG00000120113	Fam120aos	0.08279681	0.005286484
ENSMUSG00000026623	Lpgat1	0.082432256	2.14E-20
ENSMUSG00000034042	Gpbp1l1	0.08239878	1.66E-07
ENSMUSG00000020516	Rps6kb1	0.08236459	0.004630545
ENSMUSG00000030785	Cox6a2	0.082335641	0.023413671
ENSMUSG00000040370	Etfrf1	0.082262016	3.04E-09
ENSMUSG00000027422	Rrbp1	0.08219359	1.10E-06
ENSMUSG00000001665	Gstt3	0.081647132	0.004129941
ENSMUSG00000095026	Gm3336	0.081406329	3.89E-08
ENSMUSG00000100153	Ppp1ccb	0.08117331	0.003682118
ENSMUSG00000002661	Alkbh7	0.081058623	8.54E-05
ENSMUSG00000020440	Arf5	0.080948893	4.07E-29
ENSMUSG00000030231	Plekha5	0.08064519	0.002866355
ENSMUSG00000020709	Adap2	0.080595246	0.029880846
ENSMUSG00000018677	Slc25a39	0.080487542	0.004225507
ENSMUSG00000030339	Ltbr	0.080420446	3.95E-20
ENSMUSG00000027879	Sec22b	0.080413404	1.14E-21
ENSMUSG00000037710	Cisd1	0.080153736	4.48E-13
ENSMUSG00000000278	Scpep1	0.079838778	3.18E-07
ENSMUSG00000026356	Dars	0.079312179	0.001057081
ENSMUSG00000026796	Niban2	0.079254168	2.69E-07
ENSMUSG00000039523	Cep104	0.079083777	0.010270764
ENSMUSG00000006818	Sod2	0.079020086	0.000144226
ENSMUSG00000015501	Hivep2	0.078849102	0.001781606
ENSMUSG00000056228	Cars2	0.078762647	0.019777922
ENSMUSG00000025102	3110040N11	0.078299986	0.010951921
ENSMUSG00000021693	Kif2a	0.078295691	0.039418915
ENSMUSG00000067297	Ifit1bl2	0.078163715	3.38E-17
ENSMUSG00000032092	Mpzl2	0.077658105	0.000132731
ENSMUSG00000038267	Slc22a23	0.077556754	4.78E-10
ENSMUSG00000033429	Mcee	0.077277764	0.008082177
ENSMUSG00000019558	Slc6a8	0.077213297	0.016400404
ENSMUSG00000033918	Parl	0.077181968	0.005554182
ENSMUSG00000050732	Vamp8	0.077034022	3.69E-08
ENSMUSG00000022257	Laptm4b	0.07690283	3.20E-10
ENSMUSG00000041607	Mbp	0.076887286	0.002866831
ENSMUSG00000019578	Ubxn6	0.076585495	0.002340762
ENSMUSG00000002812	Flii	0.07657292	9.31E-07
ENSMUSG00000038766	Gabpb2	0.076483538	6.91E-08
ENSMUSG00000001632	Brpf1	0.075943602	2.33E-06
ENSMUSG00000019907	Ppp1r12a	0.075873426	5.98E-08
ENSMUSG00000104445	Rhbg	0.075811203	0.005445631
ENSMUSG00000035011	Zbtb7a	0.075511859	4.68E-11
ENSMUSG00000004264	Phb2	0.075045769	0.002246801
ENSMUSG00000032384	Csnk1g1	0.075038241	0.00099799
ENSMUSG00000024949	Sf1	0.074468798	0.001119242
ENSMUSG00000042348	Arl15	0.0744169	1.57E-06
ENSMUSG00000062300	Nectin2	0.074356605	0.00033336
ENSMUSG00000021707	Dhfr	0.074092448	1.89E-10
ENSMUSG00000046562	Unc119b	0.073986414	0.000786522
ENSMUSG00000035506	Slc12a8	0.073973067	0.000807097
ENSMUSG00000029776	Hibadh	0.073865194	7.77E-08
ENSMUSG00000041375	Ccdc9	0.073822186	0.014800023
ENSMUSG00000024036	Slc37a1	0.073794814	2.95E-05
ENSMUSG00000028433	Ubap2	0.073647582	3.01E-10
ENSMUSG00000030084	Plxna1	0.073620685	7.50E-05
ENSMUSG00000075304	Sp5	0.073487078	3.40E-06
ENSMUSG00000038615	Nfe2l1	0.07340532	3.46E-06
ENSMUSG00000018736	Ndel1	0.073247009	0.006042998
ENSMUSG00000030816	Rnf40	0.071982138	0.000355903
ENSMUSG00000025825	Iscu	0.071899606	0.036793326
ENSMUSG00000017978	Cadps2	0.071796471	0.028450328
ENSMUSG00000024959	Bad	0.071558949	3.71E-10
ENSMUSG00000032883	Acsl3	0.071501731	4.29E-07
ENSMUSG00000067787	Blcap	0.071408323	0.009711665
ENSMUSG00000030265	Kras	0.070833381	4.44E-09
ENSMUSG00000021676	Iqgap2	0.070151615	1.08E-07
ENSMUSG00000024413	Npc1	0.070030334	0.000284656
ENSMUSG00000007564	Ppp2r1a	0.069900935	0.010690033
ENSMUSG00000097415	AU020206	0.06987241	0.003958051
ENSMUSG00000039983	Ccdc32	0.069822858	0.000867627
ENSMUSG00000024563	Smad2	0.069728321	0.009196271
ENSMUSG00000040570	Rundc3b	0.069675359	0.043818152
ENSMUSG00000027684	Mecom	0.069543693	3.35E-07
ENSMUSG00000030795	Fus	0.069444098	7.76E-05
ENSMUSG00000026870	Cutal	0.069289146	9.03E-08
ENSMUSG00000036646	Man1b1	0.06919704	0.009877911
ENSMUSG00000028029	Aimp1	0.068861747	0.023165591
ENSMUSG00000059323	Tonsl	0.068853708	0.004696182
ENSMUSG00000020167	Tcf3	0.068849649	0.000126874
ENSMUSG00000002550	Uck1	0.068585848	5.04E-08
ENSMUSG00000021636	Marveld2	0.068273142	0.000651315
ENSMUSG00000050071	Bex1	0.067762295	1.78E-33
ENSMUSG00000085795	Zfp703	0.066633322	2.04E-06
ENSMUSG00000037197	Rbm17	0.066615946	0.012536388
ENSMUSG00000020451	Limk2	0.066197722	7.30E-07
ENSMUSG00000029169	Dhx15	0.066178984	0.00010504
ENSMUSG00000059518	Znhit1	0.065902518	1.69E-06
ENSMUSG00000027936	Crtc2	0.065895589	0.041129844
ENSMUSG00000024805	Pcgf5	0.065633236	9.92E-05
ENSMUSG00000069895	Atxn1l	0.065527184	0.015630553
ENSMUSG00000037896	Rcor1	0.065132886	7.09E-06
ENSMUSG00000036093	Arl5a	0.064877531	2.32E-20
ENSMUSG00000006373	Pgrmc1	0.06483251	6.76E-16
ENSMUSG00000028670	Lypla2	0.064797676	0.008747152
ENSMUSG00000021079	Timm9	0.064763417	3.00E-10
ENSMUSG00000061393	Acvr2b	0.064730801	0.010006049
ENSMUSG00000026939	Tmem141	0.064648325	0.019720579
ENSMUSG00000027610	Gss	0.063998341	0.000726248
ENSMUSG00000079419	Ms4a6c	0.063878886	0.020961664
ENSMUSG00000061461	Smim20	0.063369094	3.31E-05
ENSMUSG00000062397	Zfp706	0.063020808	0.002298702
ENSMUSG00000037236	Matr3	0.063007508	9.86E-05
ENSMUSG00000028466	Creb3	0.062986552	3.25E-12
ENSMUSG00000027937	Jtb	0.062694608	4.35E-05
ENSMUSG00000041126	H2az2	0.062629926	0.000245425
ENSMUSG00000051579	Tceal8	0.06247622	1.16E-18
ENSMUSG00000028557	Rnf11	0.062405355	0.000444132
ENSMUSG00000045455	Gm9797	0.062297795	0.005001184
ENSMUSG00000001247	Lsr	0.062136735	3.67E-16
ENSMUSG00000024158	Hagh	0.062099966	2.05E-15
ENSMUSG00000005609	Ctr9	0.061613963	0.023972146
ENSMUSG00000028262	Clca3a2	0.061502223	6.87E-11
ENSMUSG00000024740	Ddb1	0.061332022	1.22E-06

ENSMUSG00000056116	H2-T22	-0.028704397	0.009414367
ENSMUSG00000028780	Sema3c	-0.028482551	4.13E-05
ENSMUSG00000022560	Slc52a2	-0.028262586	0.013807886
ENSMUSG00000021458	Aopep	-0.028201321	0.047070601
ENSMUSG00000018604	Tbx3	-0.027875581	0.000400006
ENSMUSG00000029189	Sel1l3	-0.027833618	6.42E-14
ENSMUSG00000024853	Sf3b2	-0.02775563	1.24E-06
ENSMUSG00000027630	Tbl1xr1	-0.027431012	5.17E-08
ENSMUSG00000020196	Cabin1	-0.027095037	0.016519329
ENSMUSG00000018501	Ncor1	-0.026964225	0.013862877
ENSMUSG00000020840	Blmh	-0.026791617	1.17E-06
ENSMUSG00000003814	Calr	-0.026625737	0.000231859
ENSMUSG00000022010	Tsc22d1	-0.026508336	1.25E-21
ENSMUSG00000028519	Dab1	-0.026325362	0.041862076
ENSMUSG00000074195	Clca4b	-0.026251974	2.05E-12
ENSMUSG00000031948	Kars	-0.02621501	0.013622843
ENSMUSG00000002329	Mdp1	-0.025966368	7.05E-12
ENSMUSG00000038014	Fam120a	-0.025873804	1.36E-07
ENSMUSG00000044857	Lemd2	-0.025741313	0.003597092
ENSMUSG00000039470	Zdhhc2	-0.025701921	6.57E-08
ENSMUSG00000031812	Map1lc3b	-0.02563786	0.049500279
ENSMUSG00000036968	Cnpy4	-0.025501285	0.046330648
ENSMUSG00000068011	Mkrn2os	-0.025484132	0.00103785
ENSMUSG00000028228	Cpne3	-0.025408784	0.040412823
ENSMUSG00000044763	Trmt10c	-0.025315663	0.007005825
ENSMUSG00000057541	Pus7	-0.024984565	0.040183167
ENSMUSG00000038160	Atg5	-0.024941574	1.37E-10
ENSMUSG00000033186	Mzt1	-0.024860991	0.032357396
ENSMUSG00000070394	Tmem256	-0.024770266	4.29E-05
ENSMUSG00000021278	Amn	-0.024603308	5.28E-09
ENSMUSG00000037896	Rcor1	-0.024576968	7.09E-06
ENSMUSG00000037492	Zmat4	-0.024499247	2.63E-05
ENSMUSG00000047454	Gphn	-0.024420308	6.44E-06
ENSMUSG00000029672	Fam3c	-0.024388295	0.006365424
ENSMUSG00000023175	Bsg	-0.024329642	1.98E-08
ENSMUSG00000029580	Actb	-0.023904604	4.38E-06
ENSMUSG00000030465	Psd3	-0.023902561	0.00067234
ENSMUSG00000044408	Sptssa	-0.023832706	4.42E-06
ENSMUSG00000045287	Rtn4rl1	-0.023770798	5.24E-22
ENSMUSG00000013539	Tango2	-0.023606816	0.001895881
ENSMUSG00000018446	C1qbp	-0.023528099	0.022814218
ENSMUSG00000020163	Uqcr11	-0.023329348	8.06E-14
ENSMUSG00000042323	Pbrm1	-0.023254554	0.000223559
ENSMUSG00000031924	Cyb5b	-0.023245965	9.96E-33
ENSMUSG00000029513	Prkab1	-0.023003693	9.09E-20
ENSMUSG00000030866	Ern2	-0.022988803	9.57E-10
ENSMUSG00000061665	Cd2ap	-0.022557692	2.35E-19
ENSMUSG00000040170	Fmo2	-0.022529924	6.39E-09
ENSMUSG00000015575	Atp6v0e	-0.022233849	4.88E-10
ENSMUSG00000025246	Tbl1x	-0.022122932	5.13E-07
ENSMUSG00000058655	Eif4b	-0.021820267	7.57E-10
ENSMUSG00000045980	Tmem104	-0.021803566	0.030993454
ENSMUSG00000001280	Sp1	-0.02172373	3.30E-11
ENSMUSG00000028962	Slc4a2	-0.021720355	0.037509467
ENSMUSG00000006010	Odr4	-0.021394745	0.000816903
ENSMUSG00000038807	Rap1gap2	-0.021084647	9.92E-19
ENSMUSG00000074639	Rdh16f2	-0.021075777	2.66E-25
ENSMUSG00000031010	Usp9x	-0.021036588	0.002400918
ENSMUSG00000025575	Cant1	-0.020935493	2.12E-16
ENSMUSG00000024360	Etf1	-0.020756491	0.001456417
ENSMUSG00000090946	Ccdc71l	-0.020635668	0.000734215
ENSMUSG00000027479	Mapre1	-0.02059516	3.98E-06
ENSMUSG00000037750	Fam222b	-0.020537909	3.70E-09
ENSMUSG00000002233	Rhoc	-0.020502928	1.26E-37
ENSMUSG00000028436	Dcaf12	-0.020429189	0.016255009
ENSMUSG00000038845	Phb	-0.020409786	4.52E-05
ENSMUSG00000069874	Irgm2	-0.020403567	2.24E-12
ENSMUSG00000030824	Nucb1	-0.020390695	0.000220689
ENSMUSG00000032456	Nmnat3	-0.020182482	4.39E-16
ENSMUSG00000027665	Pik3ca	-0.020107746	0.02841654
ENSMUSG00000025225	Nfkb2	-0.019996597	1.50E-06
ENSMUSG00000051147	Nat2	-0.019951388	2.05E-05
ENSMUSG00000059586	Nsmce2	-0.019763269	0.045720225
ENSMUSG00000017119	Nbr1	-0.019645318	0.000107037
ENSMUSG00000061613	U2af1	-0.019418582	8.87E-09
ENSMUSG00000009633	G0s2	-0.019259854	3.79E-12
ENSMUSG00000018927	Ccl6	-0.019147913	0.003641836
ENSMUSG00000022016	Akap11	-0.019146855	0.000294483
ENSMUSG00000058216	Gstp3	-0.019095652	0.016410247
ENSMUSG00000025226	Fbxl15	-0.018807302	1.19E-05
ENSMUSG00000026956	Uap1l1	-0.018688439	1.64E-13
ENSMUSG00000036860	Mrpl55	-0.018543247	7.27E-11
ENSMUSG00000029014	Dnajc2	-0.018520144	2.51E-05
ENSMUSG00000032376	Usp3	-0.018436978	0.00058818
ENSMUSG00000004897	Hdgf	-0.018370086	3.14E-10
ENSMUSG00000020027	Socs2	-0.018327386	1.42E-05
ENSMUSG00000033938	Ndufb7	-0.018163074	4.63E-14
ENSMUSG00000006715	Gmnn	-0.017856731	3.85E-07
ENSMUSG00000042359	Osbpl6	-0.017814477	1.13E-10
ENSMUSG00000038127	Ccdc50	-0.017527646	0.001485771
ENSMUSG00000001911	Nfix	-0.017254525	0.001474982
ENSMUSG00000024331	Dsc2	-0.017095934	0.000569235
ENSMUSG00000052681	Rap1b	-0.016853825	0.000408695
ENSMUSG00000016427	Ndufa1	-0.016749573	1.32E-17
ENSMUSG00000040433	Zbtb38	-0.01670267	0.018278672
ENSMUSG00000079427	Mthfsl	-0.01665957	0.000291267
ENSMUSG00000046756	Mrps7	-0.01630299	0.000987671
ENSMUSG00000038422	Hdhd3	-0.016205217	4.93E-26
ENSMUSG00000004099	Dnmt1	-0.015890705	0.000252785
ENSMUSG00000056153	Socs6	-0.015644604	2.79E-06
ENSMUSG00000027253	Lrp4	-0.015402949	0.000582014
ENSMUSG00000027099	Mtx2	-0.015145236	1.46E-08
ENSMUSG00000036371	Serbp1	-0.01489195	0.011213722
ENSMUSG00000032265	Tent5a	-0.014442351	0.000199618
ENSMUSG00000043510	Hscb	-0.014317546	0.000377612
ENSMUSG00000022677	Cep20	-0.01429679	0.000137983
ENSMUSG00000030774	Pak1	-0.014072277	7.49E-08
ENSMUSG00000055401	Fbxo6	-0.013984697	4.15E-06
ENSMUSG00000021112	Pals1	-0.01367646	0.009430858
ENSMUSG00000022024	Sugt1	-0.013620427	8.85E-05
ENSMUSG00000028179	Cth	-0.01323094	1.31E-08
ENSMUSG00000031767	Nudt7	-0.012485711	1.43E-21
ENSMUSG00000032328	Tmem30a	-0.012359049	1.68E-11
ENSMUSG00000035775	Krt20	-0.012324106	0.002424176
ENSMUSG00000055296	Tmem245	-0.012121693	0.011831041
ENSMUSG00000030652	Coq7	-0.012074049	0.001296421
ENSMUSG00000020843	Timm22	-0.012035737	0.012753338
ENSMUSG00000024677	Ms4a6b	-0.011645742	7.41E-05
ENSMUSG00000042348	Arl15	-0.011585338	1.57E-06
ENSMUSG00000029474	Rnf34	-0.011577799	0.00029908
ENSMUSG00000024158	Hagh	-0.011314419	2.05E-15
ENSMUSG00000029992	Gfpt1	-0.011275966	1.28E-32

ENSMUSG00000032059	Alg9	0.067827413	0.047208109
ENSMUSG00000027940	Tpm3	0.06769932	0.005704977
ENSMUSG00000096210	H1f0	0.067641217	0.000101065
ENSMUSG00000032827	Ppp1r9a	0.067628788	0.000412672
ENSMUSG00000024769	Cdc42bpg	0.06758951	5.10E-08
ENSMUSG00000035429	Ptprh	0.067190266	0.000245171
ENSMUSG00000024130	Abca3	0.067020938	2.72E-06
ENSMUSG00000002396	Ocel1	0.066688566	0.001401274
ENSMUSG00000022807	Osbpl11	0.066687579	0.007142108
ENSMUSG00000032366	Tpm1	0.066578636	0.000464979
ENSMUSG00000032526	Ss18l2	0.066494313	1.73E-08
ENSMUSG00000047409	Ctdspl	0.066351533	1.09E-27
ENSMUSG00000023908	Pkmyt1	0.065894857	0.010111192
ENSMUSG00000032590	Apeh	0.064884327	0.004087097
ENSMUSG00000039473	Ubn1	0.064867442	4.89E-08
ENSMUSG00000021693	Kif2a	0.064482602	0.039418915
ENSMUSG00000091957	Rps2-ps10	0.064252731	0.033964931
ENSMUSG00000039166	Akap7	0.063851387	0.002448445
ENSMUSG00000036856	Wnt4	0.063847919	0.03061777
ENSMUSG00000024056	Ndc80	0.063522876	0.007864052
ENSMUSG00000027397	Slc20a1	0.063422689	0.033783473
ENSMUSG00000026254	Eif4e2	0.063379309	1.33E-06
ENSMUSG00000032757	Bet1	0.063271376	3.57E-14
ENSMUSG00000028080	Lrba	0.063206934	0.019730174
ENSMUSG00000035004	Igsf6	0.063192679	0.027059533
ENSMUSG00000040822	1700123O20	0.063079464	0.001268452
ENSMUSG00000023915	Tnfrsf21	0.062950062	0.000575101
ENSMUSG00000025651	Uqcrc1	0.062777092	4.38E-07
ENSMUSG00000021930	Spryd7	0.062470895	6.51E-15
ENSMUSG00000029394	Cdk2ap1	0.062469312	8.14E-06
ENSMUSG00000032966	Fkbp1a	0.062427975	0.003710054
ENSMUSG00000071506	Tmem139	0.062331539	7.36E-11
ENSMUSG00000036202	Rif1	0.062091018	0.002047968
ENSMUSG00000023852	Chd1	0.061877807	0.002773712
ENSMUSG00000038611	Phrf1	0.0618704	0.024130871
ENSMUSG00000034088	Hdlbp	0.061853769	0.015555348
ENSMUSG00000020640	Itsn2	0.061800022	0.002532728
ENSMUSG00000029422	Rsrc2	0.061625924	0.016141346
ENSMUSG00000025135	Anapc11	0.06153858	0.003168019
ENSMUSG00000026399	Cd55	0.061536884	0.000128063
ENSMUSG00000024539	Ptpn2	0.061490339	0.005899217
ENSMUSG00000019952	Poc1b	0.061478705	3.67E-05
ENSMUSG00000037740	Mrps26	0.061063966	0.002335977
ENSMUSG00000089764	Gm16580	0.06074017	0.01650575
ENSMUSG00000042745	Id1	0.060737037	1.86E-18
ENSMUSG00000037361	Sf3b6	0.060716533	0.015154516
ENSMUSG00000033943	Mga	0.0605898	0.017890285
ENSMUSG00000021375	Kif13a	0.060467473	8.53E-14
ENSMUSG00000032932	Hspa13	0.060309984	0.002339371
ENSMUSG00000024683	Mrpl16	0.060295954	8.90E-05
ENSMUSG00000038274	Fau	0.059965031	2.06E-05
ENSMUSG00000021065	Fut8	0.059724467	3.85E-06
ENSMUSG00000057497	Fam136a	0.05935895	5.49E-10
ENSMUSG00000026277	Stk25	0.059296956	0.015602812
ENSMUSG00000032528	Vipr1	0.059234022	0.00043244
ENSMUSG00000032803	Cdv3	0.059024341	1.50E-10
ENSMUSG00000002043	Trappc6a	0.058974404	0.000292422
ENSMUSG00000024146	Cript	0.05892308	8.75E-09
ENSMUSG00000030339	Ltbr	0.058824023	3.95E-20
ENSMUSG00000031732	Phlpp2	0.058802241	3.19E-05
ENSMUSG00000002550	Uck1	0.05855883	5.04E-08
ENSMUSG00000052456	Get3	0.058206997	0.043831375
ENSMUSG00000021589	Rhobtb3	0.058148979	2.30E-30
ENSMUSG00000022946	Dop1b	0.057906582	0.003172722
ENSMUSG00000021764	Ndufs4	0.057802138	2.43E-08
ENSMUSG00000022956	Atp5o	0.057745864	9.09E-12
ENSMUSG00000039031	Arhgap18	0.057708853	0.007910639
ENSMUSG00000062270	Morf4l1	0.057584623	0.001867921
ENSMUSG00000025026	Add3	0.057526655	2.11E-17
ENSMUSG00000036501	Fam13b	0.05744798	0.04423346
ENSMUSG00000024067	Dpy30	0.057310642	0.007258447
ENSMUSG00000021036	Sptlc2	0.057289577	0.010630951
ENSMUSG00000030327	Necap1	0.05691053	4.90E-06
ENSMUSG00000024248	Cox7a2l	0.056887707	7.83E-05
ENSMUSG00000021216	Tubal3	0.05677645	8.18E-05
ENSMUSG00000031919	Tmed6	0.056693978	0.001023102
ENSMUSG00000036136	Fam110c	0.056633363	0.000604495
ENSMUSG00000066880	Zfp617	0.056623492	0.000423217
ENSMUSG00000029279	Brdt	0.056333722	0.003677289
ENSMUSG00000001542	Ell2	0.056241865	3.03E-06
ENSMUSG00000016257	Prelid3b	0.056196946	2.87E-08
ENSMUSG00000031103	Elf4	0.055886538	2.88E-06
ENSMUSG00000000708	Kat2b	0.055808106	0.00432081
ENSMUSG00000026303	Mlph	0.055787638	1.53E-09
ENSMUSG00000044026	Slc35g1	0.05562368	2.10E-06
ENSMUSG00000048897	Zfp710	0.055470981	9.67E-06
ENSMUSG00000039220	Ppp1r10	0.055412285	0.001025322
ENSMUSG00000006740	Kif5b	0.055411742	0.003931984
ENSMUSG00000060206	Zfp462	0.055248073	6.72E-05
ENSMUSG00000007815	Rhoa	0.055065556	0.007261257
ENSMUSG00000078942	Naip6	0.054617864	8.23E-07
ENSMUSG00000024742	Fen1	0.054566753	0.008477322
ENSMUSG00000029684	Wasl	0.054555556	2.74E-32
ENSMUSG00000032279	Idh3a	0.054535273	3.48E-17
ENSMUSG00000007670	Khsrp	0.05452605	4.83E-08
ENSMUSG00000031715	Smarca5	0.054501538	0.005351987
ENSMUSG00000118052	Gm50432	0.054483349	4.80E-25
ENSMUSG00000024695	Zfp91	0.054235785	0.048165505
ENSMUSG00000024597	Slc12a2	0.054038875	4.55E-13
ENSMUSG00000002546	Golga2	0.05403483	0.000257149
ENSMUSG00000030603	Psmc4	0.053983884	0.000225596
ENSMUSG00000030869	Ndufab1	0.053901252	4.47E-09
ENSMUSG00000027363	Usp8	0.053806625	0.032834201
ENSMUSG00000075700	Selenot	0.053639316	0.000509792
ENSMUSG00000050199	Lgr4	0.0535494	2.95E-21
ENSMUSG00000021629	Slc30a5	0.053490392	7.92E-07
ENSMUSG00000032589	Bsn	0.053428765	6.60E-05
ENSMUSG00000020076	Ddx50	0.053113607	0.002355971
ENSMUSG00000025428	Atp5a1	0.053079169	1.80E-33
ENSMUSG00000031422	Morf4l2	0.052882693	8.27E-08
ENSMUSG00000005566	Trim28	0.052795236	0.000807109
ENSMUSG00000032330	Cox7a2	0.052787608	5.98E-11
ENSMUSG00000044018	Mrpl50	0.052224158	0.005297974
ENSMUSG00000026961	Lrrc26	0.052130084	0.000127622
ENSMUSG00000042041	2010003K11	0.052125502	1.04E-16
ENSMUSG00000042111	Ccdc115	0.052011564	0.008130464
ENSMUSG00000066415	Msl2	0.051779463	0.032969589
ENSMUSG00000024949	Sf1	0.051744163	0.001119242
ENSMUSG00000029068	Ccnl2	0.051425003	1.09E-05
ENSMUSG00000017670	Elmo2	0.050887567	0.036531955
ENSMUSG00000056268	Dennd1b	0.05062087	0.004474675
ENSMUSG00000039883	Lrrc17	0.050059683	0.026272758

ENSMUSG00000005881	Ergic3	-0.015302408	1.96E-05
ENSMUSG00000022664	Slc35a5	-0.015292597	0.020832341
ENSMUSG00000040139	9430038I01F	-0.015097419	4.16E-13
ENSMUSG00000021149	Gtpbp4	-0.015016408	0.011885359
ENSMUSG00000057375	Yipf1	-0.014456313	0.002666373
ENSMUSG00000033732	Sf3b3	-0.014211724	0.023833086
ENSMUSG00000068823	Csde1	-0.014184833	6.54E-05
ENSMUSG00000041765	Ubac2	-0.013997171	0.049040342
ENSMUSG00000039347	Atp6v0e2	-0.013839943	2.00E-07
ENSMUSG00000066900	Suds3	-0.013768055	2.94E-06
ENSMUSG00000039988	Ankrd13c	-0.01362406	0.01149584
ENSMUSG00000044221	Grsf1	-0.013622632	0.041878331
ENSMUSG00000026111	Unc50	-0.013620376	0.016400404
ENSMUSG00000058446	Znrf2	-0.013310559	3.10E-06
ENSMUSG00000023277	Twf2	-0.013059294	0.002820919
ENSMUSG00000024927	Rela	-0.012965487	0.00771235
ENSMUSG00000057933	Gsta2	-0.012554536	3.55E-39
ENSMUSG00000032193	Ldlr	-0.012156737	1.02E-12
ENSMUSG00000003037	Rab8a	-0.0115101	3.60E-12
ENSMUSG00000018559	Ctdnep1	-0.011272617	0.000385292
ENSMUSG00000027088	Phospho2	-0.011008855	6.76E-06
ENSMUSG00000020733	Nherf1	-0.010945803	2.62E-07
ENSMUSG00000038534	Osbpl7	-0.010242719	1.57E-10
ENSMUSG00000025757	Hspa4l	-0.010223413	0.004132785
ENSMUSG00000036390	Gadd45a	-0.010163628	5.86E-06
ENSMUSG00000042043	Tbca	-0.009808652	1.34E-07
ENSMUSG00000037262	Kin	-0.009349024	0.047903534
ENSMUSG00000004394	Tmed4	-0.009348416	6.19E-18
ENSMUSG00000029190	D5Ertd579e	-0.008980665	9.19E-13
ENSMUSG00000021033	Gstz1	-0.008491242	2.52E-09
ENSMUSG00000033499	Larp4b	-0.008322165	0.005845741
ENSMUSG00000020422	Tns3	-0.008180544	3.58E-08
ENSMUSG00000043733	Ptpn11	-0.007849113	0.027753045
ENSMUSG00000026321	Tnfrsf11a	-0.007838939	9.01E-11
ENSMUSG00000020027	Socs2	-0.007772416	1.42E-05
ENSMUSG00000028691	Prdx1	-0.007688374	0.006194536
ENSMUSG00000035258	Abi3bp	-0.007464769	1.42E-12
ENSMUSG00000032350	Gclc	-0.006786543	9.32E-15
ENSMUSG00000000168	Dlat	-0.006761378	6.09E-19
ENSMUSG00000020706	Ftsj3	-0.006702804	0.0024207
ENSMUSG00000056383	AI987944	-0.006623825	0.000882417
ENSMUSG00000005469	Prkaca	-0.006357145	0.000734215
ENSMUSG00000018765	Fxr2	-0.00626172	0.019993235
ENSMUSG00000078945	Naip2	-0.006256183	0.005131288
ENSMUSG00000024935	Slc1a1	-0.006021082	5.96E-40
ENSMUSG00000000827	Tpd52l2	-0.005832872	0.003759036
ENSMUSG00000055850	Rnf181	-0.005784547	0.025456237
ENSMUSG00000021279	Cdc42bpb	-0.005745017	1.76E-05
ENSMUSG00000020592	Sdc1	-0.005539661	4.25E-15
ENSMUSG00000015337	Endog	-0.005487315	6.86E-08
ENSMUSG00000028032	Papss1	-0.005376835	2.18E-26
ENSMUSG00000027184	Caprin1	-0.005050738	0.042573484
ENSMUSG00000056608	Chd9	-0.004773064	0.013314373
ENSMUSG00000035674	Ndufa3	-0.004695317	6.19E-12
ENSMUSG00000026289	Atg16l1	-0.004616845	0.001333094
ENSMUSG00000028426	Rad23b	-0.004222551	6.97E-05
ENSMUSG00000027274	Mkks	-0.004030755	9.17E-05
ENSMUSG00000037033	Clca3b	-0.003871788	3.66E-60
ENSMUSG00000039205	Ciz1	-0.003871536	0.008543078
ENSMUSG00000055435	Maf	-0.003857637	1.27E-12
ENSMUSG00000048701	Ccdc6	-0.003645275	0.000270554
ENSMUSG00000003752	Itpkc	-0.00347896	5.54E-05
ENSMUSG00000024867	Pip5k1b	-0.003440467	0.000248211
ENSMUSG00000029528	Pxn	-0.002488109	0.006328797
ENSMUSG00000032803	Cdv3	-0.002436561	1.50E-10
ENSMUSG00000079507	H2-Q1	-0.002395383	0.000937765
ENSMUSG00000026753	Ppp6c	-0.002009203	0.036167935
ENSMUSG00000036392	Med8	-0.001908908	0.007058908
ENSMUSG00000039159	Ube2h	-0.001769451	3.59E-05
ENSMUSG00000024732	Ccdc86	-0.001232288	0.010158039
ENSMUSG00000003062	Stard3nl	-0.000387591	0.017189204

ENSMUSG00000001786	Fbxo7	0.061275584	0.027264864
ENSMUSG00000032348	Gsta4	0.060691223	1.11E-28
ENSMUSG00000023341	Mx2	0.060596996	0.002020061
ENSMUSG00000017307	Acot8	0.060397793	0.001388687
ENSMUSG00000021000	Mia2	0.060296501	7.50E-18
ENSMUSG00000024955	Esrra	0.060195918	5.68E-07
ENSMUSG00000030894	Tpp1	0.059959422	0.000651315
ENSMUSG00000046417	Lrrc75a	0.059902065	0.004147725
ENSMUSG00000031545	Gpat4	0.059371206	0.000181809
ENSMUSG00000026303	Mlph	0.058918178	1.53E-09
ENSMUSG00000030539	Sema4b	0.058874053	1.86E-06
ENSMUSG00000039364	Sectm1b	0.058862173	7.16E-06
ENSMUSG00000061559	Skic8	0.058693221	0.001862014
ENSMUSG00000030536	Iqgap1	0.058595158	0.025806961
ENSMUSG00000028990	Lzic	0.05848002	2.00E-10
ENSMUSG00000021650	Ptcd2	0.058313451	0.045106406
ENSMUSG00000025337	Sbds	0.058296575	1.84E-22
ENSMUSG00000027599	Armc1	0.058243251	0.012869605
ENSMUSG00000038668	Lpar1	0.058160434	9.83E-09
ENSMUSG00000042506	Usp22	0.057788157	0.000193735
ENSMUSG00000041757	Plekha6	0.057663314	2.96E-13
ENSMUSG00000038365	Fbxo25	0.057135183	0.004424072
ENSMUSG00000028008	Asic5	0.057049142	0.009523928
ENSMUSG00000038335	Tsr1	0.056956105	3.62E-05
ENSMUSG00000022765	Snap29	0.056708309	0.002476358
ENSMUSG00000007827	Ankrd26	0.056686233	0.009790995
ENSMUSG00000026275	Ppp1r7	0.056605724	0.000626073
ENSMUSG00000027230	Creb3l1	0.056590273	2.04E-16
ENSMUSG00000026667	Uhmk1	0.05599132	5.81E-05
ENSMUSG00000032226	Gcnt3	0.055672859	8.39E-13
ENSMUSG00000063952	Brpf3	0.055495134	0.000170658
ENSMUSG00000029735	Tpk1	0.055458783	0.001234225
ENSMUSG00000005397	Nid1	0.055356649	0.014772576
ENSMUSG00000066324	Bpnt2	0.055185991	1.41E-17
ENSMUSG00000070697	Utp3	0.055026863	0.003453114
ENSMUSG00000004040	Stat3	0.054919935	0.000148257
ENSMUSG00000019278	Dpep1	0.054622493	1.01E-17
ENSMUSG00000060703	Cd302	0.054438502	1.20E-14
ENSMUSG00000059811	Atl2	0.054429232	1.51E-13
ENSMUSG00000032185	Carm1	0.054181916	0.007445477
ENSMUSG00000048534	Jaml	0.054172155	3.63E-12
ENSMUSG00000036959	Bcorl1	0.054012989	0.000579926
ENSMUSG00000041870	Ankrd13a	0.053998646	1.73E-11
ENSMUSG00000019505	Ubb	0.053975135	1.55E-05
ENSMUSG00000017774	Myo1c	0.053671415	0.000278975
ENSMUSG00000036214	Polr1has	0.053558562	9.27E-07
ENSMUSG00000031556	Tm2d2	0.053552246	0.004759227
ENSMUSG00000022437	Samm50	0.053487117	0.008000815
ENSMUSG00000022827	Rabl3	0.053383393	0.014528976
ENSMUSG00000021807	Rtraf	0.053210222	0.016632808
ENSMUSG00000057554	Lgals8	0.053162725	1.80E-07
ENSMUSG00000007670	Khsrp	0.053060223	4.83E-08
ENSMUSG00000026922	Agpat2	0.052826305	7.57E-05
ENSMUSG00000042487	Leo1	0.052325718	0.00686984
ENSMUSG00000024508	Gab2	0.052082305	1.28E-05
ENSMUSG00000001739	Cldn15	0.052044049	9.23E-06
ENSMUSG00000032788	Eif3c	0.051770976	3.21E-15
ENSMUSG00000030200	Bcl2l14	0.051712806	1.36E-19
ENSMUSG00000024942	Capn1	0.051579795	0.025146021
ENSMUSG00000069520	Tmem19	0.051431926	0.000242749
ENSMUSG00000027646	Src	0.051360447	0.001157494
ENSMUSG00000044768	Macir	0.051192904	0.00460451
ENSMUSG00000025467	Prap1	0.051158559	5.66E-85
ENSMUSG00000043705	Capn13	0.050995411	6.02E-08
ENSMUSG00000113198	Gm6988	0.050891698	0.007850221
ENSMUSG00000018931	Natd1	0.050782767	0.00751999
ENSMUSG00000020923	Ubtf	0.050720308	0.001666561
ENSMUSG00000018398	Septin8	0.050677566	3.24E-16
ENSMUSG00000036402	Gng12	0.050485163	0.010434852
ENSMUSG00000025135	Anapc11	0.05044586	0.003168019
ENSMUSG00000041837	Pdcd7	0.050112237	0.043831375
ENSMUSG00000053819	Camk2d	0.05011167	2.97E-15
ENSMUSG00000042599	Kdm7a	0.049598642	0.000185654
ENSMUSG00000020483	Dynll2	0.049580072	0.02430588
ENSMUSG00000052748	Swt1	0.049361458	2.78E-05
ENSMUSG00000036112	Metap2	0.049280375	4.56E-07
ENSMUSG00000039318	Rab3gap2	0.049255719	4.33E-10
ENSMUSG00000110926	Gm5917	0.049252065	0.038452778
ENSMUSG00000002250	Ppard	0.04905551	0.001191729
ENSMUSG00000026553	Copa	0.049054408	0.019989731
ENSMUSG00000033581	Igf2bp2	0.048880245	2.07E-07
ENSMUSG00000024914	Drap1	0.048870539	0.024215576
ENSMUSG00000024875	Yif1a	0.048618479	1.33E-15
ENSMUSG00000041747	Utp15	0.048311529	0.000180467
ENSMUSG00000054302	Eapp	0.048035547	0.003857249
ENSMUSG00000041605	Inava	0.04801177	7.16E-20
ENSMUSG00000107283	Mpv17	0.047717166	0.000238775
ENSMUSG00000006342	Susd2	0.047466959	0.000299811
ENSMUSG00000022090	Pdlim2	0.047438796	0.022628139
ENSMUSG00000038002	Cramp1	0.047432713	2.22E-05
ENSMUSG00000027667	Zfp639	0.047405285	0.018241225
ENSMUSG00000030613	Ccdc90b	0.047218438	1.27E-10
ENSMUSG00000001270	Ckb	0.047153707	2.14E-131
ENSMUSG00000007836	Hnrnpa0	0.047147856	4.47E-16
ENSMUSG00000046840	Hnf4aos	0.046578392	0.043365485
ENSMUSG00000020521	Rnft1	0.046163468	0.032828358
ENSMUSG00000000743	Chmp1a	0.04614049	0.007806352
ENSMUSG00000039062	Anpep	0.046081742	1.18E-07
ENSMUSG00000090019	Gimap1	0.045791236	0.019765081
ENSMUSG00000037851	Iars	0.045535105	0.021047305
ENSMUSG00000069045	Ddx3y	0.04545094	1.16E-06
ENSMUSG00000053470	Kdm3a	0.045304889	0.00288652
ENSMUSG00000061947	Serpina10	0.045289523	2.84E-34
ENSMUSG00000033434	Gtpbp6	0.044996536	0.037050981
ENSMUSG00000035840	Lysmd3	0.044856204	0.000639615
ENSMUSG00000028410	Dnaja1	0.044565565	0.007233847
ENSMUSG00000035239	Neu3	0.044462023	1.18E-22
ENSMUSG00000029535	Triap1	0.044283588	0.004892759
ENSMUSG00000028649	Macf1	0.043771066	0.008083509
ENSMUSG00000033022	Cdo1	0.04331613	1.70E-05
ENSMUSG00000040268	Plekha1	0.04313224	0.021350326
ENSMUSG00000028836	Slc30a2	0.042664523	2.64E-09
ENSMUSG00000091537	Tma7	0.042398	1.83E-12
ENSMUSG00000027282	Mtch2	0.042337405	1.69E-07
ENSMUSG00000008859	Rala	0.042079055	0.000202213
ENSMUSG00000000184	Ccnd2	0.041762973	1.51E-15
ENSMUSG00000040414	Slc25a28	0.041754988	0.008520681
ENSMUSG00000024780	Cdc37l1	0.041579994	9.15E-06
ENSMUSG00000039756	Dnttip2	0.041504465	0.019190536
ENSMUSG00000034101	Ctnnd1	0.041451436	1.59E-10
ENSMUSG00000100801	Gm15459	0.040602952	0.034609858
ENSMUSG00000046836	Brox	0.040574858	0.001925028

ENSMUSG00000004460	Dnajb11	-0.011125164	0.017673494
ENSMUSG00000027901	Dennd2d	-0.011115235	8.45E-13
ENSMUSG00000028426	Rad23b	-0.011081526	6.97E-05
ENSMUSG00000033295	Ptprf	-0.0109927	2.49E-06
ENSMUSG00000027282	Mtch2	-0.01063593	1.69E-07
ENSMUSG00000021266	Wars1	-0.010512471	1.24E-08
ENSMUSG00000027287	Snap23	-0.010068356	0.002896972
ENSMUSG00000032123	Dpagt1	-0.010029306	1.08E-07
ENSMUSG00000050721	Plekho2	-0.009875071	0.00839658
ENSMUSG00000006498	Ptbp1	-0.009803552	4.17E-05
ENSMUSG00000045482	Trrap	-0.009793579	0.014730004
ENSMUSG00000024294	Mib1	-0.009607486	0.008567265
ENSMUSG00000038965	Ube2l3	-0.009347206	0.035699997
ENSMUSG00000004054	Map3k11	-0.00921218	0.036177317
ENSMUSG00000021040	Slirp	-0.008989748	0.002407021
ENSMUSG00000024228	Nudt12	-0.008699725	6.88E-05
ENSMUSG00000001105	Ift20	-0.008519842	1.71E-10
ENSMUSG00000017376	Nlk	-0.008350983	0.024814147
ENSMUSG00000031835	Mbtps1	-0.008236108	0.012964896
ENSMUSG00000005237	Dnah2	-0.007401111	2.61E-38
ENSMUSG00000073616	Cops9	-0.007310783	2.75E-08
ENSMUSG00000021028	Mbip	-0.007228187	0.01152163
ENSMUSG00000042599	Kdm7a	-0.007129088	0.000185654
ENSMUSG00000045160	Bola3	-0.007050236	4.18E-28
ENSMUSG00000027288	Zfp106	-0.006875586	0.000146124
ENSMUSG00000025903	Lypla1	-0.006853389	4.31E-07
ENSMUSG00000007880	Arid1a	-0.006847762	2.85E-09
ENSMUSG00000041697	Atg12	-0.00679161	5.59E-06
ENSMUSG00000032459	Mrps22	-0.006486254	0.005762682
ENSMUSG00000022634	Yaf2	-0.005762199	0.008655707
ENSMUSG00000021676	Iqgap2	-0.005681515	1.08E-07
ENSMUSG00000039774	Galnt12	-0.005471851	2.21E-18
ENSMUSG00000073676	Hspe1	-0.005284115	0.001333094
ENSMUSG00000026820	Ptges2	-0.005273151	6.11E-13
ENSMUSG00000022390	Zc3h7b	-0.005224759	0.00683327
ENSMUSG00000052707	Tnrc6a	-0.004996479	0.00394793
ENSMUSG00000027642	Rpn2	-0.00476142	2.62E-07
ENSMUSG00000020415	Pttg1	-0.004618275	2.13E-10
ENSMUSG00000051339	2900026A02	-0.004242336	1.75E-12
ENSMUSG00000054733	Msra	-0.003960804	5.27E-18
ENSMUSG00000021917	Spcs1	-0.003910519	0.023607887
ENSMUSG00000000159	Igsf5	-0.003713302	1.49E-05
ENSMUSG00000040725	Hnrnpul1	-0.003335802	0.000296927
ENSMUSG00000022241	Tars	-0.003058371	0.025814688
ENSMUSG00000021076	Actr10	-0.003054867	0.043327932
ENSMUSG00000024847	Aip	-0.002973657	2.12E-08
ENSMUSG00000029082	Bst1	-0.002891399	5.52E-20
ENSMUSG00000021900	Btd	-0.002716935	0.026185226
ENSMUSG00000054302	Eapp	-0.002673266	0.003857249
ENSMUSG00000052738	Suclg1	-0.002652753	5.48E-12
ENSMUSG00000061143	Maml3	-0.002371644	0.00059653
ENSMUSG00000006782	Cnp	-0.001988999	0.023438406
ENSMUSG00000027469	Tpx2	-0.001949953	2.29E-07
ENSMUSG00000031834	Pik3r2	-0.001760259	0.000783947
ENSMUSG00000028729	Ebna1bp2	-0.001484949	0.003057355
ENSMUSG00000030104	Edem1	-0.001136995	0.000336271
ENSMUSG00000039428	Tmem135	-0.000561709	5.69E-10
ENSMUSG00000041859	Mcm3	-0.000460368	0.000144759
ENSMUSG00000052056	Zfp217	-0.000366787	0.017524734
ENSMUSG00000040771	Oard1	-0.000359323	0.029872325
ENSMUSG00000033200	Tpsg1	-0.00026796	0.012744647
ENSMUSG00000022452	Smdt1	-0.000159895	3.42E-10

ENSMUSG00000021944	Gata4	0.050005967	2.33E-08
ENSMUSG00000014426	Map3k4	0.049959645	0.026725332
ENSMUSG00000036112	Metap2	0.049749536	4.56E-07
ENSMUSG00000025609	Mkln1	0.049715501	4.78E-06
ENSMUSG00000048277	Syngr2	0.049673144	1.24E-07
ENSMUSG00000034940	Synrg	0.049572686	3.69E-05
ENSMUSG00000017774	Myo1c	0.04956033	0.000278975
ENSMUSG00000043987	Cep164	0.049412404	0.00014853
ENSMUSG00000059208	Hnrnpm	0.049345378	2.77E-07
ENSMUSG00000074440	Defa3	0.049245926	0.04521167
ENSMUSG00000018770	Atp5g3	0.049242673	2.10E-18
ENSMUSG00000046814	Gchfr	0.049240899	4.89E-13
ENSMUSG00000066043	Phactr4	0.048597614	0.023651903
ENSMUSG00000033499	Larp4b	0.048534911	0.005845741
ENSMUSG00000044496	2510039O18	0.048435521	9.53E-05
ENSMUSG00000039456	Morc3	0.048275776	0.005520237
ENSMUSG00000024743	Syt7	0.048270889	1.93E-14
ENSMUSG00000028452	Vcp	0.04811038	5.32E-05
ENSMUSG00000019991	Tmpo	0.048098492	0.049233269
ENSMUSG00000047126	Cltc	0.047727167	0.008226494
ENSMUSG00000054169	Ceacam10	0.047701083	1.05E-13
ENSMUSG00000018822	Sfrp5	0.047549687	1.17E-11
ENSMUSG00000033792	Atp7a	0.047487916	0.000127399
ENSMUSG00000105879	Gm6204	0.047485757	0.000809269
ENSMUSG00000027030	Stk39	0.04738121	1.21E-11
ENSMUSG00000037408	Cnnm4	0.047270285	9.76E-12
ENSMUSG00000022339	Ebag9	0.047026686	0.040771107
ENSMUSG00000041697	Cox6a1	0.046767747	2.48E-37
ENSMUSG00000025393	Atp5b	0.046601591	4.09E-18
ENSMUSG00000068036	Afdn	0.046598989	2.43E-11
ENSMUSG00000071652	Ints5	0.046526121	0.002222725
ENSMUSG00000030342	Cd9	0.04641274	3.87E-09
ENSMUSG00000020048	Hsp90b1	0.046163464	1.79E-09
ENSMUSG00000014313	Cox6c	0.046113499	2.39E-10
ENSMUSG00000007392	Thoc2l	0.046001993	5.76E-07
ENSMUSG00000036832	Lpar3	0.045853179	0.034275011
ENSMUSG00000025314	Ptprj	0.04573428	1.70E-05
ENSMUSG00000020581	Agr2	0.045648431	8.43E-08
ENSMUSG00000027854	Sike1	0.045531461	0.00040374
ENSMUSG00000111966	Gm5781	0.045315565	6.94E-05
ENSMUSG00000049658	Bdp1	0.045231648	1.47E-06
ENSMUSG00000003161	Sri	0.045182463	1.58E-09
ENSMUSG00000062382	Ftl1-ps2	0.045031322	0.000348845
ENSMUSG00000021774	Ube2e1	0.044544428	0.000327924
ENSMUSG00000024960	Plcb3	0.04434382	0.002032081
ENSMUSG00000039013	Siglecf	0.044303428	9.07E-06
ENSMUSG00000062867	Impdh2	0.044083755	4.83E-08
ENSMUSG00000025494	Sigirr	0.043858171	0.022849969
ENSMUSG00000032175	Tyk2	0.043778111	0.01040779
ENSMUSG00000005893	Nr2c2	0.043679573	0.000260491
ENSMUSG00000021775	Nr1d2	0.043668368	0.000743791
ENSMUSG00000031278	Acsl4	0.043523463	0.042971842
ENSMUSG00000056973	Ces1d	0.043220204	2.19E-36
ENSMUSG00000023079	Gtf2ird1	0.04294262	0.022794622
ENSMUSG00000024782	Ak3	0.042933546	1.57E-06
ENSMUSG00000026395	Ptprc	0.042825671	1.06E-06
ENSMUSG00000042198	Chchd7	0.042460613	0.000274522
ENSMUSG00000029634	Rnf6	0.042424048	0.009088058
ENSMUSG00000023939	Mrpl14	0.041748803	0.000141232
ENSMUSG00000031796	Cfap20	0.041679399	0.00027929
ENSMUSG00000062070	Pgk1	0.041435593	4.77E-05
ENSMUSG00000070493	Chchd2	0.04141485	3.59E-09
ENSMUSG00000031891	Hsd11b2	0.041355167	1.85E-05
ENSMUSG00000003033	Ap1m1	0.041284287	0.049255473
ENSMUSG00000028940	Hes2	0.041248856	0.016462958
ENSMUSG00000039942	Ptger4	0.041156344	2.92E-10
ENSMUSG00000036402	Gng12	0.041088727	0.010434852
ENSMUSG00000040891	Foxa3	0.041058814	4.24E-09
ENSMUSG00000053510	Nrd1	0.041008841	0.001972629
ENSMUSG00000032899	Styk1	0.040885326	0.001398362
ENSMUSG00000032253	Phip	0.040639737	1.07E-05
ENSMUSG00000015533	Itga2	0.040566082	2.91E-10
ENSMUSG00000001228	Uhrf1	0.040503638	0.000373215
ENSMUSG00000031605	Klhl2	0.040466078	0.008322076
ENSMUSG00000026615	Eprs	0.040450511	2.06E-06
ENSMUSG00000022983	Scaf4	0.040343702	3.04E-07
ENSMUSG00000006057	Atp5g1	0.04012277	0.001452938
ENSMUSG00000009549	Srp14	0.039886001	0.00396542
ENSMUSG00000028480	Glipr2	0.038982716	5.72E-21
ENSMUSG00000059555	Tor4a	0.038940512	0.036167935
ENSMUSG00000054693	Adam10	0.038150787	4.66E-08
ENSMUSG00000036751	Cox6b1	0.038060051	7.68E-14
ENSMUSG00000052428	Tmco1	0.038046142	0.00935203
ENSMUSG00000050982	Apol10a	0.038036988	0.001991487
ENSMUSG00000026179	Pnkd	0.037646365	2.03E-08
ENSMUSG00000033751	Gadd45gip1	0.037626977	0.013630866
ENSMUSG00000100153	Ppp1ccb	0.037140693	0.003682118
ENSMUSG00000054874	Pcnx3	0.037116999	0.00030282
ENSMUSG00000035048	Anapc13	0.037007721	0.001240041
ENSMUSG00000041685	Fcho2	0.036690823	0.013594725
ENSMUSG00000087590	Epb41l4aos	0.03664094	0.031263462
ENSMUSG00000039952	Dag1	0.036495234	2.82E-05
ENSMUSG00000022285	Ywhaz	0.036349962	3.50E-11
ENSMUSG00000013701	Timm23	0.03622034	0.003236373
ENSMUSG00000028292	Rars2	0.036178339	0.000598243
ENSMUSG00000031781	Ciapin1	0.036172141	5.09E-05
ENSMUSG00000019853	Hebp2	0.036102957	0.00160016
ENSMUSG00000031505	Naxd	0.035899866	0.009364853
ENSMUSG00000010608	Rbm25	0.035880779	2.88E-13
ENSMUSG00000022635	Zcrb1	0.035793539	0.002005285
ENSMUSG00000020544	Cox11	0.035746	1.49E-09
ENSMUSG00000025091	Pnliprp2	0.035650958	6.98E-15
ENSMUSG00000026970	Rbms1	0.03556528	0.011538857
ENSMUSG00000031257	Nox1	0.035547288	0.00318856
ENSMUSG00000040782	Cop1	0.035539303	0.007216364
ENSMUSG00000000567	Sox9	0.035498112	1.26E-12
ENSMUSG00000025504	Eps8l2	0.035431206	0.014917691
ENSMUSG00000019927	Ube2d1	0.035351737	0.001768159
ENSMUSG00000042628	Zfyve1	0.035167196	0.01962715
ENSMUSG00000036231	Agr3	0.035145566	0.004943332
ENSMUSG00000022781	Pak2	0.034993068	0.000226371
ENSMUSG00000035413	Tmem98	0.03433583	0.000148222
ENSMUSG00000037089	Slc35b2	0.034187361	6.81E-07
ENSMUSG00000055629	B4galnt4	0.034130218	0.001823988
ENSMUSG00000001313	Rnd2	0.03399145	2.40E-12
ENSMUSG00000025006	Sorbs1	0.033898221	0.020694027
ENSMUSG00000068823	Csde1	0.033741264	6.54E-05
ENSMUSG00000028412	Slc44a1	0.033558952	9.08E-24
ENSMUSG00000038618	Rassf7	0.033343945	0.000151277
ENSMUSG00000020464	Pnpt1	0.033054398	0.002881478
ENSMUSG00000022663	Atg3	0.032940182	0.046384458
ENSMUSG00000069516	Lyz2	0.032795662	0.015095697

ENSMUSG00000031103	Elf4	0.040525784	2.88E-06
ENSMUSG00000020841	Cpd	0.040307415	3.20E-12
ENSMUSG00000041681	Iapp	0.040259269	0.000111281
ENSMUSG00000016409	Nkap	0.039677802	0.048388236
ENSMUSG00000032112	Trappc4	0.039446251	7.91E-09
ENSMUSG00000017386	Traf4	0.038943997	0.004569192
ENSMUSG00000024790	Sac3d1	0.038545226	4.27E-07
ENSMUSG00000001323	Srr	0.03841591	0.002938991
ENSMUSG00000038412	Higd1a	0.037910425	1.83E-20
ENSMUSG00000055917	Zfp277	0.03776413	0.000594613
ENSMUSG00000038400	Pmepa1	0.037471018	0.001759583
ENSMUSG00000011114	Tbrg1	0.03710941	1.79E-10
ENSMUSG00000020075	Ddx21	0.036949696	1.54E-06
ENSMUSG00000076609	Igkc	0.036670603	1.95E-09
ENSMUSG00000029578	Wipi2	0.036539356	0.001377849
ENSMUSG00000031431	Tsc22d3	0.036449955	1.03E-07
ENSMUSG00000068036	Afdn	0.036399631	2.43E-11
ENSMUSG00000070002	Ell	0.036131916	2.63E-05
ENSMUSG00000057230	Aak1	0.036046695	0.045652398
ENSMUSG00000030591	Psmd8	0.03586515	0.014788109
ENSMUSG00000056851	Pcbp2	0.035837477	0.005793103
ENSMUSG00000037007	Zfp113	0.035737525	0.04575066
ENSMUSG00000020149	Rab1a	0.03564154	2.36E-06
ENSMUSG00000022350	Washc5	0.035523377	8.23E-05
ENSMUSG00000024908	Ppp6r3	0.03550247	0.042758846
ENSMUSG00000037408	Cnnm4	0.035414499	9.76E-12
ENSMUSG00000061411	Nol4l	0.035375241	0.000434132
ENSMUSG00000033845	Mrpl15	0.035040836	0.000550197
ENSMUSG00000028463	Car9	0.034873121	0.005196348
ENSMUSG00000078622	Ccdc47	0.034869093	0.007901452
ENSMUSG00000026260	Ndufa10	0.034559884	5.01E-16
ENSMUSG00000052310	Slc39a1	0.034437246	0.014625219
ENSMUSG00000061950	Ppp4r1	0.034063687	0.001581674
ENSMUSG00000000142	Axin2	0.03392597	4.37E-25
ENSMUSG00000028273	Pdlim5	0.033605343	0.006962127
ENSMUSG00000027490	E2f1	0.033541496	0.045454589
ENSMUSG00000022175	Lrp10	0.033255776	5.02E-05
ENSMUSG00000036109	Mbnl3	0.032725714	0.021126102
ENSMUSG00000044030	Irf2bp1	0.032591377	8.83E-05
ENSMUSG00000002396	Ocel1	0.032563835	0.001401274
ENSMUSG00000049295	Zfp219	0.032475405	0.00120847
ENSMUSG00000029405	G3bp2	0.031757393	0.00085329
ENSMUSG00000025557	Slc15a1	0.031595513	0.000789598
ENSMUSG00000036026	Tmem63b	0.03144866	1.03E-05
ENSMUSG00000052135	Foxo6	0.031209071	3.25E-13
ENSMUSG00000002983	Relb	0.030652784	5.64E-22
ENSMUSG00000020231	Dip2a	0.030626502	0.002463622
ENSMUSG00000019809	Pex3	0.030307395	1.22E-10
ENSMUSG00000026687	Aldh9a1	0.030304837	2.48E-06
ENSMUSG00000079659	Tmem243	0.030304109	0.000344906
ENSMUSG00000024130	Abca3	0.030013986	2.72E-06
ENSMUSG00000032458	Copb2	0.029723293	0.001129268
ENSMUSG00000042428	Mgat3	0.029613235	0.000410973
ENSMUSG00000059263	Usp47	0.029557603	0.002223072
ENSMUSG00000060992	Copz1	0.029489064	0.002269788
ENSMUSG00000096210	H1f0	0.029333779	0.000101065
ENSMUSG00000038780	Smurf1	0.028819814	0.022628139
ENSMUSG00000071711	Mpst	0.028751731	3.75E-14
ENSMUSG00000049553	Polr1a	0.028450473	1.67E-05
ENSMUSG00000092274	Neat1	0.028301375	0.000681713
ENSMUSG00000033068	Entpd6	0.027877091	0.009325215
ENSMUSG00000030447	Cyfip1	0.02784952	0.019104762
ENSMUSG00000014074	Rnf168	0.027800534	0.00107887
ENSMUSG00000025246	Tbl1x	0.027532267	5.13E-07
ENSMUSG00000004789	Dlst	0.027151619	5.48E-13
ENSMUSG00000026219	Trip12	0.026962923	0.004081927
ENSMUSG00000006345	Ggt1	0.02629631	3.31E-10
ENSMUSG00000053113	Socs3	0.026281942	0.000194357
ENSMUSG00000024998	Plce1	0.026236012	9.87E-14
ENSMUSG00000001630	Stk38l	0.026094121	0.000874659
ENSMUSG00000055629	B4galnt4	0.025861461	0.001823988
ENSMUSG00000026670	Uap1	0.02541971	2.54E-07
ENSMUSG00000003528	Slc25a1	0.025394537	1.43E-05
ENSMUSG00000078202	Nrarp	0.025072394	3.97E-06
ENSMUSG00000022052	Ppp2r2a	0.025029625	2.31E-05
ENSMUSG00000026576	Atp1b1	0.025024559	2.50E-07
ENSMUSG00000026519	Tmem63a	0.024952489	3.38E-05
ENSMUSG00000052406	Rexo4	0.024931912	0.037490416
ENSMUSG00000045594	Glb1	0.024853035	4.87E-08
ENSMUSG00000028672	Hmgcl	0.024623654	5.38E-09
ENSMUSG00000029106	Add1	0.02443176	2.03E-09
ENSMUSG00000031482	Slc25a15	0.023658268	2.71E-06
ENSMUSG00000062234	Gak	0.023631415	0.000474525
ENSMUSG00000059005	Hnrnpa3	0.023520349	1.36E-05
ENSMUSG00000027720	Il2	0.022910131	1.23E-07
ENSMUSG00000020883	Fbxl20	0.022889086	0.011413888
ENSMUSG00000063172	Ift25	0.022718239	0.04423346
ENSMUSG00000032232	Cgnl1	0.022522127	0.00447197
ENSMUSG00000017802	Retreg3	0.021965462	3.66E-05
ENSMUSG00000031245	Hmgn5	0.021385131	8.17E-10
ENSMUSG00000085427	6430710C18	0.021287232	7.06E-07
ENSMUSG00000028792	Ak2	0.021073543	6.33E-34
ENSMUSG00000024074	Crim1	0.020862242	2.93E-05
ENSMUSG00000049922	Slc35c1	0.020467378	0.000794929
ENSMUSG00000026150	Mff	0.020420595	0.002231949
ENSMUSG00000090000	Ier3ip1	0.020352232	0.000326758
ENSMUSG00000027099	Mtx2	0.020293645	1.46E-08
ENSMUSG00000026279	Thap4	0.020266006	1.71E-12
ENSMUSG00000107176	Gm9794	0.020217055	0.000658285
ENSMUSG00000028249	Sdcbp	0.020151919	0.042130774
ENSMUSG00000032637	Atxn2l	0.020045166	0.017576169
ENSMUSG00000055415	Atp10b	0.019992166	0.000117056
ENSMUSG00000051147	Nat2	0.019853887	2.05E-05
ENSMUSG00000008730	Hipk1	0.019777195	1.03E-08
ENSMUSG00000021520	Uqcrb	0.019513753	0.000127633
ENSMUSG00000035441	Myo1d	0.019509807	1.83E-20
ENSMUSG00000003345	Csnk1g2	0.019390735	0.016400404
ENSMUSG00000006678	Pola1	0.019376756	0.005994317
ENSMUSG00000047126	Cltc	0.019258538	0.008226494
ENSMUSG00000029071	Dvl1	0.01836086	0.000510606
ENSMUSG00000028381	Ugcg	0.018328757	0.001765034
ENSMUSG00000060147	Serpinb6a	0.018001895	0.002761641
ENSMUSG00000095325	Zfp870	0.017949411	4.50E-07
ENSMUSG00000026175	Vil1	0.017920381	0.003888009
ENSMUSG00000035762	Tmem161b	0.017879633	4.16E-07
ENSMUSG00000031485	Plpbp	0.01754725	0.048561538
ENSMUSG00000034343	Ube2f	0.01736434	3.24E-06
ENSMUSG00000042041	2010003K11	0.017276112	1.04E-16
ENSMUSG00000053477	Tcf4	0.017110414	0.025518977
ENSMUSG00000042644	Itpr3	0.016756841	0.000111671
ENSMUSG00000032314	Etfa	0.016581832	1.16E-10
ENSMUSG00000020003	Pex7	0.016540839	0.004490301

ENSMUSG00000041439	Mfsd6	0.032727521	3.55E-09
ENSMUSG00000022219	Cideb	0.03269756	0.004938028
ENSMUSG00000066800	Rnasel	0.032685687	4.14E-06
ENSMUSG00000057982	Zfp809	0.032418115	0.039896575
ENSMUSG00000039438	Ttc36	0.032406556	0.006308072
ENSMUSG00000071662	Polr2g	0.032150159	0.000821776
ENSMUSG00000039756	Dnttip2	0.031998587	0.019190536
ENSMUSG00000041762	Gpr155	0.031830302	1.17E-13
ENSMUSG00000015092	Edf1	0.031575319	1.96E-09
ENSMUSG00000027784	Ppm1l	0.031567568	0.031823412
ENSMUSG00000014077	Chp1	0.031564104	0.002308611
ENSMUSG00000070733	Fryl	0.031562078	0.000155063
ENSMUSG00000050390	C77080	0.031529028	7.88E-08
ENSMUSG00000049106	Dcaf5	0.03137989	0.019448091
ENSMUSG00000002345	Borcs8	0.031073591	0.022362873
ENSMUSG00000027589	Pcmtd2	0.031057317	0.000302619
ENSMUSG00000032454	Rbp2	0.031044095	1.49E-78
ENSMUSG00000020190	Mknk2	0.031022621	0.00037279
ENSMUSG00000021392	Nol8	0.031011425	0.044533113
ENSMUSG00000024006	Stk38	0.03087572	8.60E-05
ENSMUSG00000041688	Amot	0.03071643	0.00071503
ENSMUSG00000021731	Mrps30	0.030709933	6.24E-07
ENSMUSG00000070047	Fat1	0.030406806	1.36E-13
ENSMUSG00000030035	Wbp1	0.030300977	0.039111291
ENSMUSG00000066621	Tecpr1	0.030208354	0.000741664
ENSMUSG00000040850	Psme4	0.03020588	0.022279962
ENSMUSG00000027306	Nusap1	0.029969632	6.43E-07
ENSMUSG00000028955	Vamp3	0.029886937	0.000446012
ENSMUSG00000040498	Igsf23	0.029755643	1.20E-11
ENSMUSG00000026500	Cox20	0.029563841	0.035315011
ENSMUSG00000038264	Sema7a	0.029173194	0.004409113
ENSMUSG00000071178	Serpina1b	0.029073972	0.002466377
ENSMUSG00000027304	Rtf1	0.028990776	9.76E-06
ENSMUSG00000030203	Dusp16	0.028901996	0.000159141
ENSMUSG00000028173	Wls	0.028851905	4.18E-05
ENSMUSG00000073725	Lmbrd1	0.028762069	0.016279203
ENSMUSG00000025421	Hdhd2	0.028690729	9.66E-06
ENSMUSG00000048490	Nrip1	0.02848001	0.003274848
ENSMUSG00000031885	Cbfb	0.028326256	0.00038681
ENSMUSG00000048000	Gigyf2	0.02801419	0.00021241
ENSMUSG00000026775	Yme1l1	0.028005881	6.79E-08
ENSMUSG00000050002	Idnk	0.027964373	4.94E-09
ENSMUSG00000032202	Rab27a	0.027908967	0.029014665
ENSMUSG00000000399	Ndufa9	0.027859453	5.75E-06
ENSMUSG00000036632	Alg5	0.027793009	0.001926304
ENSMUSG00000032468	Armc8	0.02703445	0.003552609
ENSMUSG00000056209	Npm3	0.026862606	0.001510097
ENSMUSG00000016520	Lnx2	0.02670811	0.023590132
ENSMUSG00000029465	Arpc3	0.026596086	4.90E-05
ENSMUSG00000061740	Cyp2d22	0.026385083	8.80E-05
ENSMUSG00000067369	Trmt2b	0.026377244	2.23E-08
ENSMUSG00000035437	Rabgap1	0.026237114	0.000412556
ENSMUSG00000025812	Pard3	0.025965555	7.14E-05
ENSMUSG00000074892	B3galt5	0.025693496	0.000151277
ENSMUSG00000001786	Fbxo7	0.025600256	0.027264864
ENSMUSG00000056124	B4galt6	0.025558607	1.51E-10
ENSMUSG00000022890	Atp5j	0.025400386	8.38E-05
ENSMUSG00000009907	Vps4b	0.025390735	0.00241387
ENSMUSG00000017707	Serinc3	0.025340804	9.83E-06
ENSMUSG00000074733	Zfp950	0.024728687	0.018289775
ENSMUSG00000044433	Camsap3	0.024589424	4.08E-11
ENSMUSG00000014856	Tmem208	0.024061372	3.45E-07
ENSMUSG00000003382	Etv3	0.023918403	0.004127172
ENSMUSG00000020390	Ube2b	0.02386678	1.69E-08
ENSMUSG00000034528	Hsd17b13	0.02371851	5.76E-28
ENSMUSG00000025917	Cops5	0.023390358	0.000612087
ENSMUSG00000026049	Tex30	0.023242406	0.029257646
ENSMUSG00000035242	Oaz1	0.023115528	1.98E-11
ENSMUSG00000003534	Ddr1	0.022723465	1.15E-07
ENSMUSG00000053477	Tcf4	0.02267986	0.025518977
ENSMUSG00000015597	Zfp318	0.022651543	0.000110198
ENSMUSG00000106918	Mrpl33	0.022549884	0.007110926
ENSMUSG00000029231	Pdgfra	0.022069426	5.24E-05
ENSMUSG00000020571	Pdia6	0.022022355	9.56E-12
ENSMUSG00000037070	Rbmxl1	0.021805921	0.011726217
ENSMUSG00000101795	Gm5835	0.021736582	3.94E-11
ENSMUSG00000036833	Pnpla7	0.021732103	0.000183961
ENSMUSG00000025289	Prdx4	0.021378062	0.001842335
ENSMUSG00000040471	Ggt6	0.021026345	1.69E-05
ENSMUSG00000035621	Midn	0.02050225	1.06E-11
ENSMUSG00000021610	Clptm1l	0.020472357	0.04962789
ENSMUSG00000026087	Mrpl30	0.020340555	0.005908101
ENSMUSG00000042305	Tmem183a	0.019936214	0.023265812
ENSMUSG00000071076	Jund	0.019935314	4.33E-07
ENSMUSG00000040466	Blvrb	0.019661478	9.45E-06
ENSMUSG00000033735	Spr	0.019561184	1.17E-05
ENSMUSG00000079426	Arpc4	0.019295117	0.000422469
ENSMUSG00000001151	Pcnt	0.019160315	7.28E-10
ENSMUSG00000062169	Cnih4	0.019054632	8.41E-09
ENSMUSG00000022130	Tgds	0.019005305	2.74E-05
ENSMUSG00000063275	Hacd1	0.018915415	0.00040136
ENSMUSG00000003166	Dgcr2	0.018536761	0.00162002
ENSMUSG00000068040	Tm9sf4	0.018473885	0.000601369
ENSMUSG00000120995	Gm51425	0.018376493	0.000103279
ENSMUSG00000024393	Prrc2a	0.018178747	3.79E-05
ENSMUSG00000001289	Pfdn5	0.018051327	0.001457869
ENSMUSG00000081684	Rps2-ps13	0.017740609	0.045962863
ENSMUSG00000012422	Tmem167	0.017325531	1.09E-16
ENSMUSG00000035953	Pip4p1	0.017211534	0.028064487
ENSMUSG00000046840	Hnf4aos	0.017079259	0.043365485
ENSMUSG00000031090	Nadsyn1	0.017022418	9.62E-07
ENSMUSG00000032485	Scap	0.016572656	3.90E-05
ENSMUSG00000030059	Tmf1	0.016517665	0.0474828
ENSMUSG00000031668	Eif2ak3	0.016235474	0.002938088
ENSMUSG00000024795	Kif20b	0.016085143	1.79E-06
ENSMUSG00000001403	Ube2c	0.016061287	8.73E-07
ENSMUSG00000005686	Ampd3	0.015959515	0.000250308
ENSMUSG00000028223	Decr1	0.015924306	7.57E-15
ENSMUSG00000107176	Gm9794	0.015844564	0.000658285
ENSMUSG00000038722	Bud31	0.015503717	6.22E-05
ENSMUSG00000062624	Cyp2c67	0.015460931	0.007954786
ENSMUSG00000024426	Atat1	0.015408786	8.66E-05
ENSMUSG00000078566	Bnip3	0.015098365	0.000462906
ENSMUSG00000024935	Slc1a1	0.014922299	5.96E-40
ENSMUSG00000021607	Mrpl36	0.014842774	5.97E-05
ENSMUSG00000022185	Acin1	0.014770246	2.07E-11
ENSMUSG00000025645	Ccdc51	0.014690293	0.000657803
ENSMUSG00000038822	Hace1	0.014648948	0.022671839
ENSMUSG00000028180	Zranb2	0.014325517	0.001266844
ENSMUSG00000021366	Hivep1	0.014063193	6.04E-05
ENSMUSG00000041733	Coq5	0.013926532	0.007882978
ENSMUSG00000020368	Canx	0.013894086	2.16E-53

ENSMUSG00000000326	Comt	0.016508004	0.013773667
ENSMUSG00000032712	Resf1	0.016405359	8.14E-18
ENSMUSG00000026209	Dnpep	0.016363089	0.001037584
ENSMUSG00000049232	Tigd2	0.016110489	0.037733281
ENSMUSG00000029275	Gfi1	0.015936011	0.031129781
ENSMUSG00000063358	Mapk1	0.015712695	5.15E-06
ENSMUSG00000025950	Idh1	0.015348389	1.14E-27
ENSMUSG00000024812	Tjp2	0.01534198	0.000337374
ENSMUSG00000058240	Cryzl1	0.015233236	3.34E-06
ENSMUSG00000031333	Abcb7	0.015162852	0.001190587
ENSMUSG00000035623	Rsf1	0.014354454	0.0083493
ENSMUSG00000022283	Pabpc1	0.014068693	3.11E-16
ENSMUSG00000023961	Enpp4	0.01357977	0.0456917
ENSMUSG00000028207	Asph	0.013316661	0.000594212
ENSMUSG00000040322	Slc25a24	0.013250399	4.81E-26
ENSMUSG00000002846	Timmdc1	0.013126384	0.013911135
ENSMUSG00000071178	Serpina1b	0.012957721	0.002466377
ENSMUSG00000078572	Ndufaf8	0.012875644	5.71E-06
ENSMUSG00000066150	Slc31a1	0.012867404	4.12E-05
ENSMUSG00000026821	Ralgds	0.012823118	2.14E-06
ENSMUSG00000070733	Fryl	0.01265891	0.000155063
ENSMUSG00000026201	Stk16	0.012602861	0.000649602
ENSMUSG00000028692	Akr1a1	0.012063357	0.000567593
ENSMUSG00000030304	Ergic2	0.011827043	5.31E-08
ENSMUSG00000028521	Slc35d1	0.011660754	0.001158736
ENSMUSG00000008999	Bmp7	0.011623542	2.13E-05
ENSMUSG00000029028	Lrrc47	0.011476797	0.011874495
ENSMUSG00000024487	Yipf5	0.011156044	1.70E-14
ENSMUSG00000034022	Cpsf1	0.010802892	1.10E-05
ENSMUSG00000046761	Fam83h	0.010606012	1.54E-08
ENSMUSG00000061887	Ssbp3	0.01060521	1.23E-10
ENSMUSG00000029618	Ocm	0.010589673	1.48E-68
ENSMUSG00000044224	Dnajc21	0.010467238	0.005817429
ENSMUSG00000049799	Lrrc19	0.010315791	7.24E-08
ENSMUSG00000025162	Csnk1d	0.010007991	8.31E-05
ENSMUSG00000020840	Blmh	0.009953494	1.17E-06
ENSMUSG00000029445	Hpd	0.009586	0.004556115
ENSMUSG00000056537	Rlim	0.009428258	0.01990862
ENSMUSG00000051391	Ywhag	0.009268742	0.014621979
ENSMUSG00000001891	Ugp2	0.009164283	0.001240041
ENSMUSG00000032526	Ss18l2	0.008970852	1.73E-08
ENSMUSG00000078429	Ctdsp2	0.008681272	7.43E-22
ENSMUSG00000045934	Mtmr11	0.008585636	5.34E-11
ENSMUSG00000030327	Necap1	0.008224935	4.90E-06
ENSMUSG00000030604	Zfp626	0.008031124	0.015514179
ENSMUSG00000006651	Aplp1	0.007832415	0.010654996
ENSMUSG00000023175	Bsg	0.007696451	1.98E-08
ENSMUSG00000039286	Fndc3b	0.007579758	0.000805829
ENSMUSG00000013663	Pten	0.007357268	0.03978833
ENSMUSG00000000399	Ndufa9	0.006694838	5.75E-06
ENSMUSG00000050148	Ubqln2	0.006685311	0.015597078
ENSMUSG00000034973	Dop1a	0.006121512	0.011657083
ENSMUSG00000099632	2900093K20	0.006042813	0.034904457
ENSMUSG00000034243	Golgb1	0.006018262	9.99E-13
ENSMUSG00000020282	Rhbdf1	0.00600712	0.00065325
ENSMUSG00000026849	Tor1a	0.005384788	1.09E-11
ENSMUSG00000004530	Coro1c	0.005144555	7.64E-21
ENSMUSG00000041974	Spidr	0.005020692	0.028064487
ENSMUSG00000030059	Tmf1	0.004708625	0.0474828
ENSMUSG00000058715	Fcer1g	0.004178112	0.000194731
ENSMUSG00000031781	Ciapin1	0.004006047	5.09E-05
ENSMUSG00000032757	Bet1	0.003932332	3.57E-14
ENSMUSG00000046949	Nqo2	0.003929464	0.01634875
ENSMUSG00000014606	Slc25a11	0.003825408	8.72E-27
ENSMUSG00000006095	Tbcb	0.003639915	0.040265882
ENSMUSG00000052557	Gan	0.003206216	9.07E-10
ENSMUSG00000047990	C2cd4a	0.002993242	3.46E-06
ENSMUSG00000040048	Ndufb10	0.002807944	7.74E-14
ENSMUSG00000024006	Stk38	0.002777728	8.60E-05
ENSMUSG00000022365	Derl1	0.00261948	2.05E-10
ENSMUSG00000036461	Elf1	0.002572624	0.011223877
ENSMUSG00000038807	Rap1gap2	0.002416474	9.92E-19
ENSMUSG00000022390	Zc3h7b	0.00213772	0.00683327
ENSMUSG00000095567	Noc2l	0.002108249	2.80E-07
ENSMUSG00000032583	Mon1a	0.00173424	0.021467857
ENSMUSG00000019850	Tnfaip3	0.001487851	0.038132687
ENSMUSG00000040640	Erc2	0.001343401	3.13E-11
ENSMUSG00000040423	Rc3h1	0.00132829	0.047125216
ENSMUSG00000024892	Pcx	0.001183199	0.004761597
ENSMUSG00000038206	Fbxo8	0.001177095	0.01149584
ENSMUSG00000025410	Dctn2	0.001088531	0.022569002
ENSMUSG00000074884	Serf2	0.001084348	4.23E-14
ENSMUSG00000039753	Fbxl5	0.001034523	4.14E-08
ENSMUSG00000021286	Zfyve21	0.000937308	0.016307069
ENSMUSG00000101523	Csnk2a1-ps3	0.000867892	0.000485922
ENSMUSG00000061288	Taok3	0.000778315	3.96E-12
ENSMUSG00000052337	Immt	0.000646678	9.78E-25
ENSMUSG00000060803	Gstp1	0.000638233	4.63E-08
ENSMUSG00000081534	Slc48a1	0.000631682	0.001700455
ENSMUSG00000054414	Slc30a7	0.000169346	0.025601017

ENSMUSG00000040345	Arhgap9	0.013801282	0.007556482
ENSMUSG00000025894	Aasdhppt	0.013561142	0.003327901
ENSMUSG00000014905	Dnajb9	0.013557542	7.16E-09
ENSMUSG00000019359	Gdpd2	0.013473846	4.61E-32
ENSMUSG00000040102	Klhl42	0.012905173	0.046022438
ENSMUSG00000058756	Thra	0.012852177	0.025815408
ENSMUSG00000039531	Zup1	0.012591581	0.009380488
ENSMUSG00000042675	Ypel3	0.012499133	0.033612746
ENSMUSG00000093483	AA465934	0.012240996	0.002381984
ENSMUSG00000038495	Otud7b	0.012207341	0.035739845
ENSMUSG00000014606	Slc25a11	0.01211429	8.72E-27
ENSMUSG00000037710	Cisd1	0.012071981	4.48E-13
ENSMUSG00000028165	Cisd2	0.012055012	0.000157942
ENSMUSG00000030538	Cib1	0.011873168	0.026818078
ENSMUSG00000028572	Hook1	0.011836993	0.005157732
ENSMUSG00000042670	Immp1l	0.011828031	0.008919567
ENSMUSG00000049760	Micos13	0.011501508	1.07E-09
ENSMUSG00000023944	Hsp90ab1	0.01131466	0.000277912
ENSMUSG00000028990	Lzic	0.011240112	2.00E-10
ENSMUSG00000036805	Noxa1	0.011182066	4.43E-07
ENSMUSG00000020721	Helz	0.011047208	0.000968606
ENSMUSG00000062075	Lmnb2	0.010710426	0.003677289
ENSMUSG00000025958	Creb1	0.010512904	1.03E-06
ENSMUSG00000002010	Idh3g	0.010457048	4.89E-10
ENSMUSG00000022964	Tmem50b	0.010166998	0.000104392
ENSMUSG00000021242	Npc2	0.010098174	0.022767552
ENSMUSG00000025192	Entpd7	0.009857061	3.76E-06
ENSMUSG00000041084	Ostc	0.009766901	0.000103815
ENSMUSG00000045328	Cenpe	0.009321178	1.04E-10
ENSMUSG00000020530	Ggnbp2	0.009250667	0.020002825
ENSMUSG00000053862	Slc51b	0.009191001	1.15E-12
ENSMUSG00000051978	Erich1	0.009159775	0.012849896
ENSMUSG00000024388	Myo7b	0.00912549	0.006930624
ENSMUSG00000005161	Prdx2	0.009093448	5.37E-07
ENSMUSG00000026193	Fn1	0.008760084	2.57E-05
ENSMUSG00000022507	Hapstr1	0.00863776	0.044348297
ENSMUSG00000028167	Bdh2	0.008573589	3.67E-16
ENSMUSG00000042797	Aqp11	0.008365504	4.27E-15
ENSMUSG00000031782	Coq9	0.008283338	3.31E-11
ENSMUSG00000039983	Ccdc32	0.008239062	0.000867627
ENSMUSG00000025417	Pip4k2c	0.008159537	2.33E-05
ENSMUSG00000024812	Tjp2	0.007992594	0.000337374
ENSMUSG00000033948	Zswim5	0.00770352	0.03047745
ENSMUSG00000028833	Ncdn	0.007612665	0.004002798
ENSMUSG00000022884	Eif4a2	0.007420135	1.79E-08
ENSMUSG00000046804	Phgr1	0.007167662	4.53E-11
ENSMUSG00000029098	Acox3	0.00700648	0.005836197
ENSMUSG00000035885	Cox8a	0.006984737	4.79E-09
ENSMUSG00000053054	Adh6a	0.006786965	4.87E-60
ENSMUSG00000019039	Dalrd3	0.00678629	0.04222603
ENSMUSG00000021427	Ssr1	0.006222064	0.032469343
ENSMUSG00000022752	Tomm70a	0.006140397	7.18E-29
ENSMUSG00000057134	Ado	0.006068522	2.38E-05
ENSMUSG00000120473	Gm35742	0.00595429	2.76E-05
ENSMUSG00000019897	Ccdc59	0.005820144	1.69E-15
ENSMUSG00000024346	Pfdn1	0.005756387	0.005937245
ENSMUSG00000062014	Gmfb	0.005663075	0.000147745
ENSMUSG00000032293	Ireb2	0.005516377	6.00E-09
ENSMUSG00000028998	Tomm7	0.005431379	0.014678503
ENSMUSG00000028273	Pdlim5	0.00542954	0.006962127
ENSMUSG00000049354	Dcaf7	0.005173706	0.00128914
ENSMUSG00000051391	Ywhag	0.005032356	0.014621979
ENSMUSG00000026238	Ptma	0.004926815	0.001807062
ENSMUSG00000028085	Gatb	0.004926296	0.000519166
ENSMUSG00000030663	1110004F10	0.004793054	0.006796763
ENSMUSG00000032199	Polr2m	0.004552237	0.009282081
ENSMUSG00000040631	Dok4	0.004371374	2.17E-13
ENSMUSG00000057858	Fam204a	0.004354383	4.73E-06
ENSMUSG00000028792	Ak2	0.004224259	6.33E-34
ENSMUSG00000028159	Dapp1	0.004006484	0.01161518
ENSMUSG00000025060	Slk	0.003650142	9.32E-05
ENSMUSG00000030168	Adipor2	0.003607144	1.11E-10
ENSMUSG00000038648	Creb3l2	0.003245516	2.44E-05
ENSMUSG00000022892	App	0.003231519	5.19E-27
ENSMUSG00000029647	Pan3	0.00322931	1.77E-05
ENSMUSG00000078440	Dohh	0.003105765	0.001070734
ENSMUSG00000037106	Fer1l6	0.003014205	9.54E-10
ENSMUSG00000079494	Nat8f5	0.002770687	2.08E-24
ENSMUSG00000021759	Plpp1	0.002700178	2.46E-19
ENSMUSG00000006307	Kmt2b	0.002697503	0.001159611
ENSMUSG00000051224	Tceanc	0.002642911	0.006328797
ENSMUSG00000041629	Fam104a	0.002616732	0.000133295
ENSMUSG00000032652	Crebl2	0.002562015	0.010485435
ENSMUSG00000025921	Rdh10	0.002423786	7.95E-10
ENSMUSG00000024619	Cdx1	0.001987403	1.94E-26
ENSMUSG00000021096	Ppm1a	0.001111507	0.001433497
ENSMUSG00000030447	Cyfip1	0.001006773	0.019104762
ENSMUSG00000056941	Commd7	0.000976242	0.006017453
ENSMUSG00000004266	Ptpn6	0.000853263	0.014273307
ENSMUSG00000002015	Bcap31	0.000633892	4.11E-06
ENSMUSG00000024858	Grk2	0.000594778	0.002383016
ENSMUSG00000026032	Ndufb3	0.000487756	3.89E-12
ENSMUSG00000026918	Brd3	0.000463032	4.77E-05
ENSMUSG00000020477	Mrps24	0.000294618	4.81E-06

Table S3. Enriched gene clusters in the ileum of PD-fed mice

A) The 20 most significant GO terms downregulated in the ileum of PD-fed mice

Category	GO Term	Count	PValue	Genes
GOTERM_BP_FAT	immune response	99	1.70E-29	IFITM3, GIMAP3, SLA2, IFI44L, C4B, LGALS3, DMBT1, TRBC2, ZC3H12A, CD177, HERC6, ZBP1, CD96, GBP7, PRKCH, SP110, TAP1, IFIT1BL1, UNC13D, IGHG2B, CD8A, IGTP, CD226, TRIM15, SH2D1B1, SKAP1, IDO1, REG3B, CD274, NLRX1, CFI, H2-M3, PLA2G5, REG3G, CORO1A, C2, H2-EA, UBD, ST3GAL1, GPR17, H2-EB1, DEFA5, IRGM2, NKG7, IIGP1, IL7, KLRD1, H2-AB1, PIGR, CXCL9, CIITA, CD3G, CD3E, CD3D, IL18BP, DEFA41, CASP4, NFKBIZ, IGLC3, CD38, MBL2, APOBEC3, GPX2, MMP7, DEFA36, DEFA34, TLR1, LAT2, ZAP70, LCK, DOCK2, TLR2, SASH3, SLFN2, H2-DMA, WAS, MPTX2, CAPG, NOD1, CXCR6, USP18, CCL5, H2-DMB1, SERPINB9B, GBP2, GBP4, CD74, CD209B, PLA2G2D, GBP2B, NOS2, TRAT1, GZMB, H2-AA, ASS1, IL2RB, CD7, CD247, CCL28
GOTERM_BP_FAT	regulation of immune system process	88	5.38E-27	PIGR, GIMAP3, CD3E, SLA2, ETS1, C4B, LGALS3, TRBC2, CASP3, CASP4, ZC3H12A, NFKBIZ, IGLC3, CD38, CD177, MBL2, ZBP1, CD96, APOBEC3, GPX2, GBP7, PRKCH, ITGA4, TAP1, RHOH, IFIT1BL1, UNC13D, TLR1, LAT2, IGHG2B, ZAP70, LCK, CD8A, IGTP, PECAM1, CD226, TRIM15, SH2D1B1, HCST, SKAP1, TLR2, IDO1, SASH3, CD274, NLRX1, H2-DMA, H2-M3, 5730507C01RIK, CFI, WAS, DTX1, MPTX2, NOD1, PLA2G5, REG3G, IL2RG, USP18, CORO1A, C2, CNN2, H2-EA, THPO, H2-DMB1, CCL5, SERPINB9B, GBP2, GBP4, CD74, CAR2, GPR17, GBP2B, CD209B, PLA2G2D, H2-EB1, CDKN2A, TRAT1, TNFRSF9, GPR55, IRGM2, H2-AA, IL7, CEACAM10, KLRD1, CD247, CCL28, KLRH1, LGMN, H2-AB1
GOTERM_BP_FAT	response to biotic stimulus	80	7.55E-25	IFITM3, CXCL9, IFI44L, DEFA41, LGALS3, DMBT1, RGS1, TRBC2, CASP3, CASP4, ZC3H12A, NFKBIZ, CAPN2, IGLC3, MBL2, HERC6, ZBP1, CD52, CD96, APOBEC3, GPX2, GBP7, MMP7, SP110, ATG10, DEFA36, PLAAT3, SLC30A1, DEFA34, IFIT1BL1, LYPD8L, UNC13D, TLR1, IGHG2B, TTC39AOS1, CD8A, IGTP, CD226, TRIM15, LY6A, TLR2, IDO1, REG3B, CD274, NLRX1, SLFN2, SAA3, H2-M3, NOD1, PLA2G5, REG3G, USP18, C2, CCL5, SERPINB9B, GBP2, GBP4, DUOX2, GSTM3, GBP2B, CD209B, NOS2, 1810065E05RIK, SLC10A2, GZMA, DEFA5, IRGM2, GZMB, NKG7, AA467197, B3GALT5, LSM5, IIGP1, ASS1, PSMB9, LRG1, GSTA1, SAA1, CCL28, KLRH1
GOTERM_BP_FAT	response to other organism	78	1.30E-24	IFITM3, CXCL9, IFI44L, DEFA41, LGALS3, DMBT1, RGS1, TRBC2, CASP3, CASP4, ZC3H12A, NFKBIZ, CAPN2, IGLC3, MBL2, HERC6, ZBP1, CD52, CD96, APOBEC3, GPX2, GBP7, MMP7, SP110, DEFA36, PLAAT3, SLC30A1, DEFA34, IFIT1BL1, LYPD8L, UNC13D, TLR1, IGHG2B, TTC39AOS1, CD8A, IGTP, TRIM15, LY6A, TLR2, IDO1, REG3B, CD274, NLRX1, SLFN2, SAA3, H2-M3, NOD1, PLA2G5, REG3G, USP18, C2, CCL5, SERPINB9B, GBP2, GBP4, DUOX2, GSTM3, GBP2B, CD209B, NOS2, 1810065E05RIK, SLC10A2, GZMA, DEFA5, IRGM2, GZMB, NKG7, AA467197, B3GALT5, LSM5, IIGP1, ASS1, PSMB9, LRG1, GSTA1, SAA1, CCL28, KLRH1
GOTERM_BP_FAT	response to external biotic stimulus	78	1.52E-24	IFITM3, CXCL9, IFI44L, DEFA41, LGALS3, DMBT1, RGS1, TRBC2, CASP3, CASP4, ZC3H12A, NFKBIZ, CAPN2, IGLC3, MBL2, HERC6, ZBP1, CD52, CD96, APOBEC3, GPX2, GBP7, MMP7, SP110, DEFA36, PLAAT3, SLC30A1, DEFA34, IFIT1BL1, LYPD8L, UNC13D, TLR1, IGHG2B, TTC39AOS1, CD8A, IGTP, TRIM15, LY6A, TLR2, IDO1, REG3B, CD274, NLRX1, SLFN2, SAA3, H2-M3, NOD1, PLA2G5, REG3G, USP18, C2, CCL5, SERPINB9B, GBP2, GBP4, DUOX2, GSTM3, GBP2B, CD209B, NOS2, 1810065E05RIK, SLC10A2, GZMA, DEFA5, IRGM2, GZMB, NKG7, AA467197, B3GALT5, LSM5, IIGP1, ASS1, PSMB9, LRG1, GSTA1, SAA1, CCL28, KLRH1
GOTERM_BP_FAT	defense response	87	1.88E-23	IFITM3, CXCL9, CIITA, GIMAP3, ETS1, IFI44L, DEFA41, C4B, LGALS3, DMBT1, TRBC2, CASP4, ZC3H12A, NFKBIZ, DUOXA2, IGLC3, CD177, MBL2, HERC6, ZBP1, CD96, APOBEC3, GPX2, GBP7, MMP7, SP110, DEFA36, TAP1, SLC30A1, DEFA34, IFIT1BL1, UNC13D, TLR1, IGHG2B, ZAP70, TTC39AOS1, LCK, CD8A, IGTP, CD226, TRIM15, SH2D1B1, TLR2, IDO1, REG3B, NLRX1, AKNA, SAA3, H2-M3, CFI, WAS, MPTX2, CAPG, NOD1, CXCR6, PLA2G5, REG3G, USP18, CORO1A, C2, NT5E, UBD, CCL5, SERPINB9B, GBP2, GM5431, GBP4, DUOX2, CD74, GPR17, GBP2B, PLA2G2D, H2-EB1, NOS2, DEFA5, IRGM2, GZMB, NKG7, IFI47, H2-AA, IIGP1, ASS1, ALOX5AP, SAA1, KLRD1, KLRH1, H2-AB1
GOTERM_BP_FAT	response to external stimulus	110	2.54E-23	IFITM3, ETS1, IFI44L, LGALS3, DMBT1, RGS1, TRBC2, ZC3H12A, DUOXA2, CAPN2, HERC6, ZBP1, CD96, GBP7, PRKCH, SP110, SLC30A1, IFIT1BL1, UNC13D, IGHG2B, HOXB9, TTC39AOS1, BIN2, CD8A, IGTP, TRIM15, LY6A, IDO1, REG3B, CD274, NLRX1, AKNA, CFI, H2-M3, PLA2G5, REG3G, CORO1A, C2, CNN2, SLC38A3, GSTM3, GPR17, OPN3, 1810065E05RIK, SLC10A2, DEFA5, IRGM2, MTHFR, NKG7, ASNS, AA467197, B3GALT5, LSM5, IIGP1, BGLAP3, LGMN, CXCL9, CD3E, DEFA41, C1QTNF1, CASP3, CASP4, NFKBIZ, IGLC3, UPP1, MBL2, CD52, APOBEC3, GPX2, MMP7, DEFA36, RHOH, PLAAT3, DEFA34, LYPD8L, TLR1, TMIGD1, LCK, DOCK2, TLR2, SLFN2, SAA3, NOD1, CXCR6, USP18, NT5E, CCL5, SLITRK6, SERPINB9B, GBP2, GBP4, DUOX2, CD74, CD209B, PLA2G2D, GBP2B, NOS2, GZMA, PDX1, GZMB, BMP8B, SUOX, ASS1, PSMB9, LRG1, GSTA1, ALOX5AP, SAA1, CCL28, KLRH1
GOTERM_BP_FAT	regulation of immune response	63	5.58E-22	PIGR, GIMAP3, CD3E, SLA2, C4B, LGALS3, TRBC2, CASP4, ZC3H12A, NFKBIZ, IGLC3, CD38, CD177, MBL2, ZBP1, CD96, GPX2, GBP7, PRKCH, TAP1, UNC13D, TLR1, LAT2, IGHG2B, ZAP70, LCK, CD8A, IGTP, CD226, TRIM15, SH2D1B1, HCST, SKAP1, TLR2, IDO1, SASH3, CD274, NLRX1, H2-DMA, H2-M3, CFI, WAS, MPTX2, NOD1, PLA2G5, REG3G, USP18, C2, H2-EA, H2-DMB1, SERPINB9B, GBP2, CD74, GPR17, GBP2B, PLA2G2D, H2-EB1, TRAT1, IRGM2, H2-AA, KLRD1, CD247, H2-AB1
GOTERM_BP_FAT	response to bacterium	63	8.46E-21	CXCL9, DEFA41, DMBT1, RGS1, TRBC2, CASP3, CASP4, ZC3H12A, NFKBIZ, CAPN2, IGLC3, MBL2, HERC6, CD52, CD96, GPX2, GBP7, MMP7, SP110, DEFA36, PLAAT3, SLC30A1, DEFA34, LYPD8L, TLR1, IGHG2B, TTC39AOS1, IGTP, LY6A, TLR2, IDO1, REG3B, CD274, SLFN2, SAA3, H2-M3, NOD1, REG3G, USP18, C2, CCL5, SERPINB9B, GBP2, GBP4, GSTM3, GBP2B, CD209B, NOS2, 1810065E05RIK, SLC10A2, GZMA, DEFA5, IRGM2, GZMB, AA467197, B3GALT5, LSM5, IIGP1, ASS1, PSMB9, LRG1, GSTA1, SAA1
GOTERM_BP_FAT	innate immune response	62	1.18E-20	IFITM3, CIITA, GIMAP3, DEFA41, C4B, LGALS3, TRBC2, CASP4, NFKBIZ, IGLC3, CD177, MBL2, HERC6, ZBP1, CD96, APOBEC3, GBP7, SP110, DEFA36, TAP1, DEFA34, IFIT1BL1, UNC13D, TLR1, IGHG2B, ZAP70, LCK, IGTP, CD226, TRIM15, SH2D1B1, TLR2, NLRX1, H2-M3, CFI, WAS, MPTX2, CAPG, NOD1, PLA2G5, REG3G, USP18, CORO1A, C2, UBD, CCL5, SERPINB9B, GBP2, GBP4, CD74, GBP2B, H2-EB1, NOS2, DEFA5, IRGM2, GZMB, NKG7, H2-AA, IIGP1, ASS1, KLRD1, H2-AB1
GOTERM_BP_FAT	positive regulation of immune system process	66	1.55E-20	GIMAP3, CD3E, SLA2, ETS1, C4B, LGALS3, TRBC2, CASP4, ZC3H12A, NFKBIZ, IGLC3, CD38, CD177, MBL2, ZBP1, GBP7, PRKCH, ITGA4, RHOH, UNC13D, TLR1, LAT2, IGHG2B, ZAP70, LCK, CD8A, IGTP, PECAM1, CD226, TRIM15, SH2D1B1, SKAP1, TLR2, IDO1, SASH3, CD274, NLRX1, H2-DMA, H2-M3, CFI, MPTX2, NOD1, PLA2G5, REG3G, IL2RG, CORO1A, C2, H2-EA, THPO, H2-DMB1, CCL5, GBP2, CD74, CAR2, GBP2B, CD209B, H2-EB1, TRAT1, IRGM2, H2-AA, IL7, KLRD1, CD247, KLRH1, LGMN, H2-AB1
GOTERM_BP_FAT	leukocyte cell-cell adhesion	39	1.67E-18	SASH3, CD274, SELPLG, H2-DMA, GIMAP3, DTX1, PLA2G5, CD3E, IL2RG, CORO1A, ETS1, LGALS3, H2-EA, NT5E, CASP3, CCL5, H2-DMB1, ZC3H12A, NFKBIZ, CD177, CD74, PLA2G2D, CD209B, H2-EB1, ITGA4, CDKN2A, TNFRSF9, RHOH, H2-AA, ASS1, ZAP70, LRG1, IL7, LCK, PECAM1, CCL28, SKAP1, H2-AB1, IDO1
GOTERM_BP_FAT	positive regulation of immune response	52	3.41E-18	GIMAP3, CD3E, SLA2, C4B, LGALS3, TRBC2, CASP4, ZC3H12A, NFKBIZ, IGLC3, CD38, CD177, MBL2, ZBP1, GBP7, PRKCH, TLR1, LAT2, IGHG2B, ZAP70, LCK, CD8A, IGTP, CD226, TRIM15, SH2D1B1, SKAP1, TLR2, IDO1, SASH3, CD274, NLRX1, H2-DMA, H2-M3, CFI, MPTX2, NOD1, PLA2G5, REG3G, C2, H2-EA, H2-DMB1, GBP2, CD74, GBP2B, H2-EB1, TRAT1, IRGM2, H2-AA, KLRD1, CD247, H2-AB1
GOTERM_BP_FAT	immune effector process	55	1.42E-16	IFITM3, CXCL9, GIMAP3, IFI44L, C4B, LGALS3, TRBC2, CASP4, ZC3H12A, NFKBIZ, IGLC3, CD177, MBL2, ZBP1, CD96, APOBEC3, GBP7, TAP1, IFIT1BL1, UNC13D, LAT2, IGHG2B, CD8A, IGTP, CD226, TRIM15, SH2D1B1, DOCK2, TLR2, SASH3, NLRX1, SLFN2, H2-DMA, H2-M3, CFI, WAS, MPTX2, NOD1, PLA2G5, CORO1A, C2, H2-EA, H2-DMB1, CCL5, SERPINB9B, ST3GAL1, GBP4, CD74, GBP2B, IRGM2, GZMB, NKG7, IL2RB, KLRD1, H2-AB1
GOTERM_BP_FAT	regulation of leukocyte cell-cell adhesion	34	2.12E-15	SASH3, CD274, H2-DMA, GIMAP3, DTX1, PLA2G5, CD3E, IL2RG, CORO1A, ETS1, LGALS3, H2-EA, CASP3, CCL5, H2-DMB1, ZC3H12A, NFKBIZ, CD74, PLA2G2D, CD209B, H2-EB1, ITGA4, CDKN2A, TNFRSF9, RHOH, H2-AA, ASS1, ZAP70, IL7, LCK, CCL28, SKAP1, H2-AB1, IDO1
GOTERM_BP_FAT	leukocyte activation	57	2.81E-15	GIMAP3, CD3G, CD3E, SLA2, CD3D, LGALS3, TRBC2, CASP3, ZC3H12A, NFKBIZ, IGLC3, CD38, CD177, RHOH, UNC13D, TLR1, LAT2, IGHG2B, ZAP70, LCK, CD8A, IGTP, LY6D, CD226, SH2D1B1, DOCK2, TLR2, IDO1, SASH3, CD274, SELPLG, SLFN2, H2-DMA, H2-M3, WAS, DTX1, PLA2G5, IL2RG, CORO1A, H2-EA, H2-DMB1, UBD, CCL5, ST3GAL1, CD74, CD209B, PLA2G2D, H2-EB1, CDKN2A, TNFRSF9, NKG7, H2-AA, IL7, IL2RB, KLRD1, KLRH1, H2-AB1
GOTERM_BP_FAT	regulation of cell-cell adhesion	37	6.33E-15	SASH3, CD274, AKNA, H2-DMA, GIMAP3, DTX1, PLA2G5, CD3E, IL2RG, ZDHHC2, CORO1A, ETS1, LGALS3, H2-EA, C1QTNF1, CASP3, CCL5, H2-DMB1, ZC3H12A, NFKBIZ, CD74, PLA2G2D, CD209B, H2-EB1, ITGA4, CDKN2A, TNFRSF9, RHOH, H2-AA, ASS1, ZAP70, IL7, LCK, CCL28, SKAP1, H2-AB1, IDO1
GOTERM_BP_FAT	regulation of defense response	45	6.34E-15	NLRX1, AKNA, H2-M3, CFI, GIMAP3, NOD1, PLA2G5, REG3G, USP18, ETS1, NT5E, CCL5, CASP4, ZC3H12A, NFKBIZ, DUOXA2, SERPINB9B, GBP2, GBP4, MBL2, ZBP1, CD96, GPR17, CD74, GBP2B, PLA2G2D, APOBEC3, GPX2, GBP7, NOS2, IRGM2, TAP1, NKG7, IFIT1BL1, TLR1, IGHG2B, ALOX5AP, IGTP, KLRD1, CD226, TRIM15, SH2D1B1, KLRH1, TLR2, IDO1
GOTERM_BP_FAT	cell killing	28	6.96E-15	CXCL9, H2-M3, GIMAP3, REG3G, CORO1A, DEFA41, LGALS3, H2-EA, SERPINB9B, GBP2, MBL2, GBP2B, GBP7, NOS2, GZMA, DEFA5, IRGM2, GZMB, TAP1, NKG7, DEFA34, UNC13D, IGTP, KLRD1, CD226, SH2D1B1, CCL28, KLRH1
GOTERM_BP_FAT	adaptive immune response	43	9.61E-15	SASH3, CD274, SLFN2, H2-DMA, H2-M3, CFI, WAS, GIMAP3, MPTX2, CD3G, CD3E, SLA2, CD3D, IL18BP, C2, C4B, H2-EA, H2-DMB1, TRBC2, ZC3H12A, NFKBIZ, IGLC3, SERPINB9B, MBL2, CD74, H2-EB1, TRAT1, GZMB, TAP1, H2-AA, UNC13D, LAT2, IGHG2B, ZAP70, CD8A, CD7, IL2RB, KLRD1, CD226, CD247, SH2D1B1, SKAP1, H2-AB1

B) The 20 most significant pathways downregulated in the ileum of PD-fed mice

Category	Term	Count	PValue	Genes
KEGG_PATHWAY	Hematopoietic cell lineage	15	3.40E-10	H2-EB1, ITGA4, H2-DMA, CD3G, CD3E, H2-AA, CD3D, H2-EA, THPO, IL7, CD8A, H2-DMB1, CD7, CD38, H2-AB1
KEGG_PATHWAY	Th1 and Th2 cell differentiation	14	1.66E-09	H2-EB1, H2-DMA, CD3G, CD3E, IL2RG, H2-AA, CD3D, H2-EA, ZAP70, LCK, H2-DMB1, IL2RB, CD247, H2-AB1
KEGG_PATHWAY	Th17 cell differentiation	14	1.52E-08	H2-EB1, H2-DMA, CD3G, CD3E, IL2RG, H2-AA, CD3D, H2-EA, ZAP70, LCK, H2-DMB1, IL2RB, CD247, H2-AB1
KEGG_PATHWAY	Staphylococcus aureus infection	15	2.10E-08	H2-EB1, SELPLG, H2-DMA, CFI, DEFA5, DEFA36, DEFA34, H2-AA, C2, DEFA41, C4B, H2-EA, H2-DMB1, MBL2, H2-AB1
KEGG_PATHWAY	Antigen processing and presentation	13	2.39E-08	CD74, CIITA, H2-EB1, H2-DMA, H2-M3, TAP1, H2-AA, H2-EA, CD8A, H2-DMB1, KLRD1, LGMN, H2-AB1
KEGG_PATHWAY	Leishmaniasis	11	1.89E-07	H2-EA, H2-EB1, MARCKSL1, ITGA4, NOS2, H2-DMA, H2-DMB1, NCF4, H2-AA, H2-AB1, TLR2
KEGG_PATHWAY	Intestinal immune network for IgA production	9	4.06E-07	H2-EA, PIGR, H2-EB1, ITGA4, H2-DMA, H2-DMB1, H2-AA, CCL28, H2-AB1
KEGG_PATHWAY	Human T-cell leukemia virus 1 infection	18	7.09E-07	H2-EB1, MMP7, CDKN2A, H2-DMA, H2-M3, RASL2-9, ADCY4, CD3G, CD3E, IL2RG, H2-AA, CD3D, ETS1, H2-EA, LCK, H2-DMB1, IL2RB, H2-AB1
KEGG_PATHWAY	Primary immunodeficiency	8	1.62E-06	ZAP70, CIITA, LCK, CD8A, TAP1, CD3E, IL2RG, CD3D
KEGG_PATHWAY	Toxoplasmosis	12	1.80E-06	H2-EA, H2-EB1, CIITA, NOS2, H2-DMA, H2-DMB1, CASP3, IGTP, IRGM2, H2-AA, H2-AB1, TLR2
KEGG_PATHWAY	Tuberculosis	14	8.07E-06	CD74, CIITA, CD209B, H2-EB1, NOS2, H2-DMA, H2-AA, CORO1A, TLR1, H2-EA, CASP3, H2-DMB1, H2-AB1, TLR2
KEGG_PATHWAY	Graft-versus-host disease	9	8.21E-06	H2-EA, H2-EB1, H2-DMA, H2-DMB1, H2-M3, GZMB, KLRD1, H2-AA, H2-AB1
KEGG_PATHWAY	Cell adhesion molecules	14	9.10E-06	CD274, H2-EB1, SELPLG, ITGA4, H2-DMA, H2-M3, H2-AA, H2-EA, CD8A, H2-DMB1, SLITRK6, PECAM1, CD226, H2-AB1
KEGG_PATHWAY	Phagosome	13	4.51E-05	CD209B, H2-EB1, H2-DMA, H2-M3, NCF4, TAP1, H2-AA, CORO1A, H2-EA, H2-DMB1, MBL2, H2-AB1, TLR2
KEGG_PATHWAY	Asthma	6	4.66E-05	H2-EA, H2-EB1, H2-DMA, H2-DMB1, H2-AA, H2-AB1
KEGG_PATHWAY	Inflammatory bowel disease	8	6.67E-05	H2-EA, H2-EB1, H2-DMA, H2-DMB1, IL2RG, H2-AA, H2-AB1, TLR2
KEGG_PATHWAY	Allograft rejection	8	7.40E-05	H2-EA, H2-EB1, H2-DMA, H2-DMB1, H2-M3, GZMB, H2-AA, H2-AB1
KEGG_PATHWAY	Epstein-Barr virus infection	14	1.11E-04	H2-EB1, H2-DMA, H2-M3, TAP1, CD3G, CD3E, H2-AA, CD3D, H2-EA, CASP3, H2-DMB1, CD247, H2-AB1, TLR2
KEGG_PATHWAY	Type I diabetes mellitus	8	1.46E-04	H2-EA, H2-EB1, H2-DMA, H2-DMB1, H2-M3, GZMB, H2-AA, H2-AB1
KEGG_PATHWAY	Autoimmune thyroid disease	8	3.11E-04	H2-EA, H2-EB1, H2-DMA, H2-DMB1, H2-M3, GZMB, H2-AA, H2-AB1

C) The 20 most significant GO terms upregulated in the ileum of PD-fed mice

Category	Term	Count	PValue	Genes
GOTERM_BP_FAT	lipid metabolic process	90	6.41E-31	ACAA2, ABCD3, AQP8, RDH7, ZBTB20, ACSM5, LIPA, PLB1, CES2B, EDNRB, CES2G, CYP4V3, PDK4, HMGCS2, SCD1, CD36, GAL3ST2, PDGFRB, PDGFRA, CUBN, SREBF1, ABCA8A, ABCA8B, CYP4A10, SPHK1, ACSL6, GIP, CYP2D26, ALDH3A2, RDH16F2, ALDH1A3, POR, BDH2, CYP2J13, CYP2U1, EHHADH, ALDH1A1, CAT, RGN, CYP2C40, ACOT2, ACOT1, TRIB3, ANGPTL4, GAL3ST2B, PPARA, LCT, SLC27A2, MGLL, B4GALT5, ACOT3, RETSAT, HSD17B4, GDPD2, ACAA1B, HSD17B13, FITM2, AFP, LTC4S, HSD17B11, CYP17A1, PTGS1, SEC14L2, ADH4, ADH1, HAO2, CCL21A, WNT4, ABCA1, CYP2J6, HSD3B3, EPHX2, CYP2J9, NR1D1, EFR3B, DHRS3, FABP1, FGF15, NCEH1, CYP2C23, FABP4, CYP2C66, AADAC, CES1D, CYP2C65, P2RX1, CES1G, PXMP4, FGFR4, GSTM7
GOTERM_BP_FAT	monocarboxylic acid metabolic process	61	4.54E-28	ACAA2, ABCD3, ZBTB20, ACSM5, LIPA, CES2B, CES2G, CYP4V3, PDK4, ME1, UGT2A3, SCD1, CD36, SREBF1, ABCC2, CYP4A10, SPHK1, ACSL6, GIP, CYP2D26, ALDH3A2, RDH16F2, ALDH1A3, POR, BDH2, CYP2J13, CYP2U1, EHHADH, ALDH1A1, RGN, CYP2C40, ACOT2, ACOT1, TRIB3, PPARA, SLC27A2, MGLL, ACOT3, HSD17B4, SLC1A3, ACAA1B, LTC4S, PTGS1, ADH4, ADH1, HAO2, CYP2J6, EPHX2, CYP2J9, NR1D1, FABP1, FGF15, VNN1, CYP2C23, FABP4, CYP2C66, CES1D, CYP2C65, CES1G, FGFR4, GSTM7
GOTERM_BP_FAT	cellular lipid metabolic process	73	6.13E-27	ACAA2, ABCD3, RDH7, ACSM5, LIPA, PLB1, CES2B, CES2G, CYP4V3, PDK4, HMGCS2, SCD1, CD36, GAL3ST2, PDGFRB, PDGFRA, SREBF1, ABCA8A, ABCA8B, CYP4A10, SPHK1, ACSL6, GIP, CYP2D26, ALDH3A2, RDH16F2, ALDH1A3, POR, BDH2, CYP2J13, CYP2U1, EHHADH, ALDH1A1, CAT, RGN, CYP2C40, ACOT2, ACOT1, TRIB3, GAL3ST2B, PPARA, LCT, SLC27A2, MGLL, B4GALT5, ACOT3, RETSAT, HSD17B4, ACAA1B, FITM2, LTC4S, PTGS1, ADH4, ADH1, HAO2, CCL21A, CYP2J6, EPHX2, CYP2J9, EFR3B, DHRS3, FABP1, NCEH1, CYP2C23, FABP4, CYP2C66, AADAC, CES1D, CYP2C65, P2RX1, CES1G, PXMP4, GSTM7
GOTERM_BP_FAT	fatty acid metabolic process	48	6.41E-25	ABCD3, ACAA2, HSD17B4, ACAA1B, ACSM5, LTC4S, LIPA, PTGS1, CES2B, ADH4, CES2G, CYP4V3, PDK4, CD36, SCD1, HAO2, SREBF1, CYP2J6, EPHX2, SPHK1, CYP4A10, CYP2J9, ACSL6, GIP, CYP2D26, FABP1, ALDH3A2, POR, BDH2, CYP2C23, FABP4, CYP2C66, CYP2U1, CES1D, CYP2J13, CYP2C65, EHHADH, CES1G, ACOT2, RGN, CYP2C40, ACOT1, TRIB3, PPARA, GSTM7, SLC27A2, MGLL, ACOT3
GOTERM_BP_FAT	carboxylic acid metabolic process	65	4.91E-23	ACAA2, ABCD3, ZBTB20, ACSM5, LIPA, CES2B, IYD, CES2G, CYP4V3, PDK4, ME1, UGT2A3, SCD1, CD36, SREBF1, ABCC2, CYP4A10, SPHK1, ACSL6, RENBP, GIP, CYP2D26, ALDH3A2, RDH16F2, ALDH1A3, POR, BDH2, CYP2J13, CYP2U1, TST, EHHADH, ALDH1A1, RGN, CYP2C40, ACOT2, ACOT1, TRIB3, PPARA, SLC27A2, MGLL, ACOT3, HSD17B4, SLC1A3, ACAA1B, NPL, LTC4S, PTGS1, ADH4, ADH1, HAO2, CYP2J6, EPHX2, CYP2J9, NR1D1, FABP1, FGF15, VNN1, CYP2C23, FABP4, CYP2C66, CES1D, CYP2C65, CES1G, FGFR4, GSTM7
GOTERM_BP_FAT	oxoacid metabolic process	67	1.44E-22	ACAA2, ABCD3, ZBTB20, ACSM5, LIPA, CES2B, EDNRB, IYD, CES2G, CYP4V3, PDK4, ME1, UGT2A3, SCD1, CD36, SREBF1, ABCC2, CYP4A10, SPHK1, ACSL6, RENBP, GIP, CYP2D26, ALDH3A2, RDH16F2, ALDH1A3, POR, BDH2, CYP2J13, CYP2U1, TST, EHHADH, ALDH1A1, RGN, CYP2C40, ACOT2, ACOT1, TRIB3, PPARA, SLC27A2, MGLL, ACOT3, MTARC1, HSD17B4, SLC1A3, ACAA1B, NPL, LTC4S, PTGS1, ADH4, ADH1, HAO2, CYP2J6, EPHX2, CYP2J9, NR1D1, FABP1, FGF15, VNN1, CYP2C23, FABP4, CYP2C66, CES1D, CYP2C65, CES1G, FGFR4, GSTM7
GOTERM_BP_FAT	organic acid metabolic process	67	3.22E-22	ACAA2, ABCD3, ZBTB20, ACSM5, LIPA, CES2B, EDNRB, IYD, CES2G, CYP4V3, PDK4, ME1, UGT2A3, SCD1, CD36, SREBF1, ABCC2, CYP4A10, SPHK1, ACSL6, RENBP, GIP, CYP2D26, ALDH3A2, RDH16F2, ALDH1A3, POR, BDH2, CYP2J13, CYP2U1, TST, EHHADH, ALDH1A1, RGN, CYP2C40, ACOT2, ACOT1, TRIB3, PPARA, SLC27A2, MGLL, ACOT3, MTARC1, HSD17B4, SLC1A3, ACAA1B, NPL, LTC4S, PTGS1, ADH4, ADH1, HAO2, CYP2J6, EPHX2, CYP2J9, NR1D1, FABP1, FGF15, VNN1, CYP2C23, FABP4, CYP2C66, CES1D, CYP2C65, CES1G, FGFR4, GSTM7
GOTERM_BP_FAT	long-chain fatty acid metabolic process	23	3.81E-17	ABCD3, CYP2J6, EPHX2, SPHK1, CYP4A10, CYP2J9, ACSL6, LTC4S, CYP2D26, PTGS1, CYP2C23, CYP2C66, CYP2J13, CYP2U1, CYP2C65, ACOT2, CYP2C40, ACOT1, CD36, GSTM7, SLC27A2, MGLL, ACOT3
GOTERM_BP_FAT	lipid biosynthetic process	42	1.93E-14	ABCD3, AQP8, ZBTB20, HSD17B13, ACSM5, FITM2, LTC4S, HSD17B11, LIPA, CYP17A1, PTGS1, SEC14L2, PDK4, HMGCS2, SCD1, GAL3ST2, WNT4, SREBF1, ABCA8A, HSD3B3, ABCA8B, SPHK1, ACSL6, NR1D1, GIP, RDH16F2, ALDH1A3, FGF15, POR, CES1D, P2RX1, CES1G, ALDH1A1, RGN, TRIB3, PPARA, GAL3ST2B, FGFR4, GSTM7, SLC27A2, MGLL, B4GALT5
GOTERM_BP_FAT	regulation of hormone levels	40	1.06E-13	SLC22A3, RETSAT, RDH7, WNK4, ITPR1, HSD17B4, SLC2A2, AFP, HSD17B11, PLB1, CYP17A1, PTGS1, GHR, NEUROD1, ADH4, ADH1, GJA1, EDNRB, CLTRN, IYD, WNT4, CHST8, GPR39, PDGFRA, SREBF1, ABCC2, HSD3B3, FZD4, OPRK1, NR1D1, DHRS3, GIP, RDH16F2, ALDH1A3, SFRP1, POR, SLC7A8, ALDH1A1, NMU, FGFR4
GOTERM_BP_FAT	organic hydroxy compound metabolic process	36	2.41E-13	ABCD3, MAOB, RETSAT, AQP8, RDH7, ACAA1B, LIPA, SLC6A3, PLB1, SEC14L2, ADH4, ADH1, EDNRB, IYD, HMGCS2, HAO2, WNT4, ABCA1, CUBN, SREBF1, SPHK1, NR1D1, DHRS3, ALDH3A2, RDH16F2, ALDH1A3, FGF15, POR, CES1D, CAT, CES1G, ALDH1A1, FGFR4, LCT, SLC27A2, ALDH1A7
GOTERM_BP_FAT	steroid metabolic process	29	1.12E-12	ABCD3, AQP8, RDH7, HSD17B4, ACAA1B, AFP, HSD17B11, LIPA, CYP17A1, SEC14L2, EDNRB, HMGCS2, SCD1, WNT4, ABCA1, PDGFRA, CUBN, SREBF1, HSD3B3, EPHX2, NR1D1, RDH16F2, FGF15, POR, CES1D, CAT, CES1G, FGFR4, SLC27A2
GOTERM_BP_FAT	regulation of lipid metabolic process	31	3.72E-12	AQP8, ZBTB20, HSD17B13, FITM2, CYP17A1, SEC14L2, PDK4, CD36, SCD1, CCL21A, WNT4, PDGFRB, GPR39, PDGFRA, SREBF1, EPHX2, SPHK1, NR1D1, GIP, FABP1, RDH16F2, FGF15, POR, CES1D, AADAC, CES1G, RGN, TRIB3, ANGPTL4, PPARA, FGFR4
GOTERM_BP_FAT	response to xenobiotic stimulus	33	1.27E-11	ABCD3, MAOB, WNK4, SLC1A3, ACAA1B, LIPA, SLC6A3, CRYZ, NEUROD1, NCAM1, HMGCS2, BCHE, SREBF1, ABCC2, CYP2J6, CYP2J9, MMP9, GIP, CYP2D26, SFRP1, POR, NCEH1, CYP2C23, CYP2C66, CYP2U1, CYP2J13, CYP2C65, CAT, ALDH1A1, CYP2C40, PLIN2, LCT, GSTM7
GOTERM_BP_FAT	positive regulation of lipid metabolic process	21	5.55E-11	PDGFRB, PDGFRA, SREBF1, ZBTB20, HSD17B13, NR1D1, CYP17A1, FABP1, SEC14L2, RDH16F2, POR, AADAC, CES1D, CES1G, RGN, CD36, ANGPTL4, SCD1, PPARA, CCL21A, WNT4
GOTERM_BP_FAT	unsaturated fatty acid metabolic process	19	1.07E-10	CYP2J6, EPHX2, SPHK1, CYP4A10, CYP2J9, LTC4S, CYP2D26, PTGS1, CES2B, CYP2C23, CYP2C66, CYP2J13, CYP2U1, CYP2C65, CES2G, CYP2C40, SCD1, GSTM7, MGLL
GOTERM_BP_FAT	regulation of lipid biosynthetic process	21	2.57E-10	SREBF1, AQP8, SPHK1, ZBTB20, HSD17B13, FITM2, NR1D1, GIP, CYP17A1, SEC14L2, RDH16F2, POR, FGF15, CES1D, CES1G, PDK4, RGN, TRIB3, PPARA, FGFR4, WNT4
GOTERM_BP_FAT	cellular hormone metabolic process	17	2.74E-10	PDGFRA, RETSAT, HSD3B3, RDH7, HSD17B4, AFP, HSD17B11, DHRS3, PLB1, CYP17A1, RDH16F2, ALDH1A3, ADH4, ADH1, EDNRB, ALDH1A1, WNT4
GOTERM_BP_FAT	hormone metabolic process	21	6.69E-10	CHST8, PDGFRA, RETSAT, HSD3B3, RDH7, HSD17B4, AFP, HSD17B11, DHRS3, PLB1, CYP17A1, GHR, RDH16F2, ALDH1A3, ADH4, ADH1, POR, EDNRB, IYD, ALDH1A1, WNT4
GOTERM_BP_FAT	alcohol metabolic process	24	9.59E-10	ABCA1, CUBN, SREBF1, RETSAT, AQP8, SPHK1, RDH7, LIPA, DHRS3, PLB1, SEC14L2, ALDH3A2, RDH16F2, ALDH1A3, ADH4, ADH1, POR, CES1D, CAT, CES1G, ALDH1A1, HMGCS2, FGFR4, ALDH1A7

D) The 20 most significant pathways upregulated in the ileum of PD-fed mice

Category	Pathway	Count	PValue	Genes
KEGG_PATHWAY	PPAR signaling pathway	15	5.63E-10	CYP4A10, ACSL6, AQP7, ACAA1B, FABP1, FABP4, EHHADH, ME1, PLIN2, HMGCS2, CD36, ANGPTL4, SCD1, PPARA, SLC27A2

KEGG_PATHWAY	Retinol metabolism	15	1.82E-09	RETSAT, CYP4A10, RDH7, DHRS3, RDH16F2, ALDH1A3, ADH4, ADH1, CYP2C23, CYP2C66, CYP2C65, ALDH1A1, CYP2C40, UGT2A3, ALDH1A7
KEGG_PATHWAY	Arachidonic acid metabolism	13	3.55E-08	CYP2J6, EPHX2, CYP4A10, CYP2J9, LTC4S, PLB1, PTGS1, CYP2C23, CYP2C66, CYP2J13, CYP2U1, CYP2C65, CYP2C40
KEGG_PATHWAY	Metabolic pathways	58	1.54E-07	CDA, ACAA2, RDH7, ACSM5, SAT2, PLB1, ME1, UGT2A3, HMGCS2, SCD1, CHST8, GUCY1A1, CYP4A10, SPHK1, ACSL6, RENBP, ALDH3A2, RDH16F2, ALDH1A3, CYP2J13, CYP2U1, TST, EHHADH, ALDH1A1, CAT, RGN, CYP2C40, ACOT2, ACOT1, LCT, ALDH1A7, MGLL, B4GALT5, ACOT3, MAOB, HSD17B4, PLD4, ACAA1B, NPL, LTC4S, CYP17A1, PTGS1, ADH4, ADH1, PRDM16, HAO2, CYP2J6, HSD3B3, EPHX2, CYP2J9, BBOX1, DHRS3, VNN1, CYP2C23, CYP2C66, CYP2C65, GSTM7, GSTM6
KEGG_PATHWAY	Fatty acid degradation	9	3.77E-06	ALDH3A2, ADH4, ADH1, ACAA2, CYP2U1, EHHADH, CYP4A10, ACSL6, ACAA1B
KEGG_PATHWAY	Peroxisome	10	2.37E-05	ABCD3, EHHADH, EPHX2, CAT, ACSL6, HSD17B4, PXMP4, ACAA1B, HAO2, SLC27A2
KEGG_PATHWAY	Linoleic acid metabolism	8	2.94E-05	CYP2C23, CYP2C66, CYP2J6, CYP2J13, CYP2C65, CYP2J9, CYP2C40, PLB1
KEGG_PATHWAY	Serotonergic synapse	11	1.28E-04	CYP2C23, CYP2C66, MAOB, CYP2J6, CYP2J13, CYP2C65, CYP2J9, ITPR1, CYP2C40, CYP2D26, PTGS1
KEGG_PATHWAY	Ovarian steroidogenesis	8	1.34E-04	CYP2J6, CYP2J13, HSD3B3, CYP2J9, ACOT2, ACOT1, CYP17A1, ACOT3
KEGG_PATHWAY	Steroid hormone biosynthesis	9	2.71E-04	CYP2C23, CYP2C66, HSD3B3, CYP2C65, CYP2C40, UGT2A3, HSD17B11, CYP17A1, CYP2D26
KEGG_PATHWAY	Biosynthesis of unsaturated fatty acids	6	3.35E-04	ACOT2, HSD17B4, ACOT1, ACAA1B, SCD1, ACOT3
KEGG_PATHWAY	Inflammatory mediator regulation of TRP channels	10	4.85E-04	CYP2C23, CYP2C66, CYP2J6, CYP2J13, CYP2C65, CYP4A10, CYP2J9, ITPR1, CYP2C40, IL1RAP
KEGG_PATHWAY	Chemical carcinogenesis - DNA adducts	7	0.00411	CYP2C23, CYP2C66, CYP2C65, CYP2C40, UGT2A3, GSTM7, GSTM6
KEGG_PATHWAY	Fatty acid metabolism	6	0.005189	ACAA2, EHHADH, ACSL6, HSD17B4, ACAA1B, SCD1
KEGG_PATHWAY	Basal cell carcinoma	6	0.005555	BMP4, FZD2, GADD45B, FZD4, HHIP, WNT4
KEGG_PATHWAY	Drug metabolism - other enzymes	7	0.006406	CDA, CES2B, CES1D, CES2G, UGT2A3, GSTM7, GSTM6
KEGG_PATHWAY	Vitamin digestion and absorption	4	0.008954	CUBN, CBLIF, SLC23A1, PLB1
KEGG_PATHWAY	Drug metabolism - cytochrome P450	6	0.009169	ADH4, ADH1, MAOB, UGT2A3, GSTM7, GSTM6
KEGG_PATHWAY	Cholesterol metabolism	5	0.012556	ABCA1, NCEH1, ANGPTL4, CD36, LIPA
KEGG_PATHWAY	ABC transporters	5	0.014356	ABCA1, ABCD3, ABCC2, ABCA8A, ABCA8B

Table S4. Enriched gene clusters in the duodenum of PD-fed mice

A) The 20 most significant GO terms downregulated in the duodenum of PD-fed mice

Category	Term	Count	PValue	Genes	Fold Enrichment	FDR
GOTERM_BP_FAT	GO:0002682~regulation of immune system process	49	8.93E-08	FLT3, TMEM131L, SLA2, AQP3, MECOM, TRBC2, MYC, GPX2, TRIM62, PRKCH, EDN3, EREG, ZAP70, CD8A, KIT, CD226, DNASE1, TLR4, NBL1, SKAP1, SASH3, BLM, H2-DMA, 5730507C01RIK, CFI, DTX1, MPTX2, PLA2G5, REG3G, IL2RG, GRAMD4, CCL5, PCK1, CCL24, CAR2, FYB2, CDKN2A, TRAT1, BMX, MEIS2, H2-AA, MFHAS1, VNN1, SELL, KLRD1, CD247, KLRH1, ADA, H2-AB1	2.26468599	2.70E-05
GOTERM_BP_FAT	GO:0006629~lipid metabolic process	47	1.11E-09	SCARB1, AQP8, CYP3A11, MGST2, PLA2G5, SLA2, HSD17B11, AKR1B8, CYP17A1, CES2A, CES2B, HRH1, CES2C, CYP2C55, CYP2B10, PCK1, HAO2, ENPP7, GSTM4, GSTM2, GSTM1, UGT1A1, UGT2B38, CYP4A10, PLA2G4C, MBOAT1, ACOT12, PLAAT3, GPCPD1, BAAT, CYP2C29, CYP4F15, PLCB4, CYP2C66, ACOX2, GSTA3, CYP2C65, CES1F, GSTA1, KIT, CES1G, ALDH1A1, CYP1A1, UGT2B5, ACOT1, TLR4, ABCG1	2.676370387	5.85E-07
GOTERM_BP_FAT	GO:0006082~organic acid metabolic process	41	2.63E-10	CYP3A11, MGST2, GLYAT, PLA2G5, CES2A, CES2B, CES2C, CYP2C55, MYC, CYP2B10, SLC16A9, PCK1, HAO2, GSTM4, GSTM2, GSTM1, UGT1A1, BHMT1B, UGT2B38, CYP4A10, ACOT12, CTPS2, BAAT, CYP2C29, CYP4F15, VNN1, CYP2C66, ACOX2, TST, PSAT1, CYP2C65, CES1F, GSTA1, CES1G, ALDH1A1, CYP1A1, UGT2B5, SARDH, ACOT1, TLR4, ASPA	3.103500113	2.23E-07
GOTERM_BP_FAT	GO:0019752~carboxylic acid metabolic process	40	5.75E-11	CYP3A11, MGST2, GLYAT, PLA2G5, CES2A, CES2B, CES2C, CYP2C55, MYC, CYP2B10, PCK1, HAO2, GSTM4, GSTM2, GSTM1, UGT1A1, BHMT1B, UGT2B38, CYP4A10, ACOT12, CTPS2, BAAT, CYP2C29, CYP4F15, VNN1, CYP2C66, ACOX2, TST, PSAT1, CYP2C65, CES1F, GSTA1, CES1G, ALDH1A1, CYP1A1, UGT2B5, SARDH, ACOT1, TLR4, ASPA	3.334052439	8.88E-08
GOTERM_BP_FAT	GO:0043436~oxoacid metabolic process	40	6.17E-10	CYP3A11, MGST2, GLYAT, PLA2G5, CES2A, CES2B, CES2C, CYP2C55, MYC, CYP2B10, PCK1, HAO2, GSTM4, GSTM2, GSTM1, UGT1A1, BHMT1B, UGT2B38, CYP4A10, ACOT12, CTPS2, BAAT, CYP2C29, CYP4F15, VNN1, CYP2C66, ACOX2, TST, PSAT1, CYP2C65, CES1F, GSTA1, CES1G, ALDH1A1, CYP1A1, UGT2B5, SARDH, ACOT1, TLR4, ASPA	3.073075599	3.73E-07
GOTERM_BP_FAT	GO:0002684~positive regulation of immune system process	38	7.55E-07	SASH3, BLM, H2-DMA, CFI, MPTX2, PLA2G5, REG3G, SLA2, IL2RG, GRAMD4, AQP3, CCL5, TRBC2, PCK1, CCL24, CAR2, TRIM62, FYB2, PRKCH, EDN3, TRAT1, BMX, H2-AA, EREG, MFHAS1, ZAP70, VNN1, SELL, CD8A, KIT, KLRD1, CD226, CD247, TLR4, SKAP1, KLRH1, ADA, H2-AB1	2.431782131	2.00E-04
GOTERM_BP_FAT	GO:0044255~cellular lipid metabolic process	37	2.52E-08	SCARB1, CYP3A11, MGST2, PLA2G5, SLA2, AKR1B8, CES2A, CES2B, CES2C, CYP2C55, CYP2B10, PCK1, HAO2, ENPP7, GSTM4, GSTM2, GSTM1, CYP4A10, PLA2G4C, MBOAT1, ACOT12, PLAAT3, GPCPD1, BAAT, CYP2C29, CYP4F15, CYP2C66, ACOX2, CYP2C65, CES1F, GSTA1, KIT, CES1G, ALDH1A1, CYP1A1, ACOT1, TLR4	2.840097043	8.20E-06
GOTERM_BP_FAT	GO:0032787~monocarboxylic acid metabolic process	35	3.61E-12	CYP3A11, MGST2, GLYAT, PLA2G5, CES2A, CES2B, CES2C, CYP2C55, MYC, CYP2B10, PCK1, HAO2, GSTM4, GSTM2, GSTM1, UGT1A1, UGT2B38, CYP4A10 [illegible]	4.153975662	1.53E-08
GOTERM_BP_FAT	GO:0006631~fatty acid metabolic process	26	4.60E-10	MGST2, PLA2G5, CES2A, CES2B, CES2C, CYP2C55, CYP2B10, PCK1, HAO2, GSTM4, GSTM2, GSTM1, CYP4A10, ACOT12, BAAT, CYP2C29, CYP4F15, CYP2C66, ACOX2, CYP2C65, CES1F, GSTA1, CES1G, CYP1A1, ACOT1, TLR4	4.616171792	3.25E-07
GOTERM_BP_FAT	GO:0009410~response to xenobiotic stimulus	22	8.07E-07	GSTM4, GSTM3, GSTM2, BLM, GSTM1, UGT1A1, CDKN2A, UGT2B38, PDX1, CYP3A11, CYP2C29, GSTA5, CYP2C66, CYP2C55, GSTA3, CYP2C65, CYP2B10, MYC, GSTA1, ALDH1A1, CYP1A1, UGT2B5	3.625939295	2.01E-04
GOTERM_BP_FAT	GO:0071466~cellular response to xenobiotic stimulus	19	1.06E-10	GSTM4, GSTM3, GSTM2, BLM, GSTM1, UGT1A1, UGT2B38, CYP3A11, CYP2C29, GSTA5, CYP2C66, CYP2C55, GSTA3, CYP2C65, CYP2B10, MYC, GSTA1, CYP1A1, UGT2B5	7.40933618	1.12E-07
GOTERM_BP_FAT	GO:0008202~steroid metabolic process	17	5.41E-06	SCARB1, UGT1A1, UGT2B38, AQP8, CYP3A11, HSD17B11, BAAT, CYP17A1, HRH1, ACOX2, CYP2B10, CES1F, KIT, CES1G, CYP1A1, UGT2B5, ABCG1	4.035290643	0.00127089
GOTERM_BP_FAT	GO:0006805~xenobiotic metabolic process	16	6.29E-11	GSTM4, GSTM2, GSTM1, UGT1A1, UGT2B38, CYP3A11, CYP2C29, GSTA5, CYP2C66, CYP2C55, GSTA3, CYP2C65, CYP2B10, GSTA1, CYP1A1, UGT2B5	10.05492649	8.88E-08
GOTERM_BP_FAT	GO:0033559~unsaturated fatty acid metabolic process	16	1.64E-09	GSTM2, GSTM1, CYP4A10, MGST2, PLA2G5, CYP2C29, CES2A, CYP4F15, CES2B, CES2C, CYP2C66, CYP2C55, CYP2C65, CYP2B10, GSTA1, TLR4	7.986484472	7.71E-07
GOTERM_BP_FAT	GO:0030217~T cell differentiation	16	7.00E-06	SASH3, BLM, CDKN2A, FLT3, H2-DMA, DTX1, CD3G, TMEM131L, IL2RG, H2-AA, ZAP70, VNN1, CD8A, KIT, PCK1, ADA	4.197101449	0.00156025
GOTERM_BP_FAT	GO:0006690~icosanoid metabolic process	15	2.70E-09	GSTM1, CYP4A10, MGST2, PLA2G5, CYP2C29, CES2A, CYP4F15, CES2B, CES2C, CYP2C66, CYP2C55, CYP2C65, CYP2B10, GSTA1, TLR4	8.508328628	1.13E-06
GOTERM_BP_FAT	GO:1901568~fatty acid derivative metabolic process	15	2.94E-09	GSTM1, CYP4A10, MGST2, PLA2G5, CYP2C29, CES2A, CYP4F15, CES2B, CES2C, CYP2C66, CYP2C55, CYP2C65, CYP2B10, GSTA1, TLR4	8.453436185	1.13E-06
GOTERM_BP_FAT	GO:0001676~long-chain fatty acid metabolic process	12	3.99E-07	GSTM4, CYP4F15, GSTM2, GSTM1, CYP2C66, CYP2C55, CYP2C65, CYP2B10, CYP4A10, CYP1A1, ACOT1, CYP2C29	7.822582738	1.13E-04
GOTERM_BP_FAT	GO:0042178~xenobiotic catabolic process	9	3.55E-09	GSTM4, GSTM2, GSTA5, GSTM1, UGT1A1, CYP2B10, GSTA1, CYP1A1, CYP3A11	23.12263427	1.25E-06
GOTERM_BP_FAT	GO:0019755~one-carbon compound transport	6	8.12E-06	CAR2, AQP8, AQP7, AQP3, AQP1, CAR4	20.96452174	0.00171882

B) The 20 most significant pathways downregulated in the duodenum of PD-fed mice

Category	Pathway	Count	PValue	Genes	Fold Enrichment	FDR
KEGG_PATHWAY	mmu05204:Chemical carcinogenesis - DNA adducts	17	4.47E-14	GSTM4, GSTM3, GSTM2, GSTM1, UGT1A1, UGT2B38, CYP3A11, MGST2, CYP2C29, GSTA5, CYP2C66, CYP2C55, GSTA3, CYP2C65, GSTA1, CYP1A1, UGT2B5	13.80987654	1.05E-11
KEGG_PATHWAY	mmu00983:Drug metabolism - other enzymes	16	3.16E-12	GSTM4, GSTM3, GSTM2, GSTM1, UGT1A1, UGT2B38, MGST2, TYMP, CES2A, CES2B, CES2C, GSTA5, GSTA3, CES1F, GSTA1, UGT2B5	11.86731079	3.70E-10
KEGG_PATHWAY	mmu00980:Metabolism of xenobiotics by cytochrome P450	12	6.74E-09	GSTM4, GSTM3, GSTM2, GSTA5, GSTM1, UGT1A1, GSTA3, UGT2B38, GSTA1, CYP1A1, UGT2B5, MGST2	11.21704718	5.26E-07
KEGG_PATHWAY	mmu05207:Chemical carcinogenesis - receptor activation	18	2.40E-08	GSTM4, GSTM3, GSTM2, PAQR7, GSTM1, UGT1A1, PAQR5, UGT2B38, ADCY4, CYP3A11, MGST2, GSTA5, GSTA3, CYP2B10, MYC, GSTA1, CYP1A1, UGT2B5	5.458962963	1.41E-06
KEGG_PATHWAY	mmu00982:Drug metabolism - cytochrome P450	11	6.58E-08	GSTM4, GSTM3, GSTM2, GSTA5, GSTM1, UGT1A1, GSTA3, UGT2B38, GSTA1, UGT2B5, MGST2	10.57193532	3.08E-06
KEGG_PATHWAY	mmu00140:Steroid hormone biosynthesis	12	9.03E-08	CYP2C66, UGT1A1, CYP2C55, CYP2C65, CYP2B10, UGT2B38, CYP1A1, CYP3A11, UGT2B5, HSD17B11, CYP2C29, CYP17A1	8.804778973	3.47E-06
KEGG_PATHWAY	mmu01100:Metabolic pathways	50	1.04E-07	CYP3A11, AKR1B8, HYKK, MECOM, CYP2B10, ENPP7, CAR4, GPX2, UGT1A1, CYP4A10, PLA2G4C, ACOT12, CTPS2, PLAAT3, BAAT, PLCB4, ACOX2, TST, B3GNT6, ALDH1A1, UGT2B5, ACOT1, ASPA, LIPT2, ADCY4, MGST2, PLA2G5, TYMP, CYP17A1, CYP2C55, PCK1, HAO2, GSTM4, GSTM3, CAR2, GSTM2, GSTM1, UGT2B38, MBOAT1, CYP2C29, GSTA5, VNN1, CYP2C66, PSAT1, GSTA3, CYP2C65, GSTA1, CYP1A1, SARDH, ADA	2.107382243	3.47E-06
KEGG_PATHWAY	mmu00830:Retinol metabolism	12	1.40E-07	CYP2C66, UGT1A1, CYP2C55, CYP2C65, CYP2B10, CYP4A10, UGT2B38, ALDH1A1, CYP1A1, CYP3A11, UGT2B5, CYP2C29	8.441695304	4.10E-06
KEGG_PATHWAY	mmu00591:Linoleic acid metabolism	8	6.99E-06	CYP2C66, CYP2C55, CYP2C65, PLA2G4C, CYP3A11, PLAAT3, PLA2G5, CYP2C29	10.91792593	1.82E-04
KEGG_PATHWAY	mmu00480:Glutathione metabolism	9	8.90E-06	GSTM4, GSTM3, GPX2, GSTM2, GSTA5, GSTM1, GSTA3, GSTA1, MGST2	8.52962963	2.08E-04
KEGG_PATHWAY	mmu01524:Platinum drug resistance	9	1.95E-05	GSTM4, GSTM3, GSTM2, GSTA5, GSTM1, CDKN2A, GSTA3, GSTA1, MGST2	7.676666667	4.16E-04
KEGG_PATHWAY	mmu00590:Arachidonic acid metabolism	9	3.05E-05	CYP2C66, CYP2C55, CYP2C65, CYP2B10, CYP4A10, PLA2G4C, PLAAT3, PLA2G5, CYP2C29	7.225098039	5.95E-04
KEGG_PATHWAY	mmu04750:Inflammatory mediator regulation of TRP channels	10	9.27E-05	HRH1, PRKCH, PLCB4, CYP2C66, CYP2C55, CYP2C65, CYP4A10, PLA2G4C, ADCY4, CYP2C29	5.372995042	0.00164431
KEGG_PATHWAY	mmu04976:Bile secretion	9	9.84E-05	SCARB1, CAR2, UGT1A1, AQP8, UGT2B38, UGT2B5, ADCY4, BAAT, AQP1	6.141333333	0.00164431
KEGG_PATHWAY	mmu05225:Hepatocellular carcinoma	11	2.16E-04	GSTM4, GSTM3, GSTM2, FZD2, GSTA5, GSTM1, CDKN2A, GSTA3, MYC, GSTA1, MGST2	4.313835675	0.00337247
KEGG_PATHWAY	mmu04964:Proximal tubule bicarbonate reclamation	5	2.55E-04	CAR2, SLC38A3, PCK1, AQP1, CAR4	15.50841751	0.00373665
KEGG_PATHWAY	mmu04640:Hematopoietic cell lineage	8	4.21E-04	CD8A, FLT3, H2-DMA, CD7, KIT, CD3G, H2-AA, H2-AB1	5.807407407	0.00579787
KEGG_PATHWAY	mmu04658:Th1 and Th2 cell differentiation	7	0.00172961	ZAP70, H2-DMA, CD3G, CD247, IL2RG, H2-AA, H2-AB1	5.427946128	0.02164754
KEGG_PATHWAY	mmu05340:Primary immunodeficiency	5	0.00175771	ZAP70, CIITA, CD8A, IL2RG, ADA	9.477366255	0.02164754
KEGG_PATHWAY	mmu04913:Ovarian steroidogenesis	6	0.00216135	SCARB1, PLA2G4C, CYP1A1, ADCY4, ACOT1, CYP17A1	6.498765432	0.02451721

C) The 20 most significant GO terms upregulated in the duodenum of PD-fed mice

Category	Term	Count	PValue	Genes	Fold Enrichment	FDR
GOTERM_BP_FAT	GO:0048585~negative regulation of response to stimulus	38	4.02E-05	RIPOR1, NLRX1, SERPINE2, INSIG1, USP18, LTBP1, ARHGAP35, SLC6A3, RGS4, RGS5, IL1RL1, GJA1, RNF213, TKFC, FGF9, PIP4K2B, CD34, WNT3, GBP3, DUSP3, HEG1, FST, APOA2, WTIP, DUSP8, MMP9, TBX2, ENTREP1, SFRP1, SLC6A9, ERCC4, WNK2, SPRY1, RGS7BP, SH2D1B1, FBP1, H2-Q10, PPARD	2.02723845	0.16669511

GOTERM_BP_FAT	GO:0051240~positive regulation of multicellular organismal process	41	9.45E-05	NLRX1, BEX1, SERPINE2, CLEC4N, AMIGO2, NOD1, HAPLN4, LTBP1, ARHGAP35, SLC6A3, BICRA, RGS4, IL1RL1, GJA1, C6, TKFC, FGF9, P2RY2, SH3PXD2B, ENPP4, CD34, WNT3, PDGFRB, CADM1, TNFRSF12A, TRPA1, GP1BB, TNFSF15, HEG1, FST, APOA2, ACSL6, NPNT, MMP9, ZDHHC15, TBX2, ID2, SPRY1, TEK, H2-Q10, TLR2	1.88066438	0.16761821
GOTERM_BP_FAT	GO:0065009~regulation of molecular function	47	1.48E-04	RIPOR1, PARM1, SERPINE2, TRIM30C, RASL2-9, DCUN1D2, NOD1, NTS, SLC5A3, ARHGAP35, IFIT2, RGS4, DUSP12, GJA1, CLTRN, PIP4K2B, RALGPS1, TBC1D16, PDGFRB, R3HDML, DUSP3, TNFSF15, HEG1, APOA2, TRAPPC9, NPNT, MMP9, RENBP, SLC8A3, SGSM1, SFRP1, FABP4, SLC6A9, ERCC4, WNK2, ID2, NOS1AP, ATP13A2, NAF1, SPRY1, ALDOB, TEK, DCP1A, PLCD1, PPARD, TLR2, HCN1	1.74156584	0.16761821
GOTERM_BP_FAT	GO:0031349~positive regulation of defense response	16	1.62E-04	NLRX1, CADM1, CLEC4N, TRIM30C, TRIL, NOD1, IGHG2B, IL1RL1, GJA1, TKFC, FABP4, EPG5, SH2D1B1, H2-Q10, TLR2, GBP3	3.18273267	0.16761821
GOTERM_BP_FAT	GO:0010038~response to metal ion	14	3.35E-04	CPNE8, DNMT3A, MT2, MMP9, SLC6A3, FABP4, AKR1C14, ID2, ATP13A2, ALDOB, SLC25A23, FBP1, PLCD1, HCN1	3.30910588	0.20059627
GOTERM_BP_FAT	GO:0051050~positive regulation of transport	25	4.60E-04	RIPOR1, RASL2-9, SLC2A2, PTPN23, ERFE, GJA1, CLTRN, KIF3A, P2RY2, CD34, PDGFRB, TRPA1, ABCA8A, APOA2, ACSL6, IGHG2B, SLC6A9, WNK2, IAPP, ATP13A2, TEK, PLCD1, PPARD, TLR2, HCN1	2.18190704	0.20059627
GOTERM_BP_FAT	GO:0048584~positive regulation of response to stimulus	46	4.64E-04	H2-T24, NLRX1, CLEC4N, TRIM30C, TRIL, SLC2A2, NOD1, NTS, NID1, ERFE, RGS4, IL1RL1, GJA1, C6, TKFC, FGF9, EPG5, IGLC1, ENPP4, WNT3, GBP3, EDARADD, PDGFRB, GUCY1A1, DUSP3, CADM1, TNFRSF12A, TRPA1, GP1BB, TNFSF15, DNMT3A, NPNT, MMP9, IGHG2B, SFRP1, FABP4, CFHR2, WNK2, AKR1C14, IAPP, SH2D1B1, TEK, H2-Q10, PPARD, TLR2, C1QC	1.66941113	0.20059627
GOTERM_BP_FAT	GO:0003013~circulatory system process	16	4.88E-04	GUCY1A1, HEG1, SLC2A5, NTS, ARHGAP35, TBX2, RGS4, GJA1, TMEM65, P2RY2, ID2, NOS1AP, E2F4, CD34, PPARD, HCN1	2.86502674	0.20059627
GOTERM_BP_FAT	GO:0015711~organic anion transport	15	5.42E-04	SLC10A2, ACSL6, APOA2, SLC2A2, PCTP, ERFE, SLC16A13, RGS4, GJA1, FABP4, SLC6A9, CLTRN, P2RY2, SLC25A23, PPARD	2.97791502	0.20059627
GOTERM_BP_FAT	GO:0032101~regulation of response to external stimulus	22	6.65E-04	PDGFRB, NLRX1, DUSP3, SERPINE2, GP1BB, TRIM30C, NOD1, USP18, SLC6A3, IGHG2B, IL1RL1, GJA1, C6, TKFC, FABP4, CFHR2, ENPP4, CD34, WNT3, H2-Q10, PPARD, TLR2	2.27601442	0.20059627
GOTERM_BP_FAT	GO:0006000~fructose metabolic process	4	7.00E-04	ALDOART1, TKFC, ALDOB, FBP1	22.3233333	0.20059627
GOTERM_BP_FAT	GO:0023051~regulation of signaling	58	7.04E-04	RIPOR1, SERPINE2, CLEC4N, TRIM30C, SLC2A2, PTPN23, ERFE, ARHGAP35, RGS4, RGS5, GJA1, FGF9, PIP4K2B, RALGPS1, PDGFRB, EDARADD, GUCY1A1, DUSP3, TNFRSF12A, HEG1, FST, DUSP8, NPNT, MMP9, SFRP1, AKR1C14, IAPP, RGS7BP, SH2D1B1, FBP1, TLR2, PPARD, NRN1, NLRX1, INSIG1, NOD1, NID1, NTS, CPLX2, USP18, LTBP1, RNF213, TKFC, TMEM65, P2RY2, WNT3, TBC1D16, GBP3, TRPA1, TNFSF15, WTIP, SLC8A3, ENTREP1, SLC6A9, WNK2, SPRY1, TEK, HCN1	1.51689404	0.20059627
GOTERM_BP_FAT	GO:0006820~anion transport	17	7.09E-04	TRPA1, SLC10A2, ACSL6, ANO8, APOA2, SLC2A2, PCTP, ERFE, SLC16A13, RGS4, GJA1, FABP4, SLC6A9, CLTRN, P2RY2, SLC25A23, PPARD	2.65176242	0.20059627
GOTERM_BP_FAT	GO:0045089~positive regulation of innate immune response	12	7.48E-04	NLRX1, TKFC, CADM1, CLEC4N, TRIM30C, TRIL, EPG5, NOD1, SH2D1B1, H2-Q10, TLR2, GBP3	3.45404012	0.20059627
GOTERM_BP_FAT	GO:0001501~skeletal system development	16	8.38E-04	PDGFRB, FBXW4, RARG, CADM1, GP1BB, FST, INSIG1, HAPLN4, MMP9, GJA1, SFRP1, FGF9, BGLAP3, SH3PXD2B, POC1A, PLS3	2.71959391	0.20059627
GOTERM_BP_FAT	GO:0044724~single-organism carbohydrate catabolic process	8	9.46E-04	MGAM, ALDOART1, G6PC, TKFC, ALDOB, SIS, FBP1, XYLB	5.15153846	0.20059627
GOTERM_BP_FAT	GO:0031347~regulation of defense response	20	9.52E-04	NLRX1, CADM1, CLEC4N, TRIM30C, TRIL, NOD1, USP18, IGHG2B, IL1RL1, GJA1, C6, TKFC, FABP4, CFHR2, EPG5, SH2D1B1, H2-Q10, PPARD, TLR2, GBP3	2.33344948	0.20059627
GOTERM_BP_FAT	GO:0009968~negative regulation of signal transduction	27	9.64E-04	RIPOR1, NLRX1, SERPINE2, INSIG1, USP18, LTBP1, ARHGAP35, RGS4, RGS5, RNF213, TKFC, FGF9, PIP4K2B, GBP3, DUSP3, HEG1, FST, WTIP, DUSP8, MMP9, ENTREP1, SFRP1, SLC6A9, WNK2, SPRY1, RGS7BP, FBP1	1.99432721	0.20059627
GOTERM_BP_FAT	GO:0010648~negative regulation of cell communication	29	0.0010116	RIPOR1, NLRX1, SERPINE2, INSIG1, USP18, LTBP1, ARHGAP35, RGS4, RGS5, GJA1, RNF213, TKFC, FGF9, PIP4K2B, GBP3, DUSP3, HEG1, FST, WTIP, DUSP8, MMP9, ENTREP1, SFRP1, SLC6A9, WNK2, SPRY1, RGS7BP, FBP1, HCN1	1.92417107	0.20059627
GOTERM_BP_FAT	GO:0023057~negative regulation of signaling	29	0.0010528	RIPOR1, NLRX1, SERPINE2, INSIG1, USP18, LTBP1, ARHGAP35, RGS4, RGS5, GJA1, RNF213, TKFC, FGF9, PIP4K2B, GBP3, DUSP3, HEG1, FST, WTIP, DUSP8, MMP9, ENTREP1, SFRP1, SLC6A9, WNK2, SPRY1, RGS7BP, FBP1, HCN1	1.91910079	0.20059627

D) The 20 most significant pathways upregulated in the duodenum of PD-fed mice

Category	Pathway	Count	PValue	Genes	Fold Enrichment	FDR
KEGG_PATHWAY	mmu04973:Carbohydrate digestion and absorption	5	0.00182973	MGAM, G6PC, SLC2A2, SLC2A5, SIS	9.407679739	0.36594534
KEGG_PATHWAY	mmu00051:Fructose and mannose metabolism	4	0.00702654	ALDOART1, TKFC, ALDOB, FBP1	10.03485839	0.70265411
KEGG_PATHWAY	mmu00010:Glycolysis / Gluconeogenesis	4	0.03698516	ALDOART1, G6PC, ALDOB, FBP1	5.391864208	1
KEGG_PATHWAY	mmu00052:Galactose metabolism	3	0.04770677	MGAM, G6PC, SIS	8.466911765	1
KEGG_PATHWAY	mmu00030:Pentose phosphate pathway	3	0.05042991	ALDOART1, ALDOB, FBP1	8.210338681	1
KEGG_PATHWAY	mmu00500:Starch and sucrose metabolism	3	0.05320787	MGAM, G6PC, SIS	7.968858131	1
KEGG_PATHWAY	mmu01100:Metabolic pathways	25	0.06919559	LIPT1, ALDOART1, UAP1, NPL, TKFC, DSEL, ENPP4, PIP4K2B, XYLB, MGAM, GUCY	1.39459119	1
KEGG_PATHWAY	mmu03320:PPAR signaling pathway	4	0.07398627	FABP4, ACSL6, APOA2, PPARD	4.059043842	1
KEGG_PATHWAY	mmu04610:Complement and coagulation cascades	4	0.08404812	C6, SERPINE2, CFHR2, C1QC	3.843137255	1
