# Supplementary material for: A purified diet affects intestinal epithelial proliferation and barrier functions through gut microbial alterations
Source: Int Immunol. 2024 Jan 23;36(5):223–40. doi: 10.1093/intimm/dxae003 (PMC10989658; doi:10.1093/intimm/dxae003)
Supplement: dxae003_suppl_Supplementary_Figures [file dxae003_suppl_supplementary_figures.pdf]

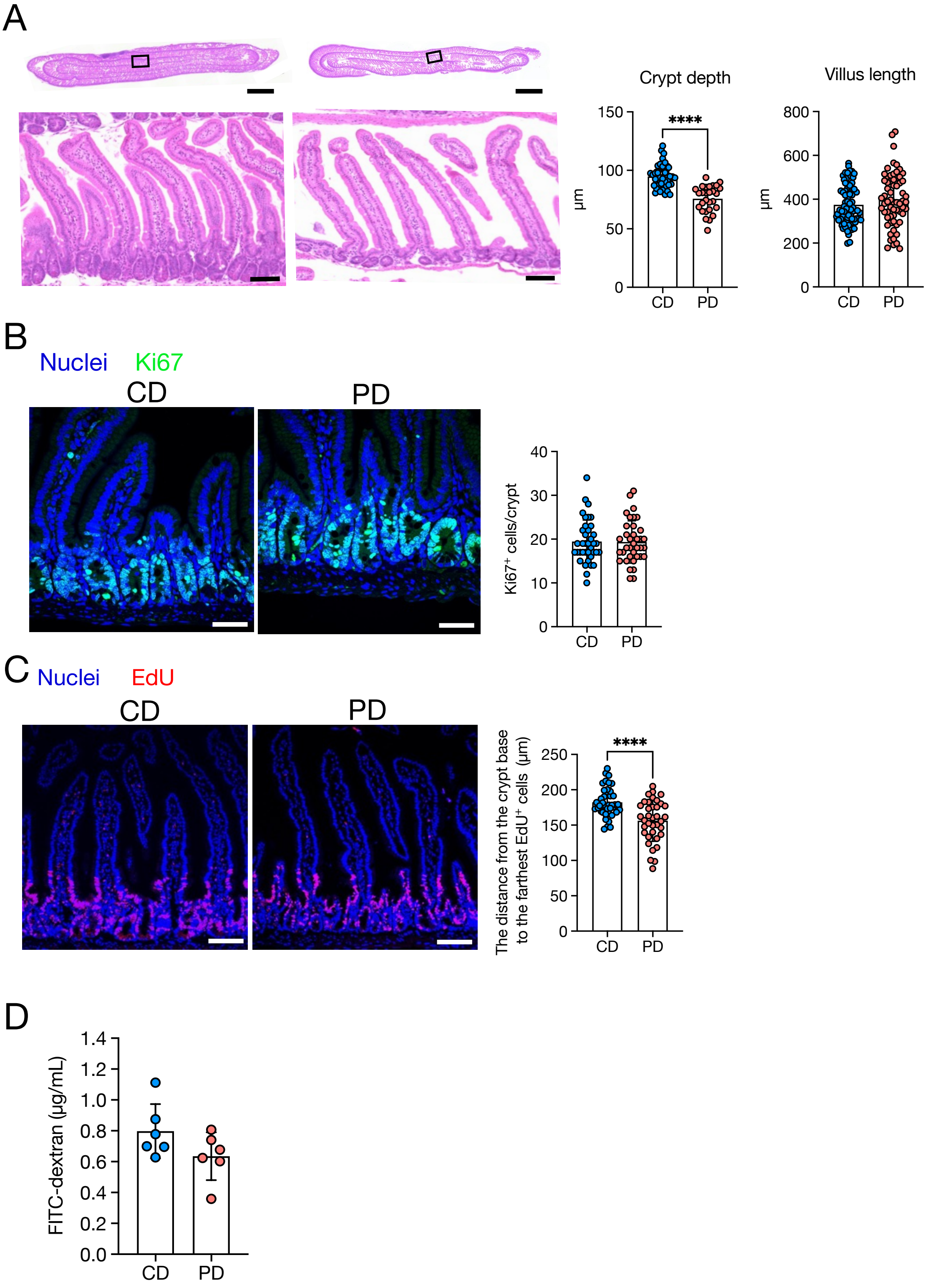

Supplementary Figure 1 | The impacts of PD on the duodenal epithelium

(A) Representative images of hematoxylin and eosin (H&E)-staining and crypt depth and villus length of the duodenum. Images of the Swiss roll-like sections (top) and ileal epithelium (bottom). Scale bars: 2,000 μm (top) or 100 μm (bottom). $n \geq 25$ crypt regions from 4 individual mice per group were analyzed for crypt depth. $n \geq 60$ villi regions from 4 individual mice per group were analyzed for villus length. (B) Representative immunofluorescent images of Ki67 and the number of $Ki67^{+}$ cells in the duodenal crypts. Scale bars: 50 μm. $n \geq 35$ crypt regions from 4 individual mice per group were analyzed. (C) Representative images of EdU and distance from the crypt base to the farthest EdU-labeled cells in the duodenum. $n \geq 35$ crypt-villi regions from 3 individual mice per group were analyzed. (D) The fluorescent intensity of FITC-dextran was measured in the plasma after feeding CD or PD for three weeks. The data represent the mean ± SD. The data represent the mean ± SD. ****$p < 0.0001$. p values were determined by unpaired *t*-test. CD: crude diet, PD: purified diet.

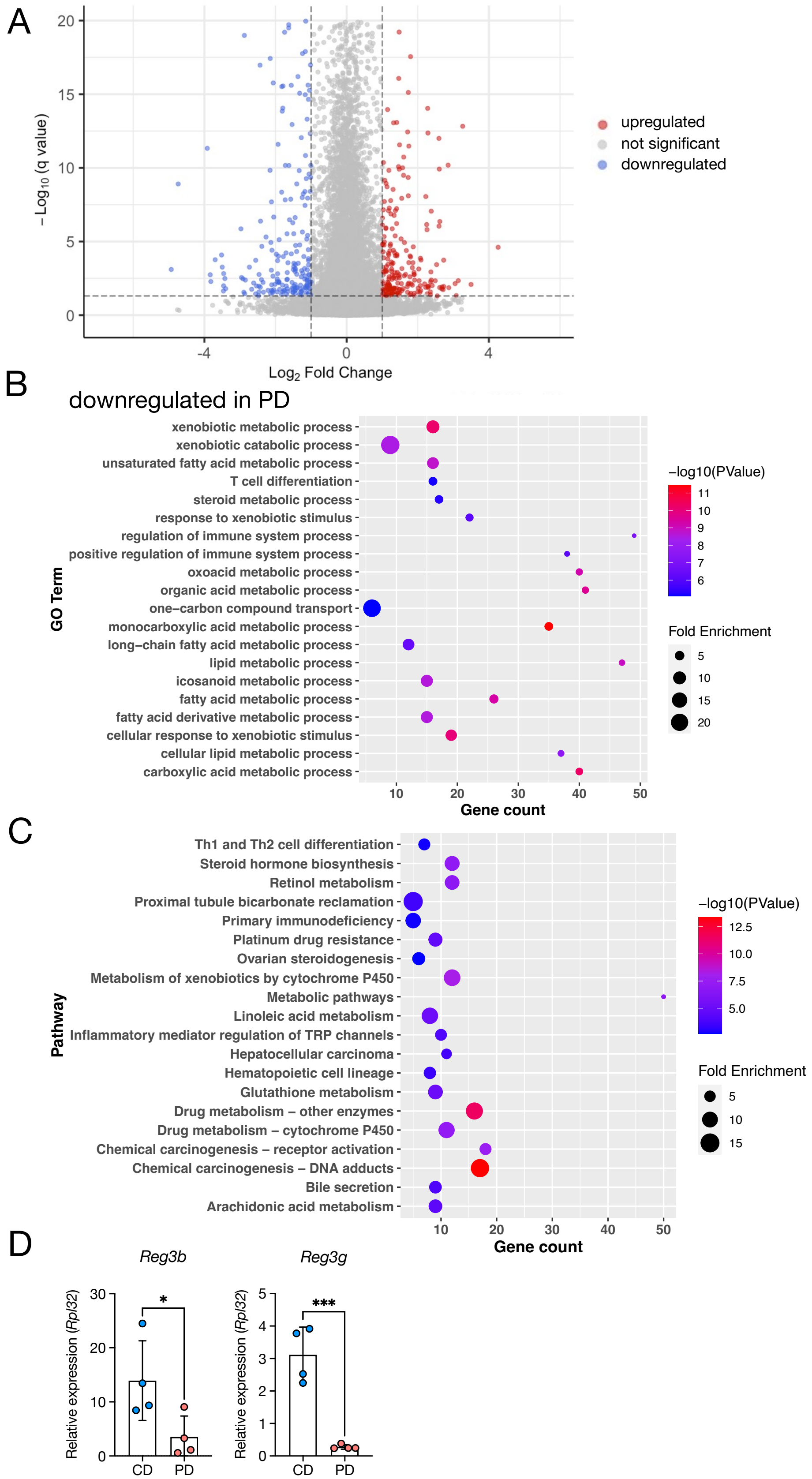

Supplementary Figure 2 | Global changes in gene expression and genes downregulated by PD in the duodenal epithelium.

(A) Volcano plots for the comparison of duodenal epithelium from CD- versus PD-fed mice based on RNA-sequencing data. $n = 6$. Genes up- or down-regulated (Fold change > 2 or q value < 0.05) are highlighted. (B, C) GO enrichment analysis (B) and KEGG pathway analysis (C) of downregulated genes in PD-fed mice. The 20 most significant GO terms are represented in the accompanying bubble plot. Bubble colors represent -$\log_{10}$ (p values). Bubble sizes indicate fold enrichment. (D) Relative mRNA expression of *Reg3b* and *Reg3g* in the duodenal epithelium from CD- and PD-fed mice. $n = 4$ mice per group. The data represent the mean ± SD. *p < 0.05, **p < 0.01, ***p < 0.001. p values were determined by unpaired t-test. CD: crude diet, PD: purified diet.

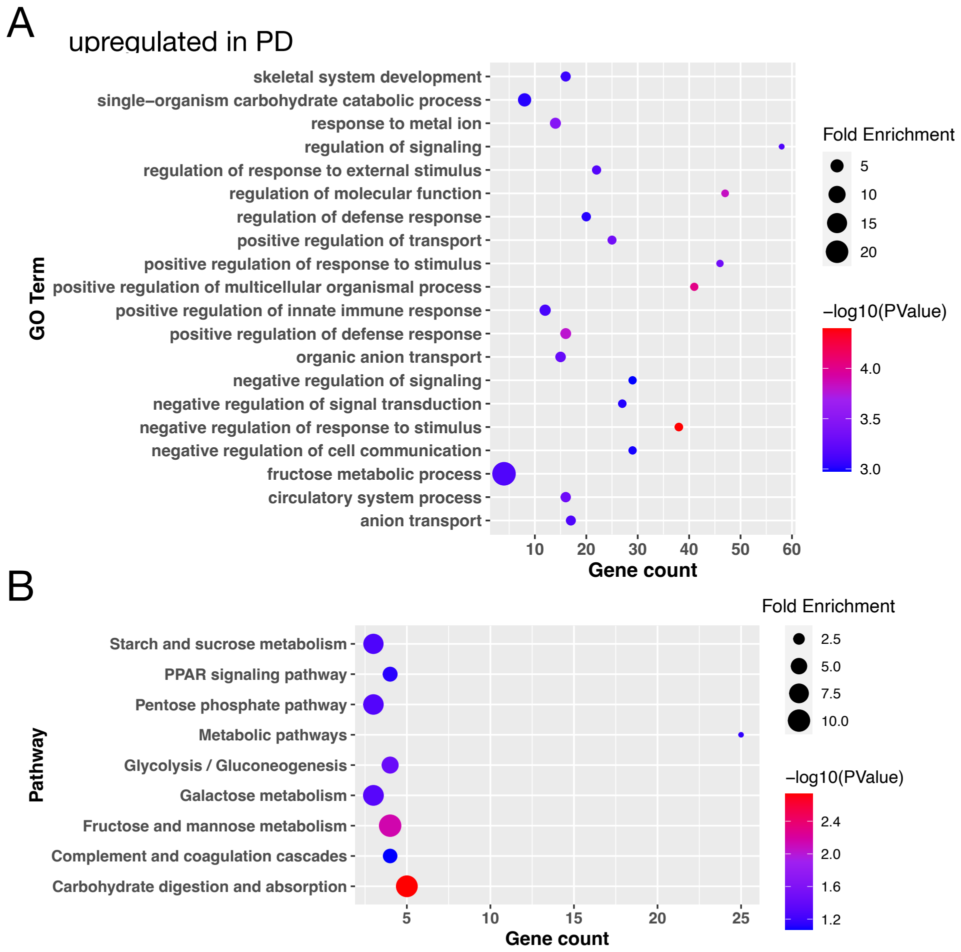

Supplementary Figure 3 | Genes upregulated by PD in the duodenal epithelium

(A, B) GO enrichment analysis (A) and KEGG pathway analysis (B) of upregulated genes in PD-fed mice. The 20 most significant GO terms are represented in the accompanying bubble plot. Bubble colors represent -$\log_{10}$ (p values). Bubble sizes indicate fold enrichment.

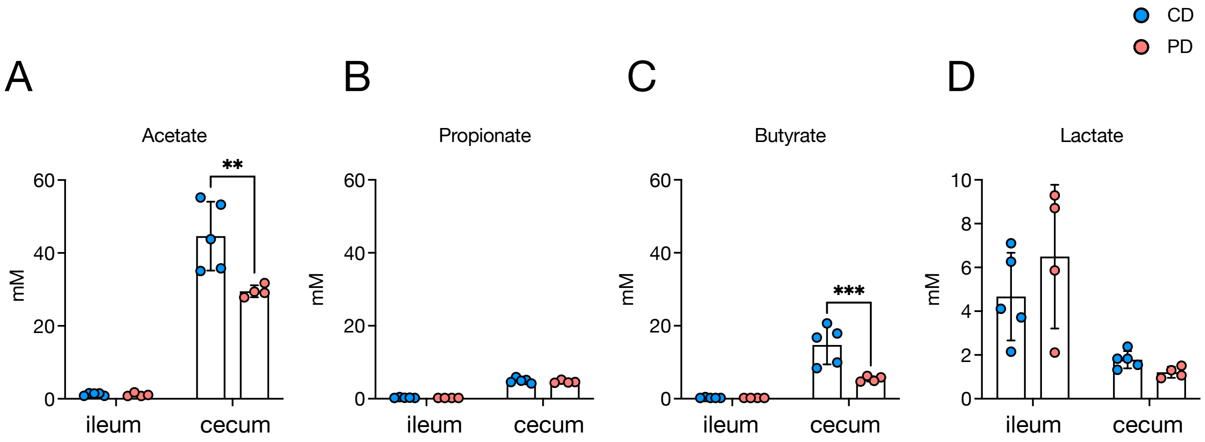

Supplementary Figure 4 | The effect of PD on luminal concentration of organic acids

. Amounts of organic acids in the small intestinal and cecal contents from CD- and PD-fed mice were measured by GC-MS. (A-D) Concentrations of Acetate (A), Propionate (B), Butyrate (C), and Lactate (D). $n >$ 4 mice per group. The data represent the mean ± SD. **$p < 0.01$, ***$p < 0.001$. p values were determined by two-way ANOVA followed by Šídák's multiple comparison test. CD: crude diet, PD: purified diet.

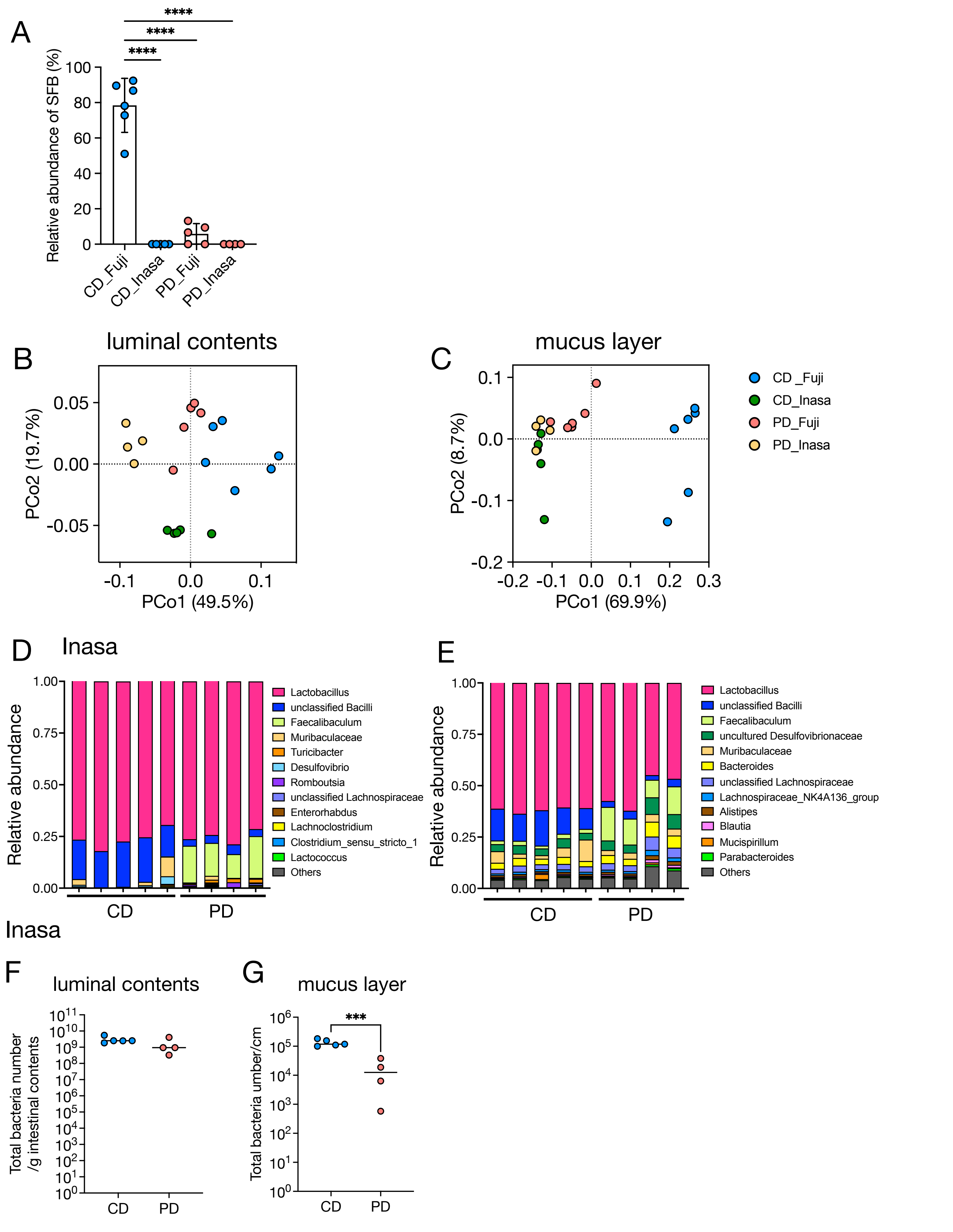

Supplementary Figure 5 | Gut microbiota changes in Inasa mice

(A) Relative abundance of SFB in the ileal mucus layers of Fuji and Inasa mice. (B , C) Principal coordinate analysis of weighted UniFrac distances among the microbiota in the ileal contents (B) and ileal mucus layer (C). (D, E) Bacterial composition of the microbiota at the genus level in the ileal contents (D) and ileal mucus layer (E). (F, G) The total bacterial load in the ileal contents (F) and ileal mucus layer (G) was analyzed by qPCR using universal primers for the 16S rRNA V3-V4 region. The data represent the mean ± SD. ***$p <$ 0.001, ****$p < 0.0001$. p values were determined by unpaired *t*-test. CD: crude diet, PD: purified diet.

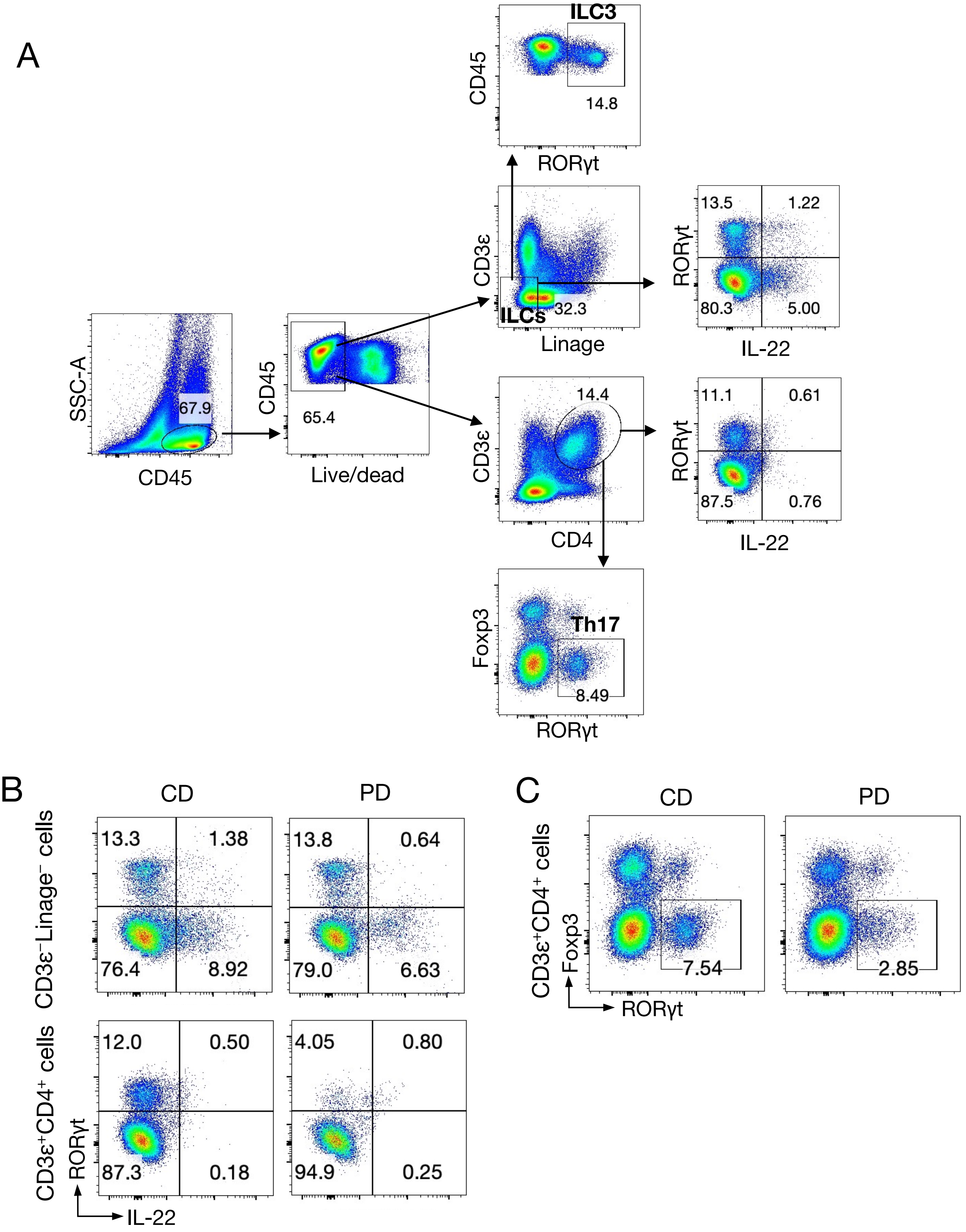

Supplementary Figure 6 | The influence of PD on ILCs and Th17 cells

(A) Gating strategy of ILCs and Th17 cells. Linage: CD8α, CD11c, B220, F4/80 (B) Representative flow cytometry plots of IL-22 and RORγt gated on $CD3\varepsilon^{-}$linage^{-} cells (upper) and $CD3^{+}CD4^{+}$ cells (lower). (C) Representative flow cytometry plots of Th17 cells (RORγt^{+}Foxp3^{-} cells) gated on $CD4^{+}$ T ($CD3\varepsilon^{+}CD4^{+}$ cells). CD: crude diet, PD: purified diet.
